# Supplementary material for: Investigation of a herpesvirus outbreak in mixed breeds of adult domestic ducks using next generation sequencing
Source: PLoS One. 2023 Jan 27;18(1):e0280923. doi: 10.1371/journal.pone.0280923 (PMC9882916; doi:10.1371/journal.pone.0280923)
Supplement: S2 File — (PDF) [file pone.0280923.s002.pdf]

Qualimap Report: BAM QC

HHV-6 vs GaHV-2 NGS
Mapping Quality Score Report

CONTENTS

- Input data & parameters
- Summary
- Coverage across reference
- Coverage Histogram
- Coverage Histogram (0-50X)
- Genome Fraction Coverage
- Duplication Rate Histogram
- Mapped Reads Nucleotide Content
- Mapped Reads GC-content Distribution
- Homopolymer Indels
- Mapping Quality Across Reference
- Mapping Quality Histogram
- Insert Size Across Reference
- Insert Size Histogram

Input data and parameters

QualiMap command line

```
qualimap bamqc -bam Bowtie2_on_data_6__data_4__and_data_3__alignments -c -nw 400 -hm 3 -sd
```

Alignment

Command line:	"/mnt/pulsar/deps/_conda/envs/mulled-v1-2c9d0d73d81f016cd458c97e185f638410841f3ecf7ae79158d90e0d58249513/bin/bowtie2-align-s --wrapper basic-0 -p 8 -x genome --passthrough -1 input_f.fastq.gz -2 input_r.fastq.gz"
Draw chromosome limits:	yes
Analyze overlapping paired-end reads:	yes
Program:	bowtie2 (2.4.2)
Analysis date:	Thu Dec 09 05:25:50 UTC 2021
Size of a homopolymer:	3
Skip duplicate alignments:	yes (only flagged)
Number of windows:	400
BAM file:	Bowtie2_on_data_6__data_4__and_data_3__alignments

Summary

Warnings

No flagged duplicates are detected	Make sure duplicate alignments are flagged in the BAM file or apply a different skip duplicates mode.

Globals

Reference size	170,950
Number of reads	224,352,814
Mapped reads	523,483 / 0.23%
Unmapped reads	223,829,331 / 99.77%
Mapped paired reads	523,483 / 0.23%
Mapped reads, first in pair	264,478 / 0.12%
Mapped reads, second in pair	259,005 / 0.12%

Mapped reads, both in pair	475,940 / 0.21%
Mapped reads, singletons	47,543 / 0.02%
Secondary alignments	0
Read min/max/mean length	151 / 151 / 151
Overlapping read pairs	235,096 / 0.21%
Duplicated reads (flagged)	0 / 0%
Duplicated reads (estimated)	522,415 / 0.23%
Duplication rate	54.49%
Clipped reads	0 / 0%

ACGT Content

Number/percentage of A's	19,765,452 / 25.46%
Number/percentage of C's	18,480,034 / 23.81%
Number/percentage of T's	19,001,607 / 24.48%
Number/percentage of G's	20,379,942 / 26.25%
Number/percentage of N's	649 / 0%
GC Percentage	50.06%

Coverage

Mean	454.7151
Standard Deviation	9,098.4215
Mean (paired-end reads overlap ignored)	295.13

Mapping Quality

Mean Mapping Quality	1.17

Insert size

Mean	180.87
Standard Deviation	28.73
P25/Median/P75	159 / 172 / 197

Mismatches and indels

General error rate	6.83%
Mismatches	3,892,954
Insertions	466,835
Mapped reads with at least one insertion	52.39%
Deletions	92,190
Mapped reads with at least one deletion	15.11%
Homopolymer indels	59.96%

Chromosome stats

Name	Length	Mapped bases	Mean coverage	Standard deviation
Gallid	170950	77733544	454.7151	9,098.4215

Coverage across reference

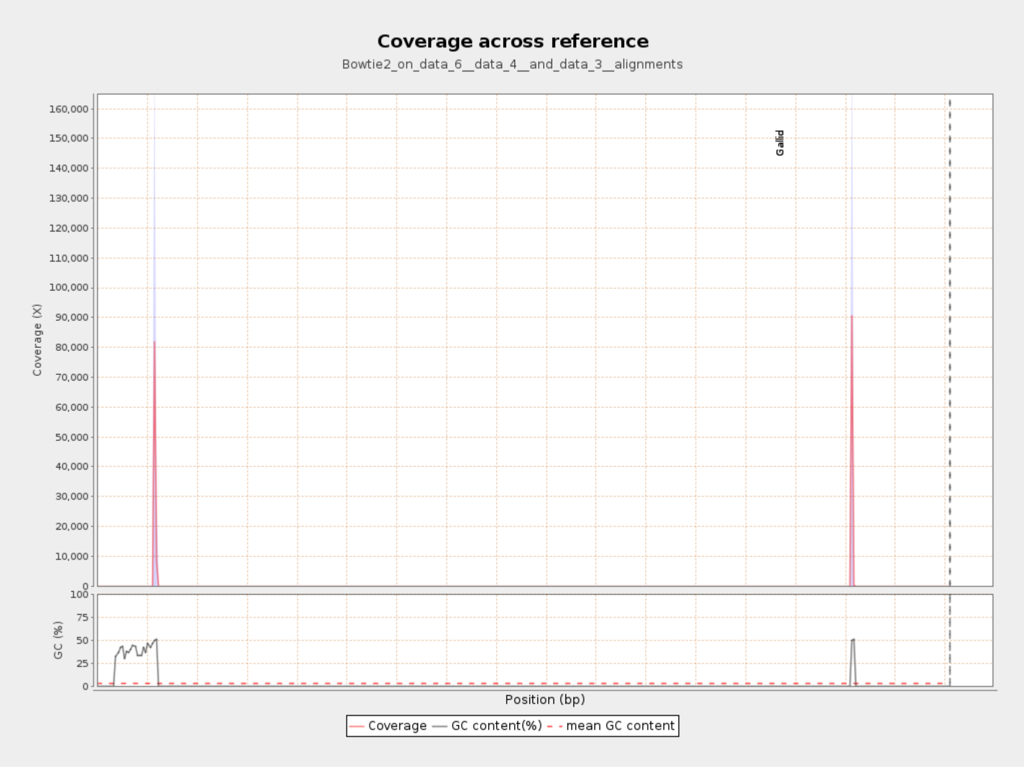

Coverage Histogram

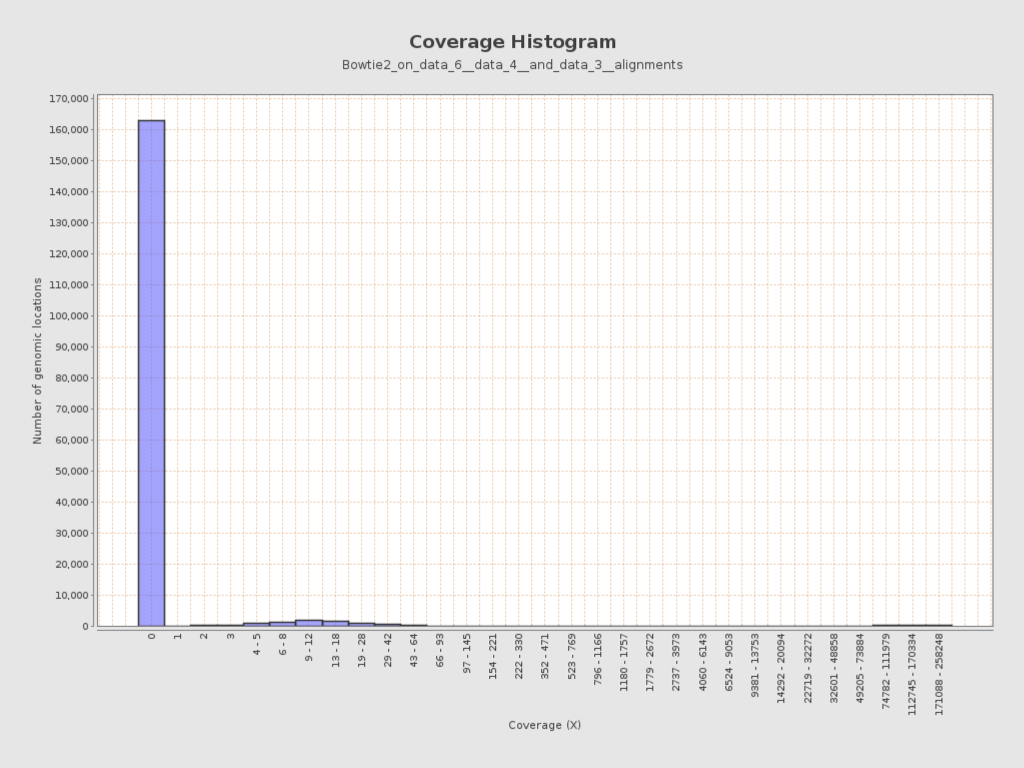

Coverage Histogram (0-50X)

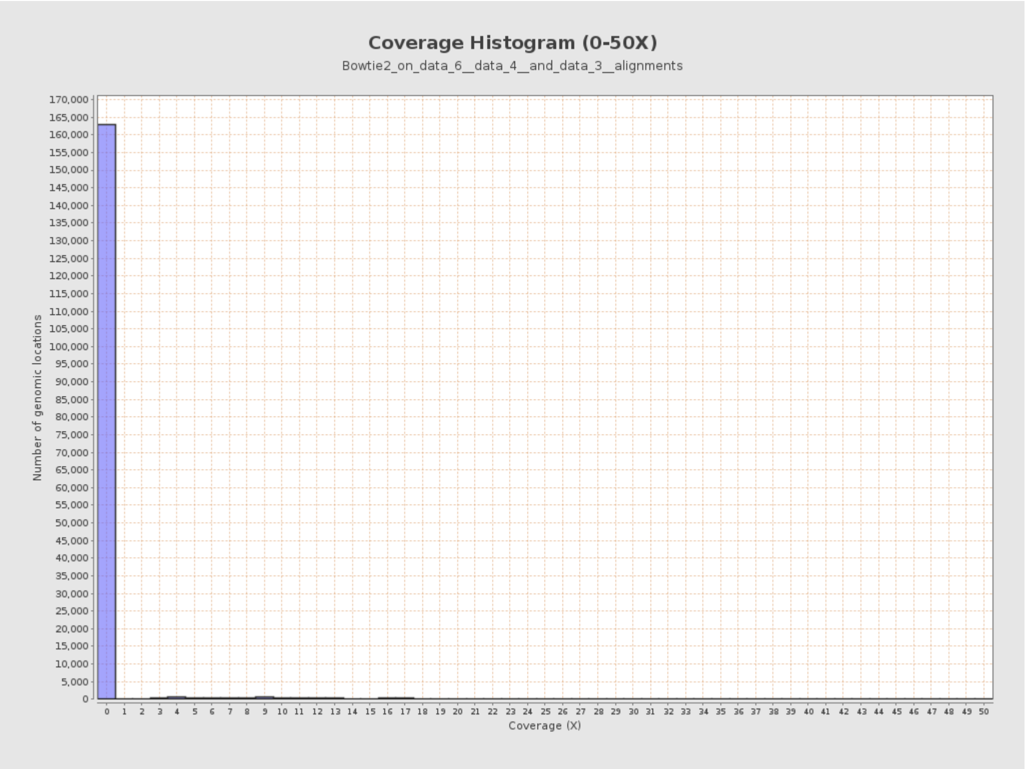

Genome Fraction Coverage

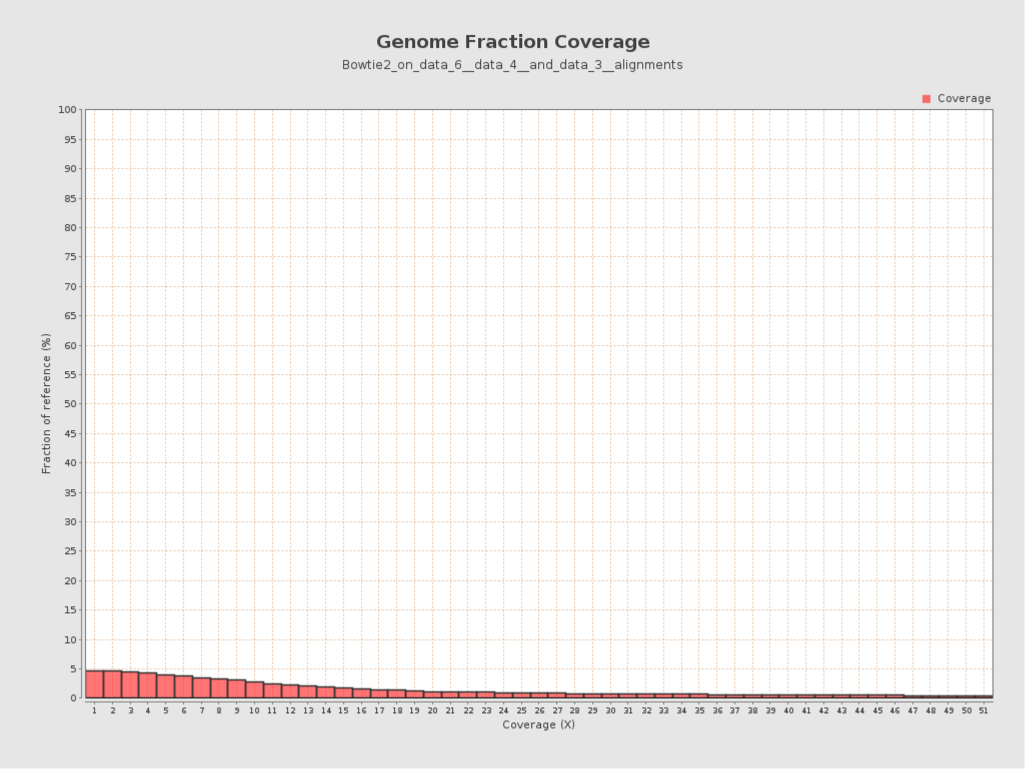

Duplication Rate Histogram

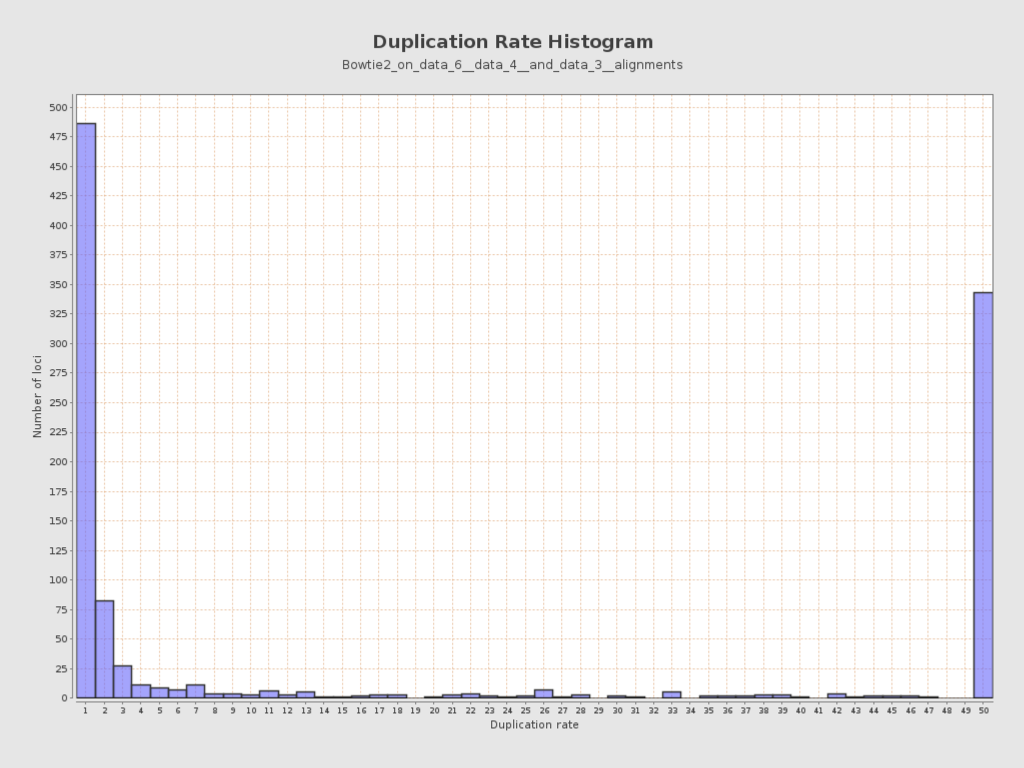

Mapped Reads Nucleotide Content

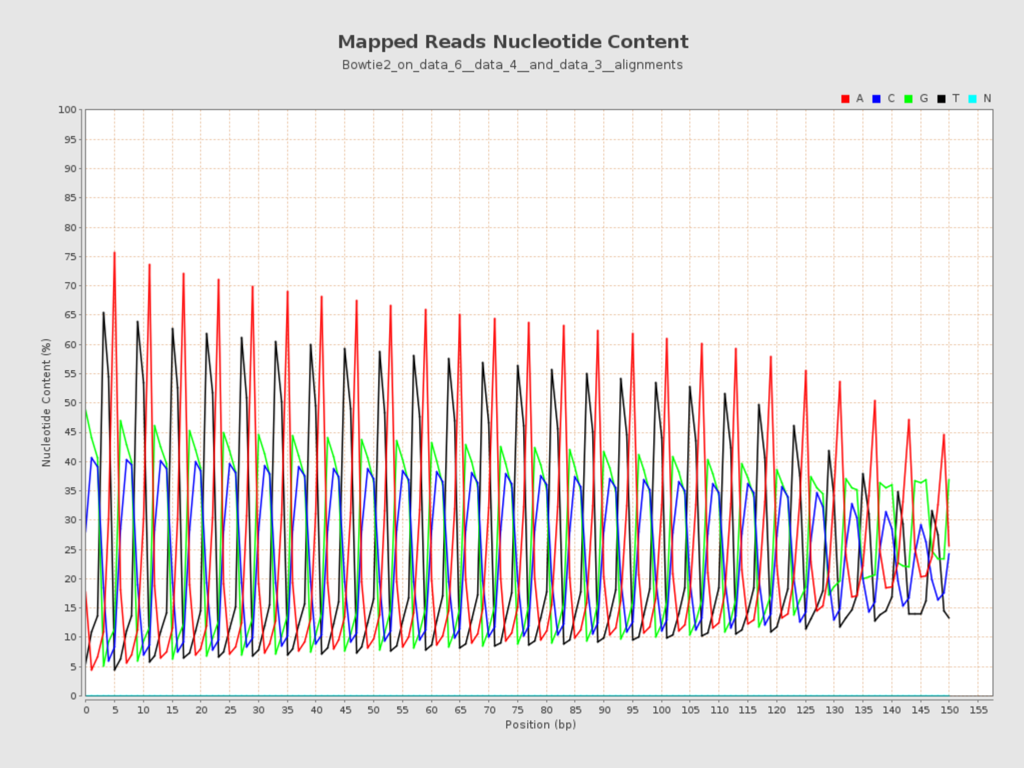

Mapped Reads GC-content Distribution

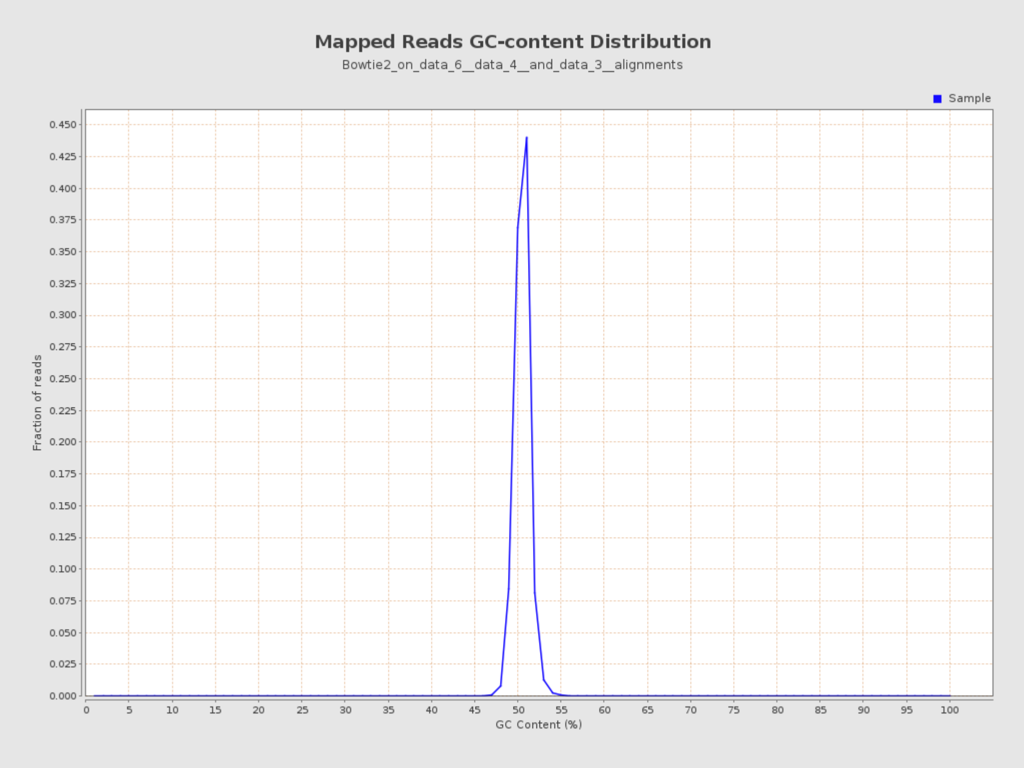

Homopolymer Indels

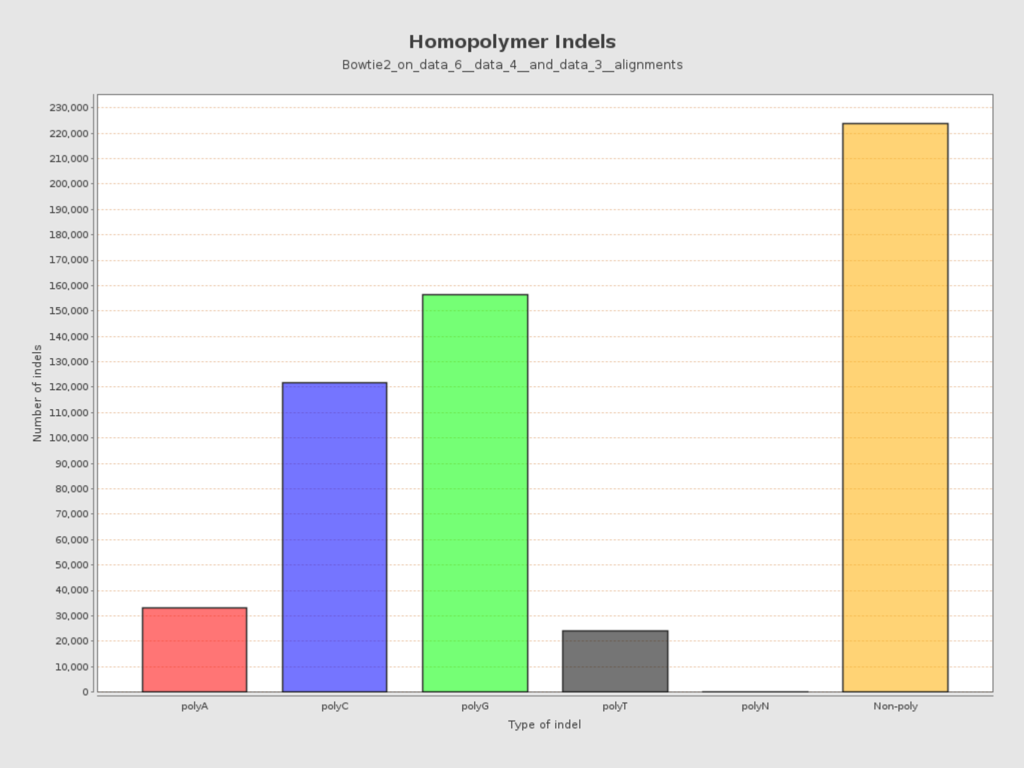

Mapping Quality Across Reference

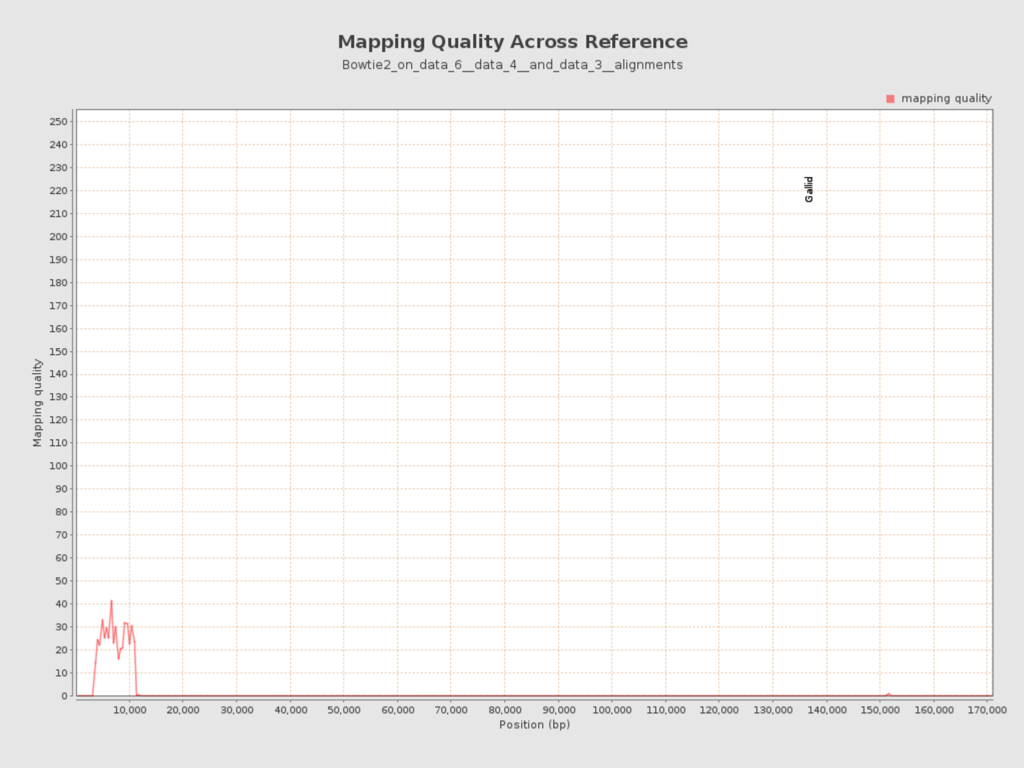

Mapping Quality Histogram

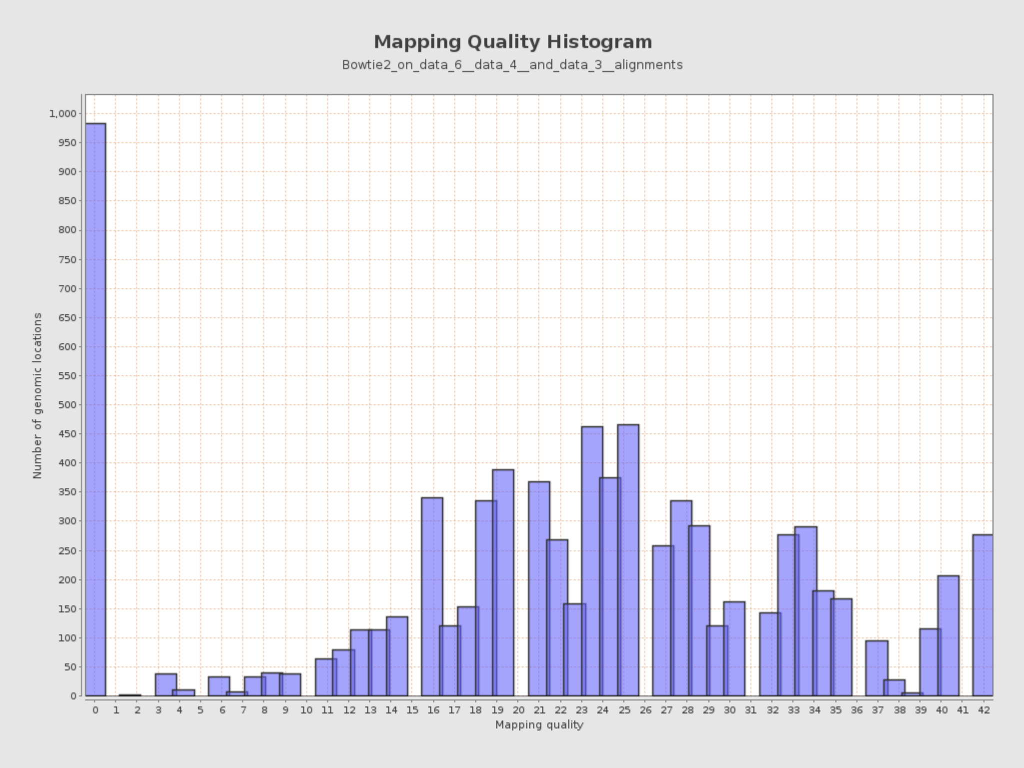

Insert Size Across Reference

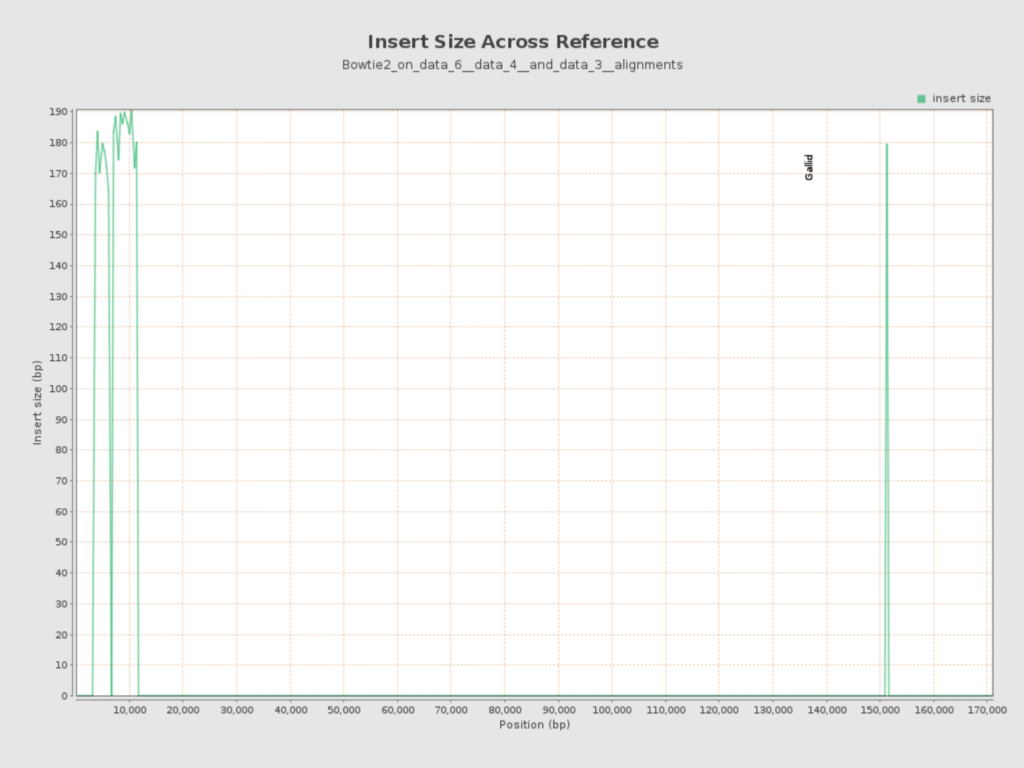

Insert Size Histogram

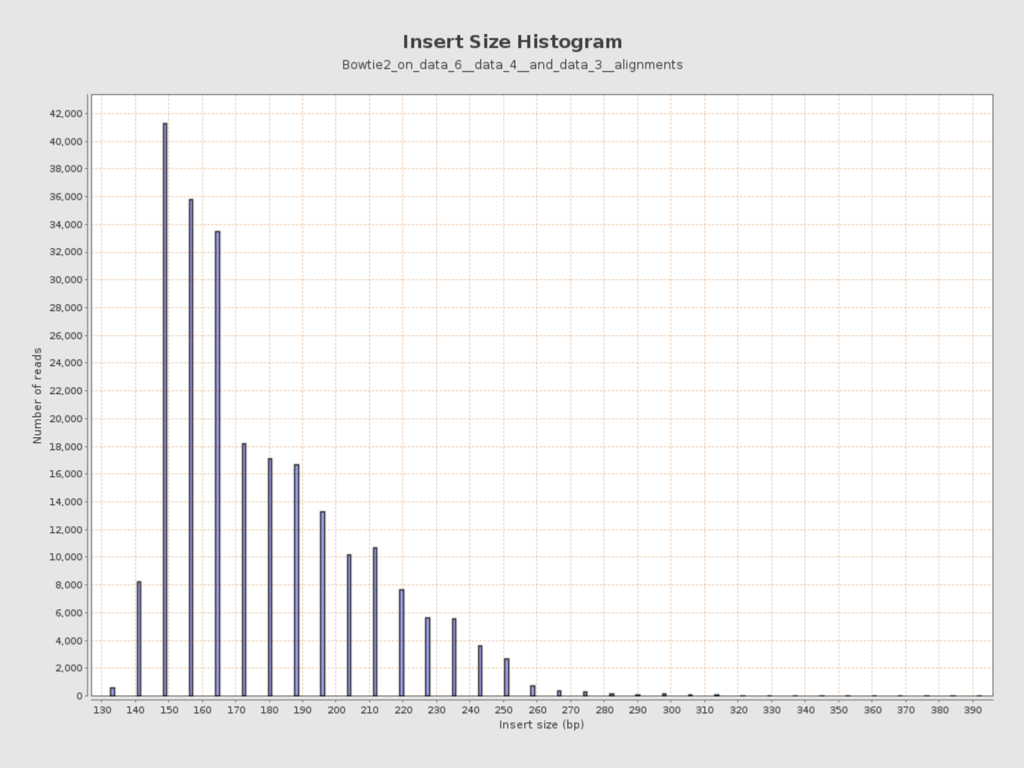

2021/12/09 05:25:51

Generated by QualiMap v.2.2.2-de

Qualimap Report: BAM QC

Input data and parameters

QualiMap command line

qualimap bamqc -bam Bowtie2_on_data_5__data_4__and_data_3__alignments -c -nw 400 -hm 3 -sd

Alignment

Command line:	"/mnt/pulsar/deps/_conda/envs/mulled-v1-2c9d0d73d81f016cd458c97e185f638410841f3ecf7ae79158d90e0d58249513/bin/bowtie2-align-s --wrapper basic-0 -p 8 -x genome --passthrough -1 input_f.fastq.gz -2 input_r.fastq.gz"
Draw chromosome limits:	yes
Analyze overlapping paired-end reads:	yes
Program:	bowtie2 (2.4.2)
Analysis date:	Thu Dec 09 05:27:30 UTC 2021
Size of a homopolymer:	3
Skip duplicate alignments:	yes (only flagged)
Number of windows:	400
BAM file:	Bowtie2_on_data_5__data_4__and_data_3__alignments

Summary

Warnings

No flagged duplicates are detected	Make sure duplicate alignments are flagged in the BAM file or apply a different skip duplicates mode.

Globals

Reference size	161,982
Number of reads	224,352,814
Mapped reads	527,752 / 0.24%
Unmapped reads	223,825,062 / 99.76%
Mapped paired reads	527,752 / 0.24%
Mapped reads, first in pair	265,019 / 0.12%
Mapped reads, second in pair	262,733 / 0.12%

CONTENTS

- Input data & parameters
- Summary
- Coverage across reference
- Coverage Histogram
- Coverage Histogram (0-50X)
- Genome Fraction Coverage
- Duplication Rate Histogram
- Mapped Reads Nucleotide Content
- Mapped Reads GC-content Distributio
- Homopolymer Indels
- Mapping Quality Across Reference
- Mapping Quality Histogram
- Insert Size Across Reference
- Insert Size Histogram

Mapped reads, both in pair	480,136 / 0.21%
Mapped reads, singletons	47,616 / 0.02%
Secondary alignments	0
Read min/max/mean length	151 / 151 / 151
Overlapping read pairs	185,248 / 0.17%
Duplicated reads (flagged)	0 / 0%
Duplicated reads (estimated)	526,911 / 0.23%
Duplication rate	95.84%
Clipped reads	0 / 0%

ACGT Content

Number/percentage of A's	20,009,387 / 25.59%
Number/percentage of C's	18,873,283 / 24.13%
Number/percentage of T's	19,058,225 / 24.37%
Number/percentage of G's	20,257,345 / 25.9%
Number/percentage of N's	658 / 0%
GC Percentage	50.04%

Coverage

Mean	483.4213
Standard Deviation	6,659.6046
Mean (paired-end reads overlap ignored)	373.63

Mapping Quality

Mean Mapping Quality	0.01

Insert size

Mean	349.87
Standard Deviation	4,209.94
P25/Median/P75	170 / 213 / 280

Mismatches and indels

General error rate	6.92%
Mismatches	3,924,331
Insertions	496,191
Mapped reads with at least one insertion	53.57%
Deletions	92,423
Mapped reads with at least one deletion	14.83%
Homopolymer indels	61.06%

Chromosome stats

Name	Length	Mapped bases	Mean coverage	Standard deviation
Human	161982	78305551	483.4213	6,659.6046

Coverage across reference

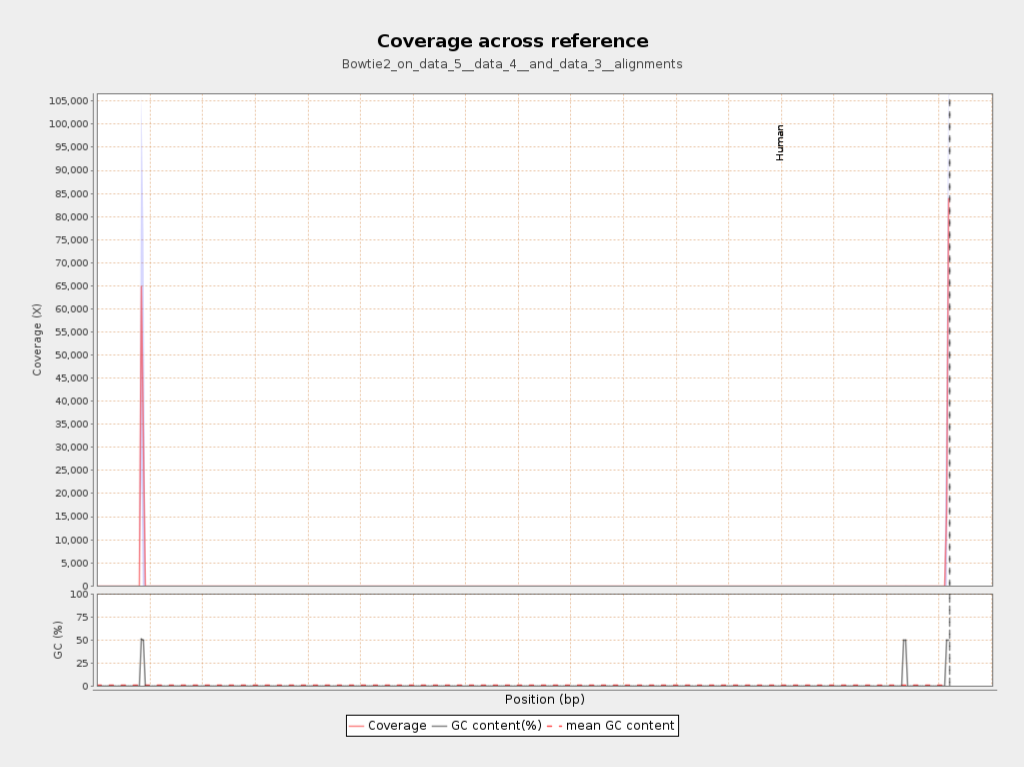

Coverage Histogram

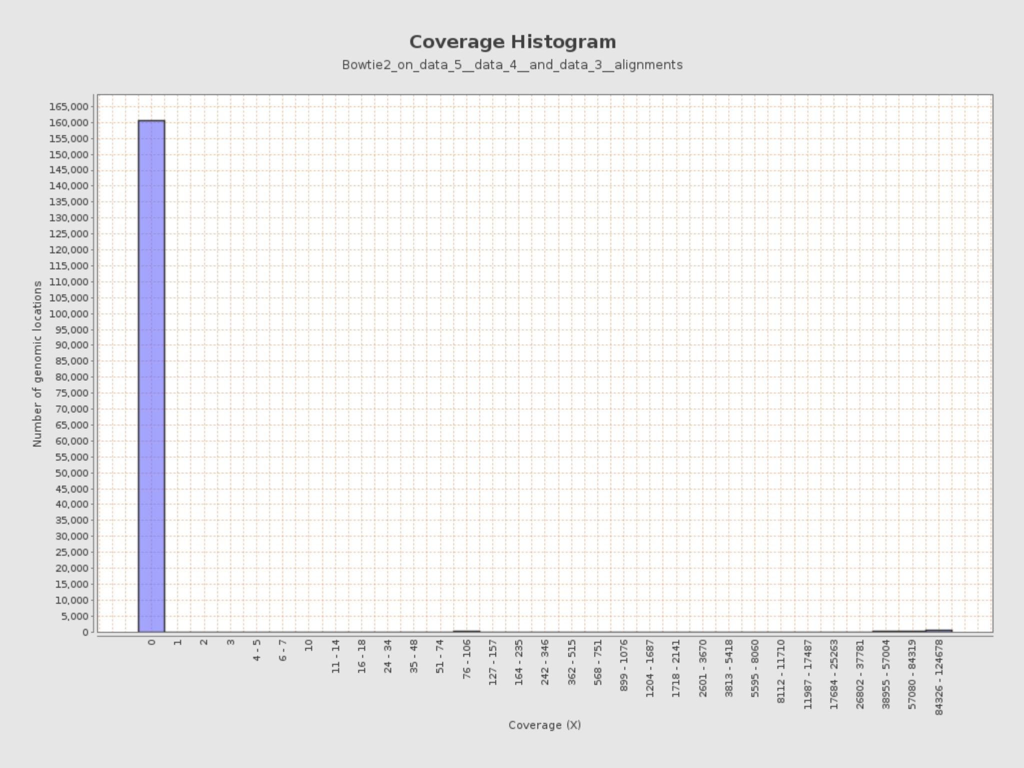

Coverage Histogram (0-50X)

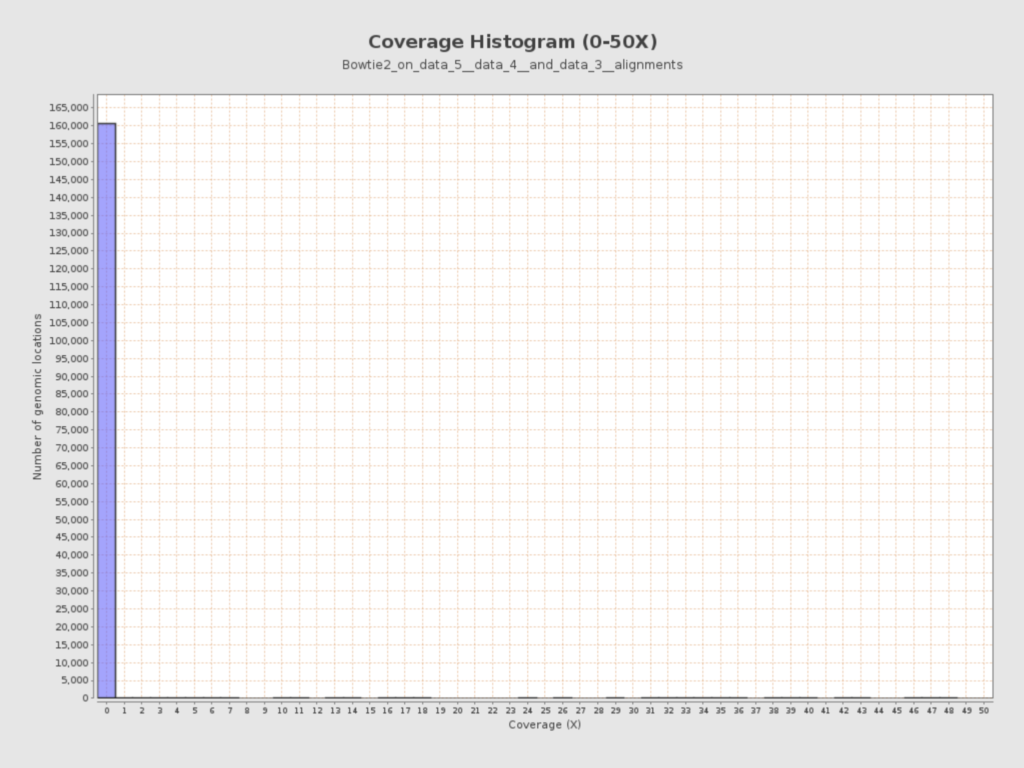

Genome Fraction Coverage

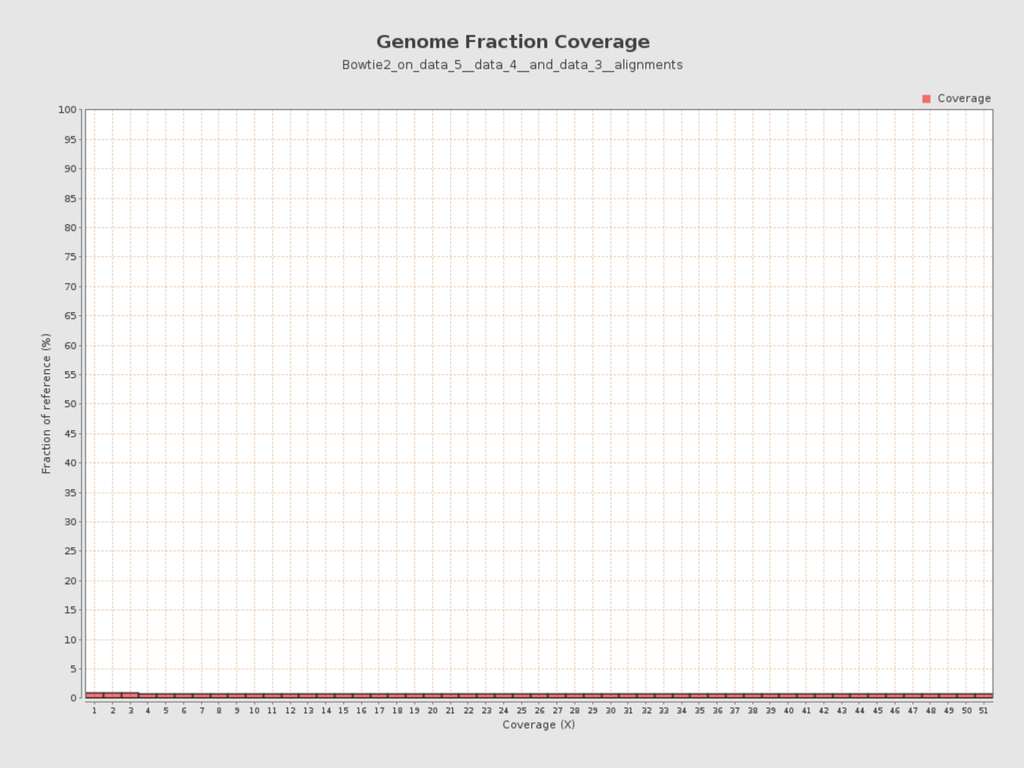

Duplication Rate Histogram

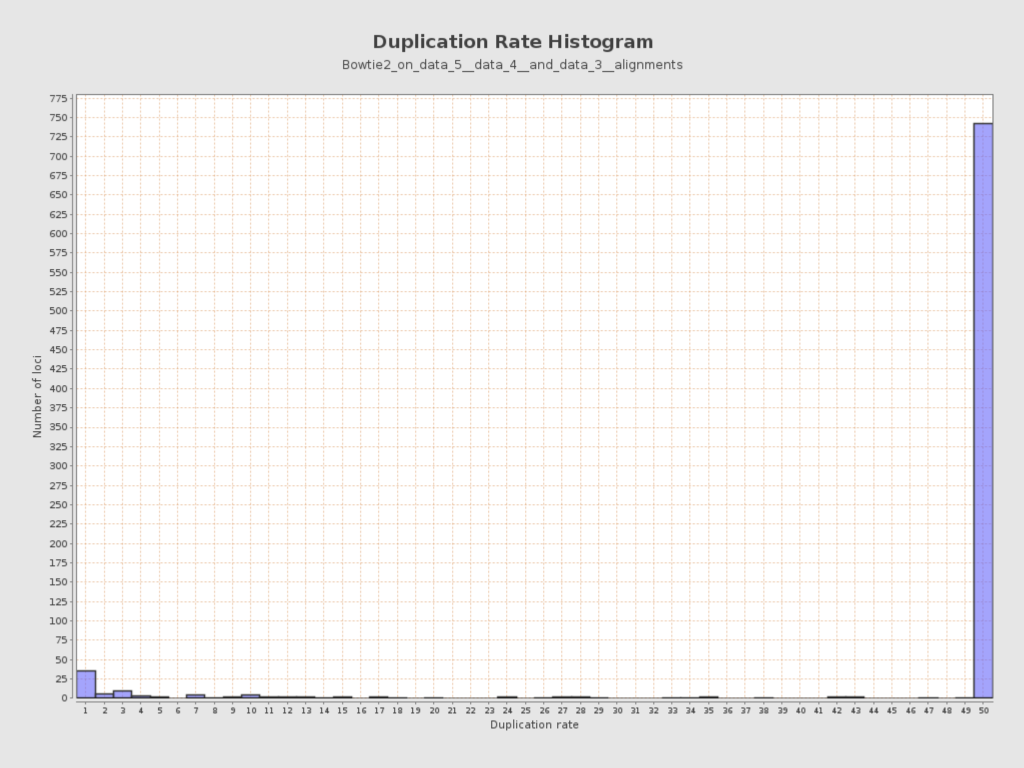

Mapped Reads Nucleotide Content

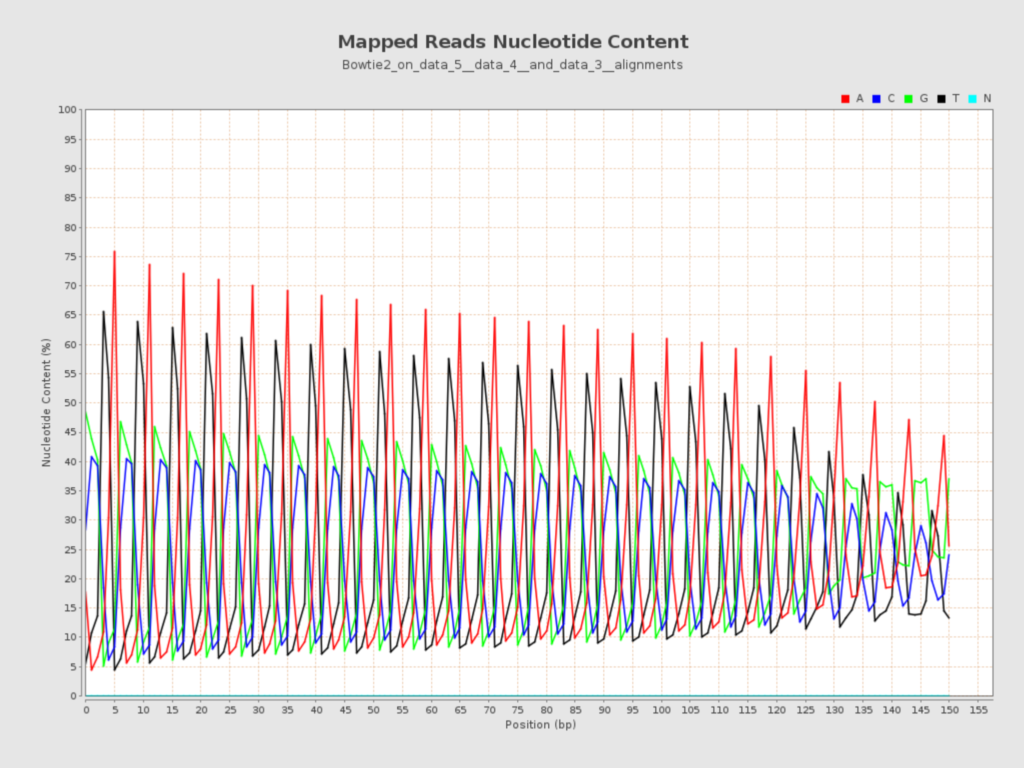

Mapped Reads GC-content Distribution

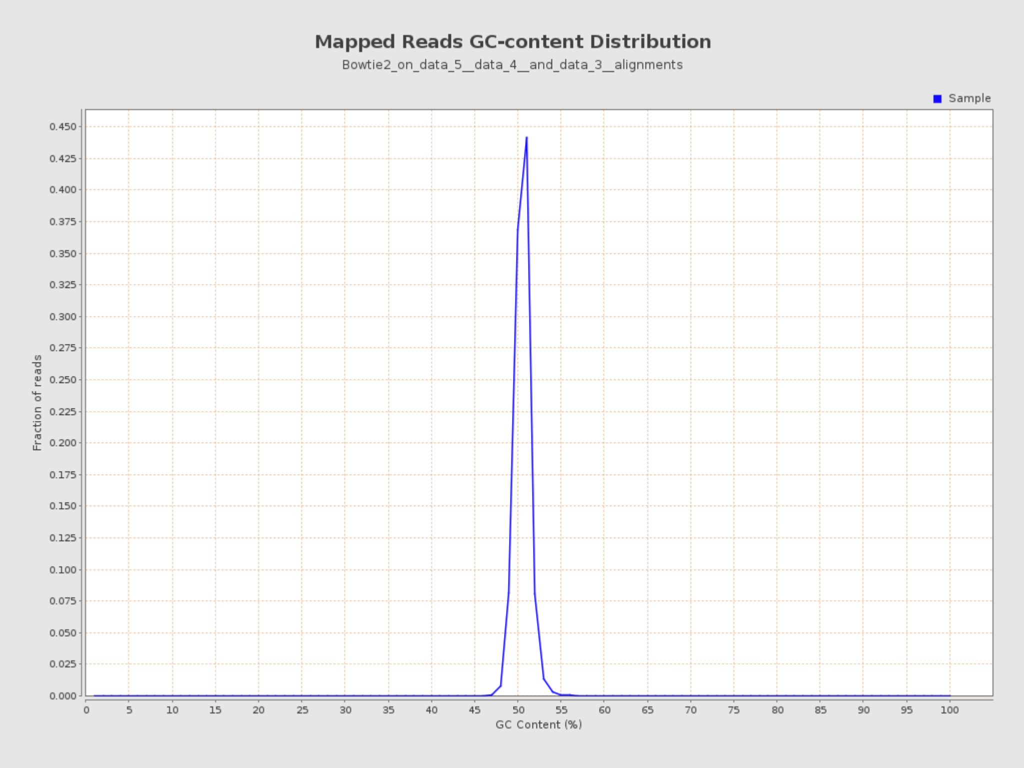

Homopolymer Indels

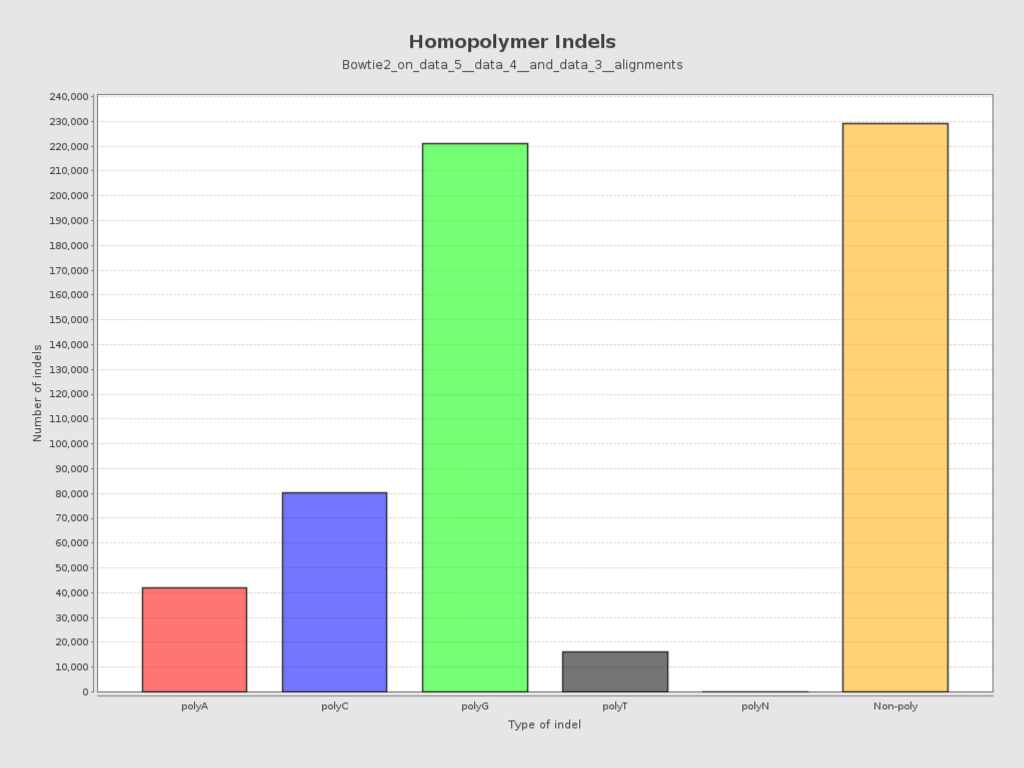

Mapping Quality Across Reference

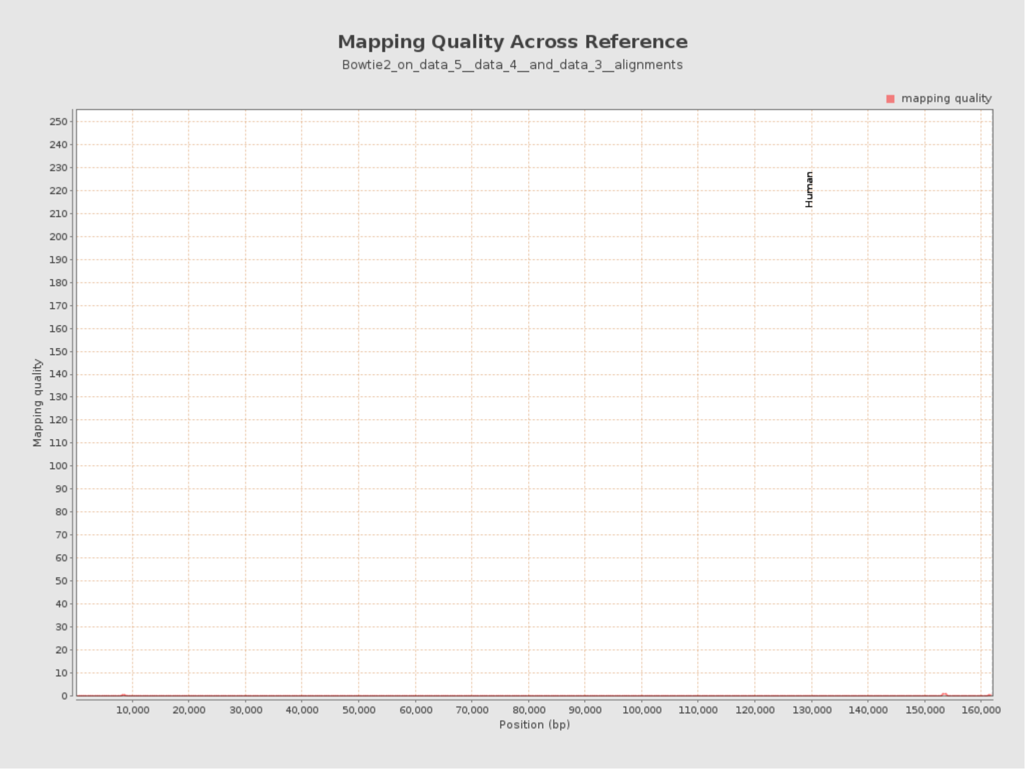

Mapping Quality Histogram

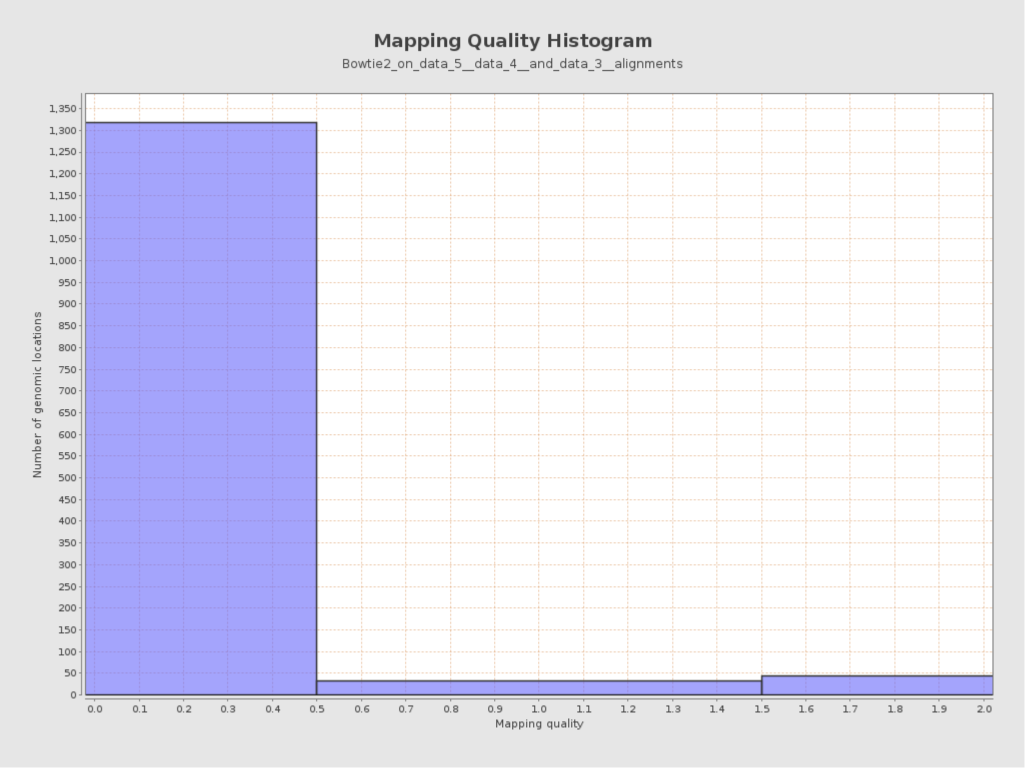

Insert Size Across Reference

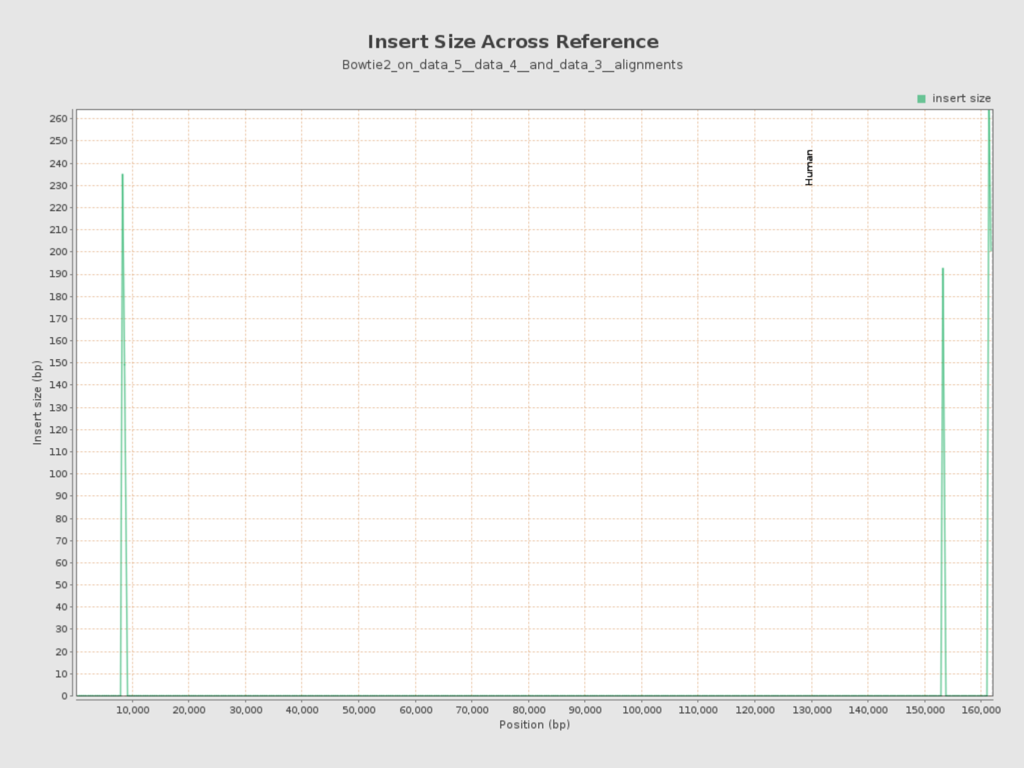

Insert Size Histogram

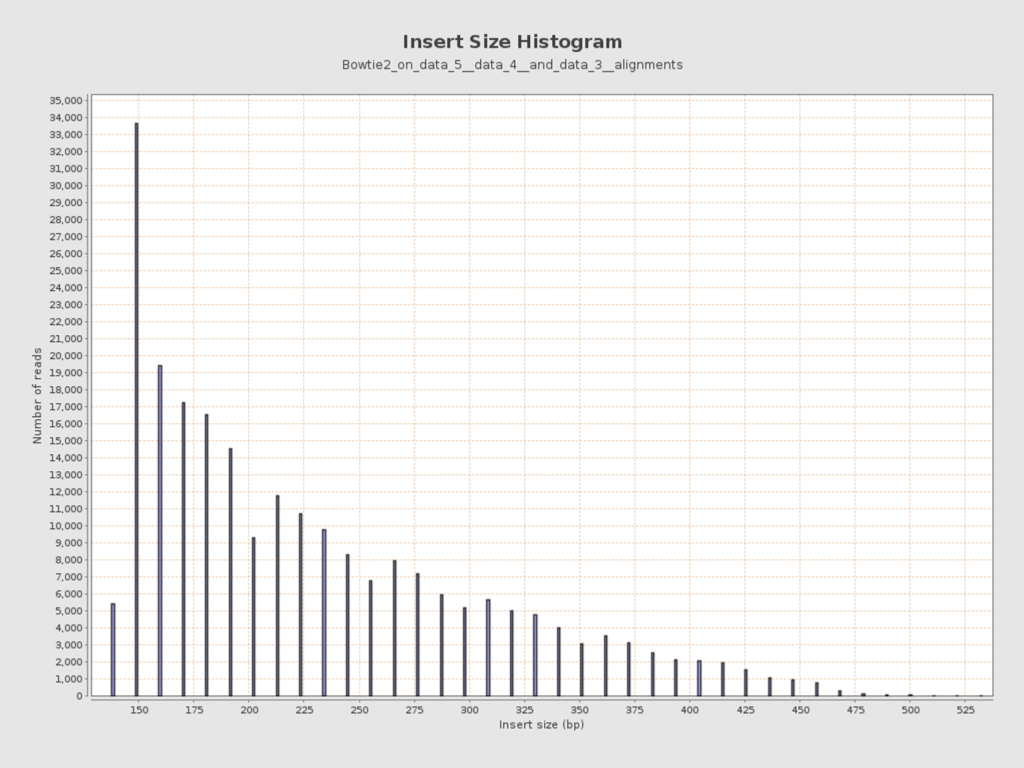

2021/12/09 05:27:31

Generated by QualiMap v.2.2.2-de
