# Supplementary material for: Investigation of a herpesvirus outbreak in mixed breeds of adult domestic ducks using next generation sequencing
Source: PLoS One. 2023 Jan 27;18(1):e0280923. doi: 10.1371/journal.pone.0280923 (PMC9882916; doi:10.1371/journal.pone.0280923)
Supplement: S3 File — (PDF) [file pone.0280923.s003.pdf]

Human herpesvirus 6 vs Gallid herpesvirus Sequence alignment

CLUSTAL 2.1 multiple sequence alignment

See page 12 for region of homology between HHV-6 and GaHV-2

Length	189217
Ave Identity (%)	45.13
Ave Similarity (%)	45.13
Ave Gaps (%)	24.39
Ave Score	28492.38

```
Human           ------------------------------------------------------------
Gallid          CCCGCCTATAACACGTGTTTGGTATGGGCGTGTCGCTATAGTGCATAAGAAGTTGACTAC 60


Human           ------------------------------------------------------------
Gallid          ATTGATCAATGACATTATATAGCTTCTTTGGTCAGATAGACGGCGTGTGTGATTGCGATG 120


Human           ------------------------------------------------------------
Gallid          TATCTACTACAATTATTATTTTGGATCCGCCTCTTTCGAGGCATCTGGTCTATAGTTTAT 180


Human           ------------------------------------------------------------
Gallid          ACTGGAACATCTGTTACGTTATCAACGGACCAATCTGCTCTTGTTGCGTTCTGCGGATTA 240


Human           ------------------------------------------------------------
Gallid          GATAAAATGGTGAATGTACGCGGCCAACTTTTATTCCTGGGCGACCAGACTCGGACCAGT 300


Human           ------------------------------------------------------------
Gallid          TCTTATACAGGAACGACGGAAATCTTGAAATGGGATGAAGAATATAAATGCTATTCCGTT 360


Human           ------------------------------------------------------------
Gallid          CTACATGCGACATCATATATGGATTGTCCTGCTATAGACGCCACGGTATTCAGAGGCTGT 420


Human           ------------------------------------------------------------
Gallid          AGAGACGCTGTGGTATATGCTCAACCTCATGATAGAGTACAACCTTTTCCCGAAAAGGGA 480


Human           ------------------------------------------------------------
Gallid          ACATTGTTGAGAATTGTCGAACCCAGAGTATCAGATACAGGCAGCTATTACATACGTGTA 540


Human           ------------------------------------------------------------
Gallid          GCTCTCGCTGGAAGAAATATGAGCGATATATTTAGAATGGCTGTTATTATAAGGAGTAGC 600


Human           ------------------------------------------------------------
Gallid          AAATCTTGGGCCTGTAATCACTCTGCTAGTTCATTTCAGGCCCATAAATGTATTCGCTAT 660


Human           ------------------------------------------------------------
Gallid          GTCGACCGTATGGCCTTTGAAAATTATCTGATTGGACATGTAGGCAATTTGCTGGACAGT 720
```

```
Human           ------------------------------------------------------------
Gallid          GACTCGGAATTGCATGCAATTTATAATATTACTCCCCAATCCATTTCCACAGATATTAAT 780

Human           ------------------------------------------------------------
Gallid          ATTATAACGACTCCATTTTACGATAATTCGGGAACAATTTATTCACCTACGGTTTTTAAT 840

Human           ------------------------------------------------------------
Gallid          TTGTTTAATAACAATTCCCATGTCGATGCAATGAATTCGACTGGTATGTGGAATACCGTT 900

Human           ------------------------------------------------------------
Gallid          TTAAAATATACCCTTCCAAGGCTTATTTACTTTTCTACGATGATTGTACTATGTATAATA 960

Human           ------------------------------------------------------------
Gallid          GCATTGGCAATTTATTTGGTCTGTGAAAGGTGCCGCTCTCCCCATCGTAGGATATACATC 1020

Human           ------------------------------------------------------------
Gallid          GGTGAACCAAGATCTGATGAGGCCCCACTCATCACTTCTGCAGTTAACGAATCATTTCAA 1080

Human           ------------------------------------------------------------
Gallid          TATGATTATAATGTAAAGGAAACTCCTTCAGATGTTATTGAAAAGGAGTTGATGGAAAAA 1140

Human           ------------------------------------------------------------
Gallid          CTGAAGAAGAAAGTCGAATTGTTGGAAAGAGAAGAATGTGTATAGGTTTGAGAAACTATT 1200

Human           ------------------------------------------------------------
Gallid          ATAGGTAGGTGGTACCTGTTAGCTTAGTATAAGGGGAGGAGCCGTTTCTTGTTTTAAAGA 1260

Human           ------------------------------------------------------------
Gallid          CACGAACACAAGGCAGTAAGTTTTATATGTGAATTTTGTGCATGTCTGCGAGTCAGCGTC 1320

Human           ------------------------------------------------------------
Gallid          ATAATGTGTGTTTTCCAAATCCTGATAATAGTGACGACGATCAAAGTAGCTGGAACGGCC 1380

Human           ------------------------------------------------------------
Gallid          AACATAAATCATATAGACGTTCCTGCAGGACATTCTGCTACAACGACGATCCCGCGATAT 1440

Human           ------------------------------------------------------------
Gallid          CCACCAGTTGTCGATGGGACCCTTTACACCGAGACGTGGACATGGATTCCCAATCACTGC 1500

Human           ------------------------------------------------------------
Gallid          AACGAAACGGCAACAGGCTATGTATGTCTGGAAAGTGCTCACTGTTTTACCGATTTGATA 1560

Human           ------------------------------------------------------------
Gallid          TTAGGAGTATCCTGCATGAGGTATGCGGATGAAATCGTCTTACGAACTGATAAATTTATT 1620

Human           ------------------------------------------------------------
Gallid          GTCGATGCGGGATCCATTAAACAAATAGAATCGCTAAGTCTGAATGGAGTTCCGAATATA 1680

Human           ------------------------------------------------------------
Gallid          TTCCTATCTACGAAAGCAAGTAACAAGTTGGAGATACTAAATGCTAGCCTACAAAATGCG 1740
```

```
Human           ------------------------------------------------------------
Gallid          GGTATCTACATTCGGTATTCTAGAAATGGGACGAGGACTGCAAAGCTGGATGTTGTTGTG 1800

Human           ------------------------------------------------------------
Gallid          GTTGGCGTTTTGGGTCAAGCAAGGGATCGCCTACCCCAAATGTCCAGTCCTATGATCTCA 1860

Human           ------------------------------------------------------------
Gallid          TCCCACGCCGATATCAAGTTGTCATTAAAAAACTTTAAAGCATTAGTATATCACGTGGGA 1920

Human           ------------------------------------------------------------
Gallid          GATACTATCAATGTCTCGACGGCGGTTATACTAGGACCTTCTCCGGAGATATTCACATTG 1980

Human           ------------------------------------------------------------
Gallid          GAATTTAGGGTGTTGTTCCTCCGTTATAATCCAACGTGCAAGTTCGTCACGATTTATGAA 2040

Human           ------------------------------------------------------------
Gallid          CCTTGTATATTTCACCCCAAAGAACCAGAGTGTATTACTACTGCAGAACAATCGGTATGT 2100

Human           ------------------------------------------------------------
Gallid          CATTTCGCATCCAACATTGACATTCTGCAGATAGCCGCCGCACGTTCTGAAAATTGTAGC 2160

Human           ------------------------------------------------------------
Gallid          ACAGGGTATCGTAGATGTATTTATGACACGGCTATCGATGAATCTGTGCAGGCCAGATTA 2220

Human           ------------------------------------------------------------
Gallid          ACATTCATAGAACCAGGAATTCCTTCCTTTAAAATGAAAGATGTCCAGGTAGACGATGCT 2280

Human           ------------------------------------------------AAAAAAAGGGGG 12
Gallid          GGATTGTATGTGGTTGTGGCTTTATACAATGGACGTCCAAGTGCATGGACTTACATTTAT 2340
                                                *   * *

Human           GTATTAACNNNNNNNNNNNNNNNNNNNNNNNNNNNNNNNNNNNNNNNNNNNNNNNNNNNN 72
Gallid          TTGTCAACGGTGGAAACATATCTTAATGTATATGAAAACTACCACAAGCCGGGATTTGGG 2400
                 * * ***

Human           NNNNNNNNNNNNNNNNNNNNNNNNNNNNNNNNNNNNNNNNNNAACCCTAACCCTAACCCT 132
Gallid          TATAAATCATTTCTACAGAACAGTAGTATCGTCGACGAAAATGAGGCTAGCGATTGGTCC 2460
                                                           *  *** *  *    *

Human           AACCCTAACCCTAACCCTAANNNNNNNNNNNNNCNNNNNNNNNNNNNNNTAACCCNNNNNNN 192
Gallid          AGCTCGTCCATTAAACGGAGAAATAATGGTACTATCATTTATGATATTTTACTCACATCG 2520
                * * *   *  *** *  *                            *   *

Human           NNNNNNNNNNNNNNNNNNNNNNNNNNNNNNNNNNNNNNNNNNNNNNNNNNNNNNNNNNNN 252
Gallid          CTATCAATTGGGGCGATTATTATCGTCATAGTAGGGGGTGTTTGTATTGCCATATTAATT 2580

Human           NNNNNNNNNNNNNNNNNNNNNNNNNNNNNNNNNNNNNNNNNNNNNNNNNNNNNNNNNNNN 312
Gallid          AGGCGTAGGAGACGACGTCGCACGAGGGGGTTATTCGATGAATATCCCAAATATATGACG 2640

Human           NNNNNNNNNNNNNNNNNNNNNNNNNNNNNATAGCCCTAACACTAATCCTCGCATCTGGCCC 372
Gallid          CTACCAGGAAACGATCTGGGGGGCATGAATGTACCGTATGATAATACATGCTCTGGTAAC 2700
                                            **   **  *   **** *  **    *   *

Human           TAACACTACCCCTCTTTCAACCACTCA-CCATCACCCCACCCCCCCCCNCACAC-ACACC 430
Gallid          CAAGTTGAATATTATCAAGAAAAGTCGGCTAAAATGAAAAGAATGGGTTCGGGTTATACC 2760
                 **    *    * *    *  * **  * *  *    *          *     * ***
```

```
Human           ACACACACCGCCACCGCTACCACCACCTCTGAACTTCACCTTTTCCCTCCATCTCGCCCC 490
Gallid          GCTTGGCTAAAAAATGATATGCCGAAAATTAGGAAACGCTTAGATTTATACCACTGATAT 2820
                 *          *  * **   * *    *      * * *              *

Human           ACTTCTCTCTACACTTCTCCGCCCCTC-TATTCTTACTCCTGTTTTCTAGGATGCCGCTG 549
Gallid          GTACATATTTAACTTAATGGGATATAGTATATGGACGTCTATATGACGAGAGTAAATAA 2880
                     * * ** ****    *    *  ***    **  ** * *     **

Human           ACGGCGCGTGCCGGCCACGCC---CTGCATCGTCTTCCGCTCTCCCACTA-TTGGTGGCT 605
Gallid          ACTGACACTGCAAATGAAGCTGATCTATATTGTGCTTTATATTGGGACAAACCACTCGCA 2940
                ** *    ***     * **    **  ** **  *      *   ** *     * **

Human           ACTGTTGGGTCGACAC-TCCCTTCGTCATGTCCATTCCTACCTTCGCCTGCACAAAGGTC 664
Gallid          CAAGCTCATTCAACACATCCACTCTTGCTATTAAATTCCCCATTATATAACAATACTGAC 3000
                   * *   ** **** ***  ** *  * *  * * *  * **      **  *  * *

Human           TACGCCTTCCTTTACCTTGGCCCGAGCAAGAATGCCTG--CATTTACATCCTA---AGCC 719
Gallid          ATAACACTCATATTAAGGGGAGAAAATAAATATGCATGGCCGATCATATTTTATTGAGAT 3060
                    *  ** * *     **    *  **  **** **  *  * * **  **   **

Human           TTACAAGTTTCTCCTGCG--TTACCCTTGTCTAACAAGACAACCGCATCTTCTTCAGGGC 777
Gallid          CCGAAAATATATCATGCAAATAAGCATGTTCTAGCACCACTGCAACATGTGGTTTATCGA 3120
                    ** * * ** ***   * * * *  **** **  **  *  *** *  ** *  *

Human           TGGCCCG----------CGGATTCTT--CTCTATGTGAGTGAC-ATTTACACTTCCA--C 822
Gallid          TTTCCGGAAAGAATAGTTGAACCATTGCCTCCGAGCAGTTGGCGATCTACTTCCGCAGCC 3180
                *  ** *           * *   **  ***   *    ** * ** ***     **  *

Human           TTGTTCACATGATTTATTGTGTTTTGTCT-GCTACACCAAGCACATTTCGGTTTCTCTTT 881
Gallid          TTCGAAATATATCTCATAGAACTCTTCCTTAAAACTGCACGGAGATTTTCCATCCTCCTT 3240
                **    * **   * ** *   * *  **    **  ** * * ****    * *** **

Human           TATACATTTGTCTCTA---------TTCTCTCGGTC--TCAGGGTT---CGACCCTAAAC 927
Gallid          AGAAGCCTTGCAGACAAAAGCCAATTTCTCTTCAACGGCCAGGGACGGGCAATCGCGATA 3300
                   *   ***     *         ******    *   *****     * * *   *

Human           CCTACCATCCTTCGGCCGACAGC-----AAGTTGCTACCGCTG-GGCCTGATCACGCTGT 981
Gallid          CATTTCACGCATGGATCCATGTCCCCTGGAGTTGGAAGAGTTATAGTTTATTTATCCAAG 3360
                * *  **  * * *  * *   *      *****  *  * *   *  *  * *  *

Human           CCGCCTTTTCCATGCGCGTTTCTGAG-CCGACACACT-GCAGCGGCTTCCACGCGGCACA 1039
Gallid          TTAATTTTCGTCAGCACATAGCTAAAATTGAATTACTTACATCGAGCTTAATTCGTCGAA 3420
                     ***     ** * *  ** *    **   ***  ** **   *  *  ** *  *

Human           TCCGTCTCTCAGTTGGTTAACGGGATCGTCCCC--TTGGCTCGTGCTCCTACAAGCGCCA 1097
Gallid          CATACAACTATGATGACAAGCAGAAGTAACCAGGACAGCTTCATGTTAAAATAAACTGTA 3480
                       **  * **   * * * *    **      *  ** ** *   * ** *   *

Human           GGAGGGTCTCTGTTCT----GCCACGACGTGTTCCAAGGCCGAC----TCTATCTCCTGT 1149
Gallid          AATCATTCAATGATAATGGGAGAAGAATGTGAGCAAGGATCCATGGTGTCTGCTTTTTAT 3540
                      **  ** *         *  * ***  * * *  * *     ***   *  * *

Human           CGCACT--CCGTGTCGCT-CTTTCTAAAGACGGGCCTTCGCCAGTG---TGAGGCCATCT 1203
Gallid          AGTATCTACCGCAATGCTACATGGTAAATTCTCCCCTCCCATAATGATTTATAGTGATGC 3600
                 * *    ***    *** * *  ****  *   *** *   * **   *   *  **

Human           ATCGCGCACCGCTG---TGGCGCGTACGGCCCCTGCCGAGCC-TATGGACGTGTCGAGAT 1259
Gallid          AATATGCAGCACAGGTTTTGTATGCATCCATTCTGATGAGCTATGCTGTTGTTTTGAAGC 3660
                *    *** * * *   * *   * *      ***  ****  *   *  ** * **

Human           CCCGACACGGCCTTCTTGCCGAAATTACTGGCGAGAACCGCCCGACGCGGCCTGGCCGCT 1319
Gallid          TTCTAAAAATATTTAACCAAGGGACTGCTCATTCTACTTGCTTCTGCCTGTTCAGTCACT 3720
                  * * *     **      *  * * **      *   **      * *    * * **

Human           TTC--TATGCCCTGTGGAGACTGCATCTGGGATCCCGCTCGGAGCTCTCTCACCCCGTGT 1377
Gallid          GAGAGTGTGATTTTGGTAAAAGGTAACAAGTCATAGAAATGGAAGTTATGCAAACC-TAG 3779
                     * **   *  * * *  * * *  *          ***  *    **  ** *
```

```
Human           TGGAGTGGGAGAGAACAGAGCTGGTCCTGACGGATCAGAGACGCAGGTGGCCGTGTACGC 1437
Gallid          TGAGATTTGAAAATGGAAAGTTTTTATGTTTGCTTAAATAAACAAATAACTGATTTCCC 3839
                **   *  ** *    * ** *  *  **   * * * * *  **  *  * *  * * *

Human           ACCTCCTGTCCGGCTCGGA-------GTTCCAGCGCGTTTC-CTCGAGTGACGC----CG 1485
Gallid          GTATTTATTTCAGCTGGATTGTTTCTGTTTTTGTATATTGAATTCGGTTGTTGTGCTGTG 3899
                   *    * * *** *         ***   *  **      ***  **  *      *

Human           GAGACACATGGAACGCA-------GCGACAGAGAAGGCGGCGGGGGGAAAAGAGGAGGCG 1538
Gallid          AAAACTTATTTCAAGCATTCTCTAGTGAGTGTGAATATAGAAGATAAATTACCAATTGCG 3959
                 * **  **   * ***       * **  * ***    *  *    *  *      ***

Human           GAGAGAGGCGGGCGACAGCAAGCCACTGACAGACTCGC---AAGTCCG-------CACCT 1588
Gallid          TTTTGTGGCTTCAGAGATGGAGCCTTTAGCATTTCTGCTGAAAATGTGTTTTGCTCCCTT 4019
                    * ***    ** *   ****  *  **     **   ** *  *       * * *

Human           GACGCGGGGCCTCCGCGACTCCGGTCGGTCCCTTCAGGGTGA----GGAGCCCAGCGCCG 1644
Gallid          AACCCAAAGTTTCCTT--TCGTTGTATATATTTTCAATTTTACACTGGAAGCAGTCACTC 4077
                 ** *   *  ***         **   *   ****   * *    ***  *   * *

Human           CGGAAGACTTTGCGAGGTGC-AGACCGCTGCTGGACGAACTGTGCGGGGAGGGCGGCTGG 1703
Gallid          AGGTAGCTTAAAATGGGTGAGAGAAGGATTCAAAGAGAGCTCTGTCACAAGAGCAGCAAC 4137
                 ** **  *      ****  ***  * * *     ** ** **     ** ** **

Human           CTT--------CCCTTTGCGTTTCTCACGGCATCTCCGCACGTCTGTCTGATCCTAACGG 1755
Gallid          CTTAGCACGTGTCACTGTAATTTATCTGATTCTTCTTGCAGTTTAGACACTGAATAATAA 4197
                ***         *  *    *** **      *    ***  *  * *      ***

Human           AGGGAGGCCCCGTCCTGGCGCTTGACCTGAACGACACCTCCCTGTGGCGCATCGCGGACG 1815
Gallid          ATTATTATTGGAATTAAAAACTACACAGAAATAAATAGCCCTCATAACAACTAATGGGTG 4257
                *                   **  **   **  *     **   *  *   *   **  *

Human           ACTTGG--AGCTGCTGCTGCGCCTGGGGAGCCTGCT-------CCTGCTCTCAGGGCTCC 1866
Gallid          GTTAAACAAGCAAAAGATGACCTTGAGGTGTTAGATATTTTAGTCTTATCTCTGGAACAT 4317
                  *     ***    * **  * ** ** *   * *        **  **** **

Human           GGCTTCCTCTCCGTC--------------CCCCGAGCGGGAGCGGCGAGGCGGCGAGAAA 1912
Gallid          GGCTAAATCTCTGCTTTCACGAATGCACTCCCTGTAAATAGGAAACTAAGCAGAAAGTTG 4377
                ****   **** *                *** *       *   * * ** *  **

Human           GCCGGGG----TACGAGAAGGAAGAGGGAAGAGGGAGAGCGGCG--ACGGCGAGCGCGAC 1966
Gallid          GCCAGCAATTCCACACAGTGCATCAGAGCTGAGGTCAGACAGGATCACAGCGGATCCCCT 4437
                *** *       **     * *  ** *  ****     * *    ** ***    *

Human           GGCCGCGACGTCGCCGCGCAGACCG--ACCCGTCCGAGGGGGGTGA----CGGAGAAGGG 2020
Gallid          CTTTGCACCCTTACCACCTGACCTGTCACTCGTCCCTCCTGGATGTACTCCGCAGGCTGG 4497
                    **  * *  ** *     * *  ** *****     ** **      ** **  **

Human           ACGTGTGACAACC--------GGGGACGTCCCTTTCTCCGCACATCCCGAATCTGAGGAA 2072
Gallid          CTGAGCCGTGATTTTTTTAATGAAAACTGCAGCTTCTAGTAGGATCTAAAATCTGAACTT 4557
                  * *     *          *   **  *   ****      ***   *******

Human           CAGACAGACG---GCCACCACGGGCGCCAGGAAAGCGGCCACGGCGACCAG-------CG 2122
Gallid          CTGATAAATGCTGGGTAATTGGAAAGAAAAGGAATTACTGGATGTGTACAGATGTGCTTG 4617
                * ** * * *   *  *    *   *  * * **         * *  ***        *

Human           CGGCGGGGAC-----------GGACGAGGACACCGCGATGACGGCGCGCGCCGCCACGCG 2171
Gallid          CACCAAGAGCCTGTTTCTTTTAGACTTGTAAAGTGAAATGATGTTGAAAGGCTCCAAAGT 4677
                *  *  *  *            ***  * * *  *  **** *  *   * * ***

Human           AA----TGACGAAACAGAGCCCCAGCAGCGCGGAGAGCACGAGGACGGGGAACAGACCGA 2227
Gallid          AAAACCTGGCCAATTTGCGTGATTGTTTGGAGGGAGACAGGAGGGTGGTAGGAATACAAG 4737
                **    ** * **   *   *     *    * **    ** ****  **     * **

Human           CTCCGGGCGCGAGGAGGACGCACAGGAGAGCGAGGTCGCAAGAAGAGACGAGAAGGGAAC 2287
Gallid          TTTT-----CATGTAAAATGTTGAAAGCAGGACGGTCCCACTGGGGACTCTGCAGGCCTC 4792
                 *       *  * *  * *   *    **   **** **    *      * ***   *
```

```
Human           GGA--GCAGGGCGGTAGCGGAAGGAGCTGCGGGAGGGC---AACGCAGACGTACGGCGGG 2342
Gallid          TGACTATAAAGCTGTCCAGGCTAGATATCCTGCAAGGCCTGAGTGCAGGGATTTATCTGC 4852
                 **    *  ** **   **   **  * * * * ***   *  ****   *    * *

Human           AGAGGCGAACATGGTGCCTGGTCGTCGATCCCCCTGTCTGTCCCCAGGCCCGATCC--CC 2400
Gallid          ACAAG-GGATTTGAT-TCAGATCACAAAAACATCAGCAGCTGTTTCAGCCAACTTAAACC 4910
                * * * * *  ** *  * * **    *  *  * *    *      ***   *    **

Human           GCGTGTGGGTCCCTCCT----CCTCATCTGTTATTCCCTTCCCCGCTGCCGTCGATAACG 2456
Gallid          AGGTACAGCTTTTTCCAGAGATTAAAATCATTACTAGTCCCCTGCATGTCAGTGATAGCC 4970
                  **   * *   ***         *    *** *     **    ** *   **** *

Human           CCCGTCGAAGACGAGCCGTCCGCCCGCCCTCGGTGCCCGCCAGGTCCCGCGGAGGAACCC 2516
Gallid          ATTTCTGCTGATAATAAGTGTTACTGGGATTAAATATCACCGTATCTAAGGAATTATTAG 5030
                      *  **  *   **    * *   *       * **   **    * *  *

Human           TCTAAGTGTTCTCCGTGCCCGCCCTGCCCGTCGCCCGAC---GCCCCGCAGTCCGCTGTC 2573
Gallid          GTGAAATGAAAATTATGCTTTTTTAAATCGCTATAAAAATTGGCTCCACAAGTC-CTGGC 5089
                   ** **       ***          **       *    ** ** **   * *** *

Human           CCGCGCCTCTCCGCTCTGT--CCGTCCCCTCGCCGTCCACCGCGCGTGTCCGTTTCTCCC 2631
Gallid          CTGCAGAAGGACTCTCAAAAACAATCTGCTAGGTGTATGCCAGTTTTCACAG--CCTTCC 5147
                * **       * ***     *  **  ** *  **   **     *  * *   ** **

Human           TCTCCTCCCTCTCCTCCTCCTCCTCCTCCTCTTCCTCCTCCTCCCCGTCCTACTCTCCCT 2691
Gallid          ATCCCACAGGGACAAGGAATTCAATAGTGCCCTACATTTTTAATGTGTTGCAGCTTTTTT 5207
                   ** *     *       **        * * *   *       **   *   *   *

Human           CCCCGTTGTCTCCGCCCTCTCCCGTCTCTCCCTCGTCTCCCAGATCTCCGTTCATCTCCC 2751
Gallid          TCTTTCTTTTTCTTTTTTTTTACAAGAAATGAGGGGGAAGAATAAAATTGTTTAATTGCT 5267
                 *    * * **     * *  *           *      * *     *** *  * *

Human           CC--ATTAG-ATCTCCGGGACTCCG------AGCAAAGCCGCGGGTGTCCTCCGGGCATC 2802
Gallid          CAGGATTAGCAGAACAGGCAGTCTGTGTCCTGGCCAGGATAAAAATCTGTTTAAAAGACT 5327
                *   ***** *   * ** * ** *       ** *         * *  *      *

Human           CC-----GTGGCGTT-CCCGCCGGCGCCCTCGTCCGCGCCGCCCTTTTCCAAAAGAGTCC 2856
Gallid          CTTTCTAGTGGAGTAATTTCCCGGTTTCCTTTTCTG-AATATACTTGTATAAAAGAATTT 5386
                *      **** **      ****   ***  ** *       *** *  ****** *

Human           CGTC---CGTTC-CCTCATCGGCATCTCCGTCCG-CGCCGTGCATCGGCAGGTCGCG--- 2908
Gallid          TGATGAGCGTTTACCTTTTTGGTATGTTAGTAAAACTGCATTCAATGTTAAATTGTGAAA 5446
                 *     ****  ***  * ** ** *  **    *  * * **  *  *  * * *

Human           --------GCCGCCCTCCGCACAGACG-GCGTGAGACGCCGCGTGAGGTGACGGAGGCGG 2959
Gallid          ATAGAGAAGTAATGTTACAAAAAGAAGTGAAATAGTTACTGGTTTGTATCCCTTAATTGC 5506
                        *      * *  * *** * *    **   * *  *    *  *  *   *

Human           CGAA-----------------------GGGACACGAGAAGAACATG---CAGAAGAACAT 2993
Gallid          TTGCCTTTTTTTTTTTTTTTTTTTTTTAATGCACTTAAGGAACCTGTCTCAGGCAGACAT 5566
                                               ***   * **** **   ***    ****

Human           GAAGACGAA-------------------GAAGACGAAGAAACGAGGACGAAAGGAGGGGA 3034
Gallid          GAAATCATATGATTTTGTTGCACTTCTGAAATGTTTTGTAATGAGCATGTGACTTAACCA 5626
                ***  *  *                    **      * ** *** * *  *       *

Human           ATACACCGGAGA-------CCGAGCGGCGGATGGAGCCCGCTCGGTCTCGCACGTCCGCG 3087
Gallid          ATTTAAAGAAAGTTCATGTCCAGTACACGCATGCGAACCTCTTCCTCGGTTAACAAAATG 5686
                **  *  * *         **      ** ***    ** **   **    *       *

Human           ATACCGAGCGGACTCCGTCGCCGCTCCGGACCCTCCACGCCCCTCCGTCCAGGACCCGAG 3147
Gallid          AAGCCAAGGCAAATTGGCAGAGCCTGGAGAAGCAATGTGACTTTTTGTTCTTGCATCACC 5746
                *  ** **   * *  *  *   **   **  *     * *  *  ** *  *   *

Human           GTCCGA-CACGCCCCCACCTCG-CGCACCTCCTCCGCCACCACCGCTGACAGTCACCGCA 3205
Gallid          CTGAGAACATAACCTCAAGTTAATTTTTCTGAAACGCTGTTGCTACTGTTTGCAGGCTTA 5806
                 *  ** **   ** ** *        **    ***     *  ***   *    *  *
```

```
Human           TATCACCC----CCATACACACCCAG--CAGCAGAGGCCGGCGCAC-ACACACGCGCGGA 3258
Gallid          TCTTCCCTTGTGCCAGAAACCGGCAGATTAATGAAGTTCAGCTTGTTGCGCTTGTTTAGA 5866
                * *  **     *** * **   ***   *    **  * **      * *  *    **

Human           GCACGCACGAGAAC---ACGCGAGACATCGGCCGCCGAAATTA-ACGGAGTCTATGCGCG 3314
Gallid          TTTTTTTCTTCAGTGGAAAACAAGGCGTAAACATGTGAGGCTGTGTACTTTATATGCTCA 5926
                       *   *     *  * ** * *   *    **   *        * ***** *

Human           CGCCGTGACACGGAAAACCAAGCGGAGCGAGACGATCGACCGACTCCTGCTATCCTTCCT 3374
Gallid          AACTGCGACTTTAACAGTTCGGGTAACTGCAGAGAACAGCGGTATTTAAAACCACAACCT 5986
                  * * ***    * *     *   *  *    ** *  * *  *         *  ***

Human           CCCTGGGCACGGTCCACACGCCAGTCTGCGGAGTCACCTGAG---GGCCGGATCCGCTCC 3431
Gallid          GTTGATTTGGAATATCCACAGCAGCTGGAATTCTCCCTTGCATGCAGCTGCATCTAGAGC 6046
                            *   ***  ***   *     ** * **      ** * ***     *

Human           GCGCCCCCCGC---CCGATCCGCCCCGATAAAAAA--AAATCATCTGACTCGTGCCAGTT 3486
Gallid          GAGAACAAAGGAAATTGAAAAGCTTTTACATAGCTTCAAAGGAATAGAAAAGCGTTTGTT 6106
                * *  *   *      **   **    * * *     ***  *   **   * *   ***

Human           CACACAGATGCAACCGATGACAAAAAAAAAACACACCACCACAAGCAAACGCTCGCC--- 3543
Gallid          ACTACTGACCCTAAGAAAGAAAAGCCATCTGCATGCTGCTAAGTTTTAGCACTTTTCCAA 6166
                   ** **  * *   * ** **   *    **  *  * *      * * **   *

Human           -GGCCCGTTCACCGTCC---CTCTCACCCCCACTCGAATCGCGCAGGCGCGTCGGCGGGC 3599
Gallid          TGAGCTGCCCAACTATTGTGCTATTACCTGTCCTATATTTAAACTATTCTGACTTTAATC 6226
                 *  * *  ** *       ** * ***    **  * *    *      * *      *

Human           --AGTCCCCCGAACGCC--TCCACGGCAACGCTCCTCCCACGCCCCCCCNNCCCCCCTCC 3655
Gallid          CAAGTTCCTGTTTCATCAGCCTATGTTTATCCATATGTTCAATCTTAGTTGTCTCTTTCC 6286
                  *** **     *  *   * * *   *  *   *       *        * *  ***

Human           CTCCATCCCCCTCCCCGGAGCAT-ACACGCCGACTCTCTCCGCAGAGGCGGCGACCACAG 3714
Gallid          TATTCTTTTTTTCTTTGGTTAATGATGCATTGACCCACAAGACTGAAATGAGTCATTTGA 6346
                     *     **   **   ** *  *   *** * *    * **   *

Human           CGGCAGCGGAAACTGTAACGGCCACGTTCTTTGAACAGTGACA--GTAACGGCGGCGGCG 3772
Gallid          TGTCCTGAGAA-----AACAAATATAATTCTCATGTGGTGAGAAAGGAGCCATGCTTTCA 6401
                 * *    ***     ***    *   *  *      **** *  * * *   *    *

Human           GCTGTCGCCGTCACGGTAGTCGTGGCGGAGCACCCGGGGAGGCGACGACGGGGACTACCG 3832
Gallid          TCTCTGCTTCTCTTGGCTTTCTTTATTCCGTCTTAAGGAGAGAGGAAACTCGAGCTCAAG 6461
                 ** *     **  **   ** *      *      **   * *   **  *  **   *

Human           ATGCTCTCAGA-TCATCGCCATAGTGAAAGAGTACCGAATAAAACAAGTTAAATTTTGTT 3891
Gallid          CTTTGCTTAACCTTATTTTCCTTGCTTGTAGCAGCAGTAAAACTCAAGCCTG-TTTTCTA 6520
                 *   ** *   * **   * * *          * * * **  ****     **** *

Human           GTAAATAAAAA---AAAATACTATG----TGGAAATTAAAAATAAAAAAAATGAAAATAA 3944
Gallid          ATAAACATTCGCCCAAAGTCTCATGACTTTGTAAACTGTAAGTAAACTGCATGACGGCAC 6580
                 **** *       *** *   ***    ** *** *  ** ****    ****    *

Human           AGGCTAAACACTGACTAAACGTGC-----ATCCTTCTCCGTACGTCCCCTTTCAATAAAT 3999
Gallid          AGGGGAGGAGCTCCCAGATTATTCCAAGGGCCGTTGCAGGAGCAAGGGCTGTCACATGCC 6640
                ***  *    **  *  *   * *       * **    *  *     ** ***

Human           GGCGGACCCGACCT-CTCCATA-AACGGAGACACGCCGGGAGACTCCGGCCCTCCCAATC 4057
Gallid          GGCAAATGTGTCTGGCATCGTGTGGCGGGGTGAGATGGCAACGCTGTGCTGAGTCAGGGA 6700
                ***  *   * *   *  * *    *** *  *    *  *  **  *      *

Human           CACAGGTAGCCCGAGACCTAA---TCACAAAACTGTACCTGAAACCGAAAGTATAGAGGG 4114
Gallid          CCAGGGCAGGCAGCAGGCTGTGGCCTGCCTGGCTCTGGCTGCAGCATGGTGACGGGGCTG 6760
                *   ** ** * *    **        *    ** *  *** * *     *    *   *

Human           CGGCCTCG--AATGCCCCACCTATGAGGCGGGACT---TCGCGACGCGCCACCCTTCCTA 4169
Gallid          CAGCCTCCCCGTTGCACGGCCCAGGCTGCAGAGCTGTGCTGCTGCTGGGCAGCACGCCGC 6820
                * *****     *** *  ** * *  ** *  **     **  *  * ** *   **
```

```
Human           TACGCCCGGCCTTCCGCGTGCGC---GCCTATAG---GAC---GCATCCCCTCGCCCCGA 4220
Gallid          TTCGCGGGGCCCCAGGGATGCACAGAGCTCGTAGTTTGACCTTGCAGGCCACAGCTTGGG 6880
                * ***  ****    *  *** *   **   ***   ***   ***  **   **   *

Human           AACC--------GCAACGCGGCAG---AGTCGGCGCCACACCCCCGTCCCCCGAGTTCGC 4269
Gallid          AACTACTGGCCTGCTGCGTGGTAGTCTGCTTTGGGAGAAGCTGAATTTCCCTAAGCTAGG 6940
                ***         **  ** ** **      *  * *  *  *     * ***  ** * *

Human           GATGGGCTGGCGCCGACCCT----GCCGCGGCAACGAAGGCGAAAC---GAGACCCCGAC 4322
Gallid          GAAGGAGGAGAGTGGGGTCTCAAAGCAATGCCTTCGTATGTGAAATCAGGAGGAGTCTGT 7000
                ** **    * *  *   **    **   * *  ** * * ****    ***    *

Human           GCATGCGGGCATAAAGAGCGAGAAGTAAAC-GCGGGGTCCCGAATTATCGCGAGACTATT 4381
Gallid          CTTGGTGGATAAAAACAACCATCTCAAGGCAGCTGGTCTCCGCAATTCTTCTGGAATAAC 7060
                    * **  * *** * * *      *  * ** **   *** * *    *  ** **

Human           TGCCCGGGCCCGCCCTCGTCCTCATGCCCTTCCCCCCGCCCTCATCCCCCGA------CA 4435
Gallid          --ACAGCACATATCTCTGGCTTCTAATCTTTTGAAATGTCTTAAGGGCCTGTGTGTATCA 7118
                   * *  *    *   * * **    * **      * * * *   ** *        **

Human           CACCCGGTGGAAAAAAACGAAAGACACATAAATAAAACGA-ATTTTGTTGTTAAAAATGTT 4494
Gallid          CATGTTTCCTATTTAAGAGCAAACAAAAAAATAAATCGGGATTATTCTTTTCCCAGGGTG 7178
                **         *   **    * *** * ******* **  *** *  * **   *  **

Human           TCCACGTGACGTCCCCGGTGATTCCCGGAGATGCGGCA-CGCGGCGGATTCGCCCGTTTA 4553
Gallid          CTGGCACAGCAATCGGAGAGCTACCTCCATATTTATGGCGAGGGCGGCCCGACTCTCTG 7238
                    *    *   *   * * * **   * **        ** **  *   ** *  * *

Human           CATCGGGGTCCGCAGACGCGGTTCTGCCAGGGCGTTGCGG-------------TGTCGGT 4600
Gallid          GTGCTGAACCTGATGATGAAGAG-TACCAGGAATTTGAGGAGATGCTGGAACATGCAGAG 7297
                   * *   * *  ** *  *   * *****   *** **              **   *

Human           TCCGCACA-----GAACACCGCGGTCCGTCGTCACCGTTCAACCGCGCGGTAGTTCCGCG 4655
Gallid          ACTGCACAAGTAAGAACAGTGCAAAACAACAGAATTAATAAAACACAGAACAACTAGATG 7357
                 * *****     *****  **    *  *   *    * ** * *      *  *    *

Human           ATTCTATGGTTCATCTCCGCTCACAGCGTTCTCTTTCTTTTATTTTTCACTGAGCCGCTC 4715
Gallid          GAGTAAATAACCAT-TCCAATTAAATGGGAATGGGCCAAAAATCCGTTGGTGCAAGTTCA 7416
                      *     *** ***  * * *  *    *    *     **    *    **

Human           CA-----CCACCAAGAGTGTTACTGA---CTACACCGTATACATGTTTTTTTATCTCTCC 4767
Gallid          CAAGGGACTTCCCAGGGTGGAAGAAGATTCTCCACCGGGTAGAGATACCTCTGGCAGCAG 7476
                **      *  ** ** ***  *        ** *****  ** *  *   * *  *

Human           ACCCCTCCCCATCACAGACCATCAAACACCTTGCCGTATCGACACTGACTATACTCGACC 4827
Gallid          GAGGATGAGAA-CGGAGCTTGTTGAAGCACATTCAGTGGTG-CAGCCAAAGTATTTG-CT 7533
                     *    * *  **    *  **   * * * **   * **   *   ** * * *

Human           GCGGATGACAACGGTCGTTGG-----TCACAACAGAGCTACAGCGGACAGCAAAAATAAG 4882
Gallid          GCAAATACTACTTCTATTTAGAGTTTCTAAAACAGCACAATAAAGGTAGGGAAAAAGTAG 7593
                **  **   *     *  ** *        * *****  * * *  **   * *****  **

Human           ATAAATA---CAGTAAGAGAGAGGCC-CCGGTCCACATAGGAAGCCAAGGGCGGACGAAA 4938
Gallid          GCTAACAGATCAGTGACAGAGGGGGTGCTAAAACAACTACGA--TTGTGGAATGGCTGCA 7651
                   ** *   **** * **** **   *      **  ** **       **   * *   *

Human           ATAAAAACCCGTCCG-------TGATTCACACACGAATCG-------GGGGTTAT----- 4979
Gallid          TTAAAAACCTCCCTGGGCCAGATGATTTTCAGTCAGTTTATCCCCCCAGGATGATACTTG 7711
                 ********   * *       *****  **  *   *           ** * **

Human           GACAACGCGACACACGCAGA-TGAGGGACGGACGCAT--CGCGATC--CGGCGCGACGGG 5034
Gallid          GCTGCCTTGCCAGTAACAGCCTCAGGGGCCAGTGCATGACCCATTCATCAGCCTAAGGGA 7771
                *    *  * **    ***  * **** *    ****  * *  **  * **   * **

Human           GCGCGATTG-GCCCACGCGCGAGCCCGTGC---CCGCTTCGAGTGGCTGCTCCTGGCTC- 5089
Gallid          GCATATTTTTACTGGTGTGAAAATGCAGCCAAACTGCTCTGAATCCCTTTTTGTTACTCT 7831
                **    **   *    * *  *   *   *   * ***  ** *  **  *  *  ***
```

```
Human           -GCGGCAGGCCGTCCAAACTG------TACGGCTATACGAGTCGGCACCGGGGAGAACGG 5142
Gallid          TGTGACTTACTTTCTTGATAGATTAAATACTGCT-TGTGTTACAGCATAGTTTGCAACTG 7890
                 * * *   *  **   *  *      *** *** *  *   * ***  *     *** *

Human           ATCCACCTACCGTGGCCG-CGGTACTGGTGCCTAGAACTCCATCCGGATCCG-----TAC 5196
Gallid          ATGCTGTACCTGAGTCAGATGTTACAGGTGA-TAATTTTTCATTCAGGTAAGGAGTTTGC 7949
                ** *     * * * * *  * *** ****  **    * *** * * *  *     * *

Human           AGGGACGCCAGGAGC----GCCACCGTGTGGGGTCACCGCTGGGGT-TGGCCGCCAACGC 5251
Gallid          ATCTTTTCCAAAAGCTACTGATAATGACTTAAATGAAATCTAATGTATTGTTGTTTGTTT 8009
                *      ***  ***    *  *  *  *    * *   **   ** * *  *

Human           ACGTGAGACCCAGATCCGTTCAAGACTGCGGTGAGTGAGCGCCGCCGCGAC-CGACGACG 5310
Gallid          GTGTTTTTGTTTTTGTTTTTCAAGCTTCTTGCCACTAATTTTCTTTGCCATATTATTCCT 8069
                  **              ******  *   *  * * *    *   ** *    *   *

Human           CTATGCGCACACGGGCGAG-CGGCGGGGAGGGAC--GTCGGCCGCAGGGCGAGCGGGTGG 5367
Gallid          CCTTATCCTGGTATTTTAGTCTATACAAAAGCACTAGGTAGAAACAGGTCATGAGACTGT 8129
                *  *   *         ** *       * * **  *   ****  *  * *  **

Human           AGGGCGAGCGGGTGGAGGGCGAGCGGGTGGAGGGCGAGCGGGTGGAGGGTGAGTGGGTGG 5427
Gallid          TGTTACACAACCAGTAGAAGCTATTTACTACTCACTGTTGTCTTTTAACTGTTCTGCTGG 8189
                 *    *      * **                 *    *  *      **    * ***

Human           AGGGTTCGTCGAAAACCACCGAAGACATTG------------GCTGGCGGAGAGTGGGGA 5475
Gallid          GGTACTTCCTTAATACAAACAAATACTTCCTGCTACCCCCATGCTCCCTCAAATCATGGA 8249
                 *   *     ** ** * * ** ** *              ***  *  * *    ***

Human           CAAAAC-------AACGTCACGTCAGGGGCGGAGAAA---------------TATAGGGT 5513
Gallid          TAAAGCTTTTGGAAATGCCGAGTTAGTAATACAAACAGGAATCTCTGTATTTTATGAGAG 8309
                 *** *       ** * *  ** **      * * *               ***  *

Human           GCGCAGACCGTCAAAGCGC----GAGT---------------------AACGGAAAACGA 5548
Gallid          CCTCCCTCTGCTAAAAAGTTCCAAAGTGTTTTTTTTTCTGTTATTCTAAGTAGTAAATGC 8369
                 * *   * *  ***  *      ***                     *   * *** *

Human           GTACGGATACNGGGGGGGGGGGGAGGAAACATGGTGAAGTCTGTGACAC--GCGTGACCG 5606
Gallid          TTCTATTTTTTAAAGATCCAGTGACAAAAAAAAAAAACTGCTTTAGTCTTTACCTGACTG 8429
                 *     *      *     * **  *** *     *   ** *        * **** *

Human           TCAGCCGGCAACGCCTGCCACGGCCTCCGGCCTCACAGACGAC------TCACGGAGTTT 5660
Gallid          TCAGTCTTTGCTTTGCAAAGTAATCCCTAGCTGCAAAAAAAAAAAAAAGTCAAACACATG 8489
                **** *                  * *  **  ** * *  *       ***   *  *

Human           CCTCCAGA-------CATTGTTTTTTTTT-TTGTATTCATC---GGTAATGCGGTATACG 5709
Gallid          GCTCGAGAGCTGAAATGCTATCTCTGTTTACTGAAACTCTCCCAGCAAATTATACGCATG 8549
                 *** ***          * * * * ***  ** *    **   *  ***       * *

Human           TAAATTCCCCG-------CAAAGGTACTTTCCGTTAAGGACGGTACAGGCGGTATGACTT 5762
Gallid          TAAAGTTCTGAGAGCAGCCAGAGCTGCTTTCCAGAACGCGCAGTAAGATTAAGATTCTTG 8609
                **** * *          ** ** * ******   * *  * ***        **   *

Human           --CCGGAACACGATGATTACGGATATCCTGCATAGTGTACAGG-AGACCGCTTTTTTTA 5819
Gallid          GGCAGAAAAAGAAAATTTTTTGCTAGAGAGCGTTTTGTCAGGACAGGCCAAAATATAGCT 8669
                  * * ** *  *   **   * **    ** *  ***   *  **  **    * *

Human           GGTTCCGTATAAGAC-----GTACGTCGGCGACGTACACACAACG-GTTAAA-AAAATAA 5872
Gallid          AGCTGCATTGACTGCCCTTAGTAAATGAATGGCAT-TGTGTAACGAGTTAAGGAAAATGA 8728
                 * * * *  *   *     ***  *    * * *      **** *****  ***** *

Human           AAAAAAGAGGAAACAGTAAAGCTCCTCCTTTCCTCTGTGTTATCGCGTTCAGTTAGTAGT 5932
Gallid          TGGTGTTATTTACCAGTACTTTTGATTTTATTTTCTTGGCAAATGAATTTGGATCGAAAT 8788
                       *   * *****    *  *  * *  ***  *  *  *  **  * * * * *

Human           CGCCATTGCTGTTGCGTATGCTTTTCGCGTTAACGGTACGCGACAGGATACGAATGCG-- 5990
Gallid          --ATGTTGTT-TTGTGGATGAATAGCCAGAAAAAAATTCTTTTAGAACTACACTTGTGGT 8845
                     *** * *** * ***  *  *  *  **   * *         ***   ** *
```

```
Human           TTACACGCCGCAAAGGCTCCCTACGATGACTCTATG-CGGCATCTCCCGTTCGCGCAGAA 6049
Gallid          TTGGTTTCTATAAAATTCTCTTAACATGAGGCTTTGACAATCTGATTAAATTGTATTGCA 8905
                **     *   ***     * **  ****  ** ** *     *       * *    * *

Human           ACGCGAGTTCCCGGGACGCCAGTCGTGACGTCCGGCCGATGGGA-TGCCTTTGCGTGTGC 6108
Gallid          -CTCATAAAACTGACAGCTCTGTTTTTTCCACTTGCTAATATGACTTCCTTTCTGCTTTT 8964
                 * *      * *  *   * **  *  *  *  **  **  ** * *****  *  *

Human           GGATGTTTTCTGCTGTTTTATAGGAAC-----AGAGACAAGAG-----------ACGAAA 6152
Gallid          GGCTTAAATCGTGTTTTTGCTAGATGTCTGTGGGAGACAGGCCTGCCTTTTCCTATGACC 9024
                ** *    **   * ***  ***          ****** *             * **

Human           ATAGCGGAACGCCCACTATAACGTGCATTTTTCTCTGT--GTTTTGCGTTTTCATGTTTT 6210
Gallid          TTGAGTAAATCCCAATTTTAAGCATAGTTTATCGCTGTTTGTCCTGTAATTGCAACTTCA 9084
                 *     **  ** * * ***      *** ** ****  **  **    ** **  **

Human           ATGATTCTTCTACGGTAT-CGGCCGGAACGGTCATCCTCGATCCTTCTCGTACGCGTGTC 6269
Gallid          TCAACTGTTATGAAGTCTACTTTTAAATCGCGCAATACCAGTTATTACC-TACATCAGCC 9143
                   * * ** *   ** * *      * **  **    *  *  **  * ***    * *

Human           CCGCCCCAGCCCTGGACTCGAGTCTG-----TACGTATGTTGCGGATACGGAGAAAAACT 6324
Gallid          TCTCCCATTCCGCAAGTTTAAGCCTTATGCTTAGGAAAATGGGGGATGTG-ACAAGTGTC 9202
                 * ***   **      *  ** **      ** * *  * * ****  * * **

Human           TCAACCCGTGGG---CTTCGTAAGC---TCGTATCTGA---------CCCACTCCCCGCT 6369
Gallid          TCATGCCATGGAGACTACCGCAAATGCTTTACATCAAATGACAGAAGCCTTCTTTCAGTA 9262
                ***  ** ***        ** **     *   ***  *          **  **  * *

Human           CGA----CACGCTTCGCGTGCTCCTGGTCGGTAGAGACGGAGCCGTGTACGTCCACCACA 6425
Gallid          TAAAGGTCTCCCTTCATCCAGCCTTTTTTGAAAGGCATGAAATTGGGTGTGTTAGATTTT 9322
                  *    * * ****       * *  * *  **  * * *   * **  **

Human           TGAGGGCGGCCAGACTCTGCCGACTGGCGTCGAACGTAACGGAGTTTGCAAGGCGAGGGC 6485
Gallid          AAAGAATAGCTT--CCCCTTCTACTTCTGCTGAGCTGCTCTATGCCTACGAGGCAAAGCC 9380
                  **    **    * *   * ***   *  ** *    *   *  * * **** * * *

Human           TG-CAGCGGGACCCCGTGGCGTATGAG-----------GAGGACCTAGAGCTGCCGGACC 6533
Gallid          AAATGGCTTGACGTTTTTGCGTGCGTGTGTTTTGTTTAGAAAGCTTGTTGCTGCTGTTTC 9440
                     **  ***    * ****  * *           **   * *  ***** *   *

Human           GGCGTATGTGCGGAACGAACGTCAGACATCTGTTCGACGTG-ATCGCCGCGGCCGCCGAC 6592
Gallid          TCAAAAACAATGGTAATTTTGAGAAACAGATGAGAATCTTCCATGGTGAGAATAGACTAT 9500
                     *     ** *     *  * ***  **     * *  ** *        * * *

Human           GAACACGACCTGCTGACCGTCGGCGGCCTGTGTCAAACGCACGCCGGAGTGAGCTGTGAA 6652
Gallid          GAATTTAACATCCAGCCTG--AATGATCTGTAAGGGGGGGAATGCGCTNGCTAGCTTGGCA 9558
                ***    ** * * * * *      *  ****       * * **    *  ****  * *

Human           TTACTAGAGACCGTGCGAGATCCGTGGAC------------GGCGGTTCCGGGCGTACGC 6700
Gallid          GTTTAAAAAAACAATAGGAAAAAAAGGAACTTAAAAGTATTAAACAGTTTAAAGTTTTAGT 9618
                 *   * * **  *  ** *     * **              * ***    *  *  *

Human           ATGACTCTGACCGTGGCGCGGGCTCAG-TATCGCTTGTGGCCCGATGCCCGGAGACAGCT 6759
Gallid          GCCCCATTCAAAATTGCACAGAGTAAAATATTATTTACTTTAAAGAGTCTAGTGATGGAT 9678
                    *  * *   * ** * *  * *  ***   **          * *  * **  * *

Human           CC---------GCCTGCACCTGTACGCGGGACACCCCCTGGGACCGTGGATAGTGTGCGC 6810
Gallid          TTTGTGTCATAAATTGCAAATCTAAGTGACCTGTCTCCTTGAGCGAGGTTTGGAGAAAAC 9738
                              ****  * ** * *      * *** *  *   *  * * *    *

Human           CGTTCTGTCTCGAGAGAGGGAGACGCAGACGCCGTCGCCTCCGATAGGCAGCGGAGGCGT 6870
Gallid          TCATTAGCCTGCAGAT---GATGTGTAAG----GCTGCCTCGGACTTTC--CATTAGCAT 9789
                   *  * **  ***    **   * *      *  ***** **    *  *    ** *

Human           GATTCTGGGAAACGTGCCCACGCCGAGGCCACGCGAAGTGGAGACGGCTTGGGTGATCGT 6930
Gallid          ACTTTAGAGAAAGCCCCTCATTTTAAAGGAACACTCACT-----TCTCCTTTCTGTGTGT 9844
                  **  * ****    * **     * *  ** *  * *        * *   **   **
```

```
Human           CACCCTGGCGGGACCGCTGTTATCGTTCTGGCCCGATAACGGCAAGATCTGCCGTCTGGC 6990
Gallid          CTCTCTG-CATGCTGACCAAC-CCATCCTGGTTACATAA-AGTGAGCTCCAGAGCTACTC 9901
                * * *** *  *    *      * * ****    ****  *  ** **    *     *

Human           GAACTCGTTCGCCGCCCTGTGGAGGATGGGGCCGCGGGCCATGAGAGGACATTGGACGTA 7050
Gallid          TTGCCTCGTGGCTGCTCTTTTTAAAATCGGGTGACTTTGCCCTGGAGAAGGAGTGCGGTT 9961
                   *    * ** ** ** *  *  ** ***   *    *    *** *     *  **

Human           TTCGGCCCCGGGTAGACATCTCCCCGGGGACGCTTGGCCACTCTGTGAACACGTGAGACC 7110
Gallid          TACAAGCAGGGCTTTTTTTTTTTTT-----TTTTTCTCCAAGCTGCACACAATGCAGAGC 10016
                * *   *  ** *     * *            **  ***  ***   ***    *** *

Human           GCCAGTCGGAAAACTTCCCCGGCAAAG-AGCGTACCTGGATTAGACGCCACGGCGGCGAC 7169
Gallid          -----CCAGCATGCTATAGAAACAGAACAACTTGGCCCAGTTTAATACTTCAAACATTAT 10071
                      * * *  **       ** *  * * *  *    **  *  *  *       *

Human           GTAAGGATGCGACCTACGGCCAGAGACATGACCGGAGGAAGATCATGACAAGGATCTCGA 7229
Gallid          TTATGAATT--ATACATGTTTAAA---ATGGCATTTGCTTGCTCTTAATTC---TCTTCC 10123
                 ** * **   *   * *   * *   *** *    *   * ** * *      ***

Human           AATATAAAACCAAAAAAAAAGTGAGGAGTG-CGTAATCGCAAGAAAAGAAAAAGCAG-AG 7287
Gallid          AGTCAAACTCCAGAGCAATGGTGACTTGCATCAAAGCTGCCCTTGGAGCAGGAGGAGTAT 10183
                * *  **  *** *  **  ****   *   *  *   **      ** *  ** ** *

Human           CCGCGGTGGAAATGTCTAT-CCGCCTGCGTGTATGTGTGGGGTGTGTATATGTGCGCGTG 7346
Gallid          CTCCAGCGCAGCCGTTCAAACCAACCTCGCAATTAGGGGAAGTGAATCTGCTCCTTAGTG 10243
                *  * * * *   **  *  **  *  **    *  * *  ***  * *        ***

Human           ACCGTATCCCTCCCTGCCTTTAACCCGATGAGAAATAAAATCGGCGAATGTACACAGTCA 7406
Gallid          CTGGGGCAGCACATTGGTAATAACACAGTGCTAGCACAGCTCCATGTCTCCACACACACG 10303
                   *     * *  **    **** *  **  *    *  **   *  *  *****  *

Human           CACACAA--TGTGCTTACGTGCCGTGTTACTGACATCACATAACAAAAAAAAAACATACT 7464
Gallid          TGCAGGGCTTTTGCTTTTTGACCGTTTAAGAGTTGCTGCTTGTGAAGTGCTCTCCTCTTT 10363
                  **     * *****     **** * *  *      * *   **        *    *

Human           TCCTGTCCGTGAGACACCGCCGCCCTCTAGCGG-GGTTGCT----ATAGCAGCGCCCGAT 7519
Gallid          TCTCTGTCATGCTGAAGCACTTAAAGCTGACGGAGGTTTCTGGTGAAAATGAAGCTTGAT 10423
                **     * **    * * *      **  *** **** **    * *     **  ***

Human           GCCAACGCGACACG--GTGAGTCGCATAGATCGGGACTGCTTGAAAGCGCGCCACATTCG 7577
Gallid          GCCAGCTTCCCTCGAGGTACTTAGCGGTAATGCGTAATGCATGCGCCCGTGCAGTGT-CA 10482
                **** *    * **  **   * **    **  * * *** **    ** **    * *

Human           TCTTTTATATAGACACCCCGGGGAGTGGG--AGGAGCCAACATACACA-CGAGGTCGGTC 7634
Gallid          CGGTGTGCTTTGCTAAGAGGAGAAATGCACCACTATCTATGCTGTATATCTAAGCC--TT 10540
                   * *   * *  *    * * * **    *  * * *   *  * * * * * *  *

Human           TGGGGTTGGAGACGAACGCGGAAAATCAACGGCGTAAAAAAAATAAAAAA-GGCGGAGAC 7693
Gallid          AATGACTGTAAGTGTTTGGGTTCATTAAGTAGCATATTTTGAAGAACTAATGATTTGCAC 10600
                   *  ** *   *   * *   * * *   ** **     ** **  ** *      **

Human           ACATAGCCTTGG--CGGGGAGACGATAACAGGTTCAAAGATAGAGAAAAAAAAAAA----- 7746
Gallid          ATTAGGCTTTGCTTCTGTGCAGTATCCCTGCAGCCAAATCTCCATGTAAAAATATTTTCT 10660
                *    ** ***   * * *               ****  *  *   ***** *

Human           AAGAGGAGACCATAACATTGAATACA--CGTGCGGCGGCAATCCAATCAACGGGACGTGC 7804
Gallid          AGTTGCCTGTGTTACTATCAGTCGCTGCTGTTTGTTTACTTCCCAAACTGCGTCTTGCCC 10720
                *   *       **  **      *    **  *    *   **** *  **    *  *

Human           CGGGGCGCAAAAAAAAAGTATACGGAAGTGCATGCGACAGACAGTCACGCGGACCGACGAC 7864
Gallid          TTGAAGGAATACAAACGTGAAAAAGGGTTAAATCCATAGA-AGTGCTGGGATGCAGCAGG 10779
                  *   * * * *** **  *     **  *  * * *** ***   * *   *  *

Human           AAAGGCCG--ACTCCTAGCATGGCAACTAGGAA--AAAAAATAAGCAACGCTGCAAAATA 7920
Gallid          GCTCGTTGTAAATGATTGAGTGCCATCTGCACACTGAATGCCGGTTGTTGCTGGGACCTG 10839
                    *  *  * *  * *  ** ** **    *   **           ****  *  *
```

```
Human           CTAACAAAGGAAACATCCCTTCACCCCTCCCTACCCCATCTACCACAACCCC-CCCCTCC 7979
Gallid          TTTTTGACTCCACTGAACCTTTACTGGTTGTGGTTTGTCTGGCCATCACATGACTTCTCT 10899
                 *    *    *     **** **    *              ***  **     *  ***

Human           GTAATCCCTTCTGTCCGCGTTTCTCCCACAGGCGCGTGCACTCGCAGACACGCAGGCACG 8039
Gallid          GAAAATTCCCCCTCCCCTCCATTTCCAGTAAACTGTTTGAACAGGAGTGACTCAGACTGA 10959
                * **   *  *   **     * ***    *  *   *  *   * **  ** *** *

Human           CACACACCACCT-----CTATGGCAGTCGCGGGCGGGCAGG-CGGGGAGCATACGGG-GG 8092
Gallid          AACCAGCTTCCCTGTAACCACAGGGCTTGTTAAAGAATTTGTCAACGTGGACTCAAGAGG 11019
                 **   *  **      * *  *   * *      *      * *   * * *  *  * **

Human           GCAGATGTAAAGACAATGAGGAACGGCAT-AGCGCGCGACGTGCC----GCCGTCTCGGA 8147
Gallid          GTTTGTATTAAACTAAGCTGCAACTGCTTCGGTTTGCATTGCGTCAGGAGTTCCACCGAA 11079
                *    * * **   **   * *** ** *  *   **   * * *    *      ** *

Human           CC-CTTGCTATTCTGGCA--CGACGCCAAGGG-AAGCCTCTGGCGCAATCTAACCCTA-- 8201
Gallid          CAACTTCCTGCTCGGAGAGCCGGCTTCTTGTCTAACCCTCTCGCGGCCACACACTGTATA 11139
                *  *** **  ** *  *  ** *  *  *   ** ***** ***    *  **  **

Human           -ACCCTAACCCTAACCCTAACCCTAACCCTAACCCTAACCCTAACCCTAACCCT-AACCC 8259
Gallid          AAAAAAAAATTTCGTCGAACCCCTAGCGGCCAACCGATCCCTAACCCTAGGCCTGAACCC 11199
                 *    **   *   *  * ***** *     * ** * ***********  *** *****
```

Matched sequences: Region A in HHV-6 with Region B in GaHV-2

```
Human           TAACCCTAACCCTAACCCT-AACCCTAACCCTAACCCT-AACCCTAACCCTAACCCTAAC 8317
Gallid          TAACCCTAACCCTAGGCCTGAACCCTAACCCTAGGCCTGAACCCTAACCCTAACCCTAAC 11259
                **************  *** *************  *** *********************

Human           CCTAACCCTAACCCTAACCCT-AACCCTAACCCTAACCCTAACCCTAACCCTAACCCTAA 8376
Gallid          CCTAACCCTAACCCTAGGCCTGAACCCTAACCCTAACCCTAACCCTAACCCTAACCCTAA 11319
                ****************  *** **************************************

Human           CCCTAACCCTAACCCTAACCCT-AACCCTAACCCTAACCCTAACCCTAACCCTAACCCTA 8435
Gallid          CCCTAACCCTAACCCTAGGCCTGAACCCTAACCCTAACCCTAACCCTAACCCTAACCCTA 11379
                *****************  *** *************************************

Human           ACCCTAACCCTAACCCTAACCCTAACCCTAACCCTAACCCTAACCCTAACCCTAACCCTA 8495
Gallid          ACCCTAACCCTAACCCTAACCCTAACCCTAACCCTAACCCTAACCCTAACCCTAACCCTA 11439
                ************************************************************

Human           ACCCTAACCCTAACCCTAACCCTAACCCTAACCCTAACCCTAACCCTAACCCTAACCCTA 8555
Gallid          ACCCTAACCCTAACCCTAACCCTAACCCTAACCCTAACCCTAACCCTAACCCTAACCCTA 11499
                ************************************************************

Human           ACCCTAACCCTAACCCTAACCCTAACCCTAACCCTAACCCTAACCCTAACCCTAACCCTA 8615
Gallid          ACCCTAACCCTAACCCTAACCCTAACCCTAACCCTAACCCTAACCCTAACCCTAACCCTA 11559
                ************************************************************

Human           ACCCTAACCCTAACCCTAACCCTAACCCTAACCCTAACCCATCCCCCAACGCGCGCGCGC 8675
Gallid          ACCCTAACCCTAACCCTAACCCTAACCCTAACCCTAACCCCCCAAATTTTCACCCCCCCC 11619
                ****************************************   *          * * * *
```

```
Human           GCGCCTCTATGGGAGGCGCCGTGTTTTTCACCAAAACGCGCGCCACTGCGAGAGGCGCGT 8735
Gallid          ACCCCCATCCAAAATAAGCCAAATATAATGT-AGAGGATGGGAGAATCCGGGGG---CGA 11675
                 * **  *      *   ***   * *       * *    * *  * * ** * *   **

Human           GAAAAAACCTCCCCTCCCGGACGGGCCCGAGTCGTCCGCGCGTGTCGCCGGTGCGCCCCG 8795
Gallid          TAAGACACTTTCCCACTCATACTGA---AGATGTCTTAACCGTGAACCTAGT----CTCG 11728
                 ** * ** * *** * *  ** *        *        ****   *  **    * **

Human           CGCCCGGGATCCCCCCACCTCCCCCCCCCAAAGAACGGGCGAGGGTATGGGGGTGGATGG 8855
Gallid          ATCCCGAGCTCAAAGCACGGAAATCCACACTGGAACCGGCACATACCACCTGTGATGTGA 11788
                  **** * **    ***      ** *    **** ***             *     **

Human           CGTGTAGTTTAAAGGCGAAGGTGCAG---CCGGGCGCCGGAGCCCGGTGAGAGCGAAAAA 8912
Gallid          CGCAAACTTGGATTATGCAAGTGGTGACTCCGTGGTGTGAAATTAGACCCCCCCTGTTTC 11848
```

```
                **   * **  *    * * ***  *   *** *    * *    *       *

Human           ACGATGGCGCGCGCGAGAGAGAAAGAGAGGCAGAAGGCAGAGCCGCAGAGGGACAGACGA 8972
Gallid          TCGACCCCCCACCCTAACCCTAACCCTAACCCTAACCCTAACCCTAACCCCAAAAACCT 11908
                 ***   * * * * *     **    *  *  **  *  * **  *     * * **

Human           GGAGACGCCGGAGGAAAGAGAGAACGGCCGCGAGGGCCATGAGCTCCACCTAGCGTCAGA 9032
Gallid          CTCGCGGCCGACAGGCAGTT-GTACACCTGCCTGCACTACTACATCCGGTCCGT---AGA 11964
                   *  ****   *  **   * **  * **  *  * *  *  ***     *    ***

Human           CTACGCCACCGCGGCCGCGGTGCGCTCGTTCCCGAGGAGGGCCCGCATGTGG-CGCGAGA 9091
Gallid          CCACATCCCTGCT----CCATCCAATAACTCGAACGCTCTTCCTATAGGTAGATACAGGA 12020
                * **  * * **     *  * *  *   **    *     **   * ** *   *  **

Human           GAGGGAGAAGAGCGGACGCGTGTAGAAACGGCCAACGGGCGGATGCGCAGAGCGCCCCGC 9151
Gallid          CATGTTTTAACGAGGTTCTGCGCTAAATC---CAGGGCGGGAAAGCTCAGCCCGCATCTC 12077
                 * *    *  * **    * *   ** *   **  * * * * ** ***  ***  * *

Human           -CGGGCGATTGACCT--TTCCACAGACCCCCGACCCATTTCGGTGATGCAGATACGCACG 9208
Gallid          GCAGCCCCCGGATCCGATCCCGCAGACCCC-GGCCCACAGGAAGGGGCGGGGCACGTGCA 12136
                 * * *    ** *   * ** ******** * ****       *     *  ***  *

Human           CGAACACGCACAGACATGCCTACGCGGCCTCACCGAGC-AGACTGCCACGTGAGCGAAAG 9267
Gallid          TGGG---GCGTGGCGGGAGATGAATGACCGCGGAGTTCCAAACTCCCGCACCGGCCCCTC 12193
                 *     **   *      *    * ** *   *  * * *** ** *    **

Human           CATACACACACACGAAAAAATACACACAAGAAAATACACACAAGAAAATACACACACAAA 9327
Gallid          TCTGCTCGCTCTCC------TCCCCGCC-GCCAATAGCTACGCGGCAGCGTACAGCCCGG 12246
                  * * * * * *       * * * *  *  ****   **  *  *    ***  *

Human           AAAATACACACACACAAAAATACACACACACAAAAATACACACACAAAAAAATACACACA 9387
Gallid          CCAATAGGCGCGC-----GGTGGGCGTAGGCGGAGGAAGCTACA-AGAGCCCCACGCGGG 12300
                  ****  * * *       *   *  *  *  *   *   *** * *     ** *

Human           CACAAAAAAATACACACACACACAAAAATACACACACAAAAAAATACACACACACAAAAA 9447
Gallid          GTTCCCCCGGCACACGTGGCGGGTGGAAGGCTC-CGCTGTGTCTAACCCTAATCGGAGGT 12359
                          ****             **  * * * *         ** *  *    *

Human           ANNNNACNNNNNNNNNNNNNNNNNNNNNNNNNNNNNNNNNNNNNNNNNNNNNNNNNNNNN 9507
Gallid          ATTGATGGTACTGTCGCCGCGCTCCCTCCGCCCGCTGTTTACTCGCTGACTTTCAGCGGG 12419
                *

Human           NNNNNNNNNNNNNNNNNNNNNNNNNNNNNNNNNNNNNNNNNNNNTACACACACAAAAAAA 9567
Gallid          CTAGGGGAGCCGCCCCAGGGGGCGCCGCGGCGGGGAGGGGGTGGGGCGGACGCGGGAGAA 12479
                                                             *  ** *   * **

Human           TACACACACACAAAAAAAATACACA-CACACAAAAAAAATACACACACAAAAAAAATACACAC 9626
Gallid          AGGAC---CGAAAGGGGCTCCACGGCAAACAAAAAAAAACGT-CAGCGAGGGGTCCTCTC 12535
                   **   *  **     * ***  ** ********* **   **    *    * * * *

Human           ACACAAAAAAAATACACACATAAAAAAATACACACAAAAAAAAAACCATTTTATTTATGCGC 9686
Gallid          GCCCCCA----TCCGCCCTGGGGTCCTCGCCCGCAGGCCGCGGTCGGCCGG--CACCCGC 12589
                 * *  *    * * * *           * * **         *         *  ***

Human           TGTTCTCACAATGTTAACATAGGACAAAGACAACCACGAAAACGACCACAACA-ACAACA 9745
Gallid          CATTGCCGCCGCGA-AGAGTTCGCCTCTGTCAGCCTCGGCGGCGCCCGGGAGATGCGGCG 12648
                  **  * *   *  *   *  * *   * ** ** **    ** **   * *  *  *

Human           GCAGCAACAGCATCAACTACAGCAACAGACGAGGGCAGGATCACGAGGTGTCAACTCCCG 9805
Gallid          CGCGGCCCCGCGCCCCCAGCAGAGCAACACGGGAGCGGCGCCCCCGGGG--CAACCCCCG 12706
                   *   * **  *  *  ***    * *** * ** *   * *  **   **** ****

Human           CATCTCC----GTCGCGCAACGTGTCCTCGCAGTCCCTACGGACTACGGTAGAAGCCCCG 9861
Gallid          CGCCCCCCTGCGCCGTGGGGCGCGCGGACGGCGTCGCTCCCA--CACGCGCGGCCCCGCG 12764
                *  * **    * ** *   ** *    **  *** ** *      ***   *   ** **

Human           CAAGAGACTGCGGACATTTCCCGCTCTACCATGTGCGGAGAAAGTTGA-TAGACGGCAGG 9920
Gallid          CGCACGACCGTTGGAG----CCGTTGAGCCGCGCGCGGGGCTCTGTGAGTAGACCGAACG 12820
```

```
                *    *** *  *       *** *   **  * **** *     *** ***** * * *

Human           TAGATTTATCACAGGCGCGTGCACGCAC---GTCACGG-GCAGCTGCATGGTCCACAGAT 9976
Gallid          GGCCCCCCGCGGAGGTGGGCGCTCGAGCCCGGTCCCTGCGCAGGTG-GTGCCCGCTGGGC 12879
                         *  *** * * ** **  *   *** * * **** **  **  *    *

Human           GTCCCGGAGGGAATCGATGGTTCTTGAAGAGTTTT--CTGACACCCA--TGGCGAGAAAC 10032
Gallid          GCCGCTCGGGGCTACCGGGGTCCGTCCGCGGCCGTGCCGGGCGCCCAGGCGCCGAGTCCT 12939
                * * *   ***   *   *** * *     *   *  * * * ****   * ****

Human           GCCCTGAAGCTATCGGACAGGCGCACCACGGCTCCCCCGTCGAGACTGGCCGCATACACC 10092
Gallid          GGCCTGGACGTGT-GGCGGTGCGCAGCGCGGAGCCCT-GTCCCGGCAGGAGCCGTCCTCT 12997
                * **** *  * * * **    ***** * ***  ***  ***  * * **   * * * *

Human           CTGCCGACCTGATCC---ATGAGCACCCGCTGTCGTCTGAGCAGAGGTAAAGTAACGCCG 10149
Gallid          GCCTCGGCACGCTCCGCAATAAGCGTGGGCAAACGTGTGGGCCGTG-CAGGGCATGAGCG 13056
                    ** *  * ***   ** ***    **   *** ** ** * *  *  * *    **

Human           CGTCCGAAGAATCTGCC---GATCACCACCGCGACGCC-TCCGCCGGGTATGAGCCGCCA 10205
Gallid          TGCACAAAGACGTGGCCCTGGGGCTTGGGCTGAGCGCAGTGCGATGCCCCCGGGTCATCA 13116
                 *  * ****    ***   *  *     *    ***  * **  *     * * *  **

Human           CCTCTTCTCCGCCCTCCTGGGTACGGCAAACTCAAAGAAAGCTCCGCGCGGCCAAGGCAA 10265
Gallid          CCCGTCCCGCAGATCCCCGTGCACGGGG---TCAGTGCCATCT---TGTGGTCTCGGCTT 13170
                **  * *  *     ** * * ****     ***  *  * **    * ** *  ***

Human           CGGCACGAGAGCCTTATCGAAAGACACGGCGAAAGCCAGGGCGTCGTCAACTCCACTCAT 10325
Gallid          TCTTTTTTCCCCCTT-TTGCACGAAGAGTCAGTGAACTTGGGGTACTTAATCGTGCTTTA 13229
                           **** * * * **   * *      *  ** **  * **     **

Human           GCCGCCGAAGAAAGAGGCGCCGGTTTC-CATGAGACACTTGGAACCGTAGACGGGCTCGA 10384
Gallid          ATTGCGCGATGGAGAACCGTTGCTAGAATATGTGGGGATAGAGAAGTCCGATACCCTCAG 13289
                   **   *   ***  **  * *     *** *    * *  *     **    ***

Human           AGCGACGCAAGCCGTGCTTCCAGAGACCGAACGCGTCCTCCGCGAGACGGTACAGCGC-G 10443
Gallid          AAATGTGCGAGTCCTGCGGGTAGAATCGGCGCA-GCACTGAATAAACCCGCGGGGCCCTA 13348
                *     ** ** * ***    ***  * *  *  *  **     *  * *    ** *

Human           TCATCGAACACG-CCGTTAAACG--CGTATATCTCCCCGCACT-CTCCGACCAGCAC-CA 10498
Gallid          AAACCTCTCGCGGCCGACAGACAGTTGTACACCTGCCTGCATTACTACATCCAGTTCGTA 13408
                  * *   * ** ***  * **    *** * ** ** *** * ** *  ****  *  *

Human           GCGCGGAAACTTCTGCGAA--GATAGTGACCCCGATGAC-------CGTGAGATCCATAC 10549
Gallid          GACCGTATCCCTGCTCCATCCAATAGCTACATTATCGATATATGTACGAGGAGTCAAAAT 13468
                *  ** *  * *   * *    ****  **      **        ** *   ** * *

Human           CGGGACAGCACCCGTATCCGCGAGACAGCTTGGC----CAACTCGGCCTGCGTGATCCCG 10605
Gallid          CGGAAAAAAAAGTGCCTTCTTTTAATTACAGGAGGTAGCAATTAATCAAAGACAGATATG 13528
                *** * *  *   *  * *     *   *  *      *** *   *            *

Human           AGCAT--GTCGGAAGTGGCG-AATCTCAGTTCTGCCCCC-ACGGGCCACGAGAGCGCCAA 10661
Gallid          GGAACCAATAGTAGGAGGTGTGGGCTCAATTGTGTTTTTTACCGGTCCCACG---GTCGA 13585
                 * *    * * * * ** *    **** ** **      ** ** * *  *   * * *

Human           CCTGCAATGGGCAAAAGTAGAGA-CGAAGTCCCTCACGTCGTCCGCCGATAGCGGCCGGG 10720
Gallid          TTTGCCACCATCTTCTCCAACCATCAGATTTTCCTTCCTTGTGCGATGCTGTCGTTCTAG 13645
                  *** *    *      *   * *  * *  *   * * ** **  * *  **  *  *

Human           C-----GACGAGAGTCCGAACGAACGCCGTCCATTTAACCTCCGGTACTGCCGCATCGCA 10775
Gallid          TTTTTTAATAAACTGCTGTACCCAAGGCGCCTCAGGGTCCACACATACCTTCCTGTTCTT 13705
                       *  *    * * **  * * ** *       ** *   ***   *   *

Human           CCGCAGGTCACGTCCCGCAGAGAAAGAGCACGAAGAAGGGCAGAGA-GCAGC-AGAGGCC 10833
Gallid          CTTGAGAGCAAAGCTACAAAAGGGAAAACCTTAACCAAATTAGCTTTACAGCCAGACACT 13765
                *   ** **   *    * **  * * *   ** *    **     **** ***  *

Human           AGACTCCAAGAAGATATGCCGAGAAACGGA--GAGCGACAAAACATTTTT------TTTC 10885
Gallid          ACAC-CTAAGATGACAACCCCATATTTTGGCCAAGTAATAGAGCTTACTTACATAATTTC 13824
```

```
                 * ** * **** ** *  ** * *    *    **  * * * * *  **       ****

Human            TGTGCGGAATCCGAGATAAAATTAAAAGGATAAAA--AAAAACGATAACGGCAGCGTTAC 10943
Gallid           AGTCCTCC-TGCAGTGTATACCCGGTGGTATCACGTCAACAGCGATAATAGGCCCCAAGC 13883
                  ** *    * *    ** *       * ** *    ** * ******  *   *    *

Human            CGGCAGTCTCACATAAACGTCTACGATCACGTTAAGGACGCTACAACTGTTAGAAACAAA 11003
Gallid           CAGTAGGCCGAT-TAGTGACTTTCACGCACTTGCACCTCTTGTCGACAGCGAGACTCTC- 13941
                 * * ** *  *  **      * *   *** *  *   *    * ** *  ***  *

Human            CAACGTTGAATCGACAT-ATTCCCGACGT-CTTCCACGAAAATGGAAGTGAACCGACCGC 11061
Gallid           CAGTGATATGCCTACATTATTAAGAGCATGCATTTATCAGAACAACACCATCCCCCGTGC 14001
                 **  * *    * **** ***     * * * *  *  * **    *     **    **

Human            CCACAAAACTGTGAACACATCCCATCCCCGGGTAAAATATACAATGATCCCAGACGTCAA 11121
Gallid           TACCTTTCCTTATACCACCATTGCTGCACATGCCAGAGACGAAACC-CCTCAGCTTTTAC 14060
                    *    **   * ***      * * *  *  * * *   **    * ***   * *

Human            AAGCGCCCGTAAGAACTCGATCTACCACGCTTC-CATTCTTTTTTTTTAAAAAAAAAAC 11180
Gallid           GAGTACTGGTTACA--TCAGTATAGCGTATTCCACAACCTCCAGTCTAGAGTTCTCTTCC 14118
                  **  *  ** * *  **  * ** *    * * **  **    * *  *         *

Human            CTAACCTTCAACGTTAGGGCGAACTATCGAACTACGTCGTGCACGGCAC---TCCTAGTG 11237
Gallid           CCTACTTACCATTTCCAGGCAACAAATAGATCTGTACTATGAATAGAACCAATACTAGCA 14178
                 *  ** * * *  *   *** *   ** ** **      ** *  * **   * ****

Human            CCGGCGTCCGGAAATT------CCTGAC-GCTCATCG---------TTTCTAGCCTTTAC 11281
Gallid           ACAACGCCTGCATTTTTGCCGCCCCAACTGCTCATCAAACCCACACCCCCTGCATTATAT 14238
                  *  ** * * *  **      **  ** *******             **    * **

Human            AAACAGCAGATCATCGTCGGCCTGCCTC---CAAGTTTTCTGATAAAACATTCGCGAGAG 11338
Gallid           ATCCATGGAACCGTACCCGTAATGCCTCGGGCGATTTCCCTGTTATTGGGTCTCCACCAA 14298
                 *  **    * * *   **   ******   * * **  *** **     *   *   *

Human            -ATGACAAGTCGGAAGAAGCGCCCATTCGGGCTGCTCCCTACGGAAAACGTAGCTCTTAT 11397
Gallid           CACGTGATATTGGA--GACCCCCAACTTAAAATTCCACGACCGTGAGATGCATTTTGTTT 14356
                  * *  *  * ***   * * ** * *     * *  *   **  * * * *  *  * *

Human            AATACTGAATGAC-TCCTATCCGTC-TAAACTGCTGAAAATTTTCCGCCACAAAGCGCGG 11455
Gallid           ATTGAAAATTTCCATTCGATGGGGCATAAACTA-TAGCATCGAAACACTAAAATGAACAG 14415
                 * *    * *  * * * * **  * * ******  *   *      * * * ** *  * *

Human            CCGTCCGTCTTC----GTCGTTGTCGTTGAATCCGAAAATCCGTCCGTCGTCGGCCACGT 11511
Gallid           GAGTTTGCATCAAAAGGTGATAATACTGGAACACAAGACTATGAGGACCCTTAAATGTAT 14475
                   **  *  *      **  *  *  * ***  * * * * *   *     * *        *

Human            AGAGAGAAATCCGACCGGCGTCCGCAAAGTACTTGTAGCCGAAGCTGCCGAAGAAACACA 11571
Gallid           ATGAGAAAATTCCCACGACCCCTGCATAGCAG--AAAACCAGGACCAC----AATAAATT 14529
                 *     **** *   ** *  * *** ** *     * **    *  *     * * *

Human            TTTCCGCGAGCCTGCGCCGTGCCTCGTCGCACCTGACGAATACAAAAGAATCCCGCCGCC 11631
Gallid           TTGCAGTACGCATGCGCC----CTCTACACACGACCCGCATGCGCAGACCACTTACTGC- 14584
                 ** * *   ** ******    ***  * ***    ** ** *  *      *   * **

Human            GATGAAAGAGCAGAGTCTCGTTCTTGGGCCACCTCAGGGCGAAAGAACGACCTATGTGCC 11691
Gallid           AGTACGCATGCGCCCTCTACACACAAAACGCATGCGCAGTCCAAAACGCATGCGCAGACC 14644
                   *      **    ***           *      *   *   ** *   *        **

Human            GGTCTCGAAAGCACAGCACGTCCCGACAAC-TGCAGACGCCGTCCACCTCGGTGTCCTCC 11750
Gallid           GCTTACGGCAGTAC-GCATGCGCCCTCTACACACAAAACGCATGCGCAGACCAATTACCC 14703
                 * *  **  ** ** *** *  **  * **   ** *   * * * *       *   **

Human            ACCACAAAC-TGCAGCGATGATACCAGCTGATGTGCGTAATAGAACCTCTTCAGACCTTT 11809
Gallid           AAAACAAGCATGCGCCGTAAGTATGGTTATCTGCGCATGCCTGGTTTTCGACACACGCGC 14763
                 *  **** * ***  **    **         ** ** *     *    **  ** **

Human            ACGAAGAAAATCG-TCAATGGTGTCACCGACGCG----GCACAGTTGCTGCG-GCAATAA 11863
Gallid           ATGCGCAGATACACTCCACCGTA-CGCATGCGCGTCATGCATAACTGGCGCATGCGTGAA 14822
```

```
                * *   * *  *  ** *  **  * *   ****    *** *  **  **  **   **

Human           CCACGTATGCGCATAAACGCGACCGGCACCGCCGAGCAGAACGACTATGTCACCTTTCAG 11923
Gallid          CGCCTTACCCGAACCAGCCCTGC--GCGCTTGCGCTATATACGCCCTGCGCACTCATCGC 14880
                *  * **  ** *  * * *  *  ** *   **      *** *     ***   **

Human           AAACCAGTAAGCCGGCTCCGATCCGATCTCCCCCCTTTTCTTCACGTACCCCAGTACGAC 11983
Gallid          GCATGCGCACAGACGTTCAAAT--AATGGCGGACATTTCCGTCAACAAACGCACTAAGGT 14938
                  *   * *     * **  **   **  *   * *** * ***   * * ** ** *

Human           GAGGCGCTCC-CGGCAACACACTAGATC--GGCCAGTTCGCCCAACTTTTCATCGGCATA 12040
Gallid          AAAAAAAAAAACCACGAACCGTTAGTTTTTAGGAGCTACGATTTAATTTTCAT-GCTCTG 14997
                 *         *  * *  *  *** *    *    * **    * ******* *   *

Human           TTCATTCTCGTCTCTACAGTTGTTA-ATAACCAGCTCGTATCCTTCAGGCCAAGCCA--- 12096
Gallid          CTTTATCAAGACTCGAAATGCGTTACAGCTTCCCCCGTACCGGCACCGACAGTTCTTTA 15057
                 *   **  * *** * *   **** *    *  * **** *      * **   *

Human           ---AGGGAATTCGCCTGCCGAAATTGACGAACGTAAACATTCGGACGCTATTCAGGCACG 12153
Gallid          CGTAAGCCCTTCCCGTTCACTCTTTCACGCGCGGCACTATCGGTACAACAGTGGCACACA 15117
                   * *   *** * * *     ** ***  **  *  **  * **   * *    ***

Human           TATAGACGCGCAAGAGATCGACGAGATC---TCTCT----------TGGACTT---GTTA 12197
Gallid          TCAAACAAAGTAAAAGGGGAAGGGAATTGAATTTCTACAAAAGATCTAAATTTTACGTAA 15177
                *  *     * ** **    * *  **    * ***          *  * **   ** *

Human           TAGAGACGTTGACGTTTCTTCATCCTCTCCTGAGACTGCAGCTCGTCATCCCCACACATC 12257
Gallid          TGGATCCCGTCCCGATCGTCCCCTCACACGTGGCACTGCCGCGTAA-ACAATGCCACATC 15236
                * **  *  *  ** *  * *   * * * **  ***** **     *      ******

Human           ATAGAATCTTGAGCCGGCTCATGAAGGCCTTCGATAAATCGCACCGTAGCCGTAGCCAAA 12317
Gallid          GTAGAG----AAAGCATAAGGGGGAGGCCATCGGGAGATTATCCCGAGAATTCAAACTA- 15291
                 ****      *  *       * ***** ***  * **    ***       *  * *

Human           ATCTGCCGTTGCGCCGAAGAAACACGCCGGCTGTTTGTCGTTTCCATTACGGGCTCACAG 12377
Gallid          --TTCTTGTAATGTCGTACGAGC-CTCGTTCCGTTCGCTCTTTCATGCGCAGCATTACCG 15348
                   *   **   * ** *  * * * *   * *** *   ****     * *  * ** *

Human           CGACAGA-GAAATAGCACTCAAAACTTAGTAAGCAAAAGTTGCAACTCGCAAAAAAAAAA 12436
Gallid          GTGTAAATGTAATAATGCCGCACAGTGAACAAGCAAAAGGGGAAG------AGAGTCTCC 15402
                    * * * ****   *   * * * *  *********  * *       * *

Human           TGAAAGATAATATAGTCACAAAAGAAACTTGTCACAATGTACACC--CAAAAAGAACGAT 12494
Gallid          GGATAGCCGTTACAATC-CAGTAACGACTTAACGCAGATTACTCCTGCATAAGCGTCTCT 15461
                 ** **    ** * ** **  *   ****  * **   *** **  ** **    *  *

Human           ATGGATGATCACACGACAACGGCAACAATAATATCGTTTTTTTTTTAAAGCTAAACAAA 12554
Gallid          GCGATGAGGTATTTTCCATATGTTCCTGGAGGTCCGTGCCTGTATATTCGTTAATGTCGA 15521
                  *       *     **   *   *   *    ***   * * * *      *     *

Human           GTGAAAGAAGCGTACCGGTTTTCCGG---ATTCGTCTGTATCAGATAAAGGTCACACAAG 12611
Gallid          CATCTGCCTATGTGCCTGTGTATCGGTGCATAGGAGTATGTCTGTGCATCCCTGTATATA 15581
                           ** ** ** *  ***   **  *  * * ** *   *       * *

Human           CCAGCTTAGGAACGCGGCGCGCTTCG----CGAATGTCTAACCCTCGC--TGTTGCACGC 12665
Gallid          TTATCTGTGCATATTTGCATAATTCATAAACGAATATTTAATTACAGTAGTGTTGCGCGA 15641
                  * **  * *     **    ***     ***** * ***     *   ****** **

Human           GTCTT---------ATAAGCCTACGTCTG-AGGACT---ATTCGCTTTTGACGTCTCCGG 12712
Gallid          GCCCCGATCCATTGATCTGCTCACGACTGTAGCACTTAGAATCGCACAGTGCACAGCCAG 15701
                * *           **  **  *** *** ** ***   * ****      *    ** *

Human           TGCTCCTGCAACCTGATGTCAAACGAGCCGCCGCCGCGCACA-GCGTAAGGAACGACGTT 12771
Gallid          TGAGCTGGCCACAGGAGAGGGACATAGGCCTGATTGCTGGGACGCGGATGAGAGATCAAT 15761
                **  *  ** **  **     *   ** *      **    * *** * *  *   *  *

Human           CG-GATACACCCCACTCCA--CATATGGGTCCCGTAAAGAT---TGCAGTCGTGAAAACG 12825
Gallid          CGTGTTGTTCCTTACGATAACCTAATCAGCACCACCGAATTATACGAGACTGCATTAACT 15821
```

```
                ** * *   **  **   *  *  **  *  **    *  *    *     *    ***

Human           AAAAAACCTCACCGGATTCGACATGTAAGATAACTCCAGAGTATTGAGGAAGCCCTCCAG 12885
Gallid          CACATCTGCTAAAGGTTTCGCTTATTCCTTTTACATCCGAATGTCCATCCTGTTATCTTT 15881
                 * *      *  **  ****    *    * **  * ** * *  *    *   **

Human           AACATCGTGCCTTATCTCGCGAGCGAGTCGCATGGATGCACACAGGTCGCCTAG-ACAGA 12944
Gallid          AATATTCCTTAATGTGCGATGTCCATATCCCTTCTAAAAAAAAAAACAGCCAGGCATAGT 15941
                ** **       * *     *  *   ** * *  *   * * *    ***  * * **

Human           TCTTTTTTAGGCACAGATGCTCCAAGAGCGCACGAACGCGATGCCGT------TCCTTGT 12998
Gallid          TTTTGAGCCTGGAAAACTTACCGCAGGCTTCATAGAAGCCATATCCCGAATAATACTCCA 16001
                * **      * * * *  *   **    **   * ** **  *        * **

Human           CATAACCCGATACGAGAATCGTCACGATCTCAAGAAAGAAAAAACACAACCGAGGGACGC 13058
Gallid          TATACCCCCCCTCCCCTTCCCCAACCGTTCTGTATAGAACGAGAATTTGCCATTTAAGCA 16061
                 *** ***    *      *   **  *        *  *  * *     **     *

Human           GCATGGGATCTTTA-ATCTCGAACTGAGAACTGACAAC-TAACAGTTTGATCCTGACACC 13116
Gallid          GTCTGGGGCCGAGAGATGTTGCCCCAGAAGTTTTCCACATAGCTAAGTTTATCTCATACT 16121
                *  ****  *   * ** * *  *    *  *  * ** ** *    *    ** * **

Human           GTGGAGGCCAGAAACGGTATCGATGCAACGCTCCATTT---CTGAGATCGCTTTTACGAA 13173
Gallid          TCGGAACTCCTGGA-GCCAACAAATCCCCTGACCATGTAACTCAAAATAGTTCTTCCGAG 16180
                  ***   *    * *  * * *  *  *  ****  *      * ** * * ** ***

Human           GAAACGAGCAATGGCCGTGAGA-TCACTTACGATGCGCTGTTTGACGCGTCTCAGCAGAA 13232
Gallid          TCTAAGCTACACGGTAAGGAAAATTTGTTACCCCAGAGGATTTTTT-TATGTCAGTA--A 16237
                   * *    * **    ** * *   ****         ***      * **** *  *

Human           ACAGACACAGAACGATGTTGCAGTACACCTTCAAGATGTTACGTTCGAAATGCCCTCTCC 13292
Gallid          ATCGATAAATAATGCCTTTAACCCTTTCCTTTATGTTGATCTTCCCGAAACTATGAAAAC 16297
                *  ** * * ** *   **        **** * * ** *     *****         *

Human           CCAACTGCTTATCGTGAAGAATGGGCAAATGCCGCCTTTCCATAGAGTTAAGAATAAGAG 13352
Gallid          TATTATATATAACTAGGGGAGAAGAAACATGGGGCATAGACGATGTGCTGCTGAGAGTCA 16357
                     *   ** *  *  **   *  * ***  ** *   *   * * *    * *

Human           AAGAACCAAG-ATCTCGGTCAATCTCCGCCCGGAGAGC----GACCGCTTCACGCGCGTC 13407
Gallid          CAATGCGGATCATCAGGGTCTCCCGTCACCTGGAAACCACCAGACCGTAGACTGAGTATC 16417
                 *   *  *  ***  ****   *  * ** *** * *    *****      * *  **

Human           GAGGTGCAGCTG-GCCTAAAATTAGATCAGCTGGATCGAATTCGG----AAATATCAGAT 13462
Gallid          CGAGGGAAACTGAGTATAAATCTGGCCCGAATACAAGGAATCCTGTTCGGGATCCTCGGT 16477
                   * * * *** *  ****  * *  *   *  *  **** * *      **    * *

Human           ATGTTCACGGCCCGGGAATCTTTCTTACCC---AGATAGATCCGAAAGAGAGACGCCCGG 13519
Gallid          AAG---ACGAGCATAAAGCCTCTCCGGCTCCGGAGCCGGATGTGGGGGAGATGGGGTAAA 16534
                * *   ***  *    *  ** **   * *   **   ***  *   ****   *

Human           CACTG--ATCGCAACTACAGAGCACAGAAGA-CACAAAGCGATAAGAAGAAGCGTACGCG 13576
Gallid          AAGGGGAACCTGGCCAACAGGACAAAGCTGAGCGTAAACCGTCCCCGGCGATGGAGGGGT 16594
                 *  *  * *    * ****  ** **  ** *  *** **         *  *   *

Human           ACAAAGAT--GTAATCGGACAAAAAGTGTTTCAAGAGCACACTCGTCGGAGACGCCGCGT 13634
Gallid          ACACGGCTCGGTAACAGGACACAATGCAGGGAAGATGCCCTCCGGAGATGGAGGCTGGGG 16654
                ***  * *  ****  ***** ** *      *   ** * *  *     ** ** * *

Human           CG--CCATAGATCTCCTTGGGATTGGGCAAAATATCGAACAAGGTCAACAGAGTATTGGA 13692
Gallid          AGGGCAGAAGAGGGAATGGGGAGTACAGATGGGAGGTGGTGGGGTCGA-GCAGAGCTGGG 16713
                 *  *   ***     * **** *    *    *        **** *   **   ***

Human           CTCACTCCTGTTCCTCAGCATAAGGAAC-----TGCAGGAAGAGGG-----AGTCCAACA 13742
Gallid          CGCAAAGCTCCTCCGCATCGGGAGGAGCGGGGGTACAGATAGGAGGTTGGAGGGGGGAAG 16773
                * **   **  *** ** *   **** *     * ***  **  **      *    *

Human           ACTCCAAAAGACGGTTGAAATGCG---CCGAGATAGTATCCCTCTTCTCCCCATCGACGA 13799
Gallid          GCCCCGGAGCGTAGATAATATGGGGAGTAGAGATGGGAGGTGG-TGGGGTCGAGCAGAGC 16832
```

```
                 * **  *     * * * *** *     ***** * *      *     * * *   *

Human           TGTACTCCAGAATCGAGAAGTGCGCCAGAGCGGTGCACTTGGTCAGGAGTCGATAAAAAG 13859
Gallid          TGGGCGCAAAGTTCCTCCGTATCGGGAGGAGGGGGGGTACAGATAGGAGGTTGGGAACCG 16892
                **  * * *   **        **  **   ** *      *  *****      **  *

Human           CTGACGTTTCGTCCTCAAAAGTTAACCGTTCTAAGAGAGACATGAAAGC---CGAGAGAT 13916
Gallid          GAGCAATGTGGAGCGTTAGGTTCATCCGGTGAGGGAGGTGGAGGAGTGCAAATGGGAGGT 16952
                  *   * * *  *   *   * * *** *    ***    * **  **    * *** *

Human           CCATGGTCGTCTTTCCACGCCTTTGGAATTC-CACAGAAACTTTCTCCGCCAGAGCCGCC 13975
Gallid          TCAGGAACGGGATCGTGCGGGGTGGTAAGCAGTCCAAGGGTCACCGTTAGGGGTACCGCC 17012
                 ** *  **   *    **   * * **      **        *       *  *****

Human           -CAGAGAACACTA--TCGTCACAA---AATCGTAAGCCGCGGGTCATGAACAGATCCTCT 14029
Gallid          ATAGGGCAAACTGGCTCATGACAAGCCAACTGTACACGCAGGGACGTGCACTCAGTCCTT 17072
                  ** * * ***   ** * ****   **  ***  *   *** * ** **  *  *  *

Human           GCGGTCAACGTGCCGCTATCTA--TAATCATCTCCTCCGTTATGTCTTTCAGAT-CAACA 14086
Gallid          AGATCTCGAATTTCCTTACGTAGGTGTTCATTGGCCCTCTGCAGCTCTTCACATGCTTCA 17132
                          *  *  **  **  *  ****   * *  *   *   **** ** *  **

Human           TCAAGTTCCCTGGAATGGTGAA----CGGTCAGCGAGAGATAGGGATGG--TTGGGAATG 14140
Gallid          TGGAGTTTGTCTACATAGTCCGTCTGCTTCCTGCGTCTTCTCCGAGCGGCGTCACGATTC 17192
                *  ****       ** **       *   * ***     *  *   **  *   ** *

Human           GTATACGTGACCCGTTCTCGAACGGGTCCCTTATAAATGTCGTGGTC--CAATAACTCCA 14198
Gallid          CTTTTTCTCCTCCTTTCCAGCTTCTGTTTCTCCTCCTCAGATAGGCCGTCAGGGAAGGGG 17252
                 * *   *   ** ***  *     **  **  *         ** *  **   *

Human           TCACTCAAGTAAGTAT-ATATCTGAGACAGCGTTTATTTTTTTGTCGCAGAAAAAAACAA 14257
Gallid          TGTTTGGAGGGGCTGTTGGGGATGTCGTGACTTTTCCTTTTTTTCCGTCTCGAAGTCGAC 17312
                *   *  **    * *      **      * ***  ******  **     **    *

Human           ACGAGCAGAATTCAAATTGCAGGAGACGG-CCACGTACATCACAATCGAGAAAACACACA 14316
Gallid          CCGAGAGAAA---GATCGAGGGGGGACGGATCGTCAGCGGGACTGTAGGGCATAGCGCCC 17369
                 ****   **    *      ** *****  *     *   **  * * * * *    *

Human           ATCACTCATCCCCATCATGTCCATTCAGTTCGCCGGCACAGTCCGGTGCATATTCATA-A 14375
Gallid          GGCTCTGGCTCCTGAGACATCTCTTTACACCTGTACCGTGCCCGCCTTCTCCCTGGTATA 17429
                  * **    **    *  **  ** *   *     *     *   * *    *  ** *

Human           TACTTGTAATCATC-CCTGAGGACGT-CCAACGACCCCAATTTATACCCGATAGCTCTG- 14432
Gallid          CACCTGCAAGAGACGCCTGCCTAGGAATCAGTGTGCGGAATTTATTCTTAACATTCCAGC 17489
                 ** ** **    * ****   * *   **  *  *  ******* *   * *   * *

Human           ACGTCTTTCTCGGGACTTATTTGAACACCC------CGATGATTACCACTCCCAAAATAT 14486
Gallid          ACCAACCTCCCCGAACCAAAATAATAATTAAAAAAGCAACACCCACAGACCCGAAAGTAT 17549
                **     ** * * **  *  * *  *         * *     **    ** *** ***

Human           TTCAAGC---CACTGAAAAAAAAAAGCCATAGTCCCGTTTATGCACCTGGTGTTAGTTAAA 14543
Gallid          TGAAGAATTACACGTTCGCGAACGGTCACAATTC-ACCTGT-CATTTTATAAAGGAGCAA 17607
                *  *      ***       **  * ** * * *    * * **  *  *    *   **

Human           TAATCTTCGGCGACGGGCAACACTACCCGTTGTAGCCGCTGTAAGAAATTCTCTTCATGG 14603
Gallid          TAGTTTATTTAAGAGGTAGGTATAAATCGAT-CATTCCCCCCCCCCCGTCTCCGTATCAC 17666
                ** * *        **     *  *  ** *  *  * *         *   * *

Human           GTATTAACGATAAAAAAACC-CGGAATGAGATTATCAGAAGACACCAGGTCACGATCATT 14662
Gallid          TCCCGAACCATTAGATATCAGTCGATCCAGCCCCCACGTCATGCATGACTATCGTCTTT 17726
                     *** ** * * * *    **   **     **    *  *  *   ** **

Human           GTGCTCGTGGAAGCGTTTTATATGAAGATGATGCAGAA--AAAACATTTCCATGA---AC 14717
Gallid          ATATCACCGAATTCGGTTATTCCAAATCCGATTCAATATTACATCACTTCAACGGTGTAT 17786
                 *      * *  ** **  *   **   *** **  *  * * ** *** * *    *

Human           CGA-GTCTCGTCGTAGTCTTT--GTAAGCAGGAAC---GGAAAAACCG-ATTCTCAGAGC 14770
Gallid          AGACGTTCTACGATGGTTTTCCAGCGATCATTTCCCCCAGAAAGGTCATGCTGTCAGAAC 17846
```

```
                 ** **       * ** **   *  * **    *    ****   *    * ***** *

Human            TTCCCAACACTTTAAGATAAACTGACTCTTTTCCCCGGGACACCTAACAAT---TTCTTT 14827
Gallid           CATTCGTGGTCACCGAACGAAGGGTTCCGATACAGCATTAAACTTCACAGGGGACATTCA 17906
                     *           *  **  *   *  * *  *   * ** * ***        *

Human            CATTTGTTTCATCGAAGAAAAGTAACACAGGTCTCCGAGGCACCCGTTCCGCCCCAGCTT 14887
Gallid           AAACAAGCCCAGAGCCGTCACGTGGAACACGTCTC-GAGTCGATCGCTCCCCTCAAGAAA 17965
                  *       **  *  *  * **   *** ***** *** *   ** *** * * **

Human            CATTTCTTCCTCCATGGTCACACACAATTTTCCCACTTCTTCCGTGAAGAGGTCGTAATA 14947
Gallid           GCTATTCTGCGGCAGTCCGTTCCAAGACCTTACAAACGGACTACCGGGCAGAATACAACT 18025
                   * *  * *  **        **  *  ** * *          *   **     **

Human            TATGTCCAACTCAGCATTTATGACACGGTAGAGGGTGTTCACGACGAACACAATGAAAAC 15007
Gallid           TTCTTTTAGGAGAATATACAAAGTACAATGC--GGCCGTCGGATCGCTCTCGCGATACGC 18083
                 *   *  *    *  **  *    **  *    **   **    **  * *     *  *

Human            AATGTTAGTTAAGAAGGTTAGCAATGCCTTCTTATCCGGGGTGACAGAGATCTGTAGCGA 15067
Gallid           GGTGTCA-CGAAGACCGATA-CACTACATACAACTTAAAAATG--TGAACCCCCCAGCAG 18139
                   *** *   ****  * ** ** * * * *   *      **   **   *   ***

Human            AATAGGCAGCCTACTGTGCTCTATCTGACCTTCAATCATGCTCAACCCTAAATCCTTCGC 15127
Gallid           ATTCTATT-TCTAGCGGGAGAGGTTTCACATTTTCATGCCGCCTGTAGTGAATCCT--GA 18196
                 * *       ***  * *     * * ** **          *     * ******  *

Human            TATGAGACTCGACAGACTCACACTGTAGGGTTCACCCAAATGCAGAAAAGCCGGAAGACG 15187
Gallid           TCAAGAACAATGCAGACGACAGGTACTAAATTCGTC--AGTACGTGGCCACAGTAAGTCT 18254
                 *     **    *****      *      ***  *  * * *       * * *** *

Human            AATTGAACCTATCATAGGAAAACACTGAATTCGGACACAGT-GGCCA---AAATCACACT 15243
Gallid           GGCTCATATCATCGCTAAGGACTTTTGGCCTCGCGTACGATCGGCCACTCGGATCAGAAC 18314
                    * *    ***       *    **   ***   **  * *****     **** *

Human            TCCTGCCTCTCTTGTTATACATGATAAACATGCGCC-TACACTCGACACAACCACACAGG 15302
Gallid           -CCTCCGTATTATGCAAACCTTTGGACATTTACATTATACGGAGGGCCTTGATCGGCGAG 18373
                  *** * * *  **  *  * *   *    * *    ***    * *         *  *

Human            AGCATAAAGTGCTCG--ATGAGACAGGATTCGATACGCTCAAAGAAATCGTATCTGAAGA 15360
Gallid           GGCATTATCCCCTCTCCATTAATTATACCGCCAGGCCTTGATCATTTTCCCATCCTGCTC 18433
                  **** *    ***   ** *   *     * *  *  * *      **  ***

Human            AGACATCGGATTTGTTCTCCAAGCAGTCCTTCAATTCTTGACCCCAGTGACAAAG-ATTA 15419
Gallid           ACCCACCGT-CCCATATCTCAAACAACGCG--AATTTGTAACACGGGGGGGGGGGGGAAGA 18490
                 *  ** **      *    *** **   *   ****  * ** *  * *     * *  *

Human            TAACCTCCCAGCTGCCGAGTAGATTCCTTTGTCAACTCTTTTAACTTGCTTTCAAACTGA 15479
Gallid           AAATAATGTATCGATTGTATTTATCGCACCAACTATTCTTTTATCCTAACAACAACTATG 18550
                  **      * *    *  *  **  *     * * ******* * *     ***

Human            AATAATAACATGGAGGCGTACTCACATAGATAGCGACACCAGTAGCCCAGCGTAGGTTTG 15539
Gallid           TCTGAAACCCTGAAGATGTTTCAATACCACCACAAACAGTAGACGACCACGATACACACG 18610
                   * * * * ** **  **    * *     *   ***  **  * ***   **     *

Human            GACGCGAAGTTAAACAGAGACGCTCCA---GCATAGAGACCGATAAAAGCCAGTTTAGTG 15596
Gallid           TATGT-ATTTTAAACCGTGTCAATGTGATCGCATACGGTTC-ATGACAGTTGATAAACTT 18668
                  * *  * ****** * * *  *      *****  *  * ** * **    *  * *

Human            ATGCACTGCCACTTCTCATCAAAATTGTCCATAAATTTCAAGTTTTTCACCATGTCAGCA 15656
Gallid           TTAAAAGGCTCGAGTCCGTCTCCCCCCTCTTTTTTTTTTCACGTTCTCCTAATATTGGTT 18728
                  *  *  **       * **       **  *   ***  *  ** **    ** *  *

Human            ATCTGATCGGCT-CTGACGTAGGTGGAAGGATACTTGT--AAGTTTCATACTCATAATCT 15713
Gallid           A--CAACCGACTGCTCACACACATACAACCCCCCTCCCCCACGATGCTCATTAGCCTTCA 18786
                 *    * ** ** ** **  *  *  **     **     * * * *  * *     **

Human            ATCAGTCTGTTCCACACTACGCAGTCCGAAGAGGGATGTAGTCCTTT---AATGCCAAAT 15770
Gallid           AAAATTATATTTTGAAGTATGTGAGTCGGGGCACTTTTTCCTGCCCTCGGAGTTTACAAC 18846
```

```
                *  * * * **    * ** *     **  *     * *  * *  *   * *    **

Human           CTA-GCTGCTGCGGTTCCCTGCAGACCGTTCGTCAACC----TAGCGCAGGAGACGGGAA 15825
Gallid          GTACGATACAGTGAACTCCGGGGGGAAGGAGAAAAACCGCTCCGGCACAAATTATATATC 18906
                 ** * * * * *    ** *  *   *      ****      ** **    *

Human           CGACAAACCTGCTGGTAGCGGGCAGTCGACCGACGAATACCGGCGTGAGAAATTTTGTGA 15885
Gallid          CGAATGAGAATTATCTCGTGCATCGCCCGCGTACGGAAATGTATATGAGTAGACTAG-AA 18965
                ***   *        * * *    * *  *  *** * *      **** *   * *  *

Human           TAAATTTAACCGTCGGCGAATCCTCTTCGTCCAGACGCACCGCAAACAGAATTTTGCTGC 15945
Gallid          AAAGTTTAAA-ATCTACTTTCCCATTTTGTTT-GGCCCACTTCAT-TAGA--TCTACC-C 19019
                 ** *****   **  *    **  ** **   * * ***  **   ***  * * *  *

Human           GGTCTTTCACGAGCCTGTTGTAAGAAAAAACAAGTTAACGGAGAGTCTTCATCCTCGAAG 16005
Gallid          GATATGTTACACTCCCGGAGATCAGGTCTCTTAGTTATCCGGGAATGATTACCTTACCCG 19079
                * * * * **   ** *  *            ***** * * ** *  * * * *    *

Human           AATTGTCTTTTCGGGAAAAACGACACACAACACATCTACCCACCTGTCCTTTCGCACGCA 16065
Gallid          CACTTTGGTTCTAAATGTCACGATCGAAAAGCCGGCTGGTAGAGGGATCGGGCGAAAAGA 19139
                 * * *  **         ****   * **  *  **        *  *   ** *   *

Human           CATATGCCGAGGCTTACTGCGGAGAACGGGCAACTCCCCATTCAACATCCTATTAAGATT 16125
Gallid          TCGGGGAGCGGACGGACTGAGCCTTAAGTAGAATAACTGGCGCG-CAGCC-----AGATC 19193
                     *    * *  **** *    * *   **   *     *  ** **     ****

Human           CTCCGTCTCAGTGTCACTTATATAAAATAAGGT--ATCGT-CATTCCAGTCCAATGAATA 16182
Gallid          CT--GTATGAAAGGTATGTATTAGAAATTCGATTGGCCGAACAGGGCAATTCGCGCACGA 19251
                **  ** * *  *  *  ***   ****  * *    **  **   ** * *    *  *

Human           CTTCTCTTTATAGAGCA-ATTCATCAAATCCTCTACGTATAAAAGTCCTCAAACTTTTA- 16240
Gallid          TTATGCGTAATCAAGCCCACAGATCGCACCTTCAACCGATAAGAGTCTCTAGATGACAAA 19311
                 *   * * **  ***  *   ***  * * ** **  **** ****   * *     *

Human           --GAGACTTTGCTGATTCTGTTTTCTACGTAGATATAGATGTGACAGTTCGAGCCCATAA 16298
Gallid          TCGACAGTTAGTTGGAAA-GTGTTCGGGGGCGGTAAAATTTACAGGGCCTCTCGACATAA 19370
                  ** * ** * **      ** ***   *  * ** *  *   *  *        *****

Human           CGATAGCAGCTCTGCAATT---TGGTTCAGCTTGAGAGCGGGCACACGTCCCTATGATAG 16355
Gallid          AGGCCTCTAAAGTATAATTCGCTGGTTCTGCTAATTAGGAAGAGCGGTTCTGAGTAAT-- 19428
                 *    *     *  ****   ****** ***    **   *  *   **    * **

Human           CATAATCCTTTTCTGTGATTTTCTCTAGAATGCCAGGTCCAGTCAGATAATAAAT----A 16411
Gallid          CGTCACACCGATCGCATATTTATGCTG--AGGCGAGGGGTAATGAGATGATAAATCAGCA 19486
                * * *  *   **    ****   **   * ** ***   * * **** ******    *

Human           TCCGACAAGGAACAAAAGGAAAAGTCAAAGTCCTGGAAAGCATCCACCCTG-ATTCTCTT 16470
Gallid          CTTAAGAAAGGGCATCTGTCTA--TCAGGGCCCCAGACATCAAAGGCACTGCACTATAAG 19544
                    * ** *  **   *   *  ***  * **  ** * **    * *** * * *

Human           GCCACAATGTTTTGCTACGTAATCCTTCAT-AGCCGGGAGATTCCTCTGTAAA--AGGAT 16527
Gallid          GTGATTCACTTAAGCTAAAATTCCGGGCATCAGCTATGAGTAAGCAATATCAGTTATCAA 19604
                *  *     **  ****      *   *** ***   ***    *  * * *   *  *

Human           AAATTCCTCTCGGCCGCATTTAGTCTCTCCGAACCACATCTTTTCCTGCTCTGGATCTAA 16587
Gallid          AAATTTCTCATGGGAACAGCGACTT-----AAAAAAAAACTTATAATAAAAGGAAATACC 19659
                ***** ***  **   **   * *       **  * * *** *  *     * *

Human           CTCCGCTTCGGCAAAGGGTGGAAATTGTCTAAGTCCGATATTGAAGAACTGGACCAGTGA 16647
Gallid          TTCAATACCCAATGACGTTATAAACAATTCCGTATAAATCCTGAGGAGCTGCCGCAAAAA 19719
                 **     *     * * *  ***   *         **  *** ** ***   **   *

Human           ---TTCTGCTATGATGTACACTTTCTCCGT-CACCGTGTCCACGGCAATGGTCTT--TCC 16701
Gallid          AAACTACCCCATTTCGCGCCATTGGAACGTATAGAATAACAACAACAAAAATAAAAACGC 19779
                    *   * **   *  *  **    ***  *   *  * **  ***   *       *

Human           AGTTGAACTA--TGCATCACGTATGGATCGTAGTCAGGATTCGGATCACGATATATCGGG 16759
Gallid          AGTCGGCACGGTTTCATAATTCCAGGATCAAACCCGGCCGAACGCTCTCGAAATATAACA 19839
```

```
                    *** *       * *** *     *****  *  * *      * ** *** ****

Human               TGAGCGTACCC-----AATGGCGGACAAAAATTCATCAGTATC--CAGAAGACTGCTC-C 16811
Gallid              CACCCCCTCCCCCGCCGCTACTGGTCCGGAAATCATTGAGTTTAGTGGCAAACGACAAAC 19899
                        *   ***       *   ** *   ** ****     *     * * **  *   *

Human               AGTCATAG---GCACTTACCCATCTATGCGTGTGAAAGTGATAAAACTCACCCAGAGT-- 16866
Gallid              AGACAAAAATTGTAGATGCCGCCAGCTAGTTGAGAAGCGCACAGAACATATATCGAGACG 19959
                    ** ** *    * *  * **      *   ** ***    * * ***  *    ***

Human               --TAATGCAT---ACATGGGAGGCCAGGCGATACAA--CATCGCAAGCCCGGAGAACTGA 16919
Gallid              CATGAAGCAACCGACACCGCAAGTTAATTTACGGCGGTTACTGTACATTTGCAGTAACTT 20019
                      * * ***    ***  * * *  *    *        *  * *     * ** *

Human               TCTCCAACCCGTCTCCGTAAGCTGCA-GACAGCACGTCTACATCAAAATCCAAAGTAGGC 16978
Gallid              TTTTTAACCT-TGATCGTACCGTACGCGAAGGGGAGTATACGA-AAGATAAAATGAACAC 20077
                    * *  ****  *   ****   * *  **  *   ** ***   ** **  ** * *  *

Human               ACACATGATGACAAGGTGAACTCATCGTTGCAG-ACCGGTACGATTT-CTGTTTGAAACA 17036
Gallid              GCATACAGTGAACCAATATCCGTAACCTTTTAATGCTCGACCGATTTACAGGATGAGGCT 20137
                     ** *   ***     *   *  * * **  *   *  *  ****** * *  ***  *

Human               TAAACGCAAAATGAAGTACAATACTTTCACACAAGACTCGCTTTCCGCCCTCCGTTTCAA 17096
Gallid              GAAAAATATACATACATTCAAAATAATTAGAAAAACAAAGACAATGAACGTCCCCGCTAC 20197
                     ***   * *   *  * *** *   * * * **     *        * ***     *

Human               GTATCAGACGAAAATTCGACATGAAC--TACTTACTGAGGCTCATGCACGATTGATAAGT 17154
Gallid              ACGTCCGCGTTCGTCTCGGCGACCGCCGTACTTCCGCAATCGTTTACAGAAGCGTCATAT 20257
                       ** *        *** *     *  ***** *  *  *   * **  *  *  *  *

Human               CTCCGACATGCCCAACGTCGACATCCGTCCGAAGCGCATGGATATTTTTCCAATCTTCTC 17214
Gallid              CCGTTCTATTTCCGCAAACAAAGACTTTGAGACAGGATTTCCGTGTTGTCGTGTCCGGTC 20317
                    *      ** **     * *   *  *  **   *  *      ** **   **   **

Human               ATAACCGCAGTCCGCAAAAAGTCTGGCAAATCCCACACGCAAAAAAGTCAGTAGACAATC 17274
Gallid              ATA--CATTCTATGTAAACAAGGAAGTTA-TCCCCTTTGCTTCCGTATTA-TCGATAATC 20373
                    ***  *    *  * *** *     *  * ****    **       * * * ** ****

Human               GCCCAGCTTCACCAGCACGTTGTCTATCCAATCGTAATAATATATTTTTCCAGATTTCCC 17334
Gallid              GGCTCCGATCCCGATTCCGCGGACCAAGCATTGGCTGCAA---ACTATATCGGAATGCGC 20430
                    * *     ** * *   **  * * *  ** * *    **   * * *  * ** * * *

Human               TATACATAGAATAGGTCTTGTACATTTAGACTCATAATCATGGTCATTCAGGTAACCTAA 17394
Gallid              CAGACCTTCTTTGCG-CGTCTCCAGACACTTCTGCGATCGCAGACG---AAGAAGCCTGA 20486
                     * ** *    *  * * * * **   *        ***   * *    * * * *** *

Human               CACTACTACTTTTTCTACTAAGTCTTCCTGTTCTTCTAGATTTGCCAAGCGACATGTCCC 17454
Gallid              CGTAGGTATGATTTTTCCTA---TTACCTATATTTCCCA--CCGCCGAATGATACAAATG 20541
                    *     **   *** * ***    * *** *  ***       *** *  ** *

Human               TATCCAGACACTG-TCACGAGTCCCTAGAGTCAAACTCTTAAAAACCGG-CATATCCAAT 17512
Gallid              GGGCGAGATACTTCTTTTTAGAGTTAACAGTTCCATTTTTCTTACATGCTCAAACCTAAA 20601
                       * *** ***  *    **     * ***   * * **   *   *  ** * * **

Human               CTCAGATCCCGGCCGACGTTGAGAGACACGAAACGTTCCACATCGTCCAAACTGGCACAA 17572
Gallid              CATGACGTCACACCGA-ACAGGCGGACGGCAAACTTTCGCCATGTGTACTGCTTGTATGC 20660
                    *       *   ****    *   ***   **** ***  ***        ** * *

Human               CGGGCCAGCTCATCAAACTCTCGCAACATGGCAATGTAATGTTT---CCTCATAGC-CTT 17628
Gallid              TACAACGGCCAATCGTGTTCCACATGCATGCGACTGCA-TGTTTAGGTCTCACCCCTCTC 20719
                         * **  ***    **      ****  * ** * *****    ****   * **

Human               GATGCCCAAAACGCCTTTCCTATTCAACTTCAGATGGATATTCAAAGTACAGGGACTCCT 17688
Gallid              GAACCCTACAACATCTCCGCTCCATGTGATCCGAAGGGAGATCGAAGATCGTAGACTCTC 20779
                    **  ** * ***  **   **        ** ** **    ** ***  *   *****

Human               TA-ACACATCATTATTCCT-TAAATTTTCCAGCGATTCGAATCTGGGGCCGTCATACAAG 17746
Gallid              TACATACACCGGCCTCAATATACCACGACCGACAGAGAAAGTTGGGGAAGGGTAAGCGAA 20839
```

```
                   ** * *** *    *   * **      **  *      * *  ***   *  *  * *

Human              CCATGACGGTACACAGGCTCCGCGCATGTGATGATGTTAGTACCCAGGACGCCGTATCTT 17806
Gallid             TCGCA----TACACACTTTTCCGGTAGATGTCAATGTGCAAAAAAAAAAAGCC----TCG 20891
                    *       ******   * *  * *  **   ****    *   *  * ***

Human              ACAAATTGC--TCAAAATTATCACACACGAAGTGGAGAGTATCCGTCTCT-AGATCGTGC 17863
Gallid             AGAAATTGAGATGCCAATTATTCCTGCTAATTCTAGGTATACTCAAATTTCATACCGT-- 20949
                   * ******   *   ******  *     *      *  **  *   * * * * ***

Human              AAGAAAATTCTTTTAGATCGGCCGATCAAAATTGGAACATCTCTGTAAGGAATCTGAATA 17923
Gallid             -TGAACTTCAATAAACAGTGATCTGTACGAAGCATTACATTCTTACA---GACTTATATG 21005
                     ***  *   *  * *  *  *  *   **     ****   *  *    *  *  **

Human              GTCCCCAGTAGACACAGT--TTTTCCACAAAACCCTGATAGTGATCATT-CATATCTTCA 17980
Gallid             TTCGTGCGTATATACAGACGTATTCTAAGAATACATAACTATACGCAGCGTCTTTCTGCA 21065
                    **    *** * ****   * *** *  **  * * *   *   **     * *** **

Human              AACATCTTTTCTGTATATCCAGCTACATCTTTACGTAGAGTGA--ATCTCAGCCAGCAGT 18038
Gallid             GTTATCTAATGCGTAGCTTTGATAATGTGCAAACAGCGACTCATTATCCCGCCTAGCTAC 21125
                      ****  *  ***  *      *  *    **   ** * *  *** *  * ***

Human              T-CCGAGGCCAGAT--AATAGACAGACACCAGTTCTTATACGTGCCGACAAAATTCTCGA 18095
Gallid             TATTTATACTGGATTCAGAGGGTCGATTCCACTTCTTTCTCTTGTGGAATCTCTGCCAGA 21185
                   *    *  *  ***  *   *   **  *** *****   * **  **     * *  **

Human              TTCGAGAAAGATCAGCAGATTCACAGAAACGACACAAT-TCGGCAA-----GAGCGTTCT 18149
Gallid             ACCTCACAGCTCTCGTTTCTACTACGCCATGTTACAAAGTTGGAAATTAAGGAGCGAGCC 21245
                     *    *      *    * *   *  * *  ****  * ** **     *****  *

Human              CGTTCGGAATCTCACCTGA---GACCTCCTTCGCCTCGAGGTCCATCTTGAAAACTCCGA 18206
Gallid             CTCTCACAAAAACTTCTGATGCGATCACCAAAACTTGATGTTCCGTTCCTAGTTGTCGCA 21305
                   *  **  **   *  ****   ** * **    * *   * *** *    *    **  *

Human              ATAACA--CTAGAAGTGAAACAATGAAATATCTGAAATATAAGACCTCGATGCGAGACGC 18264
Gallid             GTATCCGTTTCGAGATGGAA-AGTGGGTCCGCAGTCA-ATGCATCCGGGGTCTGCAACGA 21363
                    ** *    * ** ** ** * **      * *  * **    **  * *  *  ***

Human              CGTCACA-TGACAGGAAATTGGAAAAAAGAGAGTGGTGTGACAAACT-TTATTAAATTTC 18322
Gallid             TATCACAGCTGTAATCAACACGGATGGTAGGTACACCGGCACACTTTGTTAGTAGAACTT 21423
                     *****     *   **   * *      *      *  ***   * *** ** *  *

Human              AACCCCCGTTTTGACTCACACGCTTTCCTCCGCATGTACAGAAAAATCCGTTCCACCAAC 18382
Gallid             AATAGTTTTTTTTTTTCAG-----TCCTGCTTCATATACACGTTCTTAATTCTCACTCAC 21478
                   **      ****   ***      * *  *  *** ****      *   *  ***  **

Human              GATTTCCTGCGAATGTTGGACAACAAACACCTTCATTTCCTCGGAATTATGAAAATCTTC 18442
Gallid             AAAATCCAACAAA---------GTAGATATGTTC--TTCCTGAGGCATAGAAAAGTCCAC 21527
                    *  ***  * **           * * *  ***  *****  *   **  *** **  *

Human              CTTACATACATCAGAAGCAAAA-GTATATAAACACTCGCATTCCGAAAGTTTTATGCATT 18501
Gallid             ATTGC---CACCACAAATAGATCACGCATGGATATTTACCTCTGCGAAA--------ATT 21576
                    ** *   ** ** **  * *      **  * * *  * *     **         ***

Human              GCGATTTGACATAGCTGCAATCCGCATTCAAACCTTGCACCCCGTACTTTAAGCAG-GAG 18560
Gallid             GGGATCCCCCAAAGC-ATAATCCCGGATGATGAATCGAGCTCCGT--TCGAAGCAGTGAT 21633
                   * ***    ** ***   *****    * *    * *  * ****  *  ****** **

Human              ACCTCCATCGTTCCGGCTTCTGTATTG-----CACTCCTCGGATTTCGATTTAGAAAACA 18615
Gallid             TATTCTTTCTGGGGGGGCACTACCTGGGTAACCAAACCTCATTTTTTTTTTTTTGAGGGA 21693
                      **  **     **   **   * *     **  ****   ***   *** **    *

Human              GCTGTACGACTGTCTTCCATAACTTTCTAACAGCCATCGGCAGAT-CCACTTCAGAAAAA 18674
Gallid             GAGTGAAATTCAAGCTTCCCGACACACTTACTAAGTCAGGTAGAGACCCCAACCAAAATC 21753
                   *    *         * *   **   ** **       ** ***  ** *  *  ***

Human              ACGAACTCGAAGCGCCACTAAAAACAGATGGAAATAGTCACATACAAGACGGCTTCAGCG 18734
Gallid             GGGTGTAGGGAAAGTCCCAGAATGAACTTGGGATTATATAGATTGCAGGAGTCTCTGCAA 21813
```

```
                  *     * *  * * *  **   *  *** * **    * **    **  * **

Human           AGATCTCCGACCGTCACCTGG------TCATCGGGATTCGGACGAG-CTATAGCCTCAAT 18787
Gallid          ACATGGGGCGAAGCCACGAGGGAGGAGTTGTCGATGATGAGACAAATCTACTGTCTCATG 21873
                * **        * ***  **      *  ***     *  *** *  ***  * ****

Human           TCAAAAAAGGCATCAAGAAAATATCAGGA-AACCCCTACGCTTCTACAGCGGTCTCCTAC 18846
Gallid          AAACATACCCCTTCCCCCGCTTCCTATCTCAGCAGAAAAACTCATATAGCCTTCCATACT 21933
                  * * *   * **       *   *    * *    *  **  ** ***  **

Human           ACTGCCTTATAAAACAGTATGAGCATTGCCTCGTCCCGCCAAATAAATCAATACGTTTCG 18906
Gallid          ACCGTAGTATAAAA-ACTATAGATAATCCCCTCTTCTG--AGTCTAACAGTGACATTACC 21990
                ** *   ******* * ***    * * **   * * *  *    **     ** ** *

Human           ACAAAGGGAAGATTGAAGTCGCGGCCCTAATTCTAGATCTCGG-ACACCAGGTACTTGGT 18965
Gallid          TCATAAAAAAAA---AACATATACACCTGAGTAT-GATCACGATACGATTTATTCTCAGT 22046
                 ** *   ** *   **        *** * * * **** **  **      * **  **

Human           CGTCAGATACACGTCCGACAACGTATCTACAGCTGGACTAG-CATCACACTCCCTAAACT 19024
Gallid          TGTTAAGGTTCATTGTGCCAACATTTTCAGACTTTGCCCCCTCAGCTAAATCCTTTGAAG 22106
                 ** *        *  * **** * *  * *  * * *    ** *  * *** *  *

Human           CTTTACTCCCAGAGA-ACTTTACTTTTTAGTT--GCCTCTCCAGAAGACGAAGACATCGT 19081
Gallid          CAATATTATCGTTGTTACGTCGTACATTAATCTCACCTCATTAGTACACATTCAATCAAC 22166
                *  ** *  *   *  ** *      *** *    ****   ** * **    *

Human           ATTTAATCCAACTATAACAAAGGGAGGGTGGATCTCGGGAAGTTTTTCGTATCCCGTTGA 19141
Gallid          ATGTTACATATCTATAGCC---TGATGATACATCTC---AAATTAATCCCACCTCATT-A 22219
                ** * *   * ***** *     ** * *  *****   ** **  **  * * * * ** *

Human           ATACAGATCAAACTTTTCGCTAACCGGAATGTCGGCTAAC-GTATTAATGGTGCCGTTCG 19200
Gallid          GTACACATTCAATCAACATGTTACATATCTATAACTTGATTATATCAGAGGTGTCAACAG 22279
                 **** **  **        * **     * *    * *   *** *  **** *    *

Human           TTCC-GTATAGATACCCACTAAACTATGCTCGCTTCATATCGTCAATAGATCTGATGATA 19259
Gallid          CTCTTGTACAGACA-----TATCCAATACAGGCCTCCTA-CACCTGCAGTCTCAAAACTA 22333
                 **  *** *** *     **  * ** *  ** ** ** *  *    **     *   **

Human           CTAAACGAACAATTTCCGGAACACGAATGCGGAGACATACAGATTTTAAAGCAGCGAAAC 19319
Gallid          TTATATCAGCAATACACACT-CGGTCATTCCATACTAAATCAAATCTGTCATGGC-ATAC 22391
                 ** *  * ****   *    *    ** *      * *   * * *      ** * **

Human           TACCTCTACCTGGGAGTCATCAAAAATCTTACATGGAAAAAAAGCGTCACCGGCACAGGT 19379
Gallid          CGTTCCCACCCAG-AGCCGTGTCAATCGACAACTGAGAAGGGTTTCTTTCTGCCGTAGTA 22450
                     * ***  * ** * *   **     *  **  **       *  * * *  **

Human           CAGACAGCTCCACATAGAATCCTAAAAGCTTCGTTCATAGGAAGTTGGCCGGACACCTCC 19439
Gallid          TTTAT-----CATGTACAAAGACGGAAGCGCGG------GGACGTTGATGGAGGAGTTGC 22499
                   *      **  ** **      ****   *      *** ****   *   *  * *

Human           CTGCCTGACAGAGTAGCACTGCGTTTCTTTAATAACA--CTAGGTTTA-CGATACACTGC 19496
Gallid          AAACCTGACGTCACAAAACAGCTACGCAGCAAAAGCAGGTAAAGTATAACCATAATCGTG 22559
                   ******     *  ** **    *   ** * **    * ** ** * ***  *

Human           CACGAGTTCGCAATTAATATTGAAAATCTCGGTCTCGTCAA-AAATAAAGAGAAAGTCTT 19555
Gallid          CAACAACGCCCAAATAGT-TTAAATGGTGCAAT-TCGTCAATAAATGAAAGTTTCATGTC 22617
                **  *   * *** ** * ** **     *  * ******* **** **       * *

Human           TGGGACACTAGCCACCGTATGCTGCGAGCAAATCCCTTCTCTACTAACGACTGAAAATCT 19615
Gallid          GTGTATTTATTGCATCATATCTTAACAAGGGAGATTAAATCAA--AAAAAAAGAGATTGC 22675
                  * *       ** * ***  *   *    *       ** *  **  *  ** * *

Human           ACCGAGATATTTAATAGTTCAGTTTGAAGTAGTAACGCAAATCG-AAGATCCCGAACCCC 19674
Gallid          TAGGTCGAAAGCACTACCGAAGTCGCATTTAGCCATGCTCCTTATAAGACAGTTTTTTTT 22735
                   *    *   * **    ***   *  ***  * **   *   ****

Human           TGTTATTTTCCAGTAACCCGAAACTATATTTTACAGGAGACGT-ACTAAACGCAACGATG 19733
Gallid          TGTTTTTTTTTGGGGGGGGGGGGCAACTCGTAAGGCTTCCCGTCACTCAAAGGAATTACT 22795
```

```
                **** ****   *      *   * *    * *       *** *** ** * **  *

Human           CAACTACAGCACAACCCAAATTATTACGATCTACTCGTACACGCGCCATACGACATCCAT 19793
Gallid          GGACTCGAATTTAAGG-AGATCCTCGTAAGGTG-TAATATAAGGGC-ACCTAAAACAGTT 22852
                  ***  *    **   * **  *    *  *  *  ** * * ** *    * *    *

Human           TTCTATCCAAGCCGATGTCACA--------TAGTAATTCTACCTATCCGATATTTCACGA 19845
Gallid          TCTAATCGAAAGCGTTACCGAACTTGTCTTTAATGAGAATCCCTATGAGAAAGCGCTTGA 22912
                *   *** **  ** *  *  *        ** * *   * *****  ** *   *  **

Human           GGGGTGACAAGCAAATCCTCATATCCGGCT-ATCAGAACGAAGGTTTCTTCGAAACC-CA 19903
Gallid          ATGTGG--AAGTAGACATAAAAACTTGTTTCGGCAGATCGTAAGCATTGCCGAACTCACA 22970
                  *  *  *** * *     * *   *  *   **** ** * *  *   ****  * **

Human           GGTGATGCTGTGGGCTCC---TGGAACACCGTTGCATATTACGCTGC-GTTCATTCTCTC 19959
Gallid          TTTAATAAGGCGAGGCTCGTGTGAAGAACCCTAGCAAGGGCAGTTACAGAACATGCTCGC 23030
                  * **   * * *   *   ** *  *** * ***      * * * *  *** *** *

Human           CAAATCTGATCCTGCCCCA-AAGCACACCTATCGCAACCCTGTTTTATGTAGAAAGAATG 20018
Gallid          CGAGCACCACCCCCCTTGTGGAAGTAGACATAA---AAACTTGTTTCG-GCAGATCGTAAG 23086
                * *     * **        *** * ** **    ** * *****   * ***  * * *

Human           ACAAGCCAGA---ACACTGAACAAAAAGACGTAATCGCAAAGCTGTCTGAGAATGGACAT 20075
Gallid          CATTGCCGAACTCACATTTAATAAGGCGAGGCTCGTGTGAAGAACCCTAGCAAGGGCAGT 23146
                    ***  *   *** * ** **   ** *     * ***     **   ** **   *

Human           TTTATTGGCAACCTGAAGCTCCCCAGAGAAAATTTTTTACATCACGACGCGATCACTGAC 20135
Gallid          TACAGAACATGCTCGCCGAGCACCACCCC---CTTATTCCTCCATAGCACTTTCATCGCA 23203
                *  *       *  *  *  * ***        ** ** *  **   * *  ***  *

Human           TTGTCTTTGGCAGCTATACCCAAGGACTCAGCGACTCCCGGCCCAGGGACGGTTTCTTCA 20195
Gallid          TTTTTTTTAAAA--TAT---TAACGTCTCAATCTATATAGGAAGTAATACCGAACTCCAA 23258
                ** * ***   *  ***    ** * ****     *   **       ** *       *

Human           TCAGTTTCCCCGTCTTGACGTATCCTCAGAACCAT-----CTTTTCAATTACATCGTCTT 20250
Gallid          CTACATTTAAAATCGACATACTTTCTCCCACACATACATATTTTTCCATGTAATCAACAT 23318
                  *  **     **   *    * ***  *  ***      ***** **   ***  * *

Human           TTTCTTCAGGTGTCTCAAC-CTTCATTAATTGCGTGGGAATA----AGAGTTTCATCCTG 20305
Gallid          TCGCAGAATAAACCTTCCCATTTTAATGATCGCGGTCCTATATTGTGGAACTGTCCCCCC 23378
                *  *   *     **   *  ** * * ** ***     ***     **  *    **

Human           ACCGACTTTGAAATCTGAGGTTTCTCTCTCACCTGCCTTCACTTTTTGAAGAGCGAGATC 20365
Gallid          AACAAA--AAAAATATCGGATTATATCAGCCCATCCTTTCTACATTGCACGACCCAGCCG 23436
                * * *     **** *  * **       * * * * ***    **  * ** * **

Human           TATTTCCCGTTC-TCCGTTACCCGGGAGCTTCACGTCCA-----GCGCATCCTTAAATGA 20419
Gallid          CGTCGCTCATTCCTCCGATAAAAGACCATAACATGAGCAAATGAGACCATACAGAAACGA 23496
                  *  * * *** **** **   *       ** *  **     *  *** *  *** **

Human           CACA---GAATCTAAATCGCGAGA------CAGATCAGCATT--TGGCAGCCTAAA--CG 20466
Gallid          CACCCATGGGTCTGCCCAGCAGGCTCATCCCGGGTCGATGTTGACGGTGGTCTGCGGTCC 23556
                ***    *  ***     **  *       * * **    **   **  * **     *

Human           GGGACTGCACAGA---ATCGA--GATCGTTTTGCAACTTAAAATTTT--TAAACAGATT- 20518
Gallid          CGGATCGTCGGGACTGACCGACGAACCGCTTCATCATCAAATATCGCCGTAAATAAAAAA 23616
                 ***  *    **   * ***   * ** **    *   ** **     **** * *

Human           TTGAGGATCACTAACTTCAAGAATTCTTTCTACGTTAGCTTCC----TCCGATTCGCGTC 20574
Gallid          TCGCCGTTCTATTAATT-GAGATCCTTTTTTTTATTTGCTTATCGAATGCTAGTAATTTT 23675
                * *  * **  * * **  ***    *** *   ** ****      * * * *    *

Human           CCAACTTGTTTTCTGTAGGCA--ACTTAAGCTCGGTCCCAGACTGA-----------TTG 20621
Gallid          ATTACTTATTTGATGAAGGGAGAAATTCAGCTCGTTCGTCGCCTAGCGTAGCGTTCCTTA 23735
                   **** ***  ** *** *  * ** ****** **   * **             **

Human           TGCAAAATGTCTTGAATGCCCTTCTCGCCTAAACTAATCAGATTTGTAAATTCGGCTAGA 20681
Gallid          CAGGAAATATATCGGGGATCGGCCGTGCCATTCTGAGAGAGCATCGCGAAGAGAGAAGGA 23795
```

```
                    **** * * *     *   *  ***      *   **  * *  **    *   **

Human           ATCTTGTTAACTCGCTGCCCAAGACCCGCTTGAACATACTGTTCCTCTCCGGCATTTCTA 20741
Gallid          ACCTCGC-AACCGCCGCTCTTTTATACACAAGAGCCGAGCCGCCCCCACATGTACCCCCA 23854
                * ** *  ***   *   *    *  * *  ** *  *     ** * *  * *   * *

Human           GCAGCTTCCATTAAATTAGGTAATGGCTCTTGCTCGTTTTGCGTCATTTCTTTATCGACA 20801
Gallid          ACACTCAAGTGCGAATTTGG-GGCGGTACATG-TCACGTGATAACATATCGCCATATCCG 23912
                 **          **** **    **  * ** **   *     *** **   **   *

Human           TTAAACTTTCCTGAGGTTTCCTTTTTTTCTGCATTGCCGC--CAGAATACAAGTCCCTGC 20859
Gallid          ATTGGCTCACCTCGGCGTTCGCACCAGAGTCCAATAATATAATATAATATAATATATTAT 23972
                 *   **  ***  *  ***         * ** *        * **** **      *

Human           TATTTCCTTTCCCCCCTCTTGATACGTCACGAGCATATTTCTTATTTAACCCGGCA--AA 20917
Gallid          TGGTTCGCAGTGCGAACGCTGACGCGTT-----CGCACTGCTCATTTGCATACACATCAC 24027
                *  ***      *      ***  ***      *  * * ** ****       **  *

Human           AATATCATTCGCGTGCTTAGTTTTTCTCAACACTGGGGAATCCCGAGCAGTCACAGTTTC 20977
Gallid          GTGATAGTTCGAGTAGGCGGTACGCCCACCCGTATAAGAATCGTAATTTCTTGTGGCCTC 24087
                   **  **** **     **    *    *      *****   *    *    *  **

Human           -AGTCGCATCGCCAGAGTCTCCGGTCACTTCGCGTGAGTGTACTATAGCCTTATCATTTT 21036
Gallid          GAGTGGCGGTGCGACTTGCTCTCGTCGGA-CGGGAGGCGGCGGTATAGGATAAGAGATCA 24146
                 *** **   ** *    ***  ***    ** * *   *   *****  * *    *

Human           TTTCC---TTAGTCCAGCTTCCAA--GCAG-ATCTAACTTCTTCTGTT---TCCTAAAAC 21087
Gallid          CAAAAAAGCGAGACCTGGATCGAACGGCAACGTCTCGTCCCGGTTGTTAATCGTTTGGAA 24206
                          ** ** *  ** **  ***   ***     *   ****      *   *

Human           CGTCTTTGTCCC-TCAAATCTTTAATGAGAGAAACGTTATGGG-------GCGACACTCC 21139
Gallid          CTTCTTCGCCTGATCGGTGGTGTAACCGTGTAGGTATTTTTAGTTTTTATGTACCATTTC 24266
                * **** * *   **     * ***      *    ** *  *       *   ** * *

Human           GGTCTCAGTACTGTGCGTCGTAAGTTTCGCCTCTGATCCAGCCACA-TCGGAATTATTCA 21198
Gallid          GGTTGCTTTATTATATTTCCCACCCATCGTTTTTCTTTATAACATAGTTTGACCCTCTCG 24326
                ***  *  ** * *   **  *    ***  * *  *     ** * *  **     **

Human           ACACGAAGGGTG----TCCCTGTCTGCTCAGAAACCA-AACGATGCACCGGATCGAAAAC 21253
Gallid          GCATCACAGATGGCACCCTCCCTCCACTCATGACCCACAACCGCG-ATTTGTTTTTCATC 24385
                 **  *  * **     * *  **  ****  * *** ***   * *   * *    * *

Human           GTCTGCATTC-GCCGCTGTGTTTTTACTCCACGGCCGTGAAACATCCGATTTATTCTGAC 21312
Gallid          TTCAACGCACAGCCATCCTTGTCTTTCTGCCCG--CACCGCACGCTTTGCTCGTCCCCGC 24443
                 **  *   * ***    *  * ** ** * **  *     **       *  * *   *

Human           GC---AGATTGAGATTAG-CGAGACCATTTACCACATCAGTAATACCGCTACTGGGACTT 21368
Gallid          GTGCAAGATCGGCAGGGGGTGATGGAATTCGAAGCAGAACACGAAGGGCTGACGGCGTCT 24503
                *    **** *  *   *  **    ***     **  *     *  ***   **    *

Human           CTGATCGAAGTTTTATCCTGTAGTAAATCCACGTC------TCCCGTATTAGATTGCGCC 21422
Gallid          TGGGTCGCCCCCGCTCCCCAGGGTGGAAAAGGGGCGGAGGGCCGCGCAGGGGTCGCCGAC 24563
                  * ***         **    **  *     * *       * ** *   *    ** *

Human           TCAGCTGGAAGTTTAACACCAAAATCTAAAATCCCCGAAGG----GCCAATTCTATAATT 21478
Gallid          GAGGCAGGGCATGGGAAAACAGAAGCGGAATGCGCCGAGGACGGCGAGAAATGCGGGGAC 24623
                   ** **   *   * * ** ** *  **  * **** *     *  ** *

Human           ACCGTGCGACTTATCTCG---CAGATCCAGATTGGTAGAAGTCAAAGACGACAACGGCTC 21535
Gallid          GCCGAGATGAGCGCTTTGGATCGGGTCCAGAGGGACCATGGGGATAAGCATACAGAGAGT 24683
                 *** *         * *   * * ******  *      *  * *  *    *  *

Human           AACTCCGGACATTAAAGAGACAGACGAAACAGGCGTTGTCGCTCTAACGTCTCTACCCCC 21595
Gallid          ACTTCCCGAAAACGCATAG--AAGCGAAATATATGGATTTACT-TGTCGAAGCTGAACGG 24740
                *  *** ** *    * **  *   ***** *  *   *  ** *  **   **   *

Human           GAGTGTTTCTTTCTTTATCAAAATT------TTTCCCCCGGCATTATCG--ACGCTATCC 21647
Gallid          GAAAATAAAAATCTTCGAAAAAAATATAATATCATACTTGACGTTAGCACAAAAATATTA 24800
```

```
                  **   *     ****    **** *      *    *  * * *** *   *   ***

Human             GACTCTGGAA-ATTTATGTCGCCT----CTG---CGTTTTTAAGGGCGCGTCCAAACTTT 21699
Gallid            GTGTTCGGAACATGTATAATGTTTGCAACTGGAACGTTGATTGGGAAAGGTACAAAGATC 24860
                  *  *  **** ** ***   *  *    ***   ****  *  **    ** ****  *

Human             TGGGTTGACTTATTTTCAGACGAGCCAAATCCGCAGATGGTCTCTTT----GTATCTATA 21755
Gallid            CAAGTT--CCTGTTTGCA-ACAGCACTAATCTAGCTCTGGCCTTTCTTGCGGGATTTCTG 24917
                     ***  * * *** ** **    * ****      *** ** * *    * ** * *

Human             GATATACTAGGTAAACTCAAATCTGAAGGCATGCGGCCGTCTAAAGAGGCGAAATCCATT 21815
Gallid            ACTCGACATGTTTGATTTATCATTGAGTAATTATAGTTGCGTCTATTCACCAT-TGTATT 24976
                    *  **   * *  * * *    ***     *   *  *  *  *    * *  *  ***

Human             AAAGAGTGAGGCCCATTCTCAGGCACCGAAAATGCTTTCGACGCGCTGTATGATTCCAGA 21875
Gallid            AGGTAGTCGTGGTT-TGCCACGACATTAACTATACATT-GTTTAGTAGAACAAACGCAGG 25034
                  *   ***   *    * *   * **   *  ** * ** *    *  * *  *   ***

Human             CTACTTTT-GATAATCTCATAATCACTGGTGATTAT----TTTCAAAAAACTCTCTACTC 21930
Gallid            AAAGGTGCAGAAAACCTTATCATGCCGTGAAATTGTGTGCTTGTTGTGATGTTTCTGCTG 25094
                    *  *   ** ** ** ** **  *  *  *** *    **      *  * *** **

Human             TCTGACCAGTTAGAGGGGTCTGCAAAACCCTCTGAGAATACACAGCATTAAAATTCTCGT 21990
Gallid            TTATATAATTTTTAGGGAA-TATGAAACCGTGTCAGTCTACTCCTATGATACAGAATTGA 25153
                  *   *  * **  ****   *   ***** * * **  *** *       * *   * *

Human             TCTTCCTCAAATGAC-CCGAGAGATGATTTTGCGTGGTTACAATTTGAGACATAGCAACC 22049
Gallid            TCGTCTATGTACAATGCCGAGTAAAAGTATTGCG-GATCATCATGCTGGTTACGGGG--- 25209
                  ** **     *  *  *****  *   * ***** * * *  **     *  *  *

Human             TTTTCTAGCTTTGACCAGGACTGGTTTAACGTTACAAATAACTTACCAAGATAAACTAAT 22109
Gallid            -----TAGCGCT---CGCAATTG---TAGCTTTATTGTTGATCCATGGAACTGCATTGGT 25258
                       ****  *   *   * **   ** * ***    * *   *   *  *  * *  *

Human             T-TATTAATACTCGTCACTAAGTAACCGTTTTCGTCCCAGGGGTCATTTATGTT-ATAGA 22167
Gallid            TATGGTGAGTGTTTTCATGTAGTGATTGGTTTCTATATGTTATTAATTTTTATTTACAAA 25318
                  * *  * *   *  ***   *** *  * ****          * **** * ** * * *

Human             CGGTGTACCAGAGCATGTTTT----TTTCAGCGTCTTGCAAATTATTTCTATCTCGCTG 22223
Gallid            CGCTTTCCTATTAGATATTTTCAGATATTAAGGTCGGATCGCAACCTCAAGATCTAAAAG 25378
                  ** * * * *    ** ****    * * *  ***           *    ****    *

Human             -CTGTCTTCTGCAGTAAGATTTACATAAGGAGTATCGATCGGAACATTGCTACCCCACCT 22282
Gallid            ACCAGCTTTCATATCCGAGTAATTATGACGCATATACCTCGCA-TATTTCCGGAGCCGAT 25437
                   *   ***    *      *    ** * *  ***    *** *  *** *     *   *

Human             AATAGAATTTCCTAAACGCAGTAAAGCGCGATGAATATCACTGAATTGCGATTCAA---- 22338
Gallid            CATGGACATTCAGAAATGTGGACTGATATCAATCCTCCATCTAGGAAACCACTCCATGGC 25497
                   ** **  ***  *** *  *         *    *    **      * * ** *

Human             ACTTTTCTTTGTTCGTGAACATATTTTCAAAAT-TTCCTTTATTCAGACCTTTGGATTTT 22397
Gallid            GATTCCCTTGATATAAAAACAAATGAAGAACATATTACTTTATCATCGCCCAGAACATCT 25557
                    **  ***  *     **** **    ** ** ** ******     **       * *

Human             AAGTACTCGTTTACGACATTCTGGAGTTTCGTGATGTCTTGCCATATAGCATTATAATCC 22457
Gallid            ACAAAAACGACAAACGAGAATGGGCATGAGAAGGATTCAAAGGATATTAAGTTTTCATTT 25617
                  *   *  **   *         **  *     *   **     ****    ** * **

Human             GGCTTCCGTTTAGATAACACGTAG-TGATACATAAGCCATAAAATAAATGTGTTATACAG 22516
Gallid            --TCTACGAATAGACACCCCATTTCTGTTTCACCTACTACTGATATTGTTTCTATTGCAG 25675
                      * **  **** * * * *   ** * **    * *   *     * * *  * ***

Human             TGTTTTTAACTCGTCTACATCTTCTCCCTCTCTCACGA--CACAAGAATTAGTCACATAG 22574
Gallid            TTTCGCACAATAATCCAATGGGTGGGGATACATGGCAAGTTTCAAGGAATGGTCCAAACA 25735
                  * *     * *  ** *     *     *   *  * *    **** * * ***  *

Human             GGATGTTCTCGTAAATCTACATTCTCCAGATTTTCCAAATTGCTA-AGAAAATTTGTAAG 22633
Gallid            CAGTTCATGTACCAATCTATGGGAAACCCATTTTACGATTAAATGGAGAAGAATTATCT- 25794
```

```
                       *         ******        *  ***** * * *    *  **** * ** *

Human           ACGCTGAACTTTGTCTCGATCTAGCCACGCAAAC----GGGATCGACTGCG-CTTTCAAA 22688
Gallid          ACTCAGATTGCTCATATGAGTATGTGGGATGAACTTGCAGGGTCATTTAAAACGTTTGCA 25854
                ** * **    *     **    *       ***     ** **   *    * **   *

Human           TCCATCTTTTAGAAAACAAACTCAAAAACAAAACTGCTCGTCTTCGCCAAGACTCAATGT 22748
Gallid          GCGAC--TGCAGAAAGCTTCCATATGATAACTACAACCCATTTGTTTAATAAAACGTTGC 25912
                 * *   *  ***** *   *   *   *   *  **  * * * *      *  *  * **

Human           TTAATAGTCCTTTTTTTACC-CACACAGAAAGC------TGACAAGCGACGAGATGGACA 22801
Gallid          ATAAAGACGTTTTTTTCGTCGTCCATGGATGGCATGGAATTACAAATGAC-ACTCATATA 25971
                 ***      ******   *   **  **  **       * ****  *** *      * *

Human           CTGTCATTGAGCTGTCCAAACTCCTGGTAAGTCTC---CAGAGATCATTGTTACCGAATA 22858
Gallid          TTTTTATCT-GCTGTA-AGGCTTCTGACGCGTATGATGCCAACATCATGTATAATCTACC 26029
                 * * **   *****  *  ** ***    ** *    *  * *****   **     *

Human           TTTTATTG------TAGCGTACCATGACAATAACATTCAATATCTT--TAATAGCACGAT 22910
Gallid          TTTCATGGGAATCCCAGGGTGCAATTGGAACAGCTGCCGATGCCATCCTGTTGGCCCGGC 26089
                *** ** *        ** ** * **   ** * *   * **  * *  *  * ** **

Human           GAAGAGTTCAAAGACAACGCTT-CCTGTACCTCGACCCCCACGCTAAAGACA--GCACGA 22967
Gallid          GGGTAAACATTACACAGTTCTTGTCTGCCATGCCATCTCAACTGCGAATCCATTGTATGG 26149
                *   *      * ***   ***  ***      * * * * **    **  **  * * *

Human           ATCATTGAGAGCGCAGTTACCGGAATCACGCTCACTGCATCCGTACCCATGATCAT-TAT 23026
Gallid          GCCATTCCTTGGGATCATATGTCTGCGGTTCTATTTGCAGACAATATCATAGTCTTATGT 26209
                  ****    * *    **           **   ****  *     ***  ** * * *

Human           AGTGATTACCACCATGATTCTCTATCACAGAGTCGCA--AAACATAACGCT----ACTTC 23080
Gallid          CGGGAATATGTAAAGGTATATTAGGTATAAATCCGTATGAAGTGTTATTCTCAGCACCTG 26269
                 * ** **     * *  * *      * * *  ** *  **   * *  **    ** *

Human           ATTT-TACGTCATCACACTCTTTGCTAGCGATTTTGTGCTCATGTGGTGTGTCTTTTTTA 23139
Gallid          ATTTATATGCACGCATGCATGTAGATACCATACGCTTGGACGCGGAATATGTTGCAATTT 26329
                **** ** *    **  *   * * ** *        **  *  *   * ***     **

Human           TGACAGTGAACAGAGAGCAGCTGTTCTCATTTAACCGTTTCTTCTGCCAGTTAGTTTACT 23199
Gallid          TTGCTACGACTTCGCAATATTTGAGTACAT----CCGATTCAGATGCCGATGAATATATA 26385
                *  *   **      *  *  **    ***    *** ***   ****  * * * * **

Human           TCA-TCTATCATGCAGTCTGTTCATATAGCATAAGCATGC-TAGCTATAATCGCAACAAT 23257
Gallid          ATAGTCAATGATGCAGTTTTTATGAATAGCGTATGTGCAAATCCCTATGAATGGAATATC 26445
                  * ** ** ******* * *    ***** ** *      *  **** *  * ** *

Human           TAGGTACAAAACTTTGCACCGGCGTAAGCAGACCGAAAGTAAAACATATAGCACGGGGCG 23317
Gallid          CACTTATG------TACTACAGGTTATGGGGAACTAAAATCATGTGAGAGATTTGGGGCT 26499
                 *  **         * *  * *  ** *  ** * *** * *            *****

Human           AAACATAGGAATTCTCCTACTAGCTTCCTCAATGTGCGCGATTCCCACCGCTCTCTTTG- 23376
Gallid          GCCAACGTAACCACTAGTCCTGGAGTAGTAGAAG---ATGGTTCCCAAATATGTCTGCGA 26556
                    *    *   **  * ** *  *  *  * *     * ******    * ***  *

Human           ----TGCAGATTAACGGAGCAAAAAAAAACGACGGGAAAATGCGTCGTTTATCTATCCTCT 23432
Gallid          ATGCTTCCAATAATCGCAGTACTGCAATCACTAGATCTAAAATCCTCATACCCTTTGTTA 26616
                    * *  ** * ** ** *    ** *    *    *     *   ** *  *  *

Human           CCGAAAGCTTA-CGAACTGTTTCTAGCGGTGAAAATTGTTTTCAGCTTCATTTGGGGAGT 23491
Gallid          CGTATTGCCCCTCCAGATGCGTCCAATGAAGTAACGCATTTGCCA-TCAATTTGGAATAT 26675
                *  *  **    * *  **  ** *  *  * **    *** *   *  ******    *

Human           CCTCCCAACCA-TGGTGTTCAGCTTCTTCTACTTTATCTTTTGCAAAGCTTTGCACGGCG 23550
Gallid          TTACGTAGTAGGTAAAGATTA--TAGATACTCTACATATGCAAAAAACGATAGCTTATGG 26733
                   *  *     *   * * *  *   *   **  ** *     ***   * **     *

Human           TCACCAAG---AAAAAACACAAAAAGACTCTATTTTTTATTAGTATCCTTCTCCTGTCAT 23607
Gallid          TATTCGAGTGCAATATGCGCAGGAGATACAGGGTTTTCTATACCATCAGTGTT-TACTGT 26792
```

```
                *   * **   ** *  * **  *         ****   **  ***  * *  *    *

Human           TTTTACTCATACAGATCCCCTATATAGCTATCCTTATATC-TGAAATCGCATTTCTCTAC 23666
Gallid          ATTCGCTCCTCCGAGCATCTCTCTTAGGGTACGAGCTGCCGTACAACAGAGCAGCACTAT 26852
                 **  *** * *      *     ***    *    *  * *  **  *     * ***

Human           ATGCCACAGAACACCTGCTTTTGGTTAGCCCGTGCAGAGATATTGCAGTTAATCATTCGA 23726
Gallid          TTATAAGAACATAACAATTTATTCCGCATTTTTA-AAAAATACGTCCCGTTATTATCCCA 26911
                 *   * *  * * *   ** *          *  * * ***   *   * ** ** * *

Human           TTAATGCCACAGGTACATTGTTTCTCTAATCCGTTAGTCTACGCATTCACTGGAGGCGAA 23786
Gallid          CCGTTACGCTCGAAACATTAGGGC-CAATTCTATCAGCGTACGCTTGGA--GAGGGCGCA 26968
                    * *    *  *****    * * * **  * **  ***** *  *  *  **** *

Human           CTAAGAAACCGATTTACCACCTGTTTCCAATGTCCATTTTTTCCCAAAAGACTATGTAGT 23846
Gallid          TGCATAATAGTAGTTATTATCCGCTCCC------TCTTCCGGAACAGGAAATAATGGAAT 27022
                   * **    * ***  * * * * **        **      **  * *  *** * *

Human           ACACAGAATCGCAAACAG-TCAGACGTCTCCGAACAT-------GATCAGAACT-CGCCA 23897
Gallid          ATAAGTGCACGCATATAGATCGAACCTACACCTGCATTCCTACCGATCTTATTTATGCAA 27082
                * *      **** * ** **  ** *   *   ***       ****  *  *  ** *

Human           TCAGAATC----GTCAGTGGATGAAAACGAACCCCCTTAAACACGTTTTCCAACTTAATA 23953
Gallid          CTACGGTCTGGCGTCAGATATTTCAATGGGACATATTTTTCCCGTGTATCCATCCAACA 27142
                  *   **    *****    *   ** *   *   **   * *** *  * *   ** *

Human           AATGCTACCCACATAAAATCACGTGCTAGCTCTGTTTTCTT-TGTTTTGTCTTCCATA-- 24010
Gallid          ATTGCCTGCCATATCGCCCCAC-TAATGCCATTATTTGGAGACGCCCTGTATTAGAAATA 27201
                * ***   *** **     *** *  *  *  * ***      *    *** **  * *

Human           -GAGCATGGCGCACGCTAAAAAG------CGGGCACGACGAA-AACTTTTAACTTCGACG 24062
Gallid          GGAGTGTGGAATAACATAACAGCATCATTTCAACGTAACAAACAACTGATGGCTTTAACA 27261
                 *** ***   *   *** *            *   ** ** ****  *  ***  **

Human           GACGATCCCATATTGTCCAGC-ACTTTTACCATGCGCCCGACAAGTAAGATTGCAGACGC 24121
Gallid          TTCGAAAAT-CATAATACAGCCACAAATACGACCTTGTTAACGTTTCATGATGTATGTCG 27320
                  ***      **  * **** **   *** *        **   * *   ** *

Human           TGAAATCATTTCAAGAGAACATGATTATATCGCAAGCAAAACACAGGCGGATTCAAAAAA 24181
Gallid          TGAAAGCAAAATCAGATCGGTTGTCTACTTCGAGTATGATTGGCTTGAGACTTCGATGAA 27380
                ***** **     ***     **  **  ***      *    *  * *  *** *  **

Human           AAAATTATCGTCCCTGTCTGTGATTT--------TTGACAAAACTGTCCTGTTTGAATT- 24232
Gallid          CA--TTACCGTCCTGATACCTGGTCTATACACGCTTAGGTGGTTTTTCCCATTTGAGATA 27438
                 *  *** *****   *   ** * *        **        * ***  *****  *

Human           -TTACGGTATAGGAGACAACAACGAAAAAGCTATCGTCTACCCTATCGATCCAGATTTCT 24291
Gallid          ATTGAGATGCCAGTAAGAGTATCTTATAATAATTCATTTGCTCAAGCTCTCACAACTTCG 27498
                 **  * *    *  * *  * *  * **    ** * * * * * * *  **   * ***

Human           ----TATTATGTGATTCGGAAAATAATTGCACCCTATCACCTTTCTTATAAAATTTTTTT 24347
Gallid          CGGGTATGAATCGATTCGATAATAAAGTATGACAAATCACGTCAGT-GTCGGTTTTATTT 27557
                    *** *   ****** **  ** *    *  ***** *   *  *    *** ***

Human           CTCCTGATCTCACGTCACAGTTTTTTTTTCATATGTCTCGCGT-TGATACATCCCGTATG 24406
Gallid          ACTATGATCAAA-ACTGCAATATCATCTTCGTATTCGTCACTTGCGAAATGGCCTGGTAA 27616
                    *****  *     ** * *  * *** ***   ** * *  ** *   ** *

Human           AAATATTTAAGGTGGTTGAAATCA-CACCAATGATCACACACGCGATGGAAGGGTCGAAG 24465
Gallid          TTATAACATTGGGGAATGTATTTAACAGCACTGAAAATGCA---ATTTGAGGAGTGAGAG 27673
                  ***     ** *  ** * * * ** ** ***  *  **     * ** * **   **

Human           ACATTCAACATTCCCACTTTCGTCTTAGACGAAAATTGTAACTTCATACCCGATGTTCTC 24525
Gallid          ATTGTGAGAAATGTCAACTCTGAGAAAGACGCATATAATACATTAATTTTGGGTCGACGG 27733
                *   * *  * *  **  *  *    ***** * **  **  ** **    * *   *

Human           TCACGCGCCAACGCTAAATTTATCAAAGAGGTACTCATCCGAGACTCCTACA--ATGCGG 24583
Gallid          ATGGAAAATCGTGCTA---TCGGCAATGAGATACAAACTCTAGATAACGATGTTATGCAA 27790
```

```
                            ****   *   *** *** ***  *  * ***   * *    ****

Human           TTTGTCTTGCAAACAGCTTTAT---TCCCATGGCGACTCAAACCGTAGAGCAAATTCTGA 24640
Gallid          ACATAGTTGCGTTTAAACCTAGGAGTAATAATGCAAGTTAATAGACAGC-TGAAGTCCGG 27849
                      ****    *    **    *   *  ** * * **     **    ** ** *

Human           TTATTATCACAAAGTTTAAGTTCTCACGTTCACGAGATCTGCTCATGTCAGTATTTCGCC 24700
Gallid          CCGGCCTTATACAATACAGATTTTTTCTCATATGGAATGAGCATGTAATATCGACTCACA 27909
                      * * * * *  *  ** *  *     * *  **  **    *   *     ** *

Human           TCG-GCGTACATATAAACAGATTTTATGCCGGAAAAAATCAAGTCAAACA---TATGATC 24756
Gallid          ACGTATATTCCCTTAAAAGGAGTATTCCTTAAAAGGAATAATCTCGCACAATCTATGCAG 27969
                 **    * *   ****  ** * *       **  *** *  **  ***   ****

Human           ACAATGATGA---AAAGTCTTTTCGATACCGAAGAAGCCATGCGACAACTCGACAGAGCT 24813
Gallid          AAACAGATAATCTAGGGTGTGGGCGGTGCTTTGTACTTCCTACGTCGGATCAGTTATAAA 28029
                * *  *** *   *  ** *   ** * *      *   * * ** *   **

Human           CTTATGGGGCTGTTTGTCGAT-GCTCGTGATAATTCATATATGCCT---CTCATAGCATT 24869
Gallid          AACGAGAGGTTGGTAACAAACAGCTTTTGAAAAATGACTAGCGAGAGAGCTCTTACTCTC 28089
                     * ** ** *      *  ***  *** ** * *      *      *** **    *

Human           GTCCCTGC----------ACGAAAACGGCTTAC----CGGATTCTAAATTC----ATAAA 24911
Gallid          GCGCCTGGTAAAGTTTCGACGGCAGATATTTATGAAGCCGATTTCAGTTTCCGTCGTGAA 28149
                *  ****            ***  *      ***      * ****  *  ***      * **

Human           AGC---CGTCAGATTAATACAAACAACCGTAAACTCCTTTCATAATCGACCGGACGCAGA 24968
Gallid          TTTGTACGCCAAATTT-TACAACGTGAGTTTATATTGTTTGAAATTTCAATGTATATTAT 28208
                      ** ** ***  *****         * *  *  *** * * *  *  * *

Human           ---TATCG-AACAGTATGCAGAAAAAATTACGAGCATACAATT----ACCTCTATAAAATA 25020
Gallid          CTTTATCGTGACATTCTGTTATAAAATTATACTTTTTTTATTTAGGATTATTCCCAAGGA 28268
                   *****  *** * **    *******       *   ***     *    *   **  *

Human           CCCAAGTATACTCTGAAAGAAGCTGTCGACATTTATTCCGATAATCT--TAAAGATCTCA 25078
Gallid          CCTTGAC-CACCGTCAAAGAAATAGAGGGCGCAAAATGTCAGCGTCCGCCTAAGGTGGAA 28327
                **       **  * ******   *  * *    * *   *   **     *** *   *

Human           CTATTGGAGTTAATA-----AAAAGCCCACCTTACTGTTT-ACGTCTTCAGATGACGCAT 25132
Gallid          TTGTCGACACTTCTACGTATAATAGCCCACCATGAAGCCCCACACATTTATGCTCCAGGT 28387
                 * * *    *  **     ** ******** *    *     **    ** *      *   *

Human           ATTTATCTCACA---TTTACAACGACTT-------ACTATTTTTAACTTCTA-----CTT 25177
Gallid          CCCCAGCTCCCGAAGTCCACAGCAATCGTTGATGGGTTATGGGTAGCTTGTCGAGGTCTT 28447
                    * *** *    *  *** * *           ***   ** *** *      ***

Human           GGAATATGATTTATAATTGC--AAAAAAGAGATAAGGCGTTTG-AATACTTGGATCAAAT 25234
Gallid          GCTGCTGAATGCATGTTTACCGGAGGAGGAACTATTGCATTAATAGAACGTCTAGCGACT 28507
                *       **  **  ** *    *  * **  **  ** **    *  ** *  * * * *

Human           ACGAAATCAACT-CCATAATGGAAACTGCTGTATTGGTAGGAT-TCCAACTTCCAGATCT 25292
Gallid          TCCTGGCTAACGGCCATAAGATTGATT-TTATCCTGGCATCCCATTCACGCTCCCAATCG 28566
                 *       ***  ******     * *  * *  *** *      * **   ***  ***

Human           GAAGGAAACAATTCTAGAC--CTAGCGGCACTCATATCAAATATGAATCTCGTCAGCCCC 25350
Gallid          TAATCAAGAGCCTCTCGATAGCTTATGCCGCGAAGGTCGCGAATATATTGTAATGATTTC 28626
                 **  **      *** **   **    * * *  *  **     **  **            *

Human           GATAAAGA---ACTTTTCCCTCACTACAAACTCATATTAGCAAAATTGTTTGAAATCTGT 25407
Gallid          TGGGACGGTTCACCCACGACATGCTACATGGCCATTTTGGCAAGTGATGCGGAAATGTTT 28686
                    * *    **      *    *****    *** ** ****         ***** * *

Human           ATTTT--TGCAAC-TAAGGCAAACATTTGCATTTTACCATCCTTCAT--AAAGGGTCACC 25462
Gallid          AGATTGGTGCTGCGCATTTCACCCACCTGATGACCACAGTTGTGAATTCGGAGCACCACG 28746
                *  **  ***  *  *   **  **  **      **  *  *  **    **   ***

Human           TAATAGAGTTCGAGGATGTCTTAAAAAGAAGCAATGATGACGAAGACCTCAACTATCTGC 25522
Gallid          CATTGGAATCCGGCTAGAGGGCGAAAATCATTTTTTCGCACCAATTTTGGGATTGTATTC 28806
```

```
                 * * ** * **   *       ****  *    *    ** **       * * * * *

Human           TTCTGAAATCTCG-AGATTCTGATGACGAATACGATGAAGACAAACCCCCAATACAAGTC 25581
Gallid          TGTAGTAATGACATGGAGTCCAATTTC--ATGTTACCGAGA---ATTCCCAATACGTCAA 28861
                *   * ***  *   ** **  **  *  **   *   ***   *  ********

Human           GATCC-AGGCAGAGTAGACAACGTGCTTACAGATTCTGACTTTTTTAACGT-AACCCCGG 25639
Gallid          AATTCTAAGGAGCCTGATCCCCAACCATCAACATCATCCGAACCTGAGCCTCAGCCATCG 28921
                 ** * * * **  *   *  *   * *  * **  *       * * * * * **   *

Human           AAAATGCTTTCTC-------TTCTATAGCGATCATGCCAATTTCTTATGATAAGACGATA 25692
Gallid          ACATCGTCTCATCGTAACATTCCCGTAGCACGTGTAA-GACCTCTTGTCGCTCAGCAAAA 28980
                * *  *  *  **       * *  ****     *    *  **** *       * * *

Human           GATGTGGAAGACAATGAAATTCAAGTCCTAGAGGTTGAGATGCAGAGCCTCTCAGCAGTT 25752
Gallid          GGTACCCAAAAC--TAGACCTTTGGATACAGAAATACACAGGCCCGGACCAATTGCAATC 29038
                * *    ** **  *  *  *   *    ***  *  * * **   * *     *** *

Human           GTCTACGGAGCCGTTGCAAGCAAATACGGCTTAAGCCTGGAGC-AAGTTATCAGAAAGTT 25811
Gallid          CAGAACCCAACAGATACGGATGAACCTGAACTTCGCTTGAACCCGAGACCACGCCCGGGT 29098
                    **  * * * * *     **   *   *  ** ** * *  **    *     * *

Human           AAATCAAAATGAAGGACGGACCTCTTCTCGTGCATCCCCCAGCCACAGTACTTCTACCGT 25871
Gallid          CCTTCGGGGCAAAATACACGTCCAAGGACTCCCA-CTTTGGACTTGGATACCGTTGTCGT 29157
                   **      **  **    *      *   ** *      *     ***   *  ***

Human           CCCATACTCCCCCCCGCAAAGCGATCGCTCCACTCCAACTTCTATTCTAAGACAAAGAGT 25931
Gallid          TCGAGAT--CACCCAGTAACAC-ATCGTCGTCCGCGTTCTCCTAGCCCAC--CTGAGGAA 29212
                 * * *   * *** * **  * ****     * *   ** ***  * *   *  **

Human           ACCAATACGTTCCAACA-GCAGATCA-TCATCGGTTTCTTTCTCACAGGAAGACAGCAAT 25989
Gallid          GATTATACTAACCAAGATGAAAATCTCTCATATACTCCCCAATTAATCCATTCTTCCCCA 29272
                    ****   **** * * * ***  ****    * *    * *    *      *

Human           CGTAGTCATTACTCTGACGAAACAAATATTAGTGATTATTCATATCCCATGGCAGATCTA 26049
Gallid          GATAGTGAAGTTGCTGAGGAGATTTATGCTCAGCCCGACCCTTGGGGTACACAAGAACTG 29332
                  **** *     **** ** *   **  *       * *  *     *    *** **

Human           GAATTAGAAGACGAAGAACCCATGGAGGACCATCCG-CACTCCCCTCAATCAACATCATC 26108
Gallid          CTATTAGCAAATCGTGAACGCACTCCAGATGATCAAACAGATATTACGGATGATAGCG-C 29391
                  ***** * *    **** **     **  ***   **       *     * * *  *

Human           TAACAACTCCATGTCTCGTCAAAGCCGAGCTCTACAAAACGGACAAAGAAGACGT-GCTC 26167
Gallid          AGACTGGTCTGAGGGCGAAACACGTCGACCATCACATAGTGAAGTTGGGGAACGTAGATT 29451
                  **   **   *        * * *** *   *** *  * *    *   **** * *

Human           CCACAATGGTCCCATCATCTCAGACACGAA-----GACAAAATAATGCCCGCCCCAGAC- 26221
Gallid          GTCCAGAGAAAATAACAGTGAAGATCCAAACCGTAGTCGGAGCCGGAGTCGATCTAGGGA 29511
                   **  *     * **    ***  * **     * *  *        **  * **

Human           GCGTAGCC--AGGCGCCTAACGGAAATGA-TGAACG---ACGCAAGGTTATAATCACAAA 26275
Gallid          GCGTAGGCGAAGACGGCCACGAGTTAGGCCTGGGCGTAGGAGTACGGCTACAACTATACG 29571
                ****** *  ** ** * *   *  * *  **  **     * * ** ** **  * *

Human           TAATTTTATTGACTGTGAAAAGT--GCTTTATCTGGCCAAT----TCATTGTACG----- 26324
Gallid          AGATCTTGTGGTTCTTGGGATGTCGAGTTCAGATGATGAATAGCATTTGTGTATATGTTG 29631
                  ** ** * *    **  * **    ** *  **   ***    *   ****

Human           -ATAATAAAAGTTCCATTGTTTGATTGATT--TCCGTATTCTTGAGCTTGTAGATAATTT 26381
Gallid          TGTGATTGCAAATCCACTGTATGGTTATATAGTCAGAATTTAATAAAATGTTCGAAGTTT 29691
                  * **   *  **** *** ** **   *  ** * ***    *   ***    * ***

Human           CCAATAGAACATCCCGCGTTAGATGTAACTCATCAACGTAAGAAAAAAAACGAATAGCAT 26441
Gallid          ACACCTTCACGTATTGTTATGGGTATTTATGGGTGGGCGAGGGCGTGTTACAAATATTAA 29751
                 **     ** *   *   * * * *   *         * *       ** ****  *

Human           TCT----GTTCTTCAT-CATCACCA-AGTCTAAGGTGACTCTTCTCTAAAAACTAAAAAA 26495
Gallid          TTTTAGAGCCTTTCACACATCCCAACAGTTTTAATCGGAGCTGCTCGGTAGCATGAAAAT 29811
```

```
                * *    *   ****  **** * * *** * *   *   ** ***   *   * ****

Human           AAAACACACCATCAATTTTATTCAACCAATCTAAGATAGTTTTGCATTAAACAATAAATT 26555
Gallid          TTATAGAGTACTCGTGCATCTTTCCTTCGTGCTAGGTATGTTTACTAAGACCAATACGGT 29871
                  *        **     * **       *   ** **  *** *    * *****   *

Human           ACTGCCAGAA--AAACTATAGCCTAGCAAGCTAGTGAATCAAA-TACCTAATTCCAATT- 26611
Gallid          TCTCGCATGGTCGAAATACGACCTGGTACACGGCTTTATGAGGGTAGCGAACATAAGCTC 29931
                 **  **      ** **   *** * *  *   *  ** *   ** * **    *  *

Human           --TAATATATCTTGAAATCTACCATAACACGTACAAAATCAAACTCACCGCATATATCAT 26669
Gallid          CATTATGCGGCTCGACTGCCTTCCGAACTTAT-TATCTTCGAACGCGGGGTATGCCGCAC 29990
                  * **    ** **   *   *  ***   *  *   ** *** *   *  **    **

Human           ATTAAGGAAAAGCTGTTTCTCTTGTC-TACTGGTTAACATGTACAACAACACAATTA--- 26725
Gallid          TTCCATCAGACGATATTCCCACGGGCATTTTTATTAAGGTCAACTGCAGCATACCCGAAT 30050
                 *  *  * * * * ** *    * * *  *  ****  *  **  ** ** *

Human           TCTTCCTGTAAAAGAAAAACACATCAAT--CGGCATACAGTAG-CTTGTCATT--CTCTC 26780
Gallid          TCATTCTATGGTATGAACAAAAAGCCATGGCAGCGTGGATCAATCCTATTATGGGCACTG 30110
                ** * ** *   *  ** * * * * **  * ** *  *  *  * * * **   * **

Human           TTCCTGTAACAAA----------ATAAACAATTTATGCAATCCAATTAAAGTCCCTTGTC 26830
Gallid          TTCTAATGATGAATGACGTTTTGAAAAGCGGATTGGAGAATTCTGTGAAGGTGGGACTTC 30170
                ***   * *  **          * ** *   **    *** *  * ** **      **

Human           TT-TGTATCGAAA---GAAAACTATTAAAG--CCCG-----CTTCCACGTATACTTAAAT 26879
Gallid          TTACATTTCTAAAACGGATAGCTGAAAAAGGACCCAATGGACCTCTTAGAAATCGCGGAT 30230
                **   * ** ***   ** * **   ****  ***      * **   * *  *    **

Human           CAATACCCACCTTTTTA--CAGGAATATTGGTGAACTGAGCTACGACGAAACAGTCGTTT 26937
Gallid          CGGGTTGCATCAATTTAATTGCGCCTGCCGATATTTCATGCTATGGCTCAACTAGACTTG 30290
                *      ** *  ****     *  *   * *       **** * *  ***     **

Human           AC---TTT--CCGGATGTTCAATCTAAAAAATTGATTAATCTCTTCCCATCTTTTTAAAA 26992
Gallid          ACAGGTTTAACCGAGACTTCGAAGATGATAGTCGGGGAATGCCATGTCGAGCTAAAGCGA 30350
                **   ***  ***    *** *     * * * *   ***  * *  *    *      *

Human           TA-----AATAACATTTCAATAACTACGGATT------TAGTAACCGTCATACTCAACGT 27041
Gallid          TGCGCCGAACCACCTCTGGCAGCCGACGAGCCAATGCCTAAAAAATGTCGTAGGCC-TGC 30409
                *      ** ** * *      * ***          **  **  *** **  *   *

Human           CAGAGCCGGCAAAA----TAAATCTATACATAGTAGAAAAGTCCATTTTAAATAGATATT 27097
Gallid          AGGACCTCCCAAAGGTTTTATATCGACACGCGGTGATACA--TCACCTTCATCGGATAAT 30467
                  ** *   ****     ** *** * **   **   * *   **  ** *   **** *

Human           TAACGTATATAAT---TTCGGTATTTGACAAG-----------ATATTCGACAGTGACAT 27143
Gallid          AATCACATACATTCAATTCAATCCTTAACGAATGGAGACAGTTGCGTACAACCGTGGGAT 30527
                 * *  *** * *   ***  *  ** ** *               * * ** ***  **

Human           TAATACAGGCAAAGGACACAG--TTCACGGCATTCTTGAAGCCGTTGACGCTTCCACACA 27201
Gallid          -ATTATAGCGAACGCGTATAACATCCATGAAAAT-TGGAAACAACTACTACTTCCCGAAC 30585
                 * ** ** ** *   * *   * ** *  * * * *** *   *    *****  *

Human           TCCATCCTCGTCACAG-CCAAAGCTCTGTTC----------CGCTCAGCT----CGGCAG 27246
Gallid          TGTGTTGTCTTAGAGGATCAGAAATCTTAGCGGAGTATGAGCGCAGAGCTATTACCGAAG 30645
                *   *  ** *    *  ** *  ***   *          ***  ****    * * **

Human           CTCTCCGTATTATAAAAACAGATACTGAATCACACGCTTTAAGAATTAAGAATCATTGGC 27306
Gallid          AAGTCTATCCCCCCAAAATGGATATTT--TCGCATGGACCAGGTATTGCGCTCCAGAGTC 30703
                   **  *      ****  **** *   ** ** *    * * ***  *   **  * *

Human           TGCTTGCCCATCCGT--TAGGACAT--CTCAT-TCTTAACACATATCCAATACAATGGTA 27361
Gallid          TGTTAAAGCAGTAATCGTAGGACAGGACCCGTATGCTAACCCGGGTCAAGCGCACGGCTT 30763
                ** *    **    * *******   * * * *  **** *   ** *   **  * *

Human           AAAAAACAGGATTAAAAAAACAAACAACGAC----------------AAATCTTATCATC 27405
Gallid          GGCGTTTAGCGTTAAACAAGGAGTTGCCATCCCCCCGAGCTTAAAAAATATTTATTGGC 30823
```

```
                           **  ***** **  *     *  *              * ** ****   *

Human           GGCTGAAATTTTTATTCTGTATACTAACATCCAACATC-TCCATACATCAAG--TTAAAT 27462
Gallid          GGTCAAAGCATGCTATCCTTCAGCAGATCTGGGAAATCATGGATGTTTGGAGGCTTGGAG 30883
                **   **   *    **  *   *  *  *   * *** *  **   *  **  **  *

Human           ACAACTCAAAGTATGACGTATCTATCTACTCTTCATCCTCAGATGATAA-ATCATAAATG 27521
Gallid          TAAACGCGGAGTTTTATTGCTAAATTCTGTCTTAACTGTCAAGAGAGGAGACCCTGGATC 30943
                  *** *  *** * *    *  **    **** *   ***   **  * * * *  **

Human           TAATCATTCG--AGAATGTGGGCCCTAGA---AAAGTATGAGGACACAACGGC-----AG 27571
Gallid          TCACCATTCGGTAGGATGGCAGTTCTTCATCCGAAATATACTTCGGCGACTGTCATCGAC 31003
                * * ******  ** ***   *  **  *    ** ***       * ** *      *

Human           AATTTCTTGGACGGTGCTAATGTTCTGATGGAAAAACTTATAGCGACGATATCCCC---- 27627
Gallid          AACGCGTGGTATAGTATTTATGTTATGGGGAGCGCAAGCACAAACCATGTATTTTCAGAC 31063
                **    * * *  **  * ***** **  *     *   * *       ***   *

Human           ------TAACAATCCCCTGTCTTAAAAAAGATTCAA--ATTCGTCTGCTAT-TTTAACAC 27678
Gallid          TGATTATGACGACCGTCACTTGGTACTGAAATACAGCCATCCATCTCCCTTATCCAGAAA 31123
                      * ** * *  *  *    *   * ** **   ** * *** *  * *  *  *

Human           ATGATCCAATTCCTGTTCCGATCGTATCAATACCATAAATTGCCCCCGATAAATCCATCA 27738
Gallid          ACCGTTTGCTACCTGTACACATTTCAAAGAAGCCAACGATT-TTCTCAGTAAGATAGGTA 31182
                *   *    * ***** *  **   *   *  ***   ***   * *  ***       *

Human           TCACAAACATCGTCTTCGGA--TACAGTAATGTTTTTCCAATGCTC--CCAATAACATGC 27794
Gallid          GAGGCTGTATCGACTGGAGCCTTACCGCGTAAGTGACGGGATGTGTAATCATTCACATGT 31242
                        **** **   *   *** *      *      ***      ** * *****

Human           CCGTGATACGG-----CATGCTCTCGTAGACATCCG--AAAAGAAATTCCATTCCATCCT 27847
Gallid          ACAAAAGACCGGGTCACAAACACCTATCGATATCAATCATAATGGATACCAACACA-CCT 31301
                 *   * ** *     **  * *   * ** ***    * **   ** ***   ** ***

Human           ACTAGGAAAATCTTCTTTCCTAGTAAGAAATAAAA--------CAAAAACTCCGTCGTTA 27899
Gallid          TCCGTATTAACTGTGTTAGCAAGCTGGGGTTGGAATTGCGCATCATCCAGTCCGGCAACT 31361
                 *      **   * **  * **   *   *  **        **   * **** *

Human           TTTTCCAAACAAGACTTCATAATGTAATGCCTCCCA------CTATTTTTATAAA----C 27949
Gallid          CAATGCTTAGAGCGTTCCACGCAGGGATGCCTGCGAGAAGGGCCCTCAGCACAAGGTTGC 31421
                  * *  * *    * **    *  ****** * *      *  *    * **     *

Human           GATCGTAGCAAATT--TCCATAAATTATCCTGCACATCAATCAACGATTGAAGAACATCA 28007
Gallid          GATTATTGCGAACCGGTGAATGTGGAAAACGGCCCACCTA-CGACGTTTGTGAAAAATCA 31480
                ***  * ** **    *  **      *  * ** ** * * * *** ***    ** ****

Human           TCAATTTTTGTTAAAAAGCGAATGTCCTTTTCTCGATATACCGGATCATAACGACGCAAT 28067
Gallid          GGATCACGCGAAAAATCAAGAATATACCCATTTGATACTCTATTTATGGTTTCCTCTAT 31540
                  *      *  ***    **** * *   * * ****  *    *         * * **

Human           CCGAGAACTGCAAA----TCGTTCCGTCGTTTCCGCCAACAAATAC---AGTGTCTCATT 28120
Gallid          CGATGAATTGGGAAGACGTCAACTGACTGATACTATACGAAGAGACTTGAGACATTCACT 31600
                *   *** **  **    **         * * *        * * **   **    *** *

Human           CACGAAGTCGA---------GACCATAGAATCGTTCGTCTTCTCCCACAAACACCA---- 28167
Gallid          TGCCAAGTTTACCATCGCTTGTACGAAAACTTCTTCATTCTCTTCATCATATGCTACAAG 31660
                  * ****  *          *  *  * * *  *** *  *** *  ** *  * *

Human           ACGTTTTAGTTAGGCACACATCACGAGTCTCCTTTCCCCAGGCAGAAACATATCCAAGTA 28227
Gallid          AAAGGGTCGTAAAAAAAAAGGGCCCTAGAACTCAGCCGAGTAACAAGAGCTTGCAAATGTT 31720
                *     * ** *   * *   * * **    *   *      **  * * *    * **

Human           GTTGCAATCTCTCTGGGCA---GCAAAGCAATAACGAACCTTGTT-TAAAAGGAAAAATA 28283
Gallid          CATATTATGTCGTAGGGCGCACGCTAAACATATTCGAGCACAATTACAAGCGGTAATCCA 31780
                  *   ** **   ****    ** ** **    *** *    **  **  ** **   *

Human           ACTTT----ATTTTCACAACAAAAAAACGAGAAAGACACATTACAAAACTGACACGA-AAC 28338
Gallid          AGCTCGAAAACCTAGGAAATACTATACTCGGGCTATAGATGGCAGCACGCATCCAGTAGT 31840
```

```
                      *  *     *  *    ** *  * **  *    * * **  **  **  *  *   *

Human             ACTGATACTTACACAT----TTCTTGCAACT--CTGCGGCAGACTTTGTGGGAAATGGAA 28392
Gallid            ACCCGTGTTTGTATATGAGTTTGCTGCAATAGATACTGTCAGTCTACATCGAGACAACGT 31900
                  **   *  **  * **    **  *****        * *** **   * *  *

Human             TTTTACACAGTGTCGAAATCACCAAGCACCGACTTTTCGGCCAATTAAACTGTATAGTTA 28452
Gallid            GATAGTAGATTCTTCTGGTTCGTGAGTGCCTCGCTTGCATATAAT-AATGTTTGTAGTCT 31959
                    *   * * * *     *     **  **    ** *    *** **  * * ****

Human             AGCCGGTCTT-----CTGTTCAA---CGTATGTCGATAAAGAATCACGAAAAACTGCAAT 28504
Gallid            CCGCGTCTTTGGCATCTGCCTCAGACTATATGCTTTTCTTGAAT-ACGACTAGGCAAGCT 32018
                     **   **     ***    *     ****        **** ****  *       *

Human             TTTTATATCGTTTAAATTCATTAGTGCAATGTCAT--AACGAGTTT-TAACATAGTCGCC 28561
Gallid            GTTTACAAACTGCAGATT-ATGGCTCCCCCTGAATCCAGCAAGCATATTACGCAGAAGTC 32077
                   **** *   *  * *** **   * *      **  * * **  * * **  **  * *

Human             GATTCGTTCGTCTGCCATCTCTACGTTATGAAACCGTCAACTACCCCT---TTGGACCCA 28618
Gallid            CAACAGAGTTTCGAAAACAACTA--TTTCGAAAACGGCTAGTACATGAAGATGGTATACA 32135
                   *   *    **    *   ***  **  **** ** * * ***       * * *  **

Human             AAACTACAAATAAACCAGCTCGCATTT-CTTATCCATCTTTTATAGCACA-CAGAGATAA 28676
Gallid            GGATGACGAAGAATTCGATATACATTTGCTTATTTCGCTGTCCACCCATAACCGAGATAT 32195
                    *  ** ** **  *      ***** *****    ** *     ** * * ******

Human             AAAACTCAACTTAATCAACTACCATCCAAAAACAAGGAACGTCATAGTGTTGGACTAAAA 28736
Gallid            GGAACTTAA-TGATACAAGTATGTTATAATAGTATAGATGAATAAAGTG--AGACTTATA 32252
                    **** ** * *  *** **   *  ** *  *  **     * ****   **** * *

Human             TTACCATTTCGTTCGCCCATAGAAAATCAACAGAGACT-ACCGGACAATTCCCTACGTTA 28795
Gallid            ATACT-TATTGCATAGATGTGTTTTATTACTTGCCTTTTACAGGGGTAGTTTGTTTATTA 32311
                   ***  * * *        *     ** *   *    * ** **   * *   *   ***

Human             ATAATATCCATCCTTGTGTA-TGTAAAATAATACAATGTAAACAATAATGATTCAACAAT 28854
Gallid            CCTACAAGTATGTTATAATAGTATAGATGAATAAAGTGAGACTTATAATACTTATTGCAT 32371
                     * *   **  *    ** * ** *  **** * **  *   *****  **     **

Human             GACCTTGGTTTCTT------CTCATACAA------CTGATTTCCCGTAAAGGAAACGAAT 28902
Gallid            AGATGTGTTTTATTATGTGCCTTGTACAGGGGTAGTTTGTTACCAGCAAGTAAAAAAGTT 32431
                       ** *** **      **  ****        *  ** ** * **   ***    *

Human             GATAAAAAATCACAG-----TCGCGGAAAATTC----AAATACTCGTCATATGAGAAATT 28953
Gallid            GGAACTAGATCAGGGATAAATTTTCACAGATTCCAGGAGACACGAGTCTTCCGTGAAGCC 32491
                  *  *  * ****  *     *      * ****    * * **  *** *  * ***

Human             CCCTAAAATC--TTATATATAAGAAAGCCACAAACCCTCACAACTTCATAGACACCAAAC 29011
Gallid            GTATGGCATGGGTGATGGATAATTGACGGGGGAACACTGTGAATCCTCCATCCGCAAATC 32551
                     *   **   * **  ****   *      *** **   **      *  * * ** *

Human             T-TTCTGAAGAGCAAAAAGAGTAGCAAATCACGGCCACCATTACAACTATGTTCGCCTTC 29070
Gallid            CGCTTCGCGAAGCGCTTCGATTAGAATATCTTCTCCACTTATATGAGGAAATTTGTC--C 32609
                     *  *   ***     ** *** * ***    ****   **  *  *  ** *  *  *

Human             AACATCAACATTTTCT--TCATCTATTCTACCGGAAGAAACAATCCAGAGATTATCTCTC 29128
Gallid            AGTATCGGGGGTGTGTGCTCATGTAAAGTGTCAGTCGGATTGGCCCCGACATATTCTATG 32669
                  *  ***     * * *  **** **   *  * *  * *     ** ** **  *** *

Human             AAGATAAACAGATCCATAAGCGGCAGCCATCGAATGATACTGACATTGAAGTGGTAAGAA 29188
Gallid            AATCCAGGAACGCTTTTAGACAAGACTTGTGCTGCTTCGCTATTCTGTTGAGGGTCATCC 32729
                  **   *   *      **  *   *    *         **    *      *** *

Human             ATATCAAAAAACGCGGATACAAATTTTTACCGATAGAAAC-GCGATATTTATTTAAGCAC 29247
Gallid            TTTCTAGGTTCTGCTACTACAGTGGGATAGAATTGTCGACTACAATTTTGAACCAGATGC 32789
                   *   *      **   ****      **    *    **  * ** ** *   *    *

Human             ---TGTTCAATCCTTTCCACAGGCAGACT-TCTTACCCGAAAACTACAGTAGTCGAAT-- 29301
Gallid            AAATTATCAACAGTTTTCGCAAACGTATCATGTCTCCCATATATGGTAATGGTCATATAT 32849
```

```
                   *  ****   *** * **  *  *   * *  ***  * *    * * ***  **

Human           -CCGTAATTCCCACCGACAAAACAAAACGGTAAACACTTTTATCGTTTTGT---TATACA 29357
Gallid          ATCGAGCTTCCCATCCACATAATAGTTAAGACGCCGCCAGAATTGTTATACACGTACGCA 32909
                  **   ****** * *** ** *     *    * *    ** *** *     **  **

Human           CTAATACCCAGGAGAGCTCAGATTTTGACTGATCTG-CACATTACGAATACCCCAGA--- 29413
Gallid          TCTGTCCCCATTACAGTACATGTAGTAAATGGTATGACGCATAGCGATTCGGGCAGAGAT 32969
                    * ****  * **  **  *  * * ** * ** * ***  *** *    ****

Human           -------ATAT----TTACACCTGTTTGCGATTTTTTTA-----TAGG-----------T 29446
Gallid          GAGAGCGATGTAGTATGATACTCGGATCCACTTTCATCGGCGCGTAGGAGATAAGAACAT 33029
                       ** *    * * **  *  * *  ***  *       ****           *

Human           CTCACTGCTCCACATTCTAACGACACTGGGAATGAACGTTA-TCCGTGCTTCTCTATCAA 29505
Gallid          ATGTCCGTTCCGC-TGTCGACAACAATGAGTGTACCTACCGGTCCTCGTTGTATGATAAA 33088
                 *  * * *** * *    ** *** ** *  *         ***  * *     ** **

Human           ATACG---------CATTGACGCAGATGAGTAAGTTTTTT-CACTAATCCA-----TTTT 29550
Gallid          ATTCGATGATGGAATATGGTCACATCTAGATAGCTTGCCGACGGCAATCGAACGTGTTCC 33148
                ** **          ** * * **  *   **  **     *   **** *     **

Human           AAATCCG-ACAGAAAAACA--------------CACCATTGACTATAT--GTAAT----T 29589
Gallid          AGCTTCACACGAGCAAATAATTTGCTCGTAGGGTGGCATTGTCCATGCTGGTGATGCTTT 33208
                *  * *  **    *** *                 ***** * **    ** **    *

Human           AATATTTTTTTATTTCCGCA-GATATGATTTAATCCGACGAC---CACTA-CATGTTTTA 29644
Gallid          GATATTTTCCAGTGTATACGCGATAAAGGTAGATCCCATAATATGCACTAGTAGAATATA 33268
                 *******    * *   *  ****    *  **** *  *    *****  *   * **

Human           CT-GTCCACGATC--ACCTTTTCC--------TTAATGCA-AATA-GCACTCACGGCACT 29691
Gallid          CTTATATAGGACCCAACCCTTCGCCAGCCGTATCAATAGACAATATGAACCTTAGGATCT 33328
                **  *  * ** *  *** **  *        * ***  * **** * **    **  **

Human           TTA--------TAGAAAATGTCTCACGATAGTGCACACCAATTTTCTGAATGTTTTC--A 29741
Gallid          CTAAGAGCTGCTAATAAATGTTCACTAATATTGTTGTCATCCCTCGAGTGTGTTTCCCGA 33388
                 **        **  ******      *** **    *     *   *  ***** *  *

Human           TGTAATTTAATAAACATTTATTTGTATCCTAAATCTTTCAC--AACATGTTTCCGCGTAA 29799
Gallid          AGTGGATTAATATTCATTCTCAAATATTTAGAAGAAGTAACTCGGGACATTGCTACGTAT 33448
                 **   ****** ****      ***    **    * **     *  ** *  ****

Human           TCATTGATAAACAGCATAGCAATAACAGCATCGTAAATGCACCCCCAGCCAAAAAGAACA 29859
Gallid          ACACTGCTAAGTTTAAGCCCGGAATGCGCAAAGCAAATGGCTACTCGTTCTAAACTAAGG 33508
                 ** ** ***     *   *   *   ***  * *****    * *   * ***  **

Human           ACCCAGCAGC---CGACATTGAATGCGTAATTTCAGACACATTCTCGTAATCCCTTGAAA 29916
Gallid          CCTTGAGATCGCGCGATAGTCATTGC-TAATTTAGAGCTTATTC-CATAATCGGCAGTGA 33566
                 *     * *   *** * * * *** ******    *  **** * *****    *  *

Human           AATCCAACAACGCTTCA--ATCTCCTTGTATTTATACCAATTAGTCCATGCAGACTTCCA 29974
Gallid          CGGCCATTTTTAGTTCGTGATCATCAACACTTTCGACAAAGTCATTGATATTACTGTTCA 33626
                   ***       ***   ***  *     ***  ** ** *  *  **       * **

Human           TGTATTTCTTAAGAATTTTTTCGGCATC--GTATCATTTTTAAT-TAATGAA------AA 30025
Gallid          CCGCGGCCATAAAACCATGTTGATCTTTAAGAACAATCGTTGGCATATTGAGCTCACCAA 33686
                       * *** *   * **   * *   * *  **  **    ** ***       **

Human           ATATTTCGAATGCACAACCTTTTTCTTGTAAATTTCATCCGACAATTGAGGACAGATCAT 30085
Gallid          GTACCTCGGCAACGTGAGGTTGTATTT-TGCGTCTAATCGGTTCATCGGCAAATGCATTT 33745
                 **  ***    *   *  ** *  ** *   * * *** *   ** *   *  *    *

Human           GCTCAGG-AAAT-----AGCTATCCAGAGACACTCTCTCATTTAAAATGTTCAAAGTGCC 30139
Gallid          ACTCGAGCAAATGTATAACCCATCAAAGTGTAGCTGTCTGTTTGAAGAGCAAGGGATAAC 33805
                 ***  * ****     * * *** *     *        *** **  *       *  *

Human           ATTGGCATCTGGCCCGTCACTTGTCCATTGACTCGCATTCATTTCAGTGC---TGGACTT 30196
Gallid          ATTC-CCCCTCGCATGTTGTTTATGAAT-ATCTCACATCCTCTCGAACTCACATTATCGA 33863
```

```
                ***  *  ** **  **   ** *  **   *** *** *  *  *   *   *   *

Human           GAGAACAATCCGTTCCG--TTCCATAAAATAGAGTCATCACACCTCCTGCGCTAT-GACC 30253
Gallid          CATAACTATCGAATCTGGATGTTGAAAAATTACGCCCAAACAGCTCCACCATTATAGAGT 33923
                 * *** ***   ** *  *     *****   * *   *** ****  *  *** **

Human           TTCTAGCCAGCAA-------TTTACAGTACTCGATAGCTGAGCGGCATTTGGAAGCC-TT 30305
Gallid          ATCTGCCCATGAACATGGTCTTTAGCATGCTAAATTG-TGTGTAGATTTCAGAAGAGGTT 33982
                 ***  ***  **       ****   * **  ** * ** *  *  **  ****   **

Human           TTAGCG-ATCAAAGCTCTGAGCATCACTCCTCTAAAATAATAATT-AATAATCACACGTA 30363
Gallid          TCGGCGTGGCCGAACTCGTAATTGCAATACAGCAGATCAATCATTTGATCGTCCAATGCA 34042
                *  ***   *  * ***  *    ** * *   * *  *** ***  **  **  * * *

Human           GAAAAGCCGTTTTTTCAGAGATAGATGTGATATTTCCAACTTTC-ATGGCTTTAAAGCCA 30422
Gallid          GAAAATATAACATCATCCACATTTTCATGTCTTTGCTGGATATCTACATCCTCTATTCCC 34102
                *****       *     * **     **   ** *    * ** *   * *  *  **

Human           TCTGTT--AGCATCGCAAGGTTGATCAGATATGAAGTTCTGACAGAAACA-----CAGCT 30475
Gallid          TTTCCTTCAAAATGGCAAAGGTCCGTCGGCACGATCACATCGTTAAAATTTTCTCCATCT 34162
                * *  *  *  ** **** * *     *  * **     *     ***       ** **

Human           GGAATTCCCAGCTAACACTCCATTCACATAAC---AAGTAGAGATG----CATATAGCTC 30528
Gallid          CCAGCACTCAACTGTCGCTGCGGAAATAGAATCGTATTTGTAAGTGTTGCCAATTTGTTA 34222
                  *   * ** **  * ** *    * * **    *  *  *  **    **  * * *

Human           CA---AGTTTCTGTTCCTGAATTAAAACTATTTTTTGCTCGCATGTGAGATTCGATGTAA 30585
Gallid          TATGCAGTTTTTACAGCATGTTCGGAAAGACTCCGTTGTTGCAAGTAGTTATAGAAATTT 34282
                 *   ***** *    *    *   **  * *   *  * *** **     * **  *

Human           AGCAGACTCCC--ATTACAAA---GGTATAAACTAACAAGGA--CTCCATAGTTAACTTC 30638
Gallid          ATCATGCCATTGAATAGCAGATGGGATAGGAAACGGTATGCATACTCTATGGATC-CTTC 34341
                * **  *      **  ** *   * **  **     * * *  *** ** * *  ****

Human           TTCATG-ATATTTAGAA----------TTCACAGATCGTTTGCAATTTCTT-------CC 30680
Gallid          TCCGTGTGTTTTTATAAACGAGTCATCTTTTAAGACAGATAAAAAATTCTCAAAGGTGCC 34401
                * * **  * **** **          **   ***  * *   ** ****        **

Human           AAAAAATCTG----CCGCATCTCTGTGGAATACAATAGAAACAAATTTGAAACTTTATAA 30736
Gallid          GCAAAAACCGAAAACCCATTTCCTAAGTCGTGTAGTCACAGCCACTTGACTATTCAGTAC 34461
                  **** * *    **   *  **   *   *  * *   * * * **    * *   **

Human           AAGAACAAACCCATT-TAAGACGCGGCAACACAATTCAGTCATCTATAATCCACATCCAC 30795
Gallid          GTAAGTTATGTCAGAACATGAGATTGCAAGACCTTGTTGGCTTCTA-ATTTCGCATCTAG 34520
                   *   *   **    * **    **** **  *   * * **** * * * **** *

Human           TTT-----------CGTGCACTCTTGTACCAGCACTTGCCACATGCATCAAGTAAAAACA 30844
Gallid          TTTGTGTTGCGTCTTGATCACGGCTTTGTGACCAGTTGCT-CAAACGCCCCGCATTGACA 34579
                ***            *  ***   * *   * ** ****  **  *  *  * *   ***

Human           TCCA-CCATCCCTAAAAC-TACCCT----TTGCGCGCACGCTCTCCCCATCCGTGAAACA 30898
Gallid          TTGAGCCATTTCTCAAGTGTAATATGAGGTTGATTGGCAGCCGTACGATAGCGTTCAAAA 34639
                *  * ****  ** **   **   *    ***   *   **  * *     ***  ** *

Human           ATTAAAAAACCTACCTCAGCACAGCT--GCCGATTCATGAACAGCATTTTCTGGGCTCTT 30956
Gallid          TTCTCCAAACTTATAAAAGTATATGCAGGCAGAGTAAACACCACAAACTTATTGTTGCCG 34699
                 *    **** **    ** * *     ** ** * *  * **  *  ** * *   *

Human           AAG-----CCAATGAATGTTCGGTTTGCATTCCGATGGAGTAGCAATGATACAATAACCT 31011
Gallid          GAGGTTTTCAAGTAATCATGTAATCTACTCATATATGTACTGACTTCCTTATGAGATGAA 34759
                 **     * * * *   *    * * *      *** * *  *     **  * *

Human           TTTAAAGGTTTTTCTTGCGCCAAAGACGCACCACCCTGAACA--TGTTTACA-ACCTACA 31068
Gallid          TATAAACGTGTCCAGCCAGGTAGGTTCGCCGGATTATTGATAAATGCTTCCGGAACAACA 34819
                * **** ** *       *  *    ***   *   *  * *  ** ** *  * * ***

Human           TCATCGGTTGCCAT---AGCACCGACCACTGTTGTA------TCGTACACCGAGTCGATT 31119
Gallid          AAGTTATCTGCCATTCGGGCATGCTCCTCCGTTATAGGAAGTCCATATTCCAATGTTTTG 34879
```

```
                   *    ******    ***    ** * *** **       * **  ** *     *

Human           CGAGGATCAGAGACATCAAAGGTATTTTTTGCGGTCGCTTGGACTTCCGTTTCCAAAATT 31179
Gallid          AGAAGATCTCCAA-ATTCGGGTTCTGTACAGCGTTTATTATTAATAAATATTGCCCAATT 34938
                 ** ****    * **    * * * *   *** *   *   * *     ** *  ****

Human           GACTGATGAACCAAATTTCCACCGAGAAACAGAGTTTC-TGAAGAATTATCCACCACGTC 31238
Gallid          ATTGGATAAGTCTAAATATGATCGTAATGTTTGATTGCATATAATATAAGTCAGAATATT 34998
                    *** *  * ** *   * **  *       ** * *  *  ** *  **  *  *

Human           TTCAGTTTGAGTGGCA--GCGTCACACATCGCC----TCAGAC--CGATCTGTCTG---- 31286
Gallid          TTCGCTTATTCTTACATTACACCTCAGTTTGCTATGTTCAAATGTCGATTCCAATGAACT 35058
                ***  **    *  **   *  * **  * **     *** *   ****     **

Human           TGTATGCCTCGATAACATAATTTTTAT-ACCAGGAGTTTTGGTAAGTCCATAGATTGTGC 31345
Gallid          TGTTTGTGTAGGCGACCCGACACATATTAATACTGGTTTTAACCCTCTTTTATATTGTGG 35118
                *** **  * *   **   *    *** *  *   *****          ** ******

Human           AAT--GGCGAGCGGAGTTGTC---TTCTTGACACGATAAA---GCCATACACAC-CGTCC 31396
Gallid          AGTACGATACACGGCATTGGTAAGCCACCAACAGTATACAATAGCCGTAAGTAAATATTT 35178
                * *  *     ***  ***           ***  *** *   *** **    *    *

Human           CCCCAACA-TCTTTTTTCGCAGATGGCTGCAACAATTCTTCCGTCGGCCAACAAAATGGA 31455
Gallid          CCCCAACAATCCGGCTTCATCGATGATGATTATATTACTGCGTGTAAATGATGGCAGTGA 35238
                ******** **    ***   ****      * * * ** *          *    *  **

Human           CCCTTAGCACCGAAAGAGTCAGACTGAATTGGTCTCATGGAAGCA---CGAGCATCTTCT 31512
Gallid          TCCAT-GTATCCCGAAAGTCATCCAAGACAAGTTTCCTCTAGGTTTTCCCAGCAGATCTT 35297
                 ** * * * *   * *****  *   *   ** ** *  * *     * ****  *  *

Human           TCCACGCAAGCACCACTCTCTACCTTC-TGAAACCGTCTAGGTAATACACCTGTCAAAGA 31571
Gallid          CTAATGTCCGTATTACATCAAATTTCCCTGGACCTGTCTCCG-AAT-------CCAAAAC 35349
                   * *   * *  **      *  * * ** * * ****  * ***         ****

Human           AATTGGGGTATGTCTCATTCT--CGTAAAATATTTTTGAA-ATTGATCACTGTATTCATC 31628
Gallid          TTTCAGCATACTTTTCGTTATATCGGACAGGACTTCCCAATAATATACAATATCCTTTTT 35409
                  *  *  **  * ** ** *  ** * *  * **   ** * *   ** * *  *  *

Human           GCGGGACTTCTGTGCAATAAA---ATTACTCAAGATCGCGTTCTGACACGAGAACTCCAT 31685
Gallid          TTGTAATTCTTTCATTGTAGGTGGACTGGTCGAACATACATATTGCCATTTACCCAAATT 35469
                  *  * *  *      **      * *  ** *     * *  ** **       *    *

Human           GGCAGTTGTCCAGAAATCTGAAAACGTAGGTGAC--AAATAAAATATCACACCATCTTCA 31743
Gallid          GGCTTGAACATGGTTGCCTTTAAACCCAAATTCCTGGAATATAGTGTTTATATGTGGCGA 35529
                ***         *    **  ****  *  *  *   **** * * *       *    *

Human           TATCCAAGCACTTGACCAGTTGACGATAA-----CAAAAAAACAACTTGTCTCTCCGGAG 31798
Gallid          GGTATAGG-AATTACTGAGTTTGCAATATATGTTTTGAGATGCAACTT---TTGTTGTAC 35585
                  *  * * * **    ****  * ***         * *  ******   *    * *

Human           CCGCAGATGCACCTTCCCATTCCTCCGTTACACCAAACATACTCATCCTACCCTCAAAAG 31858
Gallid          CAGTAATAATGCAATCCAAAATCTCTGATAATGTTTGTATGCATGTGCT-CTTTCCAGAT 35644
                * * *      *  *** *   *** * **        ** *   * ** *  ** * *

Human           GATACAAC-CCGTTTATTAA-TAAACATCT-----CAACTGACGGTCAGATAGAATATCG 31911
Gallid          CCCGCATTGCCACTTATCAAATAAACAGCGAATGGCAAGTCTCGTGCTTCTAATTCTACA 35704
                    **   **  **** ** ****** *      *** *  **  *   **      *

Human           GAATTGTCACAGATCCGAACTTTGATACAAGTGATATGTTGAAGAA--CAATTC--CGGA 31967
Gallid          GGGTTTTCATGATTCGAAGCTTCTATA-AAATAGGATAACGGTGGAATCAATTCATCGCT 35763
                *  ** ***    **  * ***  *** ** *   **   *  * *  ******  **

Human           AGCCATAGCTTCCTGTACAACTTTCTCA-----AATCTCAGAGCGGACCCCGC------C 32016
Gallid          CAGCGTCGCGTCCGCAAGAGCTCTTACACGAGTAATTATAGATTGTATTCCATGCATGGC 35823
                   * * ** ***   * * ** *  **      ***   ***  * *  **        *

Human           GAAGAAATCATCTTA------TCCTTAAACTGCAACATGCG-CGCCAGTT-CATCACCGG 32068
Gallid          GGTAAAATTTAAATAGGTCGCTTCGCAAAATAAGTCATTCGATTCCTGTGACATTTTCAA 35883
```

```
            *   ****     **      * *  *** *    *** **   ** **  ***   *

Human       TGAATGCCATTACGATGTTTTTTCTCGTTAATGATTAAGATAATCGGCCATCCAAATATG 32128
Gallid      TAGACTCGCAAAAAATTAAATTTTTCTTGAATTATTTTAGAGGCGTGTCGTATTTCT-TG 35942
            *  *  *    *   *    *** ** * *** ***          * * *     * **

Human       TAAGAACTG--CACAATCTTAG------TTA-AATTTATACGAAAAAATACTGCAAC-TC 32178
Gallid      TAATGGATGGCTATGATCGAAGAGCGCCTTACGATTTAAATAGTAGAAAAATTAATCATC 36002
            ***    **   *  ***  **      ***  ***** *    * ** * *  * * **

Human       CGGAAATGGGTGTGGCATAATCTCTAAAAG-CACCTTAAGAAAAAAAACAGACACTGTTA 32237
Gallid      CGGACTTCGAATCGGTTATGTCGCAACGAGATACTTTTAACAACGTCTCATCAAAACAGT 36062
            ****  * *    **     ** * *  **  ** ** *  **     **   *

Human       ACAAAAAAAACCAATTTATTTAAAAAATCTCTGTTCAAATCACCTCACAGTCATGGAAAC 32297
Gallid      GTATGGAGAATCAGTGGATTGTGATCCATCCTACTCGTCAGACACGGATGTTTAAGGAGA 36122
              *   * ** ** *  ***   *      **  **     **      **    * *

Human       ATTTTACAA-------AGGCAACATTTCTAACTTGACATTGCTAAAACTTTCGTGAGATT 32350
Gallid      TCCTCGCAGGTCGATTAGGATATACTGATGGCCAGG-GTATATATAACTC-CGTAAGATC 36180
               *  **        ***  * * *  *  *  *   *   ** ****  *** ****

Human       T-TATTGACTGTCGTTCTATA--TCAAACGAATTATTTCCGCAGACAACTGCTATTGAAT 32407
Gallid      TACAGAAACCGCAATTCGACAGATTCAAAATACTATTCTCACCCTTAGCCTGGATGCGGT 36240
            *  *   ** *   *** * *  **   * ****  * *    * *    **    *

Human       CATCACGGACGTTTTGAACGCT-ATGAAGTTCCATGTCGTGATTTTCA-GCTTCGTAGTT 32465
Gallid      TAGATATGACGATCTAAAAAATGATTGGATACGGCATGCTGACATGCGTGGGATGTCAGC 36300
             *     **** * * **   * **    * *    *  ***  * *  *    **

Human       GCAGTATTCTACTTCCATAATCACCATAGGCACTTCATCATTCCCT-CTACGCCTTTTTA 32524
Gallid      AAAAAAACTTGCCAGAACATATGGTATGCATAGTGAAGCAGAAGCTGTTAAGGTAGCAGA 36360
              *  *   * *    * *      **    * *  * **    **  ** *       *

Human       GCACCCTATTTGGATTCGATGAAGATCCCCCAGAAACAAAAAACTGAATATACCGCGTTA 32584
Gallid      AAATGTTTTTGTAACATGGCGCAAAACTCTGCAAACTACATTGATAAATTTGGCTCGTCA 36420
              *   * **   *   *  * * * * *    **  * *    * *** *  * *** *

Human       A--AATATATAGAAACA-TACACCCAGCA---GCAATTGGAATTACAATGAAAATCCACA 32638
Gallid      GCTAATTTCTTGCTTTACTACGGCCAATATAAACACTTCCAGTTTTTCCAAATATATAGA 36480
               *** * * *    * ***  ***  *    ** **  * **      ** **  * *

Human       ACAGCTGCAATTTATTAAATTTTTTTCTGT-GCGGCGCTGGAAAATTATGACGAGGAAAC 32697
Gallid      TTGGATTTGTTGCCTTGGTATCGTTCCAGTTGTTAGACATGATCGTTCTGCAAGGGCAAC 36540
               * *    *   **    *  ** * ** *     *  **   ** **    ** ***

Human       GTGTCCTCGGATTCCTT---CACGGGATTCACAAAACCTTTGAAACTAAAAGCATCCTCT 32754
Gallid      AAGTTCTATACCTACATGCGCAGGCAGATCAACTGTTTTTCCAAACTGGGCATTTCATGA 36600
              ** **     * * *   ** *    ***       **  *****       ** *

Human       CTTGTTAAG-AGAGCTGGGGTAACTAATAAT-TTATGCGTAATTTCACATGAAAACTCA- 32811
Gallid      TGCCTCCAGTAGACTTCGAGTCGTCGATAGTGTTATGGCTCGTGGAAAACAAATTGTGAA 36660
                *  ** ***  * * **     *** * *****  *  *   * *  **   * *

Human       CGATGTGTCATGCTCAAAGCTCTACTCAATGCTCTTTCATAAGAGTATAGAA-GTTGACC 32870
Gallid      TTACTTATCGAATTCGATG-TCGGCAGTAAGCATTCTCG-AATATGACAGAACATTGATA 36718
              *  * **    ** * * **  *   * **  * **  ** *  * ****  ****

Human       GGAAATT-TCGGTTCTAATGAAATCGTCAGTCGTATGATCATCTGAAACTGTTTGGTTGT 32929
Gallid      GAATATAATTTCTTTAAACGAGAACTTCGGGTAAAGGAT-ATATTGAGTGGT--GAGCGT 36775
            * * **  *   **  ** ** * * ** *    * *** ** *  *   **  *   **

Human       GAAGACTCCATTGA-ATGTTCGTTCCAATGGGCCGATATCGTCGAGCTGTACA-GACGAG 32987
Gallid      GGGGAATGTATTGTTATCTGGAGGCCAGTAATGAACGATGGCGGAGTTATTTTTGATTCG 36835
            *  ** *  ****  ** *     *** *        ** * *** * *    **   *

Human       CAAACCTGTATCTCTCTTGCTGAAAACTTGCGGAGGACGATATTTAGAGTCTCCGAAAAC 33047
Gallid      CCAA--TGCAACGAATATACAAGGAGATTATTGAATGTCATGATTTGCGTCAAC--ATGC 36891
```

```
                * **  ** * *     * *    *  **   **     **  ** * ***  *  *  *

Human           AACCTTACCATAGAAATTTAAGATGTCTTCTACACAAAAACGTCTTGCATAAAACGACAA 33107
Gallid          AACTTTATGTCGACTTGTCAACACGGCTCCTGTAAAAGTTCTTATTGCAAAACGCGATGA 36951
                *** ***          * ** * * ** **  * **   * * ***** **  ***  *

Human           TACGTGCAAAG----AACGATTTTGTAAAAAACTGCTCACA----TTTATAAGATCGTGA 33159
Gallid          TGGATGTAAGGGGGTAGCAGGGGCCCAACGAGTCATCGACAAAGTTTTGGGAGATCAACC 37011
                *   ** ** *    * *        **  *       ***    ***   *****

Human           ACAAAA--CTACGATTCTCATT------AAACAGATCTATCTGTACATAGGTT------G 33205
Gallid          AGAAAATGCAGCAAGTTCTGCTGCATCCAGATTAGTCAAACTGATTATTGGTCTTAAAGG 37071
                * ****  *  * * *     *      * *    ** * ***   ** ***       *

Human           TATTTCTCGCTAAAAAAACGTAGCTGCCAGACAACTGCGTATTATTGTTGACGAAGCAAA 33265
Gallid          TATGCGTCATGTGGGAGATATTACTGACA--CGGTTCGAGACTATT-TGGAAGAAACGAG 37128
                ***   **       * *  *  *** **  *   *    * **** * ** *** * *

Human           CCAATTCGCTTACAGAAAAAT-TTTGAACTGAGACATACTGAAGCCCAGCGTGCAATCTA 33324
Gallid          CGGG--CATTTATTGGATGCTGCTTCGATCGATACATCTCAG---CCGGGTTTCGGCCAA 37183
                *     *  ***  * *   *  **  *  ** ****        ** *  * *   * *

Human           TGAAATT---CACGTATTACCACATGTCCTAAAAAACGGTCTACGGTAGAATTTTT-TCC 33380
Gallid          TCCAATCGGGCACAAGCTTCTACAA--CCGAGGAAACTCGACGTAATACCATTAAAATTC 37241
                *  ***    ***    * * ***   ** *  ****         **  ***    * *

Human           TTCTTCCAGGCTTCCCGCGAGGTCAAACAACGTCTCTCCAACAGCCGTATGGTTCCGCAA 33440
Gallid          GGGATGCATTCCATTCGTCTGTTGTAACAAGTATAAACGAGATGCTAGAAGGCTATGTGA 37301
                    * **  *    **   * *  *****       * *   **   * ** *  *  *

Human           AGTCAAATCTCCATAAAGCCTCTCGTAAGCAAAAACGATATCGTTATCAAT--GCCGATA 33498
Gallid          A--TAAATTGT--TTAACACCGTTGAAGGCTTGAAAGCTGCCAATAAAGATTTGCTAGCA 37357
                *   ****     * **  *  * * * **   ** * *  *  **   **  **    *

Human           ACGGTCTGAAGTTTACCGATTCCTTCAGCCAGAGTAGTCTTGATGTAAAGTTTAGCCGGC 33558
Gallid          AAACTTTGT--TCCAAAGAATTAGAACTCGACAGAGTTCGTACTGAACATCTTATCT--C 37413
                *   * **   *  *  ** *       * * **   ** *  ** * *  *** *   *

Human           AACGAAGAAACGCATTGAAATAAACATGCGACAAAAACAGTTATCATTTTTAAATCTTTA 33618
Gallid          AAAACAGGCACACACTGATATGGGCAATTCTCAGTA-CAGCC-CGACATTAGAAACTTTG 37471
                **   **  ** ** *** **   **     **  * ***     *  **  ** ****

Human           ATTCACGTCG--AAGAAAGGTACGGCCCTTGAAGAAACAGTTAACCGCCGTAGTCAATAA 33676
Gallid          GCGCGTGACCTTAAACATGATGTTATTGATGTAGGTGAAGTTATGGATGATGATTCCTAT 37531
                   *  * *   **  * * *        ** **    *****       *  *   **

Human           CTTATTACAAAAAGGCCACATGGAATTAACACTAACA---AATATAAAGTATATTATCTC 33733
Gallid          GTTGCTA-ATAGTTTTCAGTCAAGATATATACCTGCGTATGATGTAGACTTGAAAAGACT 37590
                 **  ** * *     **      **  * **   *     ** ** * *  *  *

Human           CTTCAGTCCTACAAAACAGGAAATACCCAGA------ATACCCACACAAACCGCCACCGC 33787
Gallid          TTCTGAACTTTGGGAACAGGAAATGCTGCGATGTTTTAAATTGACACGAGCTACCA--AT 37648
                 *     * *    ********** *   **      * *   **** * *  ***

Human           ACCCATGAACATAAGGTTCACCATGATAGATGTTATCTCTGCGAATGCTAACATAGTCGA 33847
Gallid          AATCAGGGTCATGAGGTTT-CAATTTCCTACTCGAACAGTGCGATAACTTTACTACTCGC 37707
                *  ** *  *** *****  * **     *    * *  *****   **    ** ***

Human           ACCTTCGTTATAAATCTGAATAGACTTTT-CGACATTCTTCTC-CGGAAGCGTAACGATA 33905
Gallid          TCCTTACTTTTTTTCCGTATTGAATATTTATGATATTGGGCCAATAGTAACGAATCACGA 37767
                 ****  ** *    *  * *  *  ***  ** ***   *     * * ** * *   *

Human           ACATTATCGCCACTCTCCTTATTCGTGCACATGCAGAAATATTTATCCCCTACCCACTTA 33965
Gallid          GGTTTATAAATCAGAGGAGGAGTTATGCA-ATTCGGTATTTGAAAAGACACGTATTCACG 37826
                   ****             * *  **** ** * * * *    *   *       *

Human           TCCAATGAAATAAGTCCCCACGTCACATCATCGCGAAACAAAGACGTATATGCACCGGAC 34025
Gallid          TTTATCTGGATGATTTAGCACTAATCTTTAATGCCGATGTAAAAAG-AGCCATCGCGAAA 37885
```

```
                *  *     ** * *   ***    * * *  **  *   ** * * *       ** *

Human           GATCTCATCTTTACCGTT--TTCCATACCATATCAAACGTCAGCGGCTCCTCCGCAAAGA 34083
Gallid          TATTTCCTCGTCAGAAATAATTCGGGAAGGCAACGCGAGGCAGAAGATTTGCCTGACGGC 37945
                 ** ** ** * *    *  ***   *    * *    * ***  * *   **  *  *

Human           TCTCGATCTTTCCCAGCTTGCTAACA--CCCCAACCTG--AATATTCACAA-ACTGCAAA 34138
Gallid          CATCATGGAAGAAGAAATGACTTCCATTCTCCATCCTCTCGACGTGAGCGATACTCTAGA 38005
                  **          *  *  **  **  * *** ***    *  *   * * ***  * *

Human           TTCTCCTTCTGCGCCCAGCCACCTAGAGAACGAACATTTCAAA-----TCATC----AGC 34189
Gallid          CGCTCTGGTTATAAAC-GCCATAGATGGAACAGGGAATCGAGACGCGATTATCGAAGAAC 38064
                  ***    *     * ****   *  ****    * *  * *     * ***    * *

Human           TCTGAACAATCTTTCTGAAGAAGATC-TAATAACCGGCATAAAATTTAGTTTTAACTCCT 34248
Gallid          TCAGTCGACAACCAATGGCGAGGATGATGATGGAAGTCAGAGAGATTAATGGAGTCCCCA 38124
                ** *   *       **  ** ***  * **    * ** * *  *** *     * **

Human           CGTCGTAAGACAGAGCCATTCGTCTAAATTCGCCGTACAAC---TCAGATAGGACTTTAA 34305
Gallid          CCCAGTTT-ACAGGTGTCTCCGTCTATAAACTTCGAGTAGCGAACTGCATTAGACGCTTA 38183
                *   **   ****     * ****** *  *  **   * *       **  ***  * *

Human           CGTCGTTATTGCACGAAGATATCAAGTACTGTCGATATGTCGAGAGTTCAGT-CCAAAGA 34364
Gallid          CATTTGATTTAGCAGGAACTGAAACTGACGAAGAAATAAGCAGTGATATATATTACACG 38243
                * *     **   *    *    ** *   *  ** **    ** * *   *   * *

Human           CGTAACATCGTTCTGTCTTCACCCTGGAAATTCTTATCTGCAATCCA-AGAGTTATTGCT 34423
Gallid          CAATGTATAGCTAACCCTGCGTTCAAAGGTTTCATATTTATGATTCTCACGGCTATGGAG 38303
                *     ** * *    ** *   *      *** *** *   ** *  *  * *** *

Human           ACCATCGAGGAAAAAAAGCAACACGTAGTACTTGTTTCTTGA-----GATTTGTTTCATTC 34478
Gallid          GACGTGGTGAAAACGATAGGAATACCGCCCCCACTTCTAAAATATAGATTTGTGTCGTAC 38363
                  * * * * ***  *     *    *  *    ****  *     ******* ** * *

Human           ATTCTACTGAGTCGTATCGATTTT-----TTATTAACAGAGCATTGGACTTGAATGCTAA 34533
Gallid          CATCCAGCAGAACCTTTGGATTTTGCCCTTTGTTTGCTCATCTCATATTTGGAAAACCGG 38423
                  ** *      * * * ******     ** **  *  * *       * ***  *

Human           TAAATG---TCATTTT-TCCATGACCACATTTTTGAA--CACGCTTTCTTGCAAGATGCA 34587
Gallid          AACGTCAGCTCATCCGACCCGTCACTATATGTTCAACTTCAAAGTTTTCTGAAATATGCA 38483
                 *  *    ****     ** * ** * ** **  *   **   ***  ** ** *****

Human           TGGTTG-CACTACTCACGGTATTAAACA---ATAG-TTTATGTACGTTATGAAAAACCTG 34642
Gallid          TGGTCGACAGTAACGCCGATGCGCAAAATGCGTAGATTTTTATGTATTAC-AAATACTTG 38542
                **** * ** **    ** *    ** *    *** *** * *   ***  *** ** **

Human           GAAACATCGAAAAACCAAAAACTTTCCAAAGTCGTATTCAATTTGACTGTCTTCCATTCC 34702
Gallid          GTTAC-TAAATACCCTAATGGAACTGAGTTCTATTAGTCCATTCGATAGTAATCATGTTC 38601
                *  ** *  * *  * **      *      *  ** ** *** **  **  **   * *

Human           TCCGTAAAACACTTGGTTGTTTACCGCCGCACTA-CCCCATAACTCCATTTTACCGCTTC 34761
Gallid          TTCCT----CACTACATTATATACAAACATTTAAGTTCTACTAATGGAGTTTGTGATGTG 38657
                * * *    ****   ** * ***   *     *   * *  * *  * ***      *

Human           TCCA-ATTTCTGTATGCAGATATC--TCTAAGAGCATCTCCCCAGAT-CTAACCGCTTCA 34817
Gallid          TTAATATCTTTATATGAATGTAACAATTTAGGTGAGGCGTTCCAGGTACCAGTTTCTACG 38717
                *  * ** * * **** *  ** *  * ** * *   *   **** * * *    ** *

Human           CCG------TTCCCTGCGCCAAGGGCCGGACAAGTGGAAGGATAGATTTCACCCCGGACG 34871
Gallid          CGAGGAAAATGTTCTATCGTAATCAACAAA-GGCTTGTTGAATGGAGCTTTCCAACAAAA 38776
                *        *   **     **    *  *    * *  * ** **  *  **    *

Human           AACGTTAAGACGCATAAGAAAACAAAGCAGATCATCATG--CTGCGTTTTCCTTCTCTTT 34929
Gallid          ATGGTTAAGTGATATAATATGTGATTGGTGG-CATTATGGACGAAACAATTTAAGGGGGG 38835
                *  ******    **** *    *  *  *  *** ***  *       *

Human           TGCGATCACTA----GCGCCCACTAACTCCAGCGATGCAATCTTTAATATAAGATCGCCG 34985
Gallid          AGGAATGTTTATTTCACACATACCAAAAATGAAAATAAAAAATATAATGCAAACAGAACT 38895
```

```
                    *  **   **      * *  ** **         **  **  * ****  **      *

Human           TCATACCAC--AGCGTCGAGGGGT---------TACGCCCACATATTAAACAAAGGATCC 35034
Gallid          TTATGTCATTTATTATTAATAAACATAAACCTATACGACTCAATATCGAACGTAAAATCA 38955
                * **  **    *    *   *              **** *    ****  ***  *  ***

Human           ---------AAAGCAAACCAGCAAGAACAAAAAACCACAAC-----AGCAAAAAGGTAAA 35080
Gallid          TCTCTGTTAACATTGTCCCATTTAGAAGACAGAACATTGATTAATAATCTAGAAAATGCA 39015
                         * *     ***   **** * * ***     *      * * * **  *  *

Human           CCGAGATGCAGTAATAAACAAAGCGCTCATAGCGGGGTAAAAAGCGTCTTCTTTCTTTTC 35140
Gallid          GCCAGTATTGGTTTCATATAATCGATACAATCTATGGCAGGAAGGATTATTTCTCCTTCA 39075
                 * **     **   * * **      **      ** *  ***  *  * * ** **

Human           GGCAGA----ATGCTACAGAAATGAAGCAATCACCTCTCTCTGTAGTTTTCCTAG---CG 35193
Gallid          AAAAGAGAGCATGTAACAGAAACTATTTTTCCATCTGCTGTCACAGTTTGGACAGTCTCG 39135
                   ***    ***  *******  *       ** **        *****    **   **

Human           TAATCCACTACATACGTTTTAGTTCTAATTGACTCCATCACCGATTGA-------TTTAG 35246
Gallid          CGATTGAAACCACTGGTATCAAATCTAGCTGCTGCCTTCATTCCTAGAGCAACAATGTAT 39195
                  **  *   **   ** * *  ****  **   ** ***    * **       * **

Human           TAATTGTAACTGCACA-----------CCGCTCGGAAAGGAAACATTAC-------ACGT 35288
Gallid          TGAGTGTCATCATACAATTCTGTAGGACAGCTCCAAAAGGACACATCACTGCGGTGATCA 39255
                * * *** *    ***           * ****  ****** **** **        *

Human           AGTATCATTG--------TCCACAAACACAACACCAATATTTTTATTGTATCGACATAAT 35340
Gallid          AACGCTATGGAGAGCAGCTCTTCGAACACTTCATGGGT-TTTTTCCCGAACCGTTTGAAG 39314
                *     ** *        **  * *****  **    * *****   * * **    **

Human           ATCTTAGACCCCATGGCAGTCTCGATAACGCATCGAGGGTTAAATAAAAAGGCTATATCT 35400
Gallid          AGATTCGTCAGTATCATTGCTTATGCATATTTTTTCCAGAAGAGTAGAAAGACCATGTTT 39374
                *  ** * *   **     *  *     *     *     *   * ** **** * ** * *

Human           CTTTCGAAATAAGCATTAATAGGCACACTAGCCATCCGCCACCAAGCAGTTCCACTTCTC 35460
Gallid          --TGCATAACTTGCAAATCTAGGTTGGTTAGGAAACC-CTACAAAATCCTTATGATGATT 39431
                  * *  **   ***    ****     ***  * ** * ** **    **    *  *

Human           TGAGCCATTTTT--ACAAAAATCAACCAATGTCTTA--ACTCCGTTTCACTATGTGTTCC 35516
Gallid          GGATTTATTCCACAGCCAGTAACAGTTGGCATTCCATGACATTGCATGTGTATAAATGCC 39491
                 **   ***      * *  * **       *   *  **   *  *   ***   * **

Human           GATAAAAACTGAATTTT------CATTGTTAATAATATGCAAAGACGCTATCAC--CGAC 35568
Gallid          TTCCAAACGCAGATCTAGTAGAATTTCGTCGGGAAACTGCAGCCCTATTGTCGCGGCGAA 39551
                    ***     ** *         * **    **  ****       * ** *  ***

Human           ACGTATAATACACTCACC---CATGCTAAACTCCAACCCTGAGGCACCATAATCACTAGT 35625
Gallid          ATTATTAGCGCTCTCAGCACACGCGTTCCGCAATATATTAAAGTCGCGCTCATCATCCAT 39611
                *    **   * **** *   *  * *   *   *      ** * *  * ****    *

Human           AACAATCTATGGGAGCCTCATCTGCAAGTCTTTGCATCCCTCCTGATTTGCACATCCGGT 35685
Gallid          TGGCGGACGTGGGACATTTTTTTGATTTGTCCATGAATGTGCCCCACATCCCGTCTTTGAT 39671
                         *****   *  *      ***  ** **    **     *  *   *  * *

Human           TTATTCTCTTCATCCCAAAGCAAAGACCTCTGC--TTGTCTT---TCTTATACCAAT--- 35737
Gallid          GTAAGTACAGGGAGCAAACATAAGAGAAGATGCGGTAGCCTCAACCTCAATACTAATATG 39731
                 **     *      * **   **        ***  * * **         **** ***

Human           -TATACAAAATAACTATCACAATTATGAATGCCCCGACAAAACTAATCCCGCAAA--TCC 35794
Gallid          ATTCCCAATGGAGATGATAGATTGATATACATCTTCATTAAGTTTTTAAGCAAACCTCC 39791
                 *   ***   *  *   * * * **  *   *   *  **  *  *   *****  ***

Human           AAATTACTAACGAGCTTAGAAAATCTTTAAAATACTCTTCATCTCCCTCATTTGTATCTG 35854
Gallid          AAATTCTAAGATAGGCCGTTTGAGCCATGG----TTTTTCGCGTTTTAACCTTGCCTTCA 39847
                *****   *   **         * *  *        * ***   *        ***  *

Human           TTAACTCAGGGACTTGAACA---GTATCAGTTTCCCAAAGAGCATGAACCGTCTTTTTTG 35911
Gallid          CTACATCATGTAATCGAGAATGGGTGGGAAAGAGGCCAAGATATAAAGTTGCAAAAAATG 39907
```

```
                     **  *** * * * **  *   **   *      * ****     *   *       **

Human               GTGTGGATTTCG--TCGGCAAACCCTGAGGCGTCTTTTCAGA-CACATCGGAAAGTGTCG 35968
Gallid              CACTGAAGTCAAATTTGGCGATCGCTGCATTTTCTATTAGGGTCGGATCGCGTAAAATAC 39967
                       ** * *      * *** * * ***      *** **  *  *  ****    *   *

Human               ACGCTAAGATTACTGTGAAAAACGTCTCAA-TATCCTGCGCCTCATCACCCTTGACCGTT 36027
Gallid              AAGTTATTCGTCCTGTTGTCCATTCATCGGCTGCTTTGTGTCTGATATCCGTAGAATGT- 40026
                    * * **    * ****      *     **    *     ** * ** **  ** * **  **

Human               GGAAACGAACTTGTTGTAACATTTACATTATTGCCACTGGATCGATTTGTGGTTCCCACG 36087
Gallid              ACAGATAAAAT--GTGGGACGTGTCTATCATGGGGAACGGATGGTTTTCGGGAGACCACG 40084
                      * *  ** *   **  ** * *  ** ** *  *  **** * ***  **   *****

Human               GCTAGCGTAGAGGTCACCATAGAAGACTGGTTTAAATATGCCACAGAAGCTATTGATATT 36147
Gallid              GAGCTAATAGATGAAATGTTCTAGGCCCAGCT--AATG-GCCACAG--GCCGTCCGTATC 40139
                    *      **** *  *    *  * * *  * *  ***  *******  **  *   ***

Human               TCGGTAACATTTTTACCCCACGTTTCGGGATTCGCCGAAAACGTAGTTTCAGATTTCCCA 36207
Gallid              TACATAG-GCATCTATCGCATCTTTTATAGCT-GTTTGATTTGCGGTGGAGGATTTCACA 40197
                    *   **     * ** * **  ***       * *     *   *  **    ****** **

Human               GTTAAATAGTC-----GCTGGATG------------TCGATACATTCGTTTCCA----TA 36246
Gallid              ATTGGCCAGCCTGGAAACCCGATGATACAACTATAGTCTACATCTTCACCTCGGGGATTA 40257
                     **    ** *      *  ****            ** * *  ***    **       **

Human               TTTTTAGACGCAATTGAGGCAAATTCAGTTGCCGAAACTTCTGTAG--TCGTTAGGTTGA 36304
Gallid              GTTTCAGGACCGATGTACATTAATATAGGTACCAAAATATTTGTCGATTCGTTATCACAA 40317
                     *** **   * **  *     ***  ** * ** ***  * *** *  ******      *

Human               GCATTTGCACCTCTTCACAGAGACACACGAGTAAAAAAGACAAGAACAAC--ATGTTTTT 36362
Gallid              GAGTATAGTTTCTCATGCCATTTTGCATAAGTAGCTAATAGAGCAACAAGCGAAAATTCG 40377
                    *  * *           *       **  ****   ** * *  *****   *   **

Human               GCGAT-----TACCCAA---GTGCAACGGTTTTTTAATCACACCCA-CATCAAGATAGCA 36413
Gallid              GCGGGGGTTGTAACTAAACAGTCAAAAGACGTGGGGACGAGAGACAATGCTCTTACCGGA 40437
                    ***       ** * **   **  ** *    *     *  * *  **         *  * *

Human               GAATGAAAACAATCTTTTAGAACAATGTTCCGCATTAGTTTTTATAATACAACTCGACGA 36473
Gallid              GGCTGTAAATGGTCTAAAACCAACGTATTGTCCACAACTTTAAATATTGTATTTGAATTG 40497
                    *  ** ***   ***   *  *   * **   **  * ***  *** *  *  *  *

Human               AT-TCAAATTAAAAACACAAATGCCGATCATAAGAAAACACGATTTTGCATTAAATTCAG 36532
Gallid              TTGTCACATTTAAATTTCTTATATGGGCTGTTCACTTTTTTATATTCACGAAGAAATCGT 40557
                     * *** *** ***   *  **   *    *              **  *    ** **

Human               ACTTCCTCA---TACATTCAGATTTGTCATGTTAGTTTAATAGTCGATGCCTGCTATTCC 36589
Gallid              TCTTCTTTTGTCTGTATTTCAATTC-TCATGTCA----AACTCTGGAATTTGACGGGTAC 40612
                     **** *     *  ***   ***  ****** *    **   * **       *   * *

Human               TTTTAAACAATAGAGATTTTTCAACGTTCATGTTTCCGTTCCACATCGGGTCAGCAAATG 36649
Gallid              GTTTAAGGGCTGACAGTTTAATGTGGGCCAGGGGTGCACCCTCCGCATGCTTCATGGCAG 40672
                     *****    *     ***      *  ** *  * *   *  *    * *         *

Human               CTTCTAAGACAGGTAATTCATTTTTTTCGACGATTCACATTGAAAACGTAAATAACAAAA 36709
Gallid              TCGAGATAAGACATGACTCTTCAACGCTGATCATTCCGTCCGTTTTCAATAAT--CCGGG 40730
                         *  * *  * * ** *        **  ****      *    *   ***   *

Human               GCAATAAACAAAATCATTGCGATAGCCAAGCCCGTCAAAACTAAAAAAGCAAACGT--CT 36767
Gallid              GCGAGGGATAGATCTTTCTCCTGATACCGGTTCGCAAGCTGTAACTAACCCAGTTTGACT 40790
                    ** *   * * *    *  *   *  *  *  **  *    ***  ** * *   *  **

Human               TTCGTCTCTTGTTCCACAAATTCCACGCTGAGCGAGATGACTGCGGAGGCGG--TAAGCA 36825
Gallid              GTCGATATAGATGGTTACAGGTGTATAATAAGCATGATATCCCAGACGGTGTCTTAATTT 40850
                     ***       *      *  *  *   * ***  ***  *   *  ** *   ***

Human               TTCAACAAACGATGT-ATCATTGCTAGCGTGCGGAGAAACCTGTCCGTA---CATGACGT 36881
Gallid              TGCTAGAGTCGTAGTGAGTAACGCTTTCCATAGCATGTGTTGGTCCGAAGGGCAAGATGG 40910
```

```
                * * * *  **  ** *  *  ***  *    * *       ***** *   ** ** *

Human           CCATCATCAGCACCTCTGAATAGGACGGCGGCGGCGTCCGAGGGCGATCCATCCTCTTCC 36941
Gallid          CCATAATC--CGGTTACAGATGCGGAATTGGTCATACCTGCTAACATTGTATCAGTAATC 40968
                **** ***  *   *    **  *     **      * *    *  *  ***      *

Human           CGACGAAGATTCACGTTGAAATACACGGCGAGGACAAAATTTCTCCAAACGGACT----T 36997
Gallid          ATATCTGGGTTCAGGCCCCAAAGTTTAGCCATGG----ATTTTCCGGAGAGGTTTACTGT 41024
                  *    * **** *    **      ** * *     ****  *  *  **  *    *

Human           AAATTACTAAGATAATCGGAGTAGACGAGAAATGCGAGCACAAATGCGAC-TACGAGAGT 37056
Gallid          AGAATATTGTGCCGAGTATTGACCGCATGTATTTCGAAAAAGTAGATAGCATAATGCAGT 41084
                * * ** *  *   *     *    *  * * * ***  *   *     * **    ***

Human           GTATGTTCCTTCCCAGAGTTCCTCGGCAC---TTCTAG--------GAATGGGGAATAGA 37105
Gallid          GAGTCCTTCGGTACGATTTTTATTAGTCCAAATTCTATACACAGTCGTATAACAAATACA 41144
                *  *  * *    *    **  *  *  *   *****         * **    **** *

Human           GTTTTGC-TTATAAGCAACATGGTAA-----AACAATACAACC-----TACCCTCTTTCA 37154
Gallid          TCCTCGCATTATCTGCATCATATTAGTTTTTGACGATGCTGCCATCGTTGATATGGTTTG 41204
                   * ** ****  *** ***  **       ** ** *  **     *    *  **

Human           ATACAATTAATTCCCCTGACTAGAGACG-TACATATCTTTAATAATCCTCGATCCCGGCG 37213
Gallid          ACGCATTCGGTTCCAATAATTATAAACGATGAAAATCTACAGCACCTTCTACGCGTGGCC 41264
                *  ** *   ****  * * ** * *** *  * ****  *  *         *  ***

Human           CGACCTCTGACAGTGCTCACGGAGAGGACAGAGGACGTCTCTAGAAAA-CATCTCATCCA 37272
Gallid          AGAGTGCTGACGGAATTTCCGCCAAC-ACAGATTCCAAATCTACCAGATCATGATATATA 41323
                 **   ***** *   *  **   *  *****   *   ****  * * ***   **  *

Human           CCTG---CTTAATATAGTCCGTATCTTG------CGTAAACCATTGCGTATGCAAC-CGA 37322
Gallid          CCCAACTCTTTGTCTAAACGAGATCTTAATAGGTCGTACCACTTCGGGCGTCGAATATCA 41383
                **     ***  * **  *   *****       ****   * * * *  *  **    *

Human           CGATTCTCATTATAACGCAGACATCCAATTTTTA--AAAAATCTG-ACATAT---TGCGA 37376
Gallid          TAGCGCATATTGCGAAATAATGACTTGCTTTTCATCAAGAGGGTGTAGAGATCCTTGTGA 41443
                     *  ***   *   *   *     **** *  ** *   ** * * **   ** **

Human           GCTATAAAGAGAAGGCTCTC--CTCGCGGTCGACGCAAAAAAGCAT--ACCGTGGCCGTC 37432
Gallid          GTTTTGCATGACGTTCTATCAATACCCGCTCCGTCTAACAGAGCTTCTATCTCCGCCTTC 41503
                * * *  *       ** **    * ** **     ** * *** *  * *   *** **

Human           AAACACTACTACGAGCTCCCTCCAGTCACCGAAAAAATTATGCTGTACAATAAACACTAC 37492
Gallid          CGTAAATTCTTTACTTTTGTTCTACCCGGAAATGTTGTTGTACTCTTTGTGAGGCGG-AG 41562
                    * * **      *   ** *  *    *     ** * ** *     *  *   *

Human           ATCTAGTAGACTCGTCTGCAGTTTCCTCCGCCACTGAATCAAATGTTTACTAGAAAAC-- 37550
Gallid          TCCGAAGAGAATCTCCCATGTGACTTTAAATAAAGACACTGCATGGCTCTGAGTGGATGG 41622
                  * *  *** **  *          *     *    *    ***  *   **   *

Human           CCAGTCATGCTCAGA-TTGCAAAAGCGAACAAAGG-------TCTTGTACGGATACGT-- 37600
Gallid          TTGACTATGGTGAGAATTTTGGATCCCACTCAAGGCACATTTTGTTGCTCGCACAGGGCG 41682
                      *** * *** **    *  * *   ****       * ***  ** * * *

Human           TTTCAAGGTAAAAAAAGCATTCTTTCTTTCATTTCGGTAATGTAAGAGCTCAGAAAACGA 37660
Gallid          TTTTAAGGCAGTTTGGGCGTCAATCAAGTAACATCGCGCAATTTCCAGCACAGAACGAGC 41742
                *** **** *      ** *   *    * *  ***   *  *   *** *****   *

Human           TTTACTGTGAGCAATCATATCCAGTGCCACAGTACTGTCCAGTGCATAATCACACGTCTC 37720
Gallid          ATAAATATCA-TAGTCGTCTTTCATGACGTGCAACAGTTTTGT-TGGATCTATATCAGCA 41800
                 * * * * *  * ** * *    ** *     ** **   **    *   * *

Human           ATAAATCGGATAAAAAAACCGCAAACCATTAGAAAACAAATCGTGAAAAGAAGAAGCCAC 37780
Gallid          TTGGGTCCTTCTAAAGGGTTGATGCCCAAT--AATTCGGCCCATTTAC--AACATACCCT 41856
                 *   **     ***     *    *** *  **  *    * *  *   ** *  **

Human           GTGATAAAGACTTCTGTCAAAAAAACCATTATACACATAGATGTGTCCCCGCGCCCCCTG 37840
Gallid          GTATAAGTCCCATCTTCCAGATGTATTAAAAT-CGCATGTCAGTATCGTTGTATCTA--A 41913
```

```
                ** *     * ***  ** *   *  *  ** * ***     ** **    *   *

Human           AAGGACAATCGGTCCCCTGCAGGTTCTGGTTTTTGCCTTATGTATAACCCCGACCACAAC 37900
Gallid          GGTTCCACTTGGTCCAATTATAGGTATATTTCCAGATGCATA-ATAATCATATATACGAC 41972
                     ** * *****  *    * * *  **   *    **  **** *     ** **

Human           CAATTCGCCTTCGCAACACATATAGGTCTTCGCCCAGTTTTGAACATCTTCA-CGTGA-A 37958
Gallid          AAAAT-ACCGCTTCACTGTAAAGAGCCGCCGGGGTTGCACGACATGCTTCCAGCATAGTG 42031
                 ** *  **    **    * * **       *    *       *    * ** * *

Human           ATATCCGGAATGTTTTCTACACTATGTAGAGTCAACCGATAATCCTCGGGCCAGGGCAAA 38018
Gallid          ATGTTTATGAACTGTTCTCTAGTA-ATGGGTTCCGCTAATTTTGTTAGGAGTTCTGTGAG 42090
                ** *     *  * ****  * **  * *  **  *  **  *  * **      *  *

Human           GCTAAAGTGGCGCCCTGGCGATTTTCGATAAAATTTACCAACAAATCGATGTCTTTGCTT 38078
Gallid          ATCATTGGCGAGCACAGTAGGTCTTAGATA---TTTCTGAACAAAAC-ACGCTACCGCAT 42146
                   *  *  * ** * *  * * ** ****   ***   ****** * * *     ** *

Human           GAAGACAAACCCATAACGATGAACTGC-CCGGCTCAGAGCAAATAAACACAAATTTGAAA 38137
Gallid          CACTATCAA----TTACGTCGACCTCTACCGGTAAAGGGCAGGTAG-CATTTTTGCGCAG 42201
                 *  *  **    * ***  ** **   ****   ** ***  **  **    *  * *

Human           AACATTAATACTGCAAAAAATTTTATTGGTGTCCACCAAAACAAGCACGATAAATTGTCT 38197
Gallid          AGTTTTCATATCGCGTTGGGCAGTTATAGAATCTGCTCTAAGAATTACTAAAAAATCACA 42261
                *   ** ***  **          *  * *  **  *   ** **  ** * *** *  *

Human           AATCACACACCAAAATACATGCAACACACGACTAAACAAAAAAAGCCGTTCAATAAATCA 38257
Gallid          GAAGGCTTCAGCATTTAATTGGAGATCACAGCC--ATCGAAGCGCACGTGTATTAAATTT 42319
                 *   *      *  **  ** *   ***  *   *   **     ***  * *****

Human           CCCGCATAAAAAGATCTGTCTTTCAGATCGCTGCTCAAAGTCAACATAATCGAACTAAAC 38317
Gallid          GATTCAAGGAGAG---CATGTAGAATATTAATACTATCACTTACATTGTTTAGTGTACAT 42376
                    **   * **     * *   * **   * **   * * *   *  *     ** *

Human           CCGAGACCTAAAAAGGCTTCAAAGATAATTCG---ATATAGGCAGACGTTCATCATCAGT 38374
Gallid          C---GCTCAAATAAATGTTTGTACTTAAATCGTGGGAATATGTATA-ATGCATCAGCGGC 42432
                *   *  * ** **   **   *  *** ***     *** * * *  * ***** * *

Human           CCCGACAAGATCGC-CACCATTGCGCACGCAGCAGCTTTGAAA-ACATT--TAGTTTAAA 38430
Gallid          TCTGA-ACGTTGGTATACAATCTCTTCTGAAATTGTGACACAACATATTGGTAACTTGAG 42491
                 * ** * * * *   ** **  *    * *   *      ** * ***  **  ** *

Human           AAACACGTTGTTGACCACCGTCAGAAAAAAAAAGATGATTAGATTT--TGGCATACTCAAG 38488
Gallid          AAAAGCGC-GTTGGCCATCCTCCTCCATTGGCGATTACGTGATTTAGTAGCATGGGCGTA 42550
                ***  **  **** *** * **     *     *** *   *****  * ****    *

Human           AAGATTT-TTCCGATCCAGAGTAT----CGTAAATAATAACAAAAATTCGACCGTAAACT 38543
Gallid          AAAACCGGCTCCGACCCAGCTCCCGATGCATCGATATAAATTAGCACTTCATTA-AGACG 42609
                ** *     ***** ****          * *  ***  **  *  * *  *    * **

Human           TTACAAGGTATTTCAAATCAAC--TTCACGAAACGACGTTGACTTCAAA------ATCGG 38595
Gallid          CAGTGATCTAATTCGACCCAAAGATTGGTAAACCGATACCATTTCGGGACCATTTATCGT 42669
                     *  ** *** *  ***   **    ** ***       *    *      ****

Human           ACGAT--ATTTTTGCAAAC-TAATAAGAAATCCAGATGATCGTATCATCCATCCATATCT 38652
Gallid          AGGCTTGATGTATGCAAACATACTATGAAAATGGGA--ATCGTTAAAACTAAGACCAACG 42727
                * * *  ** * ******* ** ** ****    **  *****   * * *     * *

Human           CAGAGTA-AACGGCAGGAGAAACGCAGTCGTCGTCAAGACA-GAATAATCATCGA---AC 38707
Gallid          GTTACTACGGTAGTATAAATTACGACGCGGTAGTTAGACCATGAATTTATATCTATTGGC 42787
                   * **     * *  *   ***  *  ** ** *   ** ****    *** *    *

Human           ATTTGACTCTTAAAAAAATAAAGACACCATCATCCCCGTAAACACAGCGGGGGAATAAGAC 38767
Gallid          GTATTTCTTGTGGAATTTAACACTAAAACCGAATCCGTGTATGCGAGGCAGAAACGTGCC 42847
                 * *  **  *  **  ***    *  * *    ****  *  *   *  * **   * *

Human           GGCAT----AATCAGGACACAACGACTTAAAATCA-----TTCTCAACCTCATCTCGGAA 38818
Gallid          ACAATCTCTGAAAATGATATAGTTGAGGAAAACAAACAAATATTCAACCCCCCCTC--AA 42905
```

```
                   **     *  * ** * *       ****  *     *  ****** *  ***  **

Human          AAAATCAAGCAGATCATGACCACTGCAGACAAGAATGATGGCA-GTTTCGCCGCCATTAA 38877
Gallid         GCCGTCTAGTGAGTTCTGAAAAAAACGTCCGATCATGGAGACTTATATCCAAAGAACCGT 42965
                   ** **     *  ***  *    *    * *  ***  * *   * **       *

Human          AGACAGAAACACAATCTTGAAATCACCTATGGGTTTATAAAAGC-GTGGATCAAATTTTT 38936
Gallid         TGACGCCTGTACTCTGTTTAAATATAGGCTGAGTATGAGATGGAAGTTGTTTGAATTCAT 43025
                ***      **  * ** ****      ** ** *    *  *  ** * *  ****  *

Human          TAAAAGTCAACAAAAAGG-----------AAATCGCAACGGTTATCACAACGCCTATCTG 38985
Gallid         TCATCACCGAAAGAAGGATATTAGTTCCCAAACTACGGAGGATCGTACATGACCTTCTAG 43085
               * *    * * * ** *            ***   *    ** *    ***    ***    *

Human          TTCGAGGGAGGACCTCCCCTCTGTC-ATTCTAA-ACGGATACAGCCCTGGAATAACGGCA 39043
Gallid         AAAACCCGGATGCTGCATATTCACCTACGATAACATGTATATTTTCTTCACCTCTCATGA 43145
                      *    *  *    *     * *   *** * * ***     * *      *  *    *

Human          CCCTTAGCAAGACCGAGAATAAAACTATCTGTCAGACGACGCATCTCTGTC-TCTTAGGA 39102
Gallid         TGGCTAACATATCTATCAATTGAG-TATTTATAGTAGCGTCCATTGCTACAATCTTCGGA 43204
                   ** **    *     ***  *  *** * *    *      ***  **     **** ***

Human          TTGGAGCTGTATTCAAAATGCATGGAATCGTCGCTCTCGC-TATCTTCTTTCTCATTAAA 39161
Gallid         CGATATCTGAGAAGAGATGTCAATATATTATCGACCTGACATAAATGTTTCATAGTTGGA 43264
                   * ***      * *   **     **  ***  **  * **  *  **  *  **  *

Human          TAAGCTAG--AGCCCGCAGTCC---CGTTTTTAGTGGGAACCATGTAGTTGGTTATTTTA 39216
Gallid         GAGAAAAGCTGGCCGATGGTCGACATGATTTCATCCAATATAACGATGTCA--TAATTAT 43322
                *     **   ***     ***      * *** *       *  * *  **     ** **

Human          TATTGCGTTTCCTGTTTTGATGCCAACGCACTTCCGTCTTCGTTCTTATGATTCCTATCC 39276
Gallid         CAAGAAGTTTCGAATCTACGCGATGTAGAGATTCAATTT--GTACTAAGAGTCTAGAAAA 43380
                *     *****   * *     *      *   ***  * *  ** ** *    *     *

Human          CCTTTTCCGTCATCTTGTTTACCCATTCGTTCAAAGCGAGATTCCTCTTTAAGCTGCTCC 39336
Gallid         CTCTCTACCTCGCATAATATAATCGCTGGATGACAGATAGGTACAAAATCCTGACATGCC 43440
               *  * * * **    *  * **  *  * * * * **  ** * *     *    *      **

Human          TGAGTTAAAAATGCTTCCAC------CGTCAACAACG-TGCTTTTGGAAGTTAAGATCAA 39389
Gallid         AGCCCTATTAATTTTTCGGATAATGTATTGGTAAAACTGCGTCTACAAGACACAATCAA 43500
                *   **  ***  ** *           *     **    *** * *  ***  *  *****

Human          TTTACAGTCTTTAGCGGCTACCGTACGTAAACTACA-CGACGTCTTGATTACAGCACACA 39448
Gallid         AACGCTCATATTTG-AATTGCATAAAATACACTGCAACCAGTTCATGAGAGCAGTTGTCT 43559
                   *      ** *     * *   *  ** *** ** * *  ** ***    ***     *

Human          TACTTAAAGCCTGCTGTAAATTTTTGGCAGATAAATTAAA---TCGCATGTTCTTAACAT 39505
Gallid         TGCCAGATCCCATAGGTGCACGTACTATAAGGACATTGCGCGTTCTCGGGTACAAAGGTG 43619
               * *   *  **     **  *  *      *   * ***       ** *  ** *  *

Human          CGTAAAATATAACCTTATTGCTGTTTGAAAACTCCA--GTTCATTCATCTCTGTCACGAA 39563
Gallid         ACGGAAAAGTAACGGGACCGTCGGATTGACGCTCTGTTGTTATTCCCGGTCTGTTCTTGA 43679
                   ***  ****   *  *  *  *  *  ***     ***  * *   *****      *

Human          TTTTATAGTCGGGGGGGTTAACCTGTACTATAATGTGC--GCCATGAAAGTGTCAGATTTA 39621
Gallid         TAATCCAGTCTATAAGATCCTCTCCA-TATAACATTCTAGACAAGGAGGCGCTGGATGCA 43738
               *  *  ****      * *   **  * *****  * *  * ** * * * *   ***  *

Human          ----CCAGAGCGTTTAGTTTTAGTTACGGGCGAAAGCCATTTTAACACCTCGGAAACGGT 39677
Gallid         TAGTCTATCATTTTGAGTTGTCTCTGATTACTAACGTTACGGAAGAAAGTCGCCC-TGGC 43797
                   * *      ** **** *   *      * ** *  *     *  *  ***      **

Human          CGAATGATCAAGGT-CAATCCTCAACGGCGATTTCCCGCTGTCTCGCACCACTTCTTGTC 39736
Gallid         TGAGTGTCTATCGTATAACACTGATCAGAAAACTACCGGCGGTCGTCGCTATGGCCAGTC 43857
                ** **   *  **  **  ** * * *  *  * ***  *      * * *   *  ***

Human          ---CATGCACACAGGGAA----CCGACGCCTGTGTACATGTATCGGAAGCCGTCACCAAA 39789
Gallid         GAGCACGAATGGAGCGGAATTACAGAGGACTGAGT-CACATAGACTACGTGCACAAAAAG 43916
```

```
                   ** * *   ** * *    * ** * *** ** **  **    * *    **  **

Human           ACCCTCGTATACAAATCACTATCATGCTGAATGTACATCTTCGTAACCTCAGGA---TTC 39846
Gallid          ATGTGGGTAGTCCAAGCGGTGTGCTTCGGGATCGCCGTACTGGTATTCTTTGGAACACTT 43976
                *     ***  * ** *  * *  * * * * **   * *  * ***  **  ***    *

Human           GAGATGATGTTCATAAAACTTTCAAAGAGTGGAATATGATTATTAATGGTTTTCGTTAAA 39906
Gallid          GTTGCCGCGTCTATAAATTTAACTGAGGGTTTCCCGTGCTTCTTCGCAGC---CGTTGTA 44033
                *       **  *****  *  *  ** **      ** ** **    *    ****  *

Human           AAGTGCTCCGTATCTGATAAAAACAAACATTCCGCCTGAATGGTCAGCTTTTGCACGAGA 39966
Gallid          GATTATAGAACAGTTAATACGAC--ACTAGTGCACACGGGTC-TCACCTATCCCAGGCTG 44090
                 * *       *  * ***  *   *  * * * *  *  *  *** ** *  ** *

Human           TGATTTTTAGCAGACTGAATTATTATCGAAGGCTGCGGAGTGAAAGTCACTGTAGTATTC 40026
Gallid          GGGGGCGTGGTTC-CAGTATTATTCTTCCAGACAAAGGCGGTAGTTTTTTTCTTCTATGC 44149
                 *     * *    * * ****** *   ** *   ** *  *   *   * *  *** *

Human           TCTTTTAACAGTTTTGTAAACGCTTTAAGCGGTTTATTAATAGTTTT-CCAGCTTTTCAT 40085
Gallid          AACGAGTATTGTTTT-CGTGTTTCTCGTATGTTATATTACTGTTGGTGCCATTATATCGA 44208
                       *  *****         *     * * ***** *  *  * ***   * **

Human           ATGAAAAGCTAGGGTCGGCGGTTCTCTCAGTTCTCTATGTTCCCGATGATCACGGTGATC 40145
Gallid          GCAAGAAACATGTGGGTGCGGCATACATGGGGAGTGGTGCATTTG-TGTTTTCTCTTATG 44267
                   * ** *  * *   ****    *   *       **     * ** *  *  * **

Human           ACGATGATGATCTCGA--CTACCGCGCTCCATCTCTGTCAAGAACGAAGTCCTAGCACCA 40203
Gallid          GCGTCGCCTCTGACAATCCTATTGGGTACCGTTTCTATATGGC------TTCTACAAGCA 44321
                 **  *    *  * *  ***  * *  ** * *** *   *       * ***  * **

Human           ACTCGCGCCTTCTGTGCTTTAAAAACAAATGAAATGACCAACACATTATATACTGTAT-T 40262
Gallid          GTTGTTATAGTTCTTGCCCATAAGCTGATCGTACTAGCAGCTGCGGTTTATCTTGTCCAT 44381
                  *       *   ***    **    *  * * *  *     *  * ***  ***   *

Human           TTTTTGGCGCCAACATTTTATTACA--TCATTATAAAAT-GTTCTTTACGTGCATAACCA 40319
Gallid          TTTTCAACGATAACATTTTTTTATGGGTATTTCTGTGGTAGAGGTGTAGATAGTAAGGTC 44441
                ****   **  ******** ***    *  ** *    * *   * **  *    *

Human           CACCATCCGAGCATTTC-CCGATCAACGGTAACGG-----ATTTG-ATAAATCAA----- 40367
Gallid          TACGCTGAGGATATTTCATCTGCTAAGGATATCGACGGTAGTTTGCACAAATTAATTGGT 44501
                 **  *  *   *****  *    ** * ** **       **** * **** **

Human           GGTGTTTATACATCTCAGAACTACACAAAACC--TTATACATAGCG-ACTTTGAATCCAG 40424
Gallid          AATGTTCGAGCAATGATGGTGAACTTACTATCAATTGTGTATAGCATAATTTTGATCATG 44561
                  ****    **     *    **  *  * *  ** *  *****  * ***  ***  *

Human           TTTTATACAGCAACGGTAGCAAGTGTTTCAAACTCACAGTAT--CGTTGAAATAAAATAC 40482
Gallid          TCGT---CGCTAATGTTCG-GAATGCTCTTAGCAAATAGTTTTACATTAAAATTTTG-GC 44616
                *  *   *   ** * * *  * ** *   * *  * *** *  * ** ****      *

Human           ATTTGCCTGCCCCTGATCCAAAAAAGGCAACCC-----ACTCTTATACATCTCA-----A 40532
Gallid          ATGTAATTGTCACTGTTCTAATAACGACTTCCGTTTTAACCCTGATATATCTTCTTGTCA 44676
                ** *   ** * *** ** ** ** * *  **      ** ** *** ****       *

Human           TCAAATCATT---CAAAGGATAACTAAA-ACCATCTTTGAAA-ACAGCTAAATCATCCAA 40587
Gallid          TCGAGTTCTTAATCGCACGGTATGTACACATTATTTTGGGGGCATATATAGGTCTTCTAA 44736
                ** * *  **   *  * * **  ** * *  ** ** *    * *  **  ** ** **

Human           CTCCTTGTCCGTAAATAGATTCGTGTCCACGTTTAAAACCCTATATC--CCACAGTCTTC 40645
Gallid          TTGGGTATG-GTATGCTG-TGGACCACAACATGTGATTATGTAAATCGATTCTATTATGC 44794
                 *   * *  ***    * *      * ** * * *     ** ***      * * * *

Human           AAATATTGCTGATGCTTATTAAATCTGTTGAGCAACAGATTGTTATACACGGGAATATAT 40705
Gallid          AATGGGTGCAAATGC--AAGCAATTTGC-GTATTGCATGTCATAGCGTCCTAGCCGTCTT 44851
                **    ***  ****  *   *** **  *     **  *  *      *  *      *

Human           AACGATTTTAATTCTGACGAATAAACCGAAGTGACATCATTTAAACCGTCTTTTCCAATT 40765
Gallid          TACAGTGTTAATTTTATTAGCTATGGT-AGTTCGTCTTATTCGGGCCAGTTTATATCATC 44910
```

```
                 **  * ****** *      **     *  *    * ***    **   ** *   **

Human           ACGACTTTGATGTTTGACCGCCTGAGAATTTTATTGTCTTCCATCGGCCAGAACGATTCA 40825
Gallid          GTCGACGATCTACGCGAGCATATGCCAAAGCGATGAAGTTACAACAAAATGTAAAACACA 44970
                          *    ** *   **  **    **    ** ** *     * *  *  **

Human           GAAGAGTTGATGATATCGAATTCTTTCTGCATGGCAGTACCGGCAGTGAAATGAATATTG 40885
Gallid          GACTAA--GACAATTGAGACGGAGTTATAAACAGCGGGGTAGTCAATCCGAGGATGAACG 45028
                **  *   **  **   **     ** *  *  ** *    * ** *   * **  *  *

Human           CGAACTCCGTGCATCAGCACATCTTTCCTTAGCGTTGTCCACAATTCC----TCCGGAAG 40941
Gallid          TGCACT---TACACAATCCCGTTCTGCTGAAGCATCGGATGAGGATACAATATATGATCG 45085
                 * ***   * **  * * * *  * *   *** * *        * *    *  *   *

Human           CGTGCATTCGACATT----ATCGAAT----TGATCAAAGTATAACATCC--CCAGTGCG- 40990
Gallid          AGTCTATTCAGGATCTGAGAGCGAATGGGATGATTAGGAAAAACTGTGAAACCGATATAA 45145
                 **  ****   **     * *****    **** *    * *   *    **  *

Human           TATTTTGACCTGTCAAATAAATTGCATGGCTCTGCTCCTTTCATGCAACAATCCACACTC 41050
Gallid          TAAAATGAACCATAAGACTTAATGATTGTCTCATTCTTTATTAAACATCATAACATACTT 45205
                **   *** *  * * *   * **  ** ***      * * *  ** **   ** ***

Human           GTT-CGAACACAAGTATAATAAATGTGTTCACACATCATTCGA-----TATAATCGACAA 41104
Gallid          TTCATGATCGTTATCATATTCCCTATTTCTTTTTCCTCTACGGCCATTTTTTAGTAATAC 45265
                 *   ** *   *  *** *   * * *          * **      * * *   * *

Human           GCATCTGGATGTGCATAGGATAACCCGACGGTCATGAAAACGGAATGAAGACCCGTAATA 41164
Gallid          AAACTTGGGT-TGCATGAGAGGATCGGAACATC-TCAAATCATCCAAGTCGACCGTATCA 45323
                  *  *** * *****  **  * * **   ** * *** *           *****  *

Human           CAGATACCTAGACTTCGTCCATCTACAATTCCC--TCACGCAGAAAATCTTTATTCTCTA 41222
Gallid          TAATGTTCATCATCCAGTTCGATTACTTCTCCAGATTCGGTCGTCAGTGTGTTTTTCTTG 45383
                 *     *   *    ** *   ***   ***   *   *  *  * * * * **   *

Human           CAGCATAATC-AATTACA-GCATTAGCGACCAACACCATTTTTC----GAACCACCCGC- 41275
Gallid          CAACATCGTCTAAGTGTGTATGTTAAATAC-GACACTGCTTGGCCCATAAATGATCCACA 45442
                ** ***  ** ** *       ***   **  ****   **  *     **  * ** *

Human           ----CTTAATTCATTAAACATAAAAAATTTACACTGCAAACGCACAAAATCAATCTCCTC 41331
Gallid          AGTTCTCCAAATGCCCGATATCCAGATTCTTCTATCCCAACGTAGAAAGT-ATTATCTAT 45501
                    **  *        * **  * * * * *  * * **** * *** * * *   **

Human           GGTTAGCTCATCGCTTACTTCGTCACCCAAGAAATGCACCACATTAAGCAACATTCTCAA 41391
Gallid          GATCACAGGAGTGATGA-TTAGAATGACAGGGATTGCTTGTTCGGGAGTTAT-TTCATAA 45559
                * * *    *  * * * ** *     ** * * ***         **  *  ***  **

Human           ACAAGTGTTAATACCGTC-TCTGACAGGTAAAACATCCAAAAAATCACCCCCGCAATACA 41450
Gallid          ACGACTTCCGAAGCTTCGGTATCATCGACGAACAAT---GAAAAGCACCGACCCAATTC- 45615
                ** * *    *  *     * * *  *   **  **    **** ****  * **** *

Human           AAGGCACATGAGGAATCATAGAATACTGATATACGTTTTTTCGGAAAATAAGTCCCATTC 41510
Gallid          --GGTTTGTCGTCTATGGCGACCTATAAATGTAGCTAGAAATGGAACAATCTTTCTGTTT 45673
                  **    *     **       **   ** **  *      **** *    * *  **

Human           TGCCCCTTTTTAAACAACGGCAAATTTTTTCCACAAAACTAGCCATAGTAATTTTTGCAT 41570
Gallid          CCGAAGTATCCAGAAGCTACATAGGATTGAAGAAAAATCTGTTTAAAGTTTTGATGGCGG 45733
                      * *  * *        *   **    * *** **    * ***  *  * **

Human           G-ATCTGTTG-TCCGCTCAAACTCCAAATATTTTGTCGAGAA----AGTAACTTCGTCAC 41624
Gallid          GGATTTGCAAATATGGGTATACTCAAAACTCGTTCACCGGTATTCCATAAGAGTGGATAT 45793
                * ** **    *  *   * **** ***    **  *  * *    *  *   * *  *

Human           ACTTTCCCAGTTCAAAAGCTATATTTTTATGAAAAAGAGACCACTGCGCTC---TTTTTA 41681
Gallid          ATACTGTTAGTTTGGAGGTCGGGCTCCCCAAATAACCATACGCGGGAACTCATGTTCTTA 45853
                *   *   ****   * *      *      * **  * **    *  ***   ** ***

Human           GCTCGTACCTATCCATGAAAATCCCAGGAATGATCAAGCAAAATGTGACC-CGAGAAGTC 41740
Gallid          TTCAACGTCAAGTGATGCATCTCAAATTCATTA-CATTTAATTCTTGACTGTGCGGCATG 45912
```

```
                        * *   *** *  **  *   ** * **   **    ****   * *   *

Human           TCAGGTAGAACTTGATACAAGTCCAGCCACGTTAGTGCGGACACATGCCAGACTTCCACA 41800
Gallid          GTATGCAGAATGGCCTACTTTCCAATCATCATCAGAAGTAATTAATGCTTCCTTTGCCC- 45971
                  * * ****     ***    * * *  * * **     *   ****     ** * *

Human           TAGACTGTCAACTGAACCGTCTTATCAGTATCACGTTCCACCACCTGAATTTGAGACAGT 41860
Gallid          TAGGGTAATTATCGGGTCGAA--AGTAGTCT-ACACAGGGACAGTCGA-TTGTATATAAA 46027
                ***  *    *  *   **    *  *** * **       **   ** **  * * *

Human           AATATCTCAAAAATATTCTTGGCTTCCTGTTGATAACGAGTCACATTCAT-GCTTATATT 41919
Gallid          AACCTC-CGAAACCATTGCACTGTTTTCGTTGCCATCAA---ACGTTCGTAGCACTGGGA 46083
                **  ** * ***  ***      **   ****  * * *   ** *** * **

Human           TGAACCGTTTAACAAAGCCGGAAAAAGATGCTCGCCGTTTACTGTTATAAAATCCGCAAG 41979
Gallid          TAGTTCACTTCTCATTTCTGGACAAA-ATGCATATTTAAATCTACATTTTATTTCATATG 46142
                *    *  **  **   * *** *** ****          **    *  * * *  * *

Human           CG--AATATAAACAT--GTATTGTAAATCGCCA-CGTCGAAAACCG--TACTATTAGCCT 42032
Gallid          TTTCGATATTATTATCGGTAAGACAAGGCGCCAATATTCCATGTCTATTGCGGTTACACA 46202
                     **** *  **  ***    **  *****   *   *   *   * *  ***  *

Human           TTTCCACGAGGCCCAAATTTGTCATACAAGAAAACGGTAAAACCCATTTTTGAGTAAAAA 42092
Gallid          TCACCATATCAAGGGACGCTCCCA-ACATTCCAGTTCTAATATCTATCATCAGACCACAA 46261
                *  ***         *   *  ** ***    *    *** * * **  *      * **

Human           GGGAAAACGCCAATTCTTCGAATATCACGTAA--GGATTCTC---CTGATATATCTCCCG 42147
Gallid          GAATATTTCTCTATGTCTAAAATACCG-GTAGTTGTATTATCGAATTCGTTTTTCTTATC 46320
                *   *     * **   *  **** *  ***   * *** **    *  * * ***

Human           CAGATAACCATTTCTTAACACG--ATCTCTGTCAAAGTAGCACACATACGAAGATAGATG 42205
Gallid          AGAGTTGTCAATTGTTCGGACGGAAGATTGGCTGACACAGAGTGC-TGCGATGAGTGATT 46379
                    *   ** ** **   ***  *  *  *   *   **    * * *** **  ***

Human           CATGGAAT-ACTCTCTTCG-ACACCGTTTAAAGAATTGGAAATCT--CCTCTAAAAAGCG 42261
Gallid          TTGCCAATGATTCATTGCGTGTTCCAAATAGTATACTCTCAGTTTGTCCCCGCGATGGTA 46439
                     *** * **  * **    **   **    * *   * * *  ** *   *  *

Human           AGATGCACCCAAAACTCCCGGCATATATTGGTTTTTCCAACTCACACTTACTTTATAGAG 42321
Gallid          GGTCATTGGTAATAAGTCCAGC-CGTGTTGATTGTTCCATTGGGGCTCCACTTTACCGAT 46498
                 *        ** *   ** **   * *** ** *****          ******  **

Human           GACGCTACGAATAAAATCTCGATACTTCTTTACAAAGTTCAATACATTTGGCATAATCAC 42381
Gallid          GATGCTGTCAGCAAA----CATTGTCTAAATATGTGCCACAATTCACTGTTCGCTTGTAG 46554
                ** ***   *  ***    *  *   *   **       **** ** *   *      *

Human           ATTAGGGCATGATAAAATTAAATCGTCCGGACAAAAGTCGTAAAAAATACTCGTCTTTAG 42441
Gallid          TCTAGTTAACGATTCCACCATTTTGCACCAGCGAGAAGCATGAA----------TTGATG 46604
                  ***   * ***   *  *  * *  *   * * *  * * **           *   *

Human           ATACCATCTTCCTAGCATTCGATCCACTCGTGGAACGCAATGTGTAGCTCTCAAAACAGA 42501
Gallid          GTGACAAATTATTTATACGCGATTCATC--TAGGAGGATATGTTCAG-TCTCAAG-TATA 46660
                 *  **  **  *   *  **** **    * * * *  ****  ** ******   * *

Human           GATTATGGCATCCCGCAAACACCAAGCATCTAAACCTGAGTCGAGCCGATCCATTATCTT 42561
Gallid          TATGAATACTTATTTTCGAG--CAAACTTCCC--TCCGAAATGCCTTGTTTGATTACATC 46716
                 ** *   * *       *   *** * **     * **   *    * *  ****  *

Human           AGCGATGTCACGAACCCGTTCATACAAAACA-GAAGCGCATGAATATTGAGAATAACAAA 42620
Gallid          CGCTATGTAGAATAGCCGGTGATACAAAGGATGAAGCGAGTGTCTGCAATCATTTGGATC 46776
                 ** ****     * *** * *******  * ******  **  *      * *   *

Human           AACGTTTTTGCGACGCTTCGTAATGACAAACGCATTTTCTGTTGTAGCCAAAATCTTTAT 42680
Gallid          GAT-CTTCTGTAATATGTAGT--TGCCGAAGGTATAATTTGGTA----CAATATTTTCAC 46829
                 *   ** **  *    * **  ** * ** * **  * **  *     *** ** ** *

Human           -TAATACGACGCTCCTTTCTCTTCATCTTCAGACAGAAGGCTCAGAAGCTATACGAACAC 42739
Gallid          ATATTTCATCGTAAGGTTTTGTCTTAGTTGAGTTAGATTGTTCATCTCCTACAAGGATTT 46889
```

```
                 ** * *  **     ** * *     ** **  ***  * ***    *** * * *

Human           TTTTTTCTCAG------AAAGGTCAACGAATATGTTCTCTAGCTTTATCACCG------- 42786
Gallid          GTGAATCACAGTCGATCGTAATGCCCCTCTTTCGTTTTCGGGACTCATCTTTGCAAGATT 46949
                 *   ** ***        *   *  *   *  *** **  *  * ***   *

Human           -GCAGGGTAATTCCACGATAC-----TGCAGAAAACCCGATTTAATGTCCTCCGTTGCGC 42840
Gallid          TGGAGAGTGGAAACTTGGTTCCTAGTTCCATAACAACAAATCAGCCGTTCTT-GCTACTG 47008
                 * ** **     *  * * *     * ** ** * *  **     ** **  * * *

Human           AACGACCAATGCGCTGAACGCTTCCAAAGTAACTCAGCGTGGTATTGCTATTAATGAGAT 42900
Gallid          GGTTTGCGTGACAATAGAGAGCCGCAAGCT---CCAACAGGTTGTTTAAATAGGGAAAAA 47065
                      *    *  *  *      ***  *    ** *  * * **   **     * *

Human           AGTTCAAAAACAGAAACCGCTCCTTTT------CGAAATACTGTCGCAAGATATCAACTA 42954
Gallid          TGTTTAACGAATCAAATAGCTTTCTATGTGTGATGTCATACGCTTCCAAATATTTATCAA 47125
                 *** **  *   ***  ***   * *       *  ****  *  ***    * * * *

Human           CCAGGTCCTCGTTCTGAAAACTGGTCAGAACTACGTAGATCCCATACTGGAGCTTTGATA 43014
Gallid          ACTCGGCTCTGTGGCGCGAGCTGTGCAATTGCAGACGAATCTTTTCTGCCCGTTCCCATG 47185
                 *  * *   **   *  * ***  **     *     ***   *      * *   **

Human           AAACCTCTTT-------GTAAACATGAACCACGTAAACGTAC-TGGGTGGAATGCTGCCG 43066
Gallid          GGATGCCATTTTTACTAGTTAAGGGGGTCATTGGAAATAGATGCTGGTAGAGCATGCATC 47245
                  *   * **       ** **   *  *   * ***   *    *** **

Human           CGGTTCCCAGTCTGGCTCTAGGAAAATCT-CTTTTAATACCGTAAGCCCGTGAATGTTTT 43125
Gallid          TGTATGCTAGTATATCTAAAAGATAATCTGCCCTCATCAGTCTATGATAATAAAAGTGTA 47305
                 *  * *** *  **  * ** ***** *  * *  *   ** *    * ** ** *

Human           CTTTGTTAGCGTCATAGACATTTCCTTGCATGACG--CATGACAACAG--ACAACTGCTG 43181
Gallid          GTGGATTACTGGAAATCCCGAATCGGTGTGTAAAATTCATACCGGCTGCTAGAATCACTT 47365
                 *   ***  *  *    *   **  **  * *    ***  *  * *  * **   **

Human           CCGCAAGAAACTAAGCTCGT-CACACCAAACAAA-GACACTTTGTCTTCCAGGTACTTAC 43239
Gallid          CCATAAGCGATTGGCCCAATGCGTACATATCAATTGAAACTCCTTCGTC--ACTAGTCAA 47423
                **  ***  * *   *   * *  **  * ***  ** ***   ** **    ** * *

Human           ACGTGTTGATCATCGATAACACGTTATCAACCATTGAGTTTGTATACATGGTAGTTAAAG 43299
Gallid          TGGTGTCGAACGTCGTTCGAAACCTACTCCATTTAAGGCCTTTATCATCAGTTCACAGGG 47483
                  **** ** * *** *   *   **       *   *  * ***     **    *  *

Human           CGGTTCTCACGCAAGCAGACACATTGAAAGCGTTCGAACAAAGGAAAAACAGACCCACAA 43359
Gallid          CTGTGTTTGCCCATGTCCCAAAACTAAA-----TCGAACACGGGTTTAATATTTCCTCTA 47538
                * **  *  * ** *     * * * **     *******  **   ** *   ** * *

Human           ACAGAATTTTAGGGTTGTCCACATCAGTCTCAAAACGATACATCAACATT---CCCAAAT 43416
Gallid          -CAAACTTTTAA---TGACTGGATTT-TATCAGAACT-TATATTCACATCGAACCTGGAT 47592
                 ** * *****    ** *   **   * *** ***  ** **  ****    **   **

Human           TTTCGAGCTCCGGAGCCAATAATTCGCAAGGCGTGAAAACCCTTGCCGCTATACGTAAAT 43476
Gallid          TTCAGGATGGTAGAGTTTGTGTTAAGCAATGCTAGACTAAAATCACCAAT-TACAGCATC 47651
                **  *       ***    *  *  **** **  **  *   *  **  * ***   *

Human           TCTCCCTGCCGGACAAAAACTGTTTCAGAAATTTCCTTTCTT-TTTCTACAAC-ATTATT 43534
Gallid          TACTAAAATGGGATTAGGACCCTCGGTGACGTTAACAAAGATATTCCCACATTTGATATC 47711
                *         ***  *  **  *    **  **  *     * ** * ***     ***

Human           TAAGGTTCCCGCCTCGATAGATATATATTTTCTCTTCTTCACAGCTTCCACAGTATCCAC 43594
Gallid          CAAATGAGTCAACCCACAAGACACGT---TTAGAAACACAACTGCTTTGCCGAGATCCAT 47768
                 **      *  * *   *** *  *   **     *   ** ****   *   *****

Human           -AATGGCAGCTCGTGCACT---CGATTTAGAGTTCATTCTTAGTTCACTCAAAAACAGCA 43650
Gallid          GAACGTTTTCTCTATTGCTTTCCAATGTTGCACTGTTTTATCGATTCTTGCCAATCGATG 47828
                 ** *    ***     **   * ** * *   *  **  * * *   *   ** *

Human           CGTCCCCAGAATCATTACTAGCCGCTACGGCAAAAATCGAGATTTTATCCCTGGCAGCCG 43710
Gallid          ATAATATGAATCCATATCCATATGATATGCTGGAAATACTAGTTCTTTGGATGGTATCGA 47888
```

```
                         *  ***  * *   * ** *    ****     ** * *   *** * *

Human          ATAGCGTAACACACAACAGAGTAATAGCTTTTATTAA--TCGACTGCCCCCGAAAAA-AT 43767
Gallid         AAAGGCCAATA-GTGGAATGATATTATTTATTCTTAAAGTGGATTTCGCCCGTAATGCAC 47947
               * **   ** *      *   ** **  * ** ****  * ** * * **** **   *

Human          AT-CACT--TTGACCTTATCAGGGAATATACAGTTTTCTACTTTTTAAAC---TCTACGA 43821
Gallid         ATTCACCGGCTATCAATGTCATAAGTAACTCAGTTTTAAAACATTCCAATACTTTTTTGA 48007
               ** ***    *  *  * ***      *  *******  *   **  **    * *  **

Human          CCTTAACATCAGA------AAATAAATTGCTCTCCGCAGAACTTTTACACGAAGAACTCA 43875
Gallid         CGGCTATGTCCGTCTTCTTAAATATTTAACTACTCCGTAGCTGCCACTCCCAGCATATA 48067
               *    *  ** *       *****  **     *  *  * **   ** *  ** *   *

Human          GTCAAATTCGCTCT---TCGAGTTCGCCG------GGCACAGAACTTGAAAACTTAAATA 43926
Gallid         TTGGCATCTCTTTCAGATCGATATGGCTATAATGTAGCTCCGAAGTTACATTGAAAATAA 48127
                *   **    *     ****  * **          ** * *** **  *     **  *

Human          ATGCAG--AGGTCCTTTACGTGTTCGATCG--AATACTCAATTCCATTAAAATG--CTAA 43980
Gallid         ATGTAGTCACATTCTTTATACGCGCCAATGTAAATACATGTTCCGGTAAGGACGGTCTGG 48187
               *** **  *  * *****   *  * *  *  *****    * *  * *  * *  **

Human          AAAATGAGTTATCG------TCTCCAATCGGCAAACTTCGAGT-ACAACCCGCCGCCAAC 44033
Gallid         ATACAAGCTTGTCGCTCCTTTTTTTAGCCGGTGTTTTGCGCTTCATACACAGCTGTCTGA 48247
               * *     ** ***      * *  *  ***     * **  * * *  * ** * *

Human          GAC-CCAGGTACCGATAAAACGATAAAATACTCCAAGATTCAATCCATCATACAAAAGGT 44092
Gallid         GACATCGTCTAATATTGTCGGAGAAACGTCTTCTGTGGTGTATATTTCCGTATGTATGCC 48307
               ***  *   **    *         **  *  **   * *  *      * **   * *

Human          ACACAGCTTTACACGAGAAAATTCACTTCTTGCCAACTGCCGCGCCGTCGTAGAACTGAT 44152
Gallid         GTCGGGATGTATT----ATGCGTTCCCTGGAAGTACATGTTATAACTTCGT-GAGTTGAC 48362
                    * * **      *    *  * *      *  **      * **** **  ***

Human          AGACGATCTATATCGGAAACTT-TAC----TCATGGTTTCTACATATTCTCACTTTCGAG 44207
Gallid         GGTTGTCCCAAATCTGTTGGTTGTACGAGATCGTCTTTCCCAGTTATAAAAACGTTCCTC 48422
                *  *  * * *** *    ** ***    ** *  ** * *  ***    ** ***

Human          GACA-TTCAGTTTCCAGGCGACACATTTCTAGACAGGCTGTTGAAAATGGACTACTGTTT 44266
Gallid         GATACTTTTGTCTGGAGTCCGTGTGCTGTGGGAGCAATTGCTGTT--TTCGCCACCGTTG 48480
               ** * **  ** *  ** *       *    **     ** **    *   * ** ***

Human          TACATATTACCCTTCTTCAAATCGCCATCTAATAGATCTTTTCGAAAAGACGTTGGACAA 44326
Gallid         GTCGTTTTTTTGTTTTTTGATTCAGTATCCA-----TTTTGTCAGTAAAGCCTCGGATTC 48535
                 * * **    ** **  * **   *** *     * ** **   **  * * ***

Human          TCAAAC--ATCCACAGACATCGATAA----ATTTTTTGACACTTCAGGA--AACAGT--- 44375
Gallid         TTCATCTAATTCATCGGTGTTTGTAATGGAATCGTTAAGTGTTATATCATCAATGGTTTG 48595
               *  * *  ** **  *   *   ***    **  **      *  *  *  **  **

Human          --CCAGAA---CTACTTTATCAAAAAACGTTT----AGCTTGAAAATATTTTCAAAAAAT 44426
Gallid         TGCTAGAATGTCCTCCTTTTCGAGAATAGCTTCTTCGGTCTGATCTAATTGTAACTGAAG 48655
                 * ****   *  * ** ** * **  * **      *  ***    *** * *   **

Human          CTCACAGCCCAGGACAATGGATTATATATCTATCCACTACTTAAAACCGACCTGTCCATC 44486
Gallid         TTTGGGGTTCAG--CAAT--TTCCTATTTATATCAAGTATGAGACGGCGATCTAGCTG-- 48709
                *    *  ***  ****   *  *** * **** * **    *   *** **  *

Human          CTCGACTTCCTCGGAACCGAAAATATTCTCTTCCATCGAGGTCTCATCTA-TCACATACT 44545
Gallid         CTCATCTATATGACGCTGTATAATCTTTGTCTTTCTATCGGTCGCTTCGAGTCTCATGTT 48769
               ***  **   *        * *** **     *   *   **** * ** * ** ***  *

Human          TCATCAAAAAAACAATCCCTCAAGAACGGGAGAATGATCTCAATAAAATCAATCAATTTTT 44605
Gallid         TGAACGCAGCTGTGCTTTGTAGTCACTATGGGCGTTTCTAGCTGACGTGAACACTTCTAT 48829
               * * *  *             *   **     *     ***   * *  * **    * * *

Human          CGCTACTG--TCATCCAGCAGGTTATC--GAAACGAGAAGCTCATGCTTACCCGCGTCGT 44661
Gallid         AAATTCTGGATCATCAGTTTCTACACCTTGAGAC-AAAAGTTCGAGAGTCCGTTCTTTAT 48888
```

```
                    * ***  *****         * *  ** ** * *** **  *  * *   * *  *

Human           TATCCCGACTCTTAGATACGATTTTTCACT----TCAACAGAATCGGTTTAAACATAGAA 44717
Gallid          AAACA-GCTTCTCGTGTTCGGCATTCCGCTAAAATCCGTCGCCTCCGCCTGCGCATTGCG 48947
                 * *  *  ***    * **   ** * **    **    *  ** *  *   *** *

Human           ACG--TGCAGAATCTACGTCGA-GATCCTC--TCAAACCACATGGCGACACCAGAC--AC 44770
Gallid          CTCACTGCAAACATTATCTTGTCGATTCTTGGTCAAGTGTTGAGATGTTTGGTGGTTTAC 49007
                     **** *   **  * *  *** **   ****        *  *       *    **

Human           CCAACCCATAATCAATACATTCACCATCAAC--TTAATCCATATTGTTTTC-ACCGCGCA 44827
Gallid          TTGGCGAAGAGACGA-AGAGGCACTTCGAACGGCTAATGAAAACTAAGAACGATCGTTTA 49066
                    *  * *  * * * *  ***    ***   ****  * * *     * * **   *

Human           CGTGTTTTTCATCTGCATGGAAAATTTCAGCCCGACATTCTTATTCTATAATAGGAAAAA 44887
Gallid          GGCGCGAGTCATAGAAATGAACGGT--CAATACGAGACGGTGATATGGTTGACGCCCCCT 49124
                 * *    ****    *** *   *  **   *** *   * **     *     *

Human           ACTCATATTAGAACAACAGAGAGCCATTTTGATCATCGAACGA-AATGAATATTCCACAC 44946
Gallid          TTTTAAATTTTGCAATTCCGGTGCCCAGACGA-CATCAGACAGTGATGCCTGCCATAGGC 49183
                  * * ***      *     * ***     ** ****  **    ***  *      *  *

Human           TCTGGAAACAAATATCTGATCACATCGACTGTCTCTTCAACATCTCTCTCTCCGAATCCT 45006
Gallid          ATATTACATAACTGCTGCGATAGTTTGGGTATTTATTCTGCGA-TTACTACTCGTATGTT 49242
                     * * ** *        *  * *  * * * ***  *   *  **   ** **  *

Human           TTTTTAAAGAATATACCAAAGGAGGAAACGAAGAACATAAACA-GTTTCTATACAAAAAC 45065
Gallid          GTATAGCAGCAT-TGCTTGTAGTGAATTTGATGAACTACGTCGTGATTCGGTACCTAGAT 49301
                 * *   ** ** * *     * * *   ** ****     *  * ***  ***  * *

Human           -CTATTCGAAAAATGGGGTGACGTCTTTTTCCCTTTCACGTACTCGGTAACCACCTCAAA 45124
Gallid          GTTATCCACGTATTACAAATGCACAGGCTTTCCTATCTCCGA--TGATGATGCGTGTGGC 49359
                  *** *    * *       *      ** *** ** *  *   * * *

Human           AAACTCCACGGCACACCA----TATTACAACCCTTGAGCTAAGGGCCATCTGCAAAGAGG 45180
Gallid          GAATTCCATAATATTTCAAGAATATGACGAGATGGAATGTGCAGCCCATC-GCAATGCAT 49418
                 ** ****    *   **    *** ** *      *  *   * ***** **** *

Human           TCTAT--CAGAGCGATTCCCCGGAGGC--ATACGAATCGTTACTTCCCTACAGCACTCAT 45236
Gallid          ATTACAGTACAATGAATTCTTTTATATCAATGCGAACATCTGATGCATTTAAGCAGTTAA 49478
                  **    * *  ** * *    *     ** ****    *  *  *  *  **** * *

Human           CCAGCTTT-TAAAACATTATTC---ATTAAAATCTACGTTATACCTATGGTCACATACAT 45292
Gallid          CTGTTTTCATTAGTCGTTTTTCGAAATTATTAATCGCTTCGTTCCGAGATGTAAATAAAT 49538
                *    **  * *  * ** ***   ****  *    * *  * ** *     * *** **

Human           TAC-CAATCTTACCTTTGATAAAT-TACAATCTGATTACAGACTCATAACTCTGATCCAC 45350
Gallid          TAGATGATCACACAGTTAAAAAAACGTGCAA---GAATCGACGCGCCATCCTATGATAAAC 49595
                **    ***  **  ** * ***  * ***   ** *  *  * *    ** ****  **

Human           GCCTGTAAACTCTTACTCCCCTCGCAACATCTACTACTACACTACATGGTGTGGCTATAC 45410
Gallid          TTCACGGAACTCTCGAGCTCTTT-CAAAAAATGATATTGATGCATGCCACTTATTTTGTA 49654
                  *    ******    * * *  *** *  *  ** *      *       *   *

Human           GCTTTTTCCATAAACGTAGACCACATAGATC----TAGGCACTTTCACCGTAAT------ 45460
Gallid          ACTTCTGTTTTACTTGGAGATCACGCAGAGCGTGCCGAACGTTTGCTTCGTGTTGCCTTT 49714
                 *** *    **   * *** ***  *** *        *  ** *  ***   *

Human           -AAAGTCCGTAATCTTTAAAATCGCTGACCACATAAACGTGATGACGCA-TACGATCT-- 45516
Gallid          GATACCCCCCATTTCTCAGATATAGTTACTAGACATTTCCGTCAAAGGGCTACTGTTTTT 49774
                 * *  **  * *  * * *     * ** * * *     *   * *   ***  * *

Human           ---ACTCCCCTGAGACA-----AACCTTCTCGTAAGCATTCTACTAAACGCGTATACCGA 45568
Gallid          TTGGTTCCGAGGAGGCATGGAAAAACTTGGTTTTTG-GTTCCATTGATTGCTCTTGC--A 49831
                     ***   *** **      ** ***    *  *  *** * * *  **   * *  *

Human           CTACCTTCAAAAATACGTAAACCCCTGGATCAAACAGACCATAACGGCAAACTTCTCCCT 45628
Gallid          ATGTCTTCATTCGAAGGTATACGCATTGGATATACATCACATATCCGTAAAGCAATCGAA 49891
```

```
                 *  *****     * *** ** * * *   * ***   **** * * ***    **

Human           GTTACAGACGTACATAACCTTCACGAAACAATGCGCCAGCATCTTAGCGACCAAATGCAA 45688
Gallid          CCTGTATTTGAAGACATTGGGGACCGTCTTAGGCGATGGTTTGGTGCCCATCGCGTGGAT 49951
                  *  *   * * * *      **      * ***   *  *  *  * * *   ** *

Human           CATA--AACTTAGACAATCTATTTATCTCCATGACTATCGGAACAGACAAAATTGTAACC 45746
Gallid          CATGTCAAAGGAGAAACCATAACGTTTTCATTTCCCAGTGGATTGAAAAGTACAGTCACG 50011
                ***   **   *** *   **    * **  *  * *  ***    * *  *  ** **

Human           ACTTCGTTTTACTCCTTCATCGCTACATGCAGAA-ATTTAGTACGTCAACACGAAGAATT 45805
Gallid          TTTGCATCTAGCCATAACACGAACGTAAGTATCGCATCCTTCACAAACCTGTTTCGTATA 50071
                  * * * *  *     **       * * *    **     **           * **

Human           CAAAAA-ATCACTAAAAACGATCGAAACTTCCAAAACCGCACTCA-CGAATATGCTACTA 45863
Gallid          CATTCATACGACGTTGGGCTGTCTGTCATTACATAACCTTGTCAGTCGAATGCACCCTTG 50131
                **   * *  **      *  **     ** ** ****        *****   *   *

Human           AA-CATAATCACAAGCGTCTCCTCCTCCAAGGAG--CTGCTG--ACCAACGAAGCACTAC 45918
Gallid          GATCATTATCGCACTCCATATTTCGGGCAAACAGACCAAATGCCACCCACAAGAATTTAC 50191
                 * *** *** **  *      **   ***  **  *   **  *** ** *     ***

Human           AAAAATTCATCGACACTGTCCAACGCATCTCCCAACACGTAAACGAAACATACCAGTTAA 45978
Gallid          AGGAAT--ATTACTGTAACCTACCCCGGCCACTATTACATCTTCGAGCCTCTCTG--TAA 50247
                *  ***  **         * * * *  *  * *  ** *   ***  *   *    ***

Human           TTTCCGTGAACCTCGAAAAATGTAAAATC--TCAAACGACATCCTAATCGAATCCCTAAA 46036
Gallid          TGCGTGTTTCTGTGGAGGGATTTGTTATCAGTCGGACAGAGT--TAACCATGTCCATGAA 50305
                *    **     * **   ** *   ***  **  **    *  *** *   *** * **

Human           GAAGACCATCTCCATAGTTGATGTACTCAGCTCCGATGCAATCC--TAAACACGTCGTTA 46094
Gallid          TA-GAACGCAACCGAGGCTCTTATCACCTTCCACCAAGAGATCCCTTTGACGGGCTTTTA 50364
                 * ** *    **   * *  * *   *  *  * * *  ****  *  **  *   ***

Human           ACTTC-TAGATG--TCTGGAGGCCGCCACGCTCGCGGTTTCAAACAATTCTTTCACAATA 46151
Gallid          TCCACATACATGGTCCTCGACAGAGACATGTATGTCCAAATGAACCACATTCCCATAAAA 50424
                 *  * ** ***   ** **    * ** *   *        *** *   *  ** ** *

Human           CTCGAAATTAAAAAGGACGCAG-TTGCCGTTTTCAAGCCTTTTATAACACAACTATTTGA 46210
Gallid          CATTATATGCGTCTATGTGTAGATCACAATGTTCACGGCATTCACGACATCCACTCTCGT 50484
                *   * **          * ** *  *  * **** * * ** *  ***       * *

Human           AAGCATGAAACCCACCACGAGTCTATATAAGAAATTGATGGCTACCCAAAAACTGACCAC 46270
Gallid          GATTAATAAATTTTGCTAGAGT-TGCACTTAGGCTTCGTTGCTGCCAGCCAGCGGGACAT 50543
                 *  *  ***     *  **** *  *       **  * *** **    * * *  **

Human           CGACCGCATTCCATTTCTCGATATCTTCGACGATAGGTACAAC--CTAGTCAGACAC-GT 46327
Gallid          ATTGCCAGTAAACTCTCTTCCATTCTGAGATAATAGAACCTGCGTTTAGAGAAAGACCAC 50603
                    *   *    * ***     ***  **  ****   *  *   ***  * * **

Human           CGAACGACAATTAA-ACTGGTACGCCGCATATGCCGAAGCAGCGCAGCAAGATCTTATTG 46386
Gallid          CAAACTCCGATGGATATTGGTATGCAGT---TGTGGGGATCAT-CAGGTGGCTGTGTACG 50659
                * ***  * **  * * ***** ** *    **  *        ***   * * *    *

Human           CTCCTCTTACGTTTTAACGGTCGCGTTCTAACCCATGAAAATCATTACCAGTAGCACGAA 46446
Gallid          TTCAACAAAAACATCGATG-TCAAGTTCTA-TGCACGGTG-TCGTCGGTAAAAACTC--A 50714
                 **  *  *    *  * * **  ******   ** *    ** *    *  * * *  *

Human           CCAAAATGACTCTAAATACGGACCGAGAGC-CGGAAAACAATGCATGTCGAACAGCTTTT 46505
Gallid          CTAGTCACATCAGGGATAATGGCATATGCCTCAGTAGACGAGACAT-TGGAAAAAAATAT 50773
                * *     *      ***  * *  *   * * * * ** *  *** * *** *   * *

Human           CATTTCTCCACACTGTTTACTTAAACGGAATTAACAATTCTCTCAATGCGGGTACTATAG 46565
Gallid          GACATCTGCAGAA-GATCCTTCATGTAGAATAGGGTGTATACGTAGTGTGGCAGTCACAG 50832
                 *  *** ** *  * *   * *    ****      *   *  * ** **     * **

Human           ACGCCATAATGGAAGAAG--GCTATCACTTAGACACGGCCAGCACCTTAGCTCTAATGCT 46623
Gallid          AATATGCACGTGTTCCATTTGCGAGGAACAAGATATGAAATTCTTTTCTACTCAAATAC- 50891
```

```
                              *      *   *    *   ** *  *   *** * *     *   *   *** *** *

Human                         AGATAACTCAGATTCGCAAGAC-TACAGAC-TCCTCACCGAAAT--CCCCAGAAGGATCC 46679
Gallid                        AAGTTCTTTGGTCGCGTGAGGTACATAAATATTTCCACCCATTTTGCTTTCCGCGGTTGC 50951
                              *  *   *  *   **  **    * * *  *   **** *  *  *       ** * *

Human                         ATTCTAGGTACGGCGT-CACACAGCATGAACTCTCACGGCCATTTAACGGAACCCT---A 46735
Gallid                        TTATTCGGTGTTACGTATGTACATAAGCTACTATCTAAGGCGATTAATGCAAGCATTACA 51011
                               *  * ***    ***    ***  *   *** **   * *  **** * ** * *   *

Human                         GACACACAGAAAATTGACAATGAGGTATATTTTGGCCTCATAGATTT-CATACTGTATGG 46794
Gallid                        ACCAAACGGGATTCGGCTCGCAGTGTACGCCATATGATGATACGTTCGTTTAATCTACGA 51071
                                ** ** * *    *        ***     *    ***    **    ** * ** *

Human                         CAAAACCAAGAATTGTCCAGCTTTTGCAGTCATCACGATCGGCGTGCTA-TCGCGAGCAA 46853
Gallid                        ACGAATTCGA---TATCCA--TCCCATCACAATCTCTTCCTGAGTGCTGGTGGCGTGTAG 51126
                                 **        * ****  *         *** *   * * ***** * *** * *

Human                         TTTTTTTTCTTAACAATACTCTCTACTTATTCGACTCTCA---CCCGACTGAACGAGAAG 46910
Gallid                        ATCTTGGTATCTTCAGT---CTCTGCGTAGTCATAATTCAATACCTGTGTTGAGGATTGG 51183
                               * **  * *   ** *   **** * ** **     ***   ** *  *  * **   *

Human                         CCACA-GCAGCCATCTACATCTGTCAAGACATTGAA---GAAGCTTATGAACTGCTCACC 46966
Gallid                        TCACATATACACCTCTGAAAC-GTAAGGTCGTAGCACCTGTTGCATCTTGTTGGTGCGAC 51242
                               ****   *  * ***  * * ** * * * * *   *  ** * *     *  *  *

Human                         GCCCACGGCACTGAAG-GCTTCTACTACGACGCCAGCTTCATTTT-TTTCATAGAAACCT 47024
Gallid                        AGAGACGTGAGAGAACTGCTTCAATTCGTGCGACAGGCCAATTAAATTCCGTGTGGGCAG 51302
                                  ***  *  ***  ***** * *    ** ***    ***   ** * *     *

Human                         CCAATTTGTCGCTCTCTAGT--CACGACGCCGAGCTTCTTAT---CCTAAAAACTTATAA 47079
Gallid                        TCAACATAAACCCCGCAGGCAATACAATAGCGATGTGCAAATGTTCATCATAACCCACAC 51362
                               ***  *    * * *  *    ** *   ***  * *  **   * * * ***  * *

Human                         AGACCCCGACATAGCGATTACGCTAGATAAATTTTCTTCCACGGAAATCCATGATATAAA 47139
Gallid                        ATATATAAGCATAACT-TTCTCCGGGAAGAAACTTCTCGCATGTGG-CGTGTGGTACAAA 51420
                              * *      **** *  **   *  **  **  ****  ** *        ** ** ***

Human                         AAAAACAGATGATAT-CGAATCGCAACAA---GACCTCGTTGCGGCTAAAACGACAGATC 47195
Gallid                        CCATTCAGGGAATGTACATGTCATGACAGACCGATTATATGGTTGCCTGTTCCATAAATC 51480
                                * ***   ** * *   **   ***    **     * *  **     * * * ***

Human                         TAGAACGCGCGCCCCAGAAAAGAAAGAAAAACTCTCATAGTCTAGAACTAGA-ACTAAAT 47254
Gallid                        CA--CCCCCCCGTTTAGAATTATGGGAACGGGTCT-ATTATTTAATTTTATACATTCACC 51537
                               *   * * *     ****      ***    *** **  * **    ** * * * *

Human                         GACAAGAAGAAAAAAGATACCGCATCCTTGACATATTACGCAACGGAAGTCGACCTGATT 47314
Gallid                        CCCTGTGCTATAATAATCGAAGAGTACAAGACCTGGTATTTTTGTGTTCACGAGCTGATA 51597
                                *      * ** *      *  * *  *** *  **       *    *** *****

Human                         CCGAGCTTTTATGAACTACGATCACAATTTCAATCTTTATTTCACGATCTCAAATCTTTT 47374
Gallid                        ATCACCTCCTAAATGTTCCG-CAGCATTTTCTGTTGTATATCTGCGATATG----CTTTC 51652
                                 * **  **     * **    ** ****  *  *   *   **** *     ****

Human                         CCTATAATGAAATCTCAATTCAACTGGACCATATACTTACAAGATTCTCCCATGAATCCT 47434
Gallid                        CCTCCAAATTTATTTATAT---ATCCGGCCATATAT----GAGATT--GCGGCGGATATT 51703
                              ***  **    ** *  **   *   * *******      *****   *   * **  *

Human                         AAT-CAGCCCTTCGCAACACCCTTTCTCTGGAACAGAGTTTTTCACTTATTGTGTCA-AA 47492
Gallid                        AACGCACCCGTACTAAACTTTTCCCATGAGGAATTTGAAATTGCATTAAGAGTGGCACGA 51763
                              **  ** ** * *  ***        *  ****       ** ** * *  *** **  *

Human                         TCATCGACGTGTTTGTAGGCGTCGGATCC----ACAAATGACGATTCTAGCAAACAAAAG 47548
Gallid                        ATATAATTAGGAGTACGATTGGTAATTTCTGTGATAAACGGGAGGTCGTATGCATGTATG 51823
                                **      *  *      *     * *    * *** *     **      *   * *

Human                         CAGCAAACGATTTTCATAAATTATTTA--TTGCCTTTCAAGGACTTTTCCGA-AGTATTC 47605
Gallid                        TGGTAACTGATCATTTGGTATAAATTGGGTTCCATTCTCAAGGCCTCGCCGATGGCACTT 51883
```

```
                           * **  ***  *     ** * **   ** * **   * * * *  ****  * * *

Human          A--ACGAGGCGTTGACAGCTTGTCAAGAAAACAATCTGGACATCCTCTTGATTTACAAT- 47662
Gallid         ATGACACAGTTTGAAATATGTGTTTTAAAATCGATCATTAGGTCAATTTCAGATCCAGCT 51943
               *  **   *  *  *     ***    *** * ***   *  **    ** *  * **

Human          --AATTACCTGTGCAAAACCACGACCTTTCGAACACTCGAGAGAATCTTACTAAGCAAAT 47720
Gallid         GAAAATGCTTGTAGCTGGTTGTGCTCTCTT-AATAATGGGTCCGATAA-AATAGGCGCTT 52001
                 ** * * ***           *  ** *  ** * * *     **   * ** **   *

Human          TTCTGGCAATAGCTGA---TAATGAGCACGAAAAACATTATGAATGGGTCAAATCGTGGA 47777
Gallid         GTCTAGCTATATCAATCTTTCTTTCTTGCGATAAATAACACGGATGATCTACACCTTCTT 52061
                *** ** *** *      *  *     *** *** *  * * ***    * * * *

Human          CCACACAAATGCT---CCAAGAGATGCC-AAAGAAACTAGATGACATAGAGAATTATCTA 47833
Gallid         CCTGACATGCGATAGACAGTAAAATGTTTAGAGATGTTGGATTTGGTGTAAGATGGGATG 52121
               **  ***   * *   *    * ***   * ***   * ***    *  *  **    *

Human          AAAG-CCTATGTGAGCCAGAACCCTGTGAAACACTTCCATG---AATTCGTTTGT----- 47884
Gallid         AACGTCCTCCCAAAGCTACGGAATTTTCATATGCTACAATATAAAATTGACTTTTAGTGG 52181
               ** * ***     *** *      * * * *  ** * **    ****   ** *

Human          CTGAATAAGGCAGAAAAACA--CAACATAGCTGTTCTGCTCAACGAAAAACGAAAAGAGA 47942
Gallid         TAGAATGGACCCGAGTTACAATTGATATACTTCCACATCTCCACAGATTGGCGAGTGTAA 52241
                 ****    * **   ***    * ***  *   *  *** **  *       *  *  *

Human          TTCAAG-----AAGATATTGAACGAGACAAGAATATTTTCGCACAACTCTCTAATTTTAT 47997
Gallid         TTCCATTTCCTAAGATGCCAATGGCTGTCAAAAAAAATCTCTTTGGAGTCTACAGGTTT-T 52300
               *** *      *****    *  *     * ** * * **     * ***  *  *** *

Human          TGACAAACTTGGCGAAACTCCCGCACTTCCTATCGAATCAGAAAACGTGCACAAAGTTCA 48057
Gallid         TGGGAAGGGGAATCGAACATGAGAACGTCTTCAAAATTCGTTGTGATCTCTTTTAATTGA 52360
               **  **         ***    * ** ** *    * **           *    * ** *

Human          CA-CGAACGACATCACTGAAGC---CATC-GTTCCGCGTTTCATGACTGAATCGATTGAA 48112
Gallid         TGGTTCATTGCACTGCTAAGACGTACATTAGTGTTGCCGTCTCCAACGAATCTAATTTTT 52420
                     *   **   ** *  *   ***  **   **  *     **  *    ***

Human          TTACCAAACATC--TCCACACTGAACAACACCCAACAATTATCCCTAGAAAAACAAATCA 48170
Gallid         CCAGCGGGTTCCAGTCCATTCATAACAGCCTTCTGCTGTGTATTCTTGCTTGGTAAATCA 52480
                 * *      *  ****  *  **** *   *  *  *     ** *      ******

Human          GTGAAAAACTC---ACCAACACCATCCACACACTCAGAAACAAATTCACAAAAATCGTAC 48227
Gallid         GTATTGTTTTTCAAACAAGCAATTGCTCCCTGCTTCAATCCAGAT--ACAACCCCCAGAG 52538
               **       *    ** * **    *  *   **   *  ** **  ****    *  *

Human          AAGATAACTATAACAATCTCGCTGCCGGTTTCATGCCAGTTACCGAACTGAATTGCCTAT 48287
Gallid         ATCCTTTCTCCAGTGAGCCGCTCACTGGTGCTCTTTTGGTAATAGC-TTCGGCCGCTTTC 52597
               *   *  **  *   * *      * ***    *    ** *  *   *     ** *

Human          TTGCCTACCTGGTAAACCTTTATTTTAACATTGAAGTTTTAAAACACAGCGGCCTAAACA 48347
Gallid         TTGATAAGTTGTACAATCTCTGTATTA---TCCGCCTCTATTCGCGCAGCTATATCAGGG 52654
               ***   *  **   ** ** * * ***   *     * *     * ****    * *

Human          TTAACACTGAGTTGCTTCAAGAAGTGGAAAAACTGTATGACAACACGCAATTTCTACGCT 48407
Gallid         GTATCA--AATTCATGTTTATTATTTGCTAAGAGGTGC-ATAATATCAAATTGTTTTGAC 52711
                ** **   * *    *  *  * * *  **   **   * ** *   ****  *  *

Human          TCGGAACTTCTCACTTCAATATAAACAACCTTTCGAACTT-TACCCTATCTATCAGAAAA 48466
Gallid         TGTGAATGTATTACATCATCAACGTTACCTATACATGCTTCTAATTTGGCTCCCAGTTCT 52771
               *  ***  * * ** ***  *     * *  * *   *** **   *  **  ***

Human          ATGTTCGTCGATTTCTATAACAGTCAAAAACCCTCGGACAGAGCTTCCGAAATCCTAGCC 48526
Gallid         ATAATAGTTACGGGCAATTCCGATTCAAATTTTTTTGT--ATACTGCAAAAATTGAAGAA 52829
               **  * **       * **  *  *  ***    *  *      ** *  ****   **

Human          GCAATCGAATCCATCTTAGCGGACCCCAGCAAAAACAAGACAATCGTAAATATAGAAATG 48586
Gallid         ATAATGTCTGTAATCGTTCCGTGTCGTTATTGTATCGA-------GTACCTATTGGAACT 52882
```

```
                          ***       *** *  **   *        * * *       ***  *** * **

Human           ATCAAATCACAACTGGAAGAACTGGGGAAAATGGAAATCTCCACCACCGAAAACAAGCAA 48646
Gallid          AACTCGTCTTG-----------TGTAAACAATATATCTTTGTAGCTCTTATAGTCGCCGG 52931
                * *   **              **   * ***  *  * *  * * *  * *     *

Human           ACAGCTGAAATCACAAAACAAATCCTTGGGGACCAAGAGTTAACCCCTATTTA--CGATT 48704
Gallid          T--GCGGATATCAGAACATGGAATTCGAAGCGCCACGGATGTAAACGGATCAAATCGATT 52989
                   ** ** **** ** *   *       *  *** *  *  *  *  **  *  *****

Human           TCCTGCACCATCTCTCTGCTTATAACCTACCTAATACTACGACCGTAAAAAATTTACACC 48764
Gallid          TTGCGAGTCA-CATTCAATAGGAAGACTAATAAATAAGGC-ACCCTGA-------TCGCT 53040
                *   *   ** *  **       *  ***   ****   * *** * *        * *

Human           TCCATTTCATCTTAGAAAAACGACCAGATATCGCTGC--GATTCTCCACGATAAAATACA 48822
Gallid          TTGATTAGATGTCGAAAAAAACTGCAGGAT-TCGTAACTTGGGGTACCAATAGAGAGTGTA 53099
                *  ***  ** *   ******  *  *** ***   *  *     ***  * * * *  *

Human           ATCCATACTTGACATATGTATAGACGACATGCTCAATGACATAACAGTCCCTGAGCAGAC 48882
Gallid          ATGC--ACCCGACGGA-ACATATTCTCCGTATACATTACTCCAAT-----TTGAGCATAG 53151
                ** *  **  ***  *   ***  *  * *   ** *     **       ****** *

Human           GTTTTCAACCGTTCTATTTCTTGTAGATCTCTTCCCAAACAG-CACCGAAAAAACAGCAC 48941
Gallid          GCCCGTAGC-ATTCTGTCGTATTTGAACGCCCACGGAAACAGATAGAGATGAGTAAACAC 53210
                *     * *  **** *    * *  *   *  *  ******  *  **  *   * ***

Human           TGTTCGAAT---CCGTTCTCACACTACGGCAATTAGCAAAG---AAATGCGCTAACT-TA 48994
Gallid          TGGTAGTATATCTTGGTGATGCATCTTTAAGATTGGTGAGATCTATTCCCGCTGCCGATA 53270
                ** * * **     * *    **        *** *  *     *    ****  *  **

Human           AAAACTCTAGAAGAATTCGACGATCTGGCCCAGTTCATCACGACGAACAGCGAACAACTC 49054
Gallid          AACATTCATCAGGTATTATTATATGTGTTAACAGATGTCGCATCGCCCCCCGATCAAAGT 53330
                ** * **   * * ***     ** **          ** *  **  *  *** ***

Human           CAAAACATGATGAAACAG---CATTTTGGAAAAAAAAATACCA------ACGCTGATGGAT 49105
Gallid          TAGATAATGAATATATAGTTTCGCTTTCTATATGCGCCTCCATACTGGACGTCGCTGTTA 53390
                 * *  ****  * * **   *  ***  * *       ***      ***  * **

Human           CATATCAAATTTCTC---TACTCTCAAAAAATAATCACCGCTGAAGAGAAAAAT--TGGA 49160
Gallid          TCCGTCGTGTTGCCTACGTGCACCAATGTTGTGACTAAGGTCTAGCTGTAGGATAATTAT 53450
                    **   ** *     * * *  *     * *  *  *   *   * *  **  *

Human           TACAGAGAGCAAAAACAGCCGTCATTACATCTCCCGAAGAACTGACAGCCTTCTTAGCCA 49220
Gallid          TATTAATGTTGAGGCTATGCCTACCTTCACCCTCCGATTCAATGTGATCTCTTTT--TTG 53508
                **   *     *    *  * *   * ** *  ****   * **  * *  * **

Human           CTGCACCCACTAAACACGCCTTACAAACTTGCAAGCCTGAGCTCGATAAAGCACTACAAC 49280
Gallid          TTGCGCAGAGCATTCGTGGACAAGATTTCAACTTACTATTTGTCGACGAAGCGAATTTTA 53568
                 *** *  *  *  *  *    * *      *   *      ****  ****

Human           GACATATGGA---AGAGCAAATGAA-ACAAACTGCTGAAAACGA--------CAAAAAAC 49328
Gallid          TACGTCCGGATGCAGTTCAGACGATCATAGGGTTTTTGAATCAAGCTACTTGCAAAATAA 53628
                 ** *  ***   **  ** * **  * *   *  *  ** * *        ***** *

Human           ACATCCTCACAATCAGAAACACCCTCGAAAAAAGACTGAACGACATCTTACTCATTCTAA 49388
Gallid          TTTTCGTTTCCTCTACGAACAG--TGGTAAGGCAAGCACAAGTTTTTATATGGTCTTAA 53686
                   ** *  *    *  ****   * * **    *    * *   * *   *  *  ***

Human           AAGA-CGGTCAATTCTCGTCTT----TGGAAACA-GTGCATTTGAATCTTTTAGAAACCT 49442
Gallid          AGGATCGGCTGATGATCTTCTTAATGTGGTAACTTATATATGTGACGAACATATGAAGCA 53746
                * ** ***   **  ** ****    *** ***   *  ** ***      **  ** *

Human           TTCTAAA------ACAACTGCAGGATAACGATCTCATCATTCACTTCACTCATGCACTAC 49496
Gallid          TGTAACAGATTATACAAATGCGACATCATGTTCCTGTTATGTATTGAACAAGCCTGTTTT 53806
                *   * *      **** ***    ** * * **   * **  * *  **         *

Human           TGCCAGTGTTAA-AGGACATAGAAACGACCATCAGCAAGACCATCTCCGA---CATTCTC 49552
Gallid          CATCACCATGGATGGGGCAATGAGAAGAACGGCCGAAATGTTTCTTCCGGATTCGTTTAT 53866
```

```
                             **   *  *  ** **  ** * ** *  * * **       ****    * **

Human           G-AAAAGAT-ATTAATCAAAAC----TCCTCTAAACC--CCGAACAGATGTCCAAGGAAG 49604
Gallid          GCAGGAGATTATTGGTGGTGGTGTGGTGGATAGAACCATCTGTCAAGGTGATCGAAGTAT 53926
                * *  **** ***  *          *      ****  * *   ** **  * * * *

Human           AACAAAAATATACCCCTTTACTCAGTTTCTTATCCA----------AGTTCAAAAAAACA 49654
Gallid          TTTTACGGCTTCTGCAATAGATCGGTTTCTTATATATAGACCGTCTACTGTGAACAATCA 53986
                    *     *   *  *   ** *********  *           * *   ** ** **

Human           ACATTC-TGCACAGAAGATGTAAAAACAGAGATCGACCAAATGCAAAAAGTATCAC--- 49710
Gallid          AGATCCATTCTCACAAGATCTATACGTTTATGTAGATCCAGCGTTTACGGCCAACACTAA 54046
                * ** * * * ** ***** ** *     *  * ** * *  *   *     * ***

Human           GTTCCTCACGAAAATTGCTACCTCCACTAATAAATATACTAGAATCAGCCACTCCGTCTA 49770
Gallid          AGCATCTGGGACGGGTGTCGCGGTAATTGGTAAATATGGTACAG---ATTATATAGTTTT 54103
                         **    **   *    * *  *******  ** *        *    ** *

Human           CGGACAAGAATTAAACCTCTACGAAGAACGCATCACAGAGCTGAAAAAAGAGACGAACAA 49830
Gallid          CGGTCTAGAGCATTATTTTTTGCGAGCCTTGACAGGGGAATCCTCCGATTCTATAGGCTA 54163
                *** * ***      *  * *    **      *      **         *    *    * *

Human           AATAAAAGAACAGCTCTCCA---AAGAATACGCAGTAGCTGAGAAAAAAATTCTTCTCTC 49887
Gallid          TTGTGTTGCACAGTGCCTTATACAAATATGTGCGATCCATAGGAAGCGATTTGGAGTGAT 54223
                       * ****  *   *   **  **  **  *   *  ***   * **    *

Human           GTCACAAGATGCAAAAACCAATAAAATTTATCTAGTCCTAAATACACACAC-GCTGAAA- 49945
Gallid          AAAAATAGCCATCGAAGGCAATAGCAATCAAGATTCTGCGGTTGCGATCGCTACTAGAAT 54283
                   *  **       **  *****  * * *            * *   * *  **  **

Human           -GAAATAAAAAACACCCAATTCAGAGAAACGGCATTCGC---TAAAGCTCTCACGGTCGA 50001
Gallid          TGCAATCGAAATGATATCATATATGAAAGCTGCAGTGGCACCTACACCACATAATGTCTC 54343
                 * ***  ***  *     **  *    ** * *** * **   ** * * *  *  ***

Human           AGTCAACAACAAAGAAAATCAACTCCAGGAGCTGTTAAACCACTTTAACGCACACCTAAA 50061
Gallid          GTTTTAC--CACAGCAAATCGAATGGAACGGATGTTGAATATCCCTATTTCTACTGCAA 54401
                  *  **  ** ** ***** * *  *   * **** **   *  **       **   **

Human           GGCGAAAATGGATCAGAACCATATAACAAAACTTTCATTCGATACAAAATGG--ACAGCC 50119
Gallid          CGCCAAAA--AACTACAGCT-TTTGATTTCTTTATCGCCCAGTTTAATTCGGGGAGAGTC 54458
                 ** ****   *  * * *  * * *      * **   *  *  **   **  * ** *

Human           TTTGTTTCGGAT--TCCAGGCTCTACTTCCCAGACTTCGTTGACATCAAACTGCAGGACT 50177
Gallid          TTGGCATCGCAAGATTTAGTTTCTACCACAGTGAGTC--TTACCACAGATCCGGTGGAAT 54516
                ** *  *** *   *  **  *****  *   ** *   **  **   * * *  *** *

Human           TTATTTCTGATCCCTTTAAAGTTAT----AAGTCAGCTGATGAACAAAGCAGCCAACGAG 50233
Gallid          ATTTAACAAAACAACTTACTAACATCTCGGAAGTAGTTACTGGACCAA-CATGCACTAGA 54575
                 * *  *  * *   ***     **      *   ** *  ** ** ** **  **

Human           ATGCCTTACATTCAAGCAG-AGATAACTCTCAAATGGTTAACACAATTAGTACACGACAT 50292
Gallid          ACATTTTCTGGAAAAAAAGGAGGTAATGATGATACTGTTGTGGCA-TTAACAATGGCAGT 54634
                *    **       **  ** ** ***   * * *  ***    ** ***  *   *   *

Human           CAACAAATTC-TGCCTGTCAGCTATAAGCGAATTCGGGAAAGAAGCAATCCCGTTCAATT 50351
Gallid          GTATATATCCGCACACATTCCCGATATG-GCATTTGCCCCCATTAGAGTATGATCATATG 54693
                  * * ** *   *   *   * *** * * *** *          * *    *   **

Human           ACGCGGCCCTACGAGATTTGGAGTACCAGATTAACA-CCAAGTACGTAGAAATTGAAAAC 50410
Gallid          ATGAGACCCGACAAGTGATGAGTTGTCCCTTAAGAGTCCGAATGTACGTGCGTTAATAAT 54753
                * * * *** ** **   **   *  *   * *    ** * *         ** * **

Human           AAAGTCATCTGCAACGAAACCGTCGAGAACACGA----AAAACATTCCGAAACTGACTA- 50465
Gallid          GAA-TAAAATACAAC-ATACTAGCATGAATATGATTCTATCGAATTTATTTATTTAATAC 54811
                 ** * *  * **** * **   *  *** * **    *    ***     * * * **

Human           -AGTTCTTGA----AGGAGTTAGATCCTAAACGCGT-TGCCGGT--GGCCAGAAACAGTA 50517
Gallid          CAGTTTGCAATAACAGGACATAAGTCTCCCGCTTTTATATTGGCAGGGCCATCAAACGTG 54871
```

```
                          ****    *    ****  **  **     *   * *   **   *****  **  **

Human          TCAAACTCTCATGAACAA------GATTCTCACTTCCGAAACTTCTATGCAACAG--ACC 50569
Gallid         CATACTTTTAATAGAGGATGGAGAGATAATACATTTCCTAAGTTGAGGGTCATAGTAATC 54931
                  *  * * **  *  *      ***  *   ** *  ** **    *  * **  * *

Human          TACGAAAAAGAACAACTCAAAAA-AGAATACTT---CGAGACCGTCAATAATGTCGCCAG 50625
Gallid         CACGCGGAA-AGTGCCTCATAGACGGGATATCTTCTCCAAGCATCCTGCAGGGGTGGGGG 54990
                ***   ** *    **** * *  * ***  *   * *  *   *   *  *  *   *

Human          CTTCAAGTTAGCATTCAACTTTCCTAAGCAACGACAAAATGTGGAACGACTAATGGAGAA 50685
Gallid         ATTTGGAATTCTAATGAGTTGTCCGACAGCTCTTGTCGCCGTCTCAAGACCCACGAGAGC 55050
                **     *   * * *  * *** *     *        **   * ***  * *

Human          GTTCAAGTCGCTGCCCAAAGGCCAGCCATTTGAAAAATTTCCAGA----GGAAAATGACC 50741
Gallid         GTTTATATATCCATC-GGATGCCCCCAATGTAAGTAGCGTAGGGATCATAGTATACAAAA 55109
               *** *  *  *   *   * ***  * ** * *  *   *   **     * * *  *

Human          TATTTTCTGACTCACTCATCACAGAAAATTATATT----AACGGTCTGCGCGCACTC--C 50795
Gallid         TCATATTTCCTTCATTGATCGCA-AAAATCATATTCAATAACAAAGTTCTAACGGTAGAT 55168
               *  * * *   *** * *** ** ***** *****    ***    * *   *  *

Human          TCAACTTCATAACGGCTGCGCAAAACTACATCCAGAACACA----CTTCTG----AAACA 50847
Gallid         TCTGCGGCATCTAGGCTATGAAGCGTTGGACTAATAGTATATGATCTTCCATTATACATA 55228
               **  *  ***   ****  * *    *      * *  * *    ****      * * *

Human          ATGGGCGGTATTTCA------GCAACAAA----ATTTCATCCCGATCGATTACT-CAGTA 50896
Gallid         ATTGTTTCCGACTCACGACTGGTAGGAGGGGGCATTTGTCCTCTACCGATAGCTTCAATG 55288
               ** *        ***      * *  *      ****   * * * ****  ** ** *

Human          GCG-AATGTCAAGCCTATTTCGGATTTATATGCCCGCCTCAGGATTGAACGGGACCGTCA 50955
Gallid         GCACGAGATAAAGTCTTCGCTAATATTTCGTTTGCAAGTCTAGTAGGAACAGTAACGGGA 55348
               **   *  * *** **         **   *   *   ** *   **** * * **  *

Human          AGT----GTTCTAC-CAAGTAAACTC----TGTCTTTGGAACTCAAC--TAGTTGTTG-A 51003
Gallid         AATAGGAGCTCCGTGTCAATAGATTCAAGCCGTATTCGACAACAGACGTTAGGAAATATG 55408
               * *    * **      * ** * **     ** ** *  *    **  ***    *

Human          CGAAACAGGAGTTCCGCTGCAATTCCACAACATCTTTTACAACGCCGTAGTGAAGTTTTT 51063
Gallid         GGGGGCATTAGACAGACCGTATCTCCGTTGCATAGGTCA-AACGGACCAGT--ATTTTGT 55465
                *   **  **     * * *  ***    ***   * * ****    ***  * *** *

Human          CTCCCTGAAC---TACAAGCAGATTC-ATGTACCAGAAGACACCCCCCGCCTGGT--ATC 51117
Gallid         AGCGCTAGGCCGTTAGGAATAGATCCCATTCCCAAGACTACAGCGCTCATTCGGCCAGGC 55525
                 * **   *   **  *  **** * **   * ***  *** * * *    **     *

Human          TAGCCAATATAAACTCCTGTCCGTGTGTAAATCTTTCATAATTATACTA----CAGCAGT 51173
Gallid         AAGGCACGAGTGATGACGGCAGCAAAACGAGTTTTGAATGTTGATAGGAGACTCAAGGTG 55585
                ** **  *   *   * *          * * **  **  * ***  *    **

Human          TCTGGGAGAATATAATCACTCT----GGACCTA-GGCCCGTACCTCAGAGACGGTACACA 51228
Gallid         TCCGGTTCGGTTCCCGCAATATAAAAGGACTTATAATCTACATCTTTCAG-CAATACTCT 55644
               ** **     *     ** * *    **** **    *   * **   ** *  *** *

Human          AAATTTTAAAAG----AGAACTTATAC-CGATCGTGAACTTGAAATTATTTATATACATC 51283
Gallid         AGCTCTGACGAATGGTAGGAATAGTACTCGCCCCTCACATCGTTGTAGTAAATTTGCATC 55704
               *  * * *  *     ** * *  *** **  * * *  * *   *  *  ** * ****

Human          ATA-ACACA-AGCCTGGACAGCATCTGAAGACAGTACTGTTTCCACAGCGTTCGAATTGC 51341
Gallid         GCATGTAGATAACTTGCACGGAAGCTCAAT----CTCTACTATCGTGCCGTTGGAAGTAC 55760
                 *   * * * * ** ** * * ** **       **  *  *    **** *** * *

Human          CCATCA-AGCAATTCACACTTTTGATACTATGCTCACATCCTGAATATCTCTACGGCTGT 51400
Gallid         TCATGGTAATGATACGAGAGATGTATGCGTCGTGATCCTTCTATTCACGTTCGCGGATAA 55820
                ***   *   ** *      *  ** *   *    * * **    *  *   *** *

Human          CTCAGTCATCCAACAGATCTGGTTATAAATTCATTAGCCAAAAGCATAGACAAAGA-CAG 51459
Gallid         CTATACGGGTTGACGGGACCGTCGGCCGATATAT-----GAATGAGTGGGCGGCAATCAG 55875
```

```
                **          ** *  * *       **  **      ** *  * * *    * ***

Human           TCTCTATGACACATTCGTCGTTAGCCACAACCCTCCCGAGAAGCCCATGCATTTGATGAG 51519
Gallid          TATCAGTTACAAGCTCAGCATATGGGCAAACATCCACCAATAGGCGACGCATCTCGAATT 55935
                * **  * ***   **  * *  *    ***   * * *  ** * * **** *

Human           AAGTATATGTATCGACACCCAGCTATGGCAATCCGCCAAACTTATGAAAGATACCTTCCA 51579
Gallid          AAGTAT-TGCGCCAGGTGTGTTTCTGGATTATCGGTAGTGCCTGTAGAATATGCGCGCAG 55994
                ****** **   *             *   *** *     * * *  ** ** *   *

Human           ACAAACATT-----TTTTACACAAC------TGTGCCCCCAAAACGA------AAAGTTT 51622
Gallid          TAAAGCTTTATCAGTTGCGCATAATATAGGATATGCCTCTTGAAATAGGGAACAGGGGTC 56054
                  ** * **     **   ** **       * **** *   **  *      *  * *

Human           TTTATCTATCTCACTGCCTTC--TTAATTCTCCCCTACAAATTTCTGAACTATATTTGGA 51680
Gallid          CTCGGTCAATTCGGTGGATCCGATTGGTCGTTTGAACTGTACCTCTCCATCAGACGCGAA 56114
                 *     *  **  **  * *  **  *  *         *  ***  *  * *   * *

Human           TTCAATACAAACCGATCACTTTTACCCAAAGATCTTATCAAAAC-------TTAATTAAG 51733
Gallid          AGATGCCAGAGCAGAACCAGCTTCACCAATCAGCTTATCTAGACATCGTGTTTTACAGCA 56174
                         * * ** *    **  ****  * ****** * **       ** *

Human           GACCTATGCTCCG------AATACGTACACCAAAATAAGATCACCATGTCCTCGGTAACT 51787
Gallid          CACTTCTGCCGGGGTGAAAAATTTATATGTAGGACTAAAAAGTGGAGATGCTCCGCT-CA 56233
                 ** * ***   *      ***   **      * *** *     *  * *** *   *

Human           CCCCACGAACCGGACACAATAAA--ATCCGGAGAAAAGATTACCTCAAAAATCACCG--- 51842
Gallid          GATTGTAAACCCCGTTGTATAATTTATCTGCATAGGAGAGTCTTTGTGATGCCCACGGAT 56293
                       ****       ****   *** * * *  *** *   *   *   *  **

Human           TACACAAAGCACAAAACACACCGACACTCACTCGCCTGCAGGCTCAGGAATATGTCTTCG 51902
Gallid          TATTGGTTGCACGAAATGGATATGCGGG-ATCCCCTTGTTGATGATCGTACATGACATCA 56352
                **      **** ***   *    *    *  * * **  *      * * *** * **

Human           ACTACATTCTTTACTCTTTCTTGACGGGGTA-CGAAATGACA-TTCGCTATGTACATCGA 51960
Gallid          TCTATATCGGATGCTTCTTCGCCCGAATGTATCGCACCCGCAGCCCTCCCTCTTGGATTG 56412
                 *** **    * **  ***        *** ** *    **   * *  * *

Human           CACCAT-CGAAAAAACATACCTGCTTTGCATGAGACACCTGGAAAACGTACTCCACGATA 52019
Gallid          CATGGTGTCCTAAAATACTCCAAATCAGCAGAAACTGGCGTGGGAATAAACTCGC--ATA 56470
                **   *     **** *  **   *  ***  *     *  *  **   ****    ***

Human           AAGACTTCCAATCCGTCCTCAGAGCAC---GCACATTCGACATCA----ACTACATCCTG 52072
Gallid          TAGACATCTGCCCATGTTCCAGACCACTCGGTAGATTAGGCATTAGTGTACCTAAAAATG 56530
                 **** **    *      **** *** * * *** * *** *    **   *   **

Human           AAACAGTCAT---GGACTAAGAACATAGTC-GAACATTCAATCTTTTCAGTACAGCTCA- 52127
Gallid          GGACAGTTCTTTGAGGGGCTGCCCGTTGTCCGCGTGTTGTTACTTTGCGTAAGAATTCAT 56590
                  *****  *    *     *  * * *** *    **    **** *   * *  ***

Human           ------------ATAAAAT------CGTCTCCTACCTCAACCACACAAACAGAGCGACAC 52169
Gallid          CTGCCTCTTGATGTAAAAGGGGAGGCGAACCTCGTGTTAGGTGCAAACTCTGAACGGTAT 56650
                             *****       **   *     * *    ** *  * ** **  *

Human           CCAATATTCCCCTGATACTATTTAATTATGACAACGAAGT---TGTCAACGTTTATCTAC 52226
Gallid          TTCCCATATCTGTTATAGCATTCCGCAGGAGCAAAGGAGTACACGTGGCTGTATATCCTA 56710
                     **  *  * ***  ***         *** * ***    **    ** ****

Human           CGCCGATGTCCACCGATCCAAAAAAAGTCGCTTTCTACATAAAAA-ATCCTTTCCACTTT 52285
Gallid          CACCCAGGTCAAC--GTTGGCCCGGGGTTGTGTAAGAGAAAACTGCACCCCTCCCGCATG 56768
                * ** * *** **   *         ** *  *   * * **    * ** * ** * *

Human           CCCGTGCAAGAATACGAAG--------CGACAGATTTAATTTCCTTTCATCTCTACCCAA 52337
Gallid          TGGCCGCTTGGTGACTGAGACTTGTCCTAGAAAATATGATTCCGAAGCGCGTTCAGCAAA 56828
                     **  *   **  **            * ** * *** *    *   *  * * **

Human           AAACAACAGATATTTTAAACCAACTTCC-GCCCAATAAAACTGTGT-CTACTCGCCCATA 52395
Gallid          TAAACCCATGTCTGCTAAAAATCTATCCTGTCGTATAACAGTAAATGCGATTCCTGGATG 56888
```

```
                 **   **  * *  ****       *** * *  **** * *   * * * **    **

Human            CAACCTTAGCTCTGAAACTTTAACCACCAAAAACCTCTCCGAACCAAAGTTTAAACAGCC 52455
Gallid           CAAACCCG--TCCGAAGTT---GGTGATATAGGCCTATG-GGACTTAGTTTGAAATAGCC 56942
                 *** *     ** ***  *         * *  *** *  * **  *  ** *** ****

Human            TACCGTTACTG-----GACTTATGCCCAAGAGCCAATCAATCATCCTATCCACTGATACC 52510
Gallid           TGCCATCAGCGCGTATGATATTTCATTGGGACCACATCGGCTCTCCTTTACATATTGTGC 57002
                 * ** * *  *     **  * *      ** *  ***     **** * **       *

Human            AACGTACTGGAAACCAGTCCAGACATAAAA-GCTAACACGGCCAGCGCAGCGATAAAAGA 52569
Gallid           AACGGGCTGACGGAACGTGGAATAGTAATTTGCTCCCAGAAATGGTGGAACTGGAGGAGC 57062
                 ****  ***        **  *    ***   ***  **       * * * *   *  **

Human            TGT---CACTCTAGCTCGAGAACAAATCAGCGAGTTCTCCGAAT--CGATAAATACAACT 52624
Gallid           TCTATACAACAAGGTTCGAGAGTTA-CCAGGCAGATCCCTGACTGCCGCTAAATGATCAT 57121
                 * *   **     * ******   *  ***  ** ** * ** *  ** *****     *

Human            CTTTCAAAATTAAAATCTTTGTATCTGTAACATTCGA-CACACAAAGTCATGCTCCTTTT 52683
Gallid           GACATGCAAACAAGGCGTTCACGGGGACAGGATAGAAGTATGTTCCAGCA-GCGACTGTT 57180
                        **  **    **          *  **   *  *        ** **  ** **

Human            ATAAATTTTTATTAGTTAAACATACGAAATAAATGAAGCTTTCATCAATCACTCCCTGTT 52743
Gallid           TCATCATTGCACGGATAAGCCATCATTAGGAGACCATGGTGTAATGCCCCATCGAATGTT 57240
                   *   **  *    * *  ***     *  * *  * * * * * **    **     ****

Human            TT--TGTGTTCAGTCTATGCAGCGCCAGCATTCTTATC--AAATCGAGTCGGAACGCCGT 52799
Gallid           CGCATATGTCTAGTGGATGATGAGCTGCTGCCTATATCTGGAGTACTTCCGTAGAGGATT 57300
                     * ***  ***  ***  * **          ****   * *     ** *  *   *

Human            TTTATCCACGTTACCGCCGTTCTGCTTAGCGTATTTTGGCAAAAAGACCCCAAGGACGGG 52859
Gallid           GCTGTTGTCCGCTCTGCCATATTA-TTAACAGTTTCTTGTAAAG------TAAGAAGAGC 57353
                   * *   *    * *** *  *  *** *   ** * * ***        *** *  *

Human            ATGATTGGCCATGG-TAGGCTGCAGCCCTAAAACACCGGTAAGCATCTGCTGCTTCTTCT 52918
Gallid           ATCAGCATCTACAGATATTCTAGCGTTATGCAACATCACGTTAAATGTGTTGGGAACTAA 57413
                 ** *    * *  * **  **    *   *  **** *       ** ** **      *

Human            TTTCCTCCTCTTTTTTTGAAG----AACTTCCTGAAATCTGAGTGATCTGATTCGACAAA 52974
Gallid           ATTTCTGCCATTTAAAGGATGCCTGGGATCTTCGGGACCCAGGGGAGGTTCTTCATCTGG 57473
                  ** ** *  ***    ** *        *    *  * *   * **  *  ***  *

Human            TCATCA-CCACGTATGGTCGTCATTTCAAATACACCTCACGTTTTGGGAAATCTGGCAAC 53033
Gallid           CTGGAGGTCAGGAATGATGACCGATTGCGAAGTAGCATAAATGTTGTCAAATCGTATACC 57533
                         ** * *** *   *  **   *   * *  *  * ***  *****    * *

Human            CTTGACAGATATTAACACAGTCTTCCAGATCTAAGCAAGTCTCTCTAAACGAATGCTGAC 53093
Gallid           CATAGTA-----CAGCACTGGCCTCTAGAGA-AAGAGGGGACCATTACAT-AATAATAA- 57585
                 * *   *      * *** * * ** ***   ***   *   *  ** *  ***  * *

Human            AATGCTGACACTGAAAATCAGAACCGTGTGAAACAAGCCGTAACAACTTCTTGACGGTCA 53153
Gallid           ATCTTTGAGAGTGTAGTCCACTCTTGAGCGTGATGAGGGACAAATGTTTGTCGATCTGCA 57645
                 *    *** * ** *   **      * * *  *  **     **    ** * **    **

Human            CGTTGTAGAGATGCACAGTAGAATAACAGGATCTACACGAACAAGGAACAATAACATCGA 53213
Gallid           TCTCGTATTGGCCTATTAT-GAATGAGGACAT-TGCCAGTTCTCTGAAAATCGGCGTC-- 57701
                   * ***  *    *   * **** *    ** * *  *  *   *** *    * **

Human            ACTTCACATTGTCATTTAAAATAGTGCAAGCAGAATTTGTTCCGATCACCGCCTTCCATG 53273
Gallid           ---TTGCAATTCCAACCACGGACGCACG-GCATAATTGTTTTCGCTCCCTACGTTTAGAT 57757
                    *  ** *  **   *     *  *  *** ****  ** ** ** *  * **

Human            AAACAGTTTTCACCTTCATCCCCAATACACAATGCTCAGACATTTCATATATCCGCTCGA 53333
Gallid           ATAAATCCCTATCGCGCATACCTTCAGCTTGGTATATTAGGGGGTCACAATCCCATAATA 57817
                 * * *    *  *   *** **     *    *           *** *   **      *

Human            ACACCAACTGCTGACTCCCACACAGCGAACAATACATTACATCCGTATGCATG---CTGT 53390
Gallid           ATGGCGGTAATATAGTTCTATCTATCAGAACGTGATTTAGTTCTTCATGACTCTGTCCGT 57877
```

```
                *   *        * * * *   * *  *   *   ***  **   ***  *    * **

Human           ATATCAAATT--TTTCTCCTGCTT-GTCTCTAA-AATAGAACATGGAGCTCAACGA---- 53442
Gallid          ATAATGGATCAGTTTGTACTGTATAGTTTCGGACAATTGATCGCAGAGCACGAACATGGT 57937
                ***    **   *** * ***  * ** **  * *** ** *   **** * *  *

Human           -----AAAGCCCTGCGGCGAACACAACTTTTCTTTTCCGGAGTTTAA--ACAGTGACCAC 53495
Gallid          GCATTAAATCCTTGTATACATTGGTACATTCCTCTGGCAGTTCCCCACTACATAAATAAG 57997
                     *** ** **     *     ** ** ** *  * *      *  ***   *  *

Human           ATTTTGCACAAATAACGCAAATGTAACTCTCTT-CAAGTATAAACCGAGAACATTTAGTC 53554
Gallid          TGTCTATAAAGGCTAC-CATGTGAAAGTTGTTGACGAATGCAACTCGTTTGCAGTTATCC 58056
                  * *  * *    ** **  ** ** *   *  * * *  **  **     ** ***  *

Human           TTACAA--TAGCACATGACGGGTAACGAAATAACACTGGTAGCTAAGCCATCCTTTAAGG 53612
Gallid          CAATAGCTTCGTATACATTGAATGACCAA-TCGCATTAATAATCCAAAATTACGTTCA-- 58113
                  * *   * * * *    *  * ** ** *  ** *  **    *    * * ** *

Human           ACGTTGCCAGGGCAAAGGTTAGAATCTTTGGAAAATCACCCCGTTCAGGTTTGCTCGGCA 53672
Gallid          --CTTCCGTGAATAACAGCTTCAATGATATAAAGGCAGTAGGATAGCTAATGTCGGAAA 58171
                   ** *  *   **  * *  ***  *   ***  **    * *      **   *  *

Human           CCATAGGTACTAGATTATTATGCTCCACTGGAATAAGATATCTGTCTGCGCTAAAACAGA 53732
Gallid          ATGTTGCAGACACAGCATTGATTGTTGC----ATCACTCATCTTATGGTGAGTGGATGCA 58227
                   * *     * *  ***        *    ** *  ****     * *     *   *

Human           AGTTTTCCACCAAAGCCTTCGCCATAGTAAACATG--AAATTCCCCGCCGGAGATTTCAA 53790
Gallid          AGTTCTTGACCCAGAGCATCTCTAAAATCGCTGTTGCAAAGAGGTAGAGGTATGTTACAA 58287
                **** *  *** *   * ** * * * *     *   ***      *  * *  ** ***

Human           A-GCATACTTCT-GCAACATGGAGGGAAATGACAAAACGTGCTCTCTCTGCCTATATCGC 53848
Gallid          TTACATACTCGTAGTGATGCGGAAATGGAGGCCATAACTTGTGGGACGT---TATGTGGT 58344
                   ****** * *  *   ***     * * ** *** **       *   *** * *

Human           AACATCACCGTCTCGGTCTGCTCATAGTCACGTAAACAGAATAGCCTCATACACGGTAAG 53908
Gallid          CCCGGGAG-ATCTACATCT-CCCGGGACCACAAAAAAATCA-AATGCTGGGTGCAATTCC 58401
                  *   *   ***   *** * *     ***  *** *  * *          *  *

Human           TTGACGTGCGTCGGAATCGGAAGCA-ACAACTGTTGCTCGAACAGAAACATGTGCAACTC 53967
Gallid          ATAG-ATAGATAGGGATTTCCAGTAGATAAGAATTGCTC---TACGGTCATATGACACTC 58457
                 *    *   * ** **    ** * * **   ******    *    *** **  ****

Human           ATTGATTTCGGAAAACGAGGGCATCTCCAGCAACTTATTAAGCTTGTACGAGGGATAAAC 54027
Gallid          ATCTATCC----ATCTAGGGGGGTCGCGTAGAAATTCTCCGATGTTTTCGAAAAATTGGC 58513
                **  **      *     ***  ** *    **  ** *      * * ***   **   *

Human           CCTAAATCTAGTCTCGACGTCCATCCATTTGATTGTTTTAAGTTTATAAAGAGACAACAA 54087
Gallid          GACTGGCATCAGCTAGA-GGCAACGCTGGTGGGTTTGTCACATATGCTCCAAGAACACAA 58572
                        *   ** ** * * *  *   **  * * * *  * *      ***  ****

Human           TTCGAGAACTTTGCACTGCCGATACACGAAATACAGATAGGCGAG--CAAAAC-AAAAAG 54144
Gallid          GACAAGGTGTAT---TTACCGTTACGTAAATTACCAACTGTCGCTTCCACATCTAAAAAA 58629
                  * **   * *    * *** ***   ** ***  *  * **    ** * * *****

Human           ACCCTTTGTCATAA-----CCATTTTGTTAAAAAGTCGCTTAGTGATAAATCG--CGTCT 54197
Gallid          GATGAATGGCATAGAGTTCCGATTGCGTCTGAAAGGGAGAGACTAATTACTTGATTGTCT 58689
                      ** ****      * ***  **   ****      * * ** * * *   ****

Human           CCGGCCTGCAACGCAGCCAAATTGCAAAAAACTCAT-TATAAAATTTTTCCGTTACTTCC 54256
Gallid          TTGTTATA-AAAATATATTTCTCGAGGAGGAAAAATACGTATATCCCCTACATTACATCC 58748
                  *   *  **   *      * *   *  *   **   ** *     * * **** ***

Human           TTATTCAGGAA-----TTTTTTATTTGTCAAGAACATTGTAAGAACACATTTTCCTGGCA 54311
Gallid          GGGCCCGGGAGCATAGTCCCCAATATGACGGGCATATC-TATCACCCCGTTTT--TGATA 58805
                     * ***      *     ** ** *  * * **  **  * * * ****  **  *

Human           CACAGGCGTCACCCATCACAAACAATTGCAAATGAGTGTACGGCCCATGTTTACACAACG 54371
Gallid          TATTCCTAA-AGGCATGATAAATG-TTAAATCTACATGTCCAATTAATGGATATTGAACT 58863
```

```
                 *        *  *** * ***   **  *  *   *** *     ***  **   ***

Human           GGGCCAGAAGCTGGCGTAGGAGTTTTTTTAAGTAGAACATAACAA-AAATACAAGGCTAA 54430
Gallid          TGGGTTG--CTTGGTAAACACGCCGTTCCATGGATTCTAAGAAAATAAGTTTATCGCCAA 58921
                 **   *    ***   *   *   **  * * *    *  * ** ** *  *  ** **

Human           AAAAAAGCAGATGCTGACA-ATCGCAACCCGACAATGCCTTTCACT-TAGTCTCGAAAAC 54488
Gallid          TGGTTACTAGTTCTGCAGATATCTCTGCTGTTTGAGGTCGGTCATCATCGCCCCGCACGA 58981
                     *  ** *    * * *** *  *      * * *  ***   * * * ** *

Human           TTCACACCGGACAGGAATGACTTTTCTTTTTCATAGAACTG-GTTTATACTCTCCTGTGA 54547
Gallid          TTTCATCCGTACGATCAATAATATTGTCTTCCTTTAAGTTGAGAATATATCGTGCAATGT 59041
                **    *** **    *  * * ** * ** * *  *  ** *  ****   * *  **

Human           TGTCACAACCGCAGA-AGCTTTATTTGCAATTTCTCCGCTACTTCCGAGTTGCCCCTTTT 54606
Gallid          CGTCCATGTTCCGTACAGCTTTACC--CATTACTACAGCGGTTACTAGATTGGTGCCCGA 59099
                 ***       *  * *******    ** *    * **   * *    ***   *

Human           CCAAAACATGTGAAGACCTTCTTCGCAAACCTTGACAAAACCTATTCGATTCGAGATGCT 54666
Gallid          TATTATCATTTCCCCGTATGTAACAGGTACTCTAGCAGAAG-TATCCACCACATCAAGAA 59158
                    * *** *       *    *    **  *  ** **  *** *    *   * *

Human           GTTTCCCCATAGAACACTTCTTTAAGAAAATTGCCTTCAAAAGCGAAATGTATTCA-GAT 54725
Gallid          TTCCAGATAACAATTTCTGTCTTACCATCGTAGTTGTCACTAATACACCATCCACAAGAC 59218
                 *      *   *   **   ***  *   * *   ***  *    *   *   ** **

Human           TCGTTTTCAGGCCCAAACGCCACAAACTCCACAACGTAAGTTTCGTCTCGATTACGCCTA 54785
Gallid          GCCCCTTTGAATCGGAATGTGTTAATCGTGGCATTG-AAATTGTTTGGTGAGTACAGTTG 59277
                 *   **     *  ** *    ** *    **  * ** **   *   ** ***   *

Human           ATTATACATTTCGTGAACACTGCAAAAGTTGAAGGCTTCATAGCCACTATGTCGCTCATG 54845
Gallid          ACCAT---TTTTGTGAGGAATGAAATTATAGTGTCCCGGTTGGCGTTTGTCTTATTCATG 59334
                *  **   *** ****  * ** **   * *    *    * **   * * *   *****

Human           CGAACAGTCA--------AACATTCATTAACACGCAGAATGTTGCATTCTCTCACAGCAT 54897
Gallid          ATAAAGCTCTCTGTGCGCACTGTACGTTTGAGCTCGGAG-ACCAAATTTGCTCGCATAAC 59393
                  **   **         *   * * **    * * **       ***  *** **  *

Human           GATGGCCTTCTGCTTTAACCGGGGCATGCAAATAGCAAGACATCGACA-CAGCTCCGCCT 54956
Gallid          C-TGTCCAGCAATTCTCCCCTCGGCTTGACTGCGGGAAAGCGGTATCAGTAGAGGAGCTG 59452
                  ** **  *   * *  **  *** **      * **  *     **  **    **

Human           GTG-----TTTTTGAACACAAATCTCGGTTCCTGCTTAGT--TACATGTTCCCAGAGACT 55009
Gallid          GTGGAGCTTTCTCTAGTAGAATTCTAAGCATTTGATCTACCGTACCTCTTTCAAATGAAT 59512
                ***     ** *  *  * ** *** *    ** *      *** * ** * *  ** *

Human           CAACAGATATTCT-AGATTATAAGCGTAGTCTAACTTGAGAATAATATC-ACACAAA-GG 55066
Gallid          CTAATATCGTTCTTACATTTCTAGCTAACTGTTGTATGGCTCTCATTCGGAGATGAGTGG 59572
                * *      **** * ***   ***  * * *    **    * **    * *  *  **

Human           AAATTTCGGATTTTTCGACAATAAATTCCGCTCAG-----------TCATCCTATAGTCA 55115
Gallid          ATATTGCTGTCCCATCTAAAGACACTTCAGATAATAATGCGAGGGCTTCTGCCGCGACAA 59632
                * *** * *     ** * *   * *** * * *            *  * *       *

Human           TGAGACCCCAACTTTAAGATA--CGACAGGTCGCACTCAGTAGTTCATCGTACAT---CT 55170
Gallid          CGAAAGCTGCGCTCAAAGATCTTCGACATAGACGTTTTACCATATAGGTATGAATGGGCT 59692
                 ** * *    **  *****   *****        * *  *  *     *  **   **

Human           TTTCTTTGAGTACGTTAGCCATTTCACCTATTTAAAAGCTATTCCGTATTTAAATATTC- 55229
Gallid          GATCCGCGGG-ATGTGGCCCTTCGCGCGCAATCATTGGCTGTTGTATTTCAAATTGAACA 59751
                  **   * * * **   ** *  * *  * * *   *** **   * *  ** *   *

Human           -TTGATGTTCTAATATTCCATTTACATTTAACAGCTTTGACAATATCGAAGTGAGAAGTA 55288
Gallid          GTTCCTTCTCTCATGTACATCAGATCTGGGGCGCGTGTACAAACACATGCAACAGAAAGG 59811
                 **  *  *** ** * *     *  *    *   * *   ** *        ****

Human           CCATTTTCTGAGCCATGACCTCTCGATG-CACGCAATCGACATCCCCGTGAACGTATCTA 55347
Gallid          CCCGTTTCTAGAAATCGAATTAGAGATAATGTATTGCAGTAAGTCCCGAGAAGAAGATCG 59871
```

```
                **  *****       **  *   ***           *  *  **** ***

Human           GAAAAAGCAACGTGTCTATATCCAACACTAAGAAAATTCAGTCGCGCCTCCAAAGAAGGA 55407
Gallid          AATTGTGCAGCATA---ACGTCCATTATCATCAGAACTGAATTGC---TTGAAATAGTCG 59925
                 *    *** * *    *  ****  *  *  * ** * * * **   *  *** *

Human           AGCATCATTTCATATAAT--CGTGGCAGCATTGGATAAAACACGACCTCAAACTCCCTTC 55465
Gallid          AACATACATCGATGAGATGACATCTCTATAGTGCTCAAGAGTCTACCTGTAGGCGCTAAA 59985
                * ***   *  **   **  * *  *   * **   ** *  * ****  *    *

Human           CCTGCTCACAGACTTGCTCATACAAAAGTTCAAGATTCGTCTTTTCAGGTGAGGAGTCCA 55525
Gallid          GTCGCTCCTATGTTGAACGGGGTTGAAAATGTACAT--GGTGCCACCGGCGGACAATCCC 60043
                   ****  *   *           **  *  * **  *      * ** *   * ***

Human           TCATGTACAAAGGATGGGACTCAGAAGCG-TTGTCTATACAATCTGAGATAGTAAACGAG 55584
Gallid          CTGCCGGGGGACAATGGCATCCGGCCATAATCGTCAATATACTCTGTCCGAGCTGGGAGA 60103
                          *  **** *  * *      * *** *** * ****    **

Human           ATTCTGTTATATTGCTAC---CTCACTCCCCAACCA--CCAATTCCTTCCGAAACGACAA 55639
Gallid          AGTTCGTTATAGTAGCACGATCCGACGCTGTGGATAATCCAGGGCAGACTTAATCAATAC 60163
                * *  ****** *   **   *  ** *       *  ***   *   *  ** * * *

Human           C-AGCCACCAGCCCCACGAACA-----TAGAAAACGAAATC-TCCAATTTAGAAAGCTCT 55692
Gallid          TTGGAGATCGAGTCGAGGGTCTCGTTTTAATGGACGAGGTTATTCGAATATGACACATGC 60223
                   *  * *    * * *  *      **    ****  *  * * * *  ** *  *

Human           GAAAACCTAGA--GGAATTAAAGC----GAT-------TATCCGTCGCATTAAATATAGA 55739
Gallid          GAGTGCCGATATTGTAATGGGAGCATGTGATAAAACTATGTCCATAACGAGGGAAATAGA 60283
                **   ** * *  * ***   ***    ***       * *** *  *     * *****

Human           --CAGACGATGCAATATCTG--CTCCATCGTAAATCTGTGCC-TCAAACAAAACAAATCA 55794
Gallid          ACTGCATGACAAGGTATTTGGACGCCCGCTTTTATCTTCACTATCAGACTCGGTGCA-CG 60342
                     * **     *** **  * **  * *  ****   *  *** **       * *

Human           TGGATCTACGACTACAGTCTACTGTGCTACAAATGCAACTACGCGCCAAAGACTCCATTG 55854
Gallid          TGGCCCCACAAATAAGGGCGACGCCCCTTACATTAGAATGGCACAC--ACGATTTAAGTA 60400
                ***  * ** * **  * * **    **   * *  **   * * *  * ** *  * *

Human           TCTCTTCTGATTGTCTCCGCGGAATTTATAATGTTAATAC----GAGAACGATTTCCAAA 55910
Gallid          CTGCTCTTGTCCATAACCGAGGGAGGAGAAATCCAATTGCATCTGATATAGATGTTATAG 60460
                   **  **    *  *** ** *     ***   * * *    ** *  *** *   *

Human           CATCAATTTTGACGGCCTTTTTCAGAACAACATTGTCTCCATCT-TTGACTTCCACGTTC 55969
Gallid          AATATGCGAGACTAGCTATCATCACCA-AATATATTACAAATCCGCTTGTCTTCAAGTTT 60519
                 **           **  *  ***  * ** **  *    ***   *    * ** ***

Human           ATTTTTTTATTCACAGATGCTTCGCAAATACGGTCAACGATCACATCCAAAGCGAAAACA 56029
Gallid          GTATCAAAGGCCA-AAATTTTTTTCATAAATGTTACATCTTTGAATATCATGTTGTAAGA 60578
                 * *       ** * **  **  ** * * * *  *   *   **   * *    ** *

Human           TAACCCTG---AATCACATGGCCATAATCCGATCGACCCTACTAAAAGAAGACAGTATAC 56086
Gallid          TATTGCTACGAAGATAATTGGACCTAGCATAGATAACGCTCCTATTAAAACTTGCTGGAA 60638
                **   **    *   *  *** * **         ** ** ***  * **     *  *

Human           CGCACATCAAAATAAAAAAATTTCTAACCAAAAAAATGAACCCGAAAAAAACACAAAGCC 56146
Gallid          TTTAC-TCAGAGGCATTAAGTCGTATTTTATGTTCGCGTACATATAATACATTTCTAGCA 60697
                   ** *** *   *  ** *        *       * **    ** * *     ***

Human           CCGA-ACTGAATAAAAAACTCACAGTGCCCATGAAGACTCGCTTCACGACACTCTTATTC 56205
Gallid          TCAATGTTCCATAGAAAAAT-GCAGTGATTGTTGTACCCAAAATTAAAAACCGATTGTCC 60756
                 * *   *  *** **** *  *****    *     *     * *  *  *  ** * *

Human           TATATGTGGTCTGGCACAAACGTCTTCGACCGAGTTCCCTTCACGGACCTAACTATCCGC 56265
Gallid          TGTGCGTA-TATGGCATAAA----ATAAAAAAATTAAACAGCATGAAGGT--CGAAGAAC 60809
                * *  **  * ***** ***     *  *   * *  *   ** * *  *  * *    *

Human           AAACATCGCTTCATCAAAAAC--CTGTATTCCAA-CAAAACCGACATCGAACTCACAGCT 56322
Gallid          AAACGTAGATAGACCAAAGAGTACAACATACCGAGTAAATTTGGGATGTATGTCATAGCT 60869
```

```
                ****  * * *  * **** *   *   ** ** *  ***   *  **  *  *** ****

Human           GGTCCCA-TTCTCTTGGCACAGATTCCATTTTCCATCACCAAAA--ACAAAACAACCAGC 56379
Gallid          GTTTGGAATTGTAAAGACATAATCGCCTAAATAAGACGACATAGTTGCATAATCAACTGC 60929
                * *   * ** *   * ** *    **    *    *  ** *    ** **  * * **

Human           GTCTGTCTG--CTATGTGAACTGATGGCAGCTTCCAAACAAGATTACCTATTTTTAAAAT 56437
Gallid          ATCATCCCAATCTACTTCCTCAGATAGTTGTGTCAATACAGACCCAAGAGTTTTCG---C 60986
                 **   *    ***  *   * *** *  *  ** * ***     *    ****

Human           ATCTACATCAAAGCATCATGGACTACTGTCAAAACAACCTAAAGATGATAGATAGAGTTC 56497
Gallid          ATCTGTATCCGATCGTCCATCATCAATATCATCGAGGGGGATGGTATATTCATTAGCTTC 61046
                ****  ***  * * **    *  * * ***         *  *   **  **     ***

Human           AGTTCGTCATTGCCGATATCTTCGAGAAGACAAAAATACACATGCACGTCAAGAATCTT- 56556
Gallid          TGTTGCATAGTATCCAAATCCATGCTTCGACATAGTTAAACAAGGGAGTGGCCTATGTTA 61106
                 ***    * *  * * ***   *    **** *  ** *** *   **     ** **

Human           -TCCGACTAC---AGCAAGGCGATATTCGATAACGAATTCTCATTCTCCGATGATAAC-T 56611
Gallid          RTACAATTCGTCGAATAATGTGATGGTCTGAGACCCTTATTGCTTTGCCACAAATGGCGT 61166
                 * * * *     *  ** * ***  **    **   *  *  **  **    **  * *

Human           TCACTCTCGA-------TACCCACGTTTACCTGATACTCCGCCAGACTGGAACAGTGGGA 56664
Gallid          TCAATGACGAGCTGCAATACCCAAGCTCATT--GTCGTTCATATGAACGCGACMGTAAAT 61224
                *** *  ***       ****** * * *     *  * *    **  *  ** **

Human           GT-CTATAAGCACTTCTTCTGTGATCCTCTGTGTCTGGCAAACTGTAAAACCATT--AAC 56721
Gallid          ACGCTACACG---TTAATCCGTGCACCCACGTGATTAAACAGCAATCGCATCGTCTCATC 61281
                   *** * *   **  ** ***  **   ***  *    * *  *   * * *   * *

Human           CCGGAGGTTCTCTTCAACACTACAGACGCCGGAG---AAATCCAAGACTTGAAAGTCACC 56778
Gallid          MAGGAATTGCGATTTAGCGCTACMGTGATATAGGTCTACGTCCAGAGAGTGACAGTCAAT 61341
                  ***  * *  ** * * **** *        *   *  ****     *** *****

Human           ATATGCTACCGTAACGAATATTTAAGCATCGTCGAAAAA--CATGTTTGGCTAGCGATAC 56836
Gallid          GGATATTAAATACGGACATGTTCTCGC-TTATAGAAATGTCCAGTTTTATGTATCGGAAT 61400
                  **  **         ** **   ** *  * ****    **  ***   ** **  *

Human           ATCTTTTCAAAGC------TTTTCAGATTATTAAACCAAACCATAAAAATAAAACTCAAA 56890
Gallid          GTGGATATAAAGCGTATTTCTTCTGCGGCGGTTGTCTTTTTGCTGTAGGCAGGCCTCGTA 61460
                 *   *  *****       **         *   *       *  *   *   ***  *

Human           T---TGCAGAGTTTCTCAAAGACTTCACTAACTT---ATTAGCGCTTCACCACT-----T 56939
Gallid          TAGATGATGAGTATCTACCAGAGCTCGCAAAAATTGGGTTGGTTCTACGTGGTGGTCAGT 61520
                *   **  **** ***   ***  ** * **  *    ** *  ** *           *

Human           CGATATCGTCGAT--CCCATTTTCACCGTCAACTACTACG------TTTAAAGAATGACT 56991
Gallid          CCAAACCATTGGCAGCTCATGTTCGCCGAGAATTACTTCGCAGAGGTATGAAGTGGGCTT 61580
                * * * * * *    * *** *** ***  ** **** **      * * ***   *  *

Human           GTAC-----ACAAAAACCGTTT--TCGACGATCGCGAAGCC-TGTC----CGTGAC---- 57035
Gallid          TTTCTTCCGATGAAGATGAATTGTTCATCGATAGTATAGCTGTGTTAAGTCGTGATGGTA 61640
                 * *     *  ** *    **  **  **** *   ***  ***     *****

Human           -ACACAGAATACAAAAACGACCTGA---TCACAGAGAAAAAACAAA-GCTTTACTTACAA 57090
Gallid          TATGCAGTGAACGTGAGCTTTGTGGGCTTCTCTGTCTGGAAACGTATGATCCGGATATAG 61700
                 *  ***   **   * *    **    ** * *     ****  * * *     ** *

Human           CTGAAACTCCACGATCTACA--CGCCGTCTTCAACCTGTTTCCAGAATACGAACAGAAAT 57148
Gallid          CAAAGTATATGGTACCGACAAATGCAATTTCTGGGTTAACGATACAGGCCGCATACGATT 61760
                *  *   *     * * ***   **  * *      *      * *   ** * *  * *

Human           TTCTA-GCGATCATAAAACTG------CCAATTACCGGTAAAGAACCCATCGACGTCCC- 57200
Gallid          TTCCACATGATCGTACAATTCATATACCCGAATTCCCTATAATCACGGACATATATTCAG 61820
                *** *   **** ** ** *       **  * * **    **  **  *   *  * *

Human           ATTCAGTCTCAGCAATCACCATCAACACACCTGCCTAGAATTCT-CTCCCTACGCTAACG 57259
Gallid          ATTTAGTATATGAGTGCAGTCGTGATGCATTTGTGCTAACTTGTGCAAGATTGACTGAAT 61880
```

```
                    *** *** *  *     **       *  **  **      * ** * *     *   ** *

Human               AACAAATCTCCAAAAGCGCCTGCCTACACTGCGAATCCGTCTCCGTTCCAACCTCCTCAG 57319
Gallid              TACCTAAGTCTTTGAGTGACCTCGTTGAAGGATTATTTGATGGCATTCCCACTCCTAGAG 61940
                     **  *  **    ** * *  * *  *  *   **  *    * **** **  *   **

Human               ATGCA--ATGGTCGCCCACTTGAACCAAGTCA-CCAACGTCAT-------GCAAAACAGA 57369
Gallid              AAGCATTATCATCAGAGATATTCGGCAGGCGAGTCGATGTTATAGTTACAGCAAAGAAAG 62000
                    * ***  **  **    *  *    ** *  *  * * ** **       *****  *

Human               TTTTACTT-CTACGGCTTTCGAAAGGACATGGAGC-TGATAC-GCATGTCCGCCAAACAA 57426
Gallid              CTGCAAACACGACGACTGTGCAACGCGCATGGGATATTCTACAGCATGGATGCCGG--GG 62058
                     *  *    * *** ** *  ** *  *****    *  *** *****    ***

Human               CCCACTATCTTTCAAATCTTTTATATTGTCCACAACACGATCAACAACATATTCCCTATC 57486
Gallid              TAAATTGAACGTAAACAGTAACAGGACAAATCCAGTACAACGGAAAAAACATGCTCGGTT 62118
                      * *     * **   *   *         **  ** *   * ** * ** * *  *

Human               ATGTTCG---AAAAAAAACAAAAGCTCGGAATGCACAT---AGTTTTCCAGTCTCG---- 57536
Gallid              CAGTTCTTTCGTACAAATCAAATATATACCACCCGTATTTAAGATATGGAGTTGCGATAC 62178
                      ****      * *** ****         *  *  **   ** * *  ***  **

Human               CACTCTGCACATTCCTTGTGAATGCATTAAACAAATAATAGCGGTATCTTCGGGCTACAA 57596
Gallid              CAGCTCTTGTATGCCCTCT-ACTTCTCTAAATAAGTTATGG-GAAATCTTCTGGAAAGTA 62236
                    **        ** ** * * * * *  **** ** * ** * *  ****** **  *  *

Human               CGTTTA--TCTAGATATACTACAAGACAGTGTAA-TCCTAACTGTCCTCTGCGAAACA-- 57651
Gallid              GACTCGGTTTTTAATATGAACATGCTCAATTTAAATTCTAATCTCTATACGGAGAATGGA 62296
                       *    * *  ****         ** * *** * ****       *  *   **

Human               -CTAGATACTAATACTAA--TATTCACATTGACATAGGGATGCTTCAAAAAAAGCTCGAA 57708
Gallid              TCTCAATCCGACCTGGAAATTATACAAAGTGAGTTGGGGATGATTTCAAATGCTTTATTT 62356
                     **  ** * *     **  *** ** * ***  * ****** **  ***      *

Human               GAAATGGACATCCCAAACGAAATCAGCGATAGACTCGAAAAATACAAAGGA-CATCTAAT 57767
Gallid              GGAAGACACGCATCCATGTTTGTTGGAGTTGGACCAGAAAACAAAAATATATCGCCCAGT 62416
                    * **   **    * *       *  * * * ***  *****  * **   * *  * * *

Human               AGGTTTCCATTAAACCACTTTATTGTTAACATGTACAAACGACAAAACCGAAATACGTTC 57827
Gallid              CAGAAGTTTTTG--TTGCTTCAGTATATACATATATTAAATCGTCTGCCAAATTGTTATG 62474
                      *      **      *** * * *  **** **  **         ** ** *    *

Human               ACATTACCTCTGCAATAGCAGAAAACGTTTCATCCAAATACACTTTCATAGGAAGTAAAT 57887
Gallid              AC-TTAATTCGCGAGTTGT-GCGATCATCATACCT-------CTACCATTGAGGGGGAAT 62525
                    ** ***  **   * * *  *  * * *   * *        **  *** *   *  ***

Human               ACAATAGTAACTTCTCTTTTTTTATATCGGTCTTCGGAAAAATGGGTGAAATCGCATTAG 57947
Gallid              GCCCTACTGCCATGCCCGACTGTGCTCTTGCAGACATGACAAACAGTTTATTCAGAGCGA 62585
                     *  ** *  * *  *    * *      *    *   * **   **  * **  *

Human               TCACAGC-CTTGATAATCTGATCGAAATACTTCTCCGCATGTATAGGAATCTTGT-GTTC 58005
Gallid              TTTTATTTCTTGGCATGGCTACTGAAATAGTTGTGAAC-TGGATGCCTTCCTCATTGGAA 62644
                    *   *   ****  *     *  ****** ** *   *  ** **      **  * *

Human               TAACACATAGTTTGGATCTTCTGCTAATTCATAGTT----ATGCGTCTG-TTTATTACCA 58060
Gallid              GAGCCCACATCGCGAGTTCCTAGTCCATCAGCCTTTGCAGACGCGACTACTTTATTGGAT 62704
                     * * ** *    *  *     *   **       **    * *** **  ******

Human               GTAGATGGTGCTATTA-ACACATACATAATTCGGTCACCAACATTCGGAATTTCTTCCTT 58119
Gallid              GTGGCAGAAACATCTGCACCAAAATCTATACAGGACCTCAAGATACGTAAACTGGCTCTT 62764
                    ** *  *   *   *  **  * *  **    ** *  *** ** ** **  *    ***

Human               TCTCTGCGCCAACCTTCTAATGACGCTAAGGTGAGCCAGATTCGGCTGCTTATAT----G 58175
Gallid              ATTCTAGACGGTCTATATAAAGACATTGATCCCATAGACGTCGCGTTACGAGAATCGGTG 62824
                      ***   *   *  * *** ***  * *    *   *  *   * * *    **    *

Human               CAGCCACCTCCTTGGAAAGCACAGAAGACAACATCAAATGTCTCACGTCTGCCCGATTTT 58235
Gallid              GGAGAAGATACGGCAGAATTGCTATGCGCGGCCATAGATATATCAGTTTTATCAGCCTTT 62884
```

```
                         *  * *     **   *      *  *   * ** * ***  * *  * *  ***

Human               GAAAAAGCTCCTCCCGCGCTTTGCACAGACGACGCAAAATTTTATGAATACCCACAGGCA 58295
Gallid              GAACA----TTGGGGGTATTATAGTCGATACATGCAATGTATTAT-ATCATTGATAGACA 62939
                    *** *          *   * *   *     * ****  * **** *  *   * ** **

Human               CTCCTTGCTCACGCAACTGTGTTTGCGTCATGTGAGAAAACTCCACAGCAGCAGTCTGA- 58354
Gallid              CTC---GTTTGAGAAACAGTGGGTGTATTACAATATGTT-CTTAATCGAGATAATACGTC 62995
                    ***   * *   * *** ***  **  * *    *     **  *  *    * *  *

Human               ACCTCTTCATCAAAGAACAACAAGTCCACGATGTCTTTCACCACACCTTTCACAAAATCG 58414
Gallid              AAACCCTCATAATGCATGGATCTATTACCGCTGTAATAC-CTGAAATGTGATCGGACTTG 63054
                    *   * **** *   *   *    *   ** ***  * * *   *   *   *  * * *

Human               CAAGAAGTCTTTCTCACCAGATCTACCCCCTTAAAAATTAAAAGCGAGTCATCCTGTCTA 58474
Gallid              GGAAAAATCTT-CACGCGAAATAAATTCT-------ATACATAGCTTGTTATTCCCAAAG 63106
                      * ** **** * * * * **  *  *        **  * ***  ** ** *

Human               CCAATATATCTTTTTTTACAAATCAAAATGAGCGGACATAAAATCTTCTCGAACTCGAGC 58534
Gallid              GTTCTTTATTTATTTTTGCAT-TAAAGATCGTCGTACAGGCTCAATTTCTTATATCTA-- 63163
                        * *** * ***** **  * ** **   ** ***       **    *  ** *

Human               TTGATAGGCGATTTGAACAAGCGATCTGTGATATGTTTGGCGATCATCGGCGCAATCCTC 58594
Gallid              -TGATAATTGTATCTA--AAATTATTAATAATCATTCTTACAGTAATAATTGCTATTG-- 63218
                     *****   *  *  *  **   **   * **   * *  *  * **    ** **

Human               CGCAGAGACTCATTAGCCATATTTCTGACAGACATAAAGATGCTATCCGTATCACCATAG 58654
Gallid              -ATATAGCTATCGAAGCCCCGGCTGTAGCAGTTGC---GACGTAAGTTGCAGAGATAATA 63274
                       * **       ****     * *  ***       ** *  *   * *     *

Human               ATTACCTCCACTTTTAAATCACCAGTAAAATCTGATGCCGTTAGACCCAATTCTTCGCAA 58714
Gallid              GTTACTCG---TTCCGATTCAAAAGCTAAGATCCGTACGATTGTACC-ATTTGGGTACAA 63330
                     ****      **   * ***  **  **      * *  **  *** * **     ***

Human               AAGAATTGCTCGGAC-TGCATCTTGGAATTAACATAATCCAC-CGTGGAACAAAGCATCT 58772
Gallid              TAGTAATGATGTTCCGTATATTTGGGCGATAGTAGGGTTAAAATACCGAATGGATGAATT 63390
                     ** * ** *    * *  ** * **   **  *   *  *      ***   *  *  *

Human               CTCTTCCAAGACAGGTTACAGAAGCAGCAATCGCAAC-ACACGGCAATAACCCGTGCGCC 58831
Gallid              CCCTCCTGCAATCAGTTCTCGCTGCAAATCTTGTGATGAAATATAAATGGTGTGTCTAAG 63450
                    * ** *    *   ***   *  ***    * *  *  * *    ***     **

Human               GCTCCCGTGACACCGTACACCGAGTTACATGTTGTTTTGAGAGCGAGCTGCTTTTTATCC 58891
Gallid              GTTTCCGTT-TGTTGAGTATCTCA--AAAGGACACAACCACAGTAGGG-ACAATCGCTTC 63506
                    * * ****      *   * *     * * *        * **   *   *  *   * *

Human               AGAAGGAGTTTCATCATTGGATCCGAACAGTTTTGCATCTCCGCCTTCACTTCTCGTCTC 58951
Gallid              CGAGTGGTCTAGGTAGTACCATGGGAACAATGTTAC----CGGCTGTCGTTGTACA-CGG 63561
                     **  *   *   *  *   **  ***** * ** *    * **  **  *   *  *

Human               TTGGCCAGCCAGTCCTTTAGTAGACTTCCAAGCACAGACTCGCGAACACAAG--GCTTCA 59009
Gallid              TAGTCTAACATATGTTATA-TAAAGTTGATTGTTCA----CGTCAACACCATCCACATTA 63616
                    * * * * *   *  * ** ** * **    *  **    **  ***** *    * * *

Human               CAAACCGATGGGTCTCATCCCCCAACTTCACGGTTAAGATGTCACTCTCTGATAATCCAG 59069
Gallid              TAGAC-AATACCTCTTATTCCA-AGTTGTCTTGTCACAACATAACTCCC---TAGTCCAG 63671
                     * **  **   *** ** **  *  *     ** *  *  * **** *   ** *****

Human               CTATTTGCCGTTCATCCAAAACTA-AGGTACTATAACAC--AGATTATGCGCCATCATAA 59126
Gallid              AAATGGGCAAAACTAAAAGCGCTGTAGCTTCTAAAATATCTGCATCAGAACAAGTCACAA 63731
                      **  **    *    *   **  ** * *** ** *     ** *       *** **

Human               TGCTCGGATACAAACTCTGAAAATCAAA-CACCA-CAGTGGGCACAGCATAATAAC--CC 59182
Gallid              C-TTCTGATGCATATATAGTCAACCTTCTCATCAATGGTGTTTTAAGCTTGGTGATWACC 63790
                       ** *** ** *    *  ** *    ** **   ***     *** *  * *   **

Human               GTCTTAGGCTCCAAGACGGTAGCCCCTTTGTAACCTATCCCTTGT---CTATTATGCGAA 59239
Gallid              AATTCYGCYTTTGTTTCTAATTCTTCTTCAGCATCGATACCACGGGAATCATTTTCCAAA 63850
```

```
                   *  *  *      *     *  ***    * * ** **  *      *** * * **

Human           GACACC---ATACTCGGA---AGAATCATGTT--ACGACGTTTTGCTTCCGTAAGAATGC 59291
Gallid          TACTTCTCGATTACTGGATGTAAAATCACATCGGACATTGCATATAGTCTATGATGATGT 63910
                 **  *   **    ***   * *****  *   **   *  *    **  * *  ***

Human           AGGGAAATATCTTCTTCTGCTGACCCTCGA-ACACCACACATCTAGCCGTGACGTGTGCC 59350
Gallid          TCGGAAATATC--CATCCGCAAAGCTGTAACACACGGTGTATATACATCTAAAATATGC- 63967
                  *********  * ** **  * *    * ****     ** **    * *  * ***

Human           AATCTGGCGACCTCGGCAACCTCATAATGATAATTAAT--CTGTTTAAAGAGACGCACAA 59408
Gallid          --ACTGGCGAGTTTTTAACATTGATTTTGATATACATTTCCTGTATGACCAGTTCCGTAG 64025
                   ******* *    *   * **  *****   * *  **** * *  **   *  *

Human           CTAAGACAGAGTCCT--GCAGACAAT--ATTTACCGACAACAG--CCCTGCCACTGG-GT 59461
Gallid          CTACGATATGTTCTTCAGTACACATTGAAGACGCAAACAATAGAGCCTTGCGAGCAGTGC 64085
                *** ** *   ** *  * * *** *  *    *  **** **  ** *** *   * *

Human           CCGCTA-ATAAATTTCTTTGGTATTTCCTTGTACGATAACTGCTCTTTTTCTTGCTGGAG 59520
Gallid          GAGATGCATCCCATTCATTCGGAGGGCGTTGAAAAATACTCGCAAACGCA-TAATATAAG 64144
                  * *  **     *** ** * *   * *** *  ***   **        *      **

Human           ACAGATCTTAGCAATAGTATCCAGTTTGTAATTCTGCGCC-GTTATCTTACTAGAATAGA 59579
Gallid          GCATGCCTC-GCAGAAGTGTTCCAT--GCAATTGTGTCCCTGACGGATTCCTCGTGAAGA 64201
                 **   **  ***  *** * *  *  **** **  ** *     ** ** *   ***

Human           CTGGATACATATCCAGATATAACACTCCGGAAGTAAACACCTTGGTTTGCGCCTGAAGGA 59639
Gallid          GCTATAATGAGCTCTAATGAAATGCTCGAAACATGCTCCCCCAGATTTCCACT------- 64254
                      *      *  **  **  ***   *  *   * **  * *** * *

Human           ACCCCTTCTTGTACTGTTCATGAGGA-ACAGAGATTCCAATCTTTCCATTCTTCAGTTTA 59698
Gallid          --CTCTTGGGATATGTTGAATGCATACGCAGACCTTAAAACATATAGATGTTGCAGAATA 64312
                  * ***    **   *  ***   *  ****  **  **  * *  **  * ***  **

Human           GAAAAACAACCAATCTCATAGTGGTAAATCTTATCCATCCTTATACACAAATATTTTAAA 59758
Gallid          GATAAGGAAGCTGTATTTTTGGATGCATTTATAATCGTCTTTCGAATCAAAGATTTTATA 64372
                ** **  ** *  * *  * *     * *  **  * ** **  *  **** ****** *

Human           TCAAAATTGTTAATATTATAACCGGTAATAAACTCCGGAGACTCAATCCTTAAAAATATA 59818
Gallid          ATATCAGCATTTTCATT----CCGCAAATGCATTCCAGATAACAAG-----AAGTATGTA 64423
                  *  *   **   ***    ***  ***  * *** ** *   *      **  ** **

Human           AAAAATCCCAAAAGTAATTCAAACTCTGACGCAAATTCA-TATATATGCACGCCGTCAAT 59877
Gallid          CCAGGGTTTGAATGCAAGGCAAAAGTTGAAAGAGTTTCGGTAATCATTTTTATATTAGCT 64483
                  *       ** * **  ****   ***   *  ***  **   **        *   *

Human           TTGTTCACAGGTTCCCAGAGTAAACAGATGTCTCTCATCACGGTCGCCCTCAGTATCAAA 59937
Gallid          TCAGTTTTAATTATTTCATCGAAAGAAATATAGCCAGATTGAAACGCGTTGCGTACAGAT 64543
                *   *   *  *         *** * ** *  *          ***  *  ***   *

Human           ACTAACTACAGAAATCTGAATAACTATATCACCCAAATTTTCGGCATCCGGGAAATTCCC 59997
Gallid          GCTACT---AGAGACATAAATAAGCGTGT-------AGTTACGACTCTAAAACAATATAT 64593
                 ***     *** *  * *****   * *       * ** ** *        ***

Human           ATTTTGGCCCAAACACTCTATGTCGAAGGACCAGCATCCATATAAGGGCCAATTGACGTT 60057
Gallid          ATCATT-----AATATCAGATATCGGTGTGGCAGCAAATATAAAAAGCTGGGCATGCGTT 64648
                **  *      ** *    ** ***  *   *****   *** ** *         ****

Human           TTCCAAAGAAACTA-AATCCGAGACATGACAGTTAATTTCCACCT-CAAGATTACTCCCT 60115
Gallid          T---GAGCAATCTGGAAATCATAATTGGATGATTCCTCTACACTTTCGATCATATTAATA 64705
                *    *  ** **  **  *   *   **    **  * * *** * * *   ** *

Human           TTTCCCATATCTTGAGGAATATATTTTTTTACATTATACCATCCGAAACTCAAAAAACCA 60175
Gallid          TATGATTTAATTTCCATATCCGATGCGTTCATAT-GTGGTCCTAGCAAATAAATCAAATA 64764
                * *    **  **    *    **   ** * **  *       * ** * **  **  *

Human           TTATCGACGAAGAAACGAGTCAAAACATCTACATCGATTTCATAAACTACGAATC--CCT 60233
Gallid          CGGACGTCGAGTAAACTTATCCACGGGTTCATAAGGTTTCAGCGCTGGCCAGATCAACGA 64824
```

```
                   ** ***  ****   ** *    *  * *  * **          *  ***  *

Human           CATTCTGCAGAATCTTCCCAATACGTTGAGATACATAAAAATTTCCAAAACTCACTTTAA 60293
Gallid          AATATTTCTAGCTCTTTCCGATAATATCAATTCGACGGGAGGTGCCGA------TTTCAT 64878
                 **  * *    **** ** ***    * *  *  *    *  * ** *       ** *

Human           ACAGATTAACAACAGTGTTGGCATTGTACCCATACA-ATGACAACTTATCAGCCGGCTCA 60352
Gallid          AGAAATTGTAATGGAGGCAAATGTTATCCCTATCTTCATTATAACTTCAGCGCCATCGGA 64938
                * * ***   *      *      ** * ** **    ** * *****    ***  *  *

Human           ATGACAAAAGAACACG-ACATCTTAACCTCGCCGGTTAACATCAAATTGTTTATAGTAGC 60411
Gallid          ACCAAATATAATCGTTCGTATATGTGCATAATCAACCGGAATGAGCATGTCCATGGGATC 64998
                *  * * *  * *      ** *   * *   *       ** *   ***  ** * * *

Human           CTTCAGGCTTTTTCCGTCGACACAT-TCGCAGTAAAAATATTGCTCCTGTCCAAAC-ACG 60469
Gallid          CACAAAACATACATGTTTTACATAGGTTCCGGTCAATTCACTGGTGAGAATGAAACGATG 65058
                *   *  * *      *  *** *  *  * ** **   * ** *       **** * *

Human           TTCAC-ACAAATCTTCTCACC-GTCCTCAGATCTCCCAAACATCCTAATCACT--GTCCC 60525
Gallid          TTCAGGATTAACAACATCAATAGGTTTTAGGTTCTTCAATTCTGTTGGAGGATTAGTTCC 65118
                ****  *  **     ***   *   * ** *    ***   *  *       *  ** **

Human           CGAAGGAATCACAAAATGTCTATACTGAAAAGGAAGATTCTCGATCGAATCCGTAAACAT 60585
Gallid          TGTAGGTTGAGCGTCGTCTTTGTCATCTAATTTAAGA--CCAGATCGGTCCGAGGGAATT 65176
                 * ***      *     * * * *  *  **   ****  *  *****   *     *  *

Human           TA-ACGTTTCTACAGCATCATAGATGTGGAACTTCATCCTCGTATGATCATAAT----CC 60640
Gallid          TGCACATTTCGTCGGTAGGGT-GGCATGGC-CGTCATGAACGAGTGATTGATGTACGACC 65234
                *  ** ****  * * *    * *   ***  * ****   **  ****      *    **

Human           TTGGACC--GAACTCTGTCTAAACTCGGCCAAGTCATTTCTTTGCTCAGTAAATACT-GT 60697
Gallid          CCGTATTTAAAAATTTGATTAGATTATTGGACGCCAACAGTTTGGATACCGGCCGTTTGT 65294
                  * *     ** * **  ** * *     * * **    ****   *         * **

Human           CGGTCTCGATAAAACATACGCGGTTCAGAGTCACA-AACATCCTTTATTAAGCCCGCAGC 60756
Gallid          AGAAAGTCATTTGTTGTACATGACTTGTTGGCATATAAAACAACACACCATTTGCTGGGC 65354
                 *      **      ***  *  *    * ** * ** *      *  *    *   **

Human           ACCATCATGCATTATACCGCGAGGAAGTATACGAATGTAACTCGATCTGCTTTTTTTCTT 60816
Gallid          TGGATGCCCCAATATACACAAATGTAGTATTGTCACGTACCCAGGA--GCCTTGGTTATT 65412
                   **    ** *****    * * *****    * *** *  *    ** **  ** **

Human           TAAGCGATTCGCTTCCAAATATGGATTAAAAAATGACACCGAATCCATCACGCTTCTTCT 60876
Gallid          C--GCGCTTC-CATCGAGAGATAAAATATTCAGTCGCAGAGAAGGCAGATCTGTAATTCC 65469
                   *** *** * ** * * **  * **   * *  **  ***  **   *  *  ***

Human           ACATTTACACTCTTATAACCTCTATACCTGATCCTGTCTATCAAACTAGGTTTTGAGGCA 60936
Gallid          CAACAAGGACAATAACGATGGTAGTGTAT--TCGTTGGTGTCTTCTTTGCACAAAATGCA 65527
                  *     ** * *  *       *   *  ** *   * **    * *      * ***

Human           TGAGACTCAGAAGACGAAGGTTTTCTTCTGT-AGGACTCGT-CTAAAGACTTTATGGCCT 60994
Gallid          TGGATCATTATCAAAAAAACTATACTACCGGGAAGACCCATGTTGGATATGTAGCCATCC 65587
                **   *       *  **  * * ** * *  * *** * *  *  * *  *      *

Human           TGAGCATTTTTAAAGCATC----TTCTTGTGAATAT--TCATCACCACTTATTTGTCCAC 61048
Gallid          AATGCAACTTGCAAGTGCTACGATCCGTATGAACACACTTACTAAATCATAGATATTTAT 65647
                   ***  **  ***        * * * **** *   * *  *   * **  * *  *

Human           TGACGTCAGCCATACCCTCCTCTTTTTCCGAAACCGCAACAGATGTGCCCCCGTCAGCAT 61108
Gallid          TGGATCTAAGGACGCCAAATTACTATCTTAATATA--AGTCAATATTGCCGCATCATTGT 65705
                **      *   *  **     *  * *     * *     *      ** *  ** * ***   *

Human           CTTTAACACTAGGCGTCTTGACGACAGTGGTCCCCGTAAC--ACTGGACACAGTAGTCAC 61166
Gallid          GATGTGGGCAAGCTAATTATAGAAGCGCGATATGAATGGGTAGTTTTACATAGCCATCTC 65765
                  *     * **      *  *  *  * * *     *       *  *** **   ** *

Human           CGGATTGGTGGC--ATAAGGAAACATCA-TGTCAATAGGCTTACTA-AGCACATGTTTTT 61222
Gallid          TTTATTATAGGCTAGTACATCAGCTTCAATCTCGAAGGCATCATTCCAGCTCATATGTGT 65825
```

```
                                ***   ***   **    * * *** * ** *  *  * * *  *** *** * * *

Human           GTCTAACGAAAAC--CACAATTATTATAACGACAACTACTATCGCTAAAATCAACATGAG 61280
Gallid          TACCAATCTCTCCGACATAGTATCCATGAACGATTCTACACACTTTGTTGTGGACAGGCC 65885
                  * **       *  ** * *    ** *      ****   *  *    *  *** *

Human           ACCACCCCCGAATGGATTTTT-TAAAAAAGACACCACTCCACCCACAATATCCCCAAGTG 61339
Gallid          AGTAATATTAAATCGTTCGACGTTAATGTTCCGCAGTTTCATTAATAATCCCCACAA-TA 65944
                *  *      *** * *     * **     * *   * **   * ***  ** *** *

Human           CTCCTGC-CGTAACACTTATAACCGAGCCCAATCCAGTGCCTATAGCCCCAAGCCCTTGT 61398
Gallid          TCCATGCATGCGTTGTTGATAAGCTA-CCATCTTCGGCACATAATTCTCGCCGCTTAAAT 66003
                  * ***  *      * **** * * **   * * *  * **   * *   **     *

Human           AA----AAAAGAATTAATCCCATTGACATACGACGGCGTATTAGTAGCAATTTTAGCTTC 61454
Gallid          ATCGCTAAAAGCGTATGGTGTAATGACACCCTACTAC-CGCGATAATCAGTACGAGGTTC 66062
                *     *****  *        * *****  * **  *      *  * ** *   ** ***

Human           TATAGTATATAGTGCGCTCTTATATGAGTTATATTCACGAAGAATATTCTCTAAATCGAA 61514
Gallid          GTTTATAAACGTTGTGGCCAAAGATGTATCAAAC---CATGGTAGATCCAACCATTC--A 66117
                  *  ** *   ** *  *  * ***  * * *     *   * * ** *    * **  *

Human           AACGTTTGCTCTACTCAATTCGTCCGGCGAATATAAATCAAGTAGTTTAAAATCAGCATT 61574
Gallid          GATTCCCATTCATCCTTACTGTAGGGACAAATATGATTTGCGTACATAATAGTGTCTACT 66177
                 *        **  *   * *     * * ***** * *   ***  * * * *    * *

Human           TTCGAGAGGGTCGATCTTTAGTCTAATAAAAGCATCCAATACCTCAATGTCTTCTATGGG 61634
Gallid          AAACAGGAATACACTGCATTAAGTGCCCTGAGCATT-AGTAGATCTGTTTTCTCGCCGGT 66236
                    **      *  *   *    *      *****  * **  **  * *  **   **

Human           CGTCGAATTCGTATGCGTATAATCGGTATACACGTGTGCATGATTTCCAGATAGAAATAT 61694
Gallid          CCTATTCCGTGAAGATAGACGGCTAACATGCTCCTTTTCGTCATGT--AGATCGACA-AT 66293
                * *       * *     *         ** * * * * * * * ** *  **** ** * **

Human           CTTTGTACTAGGTATCTCACATTCCT----CTGTCCTATGATCACCCAA--CAGAATCTC 61748
Gallid          CACCAAGTTGCATCCTGGCGGTTCCTGGGGCAACCTTATTATCATACTAATTAGCATCTC 66353
                *       *   *        *****    *   * *** ****  * *   ** *****

Human           --ATTATCTAACCCTAGCTGACCAGGGA-CAACCTCAGG-CGTGGAGTTCACAAAGCTAA 61804
Gallid          CAAGGAACAATCTCCAGTTAAATGTCGAGCAATGGGAAAACATACGGTAGCGGATATGGG 66413
                  *  * * * * * * ** * *     ** ***     *   * *   **     *

Human           ACGTCACCAAGGGCCGATTATAACACATCGTTTCACTCCTTACTCCCTTCGCATCGACGA 61864
Gallid          ATGTCGATCTAGAATTAATATAA-GCGATGGATTGCCACGTGTTCCTGTTATG-CGATGA 66471
                * ***      *    * *****  *   *  *  *  * *  ***  *     *** **

Human           CCCGCATACTCTTATGAAGCTGAACGGATGATTGATTAACTTCTATGCATTTCGAGATAG 61924
Gallid          CACTTCGACGATAA--GCGCTCATGAAATACAAGATAGGGATCTGCAAACTTTG--ATTG 66527
                * *    **  * *    *** *    **    ***      ***    * ** *  ** *

Human           CTAACACATCACCATGCAACTGTGCAGATATCGGACGACCGTAAACCTCAGAC-ACGAT- 61982
Gallid          TAAAGCTGTTACAATCATGCTAGATTGAAATAAAGAAAACTCTCCCCTGCGACGACGATC 66587
                  **    * ** **    **      ** **       * *     ***  *** *****

Human           ACTCG----ATGGACTGATCTTACTAAGTTCGTGCAACATCGTTATCGTTCGTTTTTGA- 62037
Gallid          ACATGTATCATTTACGGTCGTGACCAAATCAGTAAAATAATACCGCCAGTATTTCATAGG 66647
                **  *    **  ** *   * ** ** *  **  ** *        *  *  **  *

Human           -TCGAGGCACCAAGATTCTGCCAAATTCCCCAAGGCAT-CGTTGATATAATCTTTCAAAG 62095
Gallid          TTCGAAAACCTTTAGTACGGGTACTCCCATTAAAGAGTGCGTCGGTATCTCATTCAACAT 66707
                 ****    *     * * *  *     *   ** *  * *** * ***    **  * *

Human           TATCATAG-AGGTACTGTAATTG-CACATAAAGAATATCATGTCTAGACTTGACATCCAC 62153
Gallid          TGACGTTTTACCTATACCCATTGATCCATCGAGGTATACACGTATGAGCTGTGCAGATGT 66767
                *  * *   *  **     ****  ***  **      ** ** *   **   **

Human           CA-----AATCTCGCCTCCTACGTAAGTTT--ACTGAACCCTGCTCAAGAACCATAAGAG 62206
Gallid          CATCTGAGATGCCATTACCGACCACGATTGTGGCTCCGACATGTTCAATGGGAGAGAAGA 66827
```

```
                          **      **  *    ** **     **     **     * ** ****        *

Human                ATTTTTGCACAAGAGGCTGCCAAATCAAAATCAGATCTCCCGTAGTCTTAAAAATTTGAT 62266
Gallid               ACGTACTCAAAAGATATCGC--AGACAGGTTAGTAGCACTCGAAATTTCAAACGGCATGT 66885
                     *  *   ** ****    **  *  **   *   * * * ** * * * ***       *

Human                AACTACCGTTCATGCT-GTATGTATCATT-ATAATCTGACATATAGACATCTGTAATTAT 62324
Gallid               AAATACGAATGTCCTTAGAAAGCGACGTTTAGCGGCTGGTGT-TCGATGTC-ACGATCGC 66943
                     ** ***   *     * * * *   * ** *    ***   * * **  **    **

Human                CTTCTCAAATTCATTCTTA-ATACACGTT-TGTTTGGGATCGGTAAGATTTAAA-GACTC 62381
Gallid               TTTTACAAACGGCTTTATGCAGAAGCGATGTGTCTAGGATCTCAGGTGTATGATTGGCCG 67003
                      **  ****    **  *  * *  ** * *** * *****       * * *  * *

Human                CTTGGAGGCGACG--AAAGCGGCTGTCAACTCCTTAG----AAATAAAGTGATAAGTCTC 62435
Gallid               GGGAGAAGCTTTGCCAAAATTTTTGGGAAGGTCATGGTATTAGATGTATTTAAACAGTTG 67063
                         ** **   *  ***     **  **   * * *    * **  * * * *    *

Human                ATCCGTCTCCGCTCGAAGCCCGTGAGTCACCGTAGTCCAGTG----CTTTAGCATACACA 62491
Gallid               ACGGATTTTCGATTGGTCTTCG-AAGTAAACCTAGAACGACGTAGACCGGATTGTATTTG 67122
                     *    * * ** * *     **  *** * * ***  *   *    *   *   **

Human                CCGATTCATTCTCTTCCTTGATTTCCCAAGAA--------AACAAAGTATCC-CCTTTCT 62542
Gallid               CATGTTTAAGTTGCCTCGTGATTTGTGGGAAGTAGGCTGTGATGGAGTATGCGTCATTCT 67182
                     *   ** *   *    * ******      *          *   ***** *  * ****

Human                ----CCAGAAAA--GCGATCTTCCTTACTAACGTAGTAGCCCCATTCATACCATTCATTA 62596
Gallid               AGAACTAAAAACTTGCAAATTTTCTCGTAACCTGAAGACACGCAGTAAACATGAACAA-A 67241
                         * * ***   ** *  ** **    * *  *  *  * ** * *       **  *

Human                GATCTTCTATCATAGTATAGTTTTCCAAGATTGTCAATTTTTCTAACGGCTCTGCAAAAT 62656
Gallid               GACTCACCGGCATAAAGCAGTTATT--GGATTCCAAATCTCT-----GATTGGGCAAATT 67294
                     **    *   ****    **** *    ****   *** * *      *  *  ***** *

Human                GTTTACCGTTTGAACCGTCGAAGAACGGAGACCCTTCCACGATTTCACCAGTCGT--CAA 62714
Gallid               G-----CGCCTCAAGGATCAGATTGTATCGTTATTTGTCCAATGTTAGTATTTGTAGCAA 67349
                     *     **  * **   **  *        *    **   * ** * *  * * **  ***

Human                AGCAAAG-----TAACTAAACGGATATTTCGCCTTAGCCGTAGCCTCCGTCACAATACAA 62769
Gallid               GACGAAAACTGTCTGTTCTACATGTGATGTGCCTAAAGAAAAGGCACATAGTGACTGATT 67409
                       * **          *  **   *  *  **** *     ** * *       * *

Human                TTGAGAGACGTCGATGTAGAATAGAG-CCACAAAGGACCCCTTGCAAAGTAGGGTTCTTT 62828
Gallid               TTCATAGACTATGCTCTATATTGGCGACCGCGTCTGAC----TATAAAGCACGGATAACT 67465
                     ** * ****   * * ** * * * * * ** *    ***    *  **** * ** *   *

Human                CGTAGTGATAAATCTTTTAT---TAGTAACAGACTTGAAATTCAGAGGAAATAATTCTAG 62885
Gallid               GACACTCATAAAATTTTAAAACGTCATAATACAAACACGATACATATACAACGACGCAGT 67525
                        * * *****  *** *    *  *** * *      ** ** *   **  *  *

Human                AGTTTCGTTTTTATTATTATC-CTCATGATAGGCACTAAACACCGTACCATCGGGTCGTT 62944
Gallid               GGAAAGGCGTCTGCTAACGTAACGGATTTGAGGAATCATATAGCTTGTAATAAAACGGCT 67585
                      *    *  * *  **   *  *  **   *** *  * * * * * *   **      * *

Human                TTATCGCTACGGCTGAATAACACTGCGCACGAGAATTAACTAAATTTGCTTCGTACACCG 63004
Gallid               ACTTTATCATTTTCAAATAAT------CAGGAGAG--AACGGGAGGTACGGC-TATGCGA 67636
                        *    *      *****       ** ****   ***   *  * *  * **  *

Human                G-CATGGCCAAACCCATCACC-GTCCGATCCAGAAAATAAACCACACCCACATCACGGTA 63062
Gallid               AACATGGCTAATATTATCGCCCGTCTTGTTCAGAGACCGTA--ACACTTTTATAAGCTTG 67694
                       ****** **    *** ** ***   * **** *    *  ****    ** *   *

Human                ACTGGTTTGGAACGTCAGCTCGTTTTTATATGT----TCTCACTGGAAAAGTGTAGGTCT 63118
Gallid               TGTAGTTTA-AATGTAGTATTATTTTCTCATGTGGCGTTTCCCTGTAA---CTTCTGTCT 67750
                       * ****  ** **    *  ****   ****    * ** *** **     *  ****

Human                CGATAT-TTGTTTTGTAA-ATGATGAAAAAACCCTCCGACATCTTTGCATTAGACTTATA 63176
Gallid               GATTATATCGAGTAGCACCATCATGGCAAACTTTATTTGGGACGCGAGAATATTGACAGG 67810
```

```
                   *** * *  * * *  ** ***  ***            *     * **     *

Human           TGGCGAACACGAAATAT-CTCTGTCGAACCGCATCAAATCAGTGC-CTTTGGCAATCGAA 63234
Gallid          CGATGGAATGGAAATGTTCCCTGCCGATGTGAAGAACTTTATAGCTCCCCCATGGCCCAT 67870
                 *  * *   ***** * * *** ***   * *  *  * *  ** *         * *

Human           CAAATCCGAAAAGGATATTTGTGATTATAGC---CCGCTCTGATATAATCATCCGAATCG 63291
Gallid          CGAGTTCTGGAAGGA-ACCGGTATTTACATCTAACCGCGCCAATATGGAACGACAACTTG 67929
                * * * *   ***** *   **  *** * *   **** *  ****       * * * *

Human           CAATATATCATTAAAACACTATTCATCAAAAAGACAGCCAGGAATAATACTC-TCATCTT 63350
Gallid          CGAT-CATTACTGCACGAAACAATGCCGCCACTGCAGCATTAAATAATTTGGATGGTCAT 67988
                * **  ** * *  *  *        *   *   ****    ******     *  ** *

Human           GCTCATTCTCCC-TAACGAGATCTA---GTAGATTAGGCGTTCGAACAGAGTAACTTACT 63406
Gallid          ACCGACTCTATTGCAATAGAAATTGACCGCAGACTTCGACCAATAGAAGAAAAAATTGAA 68048
                 *  * ***     **    *  *    * *** *  *      *  ***  ** **

Human           CCTTCCGGCACGTCTACAGTCTCCCTCAACAACTGAGCTTGCAAATCACAATATAACAGC 63466
Gallid          CATATCGCAACAGCGTTAGCGGATCTGGAACATGCAGCGGCTGCAGCAGAACTTGCCGAC 68108
                * *  **  **  *   **     **  *  *   ***      * ** **  *  *  *

Human           GAATACACGTCTTTAAACTTTGTCATGCCACCACCGGTGTGAAATAATAGAGGAGACTCG 63526
Gallid          GCTGCAATGGATATTGCCGTTA--GTGCTGTTGAAAAATCTGGACAGCACAAGGAAGACG 68166
                *     * *  * *   * **    ***               * *  * * *  *  **

Human           TGATTAA-ATGTCAAAACG--ACATTTCCATT-CAAATCCTCCTCCTCATCTATACGTAC 63582
Gallid          TTAATATCACTTCGGCCCGTGAGATTCAAATCGTGAAAAATGATCCTTTGCT-CAGATAC 68225
                * * **  *  **    **  * ***   **    **   *  ****   **  *  ***

Human           AATTTTTAATCGCTTTCCGAAAACATCATTATATAGTGCAACAGAAAGTGTTAACTCTCT 63642
Gallid          GACTCTAATTTG---TCTGTGGATCTCTTGAACTTAGTCTATGCAAA----TAGGAATGT 68278
                 * * * * * *   ** *   *  ** * *  *    * *   ***    **    * *

Human           GATGAAATTCCACATTTTTTTCTGCATGTCATTT---ACATCA-GTTACACCGGAAAAGT 63698
Gallid          AGTGAACTCCGGTGTCGTTTTCGGCACGTGGTATCGTACTTTACAAAATGCTCTGGTTGC 68338
                  **** * *    *  ***** *** **  * *   ** * *    *  *       *

Human           TAAAAAACTCATTATATTCGCACACCATCCACACACTGGGATGAATAGTGCCCTCTATGC 63758
Gallid          CGACAAGCCCAGCGTTGCCAGAAAA-ATAGACTATCATGGTGGAAGAATGTCGCGAACGT 68397
                  * ** * **   *   *  * *  **  **   *  **  *** * ** *    * *

Human           ATCTCGCTAAATCT-TCCTTCATATGCGGTAACATTTCTGCGTGGTCGCAACCATACGCC 63817
Gallid          TTATTGTGACGGCAATCACATCTTTACAATCATGCGGACGTTTATATGTAGGCACCCGTT 68457
                 * * *  *   *  **     * * *  * *       *  *    * *  **  **

Human           ATTGAGATATTCGCCGGAA-GTGGATAGGCAGATTTATGAAAATCCGATAACGGACCCGT 63876
Gallid          ATTAT--TCCTCTCTAGAATGTGCGATTCTTTGTTTATATGCATTCTATGCAAAAACTGG 68515
                ***    *  ** *  *** ***          *****    ** * **     * * *

Human           TAACAATGTGTACATAATCTCACTTAAATCCGGCAAAAGCTCCAAAGGGAGCTTCTTCGG 63936
Gallid          GACTAATATTTC--TCATCCCACT--AACTTTATGAGTGCGATTGAGTCGGTTCCCACTT 68571
                 *  *** * *   * *** ****  **       *  **     **   * * *  *

Human           CATCAGATTATTTTTAATATAAAGATGTTCATCGTATGTACAGCTATCTACACGAAGAGA 63996
Gallid          ATTTGGAGCATCTGTCAACGAGACTTGCTAGTTCTGATAGCAGACAAAAATATG----GT 68627
                  *  **  ** * * *   * *  ** *  *  *     ***  *   * * *    *

Human           GCCATCCAAGTACATTTTTCTGAGAACGAAACCG-TTCATAATCTTCGATAAAGCCTCAA 64055
Gallid          TTCGATTGGGCTCGACTTCCAAAAGACACTTTCGACTCACCGTGTG-GAAAATACGAACG 68686
                  *      *  *   ** *  *  **     **  ***   * *  ** **  *

Human           AAATAAAATTCCCATACACGTTCACACTCACTACCTTATTGA-TGAGTGATTCCTGCTCT 64114
Gallid          GGGTGCATTACACGATCATAG-CATTTTAAGGGCTTTAGTAAATTCCCGAGTTTTGCCCC 68745
                   *  * * * *   **    **   * *   * *** * * *    ** *  *** *

Human           CGAATACATG--CCATCACCCTATTATAACCAGATTCAGAAACCTTTTGAAAATACGCCT 64172
Gallid          CCGGTGCGGGATCCCTTCCCCGTGGAGATGTAATTCCAGAAATTGATGCTGAACAAGGCA 68805
```

```
                *   * *  *  ** *  ***    * *   *  * ******    *    ** * * *

Human           TTTTCCGAGCCAAGACTTCCTG--CTGAAG-ATTATGAGTTTGAAGCATGCAATTGATCG 64229
Gallid          TACGCAACGACGAAGTTAACAGGGCTGCTGCAGCGTTACTGGGACGAGCACAACCTCTCT 68865
                *   *   * * *   *  * *  ***  * *   * * *  ** *    ***    **

Human           TGTTCA-----GCCTTCCCGGTGTTTCTGTCGTCTGTGGATCCCGA-ATGTCAT-GCAAC 64282
Gallid          TTCTCATGGAAGATCAACCATTGTTGAGGTCTACTATTGATACAATTACGGCATTGCTTT 68925
                *  ***     *     **  ****   ***  ** * *** *    * * *** **

Human           AGACTCTTAATTTTATGTTGAACCTCATCTTCC--TCAGCCAATCTTTTAAATATTGGAC 64340
Gallid          TACTTCACCGTTTGCTTTGGAACACTAATATCTATTCGGCCAGGGTAAAAAACAT---AT 68982
                    **    ***  * * ****   *   **   ** ****   *   *** **   *

Human           TTTCCTCGAGATTTTTAATGCTCAGACCAGTGACAAGATCTATCAACCTCGAA----GGA 64396
Gallid          TTCAGTTAGGAGCATTCGTTCCGGGTATAGTTCCA-GATTTAACAGTGGGGGCATCTGTA 69041
                **   *   **   **  * *   *   ***  ** *** ** **     *      * *

Human           GACACATAAATAGAGCCAACGTACAAGCGCTCGTCATGCGTGAGCAAATTTTCACTCAAA 64456
Gallid          GATACGCCGGGGGATATAAT-TAAAAGCGA-CGGCAGAAATTTGATGTTTTTATTTCAAA 69099
                ** **       **   **  ** *****  ** **    *  *   ****    *****

Human           GCCAAGAAGTCTTCAGAGACTTCACCGTAAAG---ATAAAGACTA-ACA--TCATTCTGT 64510
Gallid          GATATGTAGCTCCCATGTATGGTACTGTGAAAGGAATAGAGTTTACACAATTATTTCCGG 69159
                *  * * **    **   *    ** ** **    *** **  ** ***  *  *** *

Human           AATTGCTTATACAACGTGTGACAA--CTATTTAACTGCTGAAGCGTAAT---------AG 64559
Gallid          GGTTGGTAGCTTTGTGTTTGGACGTCCCACTTTCAGCGGAGGTATATTTAGTCATAGAG 69219
                  *** *        ** **      * * **  * ** ** *  ** *         **

Human           CAACATTTCTTACTATCGTTTCTGATATTTTAGACCAATACGTAAACTCACTTAAAGAAT 64619
Gallid          CACCATTATCACGTGTCGTTGATGTCTCTCTGGGTAAATATCAAGCCTCTCTTGTAAAGC 69279
                ** ****      * *****  **    * * *   ****   *  *** ***  * *

Human           ATATCACGTCAGGTACCTTGCTAAAAATATTATACTTTTTCAGAGTTTTCTCA-GCATCA 64678
Gallid          TCATTTC-CCTGGAGCTGGAAAATAGATCTCGTGCCAATATTGTATCTGTTTGTGAAGTG 69338
                  **  *  * **  *      * * ** *  * *   *   *  * *  *   * *

Human           CTAACATGCAACGATTCGGCTGAACGCTGCTGCTTAAAAATCTTCTTGATTTCAGTCACA 64738
Gallid          ATTACAGCGCATGATTTGGT--GACCCTTCAATATGAACAGGGGTTAGAATC--ATTGAT 69394
                 * ***    * **** **    ** ** *    * ** *     * ** *    *

Human           ACAGACTGAATAGTCCCATAGTT-CAAAATGGCCTCTCCTAAGTCTTTTTCTATCGTCTC 64797
Gallid          GCAGATACAACGACCACGCAGCCGCTTGTTCGAGACTAAGAAATTGTCAGCTTTCAATGT 69454
                 ****   **    * *  **   *    * *   **   ** *  *   ** **

Human           AATATTTTTTTCAATATTAAAAAAAGCTTTTTG-CTCGGTCAAATGATTACAGAACTTTC 64856
Gallid          AGAAACGGATTATGATCTAATTTATTTTATTTGTCTGGGGTATATTCCGAAATTAATATC 69514
                *  *     **      ***   *   * **** ** **  * **    * *  * * **

Human           CATGTAATCTT-TTCCGAAGTGATTTTCCTTGGTTTGGAGTCAAAGTCAATTCTTCATAA 64915
Gallid          TACGTTGTAAAGCTCGGAAATATTGGTACATAGCTCAATTTTTATCTGAAATGAATCCGG 69574
                 * **  *     ** *** *  *  * * * * *     *  *  * ** *

Human           CACTTCATGCA--AGTCTTGGTTTCGAGATAACTCTCCGGTTTCGCAACC-ATACATGCG 64972
Gallid          CCGACCATCCATCGGTGTATGTAGCGGGCTATCTGGCATTATATGGGGCGGATGAAAGTG 69634
                *    *** **   ** *  **  ** * ** **  *    *  *   *  **  * * *

Human           CCACACATGATAGTTAGCAAGTCTATAATTTTTCCGCAAATCTG----CAACCCTTCTTG 65028
Gallid          ATGAATTGAATATCGACCGCAAAGATATTCGCGCCGCGATTCCGACACCAGCTCCTTTAC 69694
                    *    ***   * *      *** *    **** * ** *    ** * * * *

Human           GGGCAGAAGTAAAGCATATACGGAGTTGATCTTACTGATTAATTGTT---CTATATCAT- 65084
Gallid          CAATAAATATAGATCACAGAAGAGATTGCACAGTCGGAGCAGTTCTTGCGCTAATAGATG 69754
                    * *  ** * ** * * *   ***  *   * **  * ** **   ***    **

Human           -TTAGCGTAGATAATTGCAC--GATAAACTCAACACCGTTATTTAATGAAATGTTTATT- 65140
Gallid          ATGAACATGGATTATTTTTCCTGGGAAAGATAA-ATTGTCCTGTGATGGTACGTACACTA 69813
```

```
                 * * * * *** ***   *  *  ***   ** *  **  * * ***  * **  * *

Human            --GACAAGTGCTCAGCACAACGTTT----CAAACACAACACCTTT---TCAAAATATTTT 65191
Gallid           GAGACAGCCGCCAGTCAAGAAATATTCAGCGAACTTGATAATCTTAAACCAGATGATAAA 69873
                   ****   **    **  *  * *    * ***   * *   **    ** *  **

Human            TTTCTATCCTGGTCTACCATAAAGCTTTC-----CAACGCTGCATCCAAAAGA---AGCA 65243
Gallid           TTGCTATATATAATTACAAATTATCTTCCATCGGTATCGCTGTCCTCACGACGCCTAGCA 69933
                 ** ****       *** *   * *** *      * *****    **  *     ****

Human            CCTGTTCATACAT------AGCTTTTAGCAGCACCTGCATATAAACCGTCAAAGCTGACG 65297
Gallid           CCGGGGGAAACGGCAGACGAGACTTTTTGGCACATGTTGCTTTGTGTTTATTGGGGAAG 69993
                 ** *   * **        **  ***    **** **    *      * *  *  ** *

Human            CACAAAGAAGATTCTGTTCTTTGATACCTT----CAAAAAAAGTTTGATATAAATGCAGA 65353
Gallid           CGAATTGGAACTATTGTTACATATGATCTCACCCCGGAAGAGGCTATAGAGCCGTTCAGA 70053
                 *  *  * *  *  ****   *   * **     *  ** * * *  * *    * ****

Human            ATGACAATCCCA--TTCTTTTTGAAATTTGCCATATCAGA-CAATACTATTACAGGATCA 65410
Gallid           AAGCTTTCTCCAAATTCTAAAGCGACCTTGCTATCACAGGGCAAGGAAACTGAACGGCT- 70112
                 * *      ***  ****      *  **** **  ***  ***    * *  * *

Human            CAAAACTTTAAACATTCAAGGTCCAAA-GCACATTCATTAAGCCTAGCGCACAACACACA 65469
Gallid           CTTAGGTGAGATGGTGTGGTATCCGAGCAAAAATGCAATAACCAAAGCGT-TATTAGGAA 70171
                 *  *  *   *   *      *** *    * ** ** *** *  ****   *  *   *

Human            CAGAGATTG-TAACGAATTCATACTCTGATTATAATCGGTTTTTCTTAGTCATCGGCTCA 65528
Gallid           CGGCGGTTAATAATATGTTACTGCGAGATAGATGGCAAATTATCTCCGAACGAAGACGCA 70231
                 * * * **  ***    **  * *        **      ** *        *   * * **

Human            TCCGTTTCGGTTTCCGTTTCATCACATTTAATACACCGCTTGCTTGATCGTCCTCCTCC 65588
Gallid           TG-GCTGGTATAACTGGACAAAAGTATTTGCAAGCATCATCTTTTACGGCATTGACCGAT 70290
                 *  * *    *  * *    *    ****  *  ** *   *  *    * *   **

Human            GCTAGAATTTCAGAAAA---GTCATAGACCGGAGCATCGTTTGAAGACACCGCCTCAAAT 65645
Gallid           TCAATGACGTCAAATAACGTGTCAGTCACCCACCCAATTTGTGAAAACGCAAACCCGGGT 70350
                  * *  *  *** * **   ****   ***    **   * **** ** *   * *   *

Human            ACCGACTGCAA--ATTAGCTATTGAAAA-ATCTCTCACATTCATATCAACTAAGTGATTC 65702
Gallid           AACATACAAAAGGATGAGGAAATGCAAGTGTGTATCA-GTCCAGCACAAACGAGTGAAAC 70409
                 * *      **  ** **  * ** **   * * ***  * **   ***   *****  *

Human            A------GTTTAAGCATTAT--GGATGCCGCTA-------AAGCTTCCCTAC-------- 65739
Gallid           GTTAAATGCTGGAGTGCTGTCTGGATGCAACGATTTCCATAGACTTCCCCACTCCGACCC 70469
                        * *  **   * *  ******  * *       *  ****** **

Human            --CATCCACAA------AAAAAAGCATATCATCCATAGTCGGCTTGTCCTCCCTATCCCT 65791
Gallid           TGCATCAACGAGCGATCAAACCAATTTGCAATCGCTAATAGAACCGTCCATGA-ACACTC 70528
                   **** ** *      ***  *   *   ***  ** * *    ****     *  *

Human            CATCACCTCAGCTATTATTA-ATAATTCTGGATCAATATCGTTAGTTAAATTT--TCAAC 65848
Gallid           AATCTTCTCGCCCACCCGGAGACGATTTTATTTGGGTCCCGATTAAAAGCTATAATCAGC 70588
                  ***  ***  * *     * *  *** *   *   *  ** *    *  * *  *** *

Human            -AATGCTGAGAA------CCTTGCCCCTTGACCACTTCCG-TATCAAACATA-ATTGTCTC 65899
Gallid           TAGTATCGAGAAATGCTTCTCAGCCAACGAATATTCCCGATATTGCAATTACATCGAATC 70648
                  * *   *****      *    ***    **  * * *** ***   *  ** ** *  **

Human            ACGCCGAACCTTTTTCACTATAACTTCTGAGAACTTTG-TAGCAA-TAATCGTTTTCTGC 65957
Gallid           AGCCTCCGTTTATTCCCCCGGCGTTAATGAATACATCGATATCAGGTCAACACTCCATCC 70708
                 *  *      * ** * *      *  ***   ** * * * ** **  * * *  *   * *

Human            CTCATAAATCTAA--AATCTTGCAATGCAGAAGAGGTAGGGTTTAAGTTCCTATCCACGC 66015
Gallid           CAAGTGGATATGCCCAATATGGGTACCCTACACCCGTAGGTACCCATAACTCTCTGCTTC 70768
                 *   *  ** *    *** * *  *  *   *   *****     *   *         *

Human            CACTTCCACCTATCCAACCTAACTGGCCAAACTGAAAATATTCCCTATTAGCCAAGCTTA 66075
Gallid           CATTGGGACCTGTA-AATCAAATGGGCGGATTTCAATATGGACCTCAGGTGTACCCCTTG 70827
```

```
                ** *   **** *  ** * **  ***  *  * ** **   **  *   *     ***

Human           CAAATTTCTCTAATGTCAAACCAAATGTAACCAA-AGATCGACTTGTAAAAATTGTACCA 66134
Gallid          TCATATGGACAATCGCCTTTAGAAGCAAAACTGACAGCCTTACTTGAATGCATGACAAAG 70887
                  *  *   * *  * *     **    ***  * **    ***** *   **   *

Human           AAATTGGGTTCTTTGGACAGTGACGCAATCACAGGATTCGGAACGCCATCCAAAACTAAC 66194
Gallid          GAAAAGAGACCAGTGGATGAGGA-GCA--CAGAGGCGACGATATGCATACTACTAGGGAA 70944
                 **  * *  *  ****    ** ***  ** ***   **  * **   * *  *   *

Human           TTTTTAACATAAACTAAATATTCCTGAATAGAAGAAAATTTAGGCTGCTTGTCTAACACC 66254
Gallid          GAACGAGGACGACGTGGACGTAAGCGACCATACGA--ATTTGACAGATCTATCGAGTCTG 71002
                     *  *  *  *  *  *    **  * * **  ****        * ** *

Human           ACAGGAAACAACACAGGATTCTGCGTAACCAGCTTAGATGTAAGACCATGTAAGCTAGTC 66314
Gallid          ATCTTTATTATCCCGGTGAATTCCGT---CGGTCTAATTTTTCTCCTCCTCAAGCCAGT- 71058
                *     *  * * * *     * ***   * *  **  * *    *     **** ***

Human           AAGTAGTCATGAAAACCTAAAGCCGACAAAAACTTGTTATGAAAGTAACAATCTATAAAA 66374
Gallid          ----AGTATGAAATATGAAGAAACTACTGGGGGTCGGCATGATCTGAGTCAAACAGGACC 71114
                    ***    ** *   * *  * **      * *  ****    *    *   *  *

Human           GTTGAGAGACACTCTGGCTGTATGTCGATAATGTCTATCTCATCATAGGTATTCGTAATA 66434
Gallid          CGTATTAAATAGTCTA-----ATGGGAGCTGTGACTTCCCTACAAAAAGAAGTCGAACGG 71169
                  *   * * * ***      ***       ** **  *  *  * * * * *** *

Human           CTGAATGCA---AATTT---GATA---AAAGTCTTAACTTCTTCGGGGTTACCAATATCT 66485
Gallid          CTAAATGGAGGAAATTTACCGATATCAAATGCACAAAGTTCATATGGAGTGCCCA-ATGG 71228
                ** **** *   *****   ****   ** *     ** *** *  **  * ** * **

Human           AAAGTTTTCGGAACATTGTTGAGCAGAAAACGTTGCCATAACTCTAAGCACGAAATTTTG 66545
Gallid          GATGCATGCCCCAGTTTATTACTCATACCCTCCTCCGGGAACACATCCCACAGTTTCATG 71288
                 * *  * *   *  ** **   ** *      * *   *** *    ***    *  **

Human           ACATTCGGAAACAATTTATCATGATATTTAAATAAAAGAAAGGAGATACAGCCAGACAAG 66605
Gallid          GCCCATGGGAGTCGAACGCCCTATGCCTTCCACGGAAGGAAAAACTTCTA-CCAATTCCA 71347
                 *    ** *          * *     **  *   *** **  *  *  * ***

Human           GGATTCTTCTTGTG----TTTCACGTTC------TTTTTAAAATAAG--GGTTGTTGTGT 66653
Gallid          CGGTCATTCCTGTGCCAGTTTCAGATCCGGAGGCTGGTCGAAATGTACCAATAACTGCG- 71406
                 * *  *** ****    *****  * *      *  *  ****       *   ** *

Human           GCTGTCTTACTGGACTTGTTGAACGGTCTATTTTTTATTCTAAAATCTCGAAGACATTGC 66713
Gallid          ACCATCTCTCAGGAGCGTTCCGACGGA--ATTCAGAAGGAAAGCATCGAGCAATCACGGG 71464
                 *  ***  * ***    *   ****   ***    *    *  ***  * *  **  *

Human           ATAG-ACAAACGGCAAAGTTTCGCCTGAATCGTAGTTTTCACATATTTACCCGATTTATA 66772
Gallid          ATACCATGAATGCTAGCGCCGTAGCTGGTATACACCGCACCAGTGATGCCGGCGTCGATG 71524
                ***  *  ** *  *  *      ***      *     *   *  *  *    *  **

Human           TAGATCGCAATCTAAGATCTGCTCCATATCAACATTCGAAGCGACTTTAACCGT-TCTCG 66831
Gallid          TATTTATTAATCAAATGATGGCGCATCAATAATACAGGGAGCTATGTTTTTAGCATCACG 71584
                **  *   **** **     ** *   *  ** *   * *** *  **    *  ** **

Human           TATTCAGAAAAACCCTTTTTGAGATAACTGCTGTGAAACGTTCCGTACAGTGATTGATAC- 66890
Gallid          TTCGCAATATATCGTATTGAATATGCTTTATATCTCGCAATAAATG-AGTAAACCATATC 71643
                *   **  * * * * **  * **   *  * *    *  *   *  *** *   ***

Human           -TGCTGAACGAGCCATTTAGAAATTGAAT-TACCAGTACAAGGTCTGTCGACAACATGAC 66948
Gallid          ATGCTCTATGCGTTTTATTCAGTTCAAATATAATAGTGCCCACTTACACAGCATCATCTT 71703
                 ****  * * *    * *  *  *  *** **  *** *    *    *  ** ***

Human           CTGAAGCCACTATCAATGCGAGGTTCTGCAAAACCGTTAAAATGACCTTATAATATGCAA 67008
Gallid          CTGA-GTCTGAATATGTAGTAGGTAACTTATCATATTTAGGGTTACTATTTTTAATCCTC 71762
                **** * *   **   *   ****     *  *   ***   * **  * *   ** *

Human           ACGTCAAAAAAAGGAGAA-AATGCAGTTGCA-TAAGGGGTCGTATCTACATT---AAATGA 67063
Gallid          ATTTTTGCCAGGTGGTCCGATAGAACGGCGGTAGTGCCTCGCCTCTTTCTCCGCAGTTTT 71822
```

```
                *  *     * *  *    **  *   **  **  *  ***  ***   *    *  *

Human           TTGTAGACAATTTTCTATCTGTTCTCTAGGCGTTTGTGTCCTTCTCATTTCAACAATGCA 67123
Gallid          TTCTCGTGGCGTTCTTCCGCGGAGACTAACGCCATATATT-TTATCATTTCTCTAGCTTC 71881
                ** * *     **  *    *    ***      * * *  ** *******   *

Human           TCTAGAAACACATTCCTCGATACAT---TGTGTCAATCCGT--GGATTATAGAAACAAAA 67178
Gallid          TTCTAATTTTCTTTCATCAATAGATGTTCGTTCCAAATCATCTGATTCCTCGCCATGCAA 71941
                *    *    * *** ** *** **    **  ***  * *  *  *  *  * *  *   **

Human           TCTTTTTTACTTCGGACATTGAAAGTATCTTCGCCTGTTACAGGATCAA------TCAGA 67232
Gallid          -CTCACGCGTTGCCTGTGCCTTAAGCA-CTTCTGTTGTCATAGGATAAAGGGCTTTCATT 71999
                 **       * *         *** * ****   *** * ***** **      ***

Human           GAATTTTTTTTACAGTAATCGAAGATCTGAGATAC---AAATTTACCCCTG--TCCAGTG 67287
Gallid          GGATTGCTTTTAAGCTTGTTTACATAACGATATGCTAAAAATGCAGCCACGAGTCCTGCT 72059
                * ***  *****   *  *  *      ** ** *   ****  * **  *  *** *

Human           AGAGCGTGTTTTGTTGATCTTTTCCAATCTCTTTGAGCTCGC--TGACAGGCTTCCTGCC 67345
Gallid          ATAATG-ATTAAACCGATTGCCAAAGCCCCAAAGGGATTTGACATGAAAGCAGAGACACC 72118
                * *  *  **     ***          *     *   * *   *** **        **

Human           AAAAGAACCTAATATATCAACCTCGGCATAATACCGAGAAAACATGCTCATAACAAGTGG 67405
Gallid          AGATATGGTAGATACGATTGCACCGGCAGCCCCTACTACAACTTTGCCTATAGCTTGCCC 72178
                * *        ***      *  *****           **   ***  *** *  *

Human           TTCCTTTTTAACATTTTTTGGTATTGCCGGCAAACGAGT-ACCAACCCGCACCATGG--- 67461
Gallid          TACCTGACCCATACCGTTAAACAATTCGGCCAAACCGTTCATAAACGCGTAATTTGTATC 72238
                * ***     * *   **    * * * * *****   * *  *** ** *   **

Human           CAGTTCCAATACACGATTGACAACATTTCCCATCACAGAATTCACATAAATTCGCCGAAC 67521
Gallid          CACTTCTATTACTTTGTTTATGTCATAAAATTTAAGTTCATGTAGTTGATTGCGGCGAGC 72298
                ** *** * ***    ** *   ***      * *    **  *  * * * ** *** *

Human           AATTTACCAGATGATTGTAAATTTCAGTGT-ACGCATTACCCGCGTTATAAACGCTCATA 67580
Gallid          TACTT-CTGCATAATCCAATACACCAACATCACGCAACTCTTCTTTTGTGTAAACGGATA 72357
                 * ** *   ** **   * *   **   * *****   *     ** *  *  *  ***

Human           CGATTTAAATTCCACACTACATAAGAAAGCAGCTGTGGGCAAGTCG-CACAGGCATAAGC 67639
Gallid          AAGGCAAAATTTCCCGATCTTCTAGCAGG--GTTAGATTAAGCTCGACAAATGTGCTAGC 72415
                      ***** * *  *     ** * *  * *      *  *** ** * *    ***

Human           CAAGTGATTAGCTGAAA-ATTTGTCATCTTTGCCTGATG-------TCGAATCACATTTG 67691
Gallid          AATCTGTATATCGGCAGCGTCTACCATCTTAACAAAATTATAGTTTTCAAATAAAGCATA 72475
                 *  **  ** * * *   * *  ******  *   **        ** *** *    *

Human           ATGATTTTTCTATTATCGTTATACAC-ATCCTCAGTCAAAGACGACAACGCATTACAAAA 67750
Gallid          ACCGGATCCAAACAGAAAATATCTACGATGATTAGCCGAGCATGGCTCTACAGCCTCTAG 72535
                *     *    *       ***  ** **  * ** * *  * * *    **      *

Human           AGACAAAGATCGCA-GCAGTAATTCTTGAGTCATCACCGAATTAAACGCTTGTTTCA--C 67807
Gallid          CGTTGGAAGCAACTCGTTGTTTTCACCGAGTTGTCCCTGTATGTTTCCTTGGTTTTCTCC 72595
                 *    *     *  *  **  *    ****  ** * * **    *  * ****    *

Human           GTTTGGTATAAATGCCAATTTTGAGCAATATAAGATAGAATTTGGCGCAAAGATCAAAAC 67867
Gallid          ATATGAAAATAGAACCAATGGTCGGCTATAACATGTATTAGTGGATGTGATAACTCGCAT 72655
                 * **  *  *   *****  *  ** ***  *  **  * * *  *  *  *     *

Human           GGCAAGATGACTAG--ACAGATGTAACTTTAAATTCGTGAGCTGCGCCATGCGTTCAGCG 67925
Gallid          AGAATTTTGCAAAGTGACGGATTCCGCATCTATAGCA-GTGCAGCTCGATACAG-CAGCG 72713
                 * *   **   **  ** ***    * *  *   *  * ** ** * ** *   *****

Human           TGTGTCTCTGAGTTTCTTATGATTGGCCAGTCATTGAAATTCATTATCTGACTGGGGTCA 67985
Gallid          -ACATCCCCCAACATCTTTGCAGCCACTCTCCTTCCTAATGTTGCA-CTCGCTGTAGCGC 72771
                    ** *  *   ****   *    *    * *   ***     * **  ***  *

Human           TACACCGAATCCAGGTATTCGGCTACATGACTAAACGATAATTCTGTCACAACGGAATCA 68045
Gallid          TAGGATTAATCT--TTATCCCTTCGTGCCATAAAACAAGTTCTCTATT-CTGCAATTCGC 72828
```

```
                 **     ****    *** *          *  **** *    *** *  *  *

Human            ACCAACATCAAAAAATCTTTCTCGAC-CGCTGAAATTTTCTGTCCGGAGATACCCAGATA 68104
Gallid           ACCAAGCTGTGGCAATCCTACTAAACATATCATTAATATGGGTTTGTATATGATCATAAA 72888
                 *****  *     **** * **  **         * * *  **  * * **   ** * *

Human            AGTTTTGTGGGGTGCTAATTTGACAGTTTGCTCCCCATCCTGTGTAAATTGTTTCAGGCC 68164
Gallid           GAAATTGGAGCATGGCGAATTGAACAGACGATGTCGATTTAATAGCTGTGGTGTC-GTCT 72947
                     ***  *  **   * ****      * *  * **    *     * ** ** * *

Human            AGCATCGATTAATTCCTTAGTA---TTAGAAAATCGTAACGTTGTACC--CCATGATGTA 68219
Gallid           AATGTTATTTTTCTATTTGGTGCATTTCGAATATCTCGCCGCAATCGTGACAATGAGGTA 73007
                 *   *   **   *  ** **    ** *** ***    **   *     * **** ***

Human            AAAACATAATAAAAAAGCGTTTCACTTAACG-----CAGGCAGAAAAAATCCTCGTTCCT 68274
Gallid           GCATTTTTCTTATAAATTGCAT-GCTTATTGTTTACCAGGTCGAGCATCTCATCGGTCCT 73066
                   *   *  * * ***  *  *  ****  *     ****  **  *  ** *** ****

Human            GA--------ATAAATTCT-----GTACAGG-AAGATAAAGAAATCGTTTCTTCAGTTTC 68320
Gallid           GTTGTCTCTCATCAATTCTCTGAGGTACATATGAGCCAGGGATTTGGATAGAACAGGCTG 73126
                 *         ** ******     *****    **  *  **  * * *    ***  *

Human            AAATAAAGTTGCAGAGTATAGAGGAATTTTCACACACGAATACTCCCCAACTTGTACTTG 68380
Gallid           ATATGCTACAATAAATCCCCCGAGAGCCAAGAAATATTGTACATGTCCAACCTTGACGTG 73186
                 * **        * *         **       * * *        *  ***** *  ** **

Human            AAT----TTGTTCTTCAACACAAGGAATTAACAGACCAGCGAACAATAACTC---TTTGA 68433
Gallid           ACTGTCATTATATTTTGTCCTAAATATCTGCTCGATT-GCTGCTTCTGCCTCGCGTTTAA 73245
                 * *    ** *  **   *  **  *  *    **   **       *  ***   *** *

Human            AACCGTTTCCACACGCAACATGGCAAATAACAGAATCTGCATCTTTGCCGACAGCAGAAC 68493
Gallid           TACATTGTCCTAATATGATGCGATTTGGATCAAACTCAGTCGTATTACTGATAAACGTTG 73305
                  **  * ***  *     *   *       * ** * ** *     ** * ** *   *

Human            AAAGAGCTTTGATATCGATTACTTTGCGTGCTGTAGGCACCTCAAACGACTGCAAGTGAA 68553
Gallid           CCGAAAGTTCACGGGCCATAAAT-------CTGTATCTCCCATTAACTGTTGCACGCAAC 73358
                     *  **      * ** * *       *****    **   ***   **** *  *

Human            ATTTCTGTCTGGTTTGCTCACATAATCGAATAAGAT-CACCA-TGATCTTCGG----CCA 68607
Gallid           ATTTCAGTCACCTCTTTCCACTTAGTCATTGAACATACACGAGTAGTTTTTGGAGCCCAG 73418
                 ***** ***   * *   *** ** **    ** ** *** * *  * ** **    *

Human            TACCAACTAACTGCTCAGTTCCATGGAAAAAAAAA--CACACGGGCATAAAAGACGTAAT 68665
Gallid           TCCCACCCAACTGTGAAGTGTGATGTGATGAGAAAGTTACGCTTGACTGGAAGGCTTGCT 73478
                 * *** * *****   ***   ***  *  * ***   ** *  *  *  *** * *  *

Human            TTTAG----TTAGAATTGTGCCTCCAAAGTTAGTGAT--AGGTGTTTTAA-CCGTGGTCG 68718
Gallid           TTTCGACGCTTGTCCAAATCCATTGAAAAATAGCTATCTAGTTGTTTGAAATTATCCTGG 73538
                 *** *    **        * * *  ***  ***  **  ** ***** **    *  * *

Human            AGAAATC----GTTTTCTACAGTCAAATTCATTAACAACGGCGAAATAACTACAGGAGAA 68774
Gallid           GGATATCCCATGGGTTCTGCGGCAGCCTCTGGTGGGGATAGACCATAAAATGGAGATATG 73598
                  ** ***    *  **** * *      *    *    *  *   *  ** *  **

Human            TT-----TCTCTCCATTAACGAT-----AAAACAGATAAAACATCCAAAAGT-----TCT 68819
Gallid           TTCGCGATGTCGCCATTGGCCATTGCAAAATATGAATACGGAAACACAGAGCGGGCATCC 73658
                 **      * ** *****  * **     ** *   ***    * *  * **       **

Human            TTCTCTTTTGGAAAAACATAGATCCAAGCAGCAGTA-GAAACAGGAGCAGAAACCACCGT 68878
Gallid           ATTTCCTCTACTATACAATTGACGGAGGTTCCCGTTCGATATATCCATGGTGATCCCCAC 73718
                  * ** * *   * *  ** **   * *   * **  ** * *      *  * * **

Human            TTCGTTTTCATCAGCCATCTTTGTGGATTCGATCACAAAAACAAAGATAATGGGTTTTTG 68938
Gallid           ACGGTATACGTCT-CATTAGTCGTGTGCCATGCCCTAGATTCGGGCGTGTTGAATTTTGA 73777
                    ** * * **  *  *  * ***         *  * *  *      *  **  ****

Human            TGGTGAAATCCTTATATATTATGTTTGACGTAACA-TAACACGCGTCATCAAACATAAAA 68997
Gallid           TGGTTTTAGAAGTACTTGTTTT-TCTCCCGCATCCCTGTCAAACGC--TTCAACATATAC 73834
```

```
                ****    *     **  * ** * * *   ** * *  *  **  **    *  ****** *

Human           GTAAACCACAAGTTGAAATATACC-GTTTTCTATATGAGTTTTACGGACAAAAGAAAAAC 69056
Gallid          ATTGTTTCTAAGGTATCTTGCTTTAGATGAGCATCTTCCTTTGCCGTCGATTAGATCCGT 73894
                 *       *** *    *      * *    ** *   ***  **    *  ***

Human           GATTTTCTTA-TGCAAATATTTTCCACGCAGATGACATGACACGCCCCTTAATTTAAATT 69115
Gallid          GATCTCTTCAATGGAAACGGGCGTCCTATCTGT-ATAT----CGATTAGTGATCTGTCTA 73949
                *** *  * * ** ***        *        * * **     **      * ** *   *

Human           TATGTAAATCGTCGTCCACCTCA--GGTACAATAGTATATTATATATATAGTTTTTTAAT 69173
Gallid          TATGTCG-TCCCCGTCCATGTCGTCGTCTGAATGATATTTTTATAATAAAGCGTCACTTT 74008
                *****   **  ******  **    *    ***  *** **    *** **  *      *

Human           AAACTTATTGAGGACGGGAGAACGAAGGCGTGGCGTTTACGTCATAGCCTAATTATACAT 69233
Gallid          AAATTTAT-ATGGACTGA----------TATTCTCTTTAAATAATATCGCGATTCCTTCA 74057
                *** ****   **** *             *    ****  * *** *    ***

Human           TCTCAGAACAGGATTTAAAAAGGCTGCGAGCGGC----CGGCTGTTC-AGAGGGACGCTG 69288
Gallid          CCCCATTCGGTGGCTTTTCTAGGTTCGGGACATTTTCGCGGCGGTTCTAGACGGAT--CA 74115
                 * **      *  **    *** *  *  *        **** **** *** ***

Human           GGGTACGACTTGAGACGTTTGACTGAAAATGATCCTTCGTGTACTATTTTCTACAAAAAA 69348
Gallid          CGGTTGAACCC---ACTGGTGGGGGACAAAGATAAAACGTAGACT--CTTCCTCAGACAA 74170
                 ***   **     **   **   ** ** ***     ***  ***    ***  ** * **

Human           ATTAATTTGCCGGCGACAGTAAACTTTTCAGCGGAATTTCAAAAATTAATTCCACATGTA 69408
Gallid          CTGGA--CGCTCGAAACAACTTCTCTTGATGTCACATTTTGGGTACT-----CGGAGATG 74223
                 *  *   **  *  ***        **    *     ****      * *      *  *  *

Human           ATTTAAGCATTTTAAAACGTATAACTCACAAGGTGAAAACATATTTACGAATACAGTAGT 69468
Gallid          AGTT---CGTACCATATAGAATAACTATAAGGAAAAAAATGCAATTCCGCCTAAAATAGT 74280
                * **    * *    * *  * ******    * *   ****    * ** **   ** * ****

Human           TT-TCGTGATATTTTTTAAAAATTAATAAATTTTAAATCGTGTAAGTAATAAAGCA---- 69523
Gallid          GCATAGTGAGATGATCTTAATGATGCTGT-TCATAAATTGTGTGAGATAAAATGCAGGGA 74339
                   * **** **   * * **    *  *    *  ***** **** **   * ** ***

Human           TACTATTAGATTCTTCACGTTAACAGAGCAAGCTCTTCGAGGTTTTCAGTAGAATTTCAA 69583
Gallid          CACAAGAATATTGGACGGGTCAGATGGGGGCGTGGCTGTTGGTAATCTGTTCTACTC--G 74397
                 ** *  * ***    *  ** *    * *    *     *    ***  ** **    * *

Human           TATTTAATTTCACATGTAATTTAAGC---ATTTTAAAATGTAGAATTCACAAAGTGACAA 69640
Gallid          CACCTGATCTGCC--GTAACGGGGGCCATATACTGCAAAATAGAATA-AAGCAACGAAAA 74454
                 *  * ** *  *  ****      **    **   *   **   ******  *    *  ** **

Human           AACATTCACAAATACAGTAGTTTTCACG----GTATTTTTGAAATTAATAAATTTTAAAT 69696
Gallid          AACATCTGTATCAATAATTGCAGTCATATTTTGCGTTTCTGAAGTGCTTGATTCTGACCC 74514
                *****    *    * * * *    ***       *  *** **** *    * * * * * *

Human           CGGGTAAATGGTAAA---GCATGCTGTTAAATTCACGTTA-ACAGAGCAAGTTCTTCGA- 69751
Gallid          CAGAATCATTATAAGTGGATATTCGGTTTCAT--ATGTTATATATATTCCATTTTTCCAT 74572
                * *     **  ***       ** * ***  **  * **** * * *      ** *** *

Human           ----GGTTTGCTATG-TGTTTATCAGTGAA-TGTATTTAGTAT-----TTCAATTT--TA 69798
Gallid          ACATAGTCTCCATTGCTGTTGATCTGCAAACTGTCGGCACGACAGATGTGCAATTCGCCA 74632
                     ** * *  ** **** *** *  ** ***     *  *       * *****     *

Human           GATCATGGTTCGGTAAAGATATAAGTCCGTGTAAAATTTTTTGGTTTAGTTTTCATATCT 69858
Gallid          CATTTAAATACAGAAGAAAATAAAGCTGTTGCAAAAATCAGTTCTTTGATATATTTCCAA 74692
                 **      * * * * * *    ***     ** **** *    *  ***  * *    *

Human           ACTAGACTGTGAGGTGTT-TGAAGTCGATGGTTTAGCTCATGTCAAAACGCAATGTCAAA 69917
Gallid          GCCATGCGATGGGCCGCAGTTACATTATCTATAGTGCTAAAGTCATAAAACCCTCTAAAC 74752
                 * *  *  ** *   *    * *   *       *    *** * **** **   *   * * **

Human           TTG-TAATAAGTCGTTCATTTCGTGTTTAGAAATAAGGAATTGGTATTTAGAATAGCTTT 69976
Gallid          TTACTGACGGTCCAATCTGTT--TTTCTACTGCTATGAAACATGAATCGAGCCATTTCCT 74810
```

```
                **  * *     *  **  **  * * **    ** * **   * **  **        *

Human           GTCGCATTTTTAGCCGCGACGAGAAGGTTATGACGACGTTTTCGATTGCATTGTTTGAAC 70036
Gallid          CCTTTAAGTGAGGAAGCAGCCCTACATTTTCAACACTAAAATATAATGCTGTATTCGA-C 74869
                     *  *   *  **  *   *   **   **       *  * ***  * ** ** *

Human           CTTTTTCGGAGTTTGAATTTACCGT--TTAAAGAAAAACAGTTTCACGAAT----AGAAT 70090
Gallid          GGCTGGGCGAACCGATGTCTATCGTGACTAAACATCGGTCCATTCACAAGTTGAAAAAAT 74929
                   *    **       * ** ***   **** *        ***** * *    * ***

Human           CTA-AAACAGGCTTCCAAATTTTAGTTAATATTTATTTCAAATTTCACTTGGGGTA---- 70145
Gallid          TTGTGAGTGAGACTTGGAAGCATAGC-AGGATCTATTCTGTGTTTCAGTAATGATGTTTG 74988
                 *   *    *  *   **   ***  *  ** ****     ***** *   * *

Human           CAGAAAGCTTTG-GCATCGAGATTTTTTTGATTTTAAAAAGAAATAGTTCAGAGTGTGAC 70204
Gallid          CATGAACCGATGAGCATCAAATGCATCGTCACTATTACGA----TTATCAATGATGTTTG 75044
                **  ** *  ** ***** *     *  * * * * *  *    *  *  *   ***

Human           AGCAAAAACCTTTGTACAAAACATACTTGTAAGAATTTGTTTACAGTAAGATAGTTATTC 70264
Gallid          CACAGCAATTTTT------AAC-CGCTTCCAATAATCTTTTCCTGGAACGGAACCCATT- 75096
                  **  **  ***      ***   ***  ** *** * **    * * *  *   ***

Human           TGAGTCA-GAAGAATATGTGTTTAAGTTAGTTTCACAGGTGTCAGCTTCATAGTCTAAAT 70323
Gallid          -GAATAATGCCGTATAAGAGTCTA--TCAATACTTCGCCATATACATTCACACGCAACAT 75153
                 ** * * *  * *** * ** **  * * *    *       *  **** *  * * **

Human           CTAAGTCCATTTCAACACTTGATTCACTTTCATGTTTAGAGTTGACGTTTTCATTAATGT 70383
Gallid          TTTT-TCTAGTTCATGTCTTTGTTCGGTAATACATTTA---TCCAGACTCGCTAGGGACT 75209
                 *   ** * ****   ***  ***  *   *  ****   *  *   *  *       *

Human           TAATAATCACAATTTCGTCTTGTTTTGCTTGATTGAAGTTTAGTTCTCTAGGATCTGGTA 70443
Gallid          TCTTTGTTAAACGCTCTGCATACATTTTACGTCTACGAGCTACATCACTTGATGCCG--A 75267
                *  *  * * *   **  * *   **    *  *      **  ** ** *   * *  *

Human           TTTTGTTGTCGGTTTCCATTGATTCTTCTATGAGAATTTCGGTAGT-TGTTTTTGTCGTT 70502
Gallid          TTTTACCATCTCCAACCATTCATCATGGCCAAGGGAGGTCGTCGACATAGCAAAACTGTC 75327
                ****    **    ***** **   *       * * ***       *         **

Human           GTTAGGTTTGAGGTGTCTGGTTTT-TCGACGCAAACTTTGGATTTAACCTTAGGCGTACC 70561
Gallid          ATTAGTACGAAAATGCCCCGTCTCGTTCATACGCTCTCCACATGTG-CCGTCGCCAT-CT 75385
                 ****     *  ** *  ** *  *  *  *   **    ** *  ** * * * * *

Human           TTGGTTTTTATGTGAACGATGAGA-GGTCTGAGGTAGGTCGCGACTTGTTGAATAGCTGC 70620
Gallid          TGGCATTGAATGTCATTTCCTATACAATGTGAAGAAACCCGC----TCCAAAACAGTTT- 75440
                * *  **  **** *      * *   * *** * *   ***    *    ** ** *

Human           ATGAAGGTTCCATGTCGACCTTTGGACAAGTAAAAGAAAGATTTCGGTATTCGTGAGGAA 70680
Gallid          -TGAGAGCATCATGCGCCGCTTCATCGGGATATAATAGGCGTTTTAGTAATGGTGCCGAC 75499
                 ***  *   ****     ***        ** ** *    ***  *** * ***  **

Human           GATTTGGAAACAGCTTAAACTTTGGTGCGT-CTTTGGCGAAATAAGTGCAGAAAAAATAGA 70739
Gallid          ATATGATGATCATAACATGCCTTTATTAATGCTTCAATGATATCATCG-GGTGCCGCTGC 75558
                   *    * **     *  * **  *   * ***    ** ** *  *  *       *

Human           CTTGGTTTCCAAGG---AGCCAT--GATTCGTTCA---ATTCTATTTTGACAGCTGACA- 70790
Gallid          CCTACCCCCTAAAAGTAATCTATCGGCTGCATTCATATGTTCTATTTCCTGGGCAAATAC 75618
                * *     * **     * * **  * * * ****    ********     **  * *

Human           ----TCATCGC-ATTTATG-GTTGCTGAATTGCT----TTCCGATGAGCCTGCTTATCAG 70840
Gallid          ACGTTCAAAGTGATCCATACGTCGATCAAACACGGCTGTTTCTACGATTGAGGTTCTCAG 75678
                    ***  *  **  **  ** * * **   *     ** * * **    * ** ****

Human           ACCGCATTCGTATCGCTCGGCTACACGTTCTGTGTCGAAATC-GCGGATGAAT-TGCGGT 70898
Gallid          ATCGAAAAGACGTTCTTTGGATACCAATGCCTCGAGATTATCAGCAAATGATTCTACTGT 75738
                * ** *      *   * ** ***   * *   *     *** **  **** * * * **

Human           TTCTTTGTTCGTATGATATGTTCTTTAATTTGTCGTAGTA-TTCGAGCGAACGTCACAAC 70957
Gallid          ATTTCCGCGTGGTTGGTGTAGTTTCCGTCCGGATGAAGCAATCCAAAACTGTAATTCGCT 75798
```

```
                 * *  *   *  ** * *  * *       *  * ** * * * *          *

Human           GATGG-ACATGGCTTGTTGAA--TTGGGTGT------CTAAATGAGTCTGGAGT--TTGT 71006
Gallid          GATAGCATATAAATCACCCGAGGCTGGAAGAAACACGTTATGAGCGTCCAAAGCAGCAGC 75858
                *** * * **   *      *   ***  *        **   * ***   **     *

Human           TGTTGTACTTCAAATTCGGAGTTTGGCATCTCTCGATCTAGTACAGGTTT--ACCGCAGA 71064
Gallid          TGCGGCTTTCGAAACAGAAATGTTACTATCATTCTCGGTAGTAGTGTCTCTCATTGTAGA 75918
                **  *   *  ***     *  **   ***  **    *****  *  *   *  * ***

Human           GTTGCCTGAGTTTTCGGTATTCGATGTCGCCGTGTCCGTTGTCGTTGAAAACACAGGTTA 71124
Gallid          CTGGATTACATCTAGTGCAGTTTGGGCGGTCGTGCGC-TTGTCATCGGGCACATACGTAG 75977
                 * *  *   * *   * * *    *  * ****  * ***** * *   *** * **

Human           ATTTCGCCATGTTGAGTTTGTTGAAAAATTCGAATTTCATGACGGTGATGGTATACATGA 71184
Gallid          CGTGTGGTAAAT--GTGTTACCATATCATCCGAAT----TGACACGAATG-TTTATTTGT 76030
                  *  *  *  *     **     *  ** *****    ****    *** * **  **

Human           ATAAGTCTTGTGGGTTGACCGGTAGGTTGTGCAGATCG-TGAAATTTAAAATAACTGCAG 71243
Gallid          TTGG---TTATATGATCACAAATTTTTCTGACGATACGCTTATGTGTTGAATCTCCACTA 76087
                 *     ** *  * * **   *   *    *    ** * *  * *  ***  *  *

Human           GCTTCTTCTACTGTAGCAACTGTAAAAGCAAGACAAGGTGAGTTGGTCAGGGAGAATA-- 71301
Gallid          TTTGCTGTTACACAAAGTTCTTCAAAACAAACAGAACATGGTTGGGAAGGGTCATATAAC 76147
                  * **  ***   *    **  ****  ** * **  **  * **   **    ***

Human           TCCGGTCGCCCATTAGTTGCTGATTTTCT--TCTCGCGTGAGCGA--GTCGAGACATTGT 71357
Gallid          TCTGGTGGGACGACTGACCCCGAACCTAAAGTTTTAAGTAGATAATTGTCTAGACTCACC 76207
                ** *** *  *    *   * **   *    * *   **     *  *** ****

Human           CGATAGCGAAGCAGCAGGTCGGTTTCCAGTATGTCATTAGTTAGGAGACAAATTCGTTCA 71417
Gallid          ATGAAATTTTCCACGGTATCGGCTTTTAAAATTAGAT--GGCAGTAATTGAGCTGCTTCA 76265
                    *      **     **** **  *  **   **  *  ** *    *  *  ****

Human           -TGCGTCTGTGGAAATCTGTCTCGGATGCGTTGTTTTGGATCACGGGCGTCTCCGTTGTA 71476
Gallid          ATACATTTTCCACATCATGT---AGAAACTTTATTTCGG--TGGATACCTTCCCACCATA 76320
                 * * * *     *   ***    **  * ** *** **        * *  **    **

Human           AGGTCTCAGGCCAA--GAGATTTTATCGAAAAGTTTGTGTCG-GCAAAGGGGACTGTTGG 71533
Gallid          AGTATCCAACTCGACTGTGCTGTGATAGGGACAGTCGCCACACATAGACATAGATTTTCT 76380
                **    **   * *  * * * * ** *  *   * *   *    * *      * **

Human           AAGGTTAGGCAGAAAGGTGATTGCATTGTTTTGAGCGCGTTTTTCTT------GTTGTTT 71587
Gallid          AAAAAAGTCTCGCAGATTTCCTCCATCCTTAAACTCGCACAATGCATCCAATAACAATTC 76440
                **         * *   *   * ***  **     ***    * * *          **

Human           CTCTTCGATGATGGTCTTCAGCTGTTCTAACTCT--GTCAGAC-TTAGTGCGTCTAGTCT 71644
Gallid          CCCTTCTAATTTGGCCCGCCGCAGAGCCAACCATAAGCCATAGGTTAAGGGGGTAAGATT 76500
                * **** *   *** *  * ** *  * ***  *  * ** *  ***  * *   **  *

Human           AGATTCATCCAGTTGTTTCAG---AGGTGCGAGTTTTCCTCGAAAATTTTTGGCGAAATG 71701
Gallid          CATCTCACGTTGTTCCTTTAATGCAGGCACCATCTTCTTTTCCAGGTAGTATATCATAAG 76560
                    ***    ***  ** *    ***  * *  **   *   *  *  *     * * *

Human           GCTGATGAACTT--GCTGGTTGGTC-----TATGCGTGATGGAACGTTTTATCGTTCTGA 71754
Gallid          AACATTCAACTTCAACCGATTGATCATCCGCACGCCTACCGTCGGATCGCATCGTTTCAA 76620
                     * *****   * * *** **      * ** *   *     *   ******   *

Human           AGGCGGTTTGTTTATGACGATCATGTTGTGAGCGTTTTACTCCACGAGGATACATGTTTG 71814
Gallid          GAGT--TCTACCTGAAACAAGTATGATT---GTATCTGGCCCCAAATAGCGAATAGTTTC 76675
                  *   * *   *   ** *  *** *    *  * *  * ***    *  *   ****

Human           CTTATAATAAATGAA--ATGACATAAATTGGCCTCTTTATATGTTTTTTGAGATGCTTTC 71872
Gallid          TGAGTGGCAAATTTATCATGCTCTGTACCCTGCGACTGCAACCGGTTATGAGACATTCCC 76735
                    *   **** *  ***   *  *     *   *   *    ** *****   *  *

Human           CGTTGGCGC---TTTTTTGCGGTTAAGTTGTGTGAAAGAACGAAGGACGGTGTTGCTAAT 71929
Gallid          AACATGCTTCAATCCCGCACAACCGACGTGTGAGAAAATATAAAGATTTGTGTGGAATAT 76795
```

```
                 **     *      *     *  **** ****  *  ***    **** *   **

Human       GCTTTAATCTCCGGAAATGTTGTGATATGGCGCGATTTAGTTAGCATGTGCGTTGGAGTC 71989
Gallid      ATCAGAGTTGTCA-AAATGTTAT--TACGCCATTTCCGGAATAAAGAAAGATTACAAGTC 76852
                 * *   *  ******* *  ** * *          **      *  *   ****

Human       AAATGGTAAATACTAAAAGAAGGTCTGGGTTTATAGGTAGTAAGTTAGTTTTTTAATTGC 72049
Gallid      GCCACAAAAATATAGCTATAGCTTTATTGTACATGAATCG-AAGACAATGGTATACATGC 76911
                   *****     * *   *    **  **   * * ***  * *  * **  ***

Human       TCTA---AGGCGATGGAACAATGTA-CTTGTTTGCTGTTGG-ACTGGCATTTAGTAGCAA 72104
Gallid      CCGATGTAACCTATCCAGAATTACAACATATCTACAGATAACACTGGTACGCGCTTCTCG 76971
             * *   *  * **  *  * *  * * * * * * * *   ***** *     *

Human       AGCATGTGCTCCACAAGATGAGAATTAACCGATTTCCGTCGGCTTTGAGTTGTATGTAAA 72164
Gallid      GGAATGGGCTCCA---GGTCAAACACATTCATATTCGGACGTTTTAAGTTGGTTTCTGAT 77028
             * *** ******   * * * *   *  *   *** * **  **    * ** * * *

Human       CTAGGACGTT-ATCTCGATTTCCT-CTATGTTGTCTGTTCAGGCAGCTGAAATCCGAAAC 72222
Gallid      ATGGTCTGCCCATCTGCATGTCCTTCCAAATTCTCGTCACATGCATC--AAAATCGAAAG 77086
             * *   *   ****  ** **** * *  ** **    ** *** *  ***  *****

Human       TCTTCCAAATTGTGTTTTTTTGATCTGAAGGTTCTTTGAATCTATTTTGAGAAAA----- 72277
Gallid      TAGGTCCATCCGTGGGTATG-GACATGGCCATATTAACCATCTCCTTTGCTAAGGTCATC 77145
            *    * *   ***  * *  **  **    *  *    ****  ****  **

Human       GTTACTTGTGAAAACTGTTGCGCTATCCTAGTCTCGTAATGAGTAATTAATTGCGTTT-- 72335
Gallid      GCCGCATCTGCATTCCATC-CACCCTCTGCCTCTTCTACCTGTTTGTTGAGCTCGTCTAT 77204
            *   * * ** *  *  *  * *  **    ***  **     *  ** *   *** *

Human       TAATGATTGGCCAT-CCGATTA------------ATCTAATAAACGA--TAAAGGAGT 72380
Gallid      CAACGATGAGATATACTCATCATTTGTTATTTCCATCCAGTCCTCCAACTCCATTTGACG 77264
             ** ***  *  ** *  ** *            *** * *   * *  *  *   **

Human       CATCCGCTGATGTTGCATTGTTGTTAAAACCATGCGTGTTCAGGTACTTGGTGATTAGAG 72440
Gallid      CACACGATCACCTACCATTTTTAATGCGAGCATGTATACCGCTGCATGTGGTGCTGCGGA 77324
            **  ** * *  *  **** **  *   * ****  *      * *  ***** *  *

Human       TGTCT---GTAGCTATGTT--AACTTTGTTCCACAGTTGTCTGGATTTTAAATCC--ATG 72493
Gallid      TTCCTCGGATAGCACCATTCGGACTCTAT-CTATAAGCGTGGTTTCTTTTATTCCCGAGG 77383
            *  **    ****    **   *** * * * * *   **      *** * ***  * *

Human       AAACTTAGGTTTTCTTTAGGGCAGCCTTTATCCGTTATGCTATAGATTAATTCTTCGATG 72553
Gallid      AAAATATTGGCTGTTCTGCTACAAACCCAACCCG--AGGGCATGTCATTACATAGCGATG 77441
            *** *   *  *  * *    **  *   * ***  * *  **    * *     *****

Human       GGAGCCTTCTTAGTACCGTGATGTAGA-AGGTTGTTTAAATCCATT-TGC-ATCTGAATA 72610
Gallid      TGTTCTATCAAAAGACATTAATGAACACACGTTTCTCCCACCCATTATGTTGCCCCAGTT 77501
             *  *  **  *  **  * *** * * * ***  *   * ***** **    *  * *

Human       AACGTCTTGCAAGAATTTCGGTAACCTTCTGGGACGATAAATATGGGAGTTAGCCGTCCA 72670
Gallid      GCCGGACTGGAATACTTTAGTACTGCCAGCCATTCCGTGATACTTGCTGATACTTATGCC 77561
              **   ** ** * *** *     *        *  * *   * *  * **    * *

Human       TGTAGCATTTTCCCTTTGTCCATGTCTGTCTTGTACATATAGCCTATTCTGATGCAGTGA 72730
Gallid      TATTACAACAAAGGGTTTCGTAGTCATAATTGACCCAACTAGATTGGTAGAAGAAAAGGT 77621
            * *  **        **    *    *   *    **  ***  *  *   *   *  *

Human       CCTGTGTGGTAAATGCCGATGTCGAAACATT--CTCCGGGAAATGTTAGGGAATTGAGGA 72788
Gallid      GGCACACCATATATCTTTATGGTCAGACATGGATGCAATGAGCTGCTGGGCGGCAGGGGC 77681
                     ** **    ***   * ****     *   **  ** * **     * **

Human       TCTGACTAAGCTCTTGATCTAAACACAT--AACGTGATTTAAAATCTTGGATAATTGTTT 72846
Gallid      CACGTCTTGCCCTTTTGTTATAGGACGTTCAATCCAAGGCAAAACGATAGCTGGATCCCT 77741
               * **   *  **  *   *  ** *  **    *   ****   * * *   *   *

Human       TAATGGTTCTCCCCCGGCGATCGCTGT-GCCATTAGGT-AATGGGATTGATATTCTAAGA 72904
Gallid      GGGTCGCCTGGAGACAGCAACCAATGCCGAAATTGTATTAATAAAAAGTGTTTGTGGGA 77801
```

```
                   * *        * ** * *  **  *  ***   * ***   *  *   ** *  **

Human           CCGATCTTTTTCCGACAAATACAGAATTGTTTGATTTCGGTTGGATCTTCAGTATA---- 72960
Gallid          ACAGTACTT--CAAAATAGTATTTGCTAGATAAAATTGTGCCAAATCACCAAAGCACGTG 77859
                 *  *  **  *  *  * **     * * *  * **  *    ***  **    *

Human           -GTCTAGTGCGT-CGTTAGTCGTGTCGCACTGGGTTTTGAAGAA------GAAGATGGGA 73012
Gallid          GGACTAATATTTATGTAATTCATATCAGCATAACTCTTTGTGAATTTTTTAATGAACAAA 77919
                 * *** *   *  ** * ** * **    *   * **   ***       * **    *

Human           TGTGTGTGTGTGTCGATGCTTGGAAAAATTTTTTTCCAGGCGTTGAT-GAGGGTAAGTCT 73071
Gallid          GATTTATCTTCTTCT-TCCTTAGAGAGTATACCAGCTGGTAACTGATTGCGTAGGAGCAG 77978
                  * * * *   **  * *** ** *   *     *  *    **** * *    **

Human           TATGAGGCGACACATCGAGAAAAATGTTTCCTCCGTAATGGTTGCTGTATCTTGCAGCGG 73131
Gallid          GATCCAAAACCAAAGAGCATTAGGAGTCGCCCCATTGGGCATTTTAACATCCGGGAACAA 78038
                 **       ** *  *    *   **  ** *  *     **     ***  * * *

Human           AAGATCCAAGT----CTCCTATGTAATTTGTGACTGGTATGCGTTCGT-TGAATATTTCG 73186
Gallid          TAGAGTGTGATAGCGCTTCACTGCAAATCCAAGCGGACCATTGAGAACATGCATAGTCTT 78098
                 ***      *    ** *  ** ** *     * *      *      ** *** *

Human           TGTCGTGTAGTATAGAATTGTTTTTCGAAGTCGTCGCAGAGAAAATGTGTGTCTTGTAGC 73246
Gallid          AGTCG-GTCGT-TGATACGCTTCTACCATACCCGCGAGCCTAGACTTTGTAGCTTCCG-- 78154
                 **** ** ** *   *   ** * * *   *  **     * * * ***  ***

Human           CAGATGGCGCGAGTTAGAGCTTCATCAGAGA------TATATTCGTCGGGGAGATACTGT 73300
Gallid          -ATGCAGCACCGGCGATACCTCCGCCGAACAGCACCTTGTTTTTACTTTAAGCTCTCTG 78213
                 *    ** *  *  * * ** *  *  * *      * * **   *    ** *

Human           AAGAT----GCGGTTAAATCCCATGTTTTCGTACCAATCTTCAGAGTTCCCGAAGCAAAA 73356
Gallid          AATATCTCGACAGTCACCTTTACTAGATCCCCTTCCATATTCGAAATCACGGAATCATAT 78273
                ** **     * ** *  *    *   * *    * ** ***  * *  * *** ** *

Human           AATATTTTTATG-----TAAAAGCTGCA-CTCTGAATACAGGAAAGGGACGGTGATAATC 73410
Gallid          TGCGATTTACTGGGGTCTGGAACACATATCTGTTGGTCTTCCAAGGTGACCGTTATAGTT 78333
                     ***  **     *  **     * ** *   *     ** * *** ** *** *

Human           TT---TGTG-TAGGTGTTGTCTTCTGTAGATGCGCTTATC-TTGTATCTGAACGTATTT- 73464
Gallid          TTAGATGTAATAAACCCGGCATTCTGTAATTCCAATACCCGTCGTTTCAGTACTGCTTGG 78393
                **   ***  **      *  *******  * *  *   * * ** ** * **   **

Human           -ATCGGAAGCGGAGATTTCTAATAATC-CTTGTAATCCTGGTTTTACTGGAGAAAAAAGA 73522
Gallid          AATTGAGTTCTGAAATTTCTAGCTTCAACTTGCTGTCCCTGCATTACTATGGAACACTGG 78453
                 ** *    * ** *******       ****   ***  *  *****   *** *  *

Human           CCTT---CTCCGTTTTTATTACATTTCGCTATGGCACGCGT---TATTTGTTCGGAAGTT 73576
Gallid          CTTAGGGCCATATCTTGAACAACGGCAATGATGGTGCGCCTGGATAAAAACGCTAATACT 78513
                * *    *    * ** *  *         ****  *** *   **      *  *   *

Human           A---ATGCTTGGATGGTCTTCTTTTTAGAGGCCAGAGCATACTCGTATTCTGTAACGTTT 73633
Gallid          GGACATATGCCGGAGGCATATGGTTCTACTGCCAGCGACAGGGTATGATTTGCATCACCC 78573
                    **     *  **  *    **     ***** *        *  * ** * *

Human           AGATCT----GGATTTTTCAGTCTT---TTTATATCGATGTATGTTTTAAA-GTAACAAT 73685
Gallid          AGACCCTCGCGTATATTATATTCTCGAATTTCTGTCAAATTTCGCATAAGCTGCGAAGCC 78633
                *** *     * ** **  * ***    *** * ** *  *  *  * *   *  *

Human           CCGCATTGAAGTAT-TGCTTCATGATACACAGTAAATTTTCGTGCAGGGAATTTCCAAGA 73744
Gallid          TCACTTTCGATTATATTTTTCATGGTCGATATAGCACGTATGAATGAATTGCCATCTCTA 78693
                 * * **  * *** *  ****** *  * *    *  *  *             *   *

Human           ATGTGGACATTTCTCTGGCATTGTTGTCCTATTGGTAAATTAGAGTCGTAAGAAGTGATA 73804
Gallid          ATGTTAGCATC-CGCGGACGCCATGTTCGTAGGATCATCACAAGTTAACAGACCTTCCTT 78752
                ****   ***  * * * *    *  ** **     *    *   *   *     *  *

Human           TTGCAGTAATTG----ACGAATTTTGTCGTC--TCTAGGGCCGTGTTGTAA-GCGATATA 73857
Gallid          TTCCAATGCTTTCATCATTCGTTCTACCGTCAATCTATACGTATCCTGCATTACGCTACG 78812
```

```
                ** ** *  **      *     ** *  ****  ****      *  ** *    ** **

Human           -TATGTAATGGACACGGAATG--TGTTGGTTAGATGTGAACTCT---TTCTAAATAGATT 73911
Gallid          GCTTGCCTCGCCTTCTGATCGCTTGAGGGTCGAAAATTGAGCGTAACTCCCAAGCGCATT 78872
                   **    *    * **  *  **  ***    *  *  *    *   * * **    ***

Human           GATTTGAGTGTCTAGATATTTAAATTCTTCC--TTATCGCGGAGATTTTTTTGTCTGGCG 73969
Gallid          TACATCACAATAATTATTTGTCATTGCTCCAAATAGACCCATAGCCCCACGCATCTGGTA 78932
                 *  * *   *    ** * * * * ** *    *   * *  **         *****

Human           TACG---TTGCGAAGTCTGCAAATAGTTTTCTAAATTTTGATTTCAAAGTAATGTTTATA 74026
Gallid          TCCGAATTTGGGCAAGCGGTATACTAGGCGCTTAATTGTAGTATGAGCACAGTATATACG 78992
                * **   *** * *  * * * *       ** **** *  * * *    * * * **

Human           AATTCGGACATGGAAGAATACGTG-AACACCATGCTTCCTGTGTTTTCCGTAAAGCATTG 74085
Gallid          CGACGTCTTATCACATAATTCGCATGGCATGTCGGCATCTATGGCCGACGAGACAAACTT 79052
                         **   * *** **     **     *     ** **      **  *   * *

Human           AACATAATTTTTGGTAGTTGTGATGGTGCTCTTTTGTTCCTCGAACAAATAGTAATACAT 74145
Gallid          TACAGTATCT----AAATCATGATGGCAAGCTTTGGCACCACCATCACATCGTTCCAAAT 79108
                 ***  ** *     * *  ******    **** *  ** * * ** ** **   * **

Human           CGTTAATAAAAGCAGACCTTCTGTTTGCCCGTAGG-TCGATAG---AAACCAAAAGGGGG 74201
Gallid          AG--AATAAAAATCTAGCCAACAATTGCGGACAAAATCCACAGGCCAAAATTAGATAATC 79166
                 *  *******    * *      ****     *    ** * **   ***    * *

Human           AAGTGGGCAATAGGAATTTACCGGTGAG-ATATTTGATAATGAGCGATTTGTGGAAATAG 74260
Gallid          CAATGAATACTCGGA-TTGGCTGGCGGATGTGCTTTTGGATAAATTTTCTCCTGAAACGG 79225
                 * **   * * *** **  * ** *    *  **     ** *    * *   ****  *

Human           GCCATGTATTTAAATT---CGACGTCCTCTTTTTCTTTTCCAGT-------TTGAGATAA 74310
Gallid          GTTTCCCATCTCGGTCAACTAATGGGTTGGCTGACAAATGCGGTGCCGCTATTTGGAAAA 79285
                *      ** *   *       * *   *   *  *   * * **        **  ** **

Human           TATCATGTCGACATATAACAATTCGGTTTCTTTGTCGTTGAGGC-TGTTGGCAACATCTT 74369
Gallid          ATCTATAAAACGATGGAGCAGTTGTACTCGTTTCTTTCCTTCCGTGCTACCAGCTTCCC 79345
                    **       **  * ** **    *  *** *  *      * ** *  ** * **

Human           CAGCCTTTATCGGACTTGCAGTTTGTATGTAG-TGGCTGAGATTTAATTTCTCGGCG-TG 74427
Gallid          CTACTTCTGTCATGTATATAACTGAATTGGAACTGAACACCATCGCTCCTACCAGCCCTG 79405
                *  * * * **      *  *  *     ** *  **       **       *  * **  **

Human           GCAGACGAATACCGT--GTCGATT-CGCTTTGAATTTTT--GCATTTT----TGAGTTTG 74478
Gallid          CCACGCGGGCCATGTACGCCGATAATGCTTCTATCCTATCGGCATATCCAACTGGATTAC 79465
                 **  **       **  * ****   ****  *   * *  **** *     **  **

Human           AAGGCAGAAGCTG-------ACGTTATTTG-ATGGTTTATATTTAACAATAATCGGAAAG 74530
Gallid          AATATATCGGCCATTTATTCACATCAGGTATAGGGTCCTCATATACCGATGCTGAAATGA 79525
                **   *   **          ** * *  *  * ***    ** ** * **  *   *

Human           ATTAAATGTTCACTGGGACTTCTACATAGGATACTAAAAATGACGTTTGCGGCATCGTA- 74589
Gallid          CATTTTCGATAGCCAAAGTAGCATCCGACGCCATTAAAGAAGCAGTGCGCCCGC-TCATAA 79584
                  *    * *  *         *  *  * *  * **** * *  **  ** ** ** **

Human           -TTCAGTCGCAAACACTACGATGGTCATTGTGTAGAGCTCTG--TTGATTACAGATGAAT 74646
Gallid          CCCCCACCAGATACATCAGATCCTTCCCTTTTTCCCATTTTAGATTGACCCTTAGTAGTT 79644
                   *   *  * ***  *      **  * * *     * *   ****        *   *

Human           TTGGCTTTAAATGCCCTTAGGGT-ATTGGGGTGTTA-TGGGAAATTGTTTTCGACGATTA 74704
Gallid          TCTGCCGCAGGAGTAAAAGCGGTGAACGTTGTGTCAGCGGGCAGTAATGTCCCCTCGTGA 79704
                *  **   *   *       *** *  *  **** *  *** * *  * * *     * *

Human           TCTCTGTTTGGACAGCCGACTCAGGCAAATTATGAGATGTTGTGCTCTTCCGACGATTTA 74764
Gallid          TTTTCATCGAAGCA--TAAATGGGCAGCACCACGTGCAATAGCATCGATATTGCGAACAC 79762
                * *   *     **    * *  *     *  * * *   * *       *    ***

Human           GAAAGTGAAGAGCTGACGTATTTTCTGGATA-TGACGTA-TAAA-GATTTTGGAGTTTAT 74821
Gallid          GAAGACCTACAGCTGTAGTACTGAGCACATAGCCATGCAGTAAACGACATAAGGAATCGT 79822
```

```
                  ***     * *****  *** *      ***   * * * **** **  *  *   *  *

Human             CAGAATGATATTATAA-GTCATCAAAAAGACACGGAAACTATGAAAACT----CTGTTAG 74876
Gallid            TATAAAAAGCTTTTGGCATTATTGCGCCTTCTCCGAGTGCTCGGGGTTTTTCGCTGAAAG 79882
                   * **  *  ** *    * **        * * **      *     *    ***  **

Human             GTTTATTACCAATGTACAAAAAAACTAAGCTCAGGCATACCATTATGGAGCGATGTTTAT 74936
Gallid            GTTCATTGGTAATG--CGATTGAAGTCTGGTAATACTAAATGCAGAGGGTAGATAGGGAT 79940
                  *** ***   ****  * *   ** *  * * *  *  *       **   ***    **

Human             CCAATTGTCCTAACCAC-GTTAAAGATGCACTCTGTGTAGAGCTTATGAAAGCTGAAAAA 74995
Gallid            CCTGCGCACATCAGCATTGTTAATGGT-CACAACGT-CGCTGCCTCCAAAGTGTAAGAAC 79998
                  **      * * * **  ***** * * ***   **     ** *   **   * * **

Human             ATATTACAAACGATGGATGTCGTTTTTATGAAAACTT-TAATTGGCGAGTTTTCTAT-GT 75053
Gallid            GAATTACATA-AATATATGACCTCTTTAAATAATTCGGTGACTACCAAGTACAACATCAC 80057
                    ****** *  **  *** * * ****   **     * * *  * ***     **

Human             GCACCGATAATCTTAATCAGTTGCTTAACAAATTCGCTA-CAGATCAGTCAACACTTAGC 75112
Gallid            ATTTTGGGCATCCATTTCGAGAGCTTCACAAATTTGTTGGCCGGTGGTTTCAAACGCAGT 80117
                       *   ***    **    **** ******* * *  * * *   *  * **  **

Human             GATGTCGAGAAAATAAACAGTCTCATAG--AGATTGATGGCGAAAACAGCAAGCGTCTTT 75170
Gallid            CGGGGCGGGAGACGAATAGGCACTATAGCCAAACCTTTCTCGTGC-CAAATCGCATGCTT 80176
                     * ** ** *  **   *    ****  * *    *  **    **    ** *  **

Human             TAGTAGAGTTGGATCCGATTCTCCATGAGGAGACAGGAT-TATATCAAGCGCTGCCAAAT 75229
Gallid            TGGTGAGATTTGGAGCCTTCGTACAAGGTTTTATGCAATCTCCGCCGTAAAATACAAAAA 80236
                  * **    ** *   *  *  * ** *     *    ** *    *      * * ***

Human             GTTGTG-ACGGAAGCTCCGAGTGAAAAGGTTAAAT-CGATACACGTCGAGTCTGAAGGTG 75287
Gallid            CGCATGGATGGTAATGTGTAGGGACGAGCTTAAGGGTAGTACCCCCACTGCCCAAACCCG 80296
                      ** * ** *      ** **  ** ****      *** *     * *  **   *

Human             AGAGT---GTATGGTCAAGCGTTACTGAAGGC--GGCATAATGAAACAAGAAAAAGGAAC 75342
Gallid            TCGTTTTCGTTCCCGCTACCGCTGCCACGTTCCAAACGAAGTCTGACTCGACCGTAAGAC 80356
                      *   **     * * ** * *      *    *  * *   **  **       **

Human             TGGCGTCTAGTACCTGTAGCCGGTTTATTGCGCGTTCAGGCATTAAAC-TGAGG----CA 75397
Gallid            CTGCAACCAGCGGTAATA-CAGCATCATCACAATCATTGCTTTTTGCCGCGAGGATCGCC 80415
                    **  * **      ** * *  * **  *       *   **   *  ****    *

Human             GAAATGTCCGCAAGTAAAAATTATTCTGGCGATGCAGAT-----GCCTGTAGGGATGTAG 75452
Gallid            AATTCATCCACGGGAACGCTACATTTGGGCGTCGCATATATATAGCCTATCGGTCCGCCG 80475
                   *    *** *  *      ***  ****  *** **     **** * **   *  *

Human             -ATAGTTTTGCG--------TGTAGAGTTGAAGATCTTTATGGAGGG-AATTGT--GTCT 75500
Gallid            CAAAGTTTCACGCTTTTTCCTACACCATCCATAATGTTGTTGTTGGATAGTTGACAGAAT 80535
                   * *****  **        *  *   *  *  ** **  **  **  * ***   *  *

Human             TTGTTCCAGATGTAGGTTTCAATCAATAAGCCGAGTTTGATAA----TTTCTTTGGAGGG 75556
Gallid            CTAGTGCGAGAGGAATATAGAATCAGAGTAGCAGATGCCACAAACACTATCTCCGCCCGA 80595
                   *  * *    * *   *  *****      *   *   * **    * ***  *   *

Human             AATCATGAGAGCCTTTAGTTCATCGGCGCAAACGTA--CATGGCGTCGAAGCATTGTATC 75614
Gallid            AGACACGGTACACTTCATCATGTGGGCTGTTTTAAAGCTATAGTGCTGAAGGGGGATGTG 80655
                  *  ** *  *  *** *     * ***        *   ** * *  ****     * *

Human             GTATAGCT--GTTCGGTTTAATTATAAATTCTCTGCATGTCTTTAGATG---GAAAACTG 75669
Gallid            ACGCAGCTATGTTTATGTATGACATGAGACACGTGGGATAGTGTAGGTGTATAATAACTC 80715
                      ****  ***    *     ** *      **      * *** **    * ****

Human             CAGATTCGG---GGGGAGACAGATTGA---TCATCTCCCATGGTTCCGGGATGTAGCAAG 75723
Gallid            TAAGTCACGTTTATGAAGTAGGATTGGGGCCCAATTCTGACGATATCTGCAT-TGGCTAC 80774
                   *  *   *     * **   *****     **  **  * * *  * * ** * ** *

Human             GGAGAAAGTTGATTGAGAATTCGACATCTCCTGGGAGAAGCATTAGGGAGTGCTCGGATA 75783
Gallid            TATGTCAGTAGAC-GGAACTAAAACATTTTTCAATCCGTACATCGGAGCACGAAAGAGGA 80833
```

```
                       *  *** **  *  * *   **** *           ***  * *   *   *   *

Human               AGTTCATTAGAAAAACTGAAATGTCGGTTTC-GTCGGTTATAATTTCAGTGATG----CA 75838
Gallid              GTCTTGAAGCAAGGAATGGCTTATCATTTTCCACCGGACAAAATTATGATGAAAAGAACA 80893
                       *      **  * **   * **  ****   ***  * ****    ***      **

Human               TTCGCAGAATACGTCGTT-----------GCTATGGCCAATAATGACTCCGAAGAAACCA 75887
Gallid              ATCGACGAGATCGTAATTCAATCACGTATGTTACCACTATAGATGAGTTTAAATATATTG 80953
                     ***  **   ***  **           * **   * *   **** *   ** * *

Human               -TCCGGGATGCACAGGGAAATATCTAGTTTTACGATTCGAACTTGATCGTGCGGCACCCA 75946
Gallid              CTCCCAAATGCCTAGATGACAAAGATGTAAAACAAAAGGGAACTCATATCGGCAAATTGA 81013
                     ***   ****  **   *  *    **   ** *   * *  * **   *    *   *

Human               TATGAGTTCTC-TATT-TACGAGTGTGATTTTGTTGGCGTCCATAAAAACGTCCA---TT 76001
Gallid              AAAGATCACCGGTACTATACAAAAATGGTGAAGAATATGTCTTTCTAAACTTTGAGGATT 81073
                     * **   *   ** * *** *   ** *   *     ***  *  **** *  *   **

Human               TTAC-----TATTCCTGTGATGGCTTACTA----AGATAGATGGTTCTTCCGAAATACAA 76052
Gallid              GTGAGGATGTGTGGCCGCGGAGGTGTTCTATATGGAATAATAGATCATTCCTCCCCGCAG 81133
                     *       * *  * * *  **  * ***      ***   * *  ****      **

Human               GTGTCCGTATTTGCATTTCT---TTTTGCGCG-GTGTCTTGGGTAGAAAGTTACTGGGTT 76108
Gallid              ACTTTGATCCACGCTTCTCTAGGTTTCATGTATATGACATGA-TAGAAACTGTGGAATTT 81192
                       *   *    ** * ***   ***   *    ** * **  ****** *       **

Human               GCCA-TGTTTCCTTACATAGTTAACACAATCGGTTTCTCTTTGCT--TTTCTCTTTTGCA 76165
Gallid              GCAAGCGCCGCCATAGACAGA-GACAAAAACCGTTTTTTGGAGCTGCTTCGCCCGATGGG 81251
                    ** *  *   ** ** * **   *** ** * **** *    ***  **   *   **

Human               GGTCTTCGACACTGT--TCCATTTTGCGGAATACCTAGAAGCGAG--ATTGGGCGTTGAT 76221
Gallid              GACAATTGTTACTATGATGGGAATTACAGAATGTG-GCAAGCGAGTAGCTGTGCATGTAT 81310
                    *    * *  *** *  *     ** * ****      *******    ** ** *  **

Human               AAGAGGTTAGTTCGTTTTCTAGAAATTTTTTAAATGATTTC----TCATTTATAGCTGGC 76277
Gallid              ACGGCATCAAGCCATATTTTTATATGCGCAAAGTCGATACTGATACCATCTGCGGAAGCC 81370
                    * *   * *   * * ** *   *       *   ***        *** *   *  * *

Human               CACA-CATCGTGCGTTTTGTCTTCAGATATGTCTGTAGTCCAAAGTTCATTGATGTCCAC 76336
Gallid              GGTGTCCTCGTGAACTTG--CCGAAAAACTGGCAAATGTCGTACGTTCCTCAGTAAACGA 81428
                         * *****   **   *   * *  ** *    ***  * **** *   *   *

Human               GTTTCGAAAGACGAAAAATTCAATGTATCTTGAGCTCGTTTGTCTCTGTAGGGTAATCGT 76396
Gallid              GGTTGCGAATGCAAAACGTTTT-TGCACTCCGGTCACGCGTA-CTGTATCGGCAGACTGC 81486
                    * **   **  * ***  **   ** *    *  * **  *  ** * * **   *  *

Human               TTCAGAG---ATCTTTTCTGAAATTACGCTGTACATTTTTCCAG-----GTGACAGAGGG 76448
Gallid              TTTGAAGTAGATGTCGTACAGCGGAAAGATATATATTATTATGGAACTGGCCATGACGAA 81546
                    **   **   ** *  *        * * * ** *** **   *     *  *    *

Human               CTGTCTGCTGTTAA-TCACGTCGACGCGAAG-----ACTAGTTCAAAATAACACTTAAGA 76502
Gallid              TTTTATAGGGTAAAATCCCAGAGCGGCAAATTCATTACTCTTTTATGCGATAACTTCTAT 81606
                     * * *   ** ** ** *   *  ** **      ***  ** *    *  ****

Human               TGAGTTGTAAGAAAGGCGCAAGGCAACGCTTATATGTCAGTCTCTGGCTC-TTTTACATT 76561
Gallid              CCCTCTATAATCAAATATGAAGGAAACA--TAGATGCCATCACCCGAATGGTTCTAGATA 81664
                         * ***  **     **** ***   ** *** **    * *  *  ** ** **

Human               CTTGTTTTTGCGGCCGCTACGGA-AATGGACTTTTA---TTCTTCAGAGTGCCATTCGCA 76617
Gallid              ACAACGGATTTAGTACATTTGGATGGTACTCTTTCAAAGTTGGTAATAATGGCGAGAAAG 81724
                            *   *    *  ***   *   **** *   **  * * * ** *

Human               CACCTATGAGATCGTCTTGAATTCATTTTCTTCGATCTGGCTTTTGATAAATCTTTTTTT 76677
Gallid              TGCAAGTACGAGCTCCTTGTCATCATTGTACGTCATGTGACAT----CGAAATTAATTGC 81780
                      *   *  ** *  ****   ***** *     ** ** * *      **  *  **

Human               ACTCTTGTGTTCTTTTGCGATTTTCTTGAAGTACTGGTGTTATAAGACTTTTGCTTCAGA 76737
Gallid              ACTGTAGATAACTTGATTGGATATCCCGAAGATGATGCATGGCCAGATTACAAACTTCTA 81840
```

```
                *** * *    ***    *  * **  ****     *  *    *** *       *   *

Human           GACGGTGAAG--GGCTATTAGATCGCATGAGACGCCTATTCT--AATTATAAATGTAGCA 76793
Gallid          TGCTTTGATATTGAATGTAAATCCGGAGGAGTAAACGAATGTGCATTTCCATGCGCAACA 81900
                  *  ***    *  * * *   ** * ***    * * * *  * **  *   * * **

Human           CACGACAAATAAAAGCATTTTTTGATGTAAT-TGGATCGTGTTTTATTATCTCCAATAGA 76852
Gallid          AATGAAGAAGATGTGGTCATTCAGATTTCTTGTCTCCTATATTCTATTAACACCAAACAG 81960
                 * **  ** *   *    **  *** *  * *      * ** ***** * ****

Human           TTTCATAACGA--CAAAAGACAAACCTGATTTCAGA--------ATTGAGTTTTTATTGA 76902
Gallid          TTGGAGCATGCATTATTGTTCGCTCTTGGTGCCTGTGACCTCCCACAAACCTTTAAAGAG 82020
                **  *  * *    *     *   * ** *  * *          *   *  *** *

Human           AGACTGAGGATGAGTAGCTTTGACAAACGTATGCGGTTATGTGCTATCTGTGTTGTTTAA 76962
Gallid          ACGTTTCAAAGTAGTTACAATATTTTGCCTATAGTGTTAGAATTTGACAGCGAATTTGAG 82080
                *   *    *  ***  *  *      * ***   ****      *  * * *   ** *

Human           ATCGTGT---CGTTCGTTGTATGTGTAATGCGTTGCATTTTGGGTTGAGTATGCTTTGGT 77019
Gallid          CTCTTACTCGCGTTTATGACTTTTATTAAGCAGTATGCTCCAGAATTTGTAA---CTGGG 82137
                 ** *     ****  *    * * * * **  *    *   *  *  ***     ***

Human           AGTGAGGTTATCTGGATGGTTGTGTGAGTTTTTATCGGTGACGCGGTAGTCGTTTCCAGG 77079
Gallid          TATAACATTGTCAATTTTGATTGGGCATTCATTGTTACCAAATTAACAACAGTATACAAT 82197
                  * *  ** **    * * *  *  * *  ** *     *      *   ** * **

Human           CAGAGTTTTGTTAGCTTCCCGACTTTCTGTGGAACTTTTTTTTTAACGTCCGTAGATCG 77139
Gallid          ATGAGGTTGGATGGTTATGGAGTGGTTAATCAAAAAGGGATGTTTAAAGTGTGGGATGCA 82257
                  *** ** * * * *         *   *  **      * ***** **  *     *

Human           TTCCGTGATTGTTT-------GGGGGTAGATGTTAGTCGTTTGTGACGA-GACGTGAGCT 77191
Gallid          GGAACTAATCGATTTCAAAAAAAAGGCAAGTTCAAAGCCACTGGAATGATAACGTTAGAT 82317
                     * ** * **         ** *  *   *  *   **  * **  **** ** *

Human           TT-TGTTAGTTTG--GCTGATTGAGTTCTGTTGCTA-GTCGTTAGCTG--TGTTGTGATA 77245
Gallid          ATGTATTCAATTGCGACAGAAAAATTAAAGTTGCAAAGTTACAAGTTAGATGTCGTGGCC 82377
                 * * **   ***   * **   * *   ***** * **    ** *   *** ***

Human           TGATTTGTCGACCTTAGACTTGTATTCGTTAATGGTTCCTTTGGCGTTGTT-AGTAAGTT 77304
Gallid          GAGGCTG-CATTAGGAGAACGGAAAAAAGAATTGTCTTATAAGGAAATACCCAGCCATTT 82436
                     ** *      ***   * *      * **  *  *  **   *    **  * **

Human           TG-ACCAGGTT----AGAATGGCAGACTTT--GAAGATGTCGTTAAGGACGTTTCAGCGG 77357
Gallid          TGCAGCAGGTCCAGAAAAACGGGGAATTATAGGAGAATATTGTCTTC-AGGATTCATTGT 82495
                ** * *****     * ** **   * * *  **  ** * **     * * ****  *

Human           TGAAGGGCCATTGAGGTGTAGTCGATTTTTCCCATGTGTGAGCGTTAAGAATTG--GTGT 77415
Gallid          TGGTGGGAAAATTATTTTTAAATACATTCCCCATCTAGAACTGTCCGCAATAGCAAAAT 82555
                **  ***  * * *  * *      *  ** ***** *   *  **    *** *     *

Human           CATTCGTGGTATTTCTGTGTGGTTGATCTCTCCGTGAGTGG-----ACGCCGACATTCTA 77470
Gallid          TAGCTGGTATATTGCTGTCAAAGGCTATATTCGATGGACAACAAATACGCGTGTATACTT 82615
                 *   *   **** ****             **  **          ****    ** **

Human           TTTTGCTTTGGTTCTTCGG------TGACTTTAGTGATTGAGTGTGATGAATTACTAGAT 77524
Gallid          GTTTGTTGCGTTTAGCGAGATCGCATGGCTTTATTCTTCCGGAA--AAGAATAAGAAATT 82673
                 **** *  * **      *      ** ***** *  *   *    * **** *  *  *

Human           GGT-AAAGCATTGTTAGTTACCTGTACTTCAGTTTTGGCAGTTACATTTGACGGTAGTTT 77583
Gallid          TGCCGAAACGGTGTCCCTTACCTGCGAGGAAGATCAAACAGAAATATGTGAACACGATTC 82733
                 *   ** *  ***   *******      ** *    ***  * ** ***       **

Human           TTTTTTTGGAGTGACAGACAACGTCTGAACGGTAGGTGGCTCGCTTGAGGATTT----CA 77639
Gallid          TCCCCAAGAACCCATTCATAATATA-AAACAATCATCACTTTGTCATAGCAATAGTGGCA 82792
                *      * *   *   * **  *   ***  *       * *    ** * *      **

Human           AAGTTGTCCTCT----GGAATCTGTCCTCTTTGAAGGTTTTGACGGTCTGCACTTCTGTG 77695
Gallid          GAACTATTGGCTATCAGGGCGCAAAAGTACTGGACCCCATATCCGGATTTCACGTCGATC 82852
```

```
                 *  * *   **    **   *     *  * **     *   ***  * *** **  *

Human           CTATGGGTTTCGTTTGCGTATTCTGTTTGGTTTT-----TTACGTTGAGAAGTTGGGTAG 77750
Gallid          CCGTGATGGTCTTTGACTTTGCCAGCCTGTATCCAAGTATAATACAAGCACACAATTTAT 82912
                *  **    ** **  * *   * *  **  *       * *       *       **

Human           GATTTGTGGGTTCTTTAGGCG-TGATTCCCATC----------GTTTCTCCAAGAGAAAT 77799
Gallid          GCTTTACGACACTAGTACACGATGATACAAATTTAAGCAATTTACGTCCCCAAGATGACT 82972
                * ***  *       **  ** **** *  **              ** ******  * *

Human           TTTAATAAAATTTTCGGTCATGTTTTGTACTTCCGTGTTGTGG---CTCATGTATGTGCG 77856
Gallid          ATTTG-GAAATCAACGTGCAAGGCAAGCTATTGCGCTTTGTGAAACCGCATATTCGCGAA 83031
                 **    ****   **  ** *    *   ** **  *****    * *** *  * *

Human           CGCC-----GCG-TCTTGCCAAGTGGTAAACT--TTAAAATCGGAGTGGTGTGTTC---G 77905
Gallid          AGTTTATTGGCGATCTTATTAAAAGATTGGCTGGCCATGAGAAAAGCAATTCGTGCTAAA 83091
                 *       *** ****   **  * *   **    *  *    **   *  ** *

Human           GTGTCGGTGAATTTTGTCG-------TGCTCTGCTGTGATTTGCTCTTAA-GATAAATAG 77957
Gallid          ATTCCTGAAAGCTGCGACGAGATTGCCGTTCTTCTG-GATAAGCAGCAGGCGGCCATCAA 83150
                 *  * *  *  *  * **        * *** *** ***  **       *   *  *

Human           AGTTTTTTGG----TGAAGAATAGATATTGTCGCTAAT-------TTCCTCGATTCCTAT 78006
Gallid          AGTTGTATGTAATTCGGTATATGGTTTTTGTGGCGTATCGAATGGTTTATTACCTTGCAT 83210
                **** * **      *    ** * * **** **  **       **  *    *  **

Human           TGTTGTTGCGAAGGTGGTGCTTTCGAGCTTTTGAGTCGTGTTTGTTGC----CACTGTAG 78062
Gallid          AGATGTCGCTGCGACTGTGACTACTATAGGTCGCAATATGTTGCTTACAGTACGTGACTA 83270
                 * *** **   *   ***  * * *    * *     ****  ** *     *

Human           TGCTCTCAAACGTTTCGGGAATTCTGATTGTGGTATTTTCAAAATTTGTTGGTTTTGGTG 78122
Gallid          TATTCACAAACAATG-GGGTACTAGAGATGCTTTGCTCCGCGAATTTCCTAATTTGAGTA 83329
                *  ** *****  *  *** * *     **   *  *     *****  *  ***  **

Human           GACTTTTTGGGTTTTCGGTAGGGTTTT--CGGTGTTTTGTTCGGTTTTAATAGTGGCGTT 78180
Gallid          -ACTTTATGCGGCCAGAGGATTATTCTGTCAGCGTTATTTATGGAGATACAGACTCCGTT 83388
                 ***** ** *      * *   ** *  * * *** * *  **   **       ****

Human           ACGACGGATTGTAAGAGTTGGAATGTCAGTCGGGGTGTACATCGGTCGAGGAAAATCTG- 78239
Gallid          TTTATCAAGTTCAAAGGAGTAGATATACAT--GGACTCGTAACGACCGGGGACGATATGG 83446
                   *   * *  **  *     ** *   *  **      * **  ** ***  ** **

Human           ---AACGTTTAACTACTTTTTTGATGCTTTCCGTTGTTTGCGTTGTTATGGTGGGTGTGT 78296
Gallid          CAAAGCGCGTATCATCAGATTTATTTCCCAAACCTATAAAACTTGAATGTGAAAAAACTT 83506
                   * **  ** *  *   ***  * *       * *     ***      *       *

Human           TCGGTGTAACTTTTATGGTGTCTGTCTGCAGTTCTGTAA-AGTTTTTTGTTACGGGGGAA 78355
Gallid          TCAATAAGCTGTTACTTATAACGAAGAAGAAATATATGGGAACTATTCATGGCGGAAGGA 83566
                **  *      **  *  *  *       *  * * *   *  * **  *  ***  * *

Human           GGTTC-TGGGGTTGGTG--TGTTGGTACTAGGGATATGTGGTGATGTGATAGACGCTGAC 78412
Gallid          TGCTAATGAAAGGAGTAGATATTGTGAGAAAAAAATAACTGCCGAT-TCATAAATACATAC 83625
                 * *  **      **   * ***  *  *   ***  **   *** * *** *  *  **

Human           GTCGATGA-TGGCGTCGATGATGGCGTCGTAGTCAGTATCGAATGAAAGTTTACGATGGC 78471
Gallid          GCAAAAAAAATTAAGTGACTTGCTATTTC-TGGACGACACCGTGGCAAAAGCTGCTGCTAC 83684
                *   *  * *   **   *      ** * * *   * **    ***   * *     *

Human           TCGTCCAAAGGAAGTGGTAACATTTATAGGTTTTGGCGAATGTTCGAAATA---TTGATC 78528
Gallid          AGTTGCGGAAAAACCTCCGTCGTTT-TGGGCTACATCTCCCCTTCCAGAAGGGCTTAATT 83743
                   * *  *  **       * *** * ** *    *     *** * *      ** **

Human           ATTTGAACGGATGCATGAT---GTTGTGTTATAGAGCAAGAAGTAAACAAAGCTGGTGTA 78585
Gallid          CTTTTGGCGGTGTCCTGGCCGAAGCGTATACTCGAATGATGA-TAAACAATATAACTGAG 83802
                 ***   ***   * **         ** *  * **   *  * *******      **

Human           ATTAAAGAAGATTTCAGGAGTG---AACGGAGTCAAT-GACCGTGTCAGATTCGAGTTGG 78641
Gallid          GTGGAAGACTTTGCCATGTCTGCCGAATTGAGTCGGCCGCCCGACGCCTATACTAACAAA 83862
```

```
                 *  ****   *  ** *  **   **  *****    * ***   *  ** * *

Human            AAAATTTACTGA-TTGCCATCTGCAAGGATTCGTCGGTCAGTTTTATGTCAAATAACTGT 78700
Gallid           AGAATTCCTCATCTTACCGTTTATTACAAATTGGCCATGAGGTCCG-AACAATTGCCTGT 83921
                 * ****       ** ** * *   *  * * * *  * ** *      *** *  ****

Human            GTTCC----CAGAATTTTAAACGGTAGTAGCTCTACGCATGGTGGATCT-GCGGTATATG 78755
Gallid           AGTGAAAGATAGAATATCATATGTTA-TAGCTGCGGCTACACCAGAAGTAGTGCGGGATT 83980
                   *       ***** * * * * ** *****       *     **  * * *    **

Human            TAGATAGTGTGGCAAA---CAGAAGATAA-------------AATAGAGAAAAACTGCTA 78799
Gallid           CTGCCCGTGTAGCAGAATTCAGAGGGGAGTTGGATCTCTGTCATCAGAATARCAACACTT 84040
                   *   **** *** *   **** *  *              *  ***  *  *   **

Human            GCGAC--GATAGATTTGTTTGCAGCTGTTGAGGATAGCACTATTTTGGTTTCTGCCCGCG 78857
Gallid           CCTGCCCGGGGGATTCAGTCATGACTAACAAAGAAACTTATGTGCGACATTCCCCCCGAA 84100
                  *  *  *   ****   *     **    * ** *    * *      ***  ****

Human            GTAAATTCTGTAATGGAGACAAGTTA----ACCGCAGACTTGCATT-GTAAATCTCGAAT 78912
Gallid           ACAAGTTATTGATTTCCGATATGGCAGAAGATCCTAAGTATCTATTAGCGAATAATATTC 84160
                   ** ** *  * *   ** * *  *    * *  *    *  *** *  ***

Human            GTTCCAAGAGAAAACGTTAGTTAAAATTTGAGTGAAGCAGTCGAGGGATAGATTT----- 78967
Gallid           CTCTCAATACAGATTATTACCTCTCACATCTGTTGGGGACACTATGCGTAACTTTTAAGG 84220
                  *  *** * * *   ***  *   *  *  **   * *  * * *  **  ***

Human            CTCTGACAAAAAGTTGTATACAAGTTTCGGAGGTCGTA--AAAATGTTCTCTTTGTTCGT 79025
Gallid           CTTTGTTCGGAAATGATGTGAAAATAACGGAAACTGTGTTGAGAAGATTTATTCCAGAAA 84280
                 ** **     ** *  * *  ** *  ****    **    * * * * * **

Human            CGCTCC-----AATTGTGG-----CGGTTGACGGTTT---TGAAAGCTTCTAAGGGATCA 79072
Gallid           CGTTTACAGAAGATTGTAGTTACACGGAACGTGTCTCCAGTGAAATGTTCACAACGATAC 84340
                 ** *        ***** *     ***     *  *    *****  ***  *  ***

Human            GATGTGTATTTTATT---AGATTAACGGAGTGCATGACTATCGCGAAAAGGGCCAGAAAA 79129
Gallid           GAAGCGGAATCGGTTTACAAGTCAACGAGGAGGAAGAAACTCGTCGAAAGCTGAATATAG 84400
                 ** * * * *   **   *  * ****  * * * **   ***   ****    * * *

Human            A-------GGCCGAGTCGCGAGATGTGAAGCATGTCGGG--TGAGTTGACCACAGTGAAT 79180
Gallid           CTTTCCGTATTCTAACAGCAACTCCCCATCGATATTAAGATCGCATAATTTACAATAAAC 84460
                            * *   ** *      *   ** *   *   *  *     *** * **

Human            TTTTACAAGGAGAATGTGTAAC----TAAAATAGTATTTGCTTTTTTTATAGGGTTGGAC 79236
Gallid           ATCTTCAGCATTAACCGCTGGCACATCACAAGAATCTGTTTGTCTGTCATATTCCTTTCT 84520
                  * * **     **    *  *     * ** * * * *   * * * ***    *

Human            GATGACCTCCAAATGCATGCTGTGCTGGACTGCGATTGTGAATGCCCTGGCTATCTTA-A 79295
Gallid           AATTACTAACAAAAAGTTAGTACGCCATAC-ATGAGCAATTATGCGGTAGCTCTTACATG 84579
                  ** **   ****    *  *  **   **   **      ****  * *** *   *

Human            TGTTCTTACTAGTAATTTATCGGGCGCTAATCGGATTTAATGACGATATTTTCACGAGCA 79355
Gallid           CACTCTTCATA-CAATCTATTAATCTGTAGTGGA----GGTGGAGAGCCTTTCCGGGGAA 84634
                    ****  **  *** ***    *  ** * *       **  **   ****  * * *

Human            CGTTGTCGGCGTTTAGCTGTATACGGACAAATCTATCTAATG--CCAAATATAAATAAAA 79413
Gallid           AACGATATACATCA---TATATTCCGACGATCCTGCTTCACGATCTAAGTA-AAATAGCA 84690
                      *   * *     * *** * *** *  **   * *  * *  * ** ** *****  *

Human            CGTTTA--TTCAACACAATCTGATGAGTCTATATAAGCCGAATAAAGCAATTATTGCGAT 79471
Gallid           CTTTCACGTTCTTTATCCCTGAATCAAGTAATACATAATAAGCAAAA-AATAGTTCTGGT 84749
                 * ** *  ***   *    *  ** *    *** *     *  ***  ***  **  * *

Human            CAGAACATAA-ATGA-TAACCA-ATATGATAGATACTTGGTCTATATCGATTCCGACTTC 79528
Gallid           ATTGCTATAATACGAGTAAGTATAGATTCCATATCTTTATCCCGCGCATCTCCCCGCAGT 84809
                       **** * ** ***  * * **   * **  **   *        * **  *

Human            AGACATCTCAAAAACAGAGCCGTTTTTGGCTAA--CAAAAGGTAGTGCGTCCTGGT-GTT 79585
Gallid           CGATCTCCCAAGACAATTATAGAAGCCAAAAAAATCCTATAATGATAAATGTTATTTATC 84869
```

```
                 **  ** *** *  *     *         **  *  *   *  *   *  *  *  *

Human           GGGAACAAGTAAATTGTTGTTGGGATCGGTCAGTGTTTTTAGTTCGTCTATGTTTTTTAT 79645
Gallid          TGTTGTACATATGCCAATATTAAAGCTGCTCTGTCGTTTCGTCCCAAACGTGGATCTCCA 84929
                 *    *  **      * **      * ** **  ***     *     **  * *

Human           GTAGATGTAT-TGTAAGGGACCGTCGAT-ATCATCATATTCCATGACTACGCTCTGACAA 79703
Gallid          CTAGATACGCATGTAGGACAACATCCACCAACACCA-AGATAATATCCCATCCCAGATAA 84988
                 *****     **** *  * * ** *  * ** ** *    **  *    * * ** **

Human           A-ACTCGCA--TGTTTGAGATGATATGTTTCGTAGCACAAGCAGATCTTGTC--CTGCAT 79758
Gallid          AGACAAACAGTTATCCGCAACCGCCTCTGACATATCAAATGGTAATGATATTGACGTTGT 85048
                * **   **  * *  *  *     * *  * ** ** * *   **  * *   *    *

Human           ATTTATAATTTGTTGAAACGCAGGTAGAATTGAGA--GGTATGGCTG-TGATTATTATAC 79815
Gallid          CTTGATAAGCGGTGTAACTAAATCCTTGACTAACATCAGTTCATCAGCTGGAGACTCGGC 85108
                 ** ****   **  **    *      * * * *   **    * * **   * *   *

Human           T-CGTAGCCACGATTGTCGTTGTAATTGTTAAACTCACTCCTTT---AATCAC-ATAAGA 79870
Gallid          GATGAATTCAAAATAGGCACGATATATATCCATATTAGTCCCTTTAGAATTGCCATAAGA 85168
                   * *  **  ** * *    **  * *  *  * * *** **   ***  * ******

Human           GGGAGAAATTACAAATGTCACATTCGGCAGAGGCAGAGAAGCTGCTGGCTCA------CT 79924
Gallid          TGGTAAATTCATATCTCTCCATTGTCGCCGACTCCGTCCAACTAGCCTTTTATCGCGCCC 85228
                 **  ** * * *  * **   *   ** **  * *   * **      * *      *

Human           TGGAGAGAGACACTGAATGGCCGCTACTCTCGCCATATCTGGCGGTCTGTATACGGATAG 79984
Gallid          TATAGATTTGCGTCTCACTAAGGTATGACCAGTCATATTAAG-AATTAATATATTACACG 85287
                *  ***    *     *     *     *  * *****   *   *   ****      *

Human           CATCTCCATCATGTCTGATCGATAACGGTCGAGGGAAGCGTACGT------GAGAAC--- 80035
Gallid          TAGACTCCTAATGTATGCTCGAGAGATATCAAGGAGATGGAATGTCTTTGCAACAATGCC 85347
                 *    * * **** ** **** *    ** ***  *  * * **       * **

Human           ----TGTTTTTA-CAAAAGAATTTGTTACATCTCTGCGTACGGGAGAGGC---ACAAGGC 80087
Gallid          CCAGCGTCTTTAACGAAAGCAGACAGTGCGGTTGGACGGGGAGGAAAGACCTGATAAGTT 85407
                     ** **** * **** *     * *   *   **    *** ** *   * ***

Human           GAATAAATATTTTCTAGA-TTCGGTGCCTTGT-CAGCTGTAAGCATGTGGGCCCATGAAA 80145
Gallid          TTAAACGTATACGCCAAAGCCCAAAGTCCAGCACATACGCGACCTTCAAAATGGTTCTGA 85467
                  * *  ***   * * *   *   * *  *  **   *  * * *         *   *

Human           CTATTTCTACGGGGTT------ACACAGGTTTCCAATTAGTAGTTGCATGTGCCTCTGCA 80199
Gallid          CTGGCAAGACGGGCTTTATATAATGCTAGTTCGTCATGATTGGTGGCATCTGGGTGATCA 85527
                **      ***** **      *  *  ***    ** * * ** **** **  *   **

Human           TG---CGTTCATCGGCAGGGATGAA--ATTCGC------TATTTCGGCTAGCATGA---- 80244
Gallid          AATAACGATCGTGGATTGGTCTGGGCTATTCGTGCCGCACACATTGGATCGCAGAATAGG 85587
                     ** ** * *   **  **    *****        *  * ** * ***  *

Human           -----ATAGA--GTTTCCCTATCGGAAAATGTCAG-ATTCGTAAAAAACATGTGTTTTTC 80296
Gallid          TGTTTATAAATGGCCTCCGTTCCCGCGCTTTTCATTAATGTTACAAAACATCCTTCGTCC 85647
                     *** *  *  *** *  * *    * ***  * *  ** *******   *  * *

Human           ATAGATCTTTTGTAGAGTGTTGGCAATGTCAGACCACTTGTACCTGGAAAT-CGTTACAT 80355
Gallid          CCCAAATCTGTACGATGTCCCTGTGATCGCCAAGCATCTAGAACAGGTTCTATGCTATGA 85707
                    *   * *     **    *  **  *  * **  *  * * **   *  * **

Human           TGGTTTCGTACGCTGTCG-CTAAGTACGTGA--GCAGCGTAGATACGGGAATGGATTTGT 80412
Gallid          AATAAACCTATATTGTTGGCCGAATATGTGATGATATCGCGTTTAAATTTACGCATTGCC 85767
                      * **   *** * *  * ** ****    * **    **     * * ***

Human           TTTTGGTGATGTAGCTATAGTCCGAGGGCTCGTATTTTAAAAATTCCTT---CATTTTTG 80469
Gallid          AGCCAAT-ATGTACGTATCAGCAGCAGG-TTACATAATAAACACTCCGCAGATGTTCCTC 85825
                      * *****  ***   * *  ** *   **  **** * ***       **  *

Human           GGTATTGTATCGTACCTTCCGAG-ACGTTCACAC--ATCGGTTGACAAATGAGTCGATGG 80526
Gallid          CTTTTGTTATCGCGTGCTTTAACTGTGTTATCATCAATGGGCCTAACTCTAAATCGGGTT 85885
```

```
                  * *  *****     *   *    ***  **   ** **   *    * * ***

Human           ACAAGAGATTTTCGTG-TGTCTTTAACACTCT-TAACACGGGCATCGGTCCTGTGTTTTT 80584
Gallid          CCGAATCCTCACCGTAACATGCACTGTATGCTGCAGTAACGTCATTAGCAAATTCCCTTC 85945
                 * *    *   ***    *       *  **  *  *  * ***  *     *   **

Human           TGTGCTATATGAGAAATGTACGATACCGTAAAGAAACATCATCTGCGGTATGCCGGCATA 80644
Gallid          TATGATTTTTCCAAAATATATC-CACCGTT------TCCCTTCTAACTTTTGCGGCCATG 85998
                * ** * * *   **** **    *****          * ***    * *** * ***

Human           GTCGGGTTTTTTACAGTCTCCCCTTAATAGGGTTTGGATTCCTAGGTTATTAAATTTGAG 80704
Gallid          AATGCGAGTTC-GCTCGCTGCCTCAGGTGCAGACCCCGTTCCC-GCTAATAACAATAAAA 86056
                   * *  **   *   ** **     *   *      ****  * * ** * * *  *

Human           TAGTAGAGCTTCTACTTCATCTACGTCATCCAACGTTT---CGTCTACGAATGTCGCATC 80761
Gallid          CGAGGTTGCCATAACCCATTGTACAATCCTCATTTCTTGCATGTTCATGATGTTCGCAT- 86115
                       **   **     * ***      **    **    **  * **   ******

Human           TGATATGAAACGGTATTCTTCATCGACT--ACGTCTTTTTCGACTATCAATAGTAGATCG 80819
Gallid          TGAGACGCATCTATCTGTTGCATGTCCTGAACATTTTCCACCACATTTAACTTTTCTTCA 86175
                *** * * * *  * *  * ***   **  ** * **   * **  * **   *   **

Human           TCTG-TCAATGTCTGTTTGAATATGAATGAACTTT-TCCTATAAGGGGCTTTAAAGTTTA 80877
Gallid          CTTGCTCGGAGTTCCCACTCGTGTTCATAATCATTATCCTCGGACGATGCGCGCAAAGTA 86235
                  ** **   **         * *  ** * * ** ****   * *        *   **

Human           TGTCGGTGGTATTTCCATACATCATAGTC-AGATCTCCTGCGGGATTTTGGATGTACAGT 80936
Gallid          CAACTTTCCAGTTGAAAAAAGTTACATTCTACGTTTTTAGCACAATCTT-----TCCAGC 86290
                   *  *    **   * *  * * * ** *  * *   **   ** **     * ***

Human           ATCATAAAGTC-CGGAGTAAAGAAATGGGTATAGATGTTTTCGCCCG-CATAGCGTGTAA 80994
Gallid          CCGCCAAAGCAACGGAAAGAGTAGTTGGGCATGAGTGGACTGGTCCAACGGAATGAATGA 86350
                     ****   ****   *  *  **** **   **   * * **  *  *  *  * *

Human           TTTTAGCGCATCTAACTTGGACTGGAAAGATCAGTTCGTTGAGAGAATTAGGCAGCTTAC 81054
Gallid          CATGCGCG-ACCCTTTTCCTACTCTCTATGCCA--TCCCTCAACTCATTTCGAACATCCC 86407
                  * *** * *    *   ***    *   **  **  * *    ***  * *  *  *

Human           AGGAAACGTTGTCGTATTGCATCTGTCGGACTTCCA-CAACCCTGATGTAATAAATTCCG 81113
Gallid          CCGAAATAGACTTTGTCGCCCTTCGATAACTTCTAGCGGTGCAGAACC--TAAATCAGA 86465
                  ****     * * * **    *     ***** * *    * **     *****

Human           TGTGAAGGGTA-CAGGTTTAAGGAATAGACAATCTGCGGTTGGATA-GAGTGATCCAGGT 81171
Gallid          TAAGGTCATTAACAGGGCTCGTGTA---GCACTCTG-AGCTAAATATGAGTAATTACTGT 86521
                *  *     ** ****  *   * *    ** ****  * *  *** **** **    **

Human           TAAATTGCTTGACCGCAGGTACGGTAGAAAGTCTTAAA----ATATCGTTCATTGGCGGC 81227
Gallid          ACAAGACTTTGGCATCACCCGCAGTAGAAAATTTCCCACGTCGCCCTACTAATCGTCCTT 86581
                  **    *** *  **    * ******* * *   *           * ** * *

Human           TCATAGAA--ACGTGTAACGCGTTTTTCATAAT-TGGTT----AACGATTCTGTCGTGTT 81280
Gallid          GCTTAGAACGAGACGTAATAACTTCATCATGGCGTCGTCCAGGAACGCCCCCGTCCGTTC 86641
                 * *****  *   ****    **  ****    * **     ****   * ***   *

Human           GATGGCA-TCAAACAGATGGTACGTGATATCGGATCCGAGTAAGCATTTAGGGACTTGAT 81339
Gallid          GAAAACAGCCATGCAGA-AAAAAATGTCTCTGAATATCTTCGAGAGTTAAAAGTTTTTTT 86700
                **   **  **  ****    *  **     * **       **  ** *  *  **  *

Human           ATATCCGAGTGTCGTTTTTTGTATAAAAGTTAAAAGTTATAAAGCCCGGGCGAACAATTG 81399
Gallid          CCAAAGGAGCGTA--CGAGTGTACAACCATGATCGGATAGAAAGT---GTTTATCCATTA 86755
                  *   *** **       **** **   * *   * ** ****    *   * * ***

Human           GATTTTCAGAGTTTC-CATTTTTACAATGGCTCTCGTTCGATATGGTCCACGGTCTC--C 81456
Gallid          TATATACGAATTCAAACGCGGCTATAAAGGTTGCAGTTGCACATGGTGGAGAACCAAGAC 86815
                 ** * *  * *    *     ** ** ** *   ***  * *****  *    *    *

Human           AACTGTAACAGGGAGTCAACAGAACAAAGACCCA--GAGTCGGAAGAGCATAGTTGTTCT 81514
Gallid          ATTTGGAACATAAAAAAGCATAATCTGCCATCCATAGAGCTGATAAACCGAAATTGCGCC 86875
```

```
                *  ** ****   *        * *    * ***  ***  *  * * *  * ***  *

Human           T-CATCGATGAAGTGTGAAAGGGTTCTGCTATT--CTTTGAAAGTTGAACACTTTTTGAT 81571
Gallid          TGTACATATCTAGCACACGACAAACAAGGCATTGACGATCTAAGGCATAAACATCTGCAG 86935
                *  *   **  **      *       *  ***  *  *  ***    * ** * *  *

Human           CTTTCCATATTAAATAGATTACAAAAATGATGTGTTATGGAACTCGGAATTGTTTCTAAA 81631
Gallid          CAGTCTCTATAAAAGTTACTA-ATTCCGAATGTATAACTTCATTCCCAATATCTCCCGCA 86994
                *  **  *** ***   * ** *      **** * *    * **  ***   * *   *

Human           TTCTCCGCGTCTTTTTTTCTCCGT-CAAATAACATGTCTTTGA-CAGGTTTGCCTGATAT 81689
Gallid          TCCTCCACGGTATCATCACTCTGAACATCCTGTATACTGTTGATCTTGAGCGCGTGGGCG 87054
                * **** **   *  *  *** *  **      **    **** *  *   ** **

Human           TCGGAAAAAAAATTGG---ACAGTTTCACCATTTGAGGATCTATAAG--CAGATCTTATCT 81744
Gallid          GAGAGTAATAACTCGTTAAAAAGCGTGTCATTATAC-ATCAAGACGTCCGGATTAAATGC 87113
                  *   ** ** * *   * *       ****  *  *** * * *  * ***   **

Human           TTGCAAGGAAATTTTGCGCGACTAAATTATTTTTAGGAGATGTGTTTCCGGCTAATTTG 81804
Gallid          CACATAGGGTTCTTTGAAGGACTCCTCTATCCATC--CATCTGCTTCTCCAGAAGATCGG 87171
                     ***    ****   ****    ***    *    *  **  * *** *   **  *

Human           CGATCGGCATCTGTCTC-----GGTGTTTTTCGAGGTGCGTCTAGGGCCACGCATTCCGG 81859
Gallid          ATAAAAGCTGCCAACTCTGTAGGGCGGTTGGCCATGGCCGGTGAAAATACCTCTCGTAGA 87231
                  *   **  *   ***      ** * **  * * *  **    *      * *     *

Human           ATTGTATCGTCTTGCTAAAGAGTGTAGATGCCAAAGAT-GAGTTTGCTTT--TCATTGTT 81916
Gallid          ACGGGTTCCGCACGTTTGGATGAAGTGATTCCAGAATTCGAATTAGTATCGACCGATGTT 87291
                *  *  **  *  * *     *    *** *** *  * ** ** *  *     *  ****

Human           ATTTTTTTGAGTTTAAGACGACTTTGGGGAAGTCGACAATGCAGTCTGTGCATCACAACT 81976
Gallid          AAAATTTTAGCTGAGAAGTATCTTCAGGATGAACGCATATATCGCATATGGTTTGAATAT 87351
                *   ****   *   *     ***  **      **   **   *  * **  *   *  *

Human           GCATTCATCAGGCGCAATATTTGCAAGGTTTGAGAC---AGTTGC---ATCAGTCTATTT 82030
Gallid          TTAAT-ACCAGATGAGATCGATTTAATTTTTCCGACTACAGATGGGAAATTAAACTATTT 87410
                  * * * ***  *  **   *  **  ***  ***    ** **    ** *  ******

Human           CGTTCTTGGATCAATATTTAATTGCAGGCGA--GGTTTTGT-GGAACGTTGTTCCCGTAA 82087
Gallid          -GTCATTTACTAAAAGACTCGCTTCAGCCATTCGGTATGGTCGGATCAAAACTGCAGTGA 87469
                 **  **   * **    *   * *** *    *** * ** *** *     * * ** *

Human           TT-----TGTTTTTTTCAGGCA---GTGGGGACTCAAGTTAGATTT----TTTTAAAAAGT 82135
Gallid          GTAACAATAGCTCCTCAAATTCGTATGGAAGCACCGAGCACGTCTGCGATCATGGGACCT 87529
                 *     *   *  ***        ***   * *      *  * *    *  *   *  *

Human           TTTCG---GGGAAGACCAAGCGGTTGTCATTT-TCGTTTATATGTGATTTGTT---CGCT 82188
Gallid          CTTTATGTGGGCGGAGCGAGAGATTTGCATCTGTAATTAATAGGTTCCTCGATTTACATC 87589
                 **      ***  ** * ** * **  *** * *  ** *** **   * * *   *

Human           AGGTCTCAGGA------TGGCGCAGT---GCAATCTCTTTTATCAATACC-CAATTACAC 82238
Gallid          AAATTTTGAAAGACTGTTAGGACTGTTGGGAAATAAGGTGGGGCAGCATGATTATTATAT 87649
                *  * *    *      * *  * **   * ***    *    **  *     **** *

Human           CAATTTTA---GAAGGGCATGTCAGAAACATACTGAT-TTGTACAGAAGAAGATATCAGA 82294
Gallid          AATTGTCATCGAAAAGGTAAACCATATACAAAGGAGCGCCGCATAGATAAAGATGGAAGT 87709
                 * * * *    ** ** *   ** * *** *        * * ***  *****   **

Human           AGGCTTCAAAGTCAGTCCTCACTA----AGACTTCGGGAGAAAATAGATCAAGGGCATCG 82350
Gallid          CATTCCGAACATAAATAGTCGCCGTGCGGGATTAATAGCGCGCATAAAACTTATCGTCCG 87769
                       **  * * *  ** *       ** *    * *   *** * *        **

Human           CGACAAGTTGCTAAGAATGCGCCTAAAAACCGAATTAGACG---CACTGCAAAGAAAGAT 82407
Gallid          TGGAGATTTAACAACTGGTAATTATGATCCTGTATTAGCAGAGTCATTTTCTGGTTGTAT 87829
                 *   * **   **              *  * * *****  *   ** *     *    **

Human           GCAAAAAGACAGTGACGTTTTGAATTC-TCATTTAAAAGCGATAGAGGATGCTTTGCTGT 82466
Gallid          ACCTACGCGGAGTG-CGTTTCAGTTTAGTGGAGCAGACGGAGTAGAGAGCGCATTCCCAG 87888
```

```
                 *   *    **** *****    **  *     * * *   *****   ** ** *

Human           TT--ACGAATGATGGGGA-GGTAAACGTCGAAACGAAAGCTGATACGCAACTGTTACCGA 82523
Gallid          TGGAATATGTGATGCGTATGATGAATGACTGGGCCAAAGGCGA-ATGTAATCCTTACATT 87947
                *   *    ***** * * * * ** * *    * ****  ** * * **   ****

Human           AATCTCCTGAGAGGCTGGAAAAATTTAATCAAGTTGCCATAACCCCACTCGACCCCTTTA 82583
Gallid          AAGATAC-AAAATACGGGAGTCTCTGTTTTGATAGAGGGTTTTTTGATCCTCCGATAAA 88006
                **  * *  * *  * ***     *   *  *       *        **  **  *  *

Human           TAAGATTTACAGACGATTTTAGAGGAGAAATGATCAATACTTTTTTCAATAATGCTCAGA 82643
Gallid          TGCGACTAAAGCACCGCTGTGTACC-GATAAAGTCAATGTGCTCTTGAATACTACAGAGT 88065
                *  ** * *   **   * *  *   ** *   *****    * ** **** * *  **

Human           TG--TGGAATTTTACGTTCGGGTCTTGGTTTTATAAGCTCAAGAGGGTTTTTTACAATGA 82701
Gallid          CGACCGGCATTGTTTTATCGGATATCA-----ACAAGATTAAACAATCCATCGGTGTAGA 88120
                 *   ** *** *    **** * *       * *** * **        *       **

Human           GCCTGGCTTAAGAAGGGCTTTAAAAATTACTAATGTGGATTCTTTGACTATTTCGAAAGA 82761
Gallid          TTGTCGCCCATTCCAGGCTTGTCTGATTGTGAAC-TGCTTTGTAAGATTGCCT--ATTGT 88177
                   * **  *     *****     ***   **  **  ** *  ** *   *  *  *

Human           GTTATTAACAGTTACCGTCAATGCGTTGGAGCAAGCGACGGTGTATCCTATATTCGGAAG 82821
Gallid          GCAATTGGCCTTCA-----GATTTGTCGGACCTAGTGA-------TCCTGGCCGAACAAC 88225
                *  ***  *  * *      **  ** *** * ** **       ****        **

Human           TGAGATGTCAGACCTGGAAGCAGCCTTGTGTATCCTGGCAGCTTTTTATTCCACTTATGA 82881
Gallid          TAAGCTCCTTGATTCGGCAATAGCAGCATATGATGCAAAAATTAGACAACGCAGGAAAAG 88285
                * ** *    **   ** *  ***    * *        *  *    *   **   *

Human           GAATTCACAGATCGACGAACGTACAACCCTAG----TAGATATTATA-----ACCCTTCT 82932
Gallid          AAATCTACAAATTATGGAAAGTAAATCGCAAGATCATACATCCGATGGATGCATCCCAAT 88345
                 ***  *** **    *** *** * * * **    ** **   **      * **   *

Human           GCCCGTGATATTTAGATTACTCGGTAGTGAAATTAC---AGCATTGAAAAATGTCTCTCC 82989
Gallid          ACCGATAATCGATGAAACGCACAGAAGTGGCATAACCGGAGAATCTAAAAGTCTTTTTGG 88405
                 **  * **   *  *   * * * ****  ** **   ** **  **** * * * *

Human           T---TCTGGCACCTATTTTGG--------ATTTAATGATCCTTCTTGTATGAAATTTTTT 83038
Gallid          TATGTTTAAAAACATTGCTAGTGGCGAATGTGTGACGACGATACGTATACCGCGCTACAT 88465
                *   * *   * *  *  * *         * * * **   * * * **      *   *

Human           GTTCCCA-TGAGAAAAGGCAA-GCATTAT--GCAGAAAACACATTTGGGAATCATGTGCT 83094
Gallid          AGTCATGCTGTGGATATTTAGTGTACTACTAGCAATGGTCACATGGGGCAGCTAT-CGTT 88524
                  **    ** * * *   *  * * **   ***     *****  ** *   **  * *

Human           TGTAAAGATGTTGCTAGGTAGAGGAGTGATGCAAA----AAATTCCCGGTGAGAA-AGAC 83149
Gallid          TGTACTCTTAA-ACTTCATGAAACAATGATACAGTTCCCAGTCCTACGATAACATCAGAG 88583
                ****    *    **   *  *  * **** **      *      ** * * *  ***

Human           AGCCAAAATTTTGATGTTGAAGCTCGCTTACACGGAGCCATTAAGAATGATGTTCTGGTG 83209
Gallid          AGACATCATCGAAAGAATTGTCCTTATCAGCTTCAATGTCTCGTGCATCATCCCAACAGG 88643
                ** **  **    *   *    **      *    *    *   * ** **        *

Human           TATTGGACGTATC---AGCTAATGAGACCA--AAATTGGGAAATAATGTTCCAATCTTTA 83264
Gallid          CATCGCCTACCTCGCAAACCTTTGATCCCTCTGATTTGAGCACTTATACATTAGATGTGC 88703
                 ** *      **   * *   ***  **    * *** * * * **     *    *

Human           TACACGATCAACATTATTTACGGTCTGGCTTGGTTGCAATTGAAAGTCTCTTTTTATTGT 83324
Gallid          TACATCATTACCCT--TTAGTGGACCTGCTCCA--GCAACTCAATGCCATACCGAATGAT 88759
                ****  ** * * *  **   ** *  ***     **** * ** * *       **  *

Human           GGCGGA-TCTTGAACTCAGAAAGTTTGTTTAACAAGAGAGTTGGGAAGTTTCTCCTGACT 83383
Gallid          CTCAAAATTCCGGATACTTATTCTAAAGTAGAACAAGCAGCAAGAATCAATGTTCTGCGT 88819
                  *  * *   * *  *  *   *    *  *  *   **   * *    * * ***  *

Human           TCGATTTTTCCACAGCTGGAGAATGTTGATTTCGCCGAAAATAACTTTGAAGCTGGGAAT 83443
Gallid          GCAATTTGTGTTGGATTTGCTGAAGT--ACGCCGACATAACGATAATCGGACCTTGCAAC 88877
```

```
                 * **** *       * *   * **  *   ** *  **  *   * * * ** * **

Human           ATTCAAAATTTTGAATACTTAATG-CACCATTACGT---TGTGCCTATGTACAACCTGCA 83499
Gallid          GTTCGACCATGTTTGCAGTCAACGATGCCGCTTCGCGGATACGACCATCCATTGGCTTGA 88937
                 *** *   * *    * * ** *   **  * **    *  * * **  *    **  *

Human           GAATGACATATCAA----------TTTCGACTCTTTTTCCCGGTCTCGTGGCCGTGTGTG 83549
Gallid          AACGCACATTCCCAACGGGTATCTTTTCGACTACTATACATGGA-TCGCCGTCAGATGAT 88996
                 *   ****  * *          ********  * * *  **  ***  * *   **

Human           TAAATGAGAGTGTGAGATTGGGATGGGAACATAA-GTGTGCAGGGGCTC------CGTCC 83602
Gallid          GATATATCGACGTCATAATAAAGTACAACAATAAAGTGTTTAAACAGTATACCGACACCT 89056
                 * **      ** * * *    *   *  **** ****  *     *       *  *

Human           GATGCCGTTCAGGTTCA---ATCTAAAGAAAATCCGTTCG---TGGAGTATATCCGCGCA 83656
Gallid          AATTCCATTCTTTTTTATTTATCCAGTCAGGATCATTTTAATTTTGAAAAGCTCTATTGC 89116
                 ** ** ***   ** *   *** *   *  ***  **     * **  *  **

Human           CAAA--TGGAACAACAGG---CGGATGTGGCTATACTTGAAAAGCATGACT-GC-ATTCT 83709
Gallid          CAATGTTGCAACTTTAGTTCTCAGTTGTTCCCTGACATATACAATTTGTCTCGCGACTTT 89176
                ***   ** ***   **    * * ***  *   ** *  * *   ** ** ** * * *

Human           CTTTCATTTTGAAAACGGTTTAAACATTACTCTATCTTTTACATTACCGAGACAGAGATT 83769
Gallid          TTTGCATGCTTTTGTAAGCGCATACAATGCACCTCTACTGGCATTCCTGAAATCTCGTCT 89236
                 ** ***  *       *   * *** * * *      *  **** * ** *    *  *

Human           GTTTGCAATGGCCTCTTCATTATTTAATGTAAATGATACTTATGATTTCATCTATTTTCT 83829
Gallid          AGTCAGTAATGCATCTGCAGGGATCACACGAGATGTACTGGATCCATCTGTAGTCGTATC 89296
                  *    *  ** *** **    * *    * ***      **   *   *     *

Human           AGTTTTA--GGTTTTCTGCCAATACCGGCCATTATATAAGGCGGGATGTTGTTGTGTTGA 83887
Gallid          GGTACTACCAGAGACGTGCAAATGCGAATCGTC---CGAATCGGAATCTAATGATGTCAA 89353
                 **  **   *     *** *** *    * *      *  *** ** *  *  ***  *

Human           TTACGTCAAT----ACGGATGGGGTTTTGA-ATATAAAGACTTACAGAGCT-----TTTA 83937
Gallid          TGATTCGGATTCAGACGAATGGCGCATCGGTATGTTATTGTATCCAGGGATGGAATTCTC 89413
                * *     **    *** **** *  * *  ** * *     * *** * *     * *

Human           ATGAATCATTGCTTCGTTTCTTCGG--GATGGAGAAAGAAACAAAGTCTTTGGCTTGGCC 83995
Gallid          TTTGGTGTCTGTTTCGGTTATAATGCTACTTATGTGCATATCAGAATGTTTGTCATTAGG 89473
                 *   *   ** **** ** *   *    *   *     * ** * * **** * *

Human           AGCCA---CCGCGGAGTTTTATGGCTGGGTATTCATTT-TTTCGTCCATCCAGTTGT-GT 84050
Gallid          TGCTAATCCTGTAGGGGTCTGATGATGCGTGTCATCATGTTTCGGGTATGCGACAGTTGC 89533
                 ** *   * *  * * * *   * ** ** *     * *****   ** *    ** *

Human           ACGGTGGTTTTCCTGACGGTTAGATTTAATGGTTTCAAAGTCGGTCGAGAATACGCTGTG 84110
Gallid          CTGTTCAGATGAAAGATATTTCAAGTCAATAGTTTTTGGTGACGTAGAACATGCCCCTTG 89593
                  * *    *    **   **  * * *** ****        ** **  ** * *  **

Human           TTTACAT--TTGCCGGGATGAGCTTCAATTGTTTTTTACTTCCGATAAAGATGGGATTGC 84168
Gallid          AATACATGGTGGCCAAAAAGAAGAATAATCATACAA----TCTGCCACGTATAGTACCCG 89649
                  *****  * ***   * **     ***  *        ** *  *   ** * *

Human           TGAGTGGACATTGGACTTTGCCGCGAGATTTT-TGCGCAATTCTTCTCTACATCGATGAT 84227
Gallid          CGTCCATGTTTGCTTTTTTATCACCGGATATTGTATGTGCCTTTAGTCGCTGTAGTAGAG 89709
                 *        *     ***  * *  *** ** *  *    * *  **    * *  **

Human           TTTTCTGCCTATTTCTCTTCGTGGTCGTTGGTGTTTA-----TGGCGATTGAGC-GCATC 84281
Gallid          GAGACGGTTCATTGGTTGGAGATGCCGTTCCTACCAAAATATCGGTGATTGATCTAGATG 89769
                    * *   ***  *    *  * ****  *    *      ** ****** *   **

Human           AACTACTTCTGTTACAGCACACCGTTGTTGAACGAGAATTCCAAGGCTCTGGCAAAGGTG 84341
Gallid          AATTATTCTTACGACAGTGCAATCTAGTCGTGGGCGA--------GCCTTGTCTAAGTAA 89821
                ** ** *  *   ****  **   * ** *   * **        **  ** * ***

Human           TGTTTCCCGATAGTTTGGGTCGTAT--CGGGAGTGCAAGCTCTTCAGATGCTGAATAATT 84399
Gallid          TCCAGTTCCATTTTTAAACCTATATTTCTTCAAATCTATCCCTCCTATTCCACCATAGTC 89881
```

```
                *      * ** **       ***  *   *   * * * ** *   * *   *** *

Human           ACAAAGCCACAGCTTTGCAGAACGAGACTGGACAGTGTTTTCTCGCGTTTTTAAGGAG-- 84457
Gallid          ATTGTTTGTAATAAGCGATGAAGAGGCATTGGCAATGACTGTTGGCGTAGACATCCAATC 89941
                *         *     *  ***    *  * * ** **  *  * ****     *   *

Human           CGGACACGACATGTGGTTAATGTTGGTTTATTCGGTTGTGATTCCAG--TCATGCTGGTG 84515
Gallid          CAAATCCTTCCTATGCTCACTATACGATTCCCTGGGAATGGTTTCTGACTCATGTCGATA 90001
                *  *  *  * * ** * * * *  * **    **   ** ** * *  *****  * *

Human           TTTTT--TTACCTATACAGCAAAAATTTCATGCTTTTGAAAGATGAACTTTCGTCCGTGA 84573
Gallid          TCCTTCCTTGTCTATTAAATTATCAAAA-GTGACTATACCGGGAGAATATTGATTAA--G 90058
                *  **  **  ****  *   *  *     **  * *    *  ***  **  *

Human           CTACGTATCTCTGTATTTATTTGCTGCTGGGTACAATAGCGCATCTGCCGAAAGCTGCCT 84633
Gallid          TTCCATATCATGCCGAGAGGAGGCTGCTG----CAGTACCAGTCCAATTAAAAGTCGT-- 90112
                 * * ****             *******    ** ** *     *     ****  *

Human           TAAGTGAAATTGAGAGTGACAAAATTTTTATGGTCTGCGCGATATTTTTATGGCGCTTC 84693
Gallid          --ATTGTAAACAGCAGTATGAGTATTATGCATGTGCTTTCTAGATTTTTCATTTCTATAT 90170
                  * ** **      ***   *  *** *  ***  **         **** **  *  *

Human           CTGTTCTAAAAGTTTATTATATATCCGCTATGGCCTATTGCATGGCATGTGATGATCACA 84753
Gallid          CTACTTTGAGAGCCTTTTCTCTTATCATCAGTTTTCAGTGTCGTATAACCGTTCTTTACA 90230
                **  * * * **  * ** * *   *   *      * **      *   * *  * ***

Human           CCGTGCCCGTTCGTTTGTGCAGTATCTGGCTGGTCAATTTATGTAAGAAATGTTTTTCGT 84813
Gallid          CTAT-CCGGTTTAGTTATTAAAGGTATAGCAGGCAGATTCGATTGACACGG---TTTGGT 90286
                *  * ** ***   ** *  *   * * ** **   ***    * * *      *** **

Human           GTACGCGGCGCGAAAAGGGGTCGGATTTAGAGGTTGGAA-TAAAAATGTTAAAATGAGTA 84872
Gallid          TTCCTCGGCAATAGAGGAGGTTGGA--GGGATCTTGGAAGCCGAAAAAGTATTAGGATTG 90344
                 * * ****   * * * *** ***    **  ******    ***   **  * ** *

Human           AGGAGTTTATCGTTTTT--ATTCATAAGAGAATAAATCTTCGGCGCTGATTCCTGTAAGG 84930
Gallid          GGGACCGGAGAGGTTTTGGAATCGGAATTAGGAAAAGGTACTG-GAAGAGGTTTGGAAGC 90403
                 ***    *  * ****  * **  **      ***  * * * *  **    ** ***

Human           TCAAATATGGCAGAACTGTCGAGAAACTGTGGGTTTTCAGACATGCCGCAGAAGACTTTA 84990
Gallid          AGAAGGAGACTTGGGCTTGGGAGCCGGAGAGGGCTTGGAATCGGGAGTCGGCGGAGGTTT 90463
                  **  *     *  **   ***     * *** **  *  *  *   * *  **  **

Human           AAGTCGTTTTGGAGAAATGAAAACAGTCCGGCTTTGTGCTCGGCTCTTTGCTGATTGTAC 85050
Gallid          GGG-----CTTGGGAGCCGGAGAGGGCTTGGAATCGGG---AGCCGGCGGAGGTTTGGGC 90515
                  *      * * **   * * *  *   **  * * *    **     *  * ***  *

Human           GCCAGCGTGGATATCTCAAATTTTCCAAACATTATAAGGTAGGCGAACCATAGGTGTTTG 85110
Gallid          TTGGAAGCCGGAG----AGGGCTTGGAAGCCGGAGAGGGCTTGGGGGCCGGAGTGGGCTT 90571
                      *  *       *    **  ** *   * * **   * *  **  **  *  *

Human           CAGATAAGCCGTACTGCTGGTTCTCGTTCTTGATCCCCCCAAAATCGGTTAAAGAATCTG 85170
Gallid          GAAATCGGG-ATCCGGCGGAGGTTTGGGCTTGGGGGCCGGAGAG--GGCTTGGGGGCC-G 90627
                 * **  *   * * ** *    * *  ****    **  * *   ** *   *   * *

Human           CAGATTTTTAAAAGTCGGTGATTATAGATAGCTCCAACGGAGAAAACTATATTGCCTTCG 85230
Gallid          GAGAGGGCTTGAAATCGG-GATCCGGCGGAGGTTTGGGCTTGGGGGCCGGAGTGGGCTTG 90686
                 ***    *  ** **** ***       ** *        *    *   * **   * *

Human           CAGATTATTTCTTCCGGAATTTTGAGACTT-AGACCAAACTCGTCCATGAAGAGAAAATT 85289
Gallid          AAATCGGGATCCGGCGGAGGTTTGGGCTTGGAGGCCGGAGAAGGCT-TGGGGGCCGGAGT 90745
                 *        **   ****  **** *  *  ** **  *   * *  **  *      * *

Human           AATTATGTGACTGACATTTCCGCTGTAGTGCAGCTTTTTTGAAATATATGCTGAGTGACT 85349
Gallid          GGGCTTGAAATCGGGATC-CGGCGGAGGTTTGGGCTTGGGGGC-------CGGAGAGGGC 90797
                     **  *  *  **  * ** *  **   *  **   *          * *** *

Human           TTCGTTAAACATATGTCGGAGTAGTTCCGC--GTCAGAATGCTGTTTCAGAATAGAAAAT 85407
Gallid          TTGG--------GGGCCGGAGTGGGCTTGGAAGCCGGAGAGGGCTTGGGGGCCGGAGAGG 90849
```

```
                 ** *          * ****** *    *   * * **  *   **   *    ** *

Human            AGTCGAAACCAGTAGTTGTTGCTGATTTTGCGATTCAAAATCACTATGTCTCCGATTCCA 85467
Gallid           GCTTGAAATCGGGATCCGGCGGAGGTTT-GGGCTTGGGGGCCGGAGTGGGCTTGG----G 90904
                   * **** * * *   *  *  * *** * * **      *    **     *

Human            GAATTGTATAAACTCTCGCGTAGTAGTAAGATCTTGATTCCGTGTGACAGCATTGATGTA 85527
Gallid           GGCCGGAGTGGGCTTGGGGGCCGGAGTGGGCTTGGGGGCCGGAGTGG--GCTTGGATGAA 90962
                 *    *  *   **   * *  * ***  * *   *   * * ***   ** * **** *

Human            CAGACATGTTGAATTAAAAGAAGTTCTTCTTGTTTAAAGCTAGTCAGATTCTCGTTTCTA 85587
Gallid           C-------TTTCACTGGTCGGA-----TCATGATCACTGGGAAACAAATTTGAATGTTTG 91010
                 *       **  * *    * *     ** ** * *  *  *  ** ***    * * *

Human            AGCAGTTTTGTGGTTATGTGTAATATTAAATTAG--GGACGGCG-TCGTTCAGTTTTATG 85644
Gallid           GGGAATGGAGGGGGATTCGATGAGGTTTCATCAATCGGATCACGATAGTTGGGAGATATA 91070
                  * * *   * **   *   * *  **  ** *   ***    ** * ***  *   ***

Human            TAC---TGTCCTATCTG-GGTCATCTCAAGATGTCTAAAGTTTGG--GTAGGTGGATTCC 85698
Gallid           TCCAAATGTCCTATCTGTGGATAATTCATGTTTTCGGAAACATGGCGATCGTTGTGATGA 91130
                 * *   *********** **  *  *** * * **  **   ***   * * **   *

Human            TCTGTGTCTATGGTGAAGAACCGTCGGAAGAATGTTTAGCTCTGCCCAGAGAC-ACGGTT 85757
Gallid           ATTATGTCTCCAGGCAAAAGTAATTGTACTTGTGCACCTTCCATATCATTTTCTACCACT 91190
                   * *****   *  **  *    * * *    **       *    **    * **   *

Human            CAAAAAGAATTGCGGTCCGGAAATATTCCTTTGCCGTTGAATATTAA-TCACAACGAAAA 85816
Gallid           CGATCCCACATGCTTTCAGGATCTATAC--------TTAAGTATCCGCTTCCCATAGAAT 91242
                 * *    *  ***  ** ***  *** *        ** * ***    *  * *   **

Human            GGCCACTATTGGAATGGTACGTGGTCTTTTCGATTTAGAGCATGGGCTTTTCTGCGTTGC 85876
Gallid           CTGCATAGGTATCATCTTCCGAGTCTATTCTGTTAGATGATATATCCGCGTCGCCATCCC 91302
                    **    *   **  * ** *    **  * *  *    **    *   **  * *  *

Human            GCAGATACAGTCTCAAACGTTCATGGACATCATAAGAAATATTGCTAGTAAGTCAAAGTT 85936
Gallid           G-AGAAAGATCGGGAGACACACATTTATTGTATATAGGACATGGATAT--ATTCTGCTTT 91359
                 * *** * *     * **   ***  *    ***    * ** * **   * **    **

Human            GATCGCAGCAGGTTCGGTAATTGAACCTTTACCACCGGACCCGGAAATTGAGTGTTTA-- 85994
Gallid           CGTCAGAAAAGAATT--TAGACGCTTCTTGTACATCGGCAATGACCATGTGTTCATTGGC 91417
                   **  *  **  *   **   *   ***   ** ***    *   **    *  **

Human            -AGTTCAAGTTTTCCTGGTCTATCGCTTTCTAGCAAAGTTTTACAGGATGAAAATTTAG- 86052
Gallid           CAGTTCCGGTTCTTTTCGTATACCCGCATCGACATAAGGCAATTCTGATATAAGTGCATC 91477
                  *****  *** *  * ** ** *    ** *   ***        ***  ** *  *

Human            -------ACGGTAAACCGTTTTTTCATCATGTGTCTGTATGTGGAGTCGG-TCGGAGACC 86104
Gallid           ATCCTCCGAAATTAGCCGTATTATAGGAATGGGGTCCAATATTTCCTTGGACCATAGAAC 91537
                            * * **** ** *    *** *     ** *    * **  *  *** *

Human            GGGAACTATAGCGATTTTCG----GACGAGAAATAAGCT--GGATTCTAGAT-----AGG 86153
Gallid           TTTATGGGAGCCATTCCTGAATCGACTATTAATAAACATGGGGTGCGGAAGCCAAAGG 91597
                     *    *** ***   *    *** *  ***** *   **  *   **      ***

Human            TTTTCATGCATCAGCGAGAGTGAAAAGAGACAAGTTTTGGAGGGGGTCAATGTTTATTCT 86213
Gallid           TTTGGATGCAACAATGCGGGACAAATGTTCCAACTGCTGGCCAAGGCAAGCGTTTTCGAT 91657
                 ***  ***** **  * * *  *** *   *** *  ***    **  *  ****    *

Human            CAGGGTTTCGATGAAAATTTATTTTCGGCGGATTTATATGACTTGCTCGCGGATAGTTTG 86273
Gallid           CGGATTGTTATCAGTTATCAATAACT--TAGTTCCCCAAGATGTCATATCAGATGAAACG 91715
                 * *  * *        **  **        * *    *  ** *  *  * ***     *

Human            GATACATCCTATATTAGAAAACGGTTTCCAAAGTTGCAGTTGGA--TAAACAGCTATGTG 86331
Gallid           G---CGGTTGGTTCCAAAAAACATCCCCTAGAACCATGTCTGGAATCAAATAGGCGAATG 91772
                 *   *      *   * *****     * * *        ****   *** **     **

Human            GTTTGTCTAAATGTACGT----ATATTAAAGCTAGTGAACCACCGGTGGAGATTATTGTA 86387
Gallid           CATAGACTTAACCCAGATTCTGACGAGTAGGCTTCCGGACATTCTGCTGCGATTACAATA 91832
```

```
                  * * ** **   *  *    *     * ***   * **   * *  * *****   **

Human             ---GCAACGGGCAAAGTTGCCGGTGAT--CAAGTTCA----GTTAACTACGGAACCTGGG 86438
Gallid            CGTGCCCCAAATAAAGTCGCCGTTGTTGCTATATCCATCGCATTCACTCTTAAAACGCGG 91892
                     **  *    ***** **** ** *   *  * **     ** ***    ** *  **

Human             TCTGAATTAGCGGTTGAAAC-GTGTGACGTGTCGGTTGTGCACGGGAATTACGACGCCGT 86497
Gallid            CTTGGGGGCGTGGATACTACTGTAAAAGTAGTTACATTTCCCGTGAGGTCATATAAATTC 91952
                    **     * ** *   ** **   *   **    * * *   *   * *

Human             TGAATCTGCGACAGCTACAACGGCTATGAGTAATCAAAATCTGCCAAATACTACCCCTTT 86557
Gallid            GGCTATCCGTGATCGATGATATATTTTTATTTCGGAGCATGATAGAAGTGATGTTGTC 92012
                   *  * * **  * * *  *    * *   *  **  *   **  * *    *     *

Human             GCTGTCAAGTCCACCGTTTTCGGATTGTGTTTTTTTACCGAAAGATGCTTTTTTTTCTCT 86617
Gallid            ACATCTAAACTTGATGCAAGATCACCGCGGGCCGTCACTACATCATCATAGCTCTTGCAA 92072
                   *   **      *       *  * *     * **   *  **  *   * **

Human             TTTAAATGTTA-CAACTGGACAGCAGCCGAAAGTA-GTTCCACCTGTTTCTGTTCATCCG 86675
Gallid            TTCTGTTCTAATCCGTCCGGTAGCAATGAAGACCATATTACAGCTAATGCTGT-CGGCGG 92131
                  **    * * * *     *  ****    * *  *  ** ** **  * **** *  * *

Human             CCCGTGACTGAACAGTATCAAATGCTACCGTATTCGGAGTCGGCTGCTAAGATTGCAGAA 86735
Gallid            TATGCACATACGCGCCAATAC-CGTTGTTGTTGTCAAAAG-GCTTGATGGGATAGCCG-- 92187
                    *    *   *   *  *   * *   **  **  *   *  ** *  *** ** *

Human             CAGGAGTCGAATCGATATCACAGTC--CTTGTCAGGCAATGTATCCCTATTGGCAATATT 86793
Gallid            CGCGAATTTTAGCAAGGCCATCGGCTGCATCTCCATCCAGGTCTTCCCAT-AGTTTAGA 92246
                  *  ** *   * * *   **  * *  * * **   * * ** * ** **  *   **

Human             CCCCCGTTCCCCAGTATCCGGCCGTGTTAC--ACGGTTATCGTCAACCGAAAACGTTTAA 86851
Gallid            GGATCGGTATCGGACAATTTACCTTGTCTCCAATCGGAATAACGTACTGAAAATAAGGAG 92306
                      ** *  *    *     ** ***  *  *  *  **     ** *****     *

Human             AAA---GCGGCATTTCCAGAGTGATTCGGAAGATGAATTAAGTTTCCCGGGAGATCCGGA 86908
Gallid            CCGCCTGCAGGATCCCATACACTACCCGTTAAACTTAATGGTCTACAATTCGGGTCTGGA 92366
                        ** * **  *       *  **  * *   * *    * *      * ** ***

Human             ATAC-ACAAAAAAAAGGAGGCGCCATAAAGTTGACAACGACGACGATAAGGAGATGGCTC 86967
Gallid            ATATTATCTTTGGATAGTTGTACTGATAAGCAATTCACAA--ACGCTACTGTGG-ATATC 92423
                  ***  *       *  *  *  *    ***      ** *  *** **  * *     **

Human             GAGAAAAGAACGATTTAAGAGA-ATTGGTGGATATGATAGGAATGTTAAGACAAGAGATT 87026
Gallid            GAGTCTGTCATAACTTCATAAATAGTCTTGTCCATAA-AATCATATTGATCGATTAGATC 92482
                  ***      *  * ** * * * * * *  **   ** * *   ** ** *   *  ****

Human             AATGCTTTGAAGCACGTTCGCGCTCAATCGCCGCAGAGACATGTCGTTCCGATGGAGACT 87086
Gallid            GA-GCTTTGAAA-AGGTTAACAT---GTGTCCGGGTATATTTGCCATAATGGTGACCATT 92537
                   * ********  * ***  *      *  ***   * *  ** * *   * **   * *

Human             CTACCTAC--GATCGAGGAGAAAGGCGCCGCGTCCCCAAAGCCGTCTATTTTAAACGCTT 87144
Gallid            ACATCGGCTGGATCCAATCGAACAAC-CTGC-TTCCCTCGTCCGTTCGC--CGCATTTTT 92593
                    * *  *  **** *   ***   * * ** * ***    ****          *   **

Human             CTTTGACGCCTGAAACGGTAAACAGGAGCCTTGCTGGTCAGAACGAATCCATGGATCTGC 87204
Gallid            TTTTAATTCTTTTTTTATCTACTATGGGAGTTCTCCGCCCGGGAATAAAATATATGTCTGA 92653
                   *** *  * *       **    **     *  * *  * * *  **   **   ****

Human             TAAAACTCAACAAGAAATTGTTTGTTGACGC-GTTAAATAAAATGGATAGC-TAAAATGT 87262
Gallid            TAAATTACT-CCTGTTTTTCCGCGTTAATGTTATTGCGATTAATGCACAATATATAGTAC 92712
                  ****   *  *  *   **     *** * *   **      **** * *   ** * *

Human             GTTTTTTTATGTTGTGATCAAGTGGTGTTTAGGTG---CGTGTTATGGGGAAGATCTATG 87319
Gallid            CTAGTGCTTTAATGTAATCACTCTGCATCCAATTATGTCGTTTTGTGCGAACATACTTCG 92772
                   *  *  * *  *** ****    *  *  *  *    *** ** ** * *    **  *

Human             ---AGAATTATGACTGACAAGGTATATGGCGAATGCC-CACATTTCCTTCAAAGTGA-AT 87374
Gallid            TAAAAGAATAGAGATGGTTAGCCATATCATGAACAAAACCCAATCTGAAAGTCATGATAT 92832
```

```
                   *  * **    **   **  ****   ***     * ** *          *** **

Human              TCCTGGACATG---GTGAAACTTAACACATAATTGTTTACCG-ACGCGTTAAATAAAACG 87430
Gallid             CGCTTAACATGCCTGCAGCGGCTTCCGTTTCTTCGGTTGTTATAGGCTGTATAGGGATTT 92892
                     **  *****   *       *  *   *  * * **     * **  ** *   *

Human              GATGGTTAAAAGTTGTTGTTTTGGTTTTTATTTTCGTATAAAATGGTATTTAGGAGCTA 87490
Gallid             GAGGTTTTATCCCTATGGCGGCAGGTTCGCACAAACAAGCAGCTAATTTTTCCCCAACCG 92952
                   ** * ** *    * * *     * **   *    *    *      * **     * *

Human              GTT----ACGGGGAAGAT---CTATGGGAGTAATTATTGTGACAAGGTATGAAACGAATG 87543
Gallid             TTCGAAAACAGAGAGAATACCTCAAGGGTTTATCTGTTGAATCTAGATATGTTAGGCATC 93012
                    *     ** * **   **     * ***  **  * ***   * ** ****  * * **

Human              CCGA--CGTCTCCTTCTAATATCGTTAGGCTTTGTGGGGTTTCAGGCGGCATGAATTCCA 87601
Gallid             CAGGACTATCTATTTCAGAAAAAGCT---TCCTGCAGTGTAGCTACGACTTTTGGTCTGA 93069
                   * *     *** ***   * *  * *      **  * **  *        *   *   *

Human              TGCGAGTAGATTCTCCGGGTTTTAGAAAAAAGTGCAAGGGTACCAAATGTTCTTTGTCGA 87661
Gallid             TCCAACTTGCAACTCTATGTTTTAAGTATCTATTTACGACTCCCACGTTTGATGTGTGAT 93129
                   * * * * *   ***   ******   *    *  * *  * ***  * *  * ***

Human              ACGCGATGCGGAGTG-ATTTCA------AAAAAATGCCCATGCTG-ATCTCTGGTGTTAC 87713
Gallid             TCGCTCCATTGTTTGCATCTCCTGCATCAAAGGGCAGCAACATCGGATCGCCGACATTCC 93189
                    ***      *  ** ** **       ***      * *    * *** * *   ** *

Human              GTGCGCGACCGTGGTTAGACTTGGTGCTCGCGATACTGG-CTGGCTTGCGAGAGCTACAA 87772
Gallid             C--CTCAAATGTGTTCGAATATCCAGCATATAAAGCCAGTCTTAATGTTAACAACCGCGA 93247
                      * * *  *** *   *  *   **      *  *  * **   *    * * *  * *

Human              TGTCTTTTACGGGACCATTTGGAGATTCTATGTTTACATAAATGTTGCTCATTTCTGTCC 87832
Gallid             -ATATCTTGCTACAC-GCTTGGTG-TACCAT-TTTGGA-AGACGTTTAGCGATATCCTCA 93302
                     * * ** *   **   **** * * * ** ***  * * * ***   *  *    **

Human              AGCTGAATTCGATATTATTCCCCGGTGGTAAAATTTGCTTGGGGAAGAAGAATACTTTGC 87892
Gallid             AACTGTGT---ATAT-ATTTCTTAAGATCATTTATGGCAAAGGTTGACGGGATCTTCTC 93358
                   * ***  *   **** ***  *    * * *  * **     **  ** *    ***  *

Human              CTA-GTTCTTGCGAGTAATTGATGCGACAGTTCTTTTTCTGTGCTT----TCATGGTGAT 87947
Gallid             ACACAAACCCTCCATTTGTTTTTGGGTGGCCTCTATTTCCTTGACTGTGGTAAGGAAGGC 93418
                     *    *   * * *  **  ** *     *** ****  **  *    * * *  *

Human              GTAGAGA-----GGCCGCGTCTCG-TCCCAAATGCAGGTGGTTATAGAGATTCCGTGCAA 88001
Gallid             GTCAAGAACGACGACCCTGTCTCGGTACTTGGTATCAATCAACCCTATTATTTTAGCCGG 93478
                   **  ***     * **   ****** * *    *     *        ***     *

Human              GAGTTTGGGGA----TAAAGAAACCCGTAAAGTTTGAATTTGATTCGAAGATAGTGTGGA 88057
Gallid             CAGTCTGGGATATTCCGCATTACTACGCAAAGCATTTATCATATTCACAGATACTTTTGT 93538
                    *** ****         *  *   ** ****  *  **   ****  ***** * * *

Human              AAGAGCAATGGAAAGGCGTAATCAGGTTAGAGA--CGATATTTTTAGTTGCGCCGACAGT 88115
Gallid             CCGAGCAGCACTTTCTCGAGCTGAAGAAAATCCTTCGGAACTCTTA-TCAATTCTATCTT 93597
                     *****         **   * * *  *      **  * * *** *     * *   *

Human              TAT--TGAACCGACGTTGCATTTTAGAAGT-----GTTTCTTGATTCTGCGACA-TTGTG 88167
Gallid             TATCATGTACAAATGTGGCCCATGTATCATCCCAAGCAACTTCAGCCAGCGCTAGTTCAG 93657
                   ***  ** **  * ** **   *      *     *   *** *  * ***  * **   *

Human              ATGTCTGCGGTGTGGGGGGTCAGTGAAAGTTGTATGTTAAGTTTTTCGGGAAAAAAAGTA 88227
Gallid             ATTTTAACTCTTCCAGGCGTTTGATAAATTAACTAATGCATCTATCCGGTCTGCATATA 93717
                   ** *   *  *    ** **      ** **   *  *   * * ** **     *  **

Human              TCATCAAGGAGATAGTCGTTTTCGTACACTGTCTTCAAAATTAATAGATTTCCACAAAGT 88287
Gallid             TTGACATAGGACCACT--TTCATCTATCCTAACAGTCAAATCATGAGAAT-CTATAACT- 93773
                   *   ** *    * *  **     **  **  *    **** *  *** * * * **

Human              AAGATTCTAGAGATGGAGACGTGTCGGTGTGATATAAATTCAATTTAGCAGAGTTAAAA 88347
Gallid             --GATTCGGCATGTCTCAACCATTCAACAGTTTGCGAGTCCAACTCTTTAGTTTCTTCGA 93831
```

```
                  *****   *  *    **   **       *   * * *** * *        *   *

Human           ATTTGGTCGAGCGGAGTGTT--TTGGAGATTGAT-CCATATATCTGCGTTAAATAAATA- 88403
Gallid          CTTTAGTTAAAAGCAGTGCTGCTTGAGTGACGATATCATTTACGGTCTCTGACCTATCAA 93891
                 *** **  *  * **** *  ***       ***  *** **    *  * *   *  *

Human           TCGTGTTGGCTCTTC----TGGTGTTTTTTCTGTCCATCGTATATCAGCCGCGTAAATTT 88459
Gallid          TAGCCTTGGCTATATGCGATGGCATCGAGACCAGTCTCAATAGGTTTTTAAGATTAGCTA 93951
                * *  ****** *      ***  *      *    *    **  *         * *  *

Human           TAATGCGCGTGAGAGGGCCATATAGAATAAACTGCGCCTCGCACTGTCCGTGGGGTATGG 88519
Gallid          AAAGTTCCGCAGACTCTCTGTCTGCAGCATAAGATGCTTCGGCCAAATTCTTCAGAATAG 94011
                 **    **         *  * *  *  * *    ** ***  *       *   * ** *

Human           CGTGGCGATCGTAGGCTTCGATGCGTTGCACCGGTAA--ATTTGATAAA---GTCACGTT 88574
Gallid          CTTCTCC-TCTCGTCTTTGCATCTTCAATAGCAGTGGTTACTCGATGTATTAGTGAGGTC 94070
                * *  *  **      **  **        * * **    * * ***  *   ** * **

Human           CGCGTAAGGGAGTGCGAATATGTTAAGAACGATGGGTAATCTGCCGAGATC--CAGATCT 88632
Gallid          ATTGCATCAGTATCCCGTCGGAGTAAATGAGCACCATATTCTGAATATGCTGTCCCCTCA 94130
                   * *   *  * *         ***     *     ** ****   *       *   **

Human           AGATCTGAGATGTTATGCACCGCAAAGGGTATGTTGCCGTAATCTTTTGGATCGATGTAA 88692
Gallid          AGCTCTTCTACGTGATCTTTTTTCAAT--TCTTCTGCTATGGACATTATAGTGTTTACAG 94188
                ** ***   * ** **         **   * *  ***  *   * **    *   *  *

Human           GTAAAAGGCGATGCTGCAAAACTGCTTTCGT----CTCTACAAGTGCACACCACGCTGGA 88748
Gallid          ATGGAA--TTATAATAGAGACCCCATATTCTAGAGCACGGCGAGCTCCCTCGGGGCTGTG 94246
                 *  **    **  *  * * *   * *  *    * *  * **  * * *   ****

Human           TGAAGGGGGGCAGATACCTAATCCG-CATTTAAAATATCTCAGTTCGCCGGGTTTTAAT- 88806
Gallid          TGTTCGGGCCAAGTCCTCTAATGCGGCACTTAT----TTCCAAAGCAACTCTTCCTGATG 94302
                **   ***   **    ***** ** ** ***     *  **   *  *   *  * **

Human           -ATGGCG-GCAGTTGTTAATCTTAGCTGTAGTACGTACGAAGACCATTGTAGAGTGGCGG 88864
Gallid          CATGATGCGCCTCTACTCTTCTTTGCTCAAGGTGGGCCAAAAAACTCATAAATTTCACAT 94362
                 ***  * **   *  *  **** ***  **    *  * ** * *     *   *  *

Human           GTT-GCATGTTTGCTTTTCTGGATGTATGAGGGGAATCCTGTTAG--TGTATTTTGGATG 88921
Gallid          ATCCGCTTCTATAAAAATTCACATATTTCACTAAATTGGGATAAGATTTCAGAGCAGACT 94422
                 *  ** * * *     *    ** * * *    * *    * **  *  *      **

Human           TTAAATAGTTGATGTAAACGTAGAGAGTCATAT-GCGCATTTTTATAATAGAGTGTTTTT 88980
Gallid          CAAGTTCGCCCATTAACAAATAATAATCCACATTGCCCGAGTTTGCATTTGCATGTCTTA 94482
                  *  * *   **  * *  **   *  ** ** ** *   ***  * * *  *** **

Human           CCACGGACGGGAGGATCTGCGGGTTTCCACGCGCAGGCTTGTCTTCGTATTCTAAAGGAT 89040
Gallid          GAATAGCAATTGATATATGAAGCAAATCCATTACAGTATCGCAATTG-ACACCAAAGAGC 94541
                  *  *        ** **  *     *     ***  * *   * * *  * ****

Human           GTAGTGTGAGTTCCGGTAGCAAGTATGCAGGAAAACAATTGCCCATGAAGTGTACTTCGA 89100
Gallid          TTTGTGTAG---TAGGGAGCTGCGGT-CATGAAAGAGTCTCCCCACGTAATGCTTTTTAA 94597
                 * ****        ** ***     * ** ****     * **** * * **   **  *

Human           ATGGCTTGTCGGTATTCTCCGTGTGTGTTAAAAGAGATTTCATGTCGCTGTTTGAGAGGG 89160
Gallid          TGCGGCTATAGGAGGCGAAAGACCGTGT---ACCATATTTTGTGTTGAATATGGATTAAA 94654
                   *  * * **        *   ****   *  * ****  *** *    * **

Human           AACGAAACGGCTTTCTAAACAACTTCTTCGT-CACAAAAAAG-ATACGACCTAACATGTC 89218
Gallid          TTTCAATACAGATTCTATCGAACTTTCAAGTACGCGAGCATGCGCAATAACAATTTTTTC 94714
                    **      *****   *****    ** * * *  * *   *  * * *   * **

Human           GTGT-TCGGTT----ACGGAAACTGTTTT-----TTCGCACGAAATCCGCAATTTTAACG 89268
Gallid          GGCTCTCGATTTATAACGGATAGTGTCATAACCACCTGTCCTGGCGGCATCTTCTAATCG 94774
                *  * *** **    ***** * ***  *         *  *      *    * * * **

Human           GTTCTTGGGCAAACCATAAAAATG-GGTACAGCTTGAAGACATGCGTCTGAACAG--GGA 89325
Gallid          GTTTATTTCCATAGCATCAAATTCTGATTTCACTTCAGCATTCAATAATAAAGAATTGAT 94834
```

```
                ***  *   ** * *** *** *  * *    *** *  *        * ** *   *

Human           TGAAGATGCCTACGATTTTTTTGTTAGTGAACTTGCCGCG-TAGCGTGATC-TCTTTGTT 89383
Gallid          TGAGGAAATCCATAAGTCTTCGCTACGTGATCTCTCAGCACTGATACGATTGTGCTCATA 94894
                *** **   * *  * * **   *  **** **  * **  *     ***  *  *  *

Human           TGTATTAATTACTAAGGACATATCGCTGGGAAAGAAAACCTCTAGACTCTTTCCGTCTGC 89443
Gallid          CGTCTGAATAGCTATATCCAATTCTCCAGCTC-GCTGATTTGCAGACCTTATTAGTTTTG 94953
                 ** * ***  ***    **  ** *  *    *   *  *  ****  * *  ** *

Human           GCAGATT--TTGAAATACG--GCATATTCATATTCAGCGTGACGGTACTCCGATTCGGAG 89499
Gallid          AAAGTTCAGCTATCTTGCGTTGTATGTGAGGATTTGAGGTGTATGTCAGACCTTCAAAAG 95013
                  ** *    *    * **  * ** *    ***    ***   **    *  *    **

Human           GGGAAAAGGTCAAGCGAAGATAACATTCTTTCGTGATTGGAAACCGTCGCTCGTTTTCCG 89559
Gallid          TAGAGAGGGTTGCAGTAACATTACCTACAGTTTGGATCGCATTAGAGAACTCAACTTTAG 95073
                  ** * ***      ** ** ** * *  *   *** * *        ***   **  *

Human           GCAACACTACGTTACGTAAAAAGATTGTGAGAAAAGGATCGTTAAATTCTATTTTGTAGA 89619
Gallid          CGCTCGCCACAACACTGGTATCATTCTCGGAAATAACCTCTCTGGGATTTAAACTTCTGA 95133
                    * * **   **    *    *   *  ** *   **  *    * **   *   **

Human           TAGAA--ATGCCGGGT---TCGGAAATCATGTTGACTCTGCA--GAGTGGAATCATACTT 89672
Gallid          TCTTATGATTCGAAGTAATTAGATTATCAAGTATAGTTCGCACCGACTGGATAAGAGACT 95193
                *   *  ** *   **   * *   **** **  * *  ***  ** ****        *

Human           TTGAATAGGTTTTTCCAATAGGAACATTTTCTGGTATCGATGCTGAACGCGCTAAAAACT 89732
Gallid          CCAAATGTTGTCCACGATTTGCTACGTCCGCTGCAATCTCCGCAGTGGATAATAAAGCCT 95253
                   ***    *   * * * *  ** *   ***  ***   ** *       ****  **

Human           ATTAGCAGCTTTCTGCCATAGCGGTCTAATTTTTGAGACTGATAAGGACTTTCTGTCCAG 89792
Gallid          -TTAGAAACAAAAAGGCATCGC----TCCTTTCTGTAGATGAAATG--CTCATTATTC-- 95304
                 **** * *     * *** **    *  *** **    *** * *  **   * * *

Human           CTTAGGTTAAGTAAGATCAAAGACGTTTCAACGCTATCATTCGAAACGACTTTAAATT-- 89850
Gallid          CCTGGGG-AGGGGCGATTGCATTGGCCCTATCTATATCTGATATGGCGGCTTTTAATGTT 95363
                * * **  * *   ***   *   *    * *  ****        ** **** ***

Human           -GATTTATCACGTTGGTTGTCGAGATCGGCAT--GTTGGTCAG--GCCATATTGTTCGTA 89905
Gallid          GAAACTACGGCCATCGTCTTTTCGACAGACATTGGTTCAACGGCTGCAGCAGCGATTGTT 95423
                  *  **   *  * **  *   **  * ***  ***   * *  **   *  * * **

Human           TACGAT--TGGATCGATGTTTTCTTTTTGGCA---TAGCGCATTGATGTCGTCAATCTCC 89960
Gallid          GCCGAAAGTACATCGGCGTGCTTTCCAAAGAAGACTGATGCAGCGGGATAGCTTCCCTGT 95483
                  ***   *  ****  **  * *     * *   *   ***  *   * *     **

Human           AGTGCAGGTGACGG-CAGATA---AATGGGTATGGAGAA--TAAATGAATCCGTACGGGA 90014
Gallid          GGACCAAATACTAATCTAATAGCTAGTGCAAATGGAACACCTAAACCAGATAATTCTGCT 95543
                 *  ** *      *  ***   * **   *****  *  ****  *     * * *

Human           ACTGTCCTAGAAATAGGTAAAATTTTTATTTTGATAGTCATTGTATTTTCGAGAAACTGT 90074
Gallid          GCTACTTCTGTAGTAGGGCGGGATAATATTGTTTTA--TATTGAATAAAC-AGTACCCAT 95600
                 **      * * ****      *  **** *  **   **** **   * ** * *  *

Human           ACATCGAAC---AGGACGTCCTCAGTTTCTGATTCAGTGCCATCTGTTATGAATAGGATT 90131
Gallid          AAATCTGATTGGAGGGAGCCTATTATGTCAGATGGAGCAGAAGCTAAAAAATTTTCGAAA 95660
                * ***  *    ***  * *     * ** ***  **    * **   *    *  **

Human           CCTGGTTTTGTGCTTGAGATTCGACATTTCAACGTGATTTCTTGGTAAGAATTCGGAACA 90191
Gallid          GAACCCATCCCCCTAGGAAT---AGATTCTGTTATGACTTTTACTTTTGAATCCAGAGCA 95717
                       *    ** *  **   * ***      *** ** *   *  **** * ** **

Human           ATGTGATT--GCAATCAAAAACAAGCTGTAAGACATGATTT-AGTTGAGGAGAATACATG 90248
Gallid          -CGTAACCCAACAAATTTTGGCAAGGGACGTAATATTTTGCGAATTGAGGAATATAGGTC 95776
                  ** *     ***       ****       * **  *   * *******  ***  *

Human           TT-GGTACAAAAAAACATTTTATGTT-TCACCTTGCTTTTTAAATACTGTGTTTTTTTAA 90306
Gallid          TTCGATTGTCGATTTCACTTCCTGGTACCGCTCTCGGAGATGTGTAATAAAAGTTTCATT 95836
```

```
                  ** * *     *   ** **  ** *  * *  *       *   ** *     ***

Human           GGGATGAGGTAATTTTACATGACGGATGGAGGACGCGGGTGTTCAGGCGGGGTGTGTTTT 90366
Gallid          TTTACGACGCGTGTCT-CGGAGTTTCTCCCGCACATCGGAACTTAACGAAGTTGCATTTT 95895
                   * ** *    * * *         *   * **   **    * *      * **  ****

Human           TATAATCCTGCGTATAAGTGATAGAAAAGTCATAGTAGGCCAGTGTGTTTTTAAAAAGCA 90426
Gallid          TCTTATCTTCCA---AAACTATAGCCAATTTAA-------CAATGTCTTCTCCAATTTTA 95945
                * * *** * *    **   ****  ** * *         ** *** ** *  **    *

Human           TTTAATTTTTATAAATACATGTAGCCATTCTGTTATCTGCGGAAACGTCACAGACAACAA 90486
Gallid          ATCGCTTCGTCTAG-TTTTTGTAGTTCTACTGTCGTGTCCA-AATCGGTATATGAACCAA 96003
                 *   **  * **  *   *****   * ****  * * *  ** **  * *   * ***

Human           AATACGTTTTCTCGTTGGTCAAAGAAT-TAATTTGGTCGATCATAGTTAAGACTGATTTG 90545
Gallid          ATCTAGTTTCCAGCTCATTTAGACGTTCTAGTTCTGTCAGCACAGTTTCATGGCGAGTTG 96063
                *    **** *   *   * * *   * ** **  ***         ** *    ** ***

Human           ATTTTT--TTTA----ATTCGTCTTTGATGTCCGTAGCGTGAAGTTGTCCGCAGCGCTGT 90599
Gallid          CCTTCTCGTTCACAATATCTATCTCTTTCATCAACA-TATCTAATT-CTCGCCGACTGAT 96121
                  ** *  ** *    **   *** *    **   *   *  * **   *** *     *

Human           GTGCTTAACGTTGTTCTTTTAGATAAT-AGTTCTTGGCATTTGATCAAAAGCATTGAATG 90658
Gallid          CCAACCACCAACTTGCATTGATGTCATTAAATTTTTCCACGTTGTTAGTTTTTCCGGAAC 96181
                      * *    * * ** *  * ** *  * **  **  *  * *         * *

Human           GTCTTCCAGCGTTAGCCGATCTATG-TAAGTTCTCACGATGTCTGGGGCC--ATGGCCGT 90715
Gallid          GTCTA--AATCGGAACCAATTTCTAATAAATTCCCAAGCAGCTCAGATTCGAAAGCCTGT 96239
                ****   *      * ** ** * *  *** *** ** *  *    *   *  * * * **

Human           GATCATGGACAGCGAGATGCAGGCTGTTTTCATGGAGTAC-ATTGATTGTTTGTCGTTGA 90774
Gallid          GAT-ACAGGTGATGGTACACGTGC-GTTACTACGTGATGCTATTAACACCATCACGTCAT 96297
                *** *  *      *  *  *  ** ***   * *   * * *** *      *  ***

Human           CTATGTCTGGTA------ATTTAA---TGAGTACGTCTCTGTATTGGATAGAGCATAACT 90825
Gallid          CTATCACAGATAGAGAAGATGCAAATCTGTTTATTTTTCATATTG-ATCTGTAACAACT 96356
                ****  * * **      **  **   **  **  * *   ***** **      * ****

Human           CATCGATGATTGTTTGCATCTCATTGTATTCTCTGTGAACTGCTATAAGACCAATGCATA 90885
Gallid          ATTT--TAAATCTTGATACGTCGCTCGAGCTGGCGGTAAGTGCTTTCACACTA-TACAAA 96413
                  *   * * * **   *  **  *  *      *  ** **** * * ** * * ** *

Human           GCAATCT---GATGTTGATCTCGGCCGCAATGGCGGTCGGAACGACTAAAGGTACAGTG- 90941
Gallid          GCCACCTTTAAATAGTATTCCCGGAGTGAATGACCAACGACATCAATGACGGTTTTTCCT 96473
                ** * **    **  *  ** ***    **** *   **  *  * * * ***

Human           --AGCTCCCAATCACCCAGTTTCAATAGCTTTTCTTCCGAGTGCGTCGATAAAGGTGGAA 90999
Gallid          ATATCTCCCAATCTTTGTGTAACAACAGCCGATGAAGTTTTAGCCACAATGTCCGCTGCA 96533
                  * *********     **  *** ***   *          **  * **    *  * *

Human           TCAATGTCAAGG-TGTCACCC--TTTTCCCAGGGGAAGGGT-CCTGTGTTCTTTATGGCA 91055
Gallid          TCTATCATAGGACTACTGCCCGATCCTCCCATTATAAACGCGTCTAAAGTTGTATTCATC 96593
                ** **   * *  *    ***  *  *****    **  *   **     *  *  *

Human           TACTGCTGGCCGGTCATCGGCTTTCTCAAAATTAGTTGATTACCTTCCACCTTCTGCAGA 91115
Gallid          CATCGAACACCCGCCTCAAGTGCTTCCATTTCTGCTAGGATTAATTCAGCCTTTCGAATA 96653
                 *  *    ** * *     *   *  **     *  * *  *   ***  ****  * * *

Human           ATGGTTACGACCATGGTCCGTAATACGTTCCG----GATGTGGACGT---AATCTTTGTT 91168
Gallid          GCAACGGCTTCTATATCGCGTATTAATGTACACAGTGATATAACTCTGTCAGTCAATGTT 96713
                       *  * **    **** **   * *     *** *     *   * **  ****

Human           GGAGGAGACGATGGGATAAAGACCTAAATTGCCGCTACCTATTAGA-TGGTGGTGAGCTG 91227
Gallid          TGATTGGA--ATTGGAAGACGTCTTTTCAGAATACAATTTTCCGGAGTTATGATAGAGTG 96771
                 **   **  ** ***  * * * *         * *  *    ** *  ** *    **

Human           GGATCGGTATGACGATGTTCATGAGCTTGCAGAGGGTGCTGATATCGGAAAGTGACAGTT 91287
Gallid          TATCTACCAAAATGGCGGCCATTCTTTCATAGATTCCATAAGGATCAGCGGTACCCACTT 96831
```

```
                            *  * *  *  ***    *   ***          *** *       ** **

Human               TGTGATCGAAAGTGCAGTAGACGGTTTCCATTTTAT--ATGGATGATTCGATAATAAGTT 91345
Gallid              CCTTTTCTATAGCGTCCAAAGATTTATGCAGTTCATCGGTGACTTCTTCAACTTCCAATG 96891
                      *  ** * ** *    *     * * ** ** **   **   * *** *     * *

Human               GGAGAGGTATGGTTTCTCCTATGGCGTAGTTACA-ATAATGGGTTTCGCTGATCTGCGTA 91404
Gallid              CCACTAATATCATCTTGCTTAAAGCTAAGTTTCCTATCTTGGAATTCTCCG---CAAGAA 96948
                      *    ***  * *  * **  **  **** *  **  ***  *** * *      * *

Human               TTGCCTGTTTTAGACCGACTCTCTAATAGAGCTTGGTTGGA-TGAACAGTGAAGAGGCAG 91463
Gallid              CCCTTTCTATAAGGTCTATAGATTCAGATGCTTCAGAAAGAACCATTCCTGTAGACGCGA 97008
                         * * * **  * *     * * *    *  *   **   *    ** *** **

Human               AGCGTTTTGTAAGA---ACTGGCATGGTCTGCTTACGAAGCTAT-CCGTGCCCTCAGAGC 91519
Gallid              TGAAAGTAGTAAAATAGACAGGTATTGTAAAGGCTTTAAGTAACGCCGCCATTTCATAGC 97068
                     *    * **** *   ** ** ** **         ***  *  ***     *** ***

Human               ACGAGTATTGTATTTCGCTATCGCTGTTTAAACATGAATTTGATTTCATGCAGTATTCGT 91579
Gallid              CT--ACAACGGATTCT-CTATC-CGATGTAGTTCTGG-CCCCATTCTCTATTATATAATT 97123
                          *  * ***  *****  *  * **     **     ***   *    ***   *

Human               TTATCACTTTGTACA-----TCATCTTGTTCGTTTTTAAGATGTCAGATTCGGTAAAAAA 91634
Gallid              AAATAATCTGGCAAAAATATCCATCATGCATACATATAATAATCCCCCATCACTATCATA 97183
                      ** *  * * * *      **** **      * *** *   *    **  **  * *

Human               TTGAGCGTTA----GGACTGTATGTCTTGGGATTGTAAC-ATAGT-TGTTCTCTGTGTGC 91688
Gallid              CGGACTTCCTACATGGACTATAATTCTCAGTCCTGAATCTATAGAACATGCAACATCCTC 97243
                      **          ***** **  ***  *   ** * * ****    * *    *   *

Human               CGTGTTGTACAGGATATC-GCCTAAGGAGCCTGGTAGAGATGCCCAAGGATTTGTGGTTG 91747
Gallid              TATTTTATCGCGGATGTCAGCCAAAGGATAATGAGATAAATATTCATCACTCCACAATCG 97303
                      * ** *   **** ** *** *****   **  * * **   **    *      * *

Human               ---CGGCA--AATGTATCGCTGTCTGTTTGGGTGTGATCGTACAATGCTTTTCTAGCCGC 91802
Gallid              ATCTTGCACTAATCCATCTTCATCT-TCTGTCAACGATTTAAAATTAAAAAGTGGTCTAT 97362
                         ***  ***  ***    *** * **     ***   * * *          *

Human               CTCTTCGT---TGTGTGGGTCCGTTCCCATCATACACGATTCCCTGCCTCTGGGGTTATG 91859
Gallid              TTTTTAACAAATGTTCAACTGTGGTATCTTC-TCCACGATTTGAAGTAGTTGGCGATGAA 97421
                     * **      ***     *  * *  * ** * *******    *    *** * *

Human               GGGCAATTTGAAAAAGTTA--ATATTTGTCGTGACCGGAG--TCAGTA-CGACTTCGCAG 91914
Gallid              CCTAAGGTCGGCTCATCCGGTATATTAGATATGACAGGGGGGTAAGTGTCTACATTCTCC 97481
                        *  * *    *      ***** *   **** ** *  * ***  * ** *

Human               ATAGCTTGTTGACCATGCAGCAGTATGGAGGGTGGGTTT--TTGTTAATTCCGCCGAATG 91972
Gallid              GTCACAT-CTGACGATTCTGTAGAAGTGACGGCTTCTACAATTCTAGATTCCAGAGTTCG 97540
                     *  *  * *  **** ** * * ** *  ** **    *    ** *  *****   *   *

Human               TTATGATGTTT---AGTGCCTCGCCCTCGGAGGGATTAGGTTTTTCTATCC--CGACGTG 92027
Gallid              TTTAGATTTTCTCGAGTGTTTCCTGCTCAACGAGCT--GCTTGATCCATCTAGCGATATA 97598
                    **  *** **    ****  **   ***   * * *  * **  ** ***   ***  *

Human               ATGCCGAATCCACGTATTTATGTCCGTGTTGGTAAACGCGTGTATCGGGAAGGCGGAGAA 92087
Gallid              TTTTCCGAACTTGATTGTGGTGTCCATAAAGGACGACGCCT---TTTATGTCTCGCATAA 97655
                     *  *  * *    *  *  ***** *   **    **** *   *        ** * **

Human               AAGGTTTTGTATCTTGCTTCCCATGTCTGATTTAATTCGCTTAAGATTGGCAGTGGCGGT 92147
Gallid              TTCCGTATGCAACCTTCGACAGATGTTGTACCTATTTTATTTTCAGCTACTTTTGCAAAT 97715
                         * ** * * * *  *  ****   *  ** **   **     *     **    *

Human               G-GTAGAACTAAAACCT-AAGCCCATATCTA----CAAAACTTAGATGCTGGGTGAAGTT 92201
Gallid              GTGCCGAACGAAAATTGGAAGTTGGTTTTTGGTGGCGGATCCTCGATAACATCCGAAAAT 97775
                    * *  **** ****    ***    * * *     *  * * * ***       ***  *

Human               GTAAGTGGTTACGATATCTTTAGCTTCCGCGGTGACCGTGGGATCGTCTAAAATTATAGA 92261
Gallid              ACACCCTGCCACGCGGTCACTGAAATTG--AATCTTTAGTGGATGGATCAACAATAGTGC 97833
```

```
                  *    *  ***   **  *    *      *       **** *   ** * **  *

Human           TGTGGCTGCCCTGGAGCTGTACAAC-AGGCACTCGACGTCGAAATTATCCGTCCGGACTA 92320
Gallid          TATGGGTATCGTCAAGGGTTTCTCCTGGGTTCTGAATATTGATGCAATCTCGACAGTCTT 97893
                * *** *  * *  **   * *  *  **  **  *  * **    ***    * * **

Human           GTGTGGCCGCAAAGCCCGGATGGATTTTGCTTTTGCTTTGGATCGCTATGGCAACGGGAG 92380
Gallid          TGGTCGATGGAGGTGCC--ACAGATTCGGTATTGG--TAGGAGCATCATTATA-CGAAAG 97948
                  ** *  * *    **  *  ****  *  ** *  * *** *   **   * **  **

Human           ACAGTT-TTGAGTGCATGGCGGCAAGCGTCATAATGCTCAACAGGGAATTGGGGCAGAAG 92439
Gallid          ATTATGATCGAGTGGTCTGGAATCCTCGCCATTTAAATCTTCTGCTTGTATGCAAGGATA 98008
                *   *  * *****    *       ** ***     **  * *    *  *    **

Human           GCTGAGTACACGGAAAACGGTGTTCGT--TGCCAATTATAAAATT--CTAATGCTAATGG 92495
Gallid          ATCTGAACCTTGGATAGTTGTATCTGTACTACCGATGACAAGTTTGGTTAATCTCGGTTG 98068
                        *  *** *   ** *  **  * ** ** * **  **   ****     * *

Human           GGGCGGGAGAGG-------AAAACCGCCGTCGTTTCTTT--GATAGTGAGGAAATGTATT 92546
Gallid          TGGCCTAGAAGGCGGATCAAAATCTGCCGACACTACTTTACGGTTATCTTCCTCTCTAGC 98128
                 ***     ***       *** * **** *  * ****  * *  *       * **

Human           TAGGAAAACTTGGATATCAGCATTCATGGTGCCGCAGATTCCCGGGTCAGAGAAGAATCG 92606
Gallid          TCGACCCAAATCTAAAACTATATCGGAGGTCCCATAGATCCGCGAAACGGTCGATATTAT 98188
                * *    *  *  * * *   **    *** **  **** * **   * *   * * *

Human           ATGAAATG-GGATTGGCTGATAGTA-CCGTCTTAGGGTGGTGATCGGTGAGATCAGGCAA 92664
Gallid          CTCGTCTCTGGGTGGGCAGATAGAAACCGTTGATACAAAGTATACAAATGTTGCAGACCA 98248
                 *    *  ** * *** ***** * ****         **   *        *** * *

Human           AGCC--CATTGTATAGAATGTGTTGTAGTGATTGAAA-ATGAACGGAATTGCCGTGTCGC 92721
Gallid          ATCGGGTGTTGCGGAGGGGTCGGCATACGATGTGAGATATTGATAAAAATAACCTTCACT 98308
                * *     ***   **     *   **     *** * **  *   ** *  * *

Human           TGCGCAGGGTCTAAGGAGACCTCCACTTCCAGTATTTTCGTATTT-TCATTAATGTAGAT 92780
Gallid          TACACGGACTATACATGCCTGTCCTATATGCCCATGTCCATGCGGATCAAAAATATAAAT 98368
                * * * *  * **        ***  *      ** * * *     ***  *** ** **

Human           TAGCGATTTAAACAGCTGTTTCGCGATAGATGATGTATTGGCTATGGTGTAGCCAGGACC 92840
Gallid          TCCATCGCCGGATCTGTACACTCCAATTCCTAAAACGCCAACCACAATTAAACAATAAAC 98428
                *          *    *      * **   * *        * *   *  * * *  * *

Human           CATA----GCTTCTCGTATCAGAGACATTA---GGACGTCGCTGCTGAT--CGGATTCTC 92891
Gallid          ATCACCCCGCTTTTTTTTCCATATTTGTTCTATGAACGTCCGCGCAGATATTTGAGTATC 98488
                   *    **** *  ** *    **    * *****   ** ***    ** * **

Human           C-------TGGAAACAAT--CATCTGGACAGATAAACGGTTCCGTGTAGAATAAGTCTAG 92942
Gallid          TAATATAGTGGGCGTGTTTTCTTCTAGATAGAATTCAAACTCCCCAT---ATGAACTCGA 98545
                        ***      *  * *** ** ***        ***   *   ** *

Human           AACTAGTTCCTTTACGTTGACGCCAGCGCCACAGGCCTTG---TTATTTGATAGTGCCGG 92999
Gallid          AAATGCAACACACAAATTTCCTCCTGCATCTTTTGATATAATTCTATTCGGCAGATCATG 98605
                ** *    *    *  **  * ** **  *    *   *     **** *  **  *  *

Human           GAGTACGCA-GAAGTAAAAGATCTTGCTCAGGATGGTGTTTTCGTTCGATGGTCTGTCAT 93058
Gallid          CGGAACGCACGTTGTATATGTCCCATCTTCAGAAGTCGCTATTGTCCA--GGCCTTTCCC 98663
                  * ***** *  *** * *  *   **   ** *  * * * ** *   ** ** **

Human           TGTCGGTGAAGACGACGC-TTGAATCTATTAGATTCATTCTTTGCA---CAT-CGGAGAT 93113
Gallid          TGTAATAAAAGTCTATCTATTGCATCTGATGATATGACTGTATCTAATCCATACGCAAAA 98723
                ***     *** * *    *** ****  *    * * * * *  *   *** ** * *

Human           TTCGTAATTTCTAACTCTTACGGTGTTCTGTGTCAGTGGTGTATCATCCGCTG---TTAT 93170
Gallid          ACTATACGAAGAAATGCCAACGATGAAC-GTAAACATGAAACTGAAGACGCAGGACTTAG 98782
                    **      **  *  *** **  * **     **       *  *** *   ***

Human           TTTTGCATTCGTGTCGTTTCTGGGCATGGTATGAACGAACGGGCAGAACAGACGTCCGTC 93230
Gallid          GTCCGGAGCAAACTGGTTTCTAA-TACCAACCGCCACTACCGTTACATCAGTCGT--ATC 98839
```

```
                  *  * *      * ******     *      *      ** *  * * *** ***   **

Human             GAACAATGCGTTGGCGAAATTCACCAGAGGTTCGCCGCAAAGTTGCTCGTTGAT-GTTGG 93289
Gallid            TATCACCATCTTTGCCCATTCATCTAGAA--TTACCTCAGGGTTATCCATAATTTGCCCA 98897
                   * **     ** **  * *   * ***   *  ** **  ***   * *   * *

Human             AGATAGAGATTGTCCTCTTCACTAGGCGAATTAGCGACACAAG---ATTTCTGTAGTGAG 93346
Gallid            AAATATGGGTCGTGTTTTCGATCAAGCGAACAGAGTCCAGACGTCGATTTATCTCTTAA- 98956
                  * ***  * * **  * *  *  * *****       ** * *   *** * *  * *

Human             CGAAAGCTGCTCCTGGGATCAGTTCGTCACCCATGTGGTTAGAGATTAGCATGATCATCT 93406
Gallid            -CACGTTCGCAGCTAGCATGGGTGCGTT--CGATGTAGTTCTTGAATATTTCCTGCAATT 99013
                    *     **  ** * **  ** ***   * **** ***   ** **       **  *

Human             CGAAGCTG-TTGCAAAAAAGAAGTATA-TGTTTCATGTTAAACCAATAAGAAATACACTG 93464
Gallid            TGTAGTAGCTTGTCTGCTGTCAGTAGAATCTGTCATTTTACACTCA----ATTTATA-TG 99068
                   * **  * ***         **** * * * **** *** **  *    *  ** * **

Human             GCTAATTACTTGTTTTAAGATCATGAAAGCATGCTTGTTTCCATGAACTAAGATCTCGAT 93524
Gallid            ATTGAAAATATGCGTCGAAACGACATAAGCTCCACTGCTGCCATAAAGTAT--------T 99120
                    * *  *  **  *  * *  *   ****     ** * **** ** **         *

Human             AACGTAAGCCA-ATTCTGGGTACGAGCCACTCGTCAAGCTTTCGGTGA----CAATTTTG 93579
Gallid            TATACAAGTCATATACTGGGCTGAAGTTAAAT-TCGGATATTTGACGAGGCGCAACGTTC 99179
                   *   *** ** ** *****    **  *     **     ** *  **    ***  **

Human             AGGGTGAAGTCGTAGTTGTGGGAGTTTGTCTTTGCATGTTCCAGCATTTGATTTGTACG- 93638
Gallid            CACATCAAACGTAACTAGTACAACTACGTCGTC-CAAGACGGGACCGTTGACATAAACGC 99238
                      * **     * * **   * *  *** *  ** *       *  ****  *  ***

Human             --GGCTTCGTGGAAAGAGATTGGAGCTAGAGGTAAAGGCATGTTGCC----TATCATTAT 93692
Gallid            CCGGATGTGGAGAATAGATTTCATATTATGCATTATCACCGTTTGCCCTCCTACCAGTAT 99298
                    ** *  *  ***     **     **    * *   *   *****    ** ** ***

Human             T---CTTGGCGTGCAGAGTACTTCTGTGG-----CTCGGTTTTTCTGGATATAC--GTGA 93742
Gallid            TAAACACAGTATCTATAATATTTACGAGAGCATCCGCTGTTACTCTTTGTTTAGTAATGA 99358
                  *   *   *  *  * * ** **  * *      * * ***  ***   * **    ***

Human             GATC-------AAAGAAG--GGGTGTCTTTCGGTCGG---CAGAGTTGGATTGTCCGGTT 93790
Gallid            AATCTCTAGCAAAACTAGTCAGATCTACATCATCTGGAGCTATAGTTTCGATGTCCAGTG 99418
                   ***       ***  **   * * *   **    **    * ****    ***** **

Human             TGTAAAAGTCT--------TCAACGT-TTAGTTCGTTTTTTAAGACGTTGGTGTTTTTGG 93841
Gallid            TACAAAAGTTTATTTCATCTCGGTGTGTTAAATTGTGTCTTGATGACAAGGTAACTACGA 99478
                  *  ****** *        **   ** ***  * ** * ** *      ***   *  *

Human             GTACTTCTTGTCGACTTTCGTATAAGTTGTATAAGGTCTGTA----AGATCCTG-GTTGG 93896
Gallid            GTG--TAGTGTCATCCTCAGTTATTACTGGGTA-GTCCCGCATCACAGATTCTACGGCAA 99535
                  **   *  ****  * *  **      **  ** *  * * *    **** **  *

Human             GTTTTCTCGGGTAATTTTCAGTTGACATAGTTGAGAAAAGCGGTGGCCGGTAGGCAATGG 93956
Gallid            ATTCTATTTCACCGGCCACGGATTTATCATCTGTACCAATCGAGGCCAGATCATGGTCCA 99595
                   ** * *            * * *     *  **    ** **  * * * *

Human             TT-CGTTTTTCATGAGTCGTTCGACGATGGTCGAGTCGTGGACAATGGGATGGCAAAC-C 94014
Gallid            TCATATCTTGCAATACTCCTCCTACATTTTCTATAGCACTGACCAATTCATGCGAACTTC 99655
                  *    * ** **  * ** * * **  *        *   *** *    ***  **   *

Human             GACGGCAGTATGTCGG-CAAAATCAATTCGCTGCACGACTTGGTCTTTATTGTGGAAAAA 94073
Gallid            TATCGCGATATGTAGAATTAATTTCAGATAGTATCGACCATCTTCCCAAAAGCCAACAAA 99715
                   *  **  ***** *    ** *  *     *      * *  **   *  *   * ***

Human             AACGCTGGTGGGTAGGTTG--TTTTCCATGGTGTCGTTTAATTTCACACGTGTTTCCATT 94131
Gallid            TGTCCTGCATTCCTGGTGGAGTATTACATGATGTCAACTT-CCTAATGTGCAGATCCAGT 99774
                     ***       *** *  * ** **** ****   *    * *   *    **** *

Human             GTACT--GAAACCGGTTTCTGTCGGTATG-TAGATACCCAAAGGGAAAAAGAATGTCAGT 94188
Gallid            GAATTTTGCAGACGTGTTAAACCAGCAAAATCGGCCCCCAACACAGAAAAAAGCTTCA-C 99833
```

```
               * * *  * *  **  **    * * *   * *   *****     **** *   ***

Human          TGTAAGCTTTGTTCTAGCGGATC-GTTGACGTC--TGTGTTTTTGTAAACTTTGTGTAGG 94245
Gallid         AGCA--TCTTGGCCTAATGCATTTGCTTGTTCCATTATATCATCACATAACAACAATTGT 99891
                * *    ***  ***  * **  * *      *  * * * *  *   * *       * *

Human          TGGTCGAATGCGACTAATTTATCTCCCAGTTTTAACGTGTTCAGTTTTAGGTCGGCGTAC 94305
Gallid         TTCCCAGTTTCTTCTATAATTGTCCGCAACTTCATCGAGTCGAGTCTAATGTCTGCTATA 99951
               *   *   * *  ***   *    * **  ** * ** **  *** * * *** **

Human          GCGCGGCTTTT---GTCAAAGATTTCT-GATTTGTTGTTAGTGTCTTGTTGTTCTGTCCC 94361
Gallid         ATGTTGTTTGCCGAATCATAAAGCTCTCGAATAGCTGTCATAACGTCAAAGATTT-TCCA 100010
                 *  * **      *** * *  *** ** * * *** *      *    * * * ***

Human          GGCGTTCACGGTGAATTGAGTAAAATCGGCCATGATTGCACGGTATG-CAATCGC---AG 94417
Gallid         GGTTCCCA----AAGACGAATGCAGTTCGCTATGATGATAAAGCTTGACAGTCCCCCCAG 100066
               **    **      *   ** *  * *  ** *****   *  *  ** ** ** *   **

Human          TCACTGCGTTTTC---TTTGCCCATAATAAAGTGACCATATGAGATGGGGGCGGAGACGA 94474
Gallid         ATATACCAGATACAAATTTGCAGGTAAAAAAGTCTTTAAAT---ATCGATGCAAATACAA 100123
                 *   *   * *   *****   *** *****    * **   ** *  **  * ** *

Human          CCTGT-GTGGAGATGTACTGGGAGAGTATGTTGGTGAGTT---TTTGGATAGAGCCGATC 94530
Gallid         CCAGTTGCTTGGATTTATTGGATAAATGTTCCACACAATCAAATGTACGTTCCGCCAAGT 100183
               ** ** *    *** ** ***   * * *        * *    * *   *   *** *

Human          GGTCCTAATAAA-ACACCGTCTAGGGGGATGTTTTCTT-TGGAAGTGTAGTTGTTCGTAT 94588
Gallid         AATTCAATTGAAGATGCAATGCCGTTCCGTGCATACTAATTAAGACATTGCTATTCGACG 100243
                 * * * * ** *  *  *   *      **  * **  *  *    * * * ****

Human          CATTTAGAATGCTTTCCGTGACCGATTCCATCATGTCGTTCAGAATTCT-ATAGATATAC 94647
Gallid         GGTCTGGTCGAGGGGGTAAAAATGGTCTAGCTTCCTGGGGTAACATACTTATAGCGACAC 100303
                 * * *              *  * *         * *   *  ** ** ****  * **

Human          GAACTAT-TATTATTCCTGTTCAAGAAAAATAATGACGTTAA---CAATCTGTTTTTTAG 94703
Gallid         TAATTACATCGTATAATTCTCCCAAAAGCATTTCTATATTCAATGCATCATCGGTAGTAG 100363
                ** **  *  ***   * * * * **  **     *  ** *   **    *   *  ***

Human          GC---TCTGAAACATGTTGCTGCGCTGTACTCGGCTCAGAGCTTGTTTATTATGTACTTT 94760
Gallid         GCGCATTTGATATGTCTTGTGTAAACGGAATAATCGCAGAAATGAG--AGCGTTTAGTTT 100421
               **   * *** *  * ***       * * *   * ****  *      *   * ** ***

Human          GTTC---TCATTCATGGTCAGGACGATAAATTGCGGGGGAGATTTTCTCAATAAGGTTTG 94817
Gallid         ACCCCGGTGATTTATGAAACTGTCTAAATATGGCATACCATAATCTCTAGCCCAGGTT-- 100479
                  *   * *** ***     * * * * ** **      * * * ***      *****

Human          CATGAATGCGTGAATCAGACCTCGTTCGAGCGAATCGGCTGAATTCTTTAAAGATCTTAA 94877
Gallid         -AAGACTTTTAAAGCCGTACCTAAACGAGGTGTAGAAAATATAGCTGTCGTTACACTTGT 100538
                * ** *     *  *  ****        * * *      *  *    *        ***

Human          AACTGTATGCAA-CGCATTGATGTT----TAAAATCTGATCGATGA----CTGTGTTTTT 94928
Gallid         AAGTGCATCCAAACACACAGTAGCTATAATATATTTCAAGAAATGAGGGGTTGTGCTTTC 100598
               ** ** ** *** * **  *  * *    ** * *   *   ****     **** ***

Human          AAAAGTGTTTTCCAGATGCTCCAGACA---GGCAGCACT--CAAATCGAACGATATGTTT 94983
Gallid         AGGTGTGATTGGCATGGGTTCGGGATACCGAATTGTACTAGCGAGACGAATCATCTCACT 100658
               *   *** **  **   * **  ** *        * ***  * *  ****  ** *   *

Human          ATGGGGTGTTTTTCTGAGTGTTTGGCTACCATGATGGTGGTTTCTTTTGGAGCA------ 95037
Gallid         CTCACGTTCCCCATCAAATTCATCCTGCTCAACATTGCTGTCGACATTAGAATAATCAGA 100718
                *   **          * *   *       **  ** *  **     ** **  *

Human          GTTACGTCGTTTCCTGTGGCGACTCTGGGTAATTGGATAAAGAAGAGAACC--TTCCCGA 95095
Gallid         GTTGGGCAAATTTGCATCCAAATTTAACACATTTGACTCGTCTGGTGGAACAATACCCCA 100778
               ***  *    **    *    * *       * ***  *       * * * *  * *** *

Human          GGGTCATTCGACTCACATCGTTGAAGCGAATTACG------TTCGCCGCAACGGCGATCG 95149
Gallid         GATTTCATTGGCTCG-ATTGTCAAACCATAACATGAGTGACTCTTTTGCAGACCTTATTG 100837
```

```
                *  *   * * ***  ** **  ** *  *  * *      *     ***      ** *

Human           GCGTGGTGAAAAA--GTTGATCCATTCTATTTTGT--TGCAGTAGATTCCGA--GCAAGG 95203
Gallid          AT-TGGCGGAACCCCACCGCTTCACTCTTACTTGCGACGTGATCCATTTCAGCTACAAGG 100896
                   *** * **       * * ** ***   ***    *   *  *** *     *****

Human           ----CTTCAAAACTGATGTTGTAA-CGGCTCGGATCGTCACCGAAATAAATTCGCAAATT 95258
Gallid          ACTGCTCGCGCAGCTTTAATGTAATCCTCATGGGGGATCGGTGGAATGCATCGACCTATT 100956
                    **     *    *  ***** *  *  **    **   * ***  **   *  ***

Human           GTCAAAAAGTTGTTCGGCCGTGTATGTTTTAATGTC-------ATTGAAAAT---ATTTA 95308
Gallid          ATTTGAGATATTATTGCCCGTTCTAAACCTAATGCTTCTATAAAGTGGGAATCAGATTCG 101016
                 *   * *  *  * * ****        *****         * **  ***   ***

Human           GAGGTGCTTCGATTTTAGGTAAAATTTCGGTCGCCTGCCAATTTTCCATGTTTCGGCGTT 95368
Gallid          GAAATTACACAATTCTCCATATAATTGGATAAATATGTCA---TTCCGGGTC-CGTAGCT 101072
                **  *    * *** *   ** ****         ** **   ****  **  **  * *

Human           ATGCAGCACACCGGTAATTGTGAAACTTTAATAGTTAATTCATGTTTTGGTTCCACTTGT 95428
Gallid          TTCAATCAACCCAAAGACCACGTTACC--GATGACGGCAACGCATCCAACTACCTGTTGT 101130
                 *  * **  **    *    *  **    **        *   *     * **  ****

Human           GCACGGTCGA---TTCCAGTTTT-TATAGATTCCTGTGATCTGACCGCTGAAGTGTCTCG 95484
Gallid          ACATCATCAAAAATATCACCTTCATCCACAGAGCCGTAATTT--CGTGTAAAATATGTTT 101188
                 **   ** *   *  **  **  *  * *   * ** ** *  *   * ** * * *

Human           CGACGAGGAAACTCGTTTAGCGCGATCTGTGCCTGTAGTTCTGGAAAAGATCGAGTCAAT 95544
Gallid          TTA-GTGCTGATTCAT---ACGCACGGCATACCTGTCGTACAAACCCACGCTCTATAAGT 101244
                  * * *   * ** *    ***      * ***** ** *      *       * * *

Human           CATAGAGAAAATTTTTCAAACGT----CTGGGCCAAATATCGTTCACGATAAAGACAGGG 95600
Gallid          GCCTCCAATAATTGTTTAGGAGATGAACTGTATTGGATAGCATCC-CAAGCCCGTCTGG- 101302
                       * **** ** *   *     ***      *** * * * * *    * * **

Human           CTAAAATTGCCTTGTGTCGTCTGTTGTTAGGACCGGTTGCGGTGCCATGTTTCTGTGAAG 95660
Gallid          -CAGAATTGGAAAGTTTTGCTT--TGGCCGCATCAATAGCACCGTTATCTTCCTCTAAAC 101359
                  * *****    ** * *  *  **   * * *  * **   *  ** ** ** * **

Human           AGTGGGACACCAACGACTATTTATCGAAATCTGGATGTAAATGTCTAGGTCCGATCTTAT 95720
Gallid          CA-ATGTCGGAAATAAGT-CTGAGTACAGCAGATAAATGAATTGCTAA--TGAATCTTCT 101415
                     * *   **  * *  * *    *      *  * ***  ***      ***** *

Human           ATATTCATACCAGTCGATGTCGCTGTAGCGATATTCCGGTTTTTAAGTTTTC---CATTA 95777
Gallid          GTCAATGAGGTATCCGACATAATAATATC--CGACCCGTTGCGCAACTTATCAGCCGCCA 101473
                 *         *  ***  *     ** *      *** *    ** ** **   *   *

Human           TGAAAGATTACTACGCTT-CACACGTGTTCAGAGGTTTATTATCTCTGAAAGAGT--GGA 95834
Gallid          TGCCAGGTTGTATCGTGTGTACAAATGGACTTTCATATGCTATGGCGGCAGATGCTCGAA 101533
                **  ** **    **  *  ***  **  *     * *  ***  * * *   *   * *

Human           ATACACATCTACCGAAT---GTATTGTGTACGTGCGAGTTGTCGATGAG------CGATA 95885
Gallid          CTTCATACGATGTGCGTTTAGTACTATTCGCTTCCAAGGCAGGGATAAGTTCTTTCGAAC 101593
                 * ** *      *  *   *** * *   * * * **     *** **      ***

Human           GATATGTGGCGACCGTGTTTCCTAAGCAGAATTCTATATATCTAGAATACTATCCGT--- 95942
Gallid          CCAATGCGCCAACATCTTCTGCAAAGCGATCCTTTATTTGAAAGGGTTGGGGGGCATGCA 101653
                   *** * * **    * * * ****     * *** *     *  *      * *

Human           ATTTTTTGTGTTATCTGTGTCGCTATCTTACTGTCATTGAGATT-GAGCAGTGTACAAAT 96001
Gallid          ATCCTCTAAAGTATTGATCCACCAACGCTATTGTAAAGAGTATCCGTGTAAGGGGCGTGA 101713
                **  * *    ***   *    * *   ** *** *     **  * * *  *  *

Human           GATTTAATTTCGCTTCTTGGTCCTAAAGTAGCTCAGCGAGTCATAATTCATTTTAAACTG 96061
Gallid          AAACTCTATTCG-TTGTACATCTATACATGATTACAAAAGGGGCAATCCAACC---ACTG 101769
                 *  *   **** ** *   **   *  *   *     **    *** **      ****

Human           CTTTTCGGTTTTCGTCACAAGCCGCATATGGGCACTGTTGATTCGTGGTTCTGGG-AAAA 96120
Gallid          GCTATAGATACCAGTCGAATACCCTCTTGAACAAACGGAAAATCATATATGAGTTCAAAA 101829
```

```
                     * * * *    ***  *  **   *      *  *   * ** *   *  *   ****

Human                TTTTTTTATGCTAGAATTGCATAAGCTTTGGTTAACCGTAGTCAAACATAACCGGGTGAC 96180
Gallid               CGTTTATGTGCCAAAGAGGCGGCCAGATCCCCTA-CATTAAACGAAAATTCTCGCTCAGA 101888
                       *** * *** * *   **       *    ** *  **  * ** **   **

Human                AACAGATTTCTTTAATGTAGTC-TATGAGAA-AATTCAAAAC-----TATAAACAATACG 96233
Gallid               AAGTAAATGTTCTGGCGTATACGTAAAAGCATGATTTGCAGCATCCTCAAAAACAGCAAA 101948
                     **   * *  * *   ***  * **  ** *  ***   * *      * *****  *

Human                CCATCAAGACTTTGAGAATGTCGTCTAAGGCGGTTCCTGCAATACAGAGGTT------TT 96287
Gallid               CCACTCACTTATTAAAGGCCACATAACACTCGGTCCAAATGGAGCTGATATACGTAAATT 102008
                     ***   *    ** *      * *   *  **** *        * **  *       **

Human                GTTTGGCAAAATTTAAACAGCAACTCTTGTATCTAAATATTAAGGTTACGGTAAAAAAAA 96347
Gallid               GTTCCGACTAAGCCAAGAAATAGCTTCT-TCTCCCGTCATTTCGGCCACCAGCCGACGCA 102067
                     ***  *   **   **  *  * **  * * **     ***  **  **      *   *

Human                ATAAGCGAGAACTGTGTT--TGAATGGTTTTGTTTACGGTAAAACATTGTATGTCGTTGA 96405
Gallid               AACGATGGGAATTCTCTTGCCAAGAGGTCTTATCCCACCTGACAGAT-ATGCGCCAGAGA 102126
                     *     * *** * * **    *  *** ** *      * * * **  *  * *   **

Human                ATCTTCTCAGTTAATATTT---CGAAATTTGCTTCTGTTGTATTACGATTACAGTT---T 96459
Gallid               ATTAAACCGACGAAATTTTGTGCCAACATTGATGCTTCTGGACTCCTCGTTTGTTTATAC 102186
                     **     *    **  ***   * **  *** * **  ** * * *   *    **

Human                GCCGGGCGAATGCAAAACAAACGAAGAAAACGTTTTGA--CGACTCATTACATACGGGTA 96517
Gallid               ACCTCGAGTATGGCAGAAAATGTGTCACGCCATGTCAATTCTACTTGCAGAATACTCATT 102246
                      **  * * ***  * * **      *   * * *  *  * ***      ****   *

Human                ATTTCGAGATTGTCGTTTAAGCAGTCTCGAAGTG----CGGTCCCGCCAGGCGTGAGACC 96573
Gallid               GTTCCCGAAGC-TAGAAAAAAGGATCCAATAGCGGCTCTTGCCTCCGCGGTTGCAGTAGC 102305
                      ** *   *   * *   **    **    ** *      * * *  * *  *    * *

Human                AGATTTTACCTTCGTGGCACAACTGCCTAAACGTAAAGAGTTGCCTAATGTCCCCGGTGG 96633
Gallid               ACCCCATGGTTCTGTCGGCGACGTTCTCGAAGATAAGGA---CCGTAGCGTGTCCAATAA 102362
                     *     *   * **  *   * *  *   **  *** **    * **  **   **  *

Human                TATTGATTTTGCTGAAATTACCTCAGTGAGGCATGGCGCGGTAATTCTTAACGCGTTTAA 96693
Gallid               AACT---TTCAGTGGCCATATTTCATCGGTCGTTACGACAGACATTATTCAGGCAACTAT 102419
                      * *   **   **    **  ***  *     *    * *  *** ** * **   **

Human                TACGAACAAAGTCATGAATTTAAAAGCAACCATTTCAAAAAGGGCTAATTTTGTGTATCA 96753
Gallid               CGCCAACCCTCTGTTCTCCCTAGATGCGATCGATATTAGAAAGATCTATAAAATGTAATA 102479
                       * ***    *  *     ** * ** * *  *   * ** *    **    ****  *

Human                TCGCATTC----CTAAGAC------AATGACTCACAGTTTTGTCATGTACAAGCATACAT 96803
Gallid               CGGCTTTCAATACCACAACTCCTAAAACCAGTCGTGATTACGTAGTATAGTCATGCCCAT 102539
                       ** ***    * *  **      **  * **    **  **  * **        ***

Human                TT-AAAGAACCTGCGTTTACTGTAAGCACGTTTGTTTCAAATGATGATTTAGATATGAGT 96862
Gallid               TTCAGAGAACATACAATAACACAGTAAAATCCTACTAAATATTGTATCTGCACTATTACT 102599
                     ** * ***** * *  * **       *    *  *  * **  *   *    *** * *

Human                TCATTGAATATCAATATACGTGGACCTTATTGCGACTTTT-TATATGCCTTAGGCGTTTA 96921
Gallid               TGACC--ATGCCAGAGTAAATCGTCATTTCTATTATCGCCACACAAACGACAGCAATACA 102657
                     * *    **  **   **  * * * **  *   *       * *  *   **   *  *

Human                TAAGATGCATGTTTCTATC-CAAGATCTGTTTTTGCCGGCGTTCGTTTGCAATAGCAATA 96980
Gallid               TAATACGCCTCAACCCAACGTAATATCTGCAAT-ATCGGAGTT-ATTTACAGCCATTGCC 102715
                     *** * ** *    * * *  ** *****   *   *** ***  *** **

Human                ATTCAGTGGATTTACAGGGACTGGAAGATCAAGATGTTGTGAGAAATAGAAAGAAAAAGG 97040
Gallid               GAACACGGGA----CAGTATTTTTACTAGTAGCTAATTTCGATTTCCGTCAATTATCTAC 102771
                        **  ***    ***    *  *  *  *     **  **        **  *

Human                TGTATTGGATCACTAACTTTCCGT-GCATGATTTCTAATGCCAACAAAGTGAATGTGGGA 97099
Gallid               ATCATGAAACCACT--CTTACGATCGCACGAAACACAATACTA-CAGTCTGTATCCTGAC 102828
```

```
                   **   * ****  *** *  * *** **     *** * * **   ** **   *

Human           TGGTTTAAAGCAGGAACAGGTATTATTCCTCGGGTGTCTGGCAAAGACCTTCAAAATGTT 97159
Gallid          ACGCTGAACG---GATTACACAACGTCTTCCGGACCATCGGAAATTCTGTTCAATCTGAT 102885
                  * * ** *   **  *   *   *    ***      ** **     *****  ** *

Human           -TTGCTTCAGGAATTAAATAACGTTCGAGAGATTCCAGGGTTAGTCTTTGATATGGATTT 97218
Gallid          ACCGTTCGACGTCTTAACCTAGGCTATCTCGAT---GGGGACAACAGACGAGGTAATTTG 102942
                   * *  * *  ****   * * *     ***    ***  *      **  *   **

Human           GCA--TCAATTGCTTGTTTTGCTGGAACAGCGGAATCTACATCAGATTCCGTTTCTCGTT 97276
Gallid          GCGGGTGGATTGGAATTGTTGCGCGATGCAACCACTCCAAATGCATCCCGAATGAACATA 103002
                **   *  ****    * ****  **       * ** * **      *   *   * *

Human           AAACAGTTTCTT-ATTTTTTTACGTCTTGGCCTGTTA-ATGGGTTACGGGCACTCTCGGC 97334
Gallid          ACTCGCCCTCTCGATACCTCAACGTCTGGCGCAACTGCAATGATTAT--GCAATCTCTGC 103060
                *  *    ***  **   *  ****** *  *   *  *  * ***   *** **** **

Human           GTAACAAGGTGCATGATATCATGTTACACTTAATTTCGAATGGCCTTTTTGATTTTAATA 97394
Gallid          GTACAGATGTTGCCGAAAACAT----CACTTTACTTACA--GGCGAT----AGTCGAGCT 103110
                ***   * **    ** * ***    ***** * **  *  ***  *    * *  *

Human           AGAACTCCGTAGCAAAT-ACAAAAATCAAACACGGGTGTGCTTTGGTTGGGACGC--GGC 97451
Gallid          ACAATTTCCCGACAAGTTACCCTAACAGATTTCTGTTTCCCTGATGCCGAAATGCCAGGA 103170
                * ** * *    *** * **   **   *   * * *   **   *  *  * **  **

Human           TCGCCAACAATGTTCCGAAAATCATTGCTAGGCAGAAGAAAATGAAGC----TAGATCAC 97507
Gallid          TTGATAATATTATCTATTCGACACCCACTAGATATAA-ACAGTGAAGCCCTCTACGCTAC 103229
                * *  ** * * *       *      ****  * ** * * ******    **    **

Human           ATGGGACGAAATGCTAATTCGCTTGCCGTG----TTGCGTTTTA----TCGTTAAAAGTG 97559
Gallid          TCCAGCCGGAC--GTGATCCGCGAGTCATGGAAACTGTATGGTACGAATTATCGGAGTTG 103287
                    * ** *    * ** ***  * * **     **  *  **    *  *   *  **

Human           G--GGAACATAA--AAATAAAACTGTTTTCATTAAACTGTTGGAATATTTAGCGGAAACC 97615
Gallid          GCAGCAGTATCAGTAAATAGAGTAAACGGGAGTGGAGTCCGGCCCTCCTTAGTCTCTCTT 103347
                *  * *  ** *  ***** *         * *  * *   *   *  ****

Human           TCAACTGCCATAAATAC--GCGGAATGAAGTCGCCAGATTACTTCAGACTCTGACGACTA 97673
Gallid          TCATTTCTCATCGCTGCCCGCGCAAGCGACTACGCAGATAAGTGCGGGGCCGAAGCACTA 103407
                ***  *  ***   * *  *** **   * *   ***** * * * *   *  *  ****

Human           ATATGAAAACATGAATGTACCCATGGCCGACGAATGGTTCGATTGCGCGATTAGGTTAGA 97733
Gallid          AGAGCACATGTTATTAGTAATTATGGCCGACGGCGGATGGAA--GAAAAACTTGAT-AGA 103464
                * *  * *   *    ***   **********   * *   *  *    * * * * ***

Human           TTCGGAAACCATA-GCTGTCCATGA-AATTTTCAATTCGGATTTAAGTAAACTGCTTAAC 97791
Gallid          TTCGGAATTTGTTTAATCACCATGTTAAGGTGTCGCGTATTTCCACACAGACACTTTCAG 103524
                *******    *    *  *****  **  *          *  *   * **   ** *

Human           TTGCACTCGAAAACAGTCTATATG---TCCGATCTGTGCGCCTTTATTTCTGGTTGTGTT 97848
Gallid          CTAT-TGGGAGGACTGATTTCGTGGATTTCGCAACGCGAAATTGCAAGCATTACTGCCGT 103583
                 *      **  ** *  *   **   * **    * *    *  *    *   **   *

Human           AATCGGAATGTT---GGTAAAC-TTACCATATATTGGCACGT-GTACGGAGACATAATCT 97903
Gallid          GGTTAGAGGGCCACAGGAAAGCATCAAAACAGAGCAGACTGCCATGCCAAGGTCCTCCGT 103643
                  *  **  *     ** ** * * * *  * * *   *   *   * *  **        *

Human           ACGCATTGACGGGTAT-TTTACATTGT-----GTAAAAATAACGATAGAGTGCGGGGAGA 97957
Gallid          ATATGTTCCCGCATGTGCCTATATCGATTTTGATAAAGATATACGGGTAATTCACGAGGA 103703
                *    **  **  * *   ** ** *       **** ***       * * *  *  **

Human           GAATTGCCGATGGTCGATATAGATTACACGAAATTCCGAAATTATTTTTAATGAGAGGAC 98017
Gallid          AAGGTCTTCATCATCTCTATACCTTGTGTTCGTATATACACAAAAATTCGGGCGTGAAAC 103763
                 *  *    **  **  ****  **         *    *   *  **          **

Human           AGTCAACACCCCTGGAAC-TGAAGTGGAAGCACGCCGTGGGTATCGCGACGACGAATAAG 98076
Gallid          TGTTCGGATATATGTCATGCGAAGTCGTTTGGGGGAGTGGGCATTCCGAGAAGGGCTAGG 103823
```

```
                  **    *    **  *   ***** *      *  ***** **  ***  * *  ** *

Human            CC-----TTTGCTGACGCACGTTTTAACAGATGTGTTGGAAACCTC--TCCTTTTACCTT 98129
Gallid           TTACATGTATTCTGGAGTGCGGTCCAATAACGCTATTACTGGTGTGGATGGCCTGATAGT 103883
                        * * ***  *  ** *  ** *    * **       *   *    * *   *

Human            GCCAGATACG-CTTCTGTCGGTGCAGGAGTTGTCTATTTTCAGAGAGAGATTGTCGTATA 98188
Gallid           GCCACATGGGGCAAATGTAAATATGGAATTT--CCATTAACGAAAACACTTGATCTCCG- 103940
                 **** **  * *   ***   *   * * **  * ***  *  * * *  *  **

Human            TTTACTATGTGCTCGGGTTAGATGTTGATATCGTAGCGCG-GACAGAGAGGGAGATTTTT 98247
Gallid           -CAACCGCGATCGTAGGTTGGGTATTGCAGCCAGATCTAGAGATTTAAATAAAGCCGACT 103999
                    **   *  *   **** * * ***    *  * *  * **   * *   **     *

Human            CAAAAATGTGCAGAACTAGCTCGCCTACAACA-AGTCTTTCTTATTCAAGGAAATATTAT 98306
Gallid           GGAAAGTAGATCTACGTGGTCGTCCCACAAAAGAATCTTGCATGTATGCTGCA-TATTGT 104058
                   *** *      *  * *    ** **** * * **** * * *     * * **** *

Human            GGAAAACTTTGTCCTCGTTCAGGCGTGTCTATTTCAGCTGGGGGCT---GATGGTTTGTG 98363
Gallid           CGTTTGGGACATTTAGATGAAAGT---TCGGTACCAGTCAAAAAATTCGAACGTTGTGGA 104115
                  *         *     *  * *    **  *  ***        *    * * * **

Human            GGAAGAGATGTCTG-GCTCTGTACGTCCTAGGCCGGAGTTGATGTCTAG-TGCGTTCATT 98421
Gallid           TCCCTAGATATCCCCGTTATATGGATACCGGGTGTAATTTGGAATATAGGTACATGGATC 104175
                      **** **   * * * *   * *  **    * ***   * *** * * *  **

Human            CAACACAGAGTAATGGTGAATA---ATTGTTATTGTCTCGCTGTCATCTTCAAT---GCC 98475
Gallid           GAATGTT-ATTAATGATAAATATGCAATCTTGTTGATATATAACCATATTTAATAAAGGC 104234
                  **     * ***** * ****   * * ** ***         *** ** ***   * *

Human            ATTTATAAACACAAAGTTTCCCTGCCTACCGTAGAAAGAAGCCACGAAATCGTTCATCGT 98535
Gallid           GATCAGGCGTACGTTGTGCATAAATATGATTTGGGAATGATTCATGTG-TTGGTTGGGGA 104293
                   * *     **   **         *    * * **  *  ** *   * * *    *

Human            GTAGTTCAGGAATATTATAAGTCTTATGTGAATGCTCCTCTCTCTGTTCTTGTGTGTGCG 98595
Gallid           GGAGTTCAC-AATATAAAAGTCAGAGTCTGGCGGTGAGTTACTTTGGTCACGGTTCCGTT 104352
                 * ******  ***** * *       * **   *    *  ** ** **  *  *  *

Human            ACTAAGGTGCTTACCTTGT-TTACAGAAGAATATAACTTTCAGTCAGC--TCTCGTATTT 98652
Gallid           ACCGGCGCGCATTCGACAAATTATACGGTCATCCACTCTGCGACTAAAAATCTGCTATGG 104412
                 **    * ** * *      *** *     **  *   * *    *    ***  ***

Human            GTCAGTCAGTTTTTCCAGGTGGACGTCGAGGCTTCGAGAGCGGATGTGATTCGTCTGTTT 98712
Gallid           ACCATTCAGATGTCT---ACGAACCATATACTTTCTCAGGGAAACATCA--CGTTAGAAT 104467
                   ** **** * *       * **        ***    *   *  * *  ***  *  *

Human            TTAGCGTGTCTGAAGGGTGAT-TAGATCTCTCGGAAAGAGGCTGAACTGTTTCCAGAGCA 98771
Gallid           GTACGACTTCTCAAAATTAACACCGATGT---GGAATAAAGATGCATCTACCCCACCGCG 104524
                  **     *** **   * *    *** *   ****  * * ** *      ***  **

Human            CACATAAATCGCCATAATTATGGCGATAATTAAGTCGTCAGAACAGGCTTGTTTTTTA-- 98829
Gallid           TAAATTTGCCGATATGCATATAGCATCGACTGTAGCCATGGAATTGTATAACATTGAACG 104584
                  * **    **  **   *** **    * *    *    ***  *  *    **  *

Human            -GCGCTGTATGTTATGTAATTGTTAACGGAGATCTGGTGAATGTTTCTGATTTGTTCTAG 98888
Gallid           GGAACTTTTGAATATGGGCCACTTATCTGGTATGGAGACAATGATGGTCGACGTTCGAAA 104644
                  *  ** *    ****      *** * *  **   *  **** *  *      *   *

Human            TGCGTATTCTACGGGATCGTAGGTGATTTTTATTGTAAACGATATCAATTCCTGTGATGC 98948
Gallid           ATCTCCTGAAATGATTCCGTCGACAATTAATATGCGAGACCATATAACACGCCTAGTAAA 104704
                   *   *   * *    *** *   ***  ***   * ** **** *    *   *

Human            CTTGATGTTTCCTGAATTAAAATTCGAGATGAAAAACTCAACTGCT-------AGTTTTT 99001
Gallid           TAAGATGAAGCCTATCTGCCGATTTGA--TGAACGTCTTTACTCCCTCTGCGGAGAATTG 104762
                    ****   ***   *    *** **  ****   **  *** *        **  **

Human            TTTCTTTACCGAGTAGGTAAAATGGCTGTGCTATCTGATTCTGGTCTGGGGTGTGGAAAA 99061
Gallid           GTTCATTTGCGAATCGAATTACGCGAAGTTCCTCTAGATACATGGCTGC--TGTCAAAAA 104820
```

```
                 *** **  *** * *    *   *  ** *     *** *  * ***   ***  ****

Human           AAGTCACCTGTATAGATTTATTAGCCG-TGATGTTTTCCTTTATGATACATGCGATTTTT 99120
Gallid          AGTTAAACCTTAAGTATGAAGTTGTAGATAATATACGGCGCTACAGAGCACA----TATT 104876
                *  * * *  **   **  * * *  * * ** *    *  **     **      * **

Human           ACGGCAGAAGCTTGATTGGAATTCCCTTCTATGATGATTTTTACTTCCGTGAAGAAAGGA 99180
Gallid          GAGATGGA--------TATGCTTCGTTTTTATGGGAGTATCCATCCCTG-GCTGAAACGG 104927
                  *   **        *    ***  ** ****    * *  *   * * *  **** *

Human           TGTAGGTCTAGGATTGACAATATCATATGCGCGGCACATTCAGCTATTGCTGTGTCGGAA 99240
Gallid          T-TAGGATTACAATCAGCATTGAAATATGAAGAATATCTCGTAGAATTGGAAGATGGGAA 104986
                * ****  **  **   ** *   *****      *  *      ****     * ****

Human           --------CTCGTCATTAAACTTTCTAGAAAGTAGTGCTCCATGCCGTAGACGATATATT 99292
Gallid          AAAAGAATCCCTGTGTCAATTCTTCGTTCGATTGGCGGCGGCAGCTGCAACGGAGGCGTC 105046
                        * *    * **   ***     * * * *      ** * *   **    *

Human           GATCTAGATAGGTGCCTATCGCTGCTATGCCAGTGCCAGAAGCACGGCGGTTGCCTGTAT 99352
Gallid          GATG-AAAAAACCTTTTATGACCACCTTAACAACGGGGGTAGCAAATTGGAGAACTACAT 105105
                ***  * * *      ***  *  *  *  **  *   * ****    **    **  **

Human           AGGCAGGATCTAGGTATACGTACAGATCTTTACCTAAAAAAGGAATTAGATTTTTAT-TG 99411
Gallid          TCACAACTTTTTTTTTGGCACTC-GCTAACCAGCTATTTGTGCCGTCTACTCCTTGTATG 105164
                   **   * *   *   *   * * *    * ***     *   *    *  ** * **

Human           ATGGTGCTGTATCGGAAAAACTCGAATTCGGTTTGGCCTTGTTCCGTA-ATTAAAATGTC 99470
Gallid          CTTTTCCTAGGACGAGAAGGAACCTCTACTGCGAGCTGTTATCTTATGGATCCTAGAACT 105224
                 *  * **    **  **    *   * * *   *   ** *    *  **   *

Human           GTTGATCACATTGCAAGTGGCTCCACCCATGATTTCATGGATGAAAGCTC--CTTCTAGG 99528
Gallid          AATAAT-ACACAAGATGCACTTAAGGCAATTACCGAGGATGTAGTACCCCATCTTCTGGC 105283
                  * ** ***    * *    *    * ** *         *   * * *  ***** *

Human           AAGAGGTTGGCCGTTTTTTTAACTTCGGCGTTAATGCTGATGAACTTGGGTTTGTGGAGT 99588
Gallid          GAGAGGAGGGATAGGTATATCGTTGCAACATTTA-AATGGGAAATTTGGTCTTATGCACG 105342
                 *****  **     * * *   * *  * ** *   **   ** ****  ** ** *

Human           CGGTAGCAAGAACAGGCTGTGGCGTTACCGCGTTCGTTTAGCATATGGGCGTGATCTTCG 99648
Gallid          TTATGAAAGTATTGGATTCGTTAGTTATGGCGGCAAAT--GTAAATGAATCCCGGCC-CA 105399
                   *   *  *   *  *     ****  ***     *  * * ***        *  *

Human           CATACGTAAGAAACTACGGAGAGC-ATTTCAAACGGAGAGTTGTTCAGCTTCATTAAAAA 99707
Gallid          CAGGCATTTGCGTGTACCTGGAACCATGGCACGCAGATATTATGTCAGCTCTAAATATGC 105459
                **  * *  *    ***   ** * **  **  * ** * *   ******  *   *

Human           AGAAGTTGAATGGTTTCCGGAATTGGTCGAAGATATAAATAGGATCTTGGTAGATGCTTG 99767
Gallid          GTGGGATGG-TGGCAGCGGAGGAATCTCGCAGGTGTGA-TAATGTGTTTCTTG-CCCTCT 105516
                    * **  ***   * *       *** ** * * * **   * **  * *   **

Human           GGGCAGGAAACCTAAAATCGTGCTGAACGCGTCTTTCTTTATAAAGTGGCTCTCGT-CGA 99826
Gallid          GGTCATGTGATTTACTTTTTAAACGATATCTGAGACATGTAAACGGAGAGAAAAATGTAA 105576
                ** ** *  *  **   *      **   *       * ** *  * *       *   *

Human           CAATAAGTAGATTGAAGCTTTGTCCGCGTATACT--CTGAG----AGGGGAATGAATTCG 99880
Gallid          TATGGACCCTGTTTGACTCCCGCGCATCTATACTGACCAAGCTCCATAGTGAAGAATTCG 105636
                 *   *     **  *     *  *   ******  *  **    *  *  * *******

Human           GCGTTAAACGAGATAAAAGACGATTTTGATAACTGTGAAACG--AAAAACGACCTTTTTA 99938
Gallid          AAAAGGAATATGAACGTTTAGAATCAGAAGGCTTGGGTGTCGCTAGTATCCCCATTAGGG 105696
                      **   **      *  **    *    ** *   **  *  * *  * **

Human           AAATAATTGATAAAATAAGCAAAAATTGCAATTTTATAGTGGAACAGGTCGAGTCCTTGC 99998
Gallid          ACATGATGTTTGCAATAATCAAAAGT-GCAGCCTC----TACAGGAAGTCCATTTATTCT 105751
                * ** **   *  ***** ***** * ***   *     *  *  * *** * *  **

Human           CTCGGAGGGTGGATTCAGCGGCTATCCTATTTGATAATCTCGCGGTGGAGATATTTAACG 100058
Gallid          TTTCAAAGATGCGTGTAATAGACATTATATTACAAACACGCAAGGAGATGCTATAGCAGG 105811
```

```
                 *   * * **  *  *   *  **  ****  * *  * *  ** *  * ***   * *

Human            ATGTAATATATCGACAAAATGGAGATGGCGTTCCCGCGAAAATACGACAGGGTAACGGGC 100118
Gallid           GTCAAATTTATGTACCGAAATAATTCAGAAAACCGATGCTAACACAAATGGG--GTGTGC 105869
                 *  *** ***  **  **    *     *     **    *  ** ** *  ***     * **

Human            AGAATATTGACACATAAGAATAACCAGATGTGCACAACCGAATGTTCTCAGATGTATAAT 100178
Gallid           AGCTTGGCGA-GCATCAACCTTGCCAGATGTGTCCG-CAACATAGATGGAAACCGCCAAT 105927
                 **  *   **  *** *   *  *********  *  *   **       * *     ***

Human            TTA--CATAATCCTATCACGTTTGAGTTGGGACTTGGAAACGTGTTTGTCTGTATGCGGT 100236
Gallid           TTGATTTTGATGCTCTCAGATATGCTGTGAGACTAGCTACCGTTTTCGTT--AATGCCAT 105985
                 **      * ** ** ***  * **   ** **** *  * *** ** **    ****  *

Human            GTTTGACGGTTCACCACTGTGATATGCAAACTGACTGTACCATTGTCAACA-CGCATGAG 100295
Gallid           AATGGAAGGA--AGTGATGT-ACCGACGGAAAAATCTCATTCTGGCCGAGAACGTAACAG 106042
                   * ** **   *    *** *    *  *   *    *   * * * * * * ** *  **

Human            GGGTATGTCTGTGCGAAAACGGGTTTATTTTATAGCGGTTGGAT------GCCTACATAT 100349
Gallid           ATCGATGGGCATCGGCGTGCAGGGCTTTCATACAGCTTTTCTATCTATGGGTCTTGATTT 106102
                     ***    *  *    * **  * *  ** ***  **  **       * **  ** *

Human            GC-CGACTGTTTCTTAGAACCAATCTGTGAGCCGAATATCGAAACGGTTAATGTCGT-GG 100407
Gallid           ATGCGATGAACGCGCTAGATCCCTCAACAAGCTAATTTTTGAA----TTCATGTTATTGG 106158
                    ***       *     * *  **    ***  * * * ***    ** ****  * **

Human            TGGTATTGTTATCTTATGTATATAGTTTTCTGATGGAAAACAAGGAACGATATGCTGCCA 100467
Gallid           AGGCGATGACAG-TTAGTTGCGAATTCTGCGAACG--AGGCCTGCCGCCGTTTGCTGATT 106215
                  **   **  *  ***  *    * * * *  * *  *  *  *   *  * *****

Human            TTATTGATAGC-ATTATTAAAGATGGAAAATTTATAAAAA-ACGTGGAA-GACGCCGTGT 100524
Gallid           TCTCTAACAGTTATTATGCACGAGGACGTCTGCATTTCGATGGGTGGGCTAATGTAGAAT 106275
                 *   * * **  *****  * ** *     *  **    *   ****    * *  *  *

Human            TTTATACTTTTAATGCGGTTTTTACGAACTCAACTTTCAATAAGATTCCTTTGACGACGA 100584
Gallid           TGGCTGCAGTGGAAGAGTGGAATATGT-TACGAGATAGAATAGTGTCCGCTGGATTATAC 106334
                 *   * *  *  * * *     ** *    * *  *  ****   * *  * **  *

Human            TAAGT-CGGCTTTTTGTTCAGTTGATTATAGGAGGACACGCTAAAGGAACGATTTATGAC 100643
Gallid           AATGCACAGTTCATAGCTCTAATGCCTACAGCCGCGTCAGCGCAAG---TCACTGAAGTC 106391
                  * *  * * *  * * **   **  ** **  *     **  ***     * * * * *

Human            AGTAATGTAATTCGCGTCAGTCGTCGGAAACGAGAAGACAGTTTACTAAAAAAGATGAGA 100703
Gallid           AGTGA-GGGATTTTCACCCCTCTTCAGTAATATG-------TTTAGTAAAGTGACAAGTA 106443
                 *** * *  ***  *  *  ** ** * **   *       **** ****         *

Human            TTGGAGTATGGAAACGCACTTATACTATGACACCCTGTACCAATATCAAGGCGGAGTGTA 100763
Gallid           CGGGAGAATTGTTGCGCCCCAATATT----CAGTTAATGGAAGAATTAAGAACGA-TATA 106498
                   **** ** *   *** *  *** *    **      *   *  ** ***    ** * **

Human            TCCGGCCCATATTTGCCTGCCGACAGATGCGTATCTTCCGATGAGAGTGGATTGTATCGA 100823
Gallid           CTTAGATAATGAAAATCAACGATTGGCCACA-ATTGCCGCATTAGAATCCGCAAACTGGA 106557
                     *   **       *  *     *   *  **   *  ** *** *         * **

Human            GTCTTTATATTTTCGGTGTGTATTTTTCAAGAATGGGATGCATTATACTGAATGGAGTAA 100883
Gallid           ACATACAGACTGCTT-TAGGAAATAAACCCGAATGCCATTCCCTAC-TTAAATACA--AA 106613
                    *  * * *      *  * * *   *  *****  ** *  **   * ***  *  **

Human            ATTAAAGTTTACTGTGATTTCACGTGAAATAAAGTTTAAAGATGTGTTAAAGAA-----T 100938
Gallid           ACCGCCTTCGACTATGACCAAGCCCTCCTAATTGATCTATGCAGTGATAGGGCACCATTT 106673
                 *      *  *** ***     *       *  * *  * *  *** **  * *     *

Human            GCTGACTTTGACGAACTTTTTACCGGTTTGGTGGTAATGACTATTCCAATTCCGATAGTA 100998
Gallid           GTGGACCAGAGCCAGTCGATGACTCTGTTTATCACGGAAACCGCTGACGGTACGTTGTTG 106733
                 *  ***     * *     * **    **  *        **   *     * ** *  *

Human            GATTTTCATTT--TGATATCGATTCTGTAATTTTGAAATTGGTTTATCCGCAGTTAGTGC 101056
Gallid           GCATCACGCGTCATGAACCTGCTTCTACATGCATATAA--GGCTGGTCTCAAAACAGGG- 106790
```

```
                   *  *  *   *  ***    * ****  *    *  **  ** *  **   *   ** *

Human              ACCGAGAAATAGTGCTGAGGCTCTACGATCTCATATGCATCAGACCTTCGTCAGACCGGC 101116
Gallid             ---ATGTACTACTGCAAGATACGCAAGGCCACTAATGCGGGAGTATTCTGTGGGGACGGA 106847
                        * * ** ***         * *  * *  ****   **   *  **  *  ***

Human              CGTCGGAAGTATCAGCTAAAAATATTG----GTATTGATTTCTATCAACTAACTTCA-CA 101171
Gallid             GAATTGACCTGCTCGTCGTGCGTCTTGTAAGATCCTGACACGTGCACAGTAACCGCATCA 106907
                        **  *    *       * ***     *  ***    *    * ****  ** **

Human              GGGAAATAGACAAACACCCGATGAGGAAAAACGTTGTCTATTTTTTCAGCAGGGACCTTT 101231
Gallid             TTCTTCTAAACGCACAGAAAAT-ATGAGCGGCCCTCCGTCACATGAGATCGGCATGCTAT 106966
                         ** **  ***    ** * **    *  *   *    *   * * *    ** *

Human              AGAGCCACCCTCTACCGTCAGAGGCTTAAAGGCAACCGGCAATGCAAAGCCGATGCAGAT 101291
Gallid             ACAGCCTTGCTCCATC--TGTAGATATGAAGTCCGAAAACATTGAAGACTTGCCATCGGA 107024
                   * ****   *** * *     **   * *** *      ** ** * *   *     *

Human              TCCCGCTCATGTCAACGAAAA--AATGACCGAATCTTTTTAAGCGATAGTTGGTTCGAA 101349
Gallid             AAGCATTCCTACCACCAATCACTGCGCATCAAATTACTTCTATGCACCCGAATGTCCGGA 107084
                     *  ** *  ** * *  *      * * ***   ** ** **    *   ** ** *

Human              CAAAAGGTCAGATGCAAAAA---AATATTG-GATTTTACGCAAACGTATCGAGTCGTGGT 101405
Gallid             TATAGGA-CACCTTCGAGCGTTGAGTATTATGAATCGATGGACGGAGACCGAAT--TTGT 107141
                    * * *  **  * * *      * ****  ** *  * * *     * *** *  * **

Human              ATGTTGGTACGAGCTTT----CGTTTTCCCGCGAGATGCAGATCGAGAATAATT-TACTG 101460
Gallid             AATCAGTGACGATCTTAGAGACGTTTCAATGCTTACCGAGGAGGAAAGATATTTCTACCG 107201
                   *    *  **** ***     *****    **     *  **   *  *** ** *** *

Human              TCCGCTTCCCAGCTAAAGCGGGTTAACGCTGCGGATTT--TTGGGATAGAACTGATCGGT 101518
Gallid             ATTTCTTTTTACTTT--TTTATCTGCCGCTGACGATTTAGTTAATCTTAATATAGATAAC 107259
                      ***   *  *         *  *****  *****  **    *  *  *

Human              ATTTGCGAGATATTGGGAGCAGGGTATTGACACACATCGTGAAAACGCTTCAGATTCATA 101578
Gallid             CTTTTAGGCTTATTCAGTCAAAAAGATATACAT-CACTATTATTTGAACAAGAGTGTAT 107318
                    ***  *   ****  *   *    **  ***  **   * *    *    *** *

Human              ATAGGCAATTTAAACAGAAATTTAATTGCAATTTCCCAGTTAATTTCAGCTTTGAACATC 101638
Gallid             AGAAGCTGTCCATTCGCGAGCCTA--TAGTATAATCCAGTTAATGTTATTCGACAATGAC 107376
                   * * **  *  *  *   *   **  *   **   ********* * *      **   *

Human              T-ATTATCATTTATGCAGCTCGGGAAAGATTT-TTGGATTTTAAACTTAACTTTAGACAG 101696
Gallid             TCACTGGCACGAGCAGAATATGTTAAGACTTCCTTGAATTCCCCCGCTATCCAGTCTAAA 107436
                   * * *  **       *    *  **   **  *** ***       ** *       *

Human              CTGCATTATTAAGGCAATTATCTGTTTCCTAGGTTTTCGA-AACGGGAGAAAATCTTTTT 101755
Gallid             CTAGATTGGTTGAATAAACGTGTCATCGAGTGCAGCTCAATAGCCGAAAAATATATTCTT 107496
                   **  ***  *     **   * *  *     *    ** * * * * * ** ** ** **

Human              TAGCTCAAGATGAAGTTTGGGGAGATTTAATAGACTGTTCTAAAGGATCGGTGATCTATG 101815
Gallid             ATGATCTTAATAGAAGGCATTTTC-TTCTCCGCATCTTTCGCCGCCATCGCCTATCTACG 107555
                     * **   **  *           **       *   ***      ****   ***** *

Human              GGGAAAAGATCCAATGG--ATTTTAGACTCGACTAACAATTTA--TATTCGACGCGTCGT 101871
Gallid             GATCAACAATCTATTTGTAGTTACATGTCAAATTAATAATTTGATTAGCAGAGATGAAGC 107615
                   *   **  *** * * *   **  *       * *** *****   **   **   *  *

Human              GAAAAACA---GAATAAGTCGTGGGAATTATATG--TTGATTGCTGTGCTTTGTATGTAT 101926
Gallid             AATACACGTAGAAGCATCTTGCTGTATTTTTAAGAATTATCTCATGGGGCCCAAAC-CAT 107674
                    * * **     *  *  * *  * * ** ** *  **   *  ** *      *   **

Human              CTGAAAAGTTAGAGTTGGATTTTGTGCTACCCGGCGGTTTTGCAATCACCGGTAAATTCG 101986
Gallid             CT-ATCGGTCGTGTTCAGGAATTGTTTAAAGAAGCTGTCC--AAATCGAATGTGCATTTC 107731
                   ** *   **      *  *  ****   *    ** **     ****    **  ***

Human              CTCTTACTGATG-GCGATATCGACTTTTTCAATTGGCGATTTGGGTTATCT-TAG--AAA 102042
Gallid             TTCGCACAGCTGCACCACACAACAGCCAGTTATTGGATGTTGAAGCAATATGTAGTTACG 107791
```

```
                 **  ** * **  * * *            *****   **   *  ** * ***  *

Human           TTTTTTGCAGAATGAATGTAACTGGTTCAGGATTTGTAAAAAAACATTCTATCGCGAATA 102102
Gallid          TACGTTACAGCGCGGACA-GACTACTGAAAGAATTA-GATATGCCACCTATTTACAACGA 107849
                *   ** ***   * *    ***  *  * ** **   * *   **     *  * *  *

Human           TCGCAGCGTTGCGACATCGTCTCCTATATTTTCGTTGAAGAATAAGCCTAAGAAATATTG 102162
Gallid          ACCTAAACCTACAGCTGATTTTCCGCTAGCACTGATGA----CAGCTTCAAATAATACCA 107905
                 *  *    * *  *    * ***  **     * ***     *     **  ****

Human           CATGCATTGCGAGATGGTAGTGCTCAAGCGAAGTCATGAATTTATGTTCAGCCTTGCGGT 102222
Gallid          ACTTCTTTGAGAGA-AGAAATACT---GCATACTC-TGGAAGCGTGTCAAACGAT-CTTT 107959
                  * * *** ****  * * * **   **  * ** ** *    ***  * *  * *  *

Human           AAACGGCATACATTTTGGACAGTTTTTGACCGGAATAATGAAATTTAAGAAGAAACAAGT 102282
Gallid          AATTTGTCTAGATTTTCATCATGT------CAATATAACAATATTAATAAAATTATCAGT 108013
                **   *  ** *****   **  *      *   ****  * *** *  **   *  ***

Human           TGCGGAAGGGCTCTGTTACTA-TGTATTGGAATTGGGAAGCATAAGCCCTGTGGATTTGA 102341
Gallid          CATTAGATCGTGTTGTCTTTAATGCATCGTTCCCCCAATACTTCAACAATATATGATTAA 108073
                      *  *  ***    ** ** ** *        *  * * * *  * *    ** *

Human           G----CTTTATCCCAAAATATAATTCTGACTGTGTTACAAGCATGCATTGTGTTACACCG 102397
Gallid          GAGGGCGTACCTCCGGTATACGTTTTATAAATTGGGATACGATAGTGCTAGCTTCATCTA 108133
                *    * *    **   ***   **   *   **  * * *   *   *   **   *

Human           GAGCTTATTTATGAAAATTGCTCTATTGTGTGTC--CCGAAGAGGCAAGTCGC-CTCACA 102454
Gallid          CATCAAATATGTTGCAAAAGATCAAGTCTTCAACTGCTGTCTCATCACGTCCCTCTTGCA 108193
                 * *  ** * *   **  * ** * * *     * * *      ** *** * **  **

Human           GTAAAAGGGCCCGGGGACAATAAATTGATTCCTTTAGGTGGGTGTGGAGTATGGTGTCTG 102514
Gallid          TTATAGGGA----AGCATTTCAAAATACTAACCCT-GGAAGTTCTGCGCTCTGGGATTAT 108248
                 ** * **      * *    *** *  *  *  * **  * * **   * ***  *

Human           AAAAATGGTGGCGATCT--GTATATCTATGCTTTCGTACTCGTTTACGATCTTTACGTAG 102572
Gallid          AGTAGCAATGACATGCTTAATAAATTTATACTCTAGAATTT-CACATCTTGTTCGAGGGG 108307
                *  *    ** *   **   ** ** *** ** * * * *     *   * **   *  *

Human           CTTGTTATGACAAAACCATCTTTC---CATCTCTGGCAAAAATTGTTTTTG----ATATG 102625
Gallid          GGCGTAAAGATTTAGCGCTTTTTGTACACCTCTTATAAGGTTAGTATCCGTGTAGTTGG 108367
                   ** * **    * *   ***    ** ****   **   * ** *  *     *  *

Human           ATAGCTTGCGATTCCGAAGATTGCGTCTTTTGTAAAGATCACAACAAACATGTATCGCAA 102685
Gallid          AAACATCATTATCCCAACAACT-CGTTTTTGATT--GATCTTTGCGAATATGAAGATCAA 108424
                * *  *    ** ** *  * * *** ***  *   ****    * ** *** *   ***

Human           GCCGGACATATTG-TAGGGTGCGTCTCTAATCAAGAAACCT-GTTTTTGCTACACAC--- 102740
Gallid          AACCTTCAGAATGGTAAGAGTTTTCCCCAGTCCGCCGATCTTGCTCTTCGTATAGAGGTC 108484
                  *   ** * ** ** *     ** * * **     * ** * * **  ** * *

Human           ---CGTGTCAAAAAAAAAATGACTGATATTA-ACAATCCGGAGT-TAATCTCTTTGCTCTG 102795
Gallid          TGATACACCTAGGAACATCGTCGTCAGTTGCACAATCCGTATCGTGCAAACATCGTTTTG 108544
                        * *  ** *  * *     **  ******** *   *     * * * * **

Human           TGATCAGGAAATTAATAAG-------ATAGATATTA-TGTATCCCGAGAAAAAAGCACCG 102847
Gallid          CTGACGAAAGATTAATTATTTCTTGCATTGATTTAGTGTAGGCTCGCGATGTAAATCCG 108604
                    *   * ****** *        ** *** *** ****  *     *   *   ***

Human           TTATCACTTGACATTAATTCTTACGTTCATGGGTACCTCGGCGACGAGCCTTGTGCGTTA 102907
Gallid          T-ATGACAGCGAACAAAGTTTAACAAAAAAGTGAAA-TAGTCCATTTGCAGAGC-TGCTA 108661
                * ** **     *  ** * * **    * * * *  * * * *   **   *   * **

Human           AAATGTGTTAATTGGATGCCAATCAGGATCAGCTCAGCTCTGAGCAGGCTGATTATTTTG 102967
Gallid          AAACATCTCTGCAATAAATAGTT---GGCGGGAATATCCGTGTG-AGATCGATAATAATA 108717
                ***  * *       *      *   *    *   * *  ** * **   *** **  *

Human           TCATGTCCTGTGTGTA---AGCGTGTGGTAATGGAC--TAAGTGTGCGTTATTTTCTGTA 103022
Gallid          TCACATCCCATTAATATCAAATCTGTGTCCGAAGACAATACATATGCGACCGTTTTTGTA 108777
```

```
                ***  ***  *   **   *   ****      ***  **  * ****    *** ****

Human           TTAATTTTTTGTTTCTGAAAATAAAAT-TGAATTGATAGTACTTACGTGTGTATTGTAGC 103081
Gallid          TGAAACA--GGTTCGCACAGATATCATCTGCTTCCATTGTACCGGCGTCCACATAAGGAT 108835
                * **      ***     * ***  ** **  *  ** ****   ***    **

Human           AGCTGGCGAAAAGTGCTGTGCTCTTTATATTTTGATGGTCGATTGTAATTACATTATCCA 103141
Gallid          ATCCCATATAACGGATTATACTC---ATGCAAAGTTGATAAAGTATTTTCGGGGTTTCAA 108892
                * *      ** *   * * ***   **     * ** *  * * *  *     * ** *

Human           GGCATGTGATTGTCTTTTCTGGAAACATTCGGCGGCATTTAAACTCGACTTCTTTCATCA 103201
Gallid          ACCTT----TCGCTCCATCTACAAACCTTCTTCC-CGGGGTGTTTCCTCTTACACCGCGC 108947
                  * *    * *     ***  **** ***  *  *        **  ***    *

Human           CAAAGTGAGATACATGTTTTTG-ATGTGCTACGTAACCG---ATGCTGATTCCTTCAATG 103257
Gallid          CTCTCCAACATCCGTACTTCCACATTCATCAAACAATCGTGCATGTAGTCGCCCAGAACC 109007
                *      * ** * *  **    **     *   ** **   ***  *   **   **

Human           TTTTTCAATAAAAAACTTATAATTGGA-ACTATGAACC--ACGTTTTTCCATGTCTTCTT 103314
Gallid          TCCTCTATTAGCAA---TGCAAGTGGCCGCTATCGCTTTAGCGCCGTATAGTGTTTTTCC 109064
                *  *  * **  **   *  ** ***   ****        **   *    *** **

Human           GGGACTAGGAATACGTTAGTTTTTTGTTTCAGCGTATTCAGAGTACTTTCGTTTACGAAT 103374
Gallid          ATTCCCATAAAATCCCCGATC---TGCTACAAAGACCGGATAGTATGATCGCTGGTGTAA 109121
                    * *  **  *     *    ** * **  *     * ****   *** *   * *

Human           TCAATGTCGAACACG---TGGGTCAGATAGT-TGATAACACGATTGGCTAAAGCCGGAAG 103430
Gallid          CAAACGCAGAAGACAATATAAGCACCGCAGTGTAATTAT-CGAATCGTCGACTCCGTCGA 109180
                  ** *  *** **    *  *      *** * ** *  *** * *   *  ***

Human           TTT-----GGTGACGGCGATGAAAAAGATTACATGTATGAGAAT--GTTCTTCTGAAATG 103483
Gallid          TCTTTCCCGGATATAGTTTTTCTAACAATC-CATACATAACATTCCATAAGTCTACGGCA 109239
                * *     **  *  *   *   **  **  ***  ** * * *   *   ***

Human           GTTCCAGTTGTATTTTGCGCGTGTCTCCAAACTCGTTGAAT---TCTCCTTTGATCCATT 103540
Gallid          ATAGGTGTCATATCCCCCCCGGATATA-AAGCCCGATATATGACTTCTCACTAACCCGTT 109298
                 *    **  ***    * **  * *  ** * ** *  **   *   *  * * ** **

Human           TTC---TGAAATCTTTGATAAAACCATCTATTTGCAAGAATAGCGGTTCACGATATAGAA 103597
Gallid          TGCATATGCAAAATTC-ATACATCCATATACTCCCATGATGACC--CTCGCAGTTTTTAA 109355
                * *   ** **  **  *** * **** ** *  ** **  * *   ** *  * *  **

Human           TCTTAAAGGATTCGAGAAACTCTGTGTATTGTGATT-CAAAGGATTTGTCGGAAGCGGGT 103656
Gallid          ATATAACCTAAATAGAAACCTGAAATTATCACAAGTACTACAGTTCTGTCAGATATATCC 109415
                   ***   *      ** **     ***    * * * *  * * **** **

Human           AAGAACTTCATTTCTTGTAGCTTGCGCGTAAGGCTCGGTAGTATAGTAAGAGGCTTACGG 103716
Gallid          TAACTCCTAATAACAAAGTACACGTGAAATAGGCCTAACAGTGAAACGATATACTCGGTT 109475
                 *   * * **  *      *  * *    ****     ***  *   * *  **

Human           TTT---TTAAGATGTCGCT--GTTTTTCAC------AGAAT---GTATATAGAGGTTTTA 103762
Gallid          ACTACATTAAAATATTAATCGGAATTACACTTCTGAAGGATCTGGTTCATGTAAATCTTA 109535
                  *   **** ** *   *  * ** ***      ** **   **  **  *  * ***

Human           CCTGTTGGTTATATGAATGGGCTAACCCGA-GTTCTGGCGTGAGCATTATAAAACGCTTC 103821
Gallid          ACCATTCTATATAGCAATATACGGATTCTATATTGTCTTATAAATAA-GTAAATTGCATG 109594
                 *  **   **** ***   *  *  * *  ** *    * *  *   ****  ** *

Human           TTATAGAAGATGG------CACTGTTGGGGTATTTGGTAGATATCGTAGAACAGTTTCTT 103875
Gallid          TTTTAACCTATAATTAACTCGTTATAGGTAGACATGACTCATACTAGGGTTGATTTGCAC 109654
                ** **    **        *  * * **   *  **    ***     *   * ** *

Human           -TCGCCTTTCCATATG--ATCGCTTCATAGTTGTTT--TTTATATGGGTTATGTCGCAGG 103930
Gallid          ATAGCTTAGCCAGATGCAGTAAATATATTGTCACGTGGCTAAAACGTGAGTAGTAACTTA 109714
                 * ** *  *** ***   *   *  ** **    *   * * * * *    **  *

Human           TACGTAGCATGGAGCCCG-ACCTTACGCTGGCGGCGGTCTATCAGGCGGCGGCGAACCTC 103989
Gallid          TATGGCGAATTAAACTTATATCCAATGGCGTTCGCGGACCTTCACACGGAAACATGTGTG 109774
```

```
                 ** *  * **  * *    * *  * *  *   **** *  ***  ***   *     *

Human            ACAGAGCAGGATAAGGAGATTTTTTCCG--AAGCG---GTAAAAACTGCGTTTTCAGTGT 104044
Gallid           GTGCGACAT--TCAAGTGGTTATGAGCGTCAGTTGAACGTAGAACATGCGCATTGCAATT 109832
                       **   * * * * ** *   **  *   *   *** **  ****  **     *

Human            GTAGTTCGGCAGCCCCGAGCGC-----TAGGTTGAGAATGATCGAAACGCCTACACAGAA 104099
Gallid           ATGTCCCAGAATCCTCCCCCATATAAATTCGTCTACTGAAATTCAAGAGCACTTGCTGTA 109892
                  *    * * * ** *   *       *  **  *     **  **  **     * * *

Human            TTTTATGT-TTGTGACGAGCGTTATTCCTT-CGGGTGTGCCGTCAGGTGAAAAAAAAACA 104157
Gallid           TTACTTCCACTTCGACGAACAACACACAAGACCAGTCAGCCG-CGAACGGAAATAACGCC 109951
                 **   *    *  ***** *   *  *    *  **  **** *    * *** **  *

Human            AAGTTAAATATCGATGCCGCTCTGGATAATTTGGCTTTGTCGTTTGCGAACAAAAAATCA 104217
Gallid           GAATCGAGCTCCT---CTAATTCCGATAACCGGCTGTTGTCAATGGC-----AGGAATAA 110003
                  * *  *    *    *   *   *****   *   *****  * **     *  *** *

Human            AAAAAGATGGCTAGAACGTATTTGCTG-CAGAACGTTTCGCGGACTCAAGATCAACAAGT 104276
Gallid           CTATGGGCAGCGAACACATGTATGATGATACAACGTTCC--CAACAAACGATC------C 110055
                   *  *   ** *  ** * * ** **  * ****** *    **  * ****

Human            TGCCATTTCGGGGACGTACATTTTATATACAAAAAAACACATTGAAACGTCTTTGATGCT 104336
Gallid           TGAGTCTTCATGGAAAATTGTTTTGGCTGGAGAGAAATTTATGACAGCATCGGCTGCATT 110115
                 **    *** ***       ****   *  * * ***   **   * * **        *

Human            CGATAAGACG--AAGTTAGTTAAACAAATTCTTGAGTATGCCGAG-ACCCCTAATCTGTT 104393
Gallid           AAAAACAATAGTAGGTTGCGTGAAGAACCCATTAATTACATTTAGCGACGACGGACTGAT 110175
                   * *  *    * ***   * ** **    ** * **     **   *      *** *

Human            AGGGTATACCGATGTGCGTGATCT-TGAATGTTTACTTTGGTTGGTGTTCTGTGGCCCTA 104452
Gallid           GATACAGGGCACGGTTTGCGGTCAACGTATGTTTGTTCCTATCGAT----TGTACTTCAT 110231
                      *   *   **  * * **   * ******  *    * * *    ***    *

Human            AAAGTTTTTGCCAGTCAGACAGTTGTTTCGGATACAGTAAGACGGGATATAATGCCGCAT 104512
Gallid           TCAGTGAATATGAATGGCGCGGGC----CCACAGCAATATTCCTAGCTCTTAC----CGA 110283
                   ***   *   * *    * *      *     ** **   *  * * * *     *

Human            TTCCGAATCTATTGCCTCCGTATCTGTACGAATGCGGCCAGAA-----TAATGGACTGTT 104567
Gallid           TTCTAGACGTACCCTCTTAG-ATGCATTCAAATGCGATAAGAAAAAAGTAGTAGAAGTTT 110342
                 ***   *  **    **  * **   * * ******   ****     ** * **   **

Human            TTTTGGCATTGTGCA---AGCTTACGTGTTTTCTTGGTACTCAGATTTTGATTTTTC--A 104622
Gallid           GTTTTACCTTTCGAGGAGAACCACCATGCCGACATCTAACACAAACCGTCACATATGCAA 110402
                  ***  * **  *     * *   * **    * *   ** ** *   * *  * *   *

Human            GCGCTAGAG----ATTTCAGAACGCGCTCGTCGT---CGAATCAGGTCACTCCTGTACGA 104675
Gallid           ATGATGGATGCTCATTTTCGAGTACAATCGTCAAATATGAATTATGGAGCGCATCCATCA 110462
                   * * **     ****  **   *  *****      **** * *   * * *  *  *

Human            CTTAAAACAGAAGTTTTCGGAGCAAGAAATTTCTGTTTTACCGGTAGCGTCACAGATGTG 104735
Gallid           TTTGTCCTCAAAAAACTCCAGATGCTACATTTTCATTAAACAAACAACAATTGAGCAAAA 110522
                  **        **    **        * ****   **  **    * *     **

Human            TATCTTCTGTGCATTATATAAACAAAACAAACTTAGTCTAGAATACGTTTCTGGTGACTT 104795
Gallid           TTCTAACTGTA-GCTGCAAAAGTACAACATG--AAGAATTGATCTTTGCTTTGAAAGCTG 110579
                 *     ****    *  * **  * ****     **  * **       * **    **

Human            AAAGA--CTTCTGTTTTTAGTCCAATTATAAT---AAAGGATTGTTTATGCGTGCAGACG 104850
Gallid           AAGGAGGTTTTTATGCCGGAACGATTTGTGATGTGATAAGTTTTGATATAGATGGAAGCG 110639
                 ** **   ** * *       * * ** * **  * * * **    ***   ** *  **

Human            ACAATTTCTACAACTCAGATGCTGCCAGGTACAAAGAGC--TCAGCGATATTTCCGGTAT 104908
Gallid           CAATGGTCCAATATCCCTATAATGCAACAAGTCATGCTTCGTCAGCCCTCATCGTGGCAT 110699
                   *   ** *  *  *  **  *** *      * *     *****  *  *   ** **

Human            ATGACCTTCGTAAGCTGCTCA---GTGCACTTGTCATTTCGGAAGGTAGCGTCAGATTCG 104965
Gallid           GTGGGAAGAAAAAAACAAATAAAAGTATAGCTGTAACTGCATACGGCAGCGGGAAACCTT 110759
```

```
                 **        **       *   **  *  *** * * *  * ** ****  * *

Human           ATATATAATGTCTTTGAAGG-ACTATCTGAGACAGTCAATTTCT---AAAGATTTGGAGG 105021
Gallid          -TCTGCCTTGCACTGGAAGATACTAACGCATTTAGAAATGTCGTGCAAAAAATTAAAACG 110818
                 * *    **   * ****  **** *  *    **  *  *  *    *** ***    * *

Human           TGAGACATCGAGATTCTTTAAAGATT--AGATTAGGGGAGAGACATC--CGTTGAGTGTG 105077
Gallid          GGAGCCGCTGGGGCGGATTTGGGATTTTATACAACGTGTGATCCACCGATGCTGTGTGTA 110878
                 *** *   * *     **   ****  * *  * * * * **  ** *   * ** ****

Human           CATCAGCATATGATCGCCGCTAGGCAGATCATTAAATCGGACAATGCGGA----ACACCA 105133
Gallid          CGTCCGCACGTGTTTGGAAGTCCCACGGCATTCCTGTTTTGCAATTCAGACTGTATGTCA 110938
                * ** ***  ** * *    *     *     *    *    **** * **    *   **

Human           GCATGTCA--TATCTTCTTTGAGTG-GTTTTTTGGAT-----AAGCAGAAGAGTTTT--T 105183
Gallid          ATATACGAATTGGAAGAAGTGAGTGCAGTATCTGGAGCAATAAAGTCGAAACGCATCAGC 110998
                  **   *  *        ******   * * ****       ***  ***  *  *

Human           TAAAAGTGCAACAAAGAGCTTTAAAGCAGCTAGAGAAATTGGACGTCGATGAAATAATTG 105243
Gallid          GAATATTTCCCCAAAGTATCAAATATCGGCTCCCGGAAACGGGGACCATCTTCACCCCCC 111058
                 ** * * *  *****      * * * ***   * **  **     *       *

Human           ATACGGCGGCGGAAGTGAAAGCGGTCAGTAATAATA-TAAAAGAAACTCTTATGGCAAGC 105302
Gallid          TTCGAACG--AGAAGGGAAACTTGCCAAAGTTATCAACCAATGAGACTTTCGTGAGGACC 111116
                 *    **   **** ****   * **    **  *   ** ** *** *  **   * *

Human           ACTGAATTAGAATAATTATGGACAACGGTGTGGAGACACCTCAAGGTCAAAAAACTCAGC 105362
Gallid          TGTAAGTATG--TCATGGAGTGGGAGGGTTCATTTATATTGCATG--TAAGCCTTATAGA 111172
                  * * *  *  * **   *    * ***       * *   ** *   **        **

Human           CGATAAATTTGCCAC-CAGACAGGAAAAGGTTGAGAAAACATGACGGACTTGGAAAAGGT 105421
Gallid          GGATACACCAGAAACTCATAGTTGTGCCAATTCAAACGACACCGTTCATCTGAACTACAA 111232
                 **** *   *  ** ** *   *      ** * *  ***      *  ** *  *

Human           GTTAAACGAAAACTTTTTGCCGAAGATA-GTTCTCCGTTAAAGAAACAGATCCCCGCCTG 105480
Gallid          AACAGAATACCGCTATCAAATTGGAACATGGATTCTGTCAA--CAACTCATCATTACCT- 111289
                   * *  *   ** *          * * *   ** ** **   ***  ***    ***

Human           CAGCGATATGGAAACACTTTCTTCGCCTGTAAAGTTTGGATGCAAGTCGCGAAGTGCTTC 105540
Gallid          CCGTCTTATACAACCACTGGTAGAACATATGGACATTGTCTGCAAATGCTCACATGCCTC 111349
                * *   ***  ** ****       * * *  *  ***  ***** *    *  *** **

Human           TGCTCTCGATGAA-AGTTTCGGA--AAATGTAAACACGAAACTGCTTGCGATTGTTCTGC 105597
Gallid          GAGCCACCGTGTACAGCAACAAATGGAAACGGAATATCAAACAATCGATGTCTAAAATGT 111409
                    * *  ** * **   *  *   **    ** *  ****       *  *    **

Human           GATAGAGGAATTGCTTTGTCACGAGTCGCTTTTAGA-----CTCGCCGATGAAACTGTCG 105652
Gallid          ATCGTAGTAACCATGTGTTCGATATTCTCCATTGCAGCTCATTTGGCTATCACCCTGTC- 111468
                     ** **      *  **   * ** *  **  *       * * * ** *  *****

Human           AATGCCCACACCATGTTCAGCTCAGACAAATGGAAACTGGAGCTAGAGAAAATTATAGCT 105712
Gallid          -ATGTATAACCTTGATTCAATTTATTGACCAAAAAAATT--ATCTATATAAA-CTGTACTA 111524
                 ***   *  *    ****  * *   *      *** *  * *** * ***  * **

Human           TCAAAGCAGATATTTCTAGACATGAGCGAGAATGTTGAACTTGTGGCCTACGGCGAGACT 105772
Gallid          TTTATGC---TATCACCGGATTTC--TAATTGCCTTCATCGTGCGTCTTACGATAAAATC 111579
                *  * **   ***  *  **  *     *     ** * * ** * * ****    * *

Human           TTGTGTAACCTGAGAATTTTCGAAAAGATCAGCTCG--CCGTTTTTGTTTGA----CGTG 105826
Gallid          GTCAGAAGTGCTGACATCAATTGGCAAACCGGCACAATTTATATTTGCTTTAATCTCATC 111639
                 *  * *        **         * * * ** *       * **** ** *    * *

Human           CAAAGCGAGGAGCGTTCGTATTCAGTGGTTTACGTCCCTCACAACAAAGAACTTTGTGGA 105886
Gallid          CATAGC-AGATACGCTTATTACAAGAAATATGT-TAATTGACAGTAATCCATCTTATGTA 111697
                ** *** **   ** *  *    **   * *   *   * ***  **   *  ** ** *

Human           CAGTTTTGTCAACCTGAAAAGACTATGGCTCGAGTTCTCGGAGTGGGTGCCTACGGGAAG 105946
Gallid          AAAATAT-------TGAGAGCAATAGAGATGACATCTTTGATGTGCTTTGTCATGCTTGG 111750
```

```
                 *  * *       *** *  * **  * *    *  * *  ***  *    * *    *

Human           GTGTTTGATATAGATAAAGTGGCCATAAAGACGGCCAACGAAGACGAGAGTGTCATTTCG 106006
Gallid          AGCATTCAT-TGCATCCTACCACTATGTCTGC--TTGGCAACGTCTGGAGATTTAACTTG 111807
                    ** ** *  **       * **     *      * * * *  ***  * *  * *

Human           GCTTTCATAGCCGGTGTCATCCGT-GCAAAATCGGGAGCCGACTTATTATCTCACGACTG 106065
Gallid          GAAAGCTGGGTTTTTGATATTGACCGCCGGAACAATTATCGGAATATCAGCTCCATATGG 111867
                *    *   *    **  **     **   * *      **   *** * ***   *  *

Human           TGTTATTAATAACCT--TCTGATTTCAAATTCCGT---TTGTATGGATCATAAAGTGTCT 106120
Gallid          AAACATTTCCTCCCTATTCGGATTTCTATTTCTATATACTATATTAGCCATAAACGTTGT 111927
                    ***     ***  ** ****** * ***  *    * ***    ******   * *

Human           TTGTCACGTACTTATGATGTTGATCTCTATAAGTTCGAAGATTGGGATGTCAGGAATGTA 106180
Gallid          AAGGGATGCAAGTAAAGCACTGATGAATACATGCT-----ATTATCGCATTTGTCGTGCA 111982
                  *  * * *  **      ****   ** * * *     ***      *  *   ** *

Human           ATGAATTATTACAGTGTGTTTTGTAAGTTAGCTGATGCTGTAAGGTTTCTAAATCTGAAA 106240
Gallid          ACGACTC--TACGCCATCCCTCTCGCCTCGGCTGCGGTCGTATG--TCCTCGACTCAAGA 112038
                * ** *   ***    *   *      *  ****  *  *** *  * **   *    * *

Human           TGTAGAATTAAT-CATTTCGA---TATCTCACCTATGAATATCTTTATAAATCA------ 106290
Gallid          TGTCAATGCAACGCATGAAGAAGCCATATCAAGCGCAGATACGATTGATGGTCAGATTCC 112098
                ***  *   **  ***   **    ** ***       ***   **     ***

Human           TAAAAAAGAGATAATCTTTGATGC---CGTGTTGG---CGG--ATTACAGCTTGTCCGAA 106342
Gallid          TATGGTAGTTATGAGCCACGCGACAGGCGTATTAATTCCAGTTGTTATTGCCTTGCAGAG 112158
                **    **  ** * *   *   *   *** **     * *   ***  ** *  * **

Human           ATACATCCCGAGTATAACGGCACGTGTGCTATTGCTAAAGAGTATGACAGAAATCTTCAA 106402
Gallid          GTACATGACAAAGAAGACTGTTAGTTTGACATCGACTGATATGTTACAGGGAGTCTGTGG 112218
                 *****  * *  *  ** *   ** **  ** *    * *   *    * * ***

Human           CTTGT--------GCCAATCAGTCGTAACAAATTCTGTGACAT---------GTTTAATC 106445
Gallid          CGTTTTAGTGGGGGCGAGTGTTTCAATATTTATCCCGTCACGTCGCGACGAAAGTTTATC 112278
                * * *        ** * *   **   *   ** * ** ** *           ** ***

Human           CTGGATTTCGACCACTT-GTCGCCAATGCAATGA-TATTGGTCAATGTATGCGAGGCTTT 106503
Gallid          CCGTCCAATTATCATTTTATTGTCTATAATAGGAGCAATGGCCATTACTTTGGCAGGTTT 112338
                * *       * ** **  * * * **   * **  * *** ** *   *  *  * ***

Human           TGATGGTGAAAATAATCCTCTTAGACACTGTA-ATTTGGATCTGTGCGCCTTTGCTCAGG 106562
Gallid          TGGTTTGGTACTCGGGCCAACTTTATTTTCCGCATGTGCAGCAGCTTTGTCATGTTATAC 112398
                ** *   * *      **   *  *   *    ** ** * * *        ** *

Human           TCGTATTATTGTGTGTCCTGAGAATGACAGA--TAAAC-----GCGGATGCC-GCGAAGC 106614
Gallid          CTGCATTAATATAAGGAATGCAAATAAGGGAATTAAACAATTAGCAGCTGCCTATGTAGT 112458
                  * **** * *  *   **  *** *  **  *****     ** * ****   * **

Human           TCAG-CTATACTACGA--------GAAAAGGTTGTTTGC--GTTGGCTAACGAGGCC-TG 106662
Gallid          GAAATCTATACTGGGATTTATCATAACTAGTTTACTTGTTTGTATATTAGTAGCGCTATC 112518
                  *  *******  **         *  ** **  ***   **    **     **  *

Human           TCGATTGAATCCTCTTAGATATCCATTTGCTTACAGGGATGCTTGCTGTAAAGTATTGGC 106722
Gallid          TTGACCAAATCGTTGTTCACATC---TTGGCCATATACGTATTGATCGTT--GTTTCGAA 112573
                * **   **** *  *  * ***   ***   * *    *  *    **   ** * *

Human           TGAGCATGTAGTGTTGCTAGGGTTATTGTTTTACCGAGACGTGGTTGATATATATGAAAA 106782
Gallid          CC-GCGAATAAAACTTTCATACATACTAAACGATGGAGTTGTGTTTTATGAGCGTTGAAA 112632
                   **   **    *   *    ** *     *  ***  *** ** **     *  ***

Human           A---ATATACGATT--TTCTAGATGAAAGAGGGGAATTTGGGTTACGAGACCTGTTTGAG 106837
Gallid          ACAAAGGTACCATCGGTTTAAAACTAAGTTGCATATCGTAATCCACAAAAATCATTTTAT 112692
                *   *  *** **   **  * *  **   *   *   *     ** * *    *** *

Human           GCAACTTTTTTAAATAATAGTAAACTTA-----CCAGACGTCAGCCAATCAGAGGAGGTC 106892
Gallid          ACATCATCCCGAAGAGACACCAAACGTAACCCTCTACATATCTTCCCTCATGCTCACGCC 112752
```

```
                 ** * *    **   * *  **** **     * * *  **  **     *   * * *

Human           TTGCGTCTCTACAGTCGTCCGAGTA---TGGAGAAAAACTTTTACATG-ACCTTAGAGCG 106948
Gallid          GCGTGTGT-TACGAGCTTTGGGGTGGACTGGACTCTTTTTTTTGCTTTTATCTCCGAGCA 112811
                  * ** * ***   * *  * **    ****       **** * *  * **  ****

Human           TTGTTCTTGATCACTTCTTCTGC--AGATCTGGATAAAGATACATCATCTCTCTTTCAG- 107005
Gallid          ACGTCCTAGGAGCCAGCCTTAGCCGGGATCTCGAAACACCCCCATTTCTATCCTTTGATC 112871
                  ** ** *    *  * *  **   ***** ** * *    ***       **** *

Human           -ATGTGATATAGTAATGGATCTTAATCAGATATCTGAAACACTAAGTGCCGTGGCAGAAG 107064
Gallid          CATCCAACATTTCAATTAACGGCGCGCCTTTAACTGAGGTACCTCATGCACCTTCCACAG 112931
                 **   * **   ***  *       *   ** ****   **    ***     *   **

Human           AAGAGCCTTTAACCATGTTTTTACTTGATAAACTGTACGCCATACGGGAAAAGAT---CA 107121
Gallid          AAAGTGTGTCAACAAATTCGGAAAGTACCAATGAACATACCATAACAGAAACGACGGGCA 112991
                **      * *** *  *    *  *   **     *  *****   **** **    **

Human           AGCAAGT-TCCATTTTCGATTGTTCGCTTGTGT-CATGTTTACTGCATGCTAATAAAATA 107179
Gallid          AGAACGCATACATCCACAACAATGCGTCTACGGACAAGCAAAATGCGAACGACACTCATA 113051
                ** * *  * ***   * *   * **  *  *  ** *   * ***   * *     ***

Human           TAACGCTTCTAACAATAATTGCATTTTGGG------CCGTAAACTTATTGAGGAAATGCA 107233
Gallid          AAACGC--CCAATATACTCTGCGATACGGAAGAAGTTTTTGTTTTCCTTAACGAAACGGG 113109
                 *****  * ** *     ***  *  **          *    *  ** * **** *

Human           GCAGTTTTTGTGCGGCGCGAGAGTGGA-------TGGATCGGAAGACGTTTCTA--TGGA 107284
Gallid          AAGATTTGTTTGTACTCTCAAAGTCGACCCCCCCTCGGATAGTGAATGGTCCAACTTTGT 113169
                    *** * **       * *** **       * *    *   * * * * *  * *

Human           TCTGAGTGAATTGTGCAAGCTGTACGATTACTGTCC-------GTTATTGTGTTC-TGCT 107336
Gallid          TCTAGATCTGATCTTTAACCCAATTGAATACCACGCCAACGAAAAGAATGTGGAAGCGGC 113229
                ***   *    * *  ** *     ** ***    *          * ****     *

Human           CTGTGTCGTGCGCCT-TGTGTATTTGT--GAACAAGTTATTTAAAATCGTAG-AGCGTGA 107392
Gallid          GCGTATCGCTGGTCTCTATGGAGTCCCCGGATCAGACTATGCATACCCACGTCAATCTGA 113289
                  ** ***   * ** * ** * *     ** **   ***  * *  *     *   ***

Human           A--ACTCGGGGGCAGTCGGAAAATCCGCTCTGGCATGCGTT--ACGGAGATATA---CCG 107445
Gallid          ATTAATTTCTTCGATTCGACGAGATCCCCAGGGCACATTTTGGACGAGCCCATCACCTCA 113349
                *  * *       * ***   *   * *   ****    **  ***      **    *

Human           TGACAGCAACTAAGCTGTATGACATCTACACGACAAGAAATTTTTTAGA-----ACATAA 107500
Gallid          TGGAAACAAGTACTTCATATGG-ATAAACAAAACAACCAATACGATGGGCGTGGAAATTA 113408
                **  * *** **     ****  **  ***  ****  ***    * *      * ** *

Human           GGGACAGCAGTTTTTTGGGGAAGCGGTG-ATTTACGGCGCGAAACATGAGCGTGTTATTA 107559
Gallid          GAAATGTAGATTATGCTGATAATGGCTACATGCAAGTCATTATGCGTGACCATTTTAATC 113468
                *  *      ** *   *  **  * *  **  * * *   *  * *** * * *** *

Human           GACACCTAGTAG-CGATCTTTTACGTTAAAAGGGAAGTCAAGGA-----AACGTTGGGAT 107613
Gallid          GGCCTTTAATAGATAAACATATTTACATACGTGTGTGTCAACGACCTGCATCAGTGGATG 113528
                * *   ** ***   * * * *      *   *   ***** **     * *  ***

Human           TGCTG----CTTGATCCTTCATCTGG--AGTTTTCGGTGCGTCTCTAGACGCGTGTT-TT 107666
Gallid          TACTGGCCCCTCCAGTCCTCAGCGGAGAAAATTACAAGGCATCTTGTATCGTTAGACACT 113588
                * ***    **  *  * *** * *   *  ** *   ** ***     **   *    *

Human           GGAATTTCATTCAATGAGGATGGATTCCTGATGGTTAAGGAAAAAGCCTTG-ATTTTTGA 107725
Gallid          TTTATCCCCCTGGATCTGTCTATGTATCTTGGAGACAGAATGGAAACATTGCAACTCCTC 113648
                   **  *  *  **  *  *   *  **    *  *      ** * *** *  *

Human           GATTAAATTCAGATATAAATATTTACGGG----------ATAAAGAAGATCACTTTGTTT 107775
Gallid          GGAAAGATCGCGATGGAAGTTTTTGGTGGTTCGAATCTGGTAGAGGAGCTACGTTGGTTT 113708
                *   * **   ***  ** * ***   **           ** ** ** *   ** ****

Human           CTGAACTATTA--AAAAACCCCACGGAGAAATCTTTTTCGGATTTTATTTTATCTCATCC 107833
Gallid          CTACAATAACATTGGGAAATTCAGGAATTGATTTCCCCCCCAAAATATCTTGTCTGGTTG 113768
```

```
                **  * **  *     **   ** * *   ** *    *  *   *** ** ***  *

Human           GGTGCCTGCCATAGAGTTTCG--AGAAAGAGGGAAGATTCCGTCATCTAGAGAATATTTA 107891
Gallid          CCTGGAAGCAGGGTGATATGATCAGCACGACGAATGCCACAGCTATCCCGACGGTATATC 113828
                  **   **       * *    ** * ** * * *   * *  ***  **    *** *

Human           ATGACGTATGATTTTCAATATCGTCCTCAGAGAAAGTTGCGCACT-TGCCCCACTCCAGC 107950
Gallid          ATCATCCCCGTTTATC--CCTGGCTTTTAAAGATGGGTATGCAATATGTACTATAGAATG 113886
                ** *     * ** **    * *   * * ***  * *  *** * **  * *    *

Human           TATTTTGACACCTCATATCAAACAACTGCTGTGTTTGAACGAGACACAGACGTCTGCGG- 108009
Gallid          TGTCCCCTCTGAGATTACTGTACGGTGGTTAGTACATGATGAAGCGCAGCCTAACACAAC 113946
                * *     *      **    **    * *         * **  * *** *     *

Human           TAATCGTTTT-TGATTGTAAGAGCCACTTGAGTGAGCAGAAGCTGTCTGTGTTTCAGAAG 108068
Gallid          TTATAATACTGTGGTTACAGGTCTCTGCCGGACCATCGATCGCCATAGAAATCTCCTCAG 114006
                * **  *  * ** **  * *   *    *    * *    **  *     * **   **

Human           GCTGTGTTTACCGTGAACGTATTCG-TAAATCCGAAACACAGGTATTTTTTTCAGAGTCT 108127
Gallid          CCGCATTCCAGTATGGGACAATTGGACGAAAACAAAATATACGTGCAGACT-CATAGGCT 114065
                 *    *  *   **     *** *   **  * *** * * **      * ** ** **

Human           GTTACAACAATATGTAATGACTC--AGTTTTATATTAATGATCATAGTAATCCAGAATAT 108185
Gallid          ACCCCTTCGATGAAGATAAATTTCAAGATTCGGAATATTA--CGATGCAACTCCAGCTGC 114123
                    *  * **    *   * *   ** **   * ** *   *   * **  *    *

Human           ATCGAGAGTACGGAAGTGCCTTCTGTTCACA------TTGTGAC-GGCTCT--TTTCAGG 108236
Gallid          AAGAGGAACACCCATGGTTATTACGGTTACGGCAGTTTTGGGATTGGCTGTAATTTTAGG 114183
                *    **  **  * *    **  * * **       *** **  **** *  *** ***

Human           AGAAGAACAGAGGAAGAAAGGAGTTTGCATTTGGTAATTGATGAGACGGAATATATAGAA 108296
Gallid          GATGGGGATAATCATGACTGCCCTATGTTTATACAACTCCACACGAAAAAATAT-TCGAT 114242
                    *     *  * **  *   * **  * *   * *  *   **   ***** * **

Human           GAAGAAATACCTTTGGCCT-TGATTGTGACTCCGGTGGCACCGAATCCGGAATTTACTTG 108355
Gallid          TAT--AATCTCATTGTTATGTAGTTGTGATTTATTAAACATATTTTTATAACTCTAGTA 114300
                 *   ***  * ***   * *  ****** *       **     *    ** *    *

Human           TCGTGTTA-TAACAGACATATGCAATCTGTGGGAAAATAATATTTGCAAGCAGACCAGTT 108414
Gallid          TTCTCCGAGTTACTTATATATTTA-TTTGTCAGACAATAATGCAATAGTGGAGAAACGTG 114359
                *  *   * * **  * ****  * * ***  ** ******        * ***   **

Human           TACA-AGTATGGGCACAAAGTGC-TGTAAACCAGTATCTTGCGGCATG--TGTAAGAAAA 108470
Gallid          AGGGGAGTCTGTAAACAGAATACGTATAATCATCTATTTGAATAAAAGATTGTGGTATAA 114419
                     *** **   *** * * * * *** *   *** *      * *  ***   * **

Human           CCGAAAACACCCTGATGG-ATTACAAGGGAA--ATCCTATATTACTGACG-ACGG----- 108521
Gallid          ATGAAGATAGCAGCAAGTCATTCCAAGCTCTCCATTCTATTTAAACAATGTACAGTTTAA 114479
                  *** * * *   * *  *** ****      ** **** * *   * * ** *

Human           AATTTACTGTTTT--GACCGATACCGA-ATCGGAAGAGGAAGGGATGGCAGATCTTGAAA 108578
Gallid          AGTTAGATATTTTAGGATAGAAATTAACATTGTCCCACAATGAGTAATCATGTCCACTTC 114539
                * **   * ****  **  ** *   * ** *    *  * * *    **  **

Human           AGCCCCTGCTTGAAAGAGTACAGGCCACATTAGCTGCTAAATGTGATACGGAAGTTGAAA 108638
Gallid          GGCTAGTACC--AAAGCGAGATGATCGTGCCAGTCACCACAATATTTTCATATCGAAACA 114597
                 **   * *   **** *    *  *     **   * * *     * *  *     * *

Human           AAAAACTGCCGCTTAAAT-CAAAAAAATGATAGACG-ACGCAG----------ATTTACG 108686
Gallid          ATAATCTATCACTTTGTCATGGAGATATCGTCGACATACGTATTATTAGCACTGTATACG 114657
                * ** **  * ***        * * **  * ***  *** *            * ****

Human           ACA---CACTCGTATTTTATTTTGTGGCTAT---TTGACA-ATAAACATGATTGTAATAA 108739
Gallid          GTATCTCTTTCGTATATCAGATAGATGATATACGTTCACAGACTAGCGTCCATGAAACAG 114717
                  *   *  ****** * *  * *  * ***   ** *** *  * * *   ** ** *

Human           AAAAAAGTGTCATTCGTTTTCATCCATTAGGCTCGAGC-----TTTCAGCGTTAAG-TAG 108793
Gallid          AAACTGATACAAATACTGGCAATCCGTCACTTTTAAGAGGAAATATCTCCGTCGGGCTGA 114777
```

```
                          ***    *   * *  *    **** * *   *  **      * **  ***   * *

Human           TTGCTTGTAC-----CGACGCTTCTTTTTAACTAGGTAGCGTACGAGTCGACAAATTATG 108848
Gallid          TTGTATAGACTCTGTCAACGCATTTCTTAGCCTTTTAAGAATTCATATCGTT--TCTATG 114835
                ***  *  **      * **** * * * **   **     **  * *   ***       ****

Human           AAACCGATCGAAATTATGGCGACGACCGATAAATTGATAATAAGTCCTG-TGCTCCAC-T 108906
Gallid          AATATTAGTTTTGTCATTGCATCCAGCCACACTCTATCAGTACCTTTCGATATAGTGCAT 114895
                **    *      * ** **  * * * * *   *   * **  *   * *      * *

Human           CGGATTCAAAGAAAGTTTCGTACTGGAGAATAGGGAAAGATA---GGCCTACGGCGC--- 108960
Gallid          TAGACTTACGGGTCATATGGAAAAAAATAAGTATCGAAAGTTTCCCAACCTATGACAAAAG 114955
                  ** * *  *    * * * *      * **  ***** *      **** * *

Human           -----CACAAAACATACCAACGT-----AATAGCCGAACAAGGGTTTAACGTAGCGAACC 109010
Gallid          AGTTGCACGGATCGCGCCCCCACCGACAAATACGTCGATAACAATTTTACAAATCGAATA 115015
                     ***  * *   **  *       ****     * **   *** **  * ****

Human           AGGACG-GCTTCTATTACTAT-ACTATATATGCAAGC-CATGACAAG--AAAGGTATTGA 109065
Gallid          CGAACATGCATACCGTACAATCGCTAAATATGATCGTATATAGTTACTCAGAAATATAAC 115075
                 * **  ** *    *** **  *** *****    *    **     *    * *  ***

Human           TGGAAGAGAAAGCAACGGTGATGGAACGTATGTAGATACTATTTCCGACGCCCAGGCAAG 109125
Gallid          CACGAATGTACTCCATAGACATG-ATCGTATAAGAATGATGTCGCCTACACCCGAGGACG 115134
                    *  * *  * *  *  *** * *****    **  * *  ** ** ***  * * *

Human           ---------TAGTAATGCCGAG-AAACATCGTAGAGTAACCTAGCATGAGTTCGCTTA-G 109174
Gallid          ATCGCGATTTAGTGGTAGTACGTGGACGTCTCCGAATGATGGATAACGGTGCCGAACATG 115194
                         ****  *      *   ** **   ** * *   *  * *    **   * *

Human           ATTGATGACAACGGTCTTGAATTGTACGGTCCCCTTTAGGTTA-GGATGAATTTTCTGAA 109233
Gallid          ATCGAGAACGGCGATCATACACCGCATGGCCCCATTTGTGTTGTGGATGTACCATCGGAA 115254
                ** **  **  ** ** *  *  * * ** *** ***  ***  ***** *   ** ***

Human           AGGCGAAAAACGACTTGTCCGCTGACTGAAATTGCGTTATCAGCGTAACGTTAAAGGTGG 109293
Gallid          TTATCTTAACTATGTTCGTCATCG-CGACAACGTTATTATTAGCATCATTGTTTGCATTC 115313
                       **     **   *   * *   **     **** *** * *    *      *

Human           TGAAACACACGAAGAAGATGGAATAGGCAAATGCACCTAGGGCGACCAGTCTGAAAGACA 109353
Gallid          TCGTACATGTCTCTAGAGTCG----GGAACATGC-CCTAAG--GAATGGATTGGATTAGG 115366
                *   ***       *   * *    ** * **** **** *  **    *  ** *  *

Human           GGGCGGTGACGAATAACTGAAATGTATCCATGATTAAGATGAATTGAAAGCAGGATGAGG 109413
Gallid          GTACAGTTGCATGCGCGTGGCTGGAAACAATGCTACA---GAACTCGAGGCATTA-GATA 115422
                *  * **  *       **     * * * *** *  *    *** *  * ***  * **

Human           TGTCC-CCCATCCAGGTAATATCCCTCGTTGTTTGGTTAACGTTAGATGTTTTGTTAGAG 109472
Gallid          TGTGTGCCCAGCATAACAGTAAACTCGTAGATTTTACTAACGCAAAAACATTGGTTGAAG 115482
                ***   **** *     * **  *       ***   *****  * *   ** ***  **

Human           GA---GAACACTTTTGCACAGCAGACAAT-------GTAATAAATAATAATCAGGAGGAA 109522
Gallid          CAATTGTCCCATTCGGCTCCACGAATGCTTCCTTTGGAAATATATTTAGACTACGGGACA 115542
                 *   *  *  **  ** *  *  *   *       * **** **     *  * * *  *

Human           TAAGATAGCCATTATCAGTACGTAGCATATAA---TCTGCACAGG--GTTTATGTAAAGT 109577
Gallid          GTAGATCTACCTGTATACTACCCACCATTGGAGGTCCTATATCGGTCGATTGTCCAAGGA 115602
                  ****   * *     * ***  * ***   *     **  *  **  * ** *  ** *

Human           --TGATGCGTCATCTGATATATTTCATTATTAGCGGAGAGATTGACCGCTTTGA----GG 109631
Gallid          CATGCAGCGTTGTATGCCAACGCCCCCGACCATTAAGTACCGCGGCTTCTATAATCAGAG 115662
                  **  ****  * **  *      *   *  *      *    * *  ** * *     *

Human           TCATTGATTTCAAAGTAGGCGCACGGAAATCCTAAATTTGGAAAGTTTATGGCT--ATCA 109689
Gallid          ATGCTCGTATCTATCTTCGTTTAGAACGACGCGATTACTACGAAGTTTACAGTTCCATTC 115722
                    *  * ** *  *  *   *     *  * *     *   *******  * *  **

Human           GATGGACCGTGACGTTTATA-AATGACAAGGCTGCACAG-ATTATAGAGAC--------- 109738
Gallid          TCTCGAACGCAATTATGAAATAAACACAAAAATAAACACTATTCTTAAAGCGGTTCTTTA 115782
```

```
                      * ** **  *   * * * **  ****   *  ***  *** *  *  *

Human           ---------CAGCCAGATCCGTAAATTAATGGTGTCTACACGACTGGAGGCCATACCAGC 109789
Gallid          TTTCTTTAATAATCGAGCATGAAAGGTGAAAGGGTATACATGTTTGGGACAAATCGTTAC 115842
                         *  *      * **  * *  * ** **** *  ***      **      *

Human           TCTGT-GATGGAGA---ACAGGTTTCAGAACGATACT---------CTGTTCTCAGA--- 109833
Gallid          GCTAAAGATAAAAACATATATGTTTGAACATAGTATCAATCGGTAGCCACCCTCAACCTA 115902
                 **   ***  * *   * * **** *  *   **           *    ****

Human           -GTGGT-TCGGCCAGAGTTTGTCCGATGTAAGATTTCCGGACAATGTCACCGTTTATTCT 109891
Gallid          CGCAATATTCGTTCCACTTTGGATGATGGAGATTTGTTTCGTGCGGTTTCTCTGCATACA 115962
                 *   * *  *    * ****   **** *   **           **  *  *  ** *

Human           CAAGCCGACTCGGCGGTGAGTTTTGAAAACGTCAGG-CAGCCGATAAAACTGGT--TCGC 109948
Gallid          TATACCGATTCGACGGCAACTGTAACAGGCGGTGTCTCAGCTACTGTAGTTGATGATTCA 116022
                 *  **** *** ***  * * *    *  **      ****   *  *  ** *  *

Human           GCAGCGATGGGTTCAGGGAAAACAACAGCTTTGATTCATTTTTTGAAACAG---GTACCC 110005
Gallid          ACTTCGGTTGGATCGAGGGACGCATCGGTC---ACTCTCGTTGGAGAACCGTCTGAAGCT 116079
                 *  ** * ** **  ** *  ** * *     * **   **    *** *   * * *

Human           AAGGAACTATCTGTCCTTTTG--ATTTCTTGCCGTAAAAC--GTTCGCTGCGGAAATTT- 110060
Gallid          GTCCCATTATTTGTTTGACTGGAATTTGGAGTGATCGAGTCAGCTGGCTGTGGGGCTGGA 116139
                     * *** ***     **  ****   *   *  *    * * **** **   *

Human           TGCACCGTTTTACTCTCAATGGGTTGGAGG--ATTTTGAATTATATTGCGATATTACGGA 110118
Gallid          CATAAAACTCCACCTTTCATTTCCACGTAATCGTTTTGGAGTTCGTCCGCTGACCACAAA 116199
                   *    *  **  *  **      *      ***** * *   *      *  **  *

Human           GCGGCAGATAAACAACAGAAAAGTC------ATTGTCCAGATAGAAA-GCTTGC--ATCG 110169
Gallid          GCGGTAGCGATTGAAGATAGTTGTCCCGAAGAACACTCAGGAACAATCGCTTGTTGATTA 116259
                **** **  *   ** * *   ***      *     ***  * **  *****   **

Human           TCTTACAGAAAATTACG---ATGTTTT----AATATTAGATG-----AAATTATGTCGAT 110217
Gallid          TTCTGTAATGTACTGTGTATATGTCCCGAAGAAGACGGCATGCTGGTAGATCGTGTAAAC 116319
                *  *  *    * *  *   ****       ** *    ***     * **  ***  *

Human           TATAAAGCA-GTTTTATTCCAAAACGA---TGACAAAAACCAAAGAAGTTGATTGCAAGT 110273
Gallid          CTCGTATCATGCCTTTCTCCAATTTGAGTATGCCTTGGTTTACATAAA--GGTTCAAGGA 116377
                     * ** *  **  *****   **   ** *       * * **   * **  * *

Human           TTTTATCGCTGATCAAAAATTCCAGTCATGTAATAGCTATGGATGCGACGCTGACTCGGC 110333
Gallid          CATTATTGCTCACAATGGACTTTTTGAGTA-AAGAGCCGTGAAT-TACCATTGACTTGGC 116435
                  **** *** *  *   * *       *  ** ***  ** **    *  ***** ***

Human           ATGTGGTGGAGTTTTTTGCCGCGTTCAAGCCAGATACTCAAATCGCCCTCAT-CAGAAAT 110392
Gallid          ---TAATGCAAGTTCAAACCATGCTCTGATGCTAAATTCAAGTTCTCCCCATGCCGTGGG 116492
                   *  ** *  **     **  * **       * * **** *   ** *** * *

Human           ACATTTGTGTCCGCGATGTTTTCC--AATAGGGTC--GCTTACTTTTGTGACACGTTTTT 110448
Gallid          GCGAATGGATGGGAAAAGTCCTCCCAAATAATGTTCAAACCGTTTTTGTTGTCGAATTGC 116552
                 *   **  *  *  * **  ***  ****  **          ******       **

Human           TGGTAAAGAGTTTTCTTTTTTTGCGCGCTTGGAAGACAAGCTACGATGGG-ATAAGAAAT 110507
Gallid          CGATCTTAACATAGGATTACCCAAACCCCCCTCCAACCATCTTGCATATCCATTAAAAGT 116612
                 * *    *  *    **       * *       ** * **   **    ** * ** *

Human           TGTGTTTGTTTTGCAGC-ACCGTGTTGGCAGCGGAATACATGCATGATTTGATACGTTCT 110566
Gallid          AATATTGATAATATATTGACAGTGATGGT-GCAAGAGAGAAAC-CAACGCGACA-ATGCT 116669
                  * **  *  *  *   ** *** ***  **   * * *  *   *   ** *  * **

Human           CGGTTTTC-ACTCAAAAAAGTTCTTTTGTTGACGTCTAAACAAGGGAAATGCTCTTCCAT 110625
Gallid          AACTATAGGACGCAACATTTCTCCATGTTTGGTTTCGCGCCATTGACAACATGTCCACAG 116729
                   * *  ** *** *   **  *  ***   **    **  *  **         **

Human           AGAATCT---TGGATACGGTATGACGTA--GTGATTTATACGTCCGTCGTG-ACTGTCGG 110679
Gallid          AGAACTAATATGGATAAGTGAAGACAAAAGGTGTTCTATGGAGCGACAGAGCAATGCAGT 116789
```

```
                    ****      ****** *  * ***  *  *** * ***     *     * * * **  *

Human               TCTGAGTTTCGAACCGGTTTATTTC-AGCTCTTTGTTT-------GTTTACATTCAGTTG 110731
Gallid              GCTCATCCACTTATCTGACGATAACGAACCCATAACCTCAGTAAGATTTGAAAATATATT 116849
                     ** *    *  * * *   **  * * * * *     *        ***  *   *  *

Human               GCCAAAGGAGGGCCGGACATGGTTTCAATATTTC----AATCCATTGGTCGTGTGAGGCG 110787
Gallid              ATCAATGCTGATAGGTACGCGGGTGCCGAATTTGTTAGAAACAATTCG-CGTATCTAGCA 116908
                      *** *  *    * **  ** * *   ****      ** * *** * *** *   **

Human               AGTT-ATCGATGAAGATATATACATTTACATGAATCCCGTACTAATTAAAAGCTATGATC 110846
Gallid              AGTCCATTAACCACATTGCCCACATGTACAGTGTCTTCACA-TAACCAAATAACAACTCC 116967
                    ***  **  *  *   *    **** ****        *  * ***  ***    *    *

Human               CTTTGGCGCCTATTGCCATGCCTCCTTGCAGTGATTG----GAGTGTCGCTGAGCAGTCT 110902
Gallid              AGTCGAGCGGAAGTATCGAGACTAGGTGTTGTTATTTCGTCAGGTCTCATATAATACACA 117027
                      * *      * *  *  * **   **  ** ***       ** **    *  *  *

Human               ATCATCTCAGAGAGTTGCATTGACTTCCGCGGAAA-GTGCAGCGGAGCGCATAAATATAA 110961
Gallid              TACTTCTGAATAAAATTTAACGCATCCCATATTTCCGCCCAGCATTTGACGAACATTTTG 117087
                      * *** *   *  *  *  *  *  * **        *  ****      *  * ** *

Human               TT-TCTGTTCCGTACTGAAGTGTCTTTTTCGATACAGGCATTATATCGAAAAGACTACTA 111020
Gallid              TTATCTTTTTGAAGCCGAAATGGTTTTTGTGTTA-AGGGATTTCGTTGTATAGCTGGATA 117146
                    ** *** **     * *** **  ****  * ** *** ***   * * * **     **

Human               TTACAAGTTTATCAGATAGTCTCTTTCTGCTCTGCAGTCTGTTATGTGAGAATTCTATTA 111080
Gallid              AAATAGCAACGCAACGGTGGATCTGGCAGGTTCACGA---GTTCCCGGGAGATCAACACA 117203
                      * *        *    *  ***  * * *   *     ***    *   **      *

Human               AGGTGGACATTG----TTGGAAATGGATTCCCGATGCG------CAAAGAGGTTTTTT-- 111128
Gallid              AGGTATACGCCGCCGCTTACAGACATAGCGCTGATACGAGTCTGTGTAGTAGTCTTTTAC 117263
                    ****  **   *    **  * *   *   * *** **          **  ** ****

Human               -----TGAGTTTC----TTGCAAATTCTAGTTGAGGAGTGTCATTTTATCGAAAAGAAGA 111179
Gallid              CACCGCAGGTGTCAACGCCGCCCGTTTCAATCTGCGTTTCACATTTGCGCTGTATAGGGA 117323
                            ** **      **   **  * *    *  *  *****    *   *    **

Human               TTACTTTGCCGGGTGACAACATGACGTTCCAAGAGATCATAAGTAGCAGA----GAAACT 111235
Gallid              TA--TATACTCTCCTCTAACATCTCCCAACGCAAGATGAACAATAACAGATTCTGGAACC 117381
                    *   * * *        *****  *    *   **** *  * ** ****    * ***

Human               ATCATGAACGGAGATTTTTATGAAAACGGTAATCAGCTATTACATAAGGACTATATTACC 111295
Gallid              TTATTGGAAAGAGTTTCACACAATACCGATTCCCTTATACCCAATGTCAACCCGGATGGC 117441
                     *  ** *  *** **   *  * * ** *   *   **    **     **     *  *

Human               GACATG---GGAAAGTTCAGAGCGACTTTTTTATCTCCGGGCGTTGACATTTTCATTGCA 111352
Gallid              AACTTCTCTATAAGGCTGTCCTCAAGAGCTCTTAATGCAGACGCAGATACAGTTAATTCG 117501
                     ** *      ** * *     * *    * *   * * * **  ** *   * * * *

Human               TCGGACATTGTATCTGATCTGAAAAATGAAAGCAAACGCTATGTTTTTGTGAACGTCTGG 111412
Gallid              TTGGGTAATATACATGAATATTCTTGTGGCGAATCTCGGTCAAACAGTTCCAACGACTGT 117561
                    * **  * * **  ***         **         ** *       *   **** ***

Human               TTACAAAAATGTGTGTCTGCTGGTGTAGAAAGCACGCGAATCGAAAGAGTTTTTAATGAG 111472
Gallid              TCAGAAGAGC-TGAG-CCGCTTCATTATAACCCATATGGATGGACACACTACAGAGAAAA 117619
                    * * ** *   ** * * ***    ** **  **   * ** ** * * *    *   *

Human               C--GAATTAAGTCTTATGTGCTGCCGAAGAGTTTTTTGTGCGATGAGTACTTTGTTCTAG 111530
Gallid              CTGAAATATAGTACAGGATGCTGCAAGAGTCCCGGTTTTA-AACATGTACCAC-TCTTAA 117677
                    *   ***  ***      ******   **       ** *   *   ****    *  **

Human               GTGACATCTCTGGGGTTTACGAGTGGGGAATGTTAATAGATCTGGCCTTCCTGGCGGAAA 111590
Gallid              ATGCCATTATT-----CTACA--TCCGGAGTAAAAGTCCCGCCCTCTTCCCTACGTCAAT 117730
                     ** ***   *      ***   *  *** *   * *    *   * * ***      **

Human               TGATCCGAAAAGATTTGAAACTGAAATCGTGCACAGATACTACTACTGACATTTCTGAAG 111650
Gallid              TCGCCCGTTGTGGCTCACGGCGGGCGGTTTCGACGGCTCTTTTCTCGAATATCACATCAC 117790
```

```
                *   ***    *  *     * *      *  ** * *  *    *  * **  *   *

Human           ATG-ATCTCCTTTTGTGTGCGGCCAGGAGGTCGAGTGAC-----ATCTTACAAATAATGC 111704
Gallid          TCGCATCTTCTATCGCATTCCGTAAAGGAACTAAATTACCTAGAATGTCATAATTAAT-C 117849
                  * **** ** * *  * * *  * *      * * **     ** * * ** **** *

Human           AATTAGTGTTTACGGTGCATG--TGCAATTTTTTCAAAGATACAGTCTGCAGA---CTCT 111759
Gallid          GGCGGGGGTACAAGTTTCAAAGATTCTAGTTCTTCACTTTTTTCATCTTCATAAATCACT 117909
                     * **  * * * **    * * * ** ****    *    *** ** *   * **

Human           GCAGCTTT-TTAACAAGTTGAGAGGCATGCG-GATTGTGACGGGTGTGTTTTCTATAGAA 111817
Gallid          GATGCCATGCCAGTAGGTGGATCGCCTCGTGCAAATTCAAGTGTTAAATACTCCATAGTA 117969
                *  **  *   *  * ** **  * *  * *  * *   *  * *   *  ** **** *

Human           AAAT--------TCAGCATTTCCATCCTTAGGCTTTTCTTTAAATGTGCATTTA-ATATG 111868
Gallid          TCATCACGGTCATTTATGTATTCATCAATGGTCACTGGTCTATATAAACTTTTACAGACA 118029
                  **        *     * * ****  * * *  *  * ** **   * **** * *

Human           -ACTTTGTCCGCCAGCAAACCTCGGTACATCCCGGGGAAGGCGT--ATCGCAATCTAACA 111925
Gallid          TACGATAGATATTGATACATCTTGA-ATGTCTCTGACCAAACATCCACGTCTTTTTTGTG 118088
                 **  *          * * ** *  *  ** * *   *  * *  *   *  * *

Human           AAAAATGATCTGGAGAACATGTTAGACAATTGGGAGATTTCGCGCACCAATCTTAAGACT 111985
Gallid          ATATACAGATCCAATAACCATCTGCGCGATAATGATATTTAAATGGCCGAGC--AAACGC 118146
                * * *        * ***    *   * **   ** ****      ** * *  **

Human           TGTAAAGAACTACGTAAAGCCCTCACCGAGGCCTCGAGAGCAAGGCGGAAACAAACAATT 112045
Gallid          TGTAAGACAACAGTTGCAGCAATCACGGCAGCGGTTAATTTTTCTCCCAGCTTGACATTC 118206
                *****   *  *  *  ***  **** *  **    *        *  *     *** *

Human           TAT-------AAACTGCAAGGCT--CGGATATAAGCCTTTCCGTTAGT---GAAGTTGGA 112093
Gallid          TGCTTCTCTAGAAATAAATCACTGTTTGATTGAAACTCATATATTAACTCCGTAAATGCA 118266
                *          ** *  *   **    ***  ** *   *   ***     * *  ** *

Human           GTTTTTG-GGCAACATGCATCTCCGGGGGTGTGCGTGTCATCTTAGCCTTTATTGTGTGT 112152
Gallid          GATCTTGTGGCAAGATACGCTTCTACAAGTGTTCTC----CCTAGAGGTGTACCCTCTGT 118322
                * * *** ***** ** *   **     **** *       **     * **   * ***

Human           ACAACGACTGGGAGAATAATATCTACAGAGTACCGATTTTTCAGTGT---------TTGT 112203
Gallid          ACCATACAGAGATGACTCTAACCTCCCAAGGCAGGCCCCCTCTGCATAAAGGAGATCCGT 118382
                ** *      *  ** *   * ** *  **    *     ** *  *           **

Human           TTTTAGAAGCGGAGACTCGTTCGTTAAAAACGTTTTTGATTAGGGGACAGA-GTCTAGAC 112262
Gallid          ACTTGCCATTGGTAACTT-TTTACTTCTCTCGCTGGCAGTTTTCAGCCATATGTATTCAC 118441
                  **   *  **  ***  **   *     ** *     **    * ** * ** *  **

Human           CAAGAATCGTTAAA---TGAGATAGAAGTTACAAGAAAAGAAACAATGCTTTGGGACCTT 112319
Gallid          GATTGATTTTAGAACTGTAAAGTATTTGCCATAAGGAAGTGTATAGAAATTGCACAGCTA 118501
                 *   **  *  **   * *  **   *  * *** **    * *    **    * **

Human           CAGGAACAAAGTAATATGATGG-ACAAAAAAATAGCAGCAATCAGCAGCCTCATTATGAA 112378
Gallid          TACATGCTGCCACACATTCCATCGTAATGCACGAGGATAAAATTCTAAGTTCACTCTTAT 118561
                 *    *      * **        **   *  ** *  **     *   *** * * *

Human           TAACGGAGAGCTTCTTAG--GAAACTTTCAAAATTTTTCGTGCCTCTAACTGTTGT-TCT 112435
Gallid          TCATTGACACTCTTTGGGAAGAGATCCTTGTAAGTAGATATATTTCTGCTTGCAACATAG 118621
                * *  ** *   * *  *  ** *   *   ** *     *   ***   **     *

Human           TGGAGACGACGGGTTAGAGATTTTGGAAG----CATACGTCTGTG-GCGAGGAACCCATG 112490
Gallid          CCACAATGGCATATCGTACAATTCGTTTGTTCTCAGACCCTAATGCACAGCGAAGCTCTG 118681
                     * * *   *   * * ** *   *    ** **     **  *   *** *  **

Human           CTGCCTTTGGACACGGTACCTGTTATATTACGATGTGTCGGAGACTACGCAGCCTTGGAC 112550
Gallid          --GCCAATACGCAAGCGATCCATAGGAAGCTCGTGCAGCCGAAGCCATAGGGTCTCGCAT 118739
                  ***  *   ** *  * *  *   *      **   * **  * *    * ** * *

Human           ACCAAGCATCTCTTGAGTAACGAATGT----ACACAGGCGTCCAAAAAACTTCGCTTTGG 112606
Gallid          TGTATGTATAGTACTATTTTCGAATATTTGAGCATCACCTTTCCGAACGTGTGGCATTGT 118799
```

```
                    * * **      * *  ***** *      **     * * *  **     * ** ***

Human          ATACAGTGTAATGGACTTTCACTTTTCGCTAACTGTGTCTG-ATGTAAA-AATTTGCTTT 112664
Gallid         GGATAATATTTTTACATTTCCATATTTATGCATTTTATTTTTATGTATACAAATTGCTAT 118859
                 * * * *  *    ****  * **      * * * * *  ***** * ** ***** *

Human          TCACACACGGACACCGG-------TGAGGCTGTATG--------TGAAAAAATGAAACAA 112709
Gallid         TTTTAAACAGGAATTAAGTAAAAATAAAACTGCATTGCTTTGATTCAACTCACGCAATGA 118919
               *   * ** *  *            * *  *** **          * **   * * **  *

Human          ATTTTTTATTTTTCTGTGTGTGCTTTTG-----GTGGTGAGCAAG--------TTCTCTT 112756
Gallid         CATTACAGAACTTCTTAAGGAATCTCTGCCTAAATGGAGAATAAGAGCATGGGCCTGTTT 118979
                 **       ****    *    * **      *** **  ***              **

Human          AGTGACGCCAAAAAAATGCTTATGTCTTATTGTTCGACGATGATTTGTGTTTGCTTTTACT 112816
Gallid         CGCGGTCCGTTTCCAAGCGAGAGCATTCTCAGGCGGGGGGGAAGTATACATCTTCCCATT 119039
                * *   *      * **    *  ** *    **  *  **  * *   *  *   * *

Human          G-CAGAGCGTGTTTGCTTTCCTGCACGAGAAG-------ATATTCGGAG---TTTATAAA 112865
Gallid         GGCATAATCTGGCCGGCTGTTGGAATACCAATGTGACACATATTTGAAAGTTTTTGCAGA 119099
               * ** *   **   *  *    * *    **        ***** * *    ***  * *

Human          CAGGTGT--TAGTTCAGCTTTGCGAATA-CATCGGTCCCGACTTATGGCCCTTTGGGAAT 112922
Gallid         CATATACAATAATTCATCTTCACGTATAGCCTCGGATCTGGGC-ACAGATTCTAACATTC 119158
               **  *    ** **** ***  ** *** * ****  * *    *  *    *

Human          GAAAGATCGGTTTCTTTTATAGGATATCCGAATCT-GTGGCTTTTGTCTGTATCGGACTT 112981
Gallid         GCAATAATGGAATATGGTTGATGACATCTGCGCTAAGTGTTTTATGCTCGAGAAAGGAGG 119218
               * ** *  **  * *  *  * ** *** *       ***  ** **    *     *

Human          AGAAAGGCGGGTTCCGGATACAACTTATATCTGCCGTGAAATTCTCTCATTTT-GCGGTT 113040
Gallid         CCAAAGCCGGACTCGTACCTTTACTATCACCAGTGGTACGCTTATAGGTTTTTAGCGACT 119278
                 **** ***  **         ***   * * *  **    ** *    **** ***  *

Human          TGGCGCCTATCCTGGGACCTC-----GTGGTAGACAC-GCGATCCCCGTAAT-TAGGGAG 113093
Gallid         CTGTTCTAGTATATAAAGCAGAGGGGGTTGTATAAATTGAAGTGGCCCTATTGCGAGAAA 119338
                 *  *   *      * *       ** *** * *  *   *  ** ** *    * *

Human          CTAAGTG----TTGAAATGCCTGGAAGTGAAACCTCTTTGCAACGTTTCAGGTTCAATAG 113149
Gallid         CTAATCGGTAATTGTCTTCTCCAAAACTATCATCTATGGGGGATTCACGAGTGCCCCGAG 119398
               ****  *    ***   *  *   ** *   * ** *  *  *      **   *   **

Human          TCAGTATGTTTCAAGTGAGTCTCTGTGTTTTCAGACAGGTCCCGAGGAC-ACACAT-CTA 113207
Gallid         ACGATGTATCTGAGTTTTTCGTAACCGTTTTTGATCGATATCGGATATTGGATGTTCTG 119458
                *  * * * * *  *   **       ***  **  * *  **   *    *  * **

Human          TTCTTT-AGTGACAGTGATATGTATGTAGTGACTT---TGCCAGATTGTCTGAGGCTATT 113263
Gallid         CACTTCCACTGGGAGTAATGGATGTAAAAGAATCTCCGTGACCACCGGCCCTAGGTCCTC 119518
                 ***  * **  *** **   * *  *    *  *   ** *     * *  ***   *

Human          ATTGAAGTCCACGGTTCCTAGGGC-CTTTCTGCCATGTTTTGATGAAAA--CACCACGGA 113320
Gallid         GCCTTCGTGATGACCTACGGTGGCGCCTTCTAACACGGCTTGATCGTGATGCATGATTAT 119578
                     **        * *   *** * ****  ** *  *****    *  **  *

Human          AATCGAACTGCTTTTAAAATTTATGTCGAGGCTGCAACACAGATCGTACGCTTTATTCGA 113380
Gallid         ATTCTCCTTCTCTAGACGATCCTAGACTAGATTGGGATATTCTTC-TATGTCTATCGTGA 119637
               * **    *   *  *  **    * * **  **  * *    ** ** *  *     **

Human          TGCGGTGATCTTTATG--TTAGATGCTTTTGTTTCCGCTTTTCAGCGAGCATGTACTC-- 113436
Gallid         TTTATCAAATTTTGAGAACCCGTTGCAGATCGTCTGGAACCCCCACGAAAATGCATATGC 119697
               *      *  ***  *     * ***   *  *   *      *  ***  *** *

Human          -TAATGGAAATGCGATGGCTGTTAGTGAGGGACTTACACGTGTTTTATTTAACCTGTGAC 113495
Gallid         ATGCTGGCATTACGATGGCGTCGTCTAGATGACGAGCGTCTTCTAGACCTCGCTCTCGAT 119757
                *  *** * * *******      *    ***   *   *  *  *  *  *    **

Human          GGTAAAGATTCAC--ATGTTGTCATG-------CCTTTACTGCAAACGGCCGTTGAAAAC 113546
Gallid         TTTAATGATGCATTAATCATATCACGAGATGTATCACTACCGCGACGACCCGAAGGTTGC 119817
```

```
                                *** *** **   **  * *** *        *  *** ** *    ***  *    *

Human           TGTT--GGGAAAAAATTACGGAAATCAAGCAAAGACCTGCATTT-CAGTGTATGG-AAAT 113602
Gallid          CTCTCAGTGAAAGTATCTCGGAGATAACCTAGAAAACTATGCCCATTGCTTCTGCCAGAT 119877
                   *  * ****  **  **** ** *   * * * **         *  * **  * **

Human           TTCGCGCTGTGGATTTGTCTTCTAC-GCTAGATTTTTTTTAAGCAGTG--GGTTGTCACA 113659
Gallid          CGTGTACGGTCCCGCCCCCGTGAATTGTCAAATTGTTCCTCATTAGGAATGGTTTTTACA 119937
                   *  * **        * *  *  *  * *** **  * *  **    **** * ***

Human           ATCTAAAGAAGCGCACTGGACAGTAACGGCAAGTAAATATTTATCTGCATGTATTCGGGC 113719
Gallid          AACCCATTATCCTCGCTCTCCTCTAGCGTATCGTATGTATC-GTCATCATAGGCAATCGC 119996
                * *  *  *  * * **   *  ** **    ***  ***   **  ***        **

Human           CAATAAGACAGGTCTTTGCTTTGCCAGTATAACTGTTTATTTTCAGGATATGAT-GTGTG 113778
Gallid          CGGTGCT-CTTGTATACACACTCGCCATATTATGCGGACTTCGCGATATACGATTGCGTG 120055
                *  *    *  ** *   *  *  *  *** *       **  *   *** *** * ***

Human           TTTTTATAGCTAATAGGTATAATGTTTCTTATTGGATCGAAGAGTTCGATCCTAATGATT 113838
Gallid          ATCC-ACTTCTAGTCGACAAGTAATTTCT-----GATTGATTGGTTTTCCCGTCGTTGAT 120109
                 *   *   *** * *  *     *****     *** **   ***    * *  *   *

Human           ATTGCCTGGAATATCATGAAGGACTTCTGGACTGCAGTAG-ATATACGGCCGTGATGT-C 113896
Gallid          TTTGACCAGGATGTCCATACCGATGCATAGAAGGCATTTGCATTCTTCGGTGGAATGTGC 120169
                 *** *  * ** **   *  **    * **  *** * * **     *  *  **** *

Human           TGAAGATGGACAGCTTGTCAGACAAGCACGTGGAAT--TGCATTGACTGACAAAATAAAC 113954
Gallid          TACCAGCATACTGTTATCAAAACTACGCCGTTTTATAATGTATTGATCTAACGGCGGAAG 120229
                *        ** * *    * ** *   ***   **  ** *****   *       **

Human           TTTTCTTATTACATTCTCGTTACAT---TGAGAGTGTTGAGGAGATGGGTGGAGAGTAAA 114011
Gallid          TGTAATTGCATCAATAATATTACATCATTGATGGGACGTGAGAAACAAAGACAAAGCGAA 120289
                * *  **    ** *    ******   ***  *       ** *       * **  **

Human           TTTGAAGA---CGTTGAACAGACAGAGTTTATTAGGTGGGAAAACA---GGATGCTCTAT 114065
Gallid          TACGACAAATTCGTTTATTAAACGTCACAT-TTACGTATAATATCATTTAAGTATAGTTT 120348
                *  **  *    **** *  * **      * *** **   * * **       *    * *

Human           GAACACATTCATTTGCTA-CATTTGAATTAATTT-TGTTTCTTTACATAACATAGAAA-- 114121
Gallid          CGTTCTGCCAACTCACCATCATACTAATAGACAAATACCTCCTTATAAAGTACTGATAGT 120408
                          * *  * * ***   ***  *    *   ** *** * *  *  ** *

Human           AATAAGACTTAGCTAGACGTGTC---TTCATTCTTCGAATAAAAAATTTGTGCGAATTGT 114178
Gallid          AGTGGCAACAGGATCACCAGGGGAAATTGAATTTCTGTCTGAAGAGTCCATCAACAATGC 120468
                * *   *    * *    *  *     ** * * *  *  * ** * *   *    * **

Human           GTTGCTAGTTGT---ATGACTTGGAGAGAACTGT--CTTGCAGCTGTCTAGATTCTAAGG 114233
Gallid          TTCGACAGTAGTTCCATAGTTTGGCGGTGATTCTAGCTTTTGAACATGTACATGTTCCTT 120528
                 * *  *** **   **   **** *   * * *  ***       * ** **  *

Human           ATAAATTGATGAAATTGTGACTACGTTTTGTGTTA----AAATAAAGTTCCAAA------ 114283
Gallid          ATGCAC--ATGCAATTGCGGGTTAGGGGGGTGTGATAGAGAATATACTTCCAACTTTGAT 120586
                **  *   *** ***** *  *  *    **** *     **** * ******

Human           -------ACTTCTATATGCGTGTTTTTCATTTCATTGTATAGTCTAATCATCGGAGGCAC 114336
Gallid          CTAATACACTGCAATAAATATACTGATGCCCGAGGAGCATCACCTGAAAACGGTGGGGGC 120646
                       *** * ***    *  *  *         * **   ** *  *  *  **  *

Human           CTTGTGGAGGATTTCTGAGGCGTGTTCGTTAAAAAAAATGTGTCTTAGTGTACAAACCAA 114396
Gallid          GAGATCAACGGCATGTCAGGCTCTTCTACTAATTTACAT-CGTATTAGAGGAAGACCGAA 120705
                    *  * *   * * ****   *    ***   * **  ** **** * *  * * **

Human           TTTGTCGCACGTGT-----TGAATAATATTTTCGTTACCGGAGATAGGACGGG----GAA 114447
Gallid          CTCGGAACGTATATAATTCTGAGCCCGGAGTTCAGGAGCAGTCAAAACACGCCCTTCGAC 120765
                 * *   *   * *     ***         ***   * * *  * *  ***       **

Human           AAGAACGAGCCTAGCGATTAGTTTGTGCAGGGTGCTTTGCTTCAGTGGAAGATTCTCTAA 114507
Gallid          AATAACTACGCCA-TGGTTAAAAATTACAGGTTGAAATAAA-CAATGAAAGTGTCTTTCT 120823
```

```
                  ** *** *  * *  * ***     * **** **    *     ** ** ***  *** *

Human             TGCGAACACTAGTCCGCAAAGCATA--AAT-TCTTCTGGCGTATCCACTTTCATATT--G 114562
Gallid            TGTAACCATTCGCATTTCAAATATACAAATATATTTTGTCGTCCCATTTGAGATGCTTCG 120883
                  **  * ** * *       **  ***  *** * ** ** ***  *   *   **  *  *

Human             ATTAGTC----TGCGTTTGAACTG--AAAAGGATTGAGTCGCAGTTCTTGCAGTGT---- 114612
Gallid            AGTCGCTGTGATACATCCCGCGTGGTAGAAAGGTATAAGTGCACATATAAGAGCTTCGCA 120943
                  * * *      * * *       **  * ** * *  *   ***  * *   **  *

Human             -ATCTTCCGAGTCTAGTATTATGA-TGCCAATGAAGCCTTGGAATCCCAACTCTTTTTCG 114670
Gallid            AACCTTGCCGCTTCTCGATAATACCTTTCGGCGATGTATTGTCGTGCTTTACTACACGCA 121003
                   * *** *   *     ** **   *  *   ** *  ***   * *           *

Human             CAGATGGTCTTGTATTCCA---GGCCGTTGATCATGGTTCCGTCTTCCAAGTGGA-ACCA 114726
Gallid            CCAATATTTTTATCTTCGACTCGAGCGTCCCGAAGATCTTTAATCGCCGACTGTCTAATA 121063
                  *  **  * ** * *** *   *  ***     *    *       ** * **   *  *

Human             TATTCCGTCGTCTCCTACTGTTATATTTACTAACGAATGTCGTGCGAGGGATACGCACTT 114786
Gallid            TACTCTGCCAGAGCACGACAGTATCCATAAAACATAGTAGCATATGATTTCTCCCTTGCT 121123
                  ** ** * *    *       ***   **  *   * *  * *  **    * *     *

Human             ATCAATCTTGATCACCTTTAGGATAGT---TCGGACGTGTAAGGTGTTGAATACGTCTGA 114843
Gallid            TCTAATTCTCCCAAATAGAAGTTTTGTATGTCGTCTACATAAGAAGGTAAATCGGCCTTT 121183
                     ***  *    *     **  * **   ***       ****  * * ***  * **

Human             GGTTTCTAATTCTTTTA-------ATTTTTTCAAGGAT-TTCATTCTCCGGGTTTGGGGA 114895
Gallid            GTTGTGGGAGGCATGGGCAGGGCCATCTCTCCATGTTCATTTAAATTTAAAGCCGTATAC 121243
                  * * *   *  * *           ** * * ** *    ** *    *     *

Human             GAAAATGTTCGAATCGAAAATCGGCTCGTTGTTTTCGGGGTGCGTTTGGGTTAAATTATA 114955
Gallid            GTCCATGTATTCTTCGACAACTCTTCCAATACTTCCGAAGGAGATACAGATAAAATTTTT 121303
                  *   ****     **** **      *  *  ** **  *    *   * * ***** *

Human             CACTTCTGAGATCTGATTTAGCTCTCGGACGTATACGAGTCTTAAATAATGCTTGATTTT 115015
Gallid            GCATGTTCATATAAGTGTTTATTATGAGGGAAACATGAGAATAGATCTACATTTATTTTT 121363
                     *  * * **  *  **   * *  *    * * ***  *  *   *   **  ****

Human             GC------TGCTTTCGTAGATGG----CTAATAAGATTTCACCGAGGGATAGAAAGAGAA 115065
Gallid            TCCAGACGTGCTAATATAGACGGGCCTTCGACAAAATCCAACTCATGCA---ACAGTCTA 121420
                   *      ****    **** **       * ** **   **  * * *   * **   *

Human             ATTCCTCGGGAATTGATTCGAGGTTG---TAAATATTTAAGAGTCGCCTTAGGAA-AGGC 115121
Gallid            GTGTAGAGAGAATTGGGAGGAGCTGGAGATGGGGATGGAAGTGACATTTTAGTAACAGAC 121480
                   *     * ******    *** * *   *    **  *** * *   **** ** ** *

Human             ACGATGAGCAGCT-CGATGCTATAGTTAGAGTATGTGAT---ACTTTCTTCCTGTCCTTG 115177
Gallid            GGAGCAACCAGAGGCGATGGAATAGATTGGGT-TCTGACTTCAATTTCAGAGAGAAGCGT 121539
                        * ***   *****  **** * * ** * ***    * ****     *

Human             ATTATTAACGTTTTTTCTTAACCGAAAGGTTCGCATGTATTCCA--TTTCCCATAGTTGG 115235
Gallid            TTCATCAAATTCTTCAATTGTTCGTAGGATATCAAGATCCGGGGACTTATCCATAGTATT 121599
                   * ** **  * **   **   ** * * *    *  *         **  *******

Human             TCTATTCGTTTGTCGGCATATTGTGTGTCTGGGATATACTGTGAAAAAAAACTGTTTGCG 115295
Gallid            CGCATACGCCT-CAATTTCAGCGAATAATTGGATAGGACTGTAGTAATCGTTTTCAAAAC 121658
                     ** **  *         *  *  *   ***      *****   **      *

Human             ACTGATCTGGTGTCAT-----CTATGGAAACGCTAGTGAATGGGA-GCCTTTGAACTA-T 115348
Gallid            TCATATTTGCCTCCATTCACACTATACAATAATGAGTAGATAAGATATATATGTACTAGT 121718
                   *  ** **    ***      ****  **      ***  **  **    * ** **** *

Human             GTTTAGCGCTCGCG---ATAAAGAGAGATT--CTCGAGGTCATGGTCCAGATCCGG-GTT 115402
Gallid            TTTTAATTCGGATGTCTATAAAAGACGACTTACTTGCAGTAGTAGGGCTGTTCCTATGTT 121778
                   ****   *    *   *****    ** *  ** *  **  * *  * * ***   ***

Human             ATAGCGATCACAATCGTTATT-CG----TCTCGAGCAAA-GAGTG-TAGATCTTTGATTT 115455
Gallid            ACATTATTCGCTATCACTGCTACGATATCCGCGGGCGGATGAATGATGGCGACGCGAAGT 121838
```

```
                * *    ** * ***  *  * **     * ** **  * ** ** * *      **  *

Human           TCCTTTCGAACATTTGGTTTGT-TGTTTTGAGTGTTTCGATTTCATCCATCTGTGTTTGT 115514
Gallid          TGCGGGAGTGCGAGGGTTATGTGTACGATGAG-ATCCCGGATCTGGCGATTTGATTTCAA 121897
                * *    *  *    * * *** *    ****  *  **  *    * ** **  **

Human           AT---CTGCTCCTCCATGCACTTGATGATTTGTTTTT-TGAACAGATCTCGA----ACGT 115566
Gallid          TTTCACCGCCGCGTCTTGTACGTTCAGATTTGGTTGTACGTTCAGATTTGGACTTTACGT 121957
                 *   * **  *  * ** ** *   ****** ** *  *  ***** * **    ****

Human           -CTCTCTCTCCTGTGTCGATCGCAGAGCGTTTTCGGAACTGTCCTCGGTTCAGCATGAGT 115625
Gallid          TCTCGTTCCCCTGGGATAATC-CAGACTCTTCC---AATAGTTTGCGG----GCACAGGT 122009
                 ***  ** **** *   *** ****   **     **  **   ***    ***   **

Human           TTATTTTGGTCTATTATAGAGGGGGTGATTTCTTGAATAAATCCTTCAACGCTGTCCCT- 115684
Gallid          TTCGGCT--TCCCCCATCAAATTTGGACCCTCTTGAATGGTAATTTTAATGACAGCTCTG 122067
                **    *  **    **  *    *     ********      ** ** *    * **

Human           ------AATGCCT---ATTTTAGATTTGCTGTCCGACAAG-TTGA-GTAAAAATTTGATT 115733
Gallid          GAAAGAAATGCATCTAATTCTTCATTTGTTCGCGGAGGATCTTGACGCCAAAGGGCGAGC 122127
                      ***** *   *** *  ***** *  * **  *  **** *  ***    **

Human           AAACTTTTTTTCGCGTCGCTGTTTTTTTCTTCTTTTTCTATCATGTCCATTATCTTTTTG 115793
Gallid          GCGCCTTGGTATGCGTGATATTGAGCCACAGTTGCAACCGCTCCACAAAACATACGCTGA 122187
                   * **  *  ****     *      *   *    *          *  **    *

Human           TTGGTTACATCTGTCTTTGCT------GTGGTTAGCACCTTTATGGGAAATGTATTCAAT 115847
Gallid          TTAAATGCCACTGTATTTGATCTCCAAGTGCTAGATGCTGATGAAGGAGCCGTACTGAA- 122246
                **   * *  **** **** *      *** *     *   *   ***   *** * **

Human           AATTGGCACATTTTTTGATGACGCCGCAGACTATTGTGTTTTTTTAGTTCTGGA-GAAAG 115906
Gallid          AGCGAATTTATTACTTTGTACAGCTCTGACCCCGGGCTTGGCACGGGGTTTGTTTGTAAA 122306
                *        ***  **  *   **      *    *  *       * * **   * **

Human           GTGGGCGAGGGGTGTAGTC--AGCATT-----AGGCGATTTCTAATAACATATGGGGTGC 115959
Gallid          GCCACCCCGGGTGGTAGACGAAACGTCTTCCGATACGGCTTTCGCCGATTTATG--TTTT 122364
                *    *  ***  **** *  * * *      *  **  **      *  ****   *

Human           TGTACGTGAAAAACGCTATAGCCTTCTTGTTTTTAAAGCAAGAAATTTGCTCCGAATTCC 116019
Gallid          TGTGTTGGATGGTCGGTAAATGGTCCATGTTTTGACA--AATCATCCTGGTTTAAATTTC 122422
                ***    **    ** ** *   * * ****** * *  **  *   ** *   **** *

Human           GATCTCGGAAAATCTTAATATTATGAAAATCCGGAATGA----TCACGGAAGATAGACCC 116075
Gallid          G--CTGAGTACGTGTTGATGGTCTGCGAGCAGGAATCGAGACGTCATCATATGCAGAGGG 122480
                *  **  * *  * ** **  * **  *    * *  **    ***    *   ***

Human           AATAAAATGGGAGTGACAATGTTTTG-TTTATGCCGCACTATGTCGTGGAT--TAGTTTA 116132
Gallid          ATATCCAAGGGAACGACGCCGTTCCGATTTCCGCCTTTCAGAATCCCCCATGATAACTAA 122540
                *     * ****  ***   ***  * ***  ***   *    **    **  **  * *

Human           GATGACACTTGTTGTAGAAGATTGTATAAGTTCGGGGCCGTAGGGGTGGTTCTCATGAAC 116192
Gallid          GATATAA--TATTAAACAGTAAGATATGAACAAGTGCACCTTCTATTGTACCGTGTGGCG 122598
                ***   *  * **  * *  *   *** *    * *  * *     **   *   **

Human           GGAACGATTCCTACGGTACAGATCCA--GTCTACATATCTGTTATAGTGAC-TGTTGTCT 116249
Gallid          TCTCCAACATATG-AATATTGGTTCAAAGTCAACAAATCTCTTATGAAGAGATAAGAGAT 122657
                    * *    *    **  * * **  *** *** **** ****   **  *      *

Human           ATGCTCTGAAGGATAAAATTAATTATA-----TTAGATATATCTCCCAAGATCACCTGTT 116304
Gallid          TTGTCCACGCCCACATAATTTACCACTCCTCTTTAAACATATCTGCGGTGAATAGTCGAA 122717
                 **  *      * * **** *  *       *** * ****** *   **  *   *

Human           TAATCGCGTGAGCCCAGAC-ACTAAAATAATCTTGA--AATGACAATTTTTTGTTGTGGA 116361
Gallid          AGCATGCGTGATACGAACCCGCTAAGAGGGCGGAGATTACCGCCACCAGACCCATATAAA 122777
                     ******  * *  * **** *       **  *  * **         * *  *

Human           ACGGAAATGAC--CTCACATGACGTTTCCATTCATCGTGGATTTGTTGAATAGAAACGCT 116419
Gallid          ACAGCACAGAAAACCCAGATGAAAAAGCTATTGACACGCCTACTGCAGAACACGTAGACT 122837
```

```
                    ** * *  **   * ** ****     * *** *         **  *** *   *  **

Human               GTTTAAAA------------GGGCTTGAGCTACTCCATAGGCTATTTGTTTTTTCAGTAC 116467
Gallid              CCCAAAAATCTCCCATACTTGTGATTGAACTTCCCCAATCGACAAACGTCGCTT--GCGA 122895
                        ****            * * **** ** * ***   *  *   **   **  *

Human               GGCAGAATCTCGGAGAGCGTTGTGGATTGTTTGACCTTTGAAAAAATTTTTG-TGTCCGT 116526
Gallid              AGTGGCAATCAAAATAGCAACGCCGATAACTAAACTACAGACTGCATTATGAATGTCCAT 122955
                     *  * *      * ***   *  ***   *  **    **    *** *   ***** *

Human               G--TAGCATTTCAATAAAAGTCATTATCTGCTCAGAGGGATA----AATCATGTCTATCT 116580
Gallid              GAGTCCCATCCTCGTCGAGATCGTGATTTGGAGAATATAACGTTGGAAGCACTAACAGTT 123015
                    *  *  ***     *  *  ** * ** **   *     *      ** **     *  *

Human               CTTCGTTGTTCGGAGGTAGATA-CGATAATAAGTTTT-TGCTTAACATGTCTTCGGCT-- 116636
Gallid              CCCTGGCGTCTGCAATTCCGTGTCGATGAAGCGTTATATGCAGTAAACCCAAACGGTTGG 123075
                    *   *  **  * *  *   *  **** *   *** * ***   * *      *** *

Human               -CCCAAAATAGAGTTGATTGTAGATAAGGTGGCTTCGTTATCAGAAAGACGCTTGGAGGG 116695
Gallid              ACCTGTTATATAGAAGAGCGTGATCAGCGTTGTCTTCGCGTTACCAA--CAATTGTGTGA 123133
                     **    *** **  **  **    *  ** *  *     * *  **  *  ***   *

Human               GCGTCTGCCAGAAGATTGGTTTCGCCACATCATGGATCCTGAAACCGAGTTCAACAGCGA 116755
Gallid              TCTCGTTACTGAA-ATCGGATTTAAAGCG-CAGATATCAAACGTGTACATTGAATATCGG 123191
                     *   *  * *** ** ** **      *  **   ***            ** ** * **

Human               ATTTGCTGATGCTCTGTGCATCGGTATTGATGAATTCGCTCAACCTCTGCCTTTTTTGCC 116815
Gallid              AATA--CGAGTTGCCGTTCCTCAGAATT-ACGTGGTCATTTTGGCAAAGTTAACGGATCC 123248
                    * *    **    * ** * ** * *** * *   **  *    *   *         **

Human               GTTTAAGGCCTTGTTAGTAACCGGTACGGCCGGGGCAGGGAA--AACGAACAGCATTCAG 116873
Gallid              AGACCCAACTTCTCGAGGTATACCCATCATTCAAGTCGCCAATGGATTGATAGATTCAGG 123308
                            * *    **  *     *        *  *  **   *   * **  *   *

Human               ACCTTAGCGGCCAACTTAGATTGTATCGTTACTGCAACGA-CATCTATCGCCGCTCAGAA 116932
Gallid              ATATAGGGGATCTATCCGAGCAGTATTGTTTTTGAAAAATCATGTATCATTCCAAAAAAA 123368
                    *  *  * *  * *        **** ***  *  **  * *** ****    *  * **

Human               TTTGAGCGTTGTT--TTAAACAGAAGTAAATCCGCGCAAGTTAAAAC---AATTTTTAAG 116987
Gallid              TGGGCTGGCTATTCGTTTATCACTAGTAAAGCTG-GCATCTCCCAATTTGAACACACGAG 123427
                    *  *   * * **  ** * **  ****** * * ***  *   **    **      **

Human               -ACTTTTGGGTTTAATAGTTCACATGTATCTATGAGCGAACGTCAAAGTTATATTGCAAA 117046
Gallid              TGCTATTCAATCTGTCTGACATTACACCCCACCTAGAATGTGGTCCAGACTTTTCAACTT 123487
                      ** **   * *    *     *     *    **      *    **    * *

Human               TGACGAGAGGTCGATTCAAATTCAACAAAAGCAAGACCTGTCTATCTATTGGAATGTTAT 117106
Gallid              CGATAGAAACTGCTGTCAGATTAGGAAGCGGGGAGACCAAACCA-CTGTTGCCGCCAAGT 123546
                     **     *  *    *** ***    *   *  *****   * * ** ***        *

Human               CTCCGACATAGCGGAGAGGGCGTTGGGTGCTGT-CGCGTGCAAAACTAAAGAGTTACCTG 117165
Gallid              GGAGGTGGGATATGGGCAGGAACTGGATGCCGAGCGCTCGCATGCTTATACAACGATCGG 123606
                        *    *   * *  **   *** *** *  ***  ***     ** * *   * * *

Human               ATTTGTGTGAGAGTAGTGTTATA-GTTATCG-ACGAGGCAGGCGTA---ATATTACGACA 117220
Gallid              GTATGCA-AAGCGTCGCATTATACATCATCGGATAAAAATATCGCATTTGTAGTACGGTA 123665
                     * **    ** ** *  *****  * **** *  *      ** *    ** ****  *

Human               TATT--CTGCACACTGTTGTTTTTTTCTATTGGTTTTATAACGCGTTGTATAAAACCCCT 117278
Gallid              TAATGATTCCACAAGTGTGTTAGGACTTAAAGATTTTCCGAC-TGCCGAAGACGAAACGT 123724
                    ** *   * ****    ****       **  * ****   **  *  * * *  *  * *

Human               TTGTATGAGAACGGAATTGTTCCGTGCATCGTGTGTGTGGGGTCGCCCACGCAGAGTAAC 117338
Gallid              TTGTACG---TTTCTATACATCTGGTCAATTTGCAACTCTCATTCCTTTCTTTGAGACCT 123781
                    ***** *         **   ** *  **   **    *    *  *   *   ***

Human               GCTTTGGTGACTTCATTTAATCCGCTGACT-CAAAACAAGGACGTGAAGAGAG----GAA 117393
Gallid              TTACTCCTAAACGAACAGAGGATGCTGCCTACGACATAGCCGCCCCAGGTGATATTCGAT 123841
```

```
                    *  * *    *   *    **** ** * * * *    *   * * **     **

Human           TAGATGTACTTTCAGCCTTGATC----TGCGACGAC-GTGCTCTCTAAATATTGCGAAGT 117448
Gallid          TGGGGGCATTATCGTCTACAACTATCATGATACAGCAGCGATATGTATGTATGGACGACT 123901
                * *  * * * **  *    *      **  **  * * * * * **  *** *   * *

Human           CGATAATAACTGGATAATCTTTGTAAACAACAAAAGATGTGCGGATCATGCGTTCGGAGA 117508
Gallid          CTGTAATACCTTGCATATTTGGACGCTCATCCATGAATCTACGAGGCTTGATTATAATCC 123961
                *  ***** ** *   ** *       ** * *   ** * **   * **  *

Human           TTTTTTAAAGCACATAGAATT--TGGCTTGCCGTTAAAGC-CGGAGCTTATAGAGTATGT 117565
Gallid          CCAGTAGATGGCTTCCAAATTCCTGGCTAACTATTACTATATGCAATTTGACCGAAATGA 124021
                    *  * *       ****  *****  *  ***       * *  **        ***

Human           CGATCAGTTTGTGAAGCCCGCTTCGTACATAAGAAATCCCATGAACGAAATA-GAAACGA 117624
Gallid          CCGTTATGATAAGATGTGGAGACCGTATAGCTCAATTGTTGCTTGTAGATCACGAATCGG 124081
                *  * *   *  ** *       **** *    ** *           *  * *** **

Human           CC-CGCTTGTTTTTATCGCATAACGAGGTAAAGAACTATTTCCGTTCATTGCATGAGCAG 117683
Gallid          CCACGCTAATTCCACCCACAAATGATACTACTGGAATGTTTCC---CACAGTAGGTAAAT 124138
                ** ****  **     * ** *      **  * * * *****   **  * * *   *

Human           GTTGAAGTTACCA-ACAGAAATAACTTGTTTGTGTTTCCGGTTTATTTCTCATAAAAAA 117742
Gallid          GTCGTCGCCCTGGCGCATCGGTCGGGGAGCCGAAATGGAGGGAAACCTTAGAATTTGATA 124198
                ** *  *        **    *         *   *   **   *  **   **   * *

Human           CAAGACTTTCGAAGACTACAAAAGTGAAATTGGAAATTTC-TCCTTAGAAATTGAACCGT 117801
Gallid          CAGAAGCTTACAGCAGCGAGAGACAGTTTTCCGGGTTTGGATCCACCGGTATATAATTGT 124258
                **  *  **  *  *     * *  *   *  *   **   ***    *  **  **  **

Human           GGTTTAAATCTA---ATATTCATCGGCTGAATACTTATTCTCAGTTCGCGGA------TC 117852
Gallid          ATGAAAACGCCATTCATGTACAAAGAATTATTACTTTACAACAATAAACGAAACATACTC 124318
                     **  * *   ** * **  *  * * *****     ** *   ** *      **

Human           AGGATTTATCGAAAACTGTTCAATTGGAG---------GAGATTGTTTT-----GGAAGA 117898
Gallid          GAGAGTATTTGCTTCTTTCTTTATTATAATTCGGTAATGAGATTATTTTCCCTAGAAAAC 124378
                  ** *  * *     *  *  ***  *          ****** ****     * **

Human           TGGGTCGGTGGAAGAGACTTTGATAACCTGTCATCTGAAACATATCAGAA---ACAGTTC 117955
Gallid          TTAACTGGTGGGCAATTATCCTTTGGGGTTACAGCCGGAGTAGAAATAATTGTACGTTCC 124438
                *     *****   *    *     *    *  ** * * *  * *    *    **  * *

Human           AAT---AGGTGTGACGTCCAAGATCAAGGCTT----CGACCGTGGGGTTTTCCGGAACTT 118008
Gallid          CTTTTTGACTGTGAGAGTAAGGGCGAAGATATTAGGCGATCGATGGTTGTATTATAACCG 124498
                  *      *****     * *   ***   *    *** **  ** * *     ***

Human           A-TGAAAAATTTGTAGAACTGTTAC-AGAGCGATTTGTTTATCGAAAAAACGT-CATGTG 118065
Gallid          GGTTCCATATTCACAGCATCGTCCTTATTGTCATTCATATGTGACAATACTGCACAATTT 124558
                  *   * ***   ** *  **     *  *  ***  * * *   ** *  *  **  *

Human           AGCAGACTA-TTCACGCATATTCTTTTTTATCCGGTCTTATGTTCGGAGGGATGTATTCT 118124
Gallid          TTCATCCCGGTTGACTCGTCCATTGATGCGGGCGATCTCTCATTATGTGG-ATGCGTTAA 124617
                  **  *   ** ** * *     *  *      ** ***    **  * ** ***  **

Human           TTCTGCTGTTCGGAATTCACCACCCCTGAAGTGTTAATGGAAATCAAAAACATAAAGATG 118184
Gallid          GCCCAACGCTCT--CTCCACCAGTGCTATATCGCTCATCGCAGTTTCGGTAGCAGCCGTT 124675
                  *    * **    * *****   **  *  * * ** * * *         *    *

Human           CCCAGTATTGAATTCTTAGAAAGTGAAAT-GTCGAGGATGTCTCGA--GACGTGCAAACC 118241
Gallid          AATTGTATTGCATGTTCTACAAATGCATCCGTTCGCCCTGTGCCGATAGACATATACATA 124735
                    ****** **  *    ** ** *   **      ***  ***  *** *  * *

Human           GTAGAGACAGATGAAAGGTATG-ATTTCGGATTAGTCGA---------TGATGGGTTATC 118291
Gallid          TGCAAGAGTGCGGCAGCACATGTATCTGCAACTCGCTGTGCATTCTCCCGATGCGTT-TC 124794
                    ***  *  * *    *** ** *   * * *  *           **** *** **

Human           AGATATGGA--CCTATTAGAAATTGATCCTTGCGGAGATCCATTTTTTACTAGGTATAGT 118349
Gallid          AACTACCGCATCCAATCCAGGATTTCCCCGTCCTGGAA--CATGTCTAGATAGACATTCT 124852
```

```
                *  **  *   ** **     ***   ** * * *  *  *** * *   ***  **  *

Human           AAGTTGCCTTTAACTAATTCTCTGT-CGTTCGAGGAAATTAGTTTGCTTTATACGACGTT 118408
Gallid          AAATTGCG---AAGGCATGCACTGTACGTTCTAGCCAAT-GATTGGCCACGAACCAGTTT 124908
                ** ****    **   ** * **** ***** **  ***   ** **     ** *  **

Human           TAAGGATATCTTTATATCTAGGTTTGCCATT-CTGCAG----AAGCATACAAAAGGGAAG 118463
Gallid          CCGTACCCCGTCCGCAGATAATAAAGCCATTTCTAAAGTGATGGGCGTGGGAAGAAGAAT 124968
                          *    *  **     ****** **  **      ** *   **   ***

Human           TTCGGAAAGACTCTCCTAGTGACTTATAACAGAAACAACGTTTC---TAGAAAACAGTGC 118520
Gallid          GTTAACGGCATGGACCGCCTCCCTGAGACGATGCATAGCTTTATCAGTAGAAGATGATGC 125028
                 *       *    **   *  ** * *  *   * * * **     ***** *   ***

Human           GGTGAGATATAT---TCCCATTTAAAGAGTTTTTATGGTATGCTCACCTACGCTATCCCT 118577
Gallid          AGACAATTGTTTATATTCTGCTTTGAGTCTTCTGTAGTCGTATGTTCTTGAGCTGGTTTG 125088
                 *  *  * * *   * *   **  **  ** *   *   *     * *  ***

Human           GCCAATAATTATACTCTGGAGGGTTATACAAATGAT--AATGTAGTTCATTTAGGAACGG 118635
Gallid          CATTATGGCGAGATTTTCGTCTATATCCGATACGCTCGAAAGTGATGACTCGGGAATTAA 125148
                    **    * * * * *    *     * * * *  ** **  *   *   * *

Human           ATAAACAATTACCGCAGATC-TTATATAAAAAAGGATTACCTAGATTAGTTATAAAAGAT 118694
Gallid          GGTCTTATTTGCCGTAGATGGTTGTGCCGTGTCGTTTTCCCTGGCCC--TTCTTACAGGT 125206
                      * ** *** ****  ** *        *  ** *** *     ** * * ** *

Human           GAAATGGGGTTCATT---TCTGTTTTAGATAATAACGTGTCGAAATTTGTCGAT--GTCG 118749
Gallid          CAGATACCCTCTACTAACTCCGTTTATGTTATCGGCTATTGGGATCCAAGCGACCGATTT 125266
                 * **    *  * *   ** ****  * **    *   * * *      ***    *

Human           TAAATGGTCAGAGTTTTC-------ATCTATGTAC-AACGGTCGATTAC--GCCACTGTT 118799
Gallid          TCAAGCATACCCTTTCTCGACGGGGATCCTAATACTAATGAGAGAATATCTACCACCGTT 125326
                * **   *     ** **       ***    *** ** *   ** **    **** ***

Human           TCTAA---AGTTTCTATGACCATCACGAAAAGTCAAGGGCTTTCCATACAAAA---AGTC 118853
Gallid          TGTAATTTAGAGGATGTTCCCAGCCCTCTAAGAGTAGAATTTTGTCTTCTGAACCAAATG 125386
                * ***   **    * *  *** * *   ***   **   ***   * *  **   * *

Human           GCAATAGATTTCGGGAGTGATCCAAAAAATTTAAAATTGAGTTC--TATTTATGTTGGGA 118911
Gallid          GCATCAGGTATGGGCGGTGCTGATTTAAAACTGAGAACACGTGCAATATTCGTATGCCGA 125446
                ***  ** * * **  *** *     ***  * * *    ** *  ****  * *   **

Human           TGT-CACGAGTAACCGACCCGAAT---------AATTTAATAATGAATGTTAATCC--TT 118959
Gallid          TTTACATCATGGTCCGAAATGAACGCTATCGCAAATTCAATAATTTATGGAACGCCAATT 125506
                * * **  *    ****   ***          **** ******  ***  *  **  **

Human           TGAGACTGAATTATGAGAATGATAATTTTATTGCTCCGCATATTGTAAAAGCGTTAAAGA 119019
Gallid          CAAGCCGGTGTTTTACAAGCAACAATATCTGAAACTGAAACGTTCATGTTACATGATGAA 125566
                  ** * *  ** *   *   * *** *           *  **       * * *   *

Human           ACGAAAATACCATGTTAA--TATTTTAGGGAGGTTTTTTATGAGAATATATAAAAAAAAC 119077
Gallid          TTCAACCTTGCTCTTCACGTCTTTCTCAATGGGTTATCTCTGAAGGGTCGTAACAAAAAA 125626
                   **  *  *   * *     ** *     **** * * ***       *** *****

Human           --------TAAAACATCAGATGA--ATTGTGCGTGTA----ATTGTTGAAATTCCATACG 119123
Gallid          GATGTTTGTATGTCATTGAATCACAATTATATATCGAGCGTATCTGAGAATTTCCCAAGG 125686
                        **   ***   ** *  *** *   *  *    **    *** ****  * *

Human           GTCGAATGAG--CTTATTTCCGTCGGTCCGTTTTTTCTGCAAAACA--AAAGTAGTACAG 119179
Gallid          GGTAAACGAGGTCTGACTGGACTCTATTTACAACACGAACAAAAGGTCACAGCAGCATAT 125746
                *   ** ***  ** * *    **  *            *****    * ** ** * *

Human           GGATTTA-GTACAGTATGTCT-CGAAAAAGACAAG--GGTGTGAAACTTTATGAGGAACT 119235
Gallid          CGGCGTATATATGGTGGATCTACTACAACTGCTTTTTGGTACGTGTCCAAATTCGGACCA 125806
                 *   **  **  **   *** * * **   *     ***  *   *   **  *** *

Human           GATGAGAAATGACTTAC---GGCC--ATGATTTTGTTTCGTCGCTAAATTGAATACAGCT 119290
Gallid          GATGAAAAAAGTCTTGTTTTGGCCCTACGTTATTACCTTTTGCAGGCACAGGAAGAAGTT 125866
```

```
                ***** *** * ***     ****  * * * **   *  *      *  * *   ** *

Human           GCAGGGATGTGGTATGAGATGACATCTGCAAAAACAAATGAAAGTCTCCCCCGGAGTACA 119350
Gallid          ACTGGTAT-TGCAACAGGCTATGATCTGCAAG---CCATAAAAGATATATGCAAAACATA 125922
                 * ** ** **  *   * *   ********      ** ****       *  *  * *

Human           CGTATGTACTCCGGGGGAAACCTCAAAAATGCACAATTTA-GCCTATTAAGCAGCCGCGA 119409
Gallid          CGCA-GTGTC----GGTAAATCCCAATCCCACGGGATTTTTGGCTGCCGATTTAACGTCA 125977
                ** * **       ** *** * ***     *   ****  * **    *     **  *

Human           ACTCGTTAATAGTTACTTTGATGTTAACTCAGGTGGTGGCTTGGATGGTGTCCTAAGTAT 119469
Gallid          TTTAGTAGATTATCACGTTTTTGTTGTTTAAGTTACTATTCCAAAGGCTCT-----GTGG 126032
                  * **  **  * ** **  ****   * ** *  *       * * * *     **

Human           CAATCGAAGCTCCTTTATTTTTTGCACTACTGAAGTTATTCTAGGTATCGGGGTAAGAA- 119528
Gallid          CCATAGCATTTCCATCATATGTGGAAC--GCAGGATTATGGCCGATATCGCAGAAGTGGA 126090
                * ** * *  *** * ** * * * **        ****    * *****  * *

Human           -----TAAGATGACGAAAAAAATATGCTAGATCCGTCCATTAAATCCTAGAGTTGTTTTT 119583
Gallid          TGCATTGAGAGAATATATAGAAAGAGACAGACCCAGTTTGAAGATTTCGGATTTGGAATT 126150
                     * ***  *   * *  **   *  *** **       * **    ** ***   **

Human           AGTAATAATAGGT-TATTTACAAAAGCCTGCATATGATC----CAATGTTTCTGTATGAA 119638
Gallid          CGTTA-AATATATATATTTAGCTTATTTTGAATGTTATAACCGCGAACAGTTAAAACGAC 126209
                 ** * ****  * ******    *   ** ** * **     * *    *    * **

Human           TCCAGATAAACAAAAATCCAGGAGTA----ATGATTGGAATCACAAAATG-----ATTAG 119689
Gallid          ATTTGAAAGATGTGACAGTAAGTTTGCCCGATGAAGACATTTACAAGAAGTCTTCACTAG 126269
                    ** * *    *    * *  *     ****    * * **** * *     * ***

Human           TTAGATGCTTA-------ACTTACTTAATAACTACACTTATAGACTAAACTTAACTTATC 119742
Gallid          GCAAGTGTGCAGTAGAAAATTTTTTTACACATGTGAGATCTAGATTGAACGTGAATGACC 126329
                  *  **   *       * **  ***   *    *  * **** * *** * * * * *

Human           AACTATACTTAACTTTTAGGTCCTTATAATGTGAACTAAATAATCAACTGTTTTGTGCCC 119802
Gallid          ACATAGCCCATAATGTATTGCCCGAACAAGTAGAAATGGGAAATAAGCTAGTCCGAA--- 126386
                *  **  *   * * *   * **  * **   *** *    *** * **  *  *

Human           AATCCCCCACTACTAAAGCAATCGTCTTTGATTTATGA--AACAACGAGGCCCACTTCAC 119860
Gallid          AGTTTGGACGTGCCAGAATG--TATCTGTCAACTACGATGACTAACGAGTCGCACTTCAC 126444
                * *       * * * *       *** * *  ** **  *  ****** * ********

Human           GTTATTACATAGATCAAGGTCTATCTCTTGGCGGTTTTTGAATTCTTCCGAGAGCGTAGC 119920
Gallid          TGGAATATGTGAATGTGCATCTGTGATTTTAAAGCGACTGGACACTCTAGAAA----TGA 126500
                   * **  *  **     *** *   **    *    ** *  **   ** *     *

Human           GGTTTCTAGGTTTCAGACACGTTTATCCATTCGCCGGGTATTGATCGGGTCATATCATTA 119980
Gallid          AATTGCAA------AAGTATGGTTGGCCGTCTGATCG-TGTGGATGGTTCCAATCTAATG 126553
                  ** * *      *   * * **  ** *   *  * * * *** *   **    * *

Human           GCCGTGAATAATCTCTGGCTTCATTTCGATACAACGTGTGAAAACTAG------TAAAAA 120034
Gallid          GCCGATAATCAGAAC-AACTCTACTTTAATACCGTATGATAAATCTAGGTCTTCTGGAAT 126612
                ****  *** *   *   **  * **  ****    **  *** ****      *  **

Human           GG-ACAAAAGCAGCCGCCTTTGCCGTCTTCATGAGCGTCAA--ACACTCATGAGGTCCGT 120091
Gallid          GATACTCGAGTGTTCGAACACTCATTCTCGAGGGGGGCCGATGATAGTTAAAAGGTTATT 126672
                *  **   **    **      *  ***  * * * * * *  * * * *  ****   *

Human           ATCTTACAACACAATATATGAAATACTGTGAGCGAGGTTACCGGTGTCCGAGTCGCAATC 120151
Gallid          AGCTT------TAGTATCTGCCGATTCTCGCGCAGGGGGAATCG-GCCCAGCTAACATGC 126725
                * ***       * *** **         * **  **  *   * * **   *  **  *

Human           CGAAAATCATATATTGAAATTTGTAACAATGTTATGCAGTTGTACTCAAAGTATATGGTG 120211
Gallid          TCATGGGGATTGACTCTGCAATAGATGGACCCCTTCCAGTT-TACCGTGTGGGCATGTCA 126784
                  *     **  * *      *  *   *     * ***** ***     *   ***

Human           ATGAATTGA-ACATGTTTTTGATGTTTTCAATGG--TATTCGAAAAATATT-TTAGATAT 120267
Gallid          AAGGGCAGACAGGCTTTTACGGTGCTTATGACCGAATGTTGGGAAAGGACCATTCCATCT 126844
```

```
                * *    ** *    ***  * ** **   *  *  * ** * ***  *   **  ** *

Human           CCTGTAGTTTT-------TTTCTGATGACTTTTCTGATGACTTTTCTGATGACAATATAG 120320
Gallid          CCGGGAAGTGCGAAAGCGCATTTGATCAAGCT--TAACAACTCTTACGGTACTTCGACAG 126902
                ** * *  *           * **** *   *  * *  *** **  * *      * **

Human           AAGA--TGGCAAAACAACAAGTTACTGGTAGTTCTTGATGTTAGATAAAATGAATCGGAG 120378
Gallid          AAGACTTGATTTCACGA-GACTTATTCCTAACTTCTGAAATCGAACAGCTT--ATCGGAA 126959
                ****  **     ** *  * *** *  **  *  ***  *   * *   *  ******

Human           ATATGACA-AGTAGAGAGAGGTAGGTGATACGAGTAAAATTGAAGGGTGTT-TCCACACA 120436
Gallid          GCACAGTAGAATTGCCGGAGATTACATGTGGCTCTGCCGATGAACAGCAATATATAAACC 127019
                  *    * * * *   *** *      *     *     ****  *   * *  * **

Human           GACGGGGGAAATTCTATGAGAACCACA-GCCAGAAGCAATTGACGACAATAGAGAAA-AT 120494
Gallid          GCAATGAAGTCTTTAATGGGAATCTTGCGATAGGAAATATAGTTTTAGATGTGGATATAC 127079
                *    *     **  *** *** *    *  ** *   ** *      **   ** * *

Human           AAGCAAGCATACTCATCTCGTATAGAAAGAACCAGTGACC--TACGATTTTCCATTCAGG 120552
Gallid          ATTTAAGAAACCCCATACCTCTTAGACTTATGCATGCAGCGATACGAGGTTTTA---GAA 127136
                *   *** *  * ***  *   ****   *  **   * *  *****  **  *

Human           CTAGCATGGTGTGAACGAGGGTTTTTTCCGAAAACTAGATGACTATTTCTTTAAGCAAGT 120612
Gallid          GTGGTATACTCAGAGCTTTGGCCTTATTGCTACCAAAGGCAAATATCGACCATGGCTCAT 127196
                 * * **  *  ** *   **  ** *    *    **   * ***        **   *

Human           GTAATTTTTAGCAGTCCAAGTTTTTATGCAACG----CAGATTTTAATGATGTGTCCCTC 120668
Gallid          ACCCGTGTTACTTTTATAAGAGTTCGTGCAAGAAATCTAGAGTAATGGGGGGAGCGCCTT 127256
                     * ***    *  ***  **  *****       *** *     *  * *  ***

Human           GATAACTATACGCTG--GAAACTAAACTAACTTACATCTTGGTTGATAA-ACGATTTTCT 120725
Gallid          GGATGCTCCATGATGCAGAACTTGCCCCAGATTATTCGATGTTTGAAAATGCGGAGTTTG 127316
                *    **  * * **  ***  *   * *  ***     ** **** **  **   **

Human           ATTAAAAA---GACTAAAGGGGTTTTTTTGGCATATACAAAATTGTATTGAGCTCTTG-- 120780
Gallid          ATTTAGAAATGGGCATAGATGACCCTTTACTCATAGACCAAATAG-ATGAATCTCTTACT 127375
                *** * **   * *  *   *    ***   **** ** **** * **  * *****

Human           ----GAAGTGCGTAGATGCAAAATAG-GTTAAATCTTACCTTGA--AGCTACAACAGGTC 120833
Gallid          AGATGGAGCTCAGAATCATCAAGGAGTGTCGATTTGGATCCAGATAAGCCATGCGGTTGC 127435
                    * **  *  *      **  ** **  * *   * *  **  *** *        *

Human           AACGTTCGAGT-AGACGTAAGTTCCATATGTCGA-ATGCGCTGTAAACTTTTTCACGGAA 120891
Gallid          CATGATAAAATCGGATTGAGGGTTTGCATTCCAGTACCCTCTCCATATTTACTTGTGGGT 127495
                 * * *  * *  **   * * *    **  *   *  * **  * * **  *   **

Human           CATCAAAAAC--ACCGAATTTATTCACGT--TTACATAAGCATGAATCT------GCAAT 120941
Gallid          AGCAAGACATTGGCCGGATTGTCTCGAATCATTCAACAAGCCGTCCTCTTAGAGCGCAAT 127555
                    * * *    *** ***   **   *  **  * ****     ***      *****

Human           TTTAATAGTCCCATAAAGCTTTC--------ATTTCGATCTAACTACATTTTAGTATTTA 120993
Gallid          TTTGTAGAAACTATAGGGCCATATCTGAAAAATTATGAGATAATTG-ATAGTGGCGTATA 127614
                ***       * ***  **  *         ***  **  *** *  **  * *  * **

Human           TGCTCCATAATGTATCAAAGTGTATACATTCAATCTGTATAA------GAGTCTGCATCA 121047
Gallid          TGGTCATGGGCGTAGCTTA-CGTCTGCCGTTTTTTGGCAAAATTGATGAAAACGGTATCG 127673
                ** **      *** *  *  ** * *  *   *  * * **       *  * * ***

Human           AGCTTCACGTGATTTGTAGTTCGCGGTTTAGCATTCGCTGTTT-TGCCGTGTATGAGACT 121106
Gallid          TG-TCTAGAAGACTTGTACCGTTTTTCGTGATACCAGATGATTGTGCTGACATGGAGAAG 127732
                 * *  *   ** *****          *   *   * ** ** *** *     ****

Human           TACAGTTTGATCCCTAATACTTCTGACGGGCGACAAGCCTTTTTATATGCATTTTACGGT 121166
Gallid          TTTATTGTGGCCCATTTCGAACCTAAAAACTTCCA--TTTTCACAGCTCTATCCCGCTAG 127790
                *  * * **  ** *       ** *       **    **   *  *  **    *

Human           AAAGTCCACGCCCTTGTCGCTGACGCAATACGTAAAGGTTTT-CGGTTTGGATGAGGATG 121225
Gallid          AAAAGGC-CGCCATAATTCTGAAAGATATAGGTGGCGAATATGCAGGTTTCTTCGAAAGA 127849
```

```
                ***   * **** *  *     * *  *** **   *  * * * * **   *    *

Human           TGGTTAACCGTTAGTAAAAAGTCGTTACAGTAAAAAAGCCGAACGGTATTTTTAGATGTC 121285
Gallid          AAAATTACAGTAAATAGAGATATATTTTTCGGGACTCGATTATCTTTATCAATAGCTCTC 127909
                    * ** ** * ** * *    **       *   *   * *  ***   *** * **

Human           GGACCACATATCGGTTTAATAGATTTTTATATCGTGTTCGTTGTCTGACTTAGATATGTC 121345
Gallid          AGGGAAAGGGGGGTAGATATAAATGATTGTGCTGCCATTACAACATTTGTAACAGATCAC 127969
                 *   *      *     *** **  ** *   *   *       *   * * * **  *

Human           GTTTCATGGGAGAA-ATAA-ATCGTTTTAGTTGTGTGCTGTAGTTTTTTGGATCGCAAAC 121403
Gallid          ATTTTAGATGATATTATAACATACGTATATGAGCATATACCAGATCACGCAATCG--AAT 128027
                 *** *   ** *  **** **   * **   *  *     ** *      ****  **

Human           GCTCGAGCCCGGAAGTGTGAT-TCGTGCGCATAAAAGGGCGCTGGTGTCTGTGCTCGCTA 121462
Gallid          ATCAAAATCTTTCTGTCTCGTGTTGTGT-TGTCAAATCGGATTGGATCCTGCTGCAGCTA 128086
                     *  *     ** *  * * ***    * ***  *   ***   ***     ****

Human           G--CTCATTTGTGCTGGATGA-GCGGCAAAGAACTCATCTTTCTGAGATTTTTCTGGGAT 121519
Gallid          ATCCCCAATAAAACAATAGGATATCGTCACGGGTTTACATGTGTGAGATTTA---AGCAT 128143
                   * ** *     *   * **     *  * *   * *  * * ********     * **

Human           TCTTTTGGTAAAAATGTATGT-CTCGTTTGTAGTCGTTTCTGGATTTCTGTA---ATGTT 121575
Gallid          GCAAGAGCAAGGCGAGCGAGTGCACGTTCTTATTTGGCTCTGAACGTCGATGCGCATGGT 128203
                 *    *  *      *   ** * ****  ** * *  **** *  **  *    *** *

Human           ACGATTTGC-TATGTGTATTTTGATGGTGT--------------CGAGCATGTTTATTTT 121620
Gallid          AGGTTGTGCGTATGTGTAATTCAACAGTGTTTTGCGGCCAAGTGCGGAAATAATAAACTT 128263
                * * * *** ******** **  *  ****              **   **  * *  **

Human           GTTCTTATTTTCACA-CAGATAAGATGT----ATGCTGA--AGAACGTGGATATGGGTCA 121673
Gallid          CGCACACTTTTCACGGTAGATATTGACTCGAAATGTCGATTAGAACATCAATAGCTCTCA 128323
                       *******   *****     *    ***  **  ***** *  ***    ***

Human           TTTGA----------CAATGT-----TATACAGGCTTATGAACAAATTATTAGTCAGTCT 121718
Gallid          TCGGAATTTTATTTGCTATATCGATATATACTGTTTTATTGGTAGTTTATGTATCGA-CG 128382
                *  **          * ** *     ***** *  ****    *  ****   **   *

Human           CTTCATTTGAAAAGGTTTGAATTCGACAATGGCTGTTTCATTGAGTTCTTAGCTGACTCT 121778
Gallid          CTTTCCCAAAATGGATCTGGATGTATCTATG-CTACCTTGGTGGA----CAGTAGCCTCT 128437
                ***      **  * * ** **    * *** **   *   **        **  * ****

Human           GGAACGTGTGAAACGTTTTCTAAAGGATGGATATCAATGATTTATTGGACATCGGAGACA 121838
Gallid          ATGACGCCAAAAACTTCACGTGGGAACAATATAATTCTACCTTGATATATACAGCACTGG 128497
                   ***    **** *    *          ***    *   **  *  * *  * *

Human           GATTCAATGGGTTCTCTAACGGTGGATATTGGGATG--GATGAAGGAAAATGCAGA-ACG 121895
Gallid          GGAATAAATTGCCTCTGGATGGTGGATTTGACGATTTCAGCGACGTATGTCGTACATATC 128557
                *    **   *       * ******* *   ***      ** * *    * * * *

Human           TACAGAGCTCGGGGCCTTTTACTGTGTTCAAAATCAATCACGTCGATTT--CTCAGAATA 121953
Gallid          TAGTCAACTTAACGTCTATTTCCGGACTCGCT-TCACACGTTTCTACCAAGCCCAAGATT 128616
                **    * **    * ** ** * *   **    ***  *   ** *      * **  **

Human           CTGAGGGCAGAGAGAGAGGATTTTGACTGTTTCCCATGAAAACGGAAAACTT-CAAATAACG 122012
Gallid          CGATCGGTAGTAGGAAC-CCGCAATTGTGTGACGTATCTTTGGAGGATACATATACAATC 128675
                *    ** **   **         * *** *  * *       ***  *     * * **

Human           TTTGTTACTATTGCTAAAGTTTCCCCGGAG----CACGAGCTACGAAATCTGGGCGATTT 122068
Gallid          TTTGTCCTCCTCTCTGGGGTTGTATACAATATTTTATGTCATTCGTGAATGGAGACGCAT 128735
                *****     *  **   ***        *      * *   * **  *    * *     *

Human           GAAATTTATGGAAAAGTTTGAAAAAGA----ATGTCGAGCTCTAGACAGG--AAAAAACA 122122
Gallid          GTTTGGTGTAGTAAGATTTGAGGATGATGCTATATCGACAGCAAGGTATACCAAAAATTA 128795
                *      * * * **  *****  * **     ** ****   * **  *     *****  *

Human           CGACGAT--GATCACAGAAAACGCTCGGGGAAGCAGAAAGAAAAAAGAAAAGTGGAGGAC 122180
Gallid          TGCCGCCCGAGTGATATCTAGTGTTTTACTCAACACAACCTACACTAAAATGTCTAGATT 128855
```

```
                 * **      * * *   *  * *      * ** **   * *   *** **  **

Human           ATTGACAAAAAAAAAGAAGATGAGAAACGA---AAACAAGAAGAAAAAAAACGAAACGAC 122237
Gallid          TATGTGCGAGATAATGATCTATAAAAATGCTTTGAGTAGGACTTTTAAAGATGATCCCAT 128915
                  **    * * ** **     * *** *     *  * **     *** * **  * *

Human           GAAGAC---AAACGGCCGGACAAAAAGGATGAATTTGATGGTAAATATGAATGTGTAGCT 122294
Gallid          ATCATTTTTGTTTCATCACCCTATCGCAGCAGTTCTTATTATTACTGAGGGTTTAGTGCG 128975
                                *   * *          * * **  * * *  *  * *   **

Human           GTTGTAAATTTATTTTTGGTGTGTCACGGT--TGTGGACTT-TTTTAAATAGTATCT--- 122348
Gallid          ATTAGGGGCTCAGTGTCTTTGTCTAGCGACACTATCGATGTATTTTGTACCATGTGAAAA 129035
                 **      * * * *   *** *  **    * * **  * ****  *   * *

Human           -GTTTTGTTGCGAT--TTCAGAACCCCCTAAGGAGAAGAGACAGAAATC----TCACCAC 122401
Gallid          AGTTCTCTCGAAATGGTTTTTATCCATAACAGGCATTTTTATAGGAATTATAATCTGTAT 129095
                 *** * * *  **  **   * **     ***       * ** ***     **   *

Human           GAAACGAAACGTAATTTGGAA----GAACAAAGTCACGAGGATGGCATAGCACCGACTTC 122457
Gallid          AGAATTGAGCTTGTTATTGGCTCCCGGCCCAGTTGATGGAGCTGCCATGTTGGGAGAAAC 129155
                  **   * * *  * * *      *  * *  * * *  * ** ***           *

Human           TACGACATTCGTGAATG------GAGCGGTTGAGGGTGCGTTATC-GCCCTGTGTTTCTA 122510
Gallid          AAAGCAAGTTAAGAAAGATGAATGTGCCTTGGAAACTTCCCCATCTGGCGTACATGTTTT 129215
                 * *  * *   *** *      * **  * **   * *   *** * * *   * * *

Human           TTG-----ATAATCACGAAGATCAACAACATGATGAATTAGACAAGCGCGTTTATGCGCA 122565
Gallid          TTGCTCCAATTGTTGCGCCTCTTTAATATCTAACATATTAATCAAAGTGCTGTATATATT 129275
                ***     **  *  **    *  *  *  * *   ****  ***     * ***

Human           GGTGGGTGGAGTTTTGGGTTCACCAAAACTTAGGTCTT--TGGAGTCTTTGTTGTGCGTA 122623
Gallid          GTTCATGATAATATTGATTGTAACTATCGTAAGATATGAACGAACGCTTCAAATTGCATT 129335
                * *      * * ***  *  * * *   * ** * *    * *  ***     *** *

Human           TCTAAAGCTGATCTTTTTCTTTTAGGGGACGAA--------CCGAGAAGGTCTATCCAAT 122675
Gallid          GTTTGGGCGTGCCTATTTGCCTTAGTATACTTAACTCATCTCTTAGATGATTTGCATTGC 129395
                  *   **    ** ***   ****   **  *        *  *** * * *

Human           GAAGATAATTATGGAAATTTTCAGCTTA-ATAAGTCTTTAGAACAGCTTAGG-GCCAGAC 122733
Gallid          GCGGATA-TGACATAACCTACGGGGTTATATAGGTTGATATAGAGCTATAGGAGATTGTC 129454
                *  **** * *   **  *    * *** *** **   ** *      **** *   * *

Human           TTGTTGCAAGCAGCGGCGAGGTTGT-AGAAAGATCGCTTTCGAAATTGAAAGAGCGTTTG 122792
Gallid          TCCTCAAGTGTACCTAATGGACTATTATCTATATCAAGATT-AAACAAAAAAAATGTCTG 129513
                *  *     * * *     *  * * *   * ***    *  ***   *** *  ** **

Human           GA------TTATGTAAAGGAT--AATTTAATAAAAAATGTACTTGAATGTGCTGATGT-- 122842
Gallid          TAGATGCATTCTCTCGCGAGTCCGATGACATGATGAGTTTGTTGGACTATGATTTTATAG 129573
                 *      ** * *   *  *   **   ** *  * * *  * ** * ** *  * *

Human           -----TACTGTTCCAA-GTAAATGTTTAAGTAAAACAAAACATATCGAGCAAAAGAAACA 122896
Gallid          AAGGATCCTCCTCCGATGAAAATGCCGAAGTGAC-TGAAATGGAAACATCTGCAAAAACG 129632
                     * **  *** * * *****   **** *    ***   *   * *   * ****

Human           GATAGTGTTTTCTGATTGTGTCAGGTCGGTACCTGTGTGTGAGATTAAACCGTTTATCGA 122956
Gallid          GCTAATAACAAG-AATGAAGTTTTATTCGCGCCACCGTGTACGCAGGAACTTTTGACCGA 129691
                * ** *        **   **    *  *  **   ****  *    ***  ** * ***

Human           CATGCGAGTATTTGAAACTGAAACAACGCAGAACG-CAAGAA---GAGTTCGACAACGGA 123012
Gallid          ACGACCATCTCCTGATTCCAAAAATTCGCAAGGCGACGATGACTCAAATTCAATATATGG 129751
                    * *     ***  *  ***   ****   ** * *  *    * *** * *   *

Human           CCAGAACAACCGTCG-GATCAACAGATGGTGCAATCGG---GCAGCAGCGTGTTATTTCA 123068
Gallid          CAACGTGATTCGTGATGCTCAACACTCAGCA-AGTCGATATGCTACAAGGTGTCTTGACA 129810
                * *    *  ***   * ******    *   * ***    **  **  ****  *  **

Human           GGACAAAACCGGGGCAGAGCACGAGGACGTGGCCGAGG-GAGAGTTCCCAGGAGACGGAA 123127
Gallid          ATGCAATACCACGG--AAACGTCTACGCTTAGCTAATTTGACAGTAGATTCTGCATGCAT 129868
```

```
                   *** ***  **   * *       * * **  *   ** ***         * * *

Human           TTCC-AATCTAAATAATTTAAGGACACAA--AATTATGCA-ATTGTCATAGACGATAGCA 123183
Gallid          TTCCCAAACTAAACGGCCGCACGGTACAGGCAATCGCAAACAATATCACAGACGTAATTT 129928
                **** ** *****       * *  ***   ***     * * * *** *****  *

Human           GC-GAAACCGAAAACTTTGAAAATGCTGGGAGTTTTAATGAAGACT-TACTGGCAACCAC 123241
Gallid          TCCGATGTCACCGACTTCACAAG----AAAAAATTCATCTACGATTGCACAACCGACTTG 129984
                 * **   *    ****   **         *  ** *   * ** *  **   * **

Human           AATATTGGAAACACTGTGAATTTTATTG-CTGTATTGTAATTCTTATGTAAACTAAATAG 123300
Gallid          GATCTCGGAGCGAAAAACAGCAGCGCAGTCTAAATTACGA--CCGACGTCTGCAAGAAGG 130042
                 ** * ***   *     *         * **  ***   *  *  * **   * * *  *

Human           GTTCTATTCGTTCAGAATGTTCCAATTGATTGGGTCTTTCCCGTGCTCAGTAAGAAATAA 123360
Gallid          GCATCAC-CGAAGAAGATTCTACAGTGAGAGACGTATTTATGAT-CAAAATCATAGTCAC 130100
                *    *  **   *  **  * ** *        ** ***    * *  * * * *   *

Human           ATTTGTTCGCACAAAAT--GATTGCAACCAATAAA---TGGCATTCTTGCA------GAT 123409
Gallid          CATCGTACACACGATATACGGGTACCATTGGAAAAATATAGAGTTTCCAGACAACATGAT 130160
                  * ** * *** * **  *   * * *     ***   * *  **     *      ***

Human           TTCACTTTGGGTGATGGAT------GTGCACTTTCTAAGACGAGATGTTTCTGTGCGTCT 123463
Gallid          CTCCCTGTCCATGAGGAACTAAACGAAATACTTCAAAGAGAGAAACACCGTCTGGCCTCT 130220
                 ** ** *   *** * *           ****   *    ** *          ** ***

Human           ATT--AGATAAGAGAGTTTCCGGGCATGTTTTCCCCACAACAAGA-AAACGAGAGAGTCC 123520
Gallid          ATTTCAAATGAGTGTGATTTT---CGCGTTTCGAGCAAAAATCGATGGGCTGCCGTATTA 130277
                ***  * ** ** * * **      *  ****    ** **   **    *    *  *

Human           ATTTGTTCCGATAGTCTGCTGATAATCTTGTAGCTCAGTGTTTGCCAACCAAATGCCTCG 123580
Gallid          ACATTTTCAAGCAAC--GCGGAGAGTACTTTATGTGGTCCTCAGATAACATGGGAGTATT 130335
                *  * ***    *    ** ** * *  * **  *     *  *  ***

Human           TGGGACATTGGTAATCCATGAACTACAGTGAATAT-CGAGTTTAGCAGTAGGACTCCTTC 123639
Gallid          TATTGCATGCG-GGTCCA-GAGCTACGAAACACGTTCGAAATCAGACCTAGAATATCGCT 130393
                *    ***  *   **** ** ****     *  * ***  * **   *** *   *

Human           TCTA-CACCAGGAGTTTAAACAGCCGTTGTCAGGGGGGAAAAAATTCGGGATGCTTCTTT 123698
Gallid          ACAAGCAAGTGCAGCACGAGAAGCCGT-GTTGCGAGGTGAAAGTTTCAT--TGCCGCATT 130450
                 * * **   * **     *  ****** **   * **  ***  ***    ***  * **

Human           CCAGTTCTTTAAATATATTCTTTAGAGATTCTGGAATCGAGCACTCTTTCACTGTACCGA 123758
Gallid          AGGGAGTGCTGAAGAAACTCTGTCGTGGT--TAAAACTACATGCTGTTTTAAAGTTACGC 130508
                   *     * ** * * *** * * * *  *  **       ** *** *  **  **

Human           AGGCCAAGCCGCAACCTCTGCCGTCGGGATACGGATCCTGTCCCAGA--ATCACAACTTT 123816
Gallid          CTAGTAAATCATGACCCGATTTTTAAGACCGCTGGTGCGGTTTTAGATAACCTCAGGCTG 130568
                     **  *   ***       *  *    * * * * **   ***  * * **   *

Human           AACATCG-------GAGGGTTTGCACAAAAAAACTCCAGG--------CGTGGACGTTATC 123861
Gallid          AAGCTCGCACCAATAATGATGTGTAAATATGGAACAGAGAAACGCTCCATGGGGGATATG 130628
                **  ***         * * * ** * * *     *   *        * ***  * ***

Human           CGAAGAGGGG-------TGGACGGTTAGATGAGCTCT-ATCGTTGTCCACCAATTTATAA 123913
Gallid          TTAAGAAGATCTGCTCCTGAAGATATAAACGATTCCTTAACTCTGTGCTTAATTTTGTTA 130688
                  **** *         ** *    ** * **   ** * *  *** *   * *** * *

Human           ACATGCTTAAGATGTGTAATTTCTAAC---TTGG---------AGAGTTGCAGGAAA--- 123958
Gallid          TCGCGCATTCGTCGTGTGATGCATCGCACATCGGGCAGCAAATACAGTTATATGATAGAC 130748
                 *  ** *  *  **** **   *  *   * **          * ****  * ** *

Human           CTCAACCAGTCTCTGTT------TATACC-GAAGAGTGAATGCTGATCAT------CTAT 124005
Gallid          CCTAGAGGATGTATGATAGACTATGTACCTGGAGAATGTATGACAAATATACTACGTTAT 130808
                *  *     * * ** *      * **** * *** ** ***   *  **       ***

Human           TGAAAGGTTTTCAT-AGTTTTTTTCTTCATCCTGAACGTGATCTA-ACATCCACTGTAGT 124063
Gallid          GTAGATGCGCATACGAGGAGATGTTCTGATCCCGCATGTAACTTGTATATCAGCTGCACA 130868
```

```
                  * * *     *  **     * *  * **** * * ** *  *  * ***  *** *

Human           AGGGCCATCGTTGGGAAATGAAATGCAGTGGATTAAATTCCCTAGGAGCATTT--TATGT 124121
Gallid          CTCATGCCTATTT--ATATCCATGGCAGGTATTTTTACTGCAATACTCTGTTTGGTATGT 130926
                          **   * **  *  ****     **  * * *         ***  *****

Human           GAATGGGACGTGATGTCATAGCCAATTATGTGT-TTCTAATCAGAATCGTAGGGCGGGAA 124180
Gallid          AAATAGTTATCTAAAAGACATCCTATATTTAGTATTCTACACAATTTCTTCTGACGATA- 130985
                 *** *      *    * * ** **  *  ** *****  **   ** *  * **  *

Human           CTTCGGAAAGGTAGTTTAGAATTGTAGGCTTCCATCTGTTTTAG-CAGGTCGAATTCTAC 124239
Gallid          --TTACTAACTCCTCTAATAAAGTTAAATAAATAAACGTCTCAGATATGTCTTGTTAAAG 131043
                  *    **      * * **   **        *   ** * **  * ***   **  *

Human           TCCGGTTTTCAAGTGGAAATACTTTAAAC----AATTCAAAATCGTATCAAAGAGAGTCA 124295
Gallid          TGTGGTTTTATTATCTATATATCACCGACTTTAGATACGGAAT-ATGAAAATGATGGCCC 131102
                *  ******    *  * ***       **      ** *  ***  *   ** **  * *

Human           AAGAGTTAAAAGGGAATCTG----ATAATTTTTGTGATGCCTGTAGCAGGATTCTCCAAG 124351
Gallid          TGAAATTGCACGAACAGCTGTGGTGAAGATTCCGTCAAATTTACATTGAAATTTAAGTA 131162
                   * **  * *   * ***       *  **  ** *     *  *   * * * *

Human           TATAA---TAAAAAACC----AATGTTGCTGTAAAAACTGTCT-GTGTTTTCTGAAATGT 124403
Gallid          TATAAATTCAGGTGATCCTATAACATCGTTAAACAAGCTCTCGAGTAGTTTAATGAACGC 131222
                *****    *    * *    **  * * *  * ** ** **  **  ***    ** *

Human           TAAAC--TCGAGACCTGCAATGTTATCAACAAAAAAGTT-ATTGACGTACGTAATTCTTT 124460
Gallid          TAAACATTGAAGTCCACCAGGGCGATCACAAAGGCAGTTGACTAACATGCCATGCGCTCC 131282
                *****  *  ** **  **  *  ****  **   **** * * ** * *      **

Human           CAGGTAAATATTTTCTACAGTTATCGCTGATGCAGGTTGTTATTCCGACGGAAGGCGAAC 124520
Gallid          AGGATTGTCGCGAACTGCCGAC-TCGCATAAACAAATAATTTTTCT--CGGAT--CTCTC 131337
                  * *         ** * *   ****  *  **  *  ** ***   ****   *   *

Human           ATGTCTGGAAGCCATTGAAAAAGTTTAGCCAATTGTCCCGAGATTCTGGGTCCCCTATCA 124580
Gallid          ATTTCCAGAAATCTACGCGGGTCCATCAGCAATGCCGGTTTTATTCCTGGAACACAGTTA 131397
                ** **  ***  *   *       *   ****         ****  **  * *  * *

Human           G-CGCAAACAATGTGCGTAGTTGCTCTGGGTTATCCTGTAGAAGTAAGAAGTCGCTGAGG 124639
Gallid          GACGTAAACTGCTCGAA-AACTGTTTTAAGCACAACCG-GGATGTCTGCAAAAGCCGATG 131455
                * ** ****     *   *  ** * *  *     * *  ** **  * *   ** ** *

Human           AATTCATTGGTTACCATTATACGGCTAAAAGGCTT-----TTTTTTTGTTGGGGGTTTTA 124694
Gallid          ATGCTTCCTTATAACAGCGCTCAGTTAGAAACCATAACAGTTCTTTTGGTAGTATATTTC 131515
                *          ** **     * * ** **  * *     ** ***** * *    ***

Human           GCAGTATTGAC-ATAACGTCTTCATTA-TATACCGGAGGCTGGTAGATTGGTTCTAG--T 124750
Gallid          TTCCTAGAAACCATGGCGTTCCCATCGCTAAGAACCAAGCATGTGTTTTCACTGTATCTT 131575
                    **   ** **  ***   ***   **       * **  **   **   * **   *

Human           AATAGAGGTACTTCGGATCTCGTGCAATTTATTACAGCTGCGATAC---AGTCGTCAAGT 124807
Gallid          GTCCAAAGAGCCCTCTATTCGGAGATGTATGTTGGAGATCGGATGTCCAGAGTACTGCTT 131635
                     * *  *      **   * *   * * **  ** *  ***                *

Human           TTTTCAAAATAGAAAGAATTATGATCGAAAAGAGGCATCGCTTTCGAGAAGGTTAATAAT 124867
Gallid          TATCTCGCATAGATGAGGTCGCAGTTACAA----TTCTCCCTTTCCAGCCACCCGTCAAC 131691
                * *     *****      *      *   **        ** ***** **         **

Human           ATGAGTGACATTACAAATAGTAAAAGTTCCATGGTTATTTTTTATTTGGAGAAGTTAACA 124927
Gallid          ATGATGTTTATTG-GACTACCAACA--TTGGTAATGAGTACTGGTCTGGCGA--CTATTC 131746
                ****     ***   * **  ** *  *   *  * * *  *  * *** **    **

Human           CGACGGGAACAACATCTTATATACAGTAGGGAAAAAGACTTGTCGAAATGTTCATTTGGC 124987
Gallid          CATATGCATTAGTCATTGAAATTTCTCGTGGAATCAGCCAAACTGGAGAATCTGCACAA- 131805
                *    * *  *     * * **       ****  ** *      * *   *

Human           TTTTTATTTTTTTTTATGCGGCATATCTTGGTATGGCTATCGGATT-CATCGGTAGTTCC 125046
Gallid          TCTTTATTATTCGATGTGGAATG-ACCATGATCGCTTTGTTGGTCTGTACCGTTAGCAGA 131864
```

```
                 * ****** **   * **      * * ** *     * * **  *  * ** ***

Human            CCCGATGCGGAGCTGTCTTCCGAAAATTCACGTATTTCGTCTTCTGTCTTATCAGGATG- 125105
Gallid           CAT-ATTCAGAGTAATGTCACGAAGACTTAAGGGTGGC-TTTCATTTCAAACCTGGAGAA 131922
                 *   ** * ***   * *  **** * * * *  *  * * *  * **  * * ***

Human            TTTGTTGTGTTGCACAGATTGGTCCGCTGTCGTACCTGGGAAGACAGAGACTTTCAGAAA 125165
Gallid           CTTGTAGGGTTGTATAG-TCGACAGGATGTAGGTGGAGACATGACGTCTACAAAGAGACA 131981
                  **** * **** * ** * *    * *** *     *  * ***    **    *** *

Human            ACCTTTTGTTGCAATCATGATTAAAAAGCTAAAAAGTTGTTTTGCTGCTTACCTGTCTGA 125225
Gallid           TCCTATAAGGCCATTCCTG--CAGAAACTTATAGGTTTATACCAGAGACGACTCAATGGC 132039
                  *** *     ** ** **   * ***  ** *   ** *      *   **      *

Human            TTTAGAGCAGGGCTCGACGTGTGATATGGCAAACGCATCGCCGACAAGTCTTGAATTAGG 125285
Gallid           AGCAGGGGCGATGTCATCGTCAACATTGGCTCAAATACCG--AATGTATACCAAGTTATT 132097
                   ** *  *   **  ***      ****  *   * **   *    *    * ***

Human            ATTGTCGAAATTAGACAAAGAATCATGAAACTATTCGAAGACATTAATACTGGTTTTCTT 125345
Gallid           GATCCCTTAGCGAT-TGATACATCGTCGACTTCTACAAAACGATTACTGGATGAACCTGT 132156
                   *  *  *   *    *   *** *  *  * * * **   **** *    *      *

Human            TCTCGTTATGACGATCAATATAGAGGACGCAAA------GAGAGACCAATTTAGATTC-- 125397
Gallid           ACCACACATAGGATCCATTACATCCCGTTCATATCTGCTGAGAGTAAAGTGTAGTTCTCC 132216
                  *     **      ** ** *        ** *      *****   * * *** *

Human            --GAGCCAAAAAAGTTTATTTTGTTAGTTTAATA------GTTTGCCATAAT-CATCCCA 125448
Gallid           TGAAGATTGTCACGCGTTTTTCTTTGGTCTGGCAACGGAAGCATCTGGGAATATGTCTCA 132276
                   **       * *  * ***  ** ** *   *       *  *     ***   ** **

Human            ATAAA----TATATTTGTTGAAAGAGTTGTATATGGTGATGTGTTCTC-GAAGGAGCATC 125503
Gallid           GCATGGCGCCGAATATATTGCACGCAGCATGAACGAGAAACTATACACAGGACGAAGTGC 132336
                   *         ** * *** * *     *  * *   *  * * * * * * **    *

Human            GGTTGTGTTA-GCTTCGCGATATT----CAGAGTAATCTCGTTGGGATGGCAGATTGCAA 125558
Gallid           GGACGAATTATGCCATACACCATTTTCCCACGCTACTATTCTGGATTCGTTAGATGACAA 132396
                 **  *  *** **     *   ***    **   ** * *  * *    *  ****  ***

Human            AATTGATACAAACGTGAGCGG--CGCATAATAAATCTTTTTCCGTCGGCGCGTTTATCAT 125616
Gallid           TTATACTCTAAATATTGAAGGACTGTGTTATCACTGCCATTGTGAAAACAAGTTT-TCAC 132455
                   *  *  ***  *    **   *  * ** * *    **  *    *  **** ***

Human            GAGAGAGCAAATGCCGACAAGCTTTTTTTTTGAGGCGTTTACCGGTATAGGAGCTACAGC 125676
Gallid           ACGAGTGT---TGGAAAGCAGCTTTTATTGCGGGAGAAAAGATGGCGTTACT-TTGCAAG 132511
                   *** *    **   *  ******* **  * *         **  *      * **

Human            GTTTTTTCGAAGTGC-GTCCGTATGTGGGACGGTCCCTCTAAAAA-ACTG---ATTGATT 125731
Gallid           GATCTGCGAATGTACTGTCATTAAATAAAACCGTACACCAGAATTTACTGTGCATGCATT 132571
                 * * *    * ** * ***  **  *   ** ** *  *  **   ****   **  ***

Human            GCAATAGCGACTACCGGTTCATGGTGA-ACCGATTCAGAAATTA--TTTGCTCGCCCAC- 125787
Gallid           TTGTTTTTAATTTGAGTCTAGCATTTACATTAGCTGAATTATTAAACTTACTCGATAACA 132631
                     *    * *   *  *     * * *     * *   ****   ** ****   **

Human            GTTAATAACCATGTTGCCAATGGACATCAATT-TTTCTTGCATGGCGTTTATTAAGATTT 125846
Gallid           GTTACGTATTATATGTCAATTTTATGATAACAATCTGATACATA-TGTTAATTGGAATAT 132690
                 ****   *  ** *  * * * *  *    **   * *  * ***   *** ***   ** *

Human            -TATTTCGGGAATAAACCTGTATTTGCA--CAATTGAAGCGT-TGTAACAGCGGAA---T 125899
Gallid           ACACTGCTATAGTTTACGGGCATTCGAAAATAATCCTAACACATATAACGGTAAATCTTT 132750
                   * * *   * *  **  * *** * *   ***   * *   * **** *   *     *

Human            AATCTGTAA-ATTTCTGACATAAAATTTTTAGGTGAGCACGGGGA---GTAAGGTGGAGA 125955
Gallid           CATTTCTAATACCGTCCGGATAAATTTTACGAGTTTGTAGATGTCTTTGCATGTTAGAAA 132810
                  ** * *** *         ***** ***    **  * *   *     * * * * ** *

Human            T--GTTTCG-TAAGATTGAAAAATT--TTGAAA-GCGTTGACTGCATTCGGATCTTGCCT 126009
Gallid           TTCATTTCAATGAGATTCTTTCGTCCGTTGAAGTGAGAGGGAAAAAAAAAGATCATGCTT 132870
```

```
                *   ****  * *****       *   *****  * *  *      *    **** *** *

Human           TTGGCATTCCG-TAGACTTGACAATTTCCTTTTTCT---CCGTCGTTGATGTCTTTACAG 126065
Gallid          GTCCAATTCAGACTCGCTGGTCAATCTACTGTTGTTGGTCCAAACCTTACTTTCGATGAG 132930
                 *   **** *     ** * **** * ** **  *   **     * *  *       **

Human           GCATG-TTGAATGATAGGTCGGCGAGAG-GTTTGTCCGTAAATGCAGAAAAACGATTAAT 126123
Gallid          CCATGACTTAATATTCTATCATTTGGATTGATAGTACTCAAAACTCCTGACGCGCTCCAT 132990
                 ****  * ***  *   **     **  * * ** *  ***       *  ** *  **

Human           T--TCTTTATCTTG-GTGAAACACGGCGTTTGAGATTTTCCGTAGTTTGGAATGG-GCGT 126179
Gallid          TCATGTTCGTACTGTGTGCAACTGGCTGTGTTGGCACTTCGATCGACAGATATGCCACGT 133050
                *  * **  *  ** *** ***  *  ** *  *   ***  * *   *  ***   ***

Human           AGATTGCGTTATTTCGA-TATTTTTCCTTAT--TAAATTTTATGTCGGGCG--AGGTGTG 126234
Gallid          CGGCGCCCCAAAGATGAATCGTGATACTTATGATAGCGTTTAGGTGGGGAATTAAGTTTA 133110
                 *    *   *    ** *  *  * *****  **   **** ** ***    * ** *

Human           CTGGGGAAAGGCTCTTGCAGTGGCAGAATCGA------CGGGTTTATC---GGTTTGGAT 126285
Gallid          TAACCCCCAAATGTACCCATTGTCACCATTAAATCTCTCTGACTTATCCCGGTTTTACAT 133170
                        *        ** ** **  **  *      * *  *****   * ***  **

Human           G---GTTTGCGAAGATAGATTGTTTTGTAATCGAT-TATTTCGTATAGTCGTTTTAGTGG 126341
Gallid          ATAAATTCGTGAAGATTTTGGCTCATAAAATGTATGCAGCTTGCATTTTCCACTCCATA- 133229
                     ** * ******      *  *  ***  **  *  * * **  **   *   *

Human           TTAGATTTTTTTTCCGTTCTGTGG-----ACGGTTTCGT-TGTTTTTCTAGTTCGGCGAG 126395
Gallid          -TATGTTTCCGTCCTGTCCCTTGAGAATAACAAATTCACGTGTTAGTCCATCCGTGCGTG 133288
                 **  ***   * * ** *  **      **   ***   ****  ** *     *** *

Human           ACTTGGCGGTCGGTCTTGTTT-TAGAGGTGTTTTTATTACCTCTGTCTTTCATCTCTTCT 126454
Gallid          CTCGGCCAACCCTATACATTTGTATGGATGTTCTCGATCCGGCCGTTGTAAATTTCATAG 133348
                    * *   *       *** **  * **** *   * *  * **  *  ** ** *

Human           GAACTTAAGCCATATAAATTTTAACTTTTAAATGCAACGATACACATCGAAATCA-GTAG 126513
Gallid          TCGATGTTCCAGCATTTGCAAAAATCCATAAAT-CGCCTCCTCCTGGTAAATCCATATTG 133407
                    *    *   **       **    ***** *  *    *       **  **  * *

Human           CACCATAAC--TGATATTATCAATATCAGAATTAATGGTACGATCCAGGTAAATACGATT 126571
Gallid          CGTCCGCGCAGTGATTCTATTAATGTTTGTCCGTGCGTTCAATGGGGTTGGAAAAAAATA 133467
                *  *    *  ****  *** *** *           ** * **   * *  * *  **

Human           TCGTCAGAATTTGAGCCTTTTACCGCTTCTTTACTGTATTCGTCGTGATTTTGCACAGTG 126631
Gallid          ATGCCCATTTTTTAAATTAAAAACAACAGGT-ACGATATACCAGGTAATGAAGTAAACTG 133526
                  * *    *** *   *   * *      * **  *** *   ** **   * * * **

Human           TGTTCGCCGGAAGTTACGAGAGCGCTAGCCTTTTGGTTGGTAAACCAGCTCGACGCTAGA 126691
Gallid          TAGACATGTAAAATGGTTGTACTGAAATTCGTGAGTAATATATAAAACCATAATGCAATA 133586
                *   *     ** *      *  *  *  * *  *     ** *  * *   * ** * *

Human           CATGAGATGGTTTTTCCGACA-AATTCCAGCTTGGTTCGTCTTTTGAAAGTAAAGCTCAC 126750
Gallid          GATCATTCAGTAAAACGCCCACGAGTCTGTATAAGTGAGTCAACCTAACATGT-GTCCAT 133645
                 ** *    **    *   **  * **    *  **  ***     **  *   *  **

Human           ACTAACGGTTGACGATCCGTTTTCATTCTGTCTAACCATTGGTATGTCTCTCTTATATAC 126810
Gallid          ATCTACATGTAAAGAGCTTTCTACATATT-TTTGAGCTTCGGT-CAATGCACGTGTTGGT 133703
                *   **   * * ** *  * * ***  * * * * * * * ***      * * * *

Human           TTCTCCAAGAAATTTAATAACCACATTTGGTTTTGGATAAGACG----TCACGGTACACG 126866
Gallid          ATAGCCGCGATCCGGCTCTCCCTCTCTGGGGTCGGAACACAATGCATATAAAATAATATG 133763
                 *  **  **          ** *  * ** *  * * *  * *    * *    * * *

Human           TAACGTCTAAAAAAATTGTTTAGGTAACGAAAATACAAAACGACGATGGGTTTCATAATGA 126926
Gallid          CCATATCCAAAAAAAGTTATTAGTCATGCAAGCATCTGTCAAATAGCAATCACATAATGG 133823
                  *  ** ******   * *  ** * * **  *     * *  *    *  *******

Human           GTTTTAAG----CAAC------TAGTCGCAGATGTTTTCTCGTTATCCA--CTGT----- 126969
Gallid          AATCAAAGGCCTCAAAGCTGCATAATGAGGGGAACCTTCTCCTCATTGAAACCATTTATG 133883
```

```
                  *  ***    ***       ** *    *     ***** * **  *  *  *

Human           GAATGTACAGGTTAAACATGTCGCGTCATATGTTGATCTGGCTTTAATTTTCAACAGAGA 127029
Gallid          GAACCTACGAGTATAGGTTGTAGAAATTACCGTCAAAAAAAGTATTAATAATGTAGCGAT 133943
                ***  ***  **  *   *** *        **  *      * * * *

Human           TGTAGTTCTATCGTGTTTTATGTGGAAATACAATTTACTGTCTACG------TCAAGATT 127083
Gallid          CGCACTTCCATCATGTCGAATGATAAAACGCAAGTCAACTTCTGCGCAACTTTCAGAATT 134003
                 * * *** *** ***   ***   ***  *** * *   *** **      ***  ***

Human           GTTGATCTTATTTAGTTCGTGTATAAAAACGTAGCCATCG--TCAGTGAACGATAA-GTT 127140
Gallid          GTTGACATTATGTATCGGGGATAGGGAATCGTATGAAGGGGGTGGGTCAATTGTATCATC 134063
                *****  **** **    *  **   ** ****   *  *  *  ** **   **   *

Human           GTCCATGTTTTGTACCTC---ATGATTCGATATA--AGTCGGCTTTTATTGTACCACGAG 127195
Gallid          GTATGTGGGCAACAGTGCGGAATTATTGTATCTGGTAACCGATTCTAATTGTAAATGGAT 134123
                **   **      *   *   ** ***  ** *   *  **  * * ******    **

Human           ACGCTTTGGATGTTTGTCGTTGTGTTGAGGCGACAATGGAAAGTAACAGTTGACCCTATT 127255
Gallid          ATATTCTGTGTGTCTATGTCTACATGTAAAGAGCTTTCTGCA--TATTTTGAGCTTCGG 134181
                *   * **  *** * *   *   *  *     *  *    *   *   **** * *

Human           TCGTATACTAAAGAAGGACTCGTCTCCGGGCGGGGTACAAAATGTGCGCTTATGATAATA 127315
Gallid          TCA-ATGCACGTGTTAGTATAGCCACGATCCGGCTCTCCCTCTCTGGGGTCGGAACACAA 134240
                **  ** *    *   *  * * * *    ***    *    * ** * *    * *  *

Human           CATAGAAGCGGCAATA-ATCGAAGCATCAAGGG--ACTCATAGTGTACACCTGTGCCAAA 127372
Gallid          TGCATATGAAATAATATGCCACAGCCAAAAAAGTTATTAGTCATGCAAGCATCTGTCAAA 134300
                   * * *    ****   *  ***   **  *  * *  *  ** *  * * ** ****

Human           TAGCAGTTTCTGCGCCAAAT-AGCGGTTTCGGATATATATAA-------GTCACACCCAA 127424
Gallid          TAGCAATCACATAATGGAATCAAATGCCTCAAAGCTGCATAATGAGGGGAACCTTCTCCC 134360
                ***** *  *       *** *   *  **  *  *  ****         *   * *

Human           AATAAAAAAAAGTCAAGGA--TTTCCGTTATACTGTTTTATTTTTTTATTTAAAAAAAAA 127482
Gallid          CATTGAAACCATTTATGGAACCTACGAGTATATTCTGCCAACCATCGTTTGGTTTCTGCA 134420
                 **  ***  * * * ***   * *   **** * *   *    *   **          *

Human           GTTGGGAGTTAACATTTTGAAAGTGTAAGATAT---TTCGATTTAAAATCTCTGCTGACA 127539
Gallid          GTTATCACCCGAAATCCTTTAAATGGAAAGAATAAGCCCAAGTACCAGTCATTATTTTCA 134480
                ***   *    * **  *  ** ** **   **     * * *   * **  *  *  **

Human           GAATTATAG----ATAGAG--ATAGAATTGTCACCATCAATGAT-TGAGCAGACCCGAAG 127592
Gallid          GGCTTTAGGGGGCATCGTGTCGTTAAATGGGTAACAAATTCGATCTGAGAAGGTATGTAT 134540
                *  **   *    ** * *   *  *** *  * **     *** **** **    * *

Human           GACAATTTCTTTAGGTTTATCTTTG-GCATACTTATTGCATATTTTAGAAGCCATTACAA 127651
Gallid          GTCAAATATTCAGAGCTTCTAACAATACACTCTTCCCAGTGAGATTGCAATACATTATAA 134600
                * *** *  *    * ** *       **  ***       *  **  **  ***** **

Human           AGTCGATGGGCG-TAGCGGCGTAAACCATAAGAGTTTTTACGTCCGAATGTC-TCGTA-A 127708
Gallid          TTCTCACATCTAACTACCTTGGGTGTCATACAAGCCGTAATATGTGTTGTTCGTTGTAGA 134660
                     *          *   *     ****  **   * *  *  *    ** * *** *

Human           ATAGATC-ATGTGTATTGTTTTCAAGTTTATCCCACAATTCTTGAGGTCCTTGTTTTTGT 127767
Gallid          ATACACCTATACGGACTTGTTCCAATATCAGGAATCGATTCGATGGTTGAGAGCCTACGT 134720
                *** * * **  * * *  ** ***  * *      * ****    * *    *  *  **

Human           AGTTCTATAAATTGATTAATTG-CATCTTTGTCATAATAGTTAGGCGGTGCATGATTCAT 127826
Gallid          ATTCCTG-ATACTGCTAAGAGATCACACAAAACAAGTAACTTATTAATTCCATCAATCAA 134779
                * * **  * * ** * *      **       **    * ***     * *** * ***

Human           CTTAAATGGCTCTGAAACAGTAATGTTGGGTCGAATCATTAATTCTCTTTGCATCGTCTT 127886
Gallid          AGTTGTCATATCTATTGCAGCACCCCCAAAAGAAAATAATGTTTCTGTACATGTTCTAGC 134839
                  *       ***    *** *            **  * *  **** *     *  *

Human           AATGATTTCTGC--TACTTGTTTTGATCTTGTAAAGTACATAATGATGGC----TCCGGT 127940
Gallid          ACCGTTTTCAACATTGCTCGTTACGACC--GAAAGACTAAAAATAAAAGCCAATTCCGGT 134897
```

```
                *  * ****  *  * ** ***  ** *  * **     * *** *  **    ******

Human           GTTAGAGCATCCATTAGAACAG--ATGTTGTATACAGCTTCTTTTAGCTTGTTTTCAGGG 127998
Gallid          TTAGACTTCTCAAGTGGGACGCTTATGCGGCGCACTGTACTTTCCAGCTCTTTCCTGAAG 134957
                 *       ** * * * **    ***  *   ** *    **  ****  **      *

Human           AAAGGTTT-------TTTGGGGATCTG--------------------TTTGTATTCAAAT 128031
Gallid          TTAGACCTCCCACATTTTCCAGCTCTGCTCCAATCTGCTGAAAAAAATTTACGTACAATT 135017
                  **   *       ***   * ****                    ***   * *** *

Human           GCTCGTATGGTATTTCTAAAGAGTTTTCTAAAA------TCTTTTGTAG---ACACCCTG 128082
Gallid          GGGGTTATAGACGTACCAATGAACACCTTAAAACATAAATCCTGTGGAGGGTATACTTCA 135077
                *    *** *   * * ** **      *****      ** * ** **   * **

Human           CCGCGATGAATTTTAAATTTTGCCGATTCTTTTTTTTCTTTTCGTCTAACATATTC---- 128138
Gallid          GTATTATCGATATCTAGATCTTCTAACTTTTGTTTCATCGATAGTAGAACACGTTCACAA 135137
                     **  ** *  *  * * *  * * ** ***      * **  ****  ***

Human           --TCTATAAGACAAC-----------TCTTCTTCAAT----TTCACTTATTTCA---GAA 128178
Gallid          TCTCTATTCGCGGGCGGGTTGCACAATCTTCTCAAAAAAGGTTTGATCAATGCAACTGCA 135197
                  *****  *    *           ******  **     **   * * * **   * *

Human           TCTGTTTTCTGC----GTTCCAG----ATATTCTTTTCTTGAAGAAATTTTGC-ATACTG 128229
Gallid          CCGCATCTCGACACATGTAACGGGGGCATAGTTGAACGTGCAACAGGTCTTTCTGTAAAC 135257
                 *   * **  *    **  * *    *** *      *  ** *  * ** *  **

Human           GAATCTGAATCATCATCCCGGGTGC-GTTTTTTTTTGCATTTTGTTGTGTTG----CGTG 128284
Gallid          AAATCTAGACGTATACGCCGGGAGCTGCGATACTGTATATTATGTTCGGCTGTATACGTA 135317
                 *****  *     *  ***** ** *   *  * *  *** ****  * **    ***

Human           ATATG-TTACTTTGG------TTATCCCGTGGGCGA-TAAATTGTGTTAGTGGTGGTGTT 128336
Gallid          GAATGACTACGATGGAGCCAATCATCCCATGTGCCAGTGAAGTACATTATAGGTGGAACT 135377
                  ***  ***  ***      * ***** ** ** * * ** *   ***  *****   *

Human           CGTGGTCTTAGGTGTCTTTTTTTGTTCTTGATCAATGGCGTT-TTCTAAATGAT---TGG 128392
Gallid          TTTTTTCTT---TGCCTCACCTTAGAAGTGAT-GGTGGTACTATCCCCACTGATGTGTAA 135433
                  *  ****   ** **    **     ****   ***   * * *  * ****   *

Human           ATTGTTCATGTGTGTTAGATGGACTAGAATTGTCTAGGGGAAATTTTTCACTTGTTTTCT 128452
Gallid          ATTGAGGGTTTCT-TTCCAGGGTTTTAGTTTTTCGGATATTAACATATCGTTTGCCCGTC 135492
                ****    * * * **  * **  *    ** **       **  * **  ***

Human           GGGAG---TTTCTAA--ACATATCTGCA--TGTCTATGTTTTGATTCG-TGATTTGGAGA 128504
Gallid          GACAACACTCTTCAACTACCCATTTTAAATCGTCTAAGTACACTTCAGATAATTCCTTGA 135552
                *  *    * *  **  **  ** *  *   ***** **     *  * * ***    **

Human           TTTGATTTTGGTTT-TGTCTCT-TGAAGCTGATTTAG---------GTCTTGTTTGTTTT 128553
Gallid          CATAGCTTTCATCTGCATCGCAATCACAACCATTCGGAAAATAATTGCCCTGATTATTAT 135612
                  *   ***  * *   ** *  * *     ***  *         * * ** ** ** *

Human           -TCTAGTTGTTGTA--GTCTAGATCTAGGTGAATATGTG-GCAGGAT-CTTCACTGTTTT 128608
Gallid          GTGTAGCTGCGGAACCATCAAGCTCCATTTTATGATATAAGCGAGATACAGTATCCCTGG 135672
                 * *** **  * *   ** ** ** *  * *  ** *  **  *** *   *    *

Human           TATATCGACTTTTGCGC-CAGGATTTTGATCTGGAACTGGAA--CATGATGAAGAGACTG 128665
Gallid          TATAACGATAACAGGGGGCAATATCCTTACTAGGGTATAACATGCATTCTGGAGAAACGG 135732
                **** ***     * *  **  **  * *   **   *   *  ***  ** *** ** *

Human           ATGATGATGATGATGATGATGCCGATGATGATCTAGTTCTGGAGTGCTGATAGTTAAATG 128725
Gallid          ACGTTATATATG-TGTTTGGATCTCTGGGTTCTAAATTTTCACTCGCTCGTATCCAGCCA 135791
                * * *    *** ** *     *  **       * ** *     ***  **   *

Human           TTATAAGCTTGTCTGTTCCGGTTTCTGTACGTGAATTA-GATCTGGAATTTTCTTCAATA 128784
Gallid          TATTTATCCTCTACGGGTTGGAGTATATCGGAATGTTTCGGTGGGGGAGGATCCTCTATC 135851
                *  * * * * * *  *    **  * * *  *    **  * *  ** *   ** ** **

Human           CTGGAAGAGATGCTTGATGATCCAGAT-TTGTGTTTTTTT--CTTTTTGGAGTATTGAGT 128841
Gallid          AGAAAATCAATTTTCGAGATTTCTGATGTTCTAGTATTTGAGCTATGTGGTTTATCCACC 135911
```

```
                    **   **  * **   * * *** ** *  * ***   ** * ***  ***  *

Human           GAATCTGGTGAATAACGAGAGTGAGATCTGGATCTA---GATGTAGAACTTTCTGAATCG 128898
Gallid          GTAGCTGCCGAATCAT-ACATTAACATCTCAGCTAGCATGACGTCAATATTTTCAAATGC 135970
                * * ***  **** *  * * * * ****          ** **  *  ***   ***

Human           GTGTAATTGGTGGATTCTGATTTTTTTCTGTTGGTTCTTATATGAGGTCTGAATCGGGGG 128958
Gallid          CCCTGCATACACCGCACCCATTCCCATTTGTGTACAAGTATTTGTG-TCGACATCGAGCT 136029
                   *   *        *  ***    * ***       *** ** * **   **** *

Human           TTGTAAA----ATTGATCATTGTGGTTAAAGGACTGGAGTCG-AGCTGCAGTATTTTTGT 129013
Gallid          CCCAAAACGTTGTTAGGTAATCTCCATAGAGAGCAAAATCTGCATCTGTGGTATTTCGTA 136089
                    ***     **    * * *   ** **  *   *   * * ***  ******

Human           AGAAATTGAGTTCAGGCCCGCACCGAAATTTGATTTCTGATACTTCAGCTTCTGTACTAA 129073
Gallid          CAAAGTTGACCACATGACTCCACCGGG--TAGATCCCAGGCGTATATTGAGCCATGCTAT 136147
                  ** ****   ** * *  *****    * ***  * *     *      *  * ***

Human           TTGAGGTGTCATGTGGATATATTTGTCTTTTATTAGAATCGCAATAAATCTGCCTCGGTT 129133
Gallid          TGAGTTTCTCAATTCATCATAATCAT-TTCCGTGAAATGTTTTATGATCCCATTCCAGTA 136206
                *     * ***  *    *** *  * **   * * *      ** *  *     * **

Human           TACGT-TTGTTTGAAGTTTTTTCACAAGTTTGAGGGGAAGGTATGTTGGACATTAATTTA 129192
Gallid          AAATTATTGTATACAATTTAGACTTGTATAAAGAGCGAAGATATGCCAAATCTGTGGAAG 136266
                 *  * **** *  * ***   *     *     * **** ****    *  *

Human           TTGTTAGGGGTTTTACCGGAGGCTCTGCTGGAGGCCATGCTGGAGGCTCTGCTGGAG--- 129249
Gallid          GGTTTGTAGACTCAGGCAGGGGTGTCACTGTAA-CAGCCTCGAGATTTCTACCAAAGTGA 136325
                   **   *  *    * * **     *** *  *      *     *** *   **

Human           GCTCTGCTGGAGGCCCTGCTGGAAGCCCTGCTGGAGGCTCTGCTGGAAGCCCTGCCGGAG 129309
Gallid          GTCCTACGTTCGAATCGTATCAAACCTGGCATACCCGCAGCATCGGTAACCTGAACACAT 136385
                *  ** *    *   *   *  ** *     *    **      ** * **    *  *

Human           -GCCCTGCTGGAGGCCCTGCTGGAGGCTCTGCTGGAGGCTCTGCTGGAGGCTCTGCTGGA 129368
Gallid          CGTAGGACTGTGACTTCTGTTCTTATCCTTCCCAATAATGACCATGGAGGC-CCACAATT 136444
                 *     ***      *** *     *  * *            ******* *  *

Human           GGCCTTGCTGGAGGCTCTGCT---GGAAACTCTGCTGGAGGCTC-------TGCTGGAGG 129418
Gallid          TATGTTATATAAAGCTTGGATTATGGTAGATCTGGTTGACGTACATTGAAATTCTGGAAA 136504
                    **     * ***  * *   ** *  **** * ** *  *       * *****

Human           CCCTGCTGGAGGCTCTTCTGGAGGCCCTG----CTGGA--AGCTTTGCTAGAGACTCCGC 129472
Gallid          CCATTCTGCAGACGGTTTAGGTGGGTTTAAATTCTGAATTGGCCCGCTTACTTGCATCCA 136564
                ** * *** ** *  **  ** **   *     *** *   **     **    *  *

Human           TGGAAGCTTTGCTGGAGGCCCTGCTGGAGGCTCTGCTGGAGGCTCTGCTGGAGGCTCTGC 129532
Gallid          TTCATACATTGAGCAAATTAGAGTATGAGGATC---GGGAACATCGTCAAAATGGGCAAA 136621
                *  *  * ***    *      *   **** **    ***   **  *   * *  *

Human           TGGAGGCCCTGCTGGAGGCCCTGCTGG---AGGCCCTGCTGGAGGCCCTGCTGGAGGCTC 129589
Gallid          TAGAGCTATTGTACTACGTTCCGCCGGTACGGAGCCTATAGACATAACATATCTCGGATT 136681
                * ***    **    * *  * ** **    *  ***   *      *   *   ** *

Human           TGCTGGAGGCTCTGCTGGAGGCTCTGCTGGAGGCTCTGCTGGAGACTCTGCTGGAGGCTC 129649
Gallid          TGATGGCAATCTTTTGAGTTGGTCCAGGAACAACACTGCACCCGATGAGAATACAGTCCT 136741
                ** ***      *    *  * **         * ****    **      *  ** *

Human           TGCTGGA-----GGCCTTGCTGAAGGCTCTGCTGGAGGCCCTGCTGGAGGTCTTGCTGGA 129704
Gallid          TTCAGGATCGTTGTCTTGGTTGGGGTGCCTATTACAATCCACCTTTTTTCTCCTTATAGA 136801
                * * ***     * * * * **  *   **  *  *  **    *     ** *  * **

Human           GG---CTCTGCTGGAGGCTCTGCTGGAGGCTCTGCTGGAGGCTCTGTCAGAGACCTCGGT 129761
Gallid          AGAAGCGAAGCAAGACATCCTGGCGCAGCTCTCGCCAACAGTTGTCTGAAACAAG-CAAT 136860
                 *   *   **  **    *** * **     **     * * * * * * *   *  *

Human           GAAAGTTTTACTCAGAGGTTTATCAGA------------GTTTTCGCCATTAGTTTGGTT 129809
Gallid          ATATTCTTAAGTTATAGGTACGTCAAACCACATCAAGGTATATCCCCTCTCTTTATAAAC 136920
```

```
                  *   ** * * * ****   *** *             * * * *  *   * *

Human           AGAAGTTTCAGATTTATTTTCGGTGGA-ACTGCAGTTAGGTTTCTTGTCAGTACATTCAT 129868
Gallid          TGTACAATCATACTCATATATTGTAGGTATCACAAGCGAGAAAGGAGCAAATGACAACAT 136980
                 * *   *** * * ** *   ** *  *   **     *      *  * *     ***

Human           CACCGTTAGAAGTGC-TATTCATGGTGCTGT-TGCCACTGTTGGATTTGT-TAAAAGCAG 129925
Gallid          CCGAGTGGTACGTGCATATTTATTGTATCACATACGTCAATTATGTTTCTGTGATAATAG 137040
                *   **   * **** **** ** **      * *  *  **   *** * * * *  **

Human           TAAATGAGCTAGGATTGGAATGACTCCGAATAGGTGAATTGTCTGTTAAATTTTTGTTGG 129985
Gallid          TTA-----CAAGGGTTGCAGAAACTACCGTCAAAAAAAGTAT-TAATAATGTAGCGATCG 137094
                * *     * *** *** *   *** *    *    ** * * *  ***  *   * * *

Human           CGCCTGCAGAGTGT----TTTTTAGACGCA---CAGTTTCTGTTCATAGTTGAAGG---- 130034
Gallid          CACATCCATCATGTCGAATGATAAAACGCAAGTCAACTTCTGCGCAACTTTCAGAATTGT 137154
                * * * **   ***    *  * * *****   **  *****  **   ** *

Human           -AGTTTCTTGCGTGGGGCATGCAGAAGTTGATGGAATAA-TAGTTTCATTACAAAATAGT 130092
Gallid          TAACATTATGTATCGGGGATAGGGAATCGTATGAAGGGGGTGGGTCAACTGCATCATCGT 137214
                 *   *  **  * *** **   ***    *** *     * * *  * * **  ** **

Human           -TGTG---AAAATGGCTGAATAATATTAG--AGTCCTCTATTTCTGTTTTATTTGTATTA 130146
Gallid          ATGTGGGCAACAGTGCGGAATTATTGTATCTGGTAACCGATTCTAATTGTAAATG-AATA 137273
                 ****   ** *  ** **** **  **    **   * ***    ** **  ** * **

Human           TCATTGGAAAAACTGTTTAAGTCCGTATTTTCGGTACTTTCTTC---TGTTTTATGGTTT 130203
Gallid          TATTCTGATTGTTCATCAATATCTGAAAGCACAGGTTCTTCCTCGATTGGTGGGGAGATA 137333
                *  *  **       *  *  ** * *    * *    *** **   ** *     * *

Human           GTTTTGCTGTTGGGGGTTGTTTTAGTGAAATTATCATAGAATTTATCTGATAAACAATTT 130263
Gallid          GTCTCGGGTATAGAAACGGCATC--TGCATCCAAATCGAGACTTTCGTGCTGCA-GCTGT 137390
                ** * *    * *     *  *   ** *   *       * **   ** *  *   * *

Human           CCATAATTGCTTTCACTGTTGTTGGGGG-ATTTGTATTGGCCTTCTTCAGAGCT-ACTGG 130321
Gallid          CCATTTTCCATTTTTTGGTCTTTGTCTGCACCAATATTCGTAAAGGTGAGAATTCGCTTA 137450
                ****  *   ***    **  ***   * *    **** *      * ***   *  **

Human           AATCATGTATACAATGGTGCGTTTTTGATTTTGGTGGTGTAGATATCTTTTCATTGTCGG 130381
Gallid          ATCTCCGCCTCCAACGTCGGGTA--CGGCTACACTGTATTAAATTTAATTTGATT-CAGA 137507
                *     *  * *** *  * **    *  *    **   ** ** *  ***  ***   *

Human           GTGAGGTTTTATATTGTTCATC------AGATGTTTTGCCGTGTAGGTTATGTGAGGGAT 130435
Gallid          TTTTGTTTCTCCAGATTCCACCTCCCCAGAATCCACTCCCCCAACGACAATGCCTGC-AC 137566
                 *  * ** *  *   * ** *        **    * **     *   ***    *  *

Human           CAGACTTTTTTGATGTATTTTTGGCGGCTTTCCCTGAAACATTTTTAACGACTACTTCCC 130495
Gallid          AGAAAGCCATTAACAAACAT---ACAGTATCCCATAATGGTGTTTTTACGTCTGCCGTTC 137623
                   *     ** *   *  *    * *  * ** * *     **** *** ** *    *

Human           TAACGTCTTG-GTGTCCGTTGGAGTTTGCAGTATTTTTTTTAGATGTCGTACGTCCCCGA 130554
Gallid          TACCGACTAACATACCAGCGAAAAATGACATGCACGATCCTAGTAATAATGATTTTGCAG 137683
                ** ** **    *  * *    *  *  **       *  ***   *  *   *   *

Human           GAATGTTTCATTTTAGGATCATT----AGTGTCTT-TTGGGTCATGATGTTTTTTGTA-A 130608
Gallid          AACAGATCAATGTAACCAGCATATAAGAACGCATTGTTGCATTCTCTGACTCCACGGATA 137743
                 *  * *  ** * *  * ***     *  *  ** ***  *  *     *    * * *

Human           GAGTTATCTTTGGTCGGGGCTCTTGGAGCATGTTTTGATATGAGAACTCTGTCTTGTAGA 130668
Gallid          GAATAGTACGGGGTCGTTGTACAAGAACCCGTTTTTATTCAAATGCTCC-CCCAATATA 137802
                ** *  *    *****  *  *  * * *   **** **   *   ***   *   ** *

Human           GAAGTTTTTGATCGGTGTATATTTTTTTTTGTGTGGTGGAGTTCCGAGTTAGAATGAATT 130728
Gallid          TTTGGGTTTTGTCTTTCGGCCAGCATTAATGCGCGAACGGAATGTACAACAGCTTG--CT 137860
                   *  ***  **  *         **  ** * *   *   *       **  **   *

Human           TTAGTTTTATGAACCGCAAAGTTTAAGGTGGGCTCAGGTGTCATCGGAAGAGGAG----- 130783
Gallid          CTGCTTCGACAAGCAAGTCCGCATATGTTCCTCCTTCGCACTGCTGGGCAAGCTCCCCCA 137920
```

```
                 *  **  *  * *      *  ** * *   *    *       **   **

Human           ---ATAGCAGGGGATCTAATTTTGAAAAATCCCTAGCTTCAG---TAACGGCTTCATCTT 130837
Gallid          TCTGCTTCATACCATCTCCCTCTTCTTTCCCCGTAGAGCTACCCCTTTCGGTTTGTTTCG 137980
                       **    ****   * *       ** ***    *    *  *** **  *

Human           CATGCTGATTAGATTTTCTCTGTTTAAGATCAGAACGCGATGGAACA--TGGTT-TTCAT 130894
Gallid          CATACCGACTTTCGTCAAGATGTTCATTCCCTGACCTCCGGGGCTCAGGCGGTAGCTCCT 138040
                *** * ** *    *     **** *    * ** * *   **  **   ***   ** *

Human           TATGTTTTTGTACTTTATGTTTGTTTATTGTTTGAGAACTGTGTCCTTTTGTCTTTTGGA 130954
Gallid          CCCACTGTGACAGCCACTCTCCCTCTCCGGTTAGCTCCTGGCATTCTATCGCCTTCCC-A 138099
                     * *   *     * *   * *   *** *      *  * ** * * ***    *

Human           TTGATCTACAACGGATATGTGTTTTTTTGT---ACTTCTGAGTATCCACGCCACAATTTG 131011
Gallid          TCACCCTTTATTGGAATAGCCCCCTTCCCCGTGACTCCAGAGTGAGGGGGCGGAGAACTG 138159
                *    **  *  ***   *     **       *** * ****      **    *  **

Human           AGCCATTGTCTATTAGTGCTTCCTTCGGT--AACGGGATAACTTCAGTGTCATATATTTT 131069
Gallid          AATCTCCACCGGTCCCT-CTGGACCCGATCCAAAGCGCTCATCTCGGCGTCCCCGCATTT 138218
                *  *     *  *   * **     ** *  ** * * * *  ** * ***      ***

Human           TTTAATGAGATCCATTTTTTGGGAACTAGGTCTGTATGATAAACTGATTGCGTCAGGAGA 131129
Gallid          CTCGCCGTCCTCGGCGCATTCCG--CTTCTGTTTTCCCATGCCCTGCCT-CGTCGGCGAC 138275
                 *    *   **      **  *  **     * *   **   ***  * **** *

Human           CGTTGAAACTTGCTGTTTTTTGTGTTCTTTGTTATCCTTAGCATTCTCATGTTCTTTAGT 131189
Gallid          CCCTG-----CGCGGCCCTCCGC-CCCTTTTCCACCCTGGGGAGCGGGGGCGACCCAAGA 138329
                *  **      ** *   *  *    ****   * ***  * *          *   **

Human           TTTCGT-AGTTTCTGGTGTT--GTCTGTGATTTTGTTGGATAATTGTGAT--TGTCAGAT 131244
Gallid          CGCCGTCAGCCCTTCGTGTTCTGCTTCGAATTCCATCACCCCCTGCCGATCTTGCACGCG 138389
                   *** **    * *****  *  *   ***   *       *   ***  **   *

Human           TGTACGTATGTGGGGTCTGTTGTTATTGAAGTCTGTGAAAATGCCATGCTATAGTCTTGG 131304
Gallid          GGGACGAGCAAAGCGTGCGGTGCGGGCAGAAAGACAAGGATGGCTGTGCGTTGAAGATGA 138449
                 * ***      * **  * **       *         * **   ***  *     **

Human           GCTTGAAAATTGAAGTTGGTTGTTAGTTGTGTAACATCTTTATAGTTATTCATACGGAGA 131364
Gallid          AAA-ACAAATCGCGGTTGTGGGTCATGAGTGGAGGGAGGGTGCCATCTGTGATGCCGAGA 138508
                      **** *  ****   ** *   *** *       *    *   * ** * ****

Human           TCTGGATTATATTGT-TCATAGTTTGGATGCATGTATTGTTGATATGATAAATCTGCGGA 131423
Gallid          ---GGGTCAAACTATGTTATAAAGAAAAACGATGGGTGGGAAATATAATAAAGCAACCGA 138565
                   ** * * * * * * ***      *   ***  * *   **** ***** *  * **

Human           TCTGTTTGAGAAATCGTGTAGTCTGTGGCCTCTTCCGCGTCTGTTATAACTTCTGGGAAA 131483
Gallid          AATGGTACATAAA-------AACTAAAAATACCTACACGGTTACACCACCGATCAGGCGA 138618
                  ** *  * ***         **       * * * **  *     * *     **  *

Human           AAATCTTCCATATGGCTGTTGAT-ACTTTGAGTTTGGATTGAAGTAATTACGACTATCGT 131542
Gallid          AGAAGTTCCAAACGATTAACAACCGGGACGAGACGTTGCCGTTCGATCCAGGTCTCGCTT 138678
                * *  ***** * *  *    *       ***        *    *   * * **  * *

Human           ATTTTCCCTTTCCTCCTCTGGGCCTATGGGAATGATGTCTAAAGTTAGAGTCATATCCTC 131602
Gallid          TTTTGTGATCTCTTATCCTATACCGCCGCCTCCCGTCCGACGAGAGCAAGTCGCACCGCC 138738
                 ***    * ** *   **   **   *       *      **    ****  * *  *

Human           TATCGGTATTTCTAGATTCTCTGTAGGAACTCTCTATACTCCTGTATGCATCATAATTAG 131662
Gallid          ACTCGAGGCCACAAGAAATT----ACGATTCTTATACGGGTGGGCGTACCGCCTACTCGA 138794
                  ***      * ***   *    * **    * **       *  * *  * ** *

Human           GTTGATACGGCAACGAGGTGAAATTGCACTGTGAATTTGTAGTTTGGTTCTGTGATGCGT 131722
Gallid          ACTATCACGTGATGTGTATGCAAATGAGCAGTGCGAACGC-GTCAGCGTTCGCACTGCGA 138853
                  *   ***  *      ** ** **  * ***     *  **  *  *  *   ****

Human           ACATGGGCTGTAGGACTTGATCTGGCAGTTCGGAAGCAAAACGCATATGGATCGAATTGT 131782
Gallid          ACCAATAATATATTATATTATATTATATTATTGGACTCTGGTGCGAACG--CCGAGGTGA 138911
```

```
                **      * **  *  * ** *   * *   * *       **  * *   ***  **

Human           TTCCGTTTGAT--GGTAGATTGGAAGCAGAGGATGTGCATTGTCTGTGGATCTGAGCTGC 131840
Gallid          GCCAATCGGATATGGCGATATGTTATCACGTGACATGTACCGCCCCAAATTCGCACTTGA 138971
                  *  *  ***  **     **  * **   **  ** *  * *      **  *  **

Human           TCTCCGTGGATCCTGGGGAGTTCTTGAATTTTTTCTGGAATCTTGAGAAGATATGGGATC 131900
Gallid          GTGTTGGGGGTACATGTGGGGGC--GGCTCGGCTCTTGTGTAT--AAAAGAGCGGCGGTT 139027
                     * ** * *  * * *  *  *  *    *** *  * *  * ****   * * *

Human           TTGATGCGTATTCTCACTCAATGGTTGTCTAT----GTGTGACGTCCGCATCATGGTATA 131956
Gallid          GCGAGGTTCCTTCTCTCTTCGCGATGCTCTCTCAGAATGGCACGGCCGATCCCCGATATA 139087
                  ** *    ***** **    * *  *** *     **  *** ***    *  * ****

Human           GTCCGGATAATTTCATGGCTTGGTGGACAACGT-CGTTATGAGCATTTGCTTGTTTGGAT 132015
Gallid          TTTCCTGTAAGGA-ACGCTACGCTAGGCGACGAACGAGCTGAATTTCTCCCTTCATCAAA 139146
                 * *   ***    * *    * * * * ***  **   ***   * * * *    *  *

Human           TTTTGTTCTGGAAAACATGTTTATAAAATTCAAGCTTAATAGATAGTATTTTTCCGTCGA 132075
Gallid          TAAGTAATAAAATTACTAGCATTCGATAAGCAAATAAAAAAAAGGATCTCAATTAATAGA 139206
                *          *  **  *  *   * *  ***    ** * *   * *    *   * **

Human           TTAGTGATACGCTTAATGTATTTAATAAATTATGGATAATCATTTTGATTATGATACATC 132135
Gallid          ACGGCGAT-TTTTTATTTACGGCGATATTTGATGATGAAGCGGTTCGTCGGTCAGTCCCG 139265
                   * ***    *** *       ***  * ***   ** *  ** *     * *  *

Human           CCGTGCAGAGAATGATGTTTATA--CAGATTGTGCGTAGTATGCAAATGAAAGTTAT-TC 132192
Gallid          ACGATCCGGGACCGCAGACCACCGTCAACATCGACCCGGGATGAGCCTGCTGGGCAGACC 139325
                 **  * * **  *  *   *     **   *   *   * ***    **   *  *   *

Human           CATCGTTTTGTAATATGAAAAGTTTCAAATATAGTAAAGAAAAGTACATATACGTTTTCA 132252
Gallid          CATGGGTGTCGTTTCTGTATGGTCTCA--TTTGCTCATGTTATGGTCTTTTATCGGAGGA 139383
                *** * * *    * ** *  ** ***  * *  * * *  * *  * * **        *

Human           AGGTGTGTTCTAATAAAATGATG-AATAAAATAGTAGTTTTCT-ATAGAACGTAGTATAG 132310
Gallid          ATGAGCGACGCGGCTGGGTCGTGCAATGTAGAAAGGATGGGCTGATATAATCCGATATTT 139443
                * * * *            *  ** ***  *  *    *   ** *** **     ***

Human           ACTTTTATTGCATAATAATCATTGAGTAAATACCTGGAGACATTAAATTTGTATTTTGTT 132370
Gallid          TTTTTGTTGGGGGGACAGTTCCACAATATAGGACCGCGATCATTAAAATGGGAAGGTTTA 139503
                  ***  * *    * * *     * ** *   * *    ******* * * *   * *

Human           TTTTTAGATTGTTGAGCTATATAGATAAATTTGGATGATAAAGACGTTATAGTTTGTAGT 132430
Gallid          TTCTGCGAATGTTGA-TTACATGGAAAAATATGTATGTGTGGGAGAAAGTATGTCGATTT 139562
                ** *  ** ******  ** ** ** **** ** ***      **      **  * *   *

Human           GAAATTCACTAAATCGTTTTAAACTTTTATATTTATCAATTAAGTAGTAACTTTCCGTGG 132490
Gallid          TAAATGTAGTTGGA-GTTCGGTATTACTTCCTATATAGATTGAGACGTTAATATTT-TAA 139620
                 ****  * *     ***    * *  *   * ***  *** **  ** * * *   *

Human           AAAGGTCGCAGAAACGGAATAATGCATTTACTAATTGGAAAAGATGTAGGAGAGTGGTAA 132550
Gallid          AAAAAATGCGATGAAAGTGCTATGGAGGAATAAGGGGGTGGTGCTCGGCGAGCATGTTCT 139680
                ***    **     *  *    *** *   *  *   **     * *    ***  ** *

Human           AAGCCTATTGGTTTCTTAAGTATGTATATCGTGCGATGTGTTATTAACTAGTTAGATGTG 132610
Gallid          GTAACTGCCC-TTGCTAGGGTTCTTCACACGAGCCTCGCCTTATTAAAT-GTGAGTTCGG 139738
                    **     ** **   **    *    ** **   *  ******* * ** ** *  *

Human           GATTACTAACAATGGGGGGGGGGAATTATTCGCATA-------ACATGCAATTAAAATTC 132663
Gallid          CAATGCTTACGATCTGCCGAAACAAGTTTTTATGTCTACTTCCACAAGGGGGTGGTGCTC 139798
                 * * ** ** **  *  *    ** * **     *        *** *    *     **

Human           AATTCAAAATTTATGAAATTGACATTATTTGGAGAAATTTTATGGGACGCATGATTTATA 132723
Gallid          GGCGAGCATGTTCTGTAACTGCCCTTGCTAGGGTT--CTTCACACGAGCCTCGCCTTAT- 139855
                       *  ** ** ** ** * **  * **      ** *   **  *  *  ****

Human           TAATTGATCTATTAACAGGCCAAGTGACGGGATTTAGTGAGCATGATATTAAATCTTTTC 132783
Gallid          TAAATGTGAGTTCGGCAATGCTTACGATCTGCCGAAACAAGTTTTTATGTCTACTTCCAC 139915
```

```
                *** **     *   **   *    **   *    *   **  *     *  *  *   *

Human           ATTCAGTAG--TTGAAGTTATTTTCGTGGTTGATTACATTTTTGAATAATTAATGGTATA 132841
Gallid          ATTCAAGCGCTTTCTCATAGGGATTCTCATTAAAGACAAGTTCGGTAACGCTTTCGATTA 139975
                *****   *  **    *     *  *  ** *  ***  ** *   *     * *  **

Human           TAATTAATAATAAGTAGGGCTAATAGTACAATTTATTATCACTATTCTTATGCGATGAGT 132901
Gallid          GAAACTGTTTTAGGT-GCCCTTATATTACACCTTACGAGGATC-TCCTTAAATT-CGAGT 140032
                 **    *  ** ** *  ** *** ****  ***  *  *   * ****      ****

Human           TCAAG------TTCGGGCGCTGAAAAATTACGTGTAATGAACACTTTTTTTAGAGAACTT 132955
Gallid          CCAGTAATTCCTTTGAGTGACGGGAAGCCTTACGAGTTGCCCCCCCCCCCCAAAAAAAAA 140092
                 **        ** * * *  *  **       *   **  * *       * * **

Human           -------ATGTTATTAGATAATTTATATTAATTAGTAGTTCAATAATAGAATTTATGATT 133008
Gallid          CAAAAAAAAACTGTCTTATAAGGAGCATGGCTAAATGCGACTTCGGTAGTGCTTTCGACC 140152
                       *   * *   ****     **   * * *    *     ***   **  **

Human           TATCAATCACGTGATATGCCATATATTTGGAAATCGCGTGTAATAGTATATTAGGTGTCA 133068
Gallid          TAGCAATCTCTTTTTTTTTGATTTAATCTCCCTTGTTAAGATATGATGCAATAAATACAC 140212
                ** ***** * *  * *   ** ** *      *     *  **  *  * **  *

Human           TATATTAA--TTTCATTTAAAACTAGGATGAACATTAACATAAATTATATATAATACCTG 133126
Gallid          GACATGAAACTTTCATTTAT-----TGACGAATTGCACCATTTAAACTATTTGGGCGTTG 140267
                 * ** **  *********       ** ***    * ***  *   *** *      **

Human           ATGAAGTTAATGGCAATTGAATTGGATGGTATATTAATTTATATTTACTTATTTGCATTA 133186
Gallid          TTGCA--CGATTATGGTTATACTTTACCTGCTTTTGCTGCGTAGCTGTTTTGTGACGTCA 140325
                 ** *    **     **   * *  *     * **   *   **  *  **  *  * *

Human           GATTATATTATAATAAAATTGTTTTAATGTTAAAAATTTAAATTATTGATGCATTCATTA 133246
Gallid          GGTT----TGCAACTCCTCCATCAACGTCCCCGCGCTTCCGTCTTTGTACATGATAAATA 140381
                * **    *  **        *     *        **     * *  *     * * **

Human           TTGACGT-GCAGTAAATCCAGTATAATGGGTAAAAGTGAGATTTATTGGAGGGGGGGGGG 133305
Gallid          CTACGGCAGAAAGAAACCCTTCTCAGTTGTCGATTGACACGGCTCTGGGTGGGAACGGTA 140441
                 *   *  * *  *** **     * * *   *  *  *    * * ** ***   **

Human           AAATGTATAATATCCATGTTTTTAAGATATGGT--AATATTTATCAATTATCTAATTGTC 133363
Gallid          TGCCATGACAGATTTGATTTAGTATGGAATGACCGAGTGTGTATTGCTGATATAATAGTT 140501
                     *   * **     **   ** *  ***    * * * ***   * ** ***  **

Human           T-AATTCTGTTGGTTGTCTACAGGGAACCTATAG-ATATATTCAGGAATAATTGGAGTTG 133421
Gallid          TTGAGACTGCAGGT-----GTAGGAGGCCTGTATTGGATATGTCTGTACAAGAGCTGTTG 140556
                *  *  ***  ***       ***   *** **    ****    * * **  *  ****

Human           AAATTGCTGAAGTTTGTGACTACCAAAGGGTTAATATGCATAATCAAATAGATACTGATG 133481
Gallid          ACACCTCTGATATAATCAAGTTATAGATATGTAACATGT-TGATTGAATGTGTACTAATG 140615
                * *   ****  *     * *   * *    *** ***  * **  ***   **** ***

Human           CTACCGTGGGGATGGATATGATTTG-ATAATTAGAATACAATTATATA------TTATTT 133534
Gallid          A----GGTGGGATTAATTTGAGATGTATCATCAGGCTATAGATATGTAACATGTTGATTG 140671
                     *  *****  ** ***  ** ** ** **  ** *  *** **      * ***

Human           ATTATGTATTATGGAGATGACAT-AATTTA--ATATAATACATTATATGATAATTTAAAG 133591
Gallid          AATGTGTACTAATGAGGTGAGATTAATGTACGACGTAACAACGATAATATTGCTTCAAAG 140731
                * * **** **  *** *** ** *** **  *  *** *      **  *  ** ****

Human           TTTATAGTAGACTAGTTTTATTATGTTTCTAATGTGATAGCGC-CCTCCGCTGTTCAGG- 133649
Gallid          GATTTAGCTGAGGGGGCAAAGTCTGAAAATGTTGGCACAATGAACCTTAACAACTGAGAA 140791
                  * ***  **   *    * * **    *  **  * *  *  ***   *   * **

Human           TAAATTATATGATATTGAATGTTTTGAGTAGACTAGTTTGATTATGTTTATGATGTGATA 133709
Gallid          TAAATCGTATCGT---GATCATACTCAGGTGTATATGTT--TTTTTTTTATGAGGTAATG 140846
                *****  ***  *   **   *  * **  *  **  **  ** * ******* ** **

Human           GCGCCCTCCGCTGTTCAGGTAA--ATTAT--ATGATATTGAAAGTTTTG-AGTAGACTAG 133764
Gallid          TCACTGTTAGACTCAGAAGAGGGGATTATCTATAGTTTTTATACTACGGTAGTATGGAAG 140906
```

```
                * *  *  *      * *     *****  **  * ** * * *   * ****    **

Human          TTCGATTATGTTTCTGTTGTGATAGCGCCCTTCGCTGTTCAAGTAAATTATATGATATTG 133824
Gallid         GCTATATGAGTTTTTCTGCTGAGATAGGAAGCGGGGGAAGGGGTATGTTTCATGAGACAG 140966
                     *  **** * *  *** *  *      *  *      ***  **  **** *  *

Human          AAAGTTTTGAGTTGACTAGTTTGATTATGTTTCTGATGTGATAGCGCCCTCCGCTGTTGA 133884
Gallid         TAGATTT--GTCTCATCA--TCGACAACTCCTCCCTCGTGGCTTCGCCCCATGT--TTGC 141020
                *  ***     * *  *  * **  *    **    ***    *****   *   ***

Human          GGTAAATTATATGATATTGAAAGTTTTGAATAGACT---AGTTTGATTATGTTTCTGATG 133941
Gallid         AGAGACTCCTGCAATCTATATAATCCCAAGTTCATTCTGGGACTTTCCCTACACCCGATT 141080
                *  * *  *   ** *  * * *    * *  * *    *  *     *    * ***

Human          T--GATAGCGCCCTCCGCTGTTCAGGTCAAAATTTAAGGGTGGTAAATAGACATTTTT-- 133997
Gallid         TTGGTTGGGGTCTCTACCTGACTTAGTAAGTGTGTCGGGAAGCTTGAATTTCACTCTCCC 141140
               *  * * * * *     ***     ** *   * *  **  * *  *    ** * *

Human          ---------AAAATAT----TTTAA--ACCTAAATAATTTCATCT-----ATTGTGCTTT 134037
Gallid         CTCAAAAAAAAAAAATGAGGTTTGGTTACCCAGGTAGTGCCCCCCCAGAAAGAATAATCA 141200
                        **** **    ***    *** *  ** *  *  *      *   *  *

Human          TTGTTAAGATGCTGAGTTTGTGTGTTTGTGTGTG-TTGTGTTTGTGTGTGTTGTGTTTGT 134096
Gallid         CTGCTTCGA-ACGGAGCTCGATTCATCATCCGGGATTATGCTTTGGGGGATCCCAATTTT 141259
                ** *  **  * *** * *  *  *  *  * * ** ** **  * *  *     ** *

Human          ----GTGTTGTGTTTGTGTGTTGTGTTTGTGTGTTGTGTTTGTGTGTTGTGTTTGTGTGT 134152
Gallid         CGCAGAGGTAAATATCCATGCGTGATCTATTTGTGGTGGCAATGTGG-ACTTTTCTATGC 141318
                   * * *   * *   **     * * * *** ***    ****      *** * **

Human          GTGAGAGAGAA-AGAGATAGAGAGATAGATAT-----ATGGGGGGGGGGAATATATAAAT 134206
Gallid         CTCAGGAAGAACATATCTACTTTGTTGGATTTTGTGAGTGAGAATTAAGAACGTGTATAT 141378
                * **  ***** * *  **    * * *** *      ** *      ***  * ** **

Human          ATAGTAATTTTGAAT--------CTGTGTTTCCAAAATTGGGAATTATGC-GTAGCATCC 134257
Gallid         GAAGCAGGACTGAAAAAAAAAAAACTATTAAGTTCTACTAACAAAGTGTGCCGGTGTACCT 141438
                 ** *    ****         ** *        * *    ** * *** *  * * *

Human          CACTTTCAAAGTGATTTCAGTTAATAAGATGTTG--GATTAAAGATGGATTTGGTATT-- 134313
Gallid         ACCATCCGTGTTGATTACAGCTGTGATATCGTTGCAGACCCCGGATGCATTGACTGCGGA 141498
                 * * *    ***** *** *   *    ****  **     **** ***   *

Human          TTAGATTTATACTTTCAAGTGTTTGGTGGAAAACAGATAGAACAATGGAACGTTGTCTCT 134373
Gallid         CCCACTTTCCATCTCGAAACGGATACTGCG--ACAACTAGGA--ACGGAACATCAAGTTT 141554
                    ***  *  *  **  *  *  **     ***  *** *  * ***** *    * *

Human          GAAAAAAAAAAACTAGTAGTGTGACATTTAGGAAATGTGGTTTGGATACTTTCATCGTGTA 134433
Gallid         TGGTGATCGCATCAGAAGTTT--TTGTGAGAGGGCTCGCTCCTTAATTTCCAACTTTGTA 141612
                    *    *  ** *** *     * **        * *    *   *   *   ****

Human          AAAAGTCAAAAGGTTGAAAATGTTTTTTTTTTTCATGAATAGGAGCTATTATGTGAATGT 134493
Gallid         ACATGGCGTAGTAGAAACGAGAGCTGTGAGGTTCTGGCAGAGATTCCACAAGAGAAAGAA 141672
               * * * *  *      *  *    * *    ***  * * **   * *  *    **

Human          TGAACAAAAACAGATTAAATAGACCTGAAAGGATAATATTGTG---TAGAGTGCTACGGT 134550
Gallid         GTGGAATCGACCCTCTGAATCCAGTATAAATAGTAGCTAGGCGGGATAATGAGTCGCTGT 141732
                    *   **    * ***  *    ***   **      * *   **  * *   * **

Human          CTAGCTACAACTAATGTAATACATTTTTTAGCAGTACTATGATTTATAAAATTAAGATGT 134610
Gallid         TTGCACATTATCAAAGCTACGCATTAGATAACTGCAGAAAGACGCTGCGTATAGTTATGT 141792
                *    *  *  ** *  *  ****   ** * * *  * **        **    ****

Human          CCCCGGGGCATTAGTCTTAATAAACTGGTTTA-GTTTGGAAAAGAGAAAGTGAAAATCTG 134669
Gallid         ATTCTTAGAATACGTCTGTATATACGCACGAACATATAAGTCTGTAAGAATGTAATGCTT 141852
                  *   * **  ****  *** **      *  * *      *  * * ** **  **

Human          AATACTTGGCATCTGTATAAGGTTTTCCAAGAATACAATTGTTTATAGTATC--ACATCT 134727
Gallid         CGTACAGATCA-CTGTTTATTGAAGTTCAACGGTATGAAATTTGAGTATACCTAGAATTA 141911
```

```
                  ***    ** **** **   *   * ***   **  *   ** *   ** *    **

Human           TCAAGAA-AGCTGGAATT--AACTTATGGAAA--------TATTGTATGATGACTTATAA 134776
Gallid          GCAGGAATAATTGGCATCTCAATTTCTCGAGGCTTTTTTTTTTTGCACATTGACATCTAC 141971
                 ** *** *  *** **   ** ** * **          * *** *   **** * **

Human           -GTCAGTTTATGTACATGACTTGGAATTAAAAAGAGAAAGCAATGTCTGTATGACTTTTT 134835
Gallid          CGGAAAAGTGTGTATGCGATTCGC--TTACCCTTCCCCAACTTTCTCTGTCGGTCGTGGT 142029
                 *  *   * ****   ** * *   ***         * *  * *****  * * *  *

Human           ATTATTTGTAAGGTG-ATATTGAAAATAAATT-CCTTTGAG--------AAATCACATGT 134885
Gallid          ATATTGAGGCCGGTGTATGTAGAGAGTCTACGATCTTCGATCTCCCTTCGGATCACATGG 142089
                **  *  *   **** ** * ** * *  *    *** **           ********

Human           TTGCTGAGGTTTTACATAAT--AGAAAAAGTTACATTTGCTTATGTAATTTCTAATCTAT 134943
Gallid          A-GCGGAGATGTTGTAGGGTTCGAGAGGGGTGAGACCTAAACATGCAGTCGCATGCATGT 142148
                  ** *** * **  *   *     *   ** * *  *    *** * *  *     * *

Human           AGCAACATAAAGAGTTAC-ACAGTATGGGAAAAGTATGTATTTTACAA---CAATATAAG 134999
Gallid          GG-AACACGATTGGCCGTTGTAGCATACAAGCAGTACACATGGCGAAAGTTTGCCGTCCG 142207
                 * ****  *   *       ** **   *  ****   **     **        *  *

Human           CATATTTA-CATAATACACATCTGTATGCTAATGATTGCTAATCAATTTACATCTCGGTT 135058
Gallid          CCTGTTCGGTGTGACGT-CATGTTTAGGTTTGAGCATGTAAGAAAAATGGAACTGTTAAC 142266
                * * **     * *    *** * ** * *   *  **  *   ** *   *

Human           TCATCTAGCTAATAAATTTGAGCATTTTCTTCG------AATGGATCATA---ATCAGAG 135109
Gallid          TCTAAAAAGAAGTATCTCGCCCCATTTGTATCATTCGGCGGTGGGAAATATAGGTAATAG 142326
                **    *   * **  *     *****   **         ***   ***    * * **

Human           GGATAGCCAT-CTAATTTAAAGACTTCCATTTTATCACTGTTGCAATCACTTCTAATG-- 135166
Gallid          GAAAAATCATACCTACGTCAGGCTTCTTCGTCTGCGATCGCAGAAGTGTCTGGAGACGCG 142386
                * * *  *** *  *  * * *  *     * *   *  *  * * *  **    * *

Human           ----GTACATTT--TTCATTATCATTACTTGTACCTTCCATACATCTGTTCTCATCATTT 135220
Gallid          CAAAGAAGGTCTGGCGCATTCCGATATAGTTTGCAGCCAATGC-TTGGTCCGCGGAATCG 142445
                    * *  * *    ****   **    * * *   * ** * *  ** * *   **

Human           TGACATGTTGTG-TCATTGATAATATTTAGTATGGGGGAGTTATTTCCTGTTCCCTGCGG 135279
Gallid          GGATCGGAGCCGATTATCGATAATACGGAAGCAAAGGGGATAACTTCCTTGTTTACATAG 142505
                 **   *    * * ** *******   *      ***  * * *****  *       *

Human           GTCAAATATTTGAGTTTCGGCTGTTACAAGACTTTGGGAATTTGTCACATTTGAATTTTT 135339
Gallid          ----AATGTATGA---CCGGACACGACAACACGGAAATCCTGTCTCAAAGTCTTTGTTTG 142558
                    *** * ***    ***     **** **        * * *** * *     ***

Human           CTCACATGCAGTAGAGGTGATAACAGTATATTTACTGGAAAGATCTTGGCAATGTTTG-- 135397
Gallid          CGGAAATAGAACGGATATGACGCTTCTGTAAACGATTGCGGAAGTACGGCGGTCGCCGAG 142618
                *  * **  *   **  ***      * **     * *    *    ***  *    *

Human           -TCTATGCCG-TGTTTAATGATATCAGTTAGATCCATAAAATTTTCCATGACCATTTCTG 135455
Gallid          ACGAACGCGGACGTGTAGCGGGGACGTTCATTGTCTTTGTTTTTCTAATTATTTTGAATG 142678
                    * ** *  ** **  *    *  * *    * *    ***   ** *   *   **

Human           TG-ATAAGTTTCTTGGT-TGGTGTTATGACTC--CCAAGTCTGTTTTGCTATCCTGTATT 135511
Gallid          TATGTATATTTTTCAGCCTCATCCTGTAAATCGGTCGAGCATTAAAAGGTTACGGATATT 142738
                *   **  *** *  *  *  *  * * * **   * **  *     * *  *   ****

Human           ---TTACTATAAGTATATGAGACATTATCCTTATTATATTCTTCATCATCCATTTTA--A 135566
Gallid          GGTTCACTGTATGCGTGTTC-ATTTTATCTTTCGTATACTCCCCTTCGCGTACGGTACGA 142797
                   * *** ** *  * *   *  ***** **  **** **  * **    *   **  *

Human           TGGGGAC-----AAAGTGATTGATGAAG--CAGTGATTGGCTTCTGTCCCATCAC--TGT 135617
Gallid          TCAAGGTTAAAAAAAGTTACTGCAAATGTACAGTAACCGCCGTAAATTAACTTGCGGTGT 142857
                *   *       ***** * **   * *  **** *  * * *   *    *  *  ***

Human           CATTTTTTACATTCATTTTTGCAGAA---CTGTGTATAACACAATTG----------TCT 135664
Gallid          CGGTTGCTTCATGCGTCTCGATATATGTTCTGTGCGCTTCTCAACTAGCTGGCGGCATCT 142917
```

```
                    *  **  * *** * * *    * *    *****     * *** *           ***

Human               AC----TTTGTGAGTTT-TCCTCAGAAACCACAACCAATTTTTTTTGGGAACAAATTTGT 135719
Gallid              ACAATTTTTGTCTGTTTGTCGTTTGCCACTAAACTCAATGATTTCCGG--ACCAGTAGCG 142975
                    **    *****  **** ** *  *  ** * *  ****  ***  **  ** * *

Human               TTCTGATATCATGGGTACCCGGCATTGACCTTATTGGGATGG--TAAACACTGGGGATAC 135777
Gallid              GCGGGGGAGGGGGTGTGTTATATTTCGAGAGCGTTCGGCCGGGTTTGATCCTGGAATTAT 143035
                        *  *    * **        * **     ** **  **  *  *  ****   **

Human               AAAACCTT--------TACTTTTACAATGACCATTATTGATCACTTTATCATAATCACTC 135829
Gallid              GAAACCGTGCCGACTGCGTTTTTATTTTTGTTGTTGTTATTCTATACGTTCCAATGGCGC 143095
                     ***** *           *****   *     ** **  **  *   *   ***  * *

Human               AAATGTAAGTAATTATCTT-----CAGATGTACAGTCATCTGACTCG-CTGCTCGATTCA 135883
Gallid              GAAATGGGGTAGTTTTTTTTGCGGCAGCTCCTCAGGATTTATACGGAATTGTTTATAACG 143155
                     **     *** ** * **     *** *   ***   *   **     ** *     *

Human               GAATTAGGTTCTGATT-TAATAGTTTTTTTAAGATTGAGCTATCAATATTATTGTTACC 135942
Gallid              TCATTGGGTATTGAAGGTATTTCCTTTTATTATAAGTTTTTTTTAA-GTCGCTGTTCCC 143214
                      *** ***  ***   ** *   **** ***  * *    * * **  *   **** **

Human               ATC-----CTCTTCATC-TTCACTCTCTGAACTTATAACAATTAC---GTTTGTGGTTTC 135993
Gallid              ATGAGAAATTTTGATAACTGATATTGCTTACTCATAGCTGATGCCCGGAATTTTAGCTT 143274
                    **       * ** **   * *   *    *** *** *   * *   *  * *    *

Human               AGGTAACTTATCT---ATCTTAATCTTTTTAGATTTGGATGGTTTAATTGGAACATTTTT 136050
Gallid              AAGTGAATCACCTTATAGTGCAGTGCCTTTGATGTCTGGGGCCCTGATAGACAGATGCCC 143334
                    * ** * * * **   *    * *   ***    *  *  *   * ** *  * **

Human               CCTTTTAGCTG--------TCCTCTTTTTAGATTT-------GCATTTCTGAGTCACAGG 136095
Gallid              TTTCTTAAGTGCTGATTTATCATCTCATTACCCCTCGCCTCAGCATAAATATGCGATCGG 143394
                      * ***  **        ** ***  ***    *       ****   *  *  *  **

Human               CACAAT--TTTCTCAGTTTTG-TCATTTTCCTTAGAAGGAGTGAC--------CTCTGGT 136144
Gallid              TGTGACGATTACTCAGAACCGCTCTTCCTAATTAGCAGAACCAGCGAATTATACTTTAGA 143454
                        *   ** *****    * ** *  *  **** ** *    *        ** * *

Human               GG--TGAATATAGAGACTTGCTAAATGGTGTATT-CTCATTAGTGTTATTCATATGAATG 136201
Gallid              GGCCTTTATGTCGAGAGGCCCTGTAAATTTTACCGCCCCCGAACACTTTCCAACTAACTG 143514
                    **  *  ** * ****    **  *   * **   * *   *    * * **  * * **

Human               TTATT-------------AGTTCTGACAGTTGAAT-CACAGTCTGCAA-TGTAATCA-GT 136245
Gallid              TCGATTTGTCATCTAGAGACTCTTATCGGTTGAAGGTGCGATCTGTGGGCTTGATTACGC 143574
                    *   *             * *  *  * ******    *  ****      * ** * *

Human               TTCATCATT-----GTTATCGCTTTCACTCTCATAAGGTGCTGAGTGATCAGTTTCATAA 136300
Gallid              ATAATCGTGCGCGAATTGCCCTGTTCGGCCAATCGAATTTCTAATACATACCTTTCATAC 143634
                     * *** *       **  *   ***   *     *  * ** *   **   *******

Human               -----CCAAATGT-TATCATTTCTGATATTAAAGGGTTGTCATGGAAACCATCACATAGA 136354
Gallid              AGGATCTGGCTGCGCGCCAGTTATTCTACTTAAGGCTCAGTCCGTCCGCTCCCCGATCTT 143694
                         *    **     ** ** *  ** * **** *      *    *   *  **

Human               TTCACTAGACTTTCTACAGCTTCCAATTCTTTGTCTATTTTACTTT---CTGGATT---- 136407
Gallid              TTCGCCCGA-TCCCTCTACCAGCCGGCTTTTCGATCGTGACATTTAGAACCAAAGTGCGG 143753
                    *** *  ** *  **  * *  **   * ** *    *   * **    *   * *

Human               -TAAAGTA-----TCCTGAATTGCATCATGACTT--TTGACAGGAGT-TGCTGAGTCGG- 136457
Gallid              GTAAGGTAATCATTCCCGGATAACTAAGAGACCTGATCTCCGGGAGTGTAACATATCGGG 143813
                     *** ***     *** * **  *     *** *  *   * ***** *      ****

Human               TAAAGGTCATACAAGG----AAGCGTTTCGGTACACTTGGAGTTAGAACCTTTTTCCTGA 136513
Gallid              TAGATCTAATGAAGTGGGCCAAACAAAATGGGAAAGTAGATTTTAAACTTTTTCTAGTCT 143873
                    ** *  * **  *  *    ** *     ** * * * *   *** *   *** *  *

Human               ATCCTTTC----TTTTGTCTGTGACCTTTGATCGGGGCTGTAAGC--CAAAATGTCTTGT 136567
Gallid              ACTCATATACATTTCCGTACGCGGGCGATGCACGAGATAATTCTCATTCGGATATATAAT 143933
```

```
                *  * *      **  **  * *  *  **  ** *    *   *      ** * *  *

Human           T-GTTCAGACCCGGTCTCTACGACTACTGAGTCGGGTTGAGTTTCAAGTAGTTTTT---C 136623
Gallid          TTGTGCCGGAGCGGTTTTT-CTCCTTCCCCCCGGAGTTCACTGTATCGTACGTTGTAAAC 143992
                * ** * *   **** * * *  ** *      * *** * * *   ***  ** *   *

Human           TATCAAGACGTGTTTACAACTTTCAAGGCCAGTAAT--GGATTTAAAGTTATTAGGGCCG 136681
Gallid          TCCGAGGGCAGGAAAAAGTGCCCCGACTCACATACTTCAAAATATAATTTTTGAAGGCTA 144052
                *   * * *  *   *       * *  *   ** *    * *  ** ** * * ***

Human           CCCAGATCTGTCACTGAGGCTGTACATACACA-GTTAGGGCTTGTTTGTTTTTTCC-CTA 136739
Gallid          ATGAGCATCGTGGGGGAGGGGGGTTGTATGTGTGTGAGCAGTCGGTTGTAACCAATATTA 144112
                   **    **    ****  *    **     ** **   * * ****         **

Human           TCTGTCTGCTATTTTCAATGAATG-----------TTTTTGCCTCTTTGGATTTCC---- 136784
Gallid          GGAGAACGTGAAAAAAAAAAGAGGGGGGAGACGGACTCGAGCCTTTTAAAAGTTTATCAA 144172
                   *   *  *     **   * *            *   **** **   * **

Human           CTGGCACATCTGGAGAAGGAGTATCTGGATACATTTTT-----TCTACATCTTTTTCATG 136839
Gallid          CTGTCATGAACCGTATGCGATCACATTGACACGGTTTAAAATACATACGTGTGTATCGTG 144232
                *** **      *     **  *  * ** **  ***        *** * * * ** **

Human           TATGTTTGTAAGTTTTTGTCTTGTTGGGGATTCATT--GTTCTTTGATGGGGTGGGAGTA 136897
Gallid          GTCGTCTACTGTTTGTGGTGGTATTGAAACATCTTCAGGGTTTCAGACATAGTTGTTGTT 144292
                   ** *     ** * **  * ***     ** *   * * *  **    ** *  **

Human           TTGTTATCAGTAGGAGATTGTACT-TCCATGTATTTTAAATATT-CTACATTGTCATCAT 136955
Gallid          AGGATAAAAG-AATAGTTGGTGCGATAAATACAATCGATACATTATTTCTTCCCCCCCCC 144351
                  * **  ** *  ** * ** *  *  **  * *  * * ***  *   **  *  *

Human           CTTCATGG-ATAGATTCG----ATAGAAGATTCCTCTGAATATTTTCCATGTTCATCAAA 137010
Gallid          CCCCGTGTTACAAATTCGCGTTGTTTGAGATATGGGACGGTGGGTGAGCAGGATGGGAAA 144411
                *  * **  * * *****     *   ****         *   *     *    ***

Human           TTCAGCTAACTCTAATAGTATTGATTCTGAAGGGTCTATGTTTAAATCACAGATGGAAGA 137070
Gallid          ATGATCAAGGCCTGGCGGTATAATTAATGGAGAGGGGATAATGCCCTCGCCGATC-AAGG 144470
                 * * * *   **    ****   *  ** ** *   **  *    ** * ***  ***

Human           CTCTCTATCTTTATTTATAGCAATGTAGCCTTCCATTATATCAGAGGAAGTTAAAGGTTT 137130
Gallid          CCCTCCGTATA-ATGTAAATGTCCAAAGGTTTGCATAATACGGAGGGTTCTGATCCGAGT 144529
                * ***  * *  ** ** *       **  ** *** ***     **   * *   *  *

Human           AATCAAACCAGTATCATCATAAACTGTTTCTAAGCATTCACATGCGTGGGTGATCATATT 137190
Gallid          GGCCGATCGTACGCGAGGCCAAA--AGTCCTTAGCGATGATATGAGCCAG-ACTTACTGT 144586
                   * * *       *    ***    * ** ***  * * *** *   *   * *   *

Human           CATTATGGTAATTTTAAACTGGTGTCTGTGAGGTTTGAGGTATTGCC-ATGAAAATTCAC 137249
Gallid          GGCCACGTACTGACGAATTTAGTACCTGTC--GTCTGCATTGTTCTTGATCAGGATTCAC 144644
                    * *        **  * **  ****   ** **   * **    ** *  ******

Human           TGCTGCATAGAACAATACTTTGGAGAACAGC--AGTGCTAATATTTTTGGCGGCAGTAAT 137307
Gallid          TACAG-GCGGCATGAAAATGTGAAACCTCTCCCGCTAGAAATAGAATCTGCTGGGGGGTT 144703
                * * *    * *  * * * ** *      *    *   ****   * ** *  *   *

Human           TAAATTTTTACAATTCACATGAATTTTAAGGAAT-GTTCTATCACAGTTATCCATAAAGA 137366
Gallid          CACATTTTTAAGTTGTATGTAGTGTATCGGTCTTCGTGACACCGCGTATCGCGAGAGCGA 144763
                 * *******   *  *  *    * *  *   * **   * * *   *  * * *  **

Human           TTTGAT--TCATCTGCTTATTAGCAAATTC--CTGAAACATTTTCATATTTTTAACA-TG 137421
Gallid          TCCGACGGCCGCATTGTACTTTGTATATTCTCCTAAAAGAAAGTTGTATTCTGCCCGGTA 144823
                *  **    *   *  *  ** * * ****  ** *** *   *  **** *   *  *

Human           GT---TTTCTATTATCTCATTTCTACTTTCC--AGTAGTTCCTGTTTAAGTAAATTTACT 137476
Gallid          GTCCGTTTGTAAGGTCTTGGAACGGACTGCCGCAGAATAGCTTTCTTGAGGGGAGCGATC 144883
                **   *** **   ***     *    * **  ** *   * *  ** **   *   *

Human           CGCCCACAAAAATCCTTATTGGAGATGTCAGGATTGGACATCTCTTTGTTGTGTGCCATC 137536
Gallid          GACTCGAGA-----CGTGTTCCACGTGACGGCTCTGGGCTTGTTTTGAATGTCCCCTGTG 144938
```

```
                  * *   *     * * **  *  ** * *   *** * * * **   ***   *  *

Human           AACAACAATGAGGT---GGAGACCGTGTTCAATTTTTCACAA-----TCTAATGCCACAT 137588
Gallid          AAGTTTAATGCTGTATCGGAACCCTTCGTTCGGTGACCACGAATGGTTCTGACAGCATGA 144998
                **    ****  **   ***  ** *  *    *   *** *     *** *   **

Human           AAGCTTTGGAAAAAATGTTT--TTGATGGCTATAACAAATTGATTGTTCAT--------- 137637
Gallid          CCTTTCTGGGGGAAATGATCGCTGGAAAACCATCGTAGAACGTCTATACACCGTTGAAGT 145058
                    * ***   ***** *   * **   * **   * *  *  * * **

Human           -ATTAGCTTTCAAATCC--------ATGAGCAAATCTCTGGATTCTGATGCCATCTTTTT 137688
Gallid          GATGTAATATTGAATCGGATTTGGAATAACCGAATTCGGTGATATAAAGACGATAGTCAT 145118
                 **    * *  ****         ** * * ***     ***    *  * **  *  *

Human           CACTAACCCATCAAATTTATCACATGCAAAAACAATGAATCGCGGTTCGCACCAATTAAA 137748
Gallid          GCATGACGTGGGGGGCTGGATCGACTGATATCTAATGGTTCGGGAGTGATACGGA--GAC 145176
                   * **         *      *   * *   ****  *** *  *   **  *   *

Human           AAAATCCAAGTTTTTTCCAGATAACATTTTTGCATAACATGAAACAAATTTAGATGAAAC 137808
Gallid          GGGGGGGGGGGAATGATCGATTTATACCTACCTCTTAAATAAACTATTGCTCCTTTATAA 145236
                         *   *   *   * * *  *     * * ** **  *    *   * * *

Human           ACTACTTAGTAAATTAATAT--TTATCAACTGTCCTAATTGTTC--TAATACCAGCCCCT 137864
Gallid          AATGACAGGTGAATTGTGACCGTTCGCGAACGTG-TAATTCTTCAATACTTTCGGGTCTG 145295
                * *     ** ****   *   **  * *  **  ***** ***  ** *  * *  *

Human           CCAACATGGATCTGTGCAGCGTTAATAAG--CCAGCGGAGTTAAT--TAAATCGTCTTCC 137920
Gallid          TGGGTGTTGCTTTTTTAATTATTATTTGGTTCGGGGAGGTTGGTGCTGGAATGTTAAGA 145355
                      * * * * *  *   *** *  *   * * *  ***  *  *  *  **

Human           ATGTTAGACAGTTCCTGTTTCAT-GGCAGCCTTCACTGATGCACCAATACTTTGGATGCA 137979
Gallid          ATAAATTCCGCACACTGATTCCTAGGCAGGCGTCTCTTGCAGGTGTATACCAGGGAGAAG 145415
                **      *     *** *** * ***** * ** **         ****   ***

Human           AGTGCCAACGGACTGAGCTAG---GATGTAAAAGAAGATATTCTAAGTATGCTATATGTT 138036
Gallid          GCGGGCACGGTACAGGTGTAAAGAGATGTCTCAGGAGCCAGAGCCGG-GCGCTATGCCCT 145474
                   * **  * ** *   **    *****   ** **  *      *   *****    *

Human           ACATGCTTAA-AACTATGT-TTTTTTCTTGTTTTCTATCTTGGGACA--TATAATATATA 138092
Gallid          ACAGTCCCGCTGACGATCCGTCCCCCCTCGATCTTTCTCTCGGGTCGACTTCGAGACGGA 145534
                ***  *      ** **   *     ** * * * * *** *** *   *   * *   *

Human           ATTCATGTACTCACCAACCAAAGTTTTGAAT----TCTTCAGATGCTCCTTCTTCCACA- 138147
Gallid          AAAAAAGGAAAAGTCA--CGACATCCCCAACAGCCCCTCCAAACACCCCTTCCCTGACGG 145592
                *   * * *     **  * *  *    **      ** ** *  * *****    **

Human           -TTACTGGAATAGGA----CACATTCTTGGAAGC-GATGTCGTTGGAA----GACTCTGG 138197
Gallid          CCTATCTGAGGAGGAGAAACAGAAGCTGGAAAGGAGGAGAAAAAGGAATCGTGACGCCGC 145652
                  **   **  ****    ** *  ** * ***  *  *     ****    *** * *

Human           GATGAAAAGATCAAAGGCTTCCAGTTCTGGAAAAAGCAGGCTTT-CAAAGGACACATCAC 138256
Gallid          TCGGAGAAGACGCAGGAAGCAGACGGACTATGTAGACAAACTCCATGAAGCATGTGAAGA 145712
                   ** ****   * *      *          *  **  **     *** *

Human           ACTTGAGACTCTCTTCCAATATTTCTTTGATGGATTCTTCCACCACTGGATCGGGATGGT 138316
Gallid          GCTGCAGAGGGCCAATGAACACCTACGTAAGGAAATTCGAGATCTAAGGACTGAGTGCAC 145772
                 **  ***    *    ** *  *   * * * * *     * *   ***  * *

Human           AGCTGTAAGTATGGATATTTCTTAGTACTTAGAAAAACTCCCATATAGTTTTAAATAAGA 138376
Gallid          GTCCCTGCGTGTACA-GTTGGCTTGTCATGAGCCAGTTTGCCCTATGGCGGTACCCCTAA 145831
                  *  *  ** *  *  **   * **  * **  *   * ** *** *   **      *

Human           TAGATCTAATAAGGACTAATCATACTTACTTTTGCATACGTTCCTCATTGGATTCCATGT 138436
Gallid          C-GGTGACCCTTGGACTGCTTACCACCCCGCACGATCCCGTTCCTGAAC--CTCCCATTT 145888
                  * *       *****  * *      *    *    ******* *     * **** *

Human           -TGTTTCCAGAGGGGTTTCCGAGAATGAACATAGAAGTAGAGGTTGTATCTTTTGCTGAC 138495
Gallid          GCACTCCTCCACCTCCCTCACCGGATGAACCTAACGCTCCACATTGCTCCGGTTCCCAAC 145948
```

```
                    * *   *      **   * ****** **    *  *  ***   *  ** *  **

Human           TCCATTGTATATTAAGATGCAAATTCAGACACCGCCTACTGTTCTGTTAGCCAATGAAAA 138555
Gallid          CTC-CTATCTGTACCCCCCCTCCTCCCGATACGGAGGAACTTTGCGCCCAGCTCTGCTCG 146007
                  *  * * * *       *   * * ** ** *   *   **  *     *  **

Human           AAT-----ATCTTCTGTCAGCAGATATAATACTATATA------AGGAGATTACCACCAC 138604
Gallid          ACCCCACCACCTCCCATCTCTACTCCCCATATTATCTACGCTCCGGGGCCTTCCCCCCTC 146067
                *       * ** *  **   *      *** *** **       **   ** ** ** *

Human           CTCTTTCTTTGCAGAGATTATTCTCTGCTTGAAAATCTGTAACACTGATCATGATGGGAT 138664
Gallid          CAACCTCCTATCTGTACCCCCGCTCCTCCCGATGCGGAGGAGCTTTGCGCCCAGCTCTGC 146127
                *    ** *  * *        ***  *  **      * * *  **  *

Human           ATGAGGAAA--AAGTGTCAGCTACTGGAAAGACTCGTTTAAAGATACTGGCATGTCTGAT 138722
Gallid          TCGACCCCACCACCTCCCATCTGTACTCCCCATTCCCTCTTCTGCCCTCCCCAGCCTCCA 146187
                  **    *  *  *  ** **         * **  *        **  *  * **

Human           CGTTTTAATACTAGCTGCGGCAATAACTATGTTAACGCTGGAAATTATATCGAACCAAAA 138782
Gallid          TCTCCGGAGGGCATCTTCCCTGCATTGTGTCCTGTTACCGAGCCGTGTACCCCTCCATCG 146247
                  *    *    * ** *         * *  *    * *     * ** *   ***

Human           ACGTACCACTACTGATCTCGAAGCTGTGACTGTGGC--GCTGAAGCATGTAAGCACATCT 138840
Gallid          CCGGGGACGGTTTACGCTCAGCTTTGTCCTGTTGGCCAGGTTCCCCTTTTTACCCCATCT 146307
                 **         *   ***     ***    ****   * *    * * * * * *****

Human           CTTGC-CAGCTG-CACTGAATCCACTACTTCTGTACATACCGGTTAGTGAAAGATATGTC 138898
Gallid          CCCCCACATCCGGCTCCGGAGCCGGAGAGGCTTTATGCTCGTCTTACCGAGG---ATCCC 146364
                *   * ** * * * * * * **       ** **    *   ***  **     *   *

Human           TTTTTTATGTTAATGTATTGATTATATATTTGCATACA-TTCCTTAAGAATGGAAAAATC 138957
Gallid          GAACAGGATTCCTTGTATTCGGGCCAGATTTATACTCAGTTTCCCTCGGATACTCAGTCT 146424
                         *   ******      * ****  *  ** ** *    * **    *

Human           ACGGATGGTTTGTTTTTAGATTCTG--------TGACGAGCCAACCCACGAAAAACAAAG 139009
Gallid          ACGGTCTGGTGGTTTCCAGGTGACGGGAGACCCTGATGATCCGCATTGTGACTCTCAGCA 146484
                ****   * * ****  ** *   *        *** ** **       **    **

Human           AATCGAGGAAAAAAATTGAAGGGAAATCTCCA--AGTTGGGTTCAGGCTTTAACTACAGC 139067
Gallid          GCACATCGTCTATGCCCCATGTTTCTTCTCCCCTAGTTATATATAATAGTTT-TCATAGT 146543
                   *   *   *      * *     *****   ****   *  *    **    * **

Human           ATCTGGAATTATCCTACTGTTTTGTATAATGATGATATTCATTACATGTTCCTGGACCAC 139127
Gallid          TTCGGGAAGATCAACATAAAGGAAAGGGTTAAAGGCATTATTTATCGATTTACTGAC-AT 146602
                 ** ****       *             * * *  ***  ***    **    *** *

Human           AGAAAAAGATACAGAGAAGAGTGAAGTGCAATCTTATGCTTCTT-----CAGTAGAGAC- 139181
Gallid          AAAAAAATCCTCTGGGGTAACAAATTTTCCTTACCGTGTAGCTTAGACTCGGAAGAACTA 146662
                * *****    * * *   *   *  * *  *    **   ***     * * ***

Human           TTTAGACTCTTTAAATGAGGCTATTATACCGAAAACTGAAATGAATGTGTAATGTCTGTA 139241
Gallid          TTTTGAGTTACATGGTCAGGGGATT-TGTTGGCTCCAGGAGTTCCGAAGTATGAGATAAA 146721
                *** ** *       * ***  *** *   *    * * * *      ***     *  *

Human           TTTTTCTTTACAGAGATGTACGGAGAGTTTATATTTGGGG---AAAATACCTGACTGTTC 139298
Gallid          CTTAGCTATGTGGAAAACTTCTGGGGCAACATCTCTCGGCCCCAGACTGCTTAAATGGCA 146781
                 **  ** *   ** *  * * * *     ** * * **    * * * * * * **

Human           TGCCTATATGCGAATGTTAAAGTATGTATAATATAAATTCTTACCTTTTAAGAGTGATTC 139358
Gallid          AATTCTCGTTCTATACAGAACGGTTGGGGAAGGGGAGGGGGGGTATATGGAGTATTATTC 146841
                        * * *     ** *  **  **     *         * *  **  * ****

Human           AAGGTGGAGGTTTCTTTGGAGATTGA--------TTCCAGGTGGTGGTTTCGGGTGCAAT 139410
Gallid          GGGATAT-GGCTTCTATGAAGCCTGCGGTAAGTTTTCCAGGCTCAAAAACTATGCCTGGC 146900
                  * *   ** **** ** **  **         *******            *

Human           CAATCTTTCTTCTGGGCGGGAAGAAAATCCAGCAATCCAATAATTGATGG-GATGTAATC 139469
Gallid          TGTTTTTTTTTTTAGAAGGGATATGGACATCGCACATTAAGGAATATTAAAGATAACAGG 146960
```

```
                   * *** ** * *  ****     *    ***    **  * *  *   ***   *

Human           AATGTCACAAATCTGTAAGATTAAATGTGAACAGTATAAATTCTTTTGTGCTTATCAAAT 139529
Gallid          ATGGACATTCGGATGTAAAAGGAATAAGCGAAACCTTTAGCAGATGTGAGTTAATGCAGT 147020
                *  * **      ***** *  **      * *   * *     * ** * * **  * *

Human           TACAATTATGCGCATGAAAATATCATTAAATTGTTTTAAACATTCTTAAATTCAATTTAA 139589
Gallid          CTCGTATAATTCGGTGGTGCTG--ATTAGGTTATCGTAAGGAACAACACGATTGATCTCT 147078
                  *   **      **    *   ****  ** *  ***  *     *   *  ** *

Human           AATAATTTTTGTAGCAATTTTAAATAT-TAATAGTTAAAAAATTGACTTACCAGAC-TGC 139647
Gallid          CATCCGCGTCCCAGCAATCAGGCCTATGTCCCTCTCCTGTGGCCAGCTCACTGGCTGTGC 147138
                 **     *   ******      *** *     *           ** **  *   ***

Human           A----GCGATATAAGAAAGAATCTCTGTGATGAGAATGTTCTCCGGAGCCACTTCTTGAG 139703
Gallid          ACTGTGCGATTCTAAGTGCTACAGTCGTGAGCAGATCAATGGATCGGGGCTCG-CGCAAC 147197
                *    *****   *      *     **** ***     *     * * * *  *   *

Human           AAAAGTGGCACTGAAC---TGGCTGTAACTTCTGCTTATATACA-ATTCTATAGCGGGTG 139759
Gallid          ACTACTGTAATTAAATATTCGTTTATGAATTATGCAAATATGCACAGATAATATATACAG 147257
                *  * **  * * **     *  * * * ** ***  **** ** *    ***      *

Human           TGATGTACAGGAAAATGACGAGG-----ATGAAGGAGCCAATCGCTGTATAATGTTAAAC 139814
Gallid          GGATGCACAGACATACTCCTATGCACCGATACACAGGCACATAGGCAGATGTCGACAT-T 147316
                 **** ****  * *   * * *     **  *   **  ** *    **   *  *

Human           CGCAAGTGTAGAAATACACCCCTTTTAGACAAATATGGAATGGAAATTAGCT----GATG 139870
Gallid          AACGAATATACAGGCACGGACCTCC-AGGAACATATGGAAAATACCTCATCGCAGAGACG 147375
                  * * * ** *   **   ***   **  * ********   *  * * *     ** *

Human           CTTACAAAAATTTAGGGTTTTGTCATATC-TTACTCAAA---AACAGGAATTTGTGGTCT 139926
Gallid          CTTATGCAGGAGTAAT-CTGCGTTAAGTCGTTACTGGATTGTAACGGCTATCCGGAGACT 147434
                ****   *    **    *  ** *  ** *****  *    *** *  **  *  * **

Human           GGATTTTT-TGAGGTTTTCTTCTTATGGCTGACTTGTATTTTTTTATGAGAAAGACGGAG 139985
Gallid          CTCTTCCCCTTTTGCTTGTTCACTGTGCGGCATTATTACATTTACACCGGTAATGCTGCG 147494
                   **    *   * **  *   * **    * *  **  ***  *   * **  * * *

Human           TTTGCGGTTAAAGGCGGGATAA----TAAAGAACACTTTACTGATAAATTG---TCATGT 140038
Gallid          CATGAAAGAGCGAACGGAACGAGGCTCGTACGACATTACAAGAATAGTTTGAATTCTCGG 147554
                  **          *** *  *       * ***  *  *   ***  ***   **  *

Human           TTTTTTTTTTAAAAAAAACATCTGCTACACATCATGAAACAGG-AATATGGTTTTGGTGT 140097
Gallid          GATAATCTCCCGATGGCCTCCCCCTTATGCTTTCTCTACGATGTGGCATTGTTTACGCGG 147614
                  *  * *    *        *   **  * *  *  *  * *    ** ****  * *

Human           TAGCGCTTCTCACGAAATAAATGACTCAGTCAGGG--------GAAAATGTTGTAACCGC 140149
Gallid          CAGTGCCACGTGTGAGG-GGACGATCGGGACGGGATCCATTACGTAAAATTTAGATCTTT 147673
                 ** **  *    **     * **    * * **         * ***  **  * *

Human           GGTACACACTGA--TCTTTAACTTTATGAGAAAAAACAGGAACTGCGGTTGCTCTGTTAG 140207
Gallid          TGTAGAAATTCAATTCCCTTCCCCTTTTACTTTGTTTGATGTGTGCCACTGTTGTACCGA 147733
                 *** * * * *  **  *  *  * * *              ***   ** * *

Human           CAGATTTTAGGGTTCTCGTTAGCCTGGGTAGGAAAGACCCCAACCACAGTACCCACTAAC 140267
Gallid          TAG----TGCCGCGCGTGAAAGAGTGAACGGGAAGGGCTT-ACGTAAAGAACTGTCGGTG 147788
                 **    *   *  *  *  **  **    **** * *   *   * ** **   *

Human           CTTTAACTTTATGAGAAAAACAGGAACTGCGGTTGCTTCGTTAGCAGTTTTTAGAGTTTT 140327
Gallid          CCGGTACGGGGGGAAGCTGTAACGCATTTCGAGTCTTGATAAAGCAGAGCATGAAAATT- 147847
                *    **     **       * * * * **  *  *     *****    *  *  **

Human           AGTTCGCATGAACAGGAAAGACTTCAACCGCAATATCTGCTAATTTTTAACTTTATAAGA 140387
Gallid          AAATCGTAGCTCCTAAAAACTAACGGTTCGTGGTTTTTTTTTTTACCTTAGTGCGTTTGT 147907
                *  *** *    *   ***         **   * * *  *  *   * * *   *  *

Human           AAAACAGGAACTGCGGTTGCCGCGTTAGCAGCTTTTAGAGTTCTCATT---CGCCTGGGC 140444
Gallid          TGA-CGGAAATGTCCGCCATTATTTGAACGTCTGTGCGCATGCGCGATGAGTGCGCAGGG 147966
```

```
                  * * * **   * *        * * *  ** *  *  * * *  *    **   **

Human           AGGAAATACTTCAACCGCGGTACCCGCTGATCTT--TAAATTTATGAGAAAAACAGGAAC 140502
Gallid          CGTATATAGCGCAAGCGCGCAG--GGCTGGTTCGGGTAAGGCGTTCACGCATGCGCCAGT 148024
                 * * ***   *** ****      **** *     ***     * *   *  *   *

Human           TGCGGTTGCCGCGTTAGCAGCTTTTAGAGTTCTCATTCGCCTGGGCAGGAAATACTTCAA 140562
Gallid          TATGCATGACGCGCATGCGTACGGTGGAGTGTATCTGCGCATGCGCGTG---TGTCGAAA 148081
                *  *  ** ****   **      * ****     * *** ** **  *   *     **

Human           CCGCGGTATCCGCTGATCTTTAAATTTATGAGAAAAACAGGAACTGCG--GTTG--CCGC 140618
Gallid          ACCAGGCATGCGCAGATAACCATACTTACGGCGCATGCTTGTTTTGGGTAATTGGTCTGC 148141
                 *  ** ** *** ***    * * *** *    *  *  *   ** *   ***  * **

Human           GTTAGCAGCTTTT----AGAGTTCTCATTCG------CCTGGGCAGGAAATACTTCAACC 140668
Gallid          GCATGCGTTTTGTGTGTAGAGGGCGCATGCGTACTGCCGTAAGCGGTCTGCGCATGCGTT 148201
                *   **   ** *    ****  * *** **       * *  ** *       * *

Human           GCGGTACCCGC-TGATCTTTAAATTTATGAGAAAAACAGGAACTGCGGTTG-----CCGC 140722
Gallid          TTGGACTGCGCATGCGTTTTGTGTGTAGAGGGCGCATGCGTACTGCAGTAAGTGGTCTGC 148261
                  **    *** **   ***   * **   *    *   * ***** **       * **

Human           GTTAGCAGCT----TTTAGAGTTCTCATTCGCCTGGGCAGGAAATACTTCAATCGCGGTA 140778
Gallid          GCATGCGGGTCGTGTGTAGAGGGCGCATGCGTACTG-CAAAATTTATTGTGGTCCTGGTT 148320
                *   ** * *    * *****  * *** **     * **  *  ** *    **  ***

Human           CCC-GCT-------GATCTTT--AACTTTATGAGAAAAAC---AGGAACTGCG------- 140818
Gallid          TTCTGCTATGCAGGGGTCGTGGGAATTTTCTCATATACATTTAAGGGTCCTCATAGTCTT 148380
                  * ***       * ** *   ** *** * * * * *    ***  *  *

Human           --GTTGCCGCGTTAGCAGATTTTGA----AGTTCTCATCCTTATTAGCAGGAAAGATTTC 140872
Gallid          GTGTTCCAGTATTATCACCTTTTGATGCAAACTCCTGTTCATTTTAGTGTTTCGATGCTA 148440
                  *** * *  *** **  ******    *  **   * * * ****           *

Human           AACCGCGGTACCCACTAACCTTTAATTTT--ATGAGAAAAACTGGAACTGCGGTTGCTCC 140930
Gallid          TAGTTTATGCCCCATCGAATGGAAATTTTCAATAAACAAAATGCATCTCACGGTCGTGGA 148500
                 *        ****   *     ******  ** *  ****         **** *

Human           GTTAGCAGATTTTAGGGTTCTCGTTAGCCTGGGTAGGAAAGACCCCAACCGCAGTA---- 140986
Gallid          ATT--TTAAGTTGGGGGTCTCCAATATCACGTGTTGGTGGAGACCCAATAACAGGGAAAT 148558
                 **     * **  ****   *  ** *  * ** **      *****   ***

Human           CCCACTAACCTTTA--------ACTTTATGAGAAAA----ACAGGAATTGCGGTTTCCCC 141034
Gallid          CGCCCGAGGCATTACGGGTACGGTTCCATGGATATATAATGCAGGGGGTGTGGGTTTGAT 148618
                * * * *  * ***          *  ***   * *     ****   ** ** **

Human           CATTAGC--AGATTTTGAAGTTCTCATCCTTATTAGCAGGAAAGATTTCAACCGCGGTAC 141092
Gallid          GAGCAGTTGGGGCGGCAAAAATGCAGGCGTTGTTGCTAGTATTGGTTCTATTCATAGTAC 148678
                 *  **    *      **  *     * ** **   ** *  * **  *  *   ****

Human           CCACTAACCTTTAATTTTATGAGAAAAACTGGAACTGCGGTTGCTCCGTTAGCAGATTTT 141152
Gallid          AGATCTATTTGTTGCCTGGAAATGGTAAGTAGGGGAAGAGAACTCTAGACTGGAGGTTGT 148738
                  *   *  * *     *    *    ** * *        *       *   * ** ** *

Human           AGGGTTCTCGTTAGCCTGGGTAGGAAAGACCC-CAACCGCAGTACCCACTAACCTTTAAC 141211
Gallid          GGAATACGCTATA-CTGATGTAACCAGTACTCGTAAAAGCTGAGGGGTTTCGTCTCTGGC 148797
                 *  * * *  ** *    ***   *  ** *  **  ** *        *   ** *  *

Human           TTTATGAGAAAAACAGGAATTGCGGTTTCCCCCATTAGCAGATTTTGAAGTTCTCATCCT 141271
Gallid          ATGTGCAGCAATGGTGGTATAAGGAAAGGTAGCACGGG-GGATGGTGTTGTTCTGATAAA 148856
                 *    ** **    ** **   *         **   *  ***  **  ***** **

Human           TATTAACAGGAAAGATTTCAACCGCAGTACCCACTAACCTTTAACTTTATGAGAAAAACA 141331
Gallid          TGCATGCTCTTAATAATGTAGGCATATCACTGGAGAGTCTCGCTGTCGACAAGAGGTGCA 148916
                *     *    ** * *  *  *  *  **      *  **      *  *  ***    **

Human           GGAACT-----GCGGTTGCTCCGTTAGCAGATTTTAGGGTTCTCGTTAGCCTGGGTAGGA 141386
Gallid          AGTGCGTGAAAGTCACTAATCGGCCTACTGGCTTGGGGCCTATTATCGCTGTTGACGTGA 148976
```

```
                 *  *      *    *  ** *    * *  **  **  * *  *     * *    **

Human           AAGACCCCAACCGCAGTACCCACTAACCTTTAACTTTA-TGAGAAAAACAGGAACTGCGG 141445
Gallid          TA-----CCACCG-GGTATACACTGCAGGAGGACTGAAATTATGTAAGTAAGCTCTATTA 149030
                 *     * ****  ***  ****        ***  * * *   **  * *  **

Human           TTGCTCCGTTAGCAGGTTTTAGAGTTTTAATTCGCATAATCAGG----AAAG--ACTTCA 141499
Gallid          CTTGGCCAAAATATGGGGTTGTCATCTTAGGT-GTAGTGTCTGGCTGTAAAGCTAATTTG 149089
                 *   **   *   **  **    * ***  * * *   ** **    ****  * **

Human           ACCGCGGTACCCACTAACCTTTAACTTTAT----GAGAAAAACAGGAAC-TGCGGTTGCT 141554
Gallid          GTTAAGGTTTTCCCT--TTTGTAGCTTTGCTCTCAAGAAGAACAGGAAGGTATGTGTGGA 149147
                     ***   * **    * ** ****       **** ********  *  *  **

Human           CCGTTAGCAGATTTAGGGTTCTCGTTAGCCTGGGTA--GGAAAGACCCCAACCGCAGTA 141612
Gallid          CCCTGAGGCGCCTTGGGTACAGCAGTTTATTAAAAAACTAGAACGACAGCA-TCGCACAA 149206
                ** * **  *  **  *       ***         *   *** ***  **  ****  *

Human           CCCACTAACCTTTAACTTTATGAGAAAA--ACAGAAACTGCGGTTGCTCCGTTAGCAGCT 141670
Gallid          GGAAGGAAAATCTGATGGTTGGAGAAGATGGTGGCAAAT-CGACCGTGGGACCGGTAAAA 149265
                   *  **  * * *   *  ***** *     * ** * **   *        * *

Human           TTTAGAGTTTTAATTCGCATGAAC-AGGAAAGACTTCAACCGCGGTACCCGCTGATCTTT 141729
Gallid          AACACAATTG-AGCCCACACCTCCTACTATTGGTTCCCATATCTGTCTTTGATTAATTGC 149324
                   * * **  *   * **    * *  *  *  * * *   * **    * * *  *

Human           AACTTTATGAGAAAAACAGGAACTGCGGTTGCCGCGTTAGCAGATTTTGAAGT-TCTCAT 141788
Gallid          TACCTCCTGTAATTAAAAGAAGGCACTTTT------TTTTCCGATTTTGACTCCTCGTAC 149378
                 ** *  **  *  ** ** *    *  **      **  * ********    **  *

Human           CCTTATTAGCAGGAAAGATTTCAACCGCAGTACC--CACTAACCTTTAAGTTTATGAGGA 141846
Gallid          ATATATCGATAATGTAGCTATTGGATGGAGCAGGGATACGGTCTACGAACTGGATGTAGT 149438
                   ***    *    ** * *     * ** *     **   *    ** *  ***  *

Human           AAAACAGGAACTGCGGTTGCTTTGTTAGCATCTTTTAGAGTTCTCA----TTCGTATTGG 141902
Gallid          AATGCAGGCAG-GTGTACAACTGTCTGTCGGCCGCGAGAGGTTTTAGGGCCCCGCGGGTT 149497
                **  **** *  * *      *   *  *  *    **** * * *      **

Human           CAGGAAATACTTCAACCGCGGTACCCACTGATCTTTAACTTTATGAAGAAAAACAGGAAC 141962
Gallid          TATTCAGTGCTGCGCCGATTCTACCCGCAGGACTCGCACATTTCTGAGGGTATCGGACTT 149557
                 *   * * ** *  *     ***** * *  **   ** **    **   * * *

Human           TGTTGTTGCTTTGTTAGCAGATTTTAAAGTTCTCATCCTCATTATCAGGAAAGACTTCA- 142021
Gallid          CTCTATCCCCACATATTCTAGCA--ACGGTTCTCCATCGCGCAATTAAAGCACGATTAAG 149615
                   * *  *    *   *       *  ******   * *   ** *    *   ** *

Human           -ACCGCAGTTCCCACTGACCTTTAAGTTTATGAGGAAAACAGGAAATGCGGTTGCTTTGT 142080
Gallid          TACCCCAAGTTC-ACTGACTCTTCGTGCAAAAGGGGGAAAAAAGAAAGCCGAGACCACAA 149674
                 *** **  * * ******  **      *   **  ** *   ** ** *   *

Human           TAACAGCTTTTAGAGTTCTCATCCGTATGGGCAAAAAAAATTTCAACC--GCGGTACC-C 142137
Gallid          -GATGGCACTGACCCCGTGCACGGGGATCTGCGGGACGGGTGATGACCCGGGGGCATCGC 149733
                  *  **  * *       **   * **  **   *    *    ***  * ** * * *

Human           ACTGATCTTTAGCTTTATCAGAAAAAAACAGAAACT-----GCGGTTGCCCCGTTAGCGGA 142192
Gallid          ACTG-CGCTCAGCCCAAGCCCCAGGGCCACGTCTTTGTGCACGCTCATGCCCTGCACGGC 149792
                ****    * ***   * *   *    **     *      ** *    ** *   ***

Human           TTTTAGG---GTCCTCACCCACT-TGAGTAGGAAAGACCCTAACCGCGGCACCCACTGAC 142248
Gallid          CCACACGTTTGCCCACGCTTATTGCGGAGCGTGCCGAGGCAGAGGACGGCTCCTGCCGGG 149852
                    * *   * ** * *  * *  *    *    **  *  *   **** **  * *

Human           CTTTAACTGTATGAAGAGAAACAGAAACTGCGGTTGCCCAGTTAGCAATTTTTAAGGTTC 142308
Gallid          ACAGGGCTCCGCGCTGCGCACCGCCACACGTC-CAGGCCAGGACTCGGCGCCTGGGCGCC 149911
                      **    *  * * * *   *   *     * ****    *      *  *   *

Human           TCACCCGCTTGGGTAGGAAAGACCCTAACC----GCGGCACCCACTGACCTTTAACTTTA 142364
Gallid          CGGCACGGCCGCGGACGGACCCCGGTAGCCCCGAGCGGCGCCCAGCGGGCACCACCTGCG 149971
```

```
                   * **   * * * * *   *  ** **    ***** ****  *  *   * **

Human           TGAGGAG-AAACAGAAACTGCGGTTGCCCCGTTAGCAATTTTTAAGGTTCTCAC------ 142417
Gallid          CAGGGACCGGGCTCGAGCGCCCACCTCCGCGGGGGGCCCGTTCGGTCTACTCACAGAGCC 150031
                   ***     *   * *  *     ** **   *     **     * *****

Human           CCGCTTGGGTAGGAAAGACCCTAACCGCGGCACCCACTGACCTTTAACTTTAGNAGTTGC 142477
Gallid          CCGCGCGCGGCTCAACGGCTCCAACGGTCGTGCGCGCGGGGCCGCGCGTGTGGGAGCGAC 150091
                ****  * *    ** * * * *** *  *  * * * *  *      * * * **   *

Human           CCCGTTAGCAATTTTTAAGGTTCTCACCCGCTTGGGTAGGAAAGACCCTAACCGCGGCAC 142537
Gallid          GCCGTCCGCGCGCCCCACGG----CGCAGGGGGGCGCGGGGGTTGCCCCGGGGGCGCCGC 150147
                 ****  **       * **    * *  *   * *  **     ***     *** * *

Human           CCAC-TGACCTTTAACTTTATGAGGAGAAACAGAAACTGCGGTTGCCC--CGTTAGCAAT 142594
Gallid          TCCCGTGTTGCTCTGCTGGGGGCGCGGGGCCGCGCGCCGCATCTCCCGGGCGCCGCCGAG 150207
                 * * **    *   **    * *  *   *     * **   * **   **    * *

Human           TTTTA-AGGTTCTCACCCGCTTGGGTAGGAAAGACC--------CTAACCGCGGCACCCA 142645
Gallid          GCTGACAGAGGCGAACTCTTCGCGGCGGCAATGGCGGGTGCCGGCCGACCGCGGCCTGCG 150267
                  * * **   *  ** *     **  * ** * *         *  ********   *

Human           CT----GACCTTTAACTTTTTGATGAAAAACAGAAGCTGCGGTTGCCCCGTT-------- 142693
Gallid          GGCGAGGACCCCAGGGCGGATGGGGGCGAGAGGACCCCTCGCTGACGTTTTTTTTGTTT 150327
                      ****          **  *   *   **  * ** *  *    **

Human           -------AGCAATTTTTAAGTTTCTCACCCGCTTGGGTAGGAAAGAC-CCTAACCGCGGC 142745
Gallid          GCCGTGGAGCCCCTTTCGGTCCTTTCTCCCGCGTCCGCCCCACCCCCTCCCCGCCGCGGC 150387
                       ***   ***      * ** ***** *  *    *    * **   *******

Human           ACCCACTGA----------------CATTTAAATTTATGGAAAAACAAACTTTTTTGTTC 142789
Gallid          GCCCCCTGGGGCGGCTCCCCTAGCCCGCTGAAAGTCAGCGAGTAAACAGCGGGCGGAGGG 150447
                 *** ***                 *  * *** * *  **  **  * *

Human           ATCATG----CACTTTTTTATATATC----ATTATATCTCTATCCAATC-AGCACTCTTG 142840
Gallid          AGCGCGGCGACAGTACCATCAATACCTCCGATTAGGGTTAGACACAGCGGAGCCTTCCAC 150507
                * *  *    ** *    *  *** *    ****    *  *  **    ***  **

Human           AGGGTGCATACATTAAGGCA-----GTGTTGATTTTTTTTCATTGTACCCACTTACGAAT 142895
Gallid          CCGCCACGTGTGCCGGGGGAACCCCGCGTGGGGCTCTTGTAGCTTCCTCCGCCTACGCCC 150567
                  *   * *       ** *     * ** *   * ** *   *    ** * ****

Human           AACGAAT---CAAAAGCCGTGAAGTAGAATATTT--TAATGAT--GTATTAATCATCATT 142948
Gallid          ACCGCGCGCCTATTGGCCGGGCTGTACGCTGCCGCGTAGCTATTGGCGGCGGGGAGGAGA 150627
                * **       *   **** *  ***   *      **   **  *        *  *

Human           TCCTACCACGCCTATTAACTTCAGTATTTATAGGATAGGCAATTTGCCGCTATACGCCAT 143008
Gallid          GCGAGCAGAGAGGGGCCGGTGCGGGAGTTTGGAACTCCGCGGTCATTCATCTCCCGCCAC 150687
                 *   *   *         * * * * **      *  **  *    *      *****

Human           TAGCTGTTCTTCTGCTAGCTTGGACACAGCGGTACAGGGT--GAGTTATCAGCGTCTTAT 143066
Gallid          GCCCCATGCACGTGCCCCGCCCCTTCCTGTGGGCCGGGGTCTGCGGGATCGGATCCGGGG 150747
                   *  * *   ***           * * **  * ****  * *  *** *   *

Human           AATGTGATGTCTTTTTTTTTATTTTCTTGTCGGTATTTTAAAAGTAGTCT-GTTAAGATG 143125
Gallid          GCTGCGAGATGCGGGCTGAGCTTTCCCGCCCTGGATTTAGCGCAGAACCTCGTTAAAACA 150807
                  ** **  *      *    *** *    * * ****        *  ** ***** *

Human           TTTTTAAATAGATAGTTATAAAATTTTTGGAACCGTGTAGTCAATAAAACAATTTATAGA 143185
Gallid          TGTCCTG-TATCTACCTATAGGA-----AGAGCGTTCGAGTTATTGGATGGAGCAGGGAT 150861
                * *     **  **  ****  *      ** *  *  *** * *  *   *

Human           ACGGTTTCTGGCCCAGA-ACAGAAATTGATTTATGT---CGTCTTTCTG--AATTACAGG 143239
Gallid          GTGGTCTACGGACCGGATGTAGTAGTGCAGGCAGGTGTACAACTGCCTGTCGGCCGCGAG 150921
                  *** *  ** ** **   ** * *  *   * **   *  **  ***       *  *

Human           AAAATTGCGAGGGATAACTTTA---TCAAATGTAAAATTATA--TACCCTCTGTGATAGA 143294
Gallid          AGGTTTTTGGGGGTTAGGGTTAGGGTTAGGGTTAGGGTTAGGGTTAGGGTGGGGGGTCGA 150981
```

```
                *   **  * *** **   ***   * *    **   ***     **    *  * * * **

Human           TTTATTTTGTGGATATTTGAATGTACCATCGAGCGTATATCAACGCAAGATGTGGCTATG 143354
Gallid          GAAACAGGGGGGGGTCTAATTTCACACCACGGAGTCACCACTTGCATAATCCAAGTTTGCG 151041
                   *    * ** * *        ****  ***      *    *  **      *  *  *

Human           ATGGGACACGGTCCTATCTGTACGTGGTCGTTTTCATTAATTGGGAATATTATTTTTTGA 143414
Gallid          TCACATCACAGGTG-----GTATGTGCCGGTTCC----AGTGTGGATTTCCGTGCTTTGA 151092
                      *** *        *** ***   ***      * *  *** *    *  *****

Human           CCCCCAAGGAGGGATT----TTATAATATTAAAATTTTCAAAGTTGATACTTA--TGTCT 143468
Gallid          GCTCGGGATCGAGACTAGGTTCACGGTTAAGACATCTTCAGTATGAGTGGGAAAGTGTCT 151152
                 * *      * ** *    * *   *    * ** ****    *   *    *  *****

Human           TTCCACCACAAGACTAAAACCTG-TCCGTAAGCTCTTAAATTTTTAGTATTCAAAGATCC 143527
Gallid          TATCGCCCCCGGATTCTCCCATCCTCTACATTATATTTGGCTTATTTTGGATGGGGGTGG 151212
                *  * ** *  ** *    * *  **   *   * **    ** *  *       * *

Human           GTGTAATATTCCATGTAACGGGATATTTTCAAGAAT--GGCTCCCGTTAACATCGATTTT 143585
Gallid          GGGGGGTGAAA-ATTTGGGGGGTTAGGGTTAGGGTTAGGGTTAGGGTTAGGGTTAGGGTT 151271
                * *   *     ** *   *** **    * * *  *  ** *  ****    *     **

Human           CCACATCCGGGTGGACCAATAAAACTAACGGTGTTTTTTTTCCCTCTTCTGTGGCTTAG- 143644
Gallid          AGGGTTAGGGTTAGGGT--TAGGGTTAGGGTTAGGGTTAGGGTTAGGGTTAGGGTTAGGG 151329
                     *  ** * *     **    **  * *    **           *  ** *  *

Human           CCACTGATATAAT-ATGGTTCCTGCTAATAT-AGGGTCAA----AAGACTGCAGACAGAA 143698
Gallid          TTAGGGTTAGGGTTAGGGTTAGGGTTAGGGTTAGGGTTAGGGTTAGGGTTAGGGTTAGGG 151389
                  *  * **   * * ****   * **   * ***** *     * *  *   *  **

Human           CAACTGGTAAACC--GGGTTTTCATAGATTTCAAAGTCAGTGTCTATGTGCGCTATAAAA 143756
Gallid          TTAGGGTTAGGGTTAGGGTTAGGGTTAGGGTTAGGGTTAGGGTTAGGGTTAGGGTTAGGG 151449
                  *  * **      *****    *     * *  ** ** **    **  *   **

Human           TC--GATAGCTTTTCTAGTTGCTCTTATTCTTTT--TTTAGATATAGATAGAGCCCTATG 143812
Gallid          TTAGGGTTAGGGTTAGGGTTAGGGTTAGGGTTCAGGCCTAGGGTTAGGGTTA-G 151508
                *   * *     **   ***    ***   **    ** **   ***    **   ** *

Human           GATTAGCACGTCCCCGTGTGTTAGTGTCAATAATTGCATGTATGATCTAACATCTC--TT 143870
Gallid          GGTTAGGGTTAGGGTTAGGGTTAGGGTTAGGGTTCA-GGCCTAGGGTTAGGGTT 151567
                * ****           * ***** ** *    * *  *  * *  ***   *     **

Human           CGTATCCACGC--GTCTTCCGTGACTATTCCAAGATCAGACAGCCATTCGATGGTTCCCA 143928
Gallid          AGGGTTAGGGTTAGGGTTAGGGTTCAGGCCTAGGGTTAGGGTTCAGGCCTAGGGTTAGGG 151627
                 *  *    *   *  **  *   *    * * * * **    *    * * ****

Human           G-AAATTTAGATTTTAAAGATAT---TCTGTTGGTC-CCATCCTTTGGAAACAGCATAAG 143983
Gallid          TTAGGGTTCAGGCCTAGGGTTAGGGATCGGTTGGCCGCTAGGGGTTCGACGAAATTTTTT 151687
                  *   **      ** * **    ** ***** * * *    ** **   *   *

Human           TTTAACATTAGGTTTTTTATTGAAATGG--GATATGATGTGTTTTCGATGTATTTCATCG 144041
Gallid          TTTTATACAGTGTGTGGCCGCGAGAGGGTTAGAGGGCCGCGTGCGCAGTCGGAGTTTTCC 151747
                *** * *    ** *      ** * **       *  * **   *  *     *  **

Human           CATACGTCTCCCAGTTCGTTAATATTTGTCCAT---GCCCAT-TGGAACTGTGGTCTAAT 144097
Gallid          TATTTTCGGCCCCGCGCATGCGCGGTCATGTAGAGGGCGCGTGCGCAGTCGGAGTTTTCC 151807
                 **      *** *  * *      *  *   *    ** * *  * *   *  ** *

Human           AATTTGAAGAAGGGCAAAGGGGATATAACTTAGACTGAAGACCTGTGTTCCGCCACCATT 144157
Gallid          TATTTTCGGCCCCGCGCATGCGCGGTCATGTAGAGGGCGCGTTCCTGATTTCCTTCCGCT 151867
                 ****   *    **  * * *   * * ****   *        ** *   *  **  *

Human           TTTCTTTGTTTTGGCATACGT---GCACCAAT-----CATCTATTTTAGGGGC--GTTCC 144207
Gallid          ACCCGTGCGTTTGGCACAAGTTTGGCGGCAGCTCCAGCATCCGTTTTTGGAACTCGATTC 151927
                   * *   ******* * **   **   **       ****  **** **  *  * * *

Human           CG----TCGAAGAAATCTTTAATTAATACACCCCTGATTTCCGTTGTGTGTTT-TCCTAT 144262
Gallid          CGATTACCGCAGGTCTCTCGGACTAGTTGAGA--GGGTAAGTGTTTTGTTTTTGTTTTAT 151985
```

```
                **     ** **   ***   * ** *  *     * *    *** *** *** *  ***

Human           CGTTGCGGTGGGAATTCCATCTAAAGCTATCAATAATGTTAAGGAGGGTCTGCCAGCTT- 144321
Gallid          TAATTTGTTCTGTGTTTCCTTCTCCCTATACAAACGTGTT--TGTTAATTTATAGGCTTG 152043
                   *  * *  *  ** * *          ***   ****   *    * *    ****

Human           TATTCTCCGCTTGAGCGTAC-CACTTTGCAGGGGTGGGGACGGCAGTTTTATTATTCCAG 144380
Gallid          TATGCCCCGCTTCATTTCACTCATTATGTAGGCGTTACACTGACCTTTGCACTGCGTCGG 152103
                *** * ****** *    ** ** * ** *** **      * *  **  * *    * *

Human           TAATTGA-GTATGACATCGATCATTTTGGTACATGCAGTTATCCATTTAATATCGGGCGC 144439
Gallid          GGATCTATGGGTTTCTGTGTGCTTACTACGATTTTAAACTTGTTATTTTACGGTTTGAAT 152163
                  **  * *  *  *   *  * *  *   *  *  *  *    **** *      *

Human           TCCCGGTGAGTGCATATCTCTAAAATGTGGCACGGGCACGTTAGAGAATTTACAAAAAAA 144499
Gallid          TTTTAATACTTGTCCTCGTTGAATAGATGATA---GCCTGTTAATAGATTTGAGTCAGGC 152220
                *     *   **      *  ** *  **  *   **  ****    ****     *

Human           ATC-CGTAGATATTCCAGGGATTTCTGCCTCCCACTCCACGGGGCCTTTGATAGTCAACA 144558
Gallid          ATTGCGGAGTTAT----ATGTTACGCGGTTCCCAGCCTATAAGAATCGTGGTGTTGGCGC 152276
                **  ** ** ***      * *    *  *****  * *   *     ** *  *

Human           TGATATATTTGTCCTTAGTCCAAAAATCATCACTTGGATTTATTATGGAAAACATACCGT 144618
Gallid          CAAAAAATGCGCGCCAA--CAAAAAGGCGCCA--AAAAATTGCGCGCCATTGTTTGGCGC 152332
                  * * **  *  *  *  * ****  *  **     * **       *     *  **

Human           TTGTGCCTTCCAAGATTTTTATAGTTGTATACGGAAAATAAGGTAATTCCCCTGGCTTGT 144678
Gallid          CTTTTTCTGC-GCCGTTTTCAAAATCGCGCCATACCAATTTCAAAGTTCCCGCCATTTGG 152391
                 * *  ** *     **** * * * *         ***     * *****     ***

Human           CACCTCTAGATGTCTTCAAATCTGGAAGATTTGTTAT-----GGCAACAAATTCTCAGTA 144733
Gallid          C----CAACACGCTATTATCCCTGCATGATCTTCTTTAATTGGACGACATTCCTCGATTC 152447
                *    * * * *   * *   *** * *** *  * *     * * ***       * *

Human           TGGATCCGGCGGAACTTCTGTCGGACAATGCAATCTCGTCCACGTGTGACGAAAACATCG 144793
Gallid          CCGATCCA-CATATCCAGTGACAGG-AGTTCGGAATAAACGTTGTGATACGCGATCGAGT 152505
                  *****  *  * *   ** * *  * * *    *   *   ***  ***  * *

Human           CAGCTGGACATCATTTTACGCAACCTCCGCACGTTGAAATGTCAGTCCAATCAACGACAT 144853
Gallid          TTTCGTGGCATATTCCTACGGAAACCTATTGTTCTGTGGT-TGGTTTCGATC-----TAT 152559
                   *  * ***  *  **** ** *         **   * *   * * ***      **

Human           CGGCGGGTCATACTGGAGTGATGACGACACAGTCGCAGTTTTCCAATGGCGTACGGGATC 144913
Gallid          CGTT--CTCGTACTGC-GTGACCTCTACGGAACAATAGTTTTCCAGGAGATTTCCCGGTT 152616
                **     ** *****  ****   * **  *     *********   *  * *  * *

Human           AAAACCGC-GAATCACTGT--CGACTCTGACGGGCCTCTCGCTGGAAAGCATCAACA--- 144967
Gallid          TCGACTGCCGAAGCATGGAAACGTCCTGGGAAAATCTGTTGTTCCGTAGTGTTCTCGTGA 152676
                   ** ** *** **  *   ** *   *      ** * * *    **  *   *

Human           -ATCAAATCAATGTTCAACCCACTCAGATGACCTTTCAACCCATTTCCCCGCCGATGCAG 145026
Gallid          CACTAACTCGAGATCCCTGCGAAATGACAGTTTTCTCTGGGAATTACATCGTC--CTGAT 152734
                 *  ** ** *  * *   * *       *   * **     *** *  ** *     *

Human           GGTCAGAATTATGTCTATAGCAATAATATGATCAATCCCATCAAACCTAGATCAATTATC 145086
Gallid          TGTCGCGACATGGAATGGAAGCCTCATAGGAAGAACTCGATGTGATGATGCTCTCTAGCC 152794
                 ***   *    *  *  *    * *** **  **  * **   *    * **  *   *

Human           AAATCA--CATGGTCATTCGATGGGGGAGATGTCATTTGCAGACCATTCCT-TGTATGTT 145143
Gallid          AAGAGAGCCGCGAACGCTCCA-AGGAGAACTGTTATCTCGGGGAGATCCCGATCTCTCCT 152853
                **   *  *  *  *  ** *  ** **  *** ** *   *   ** **  * * *  *

Human           AATGCTCAACCGCCTGTCCAACAGCCTCAACTTAAATCCCTTGTAGGTATGCATCCATGC 145203
Gallid          ACCAGCAACTCGAGATCTCTACGA----GATTACAGTTTTTGGGGGAAATGTGTCCT--C 152907
                *      *  **      * **       * *  * *   * *  *  ***  ***   *

Human           ATGACCGCTACCTCACAGGGTAAATATCAGACAAATAAAACTGT------GGGGCC---A 145254
Gallid          AGAACTGCTTAATCGTAGA--AGCTTCCTAGTGGATGGCGTTGTCTCGTAGAGGTCCAGA 152965
```

```
                *  ** ***   **  **   *  *  *      **     ***      * ** *   *

Human           CCCTCTATTTCAGCTTCCC-AAATTTCTACGGGAAATGCCGGTATAAGACCCGGGGAATA 145313
Gallid          TCTCTCCTGTTGGCAACTCGAAATCTCTACGAGA--TAACAGTTT--GTCTAGGAAACTT 153021
                 *     * *  **  * * **** ****** **  *  * ** *  * *  **  * *

Human           TCAGTCTGTCCATA-ATCAATCTTCTGGAAACGGGTCTAAGTCTTATGAGATCACCACGG 145372
Gallid          TCCTCCCAACTAAAGAGCGATGACTTAGGAAGTAAACGTGCCCTCATCA--CCGCCCTTA 153079
                **   *   * * * * * **    * * **     *     ** ** *   * **

Human           CATCCGGCGATGAATGGATACTGACTACACCCGGCGGGCAATCCTGGAC---TCTGAAAC 145429
Gallid          CACACTGCTAGTCATTCATGTACATTGCGATTGTGCCTTGGTGCGGGGCGGTTCCTAGGC 153139
                **  * ** *   **  **    * * *    *        * * ** *   **  *  *

Human           GGAACCCCCCTAATCCTCCGAATAACAGAACTAATTCAGTTGTCAACAAGGCACAACAG- 145488
Gallid          ACCATTTATCTTGTATTCCTG--TACATCCCCTCCTTAATACTTTAATTGGAGCCACATC 153197
                   *     **  *  ***     ***   *    * * *  *  *   **  * ***

Human           GTCTCGCATGCCCAACCTTATGTCTCTGGGTCTAGCGACGGTTTCTATCAGGGGG----- 145543
Gallid          GTTTTGCCATGTTGATCTGTTTCCTTTCCCTGTAACAATGGTTACATTCTAAACGAACTA 153257
                ** * **       * **  *  ** *   * ** * * **** *  **     *

Human           --CTGCTCTCCAATCATGTGCGTATGTAAATACGCCAGGT----TTCACTCCTGTGTGCG 145597
Gallid          ACTTGCTTTGAAGAAATCTACGAATTGATAGACTGTATATGCGCCCCTTTTCTTTATTAA 153317
                   **** *  *   ** * ** **  * * **   *  *      *  * ** * *

Human           AAACACAAAATATG--AACAACTCACAGGCGACACAACTAAGCGCATCCATGAACTGCAT 145655
Gallid          AAACGCTTAACAAGGTAACAATATACGTAACATTCGAACAAGTACCAAGAT-AACTATAT 153376
                **** *  ** * *  *****   **     *  * *  ***  *    ** ****  **

Human           AAATGCGTTATCAACTAC----TATGGATGCGATTGTCACTTCTACTTCCAAGCCGGTGG 145711
Gallid          AACGGCTTATATAACTATCTTTTTATTTAGACAAGTCACAACTATGTTCTTTTTTTTTTA 153436
                **  ** *    *****     * *   *      *****  ***  * *       *

Human           GGGTGGTATCAAATAATCGGGGTGCTAATTTTGGAATGGGAG-GCATGGAGAATTATATG 145770
Gallid          TCACTGTGTTCT-CGGTCCGGAGATCATTGTCGTTCCAGGTGCATGTGGTTCGTCCGATA 153495
                     ** *       ** **     * * * *     ** *    ***    *   **

Human           GATAATAACA-GTCCATGGAACCAATACTGTAAAGTTCAAGACATTGTATCC-CAAAACT 145828
Gallid          GATCGTAGCGTGGCGAAAGCACCGGAGCAGTCTCAGGAATTACAATGCGCCTGTAAAACC 153555
                ***  ** *  * * *  * ***    * **       *  *** **   *   *****

Human           G-------CTCCCAGGGAAAAGTTGTTTCTTCGACACCGGGAATCGCTCCT---AATCTG 145878
Gallid          GGCTAGGCTTCCGACATAGACGCGGTGGTCTGTCCCCCGAGTGCCATTCTTCTGGATCTG 153615
                *        *** *   * * *  **    *   * *** *   *  ** *    *****

Human           ATGAAGGGCAACGGG-TTGAATGTTTATGGCCATGTGGGGTGTGTAGATGCGGCCATTTC 145937
Gallid          ATGCAGCGCAATGTTCTTTTGCGTCTGCCGACGGGCGAGCTCGGTCAACGTAAGTGTCAT 153675
                *** ** **** *   **    ** *   * * * * *  **  * *      *

Human           CGATAAACAAGGTGGCACCGCTAACGTCGCAT---------------CTTCTCTATTGAA 145982
Gallid          TAACGGCCTGTGTTGACCCGCTGTCGTGGCGTAAAAACGAACATGGTTCATTCTCCTGTT 153735
                  *    *   ** *  *****  *** ** *                   ***  **

Human           TCAGGAACATCAAGATTGGATGCGGGTGACCGGGACGAACACAAATCTGTTA-AACAATA 146041
Gallid          TTACAGGAGTAAGGGATATTCCCGACATAGGGGACGGTGTACAGAGTTTCAGCAGTAGTA 153795
                * *      * * *  *     **    *  **   *   *** *  *     *  * **

Human           TAAATGCTGAAACAAAAATGGAGAACTATGGTTTTC-CGGAAAATGGGAATGTCCACGGA 146100
Gallid          TCGCTTCCGTCCTAGTAATGAAAATTTTTGTGCTCACCCTTTAATTTATCATCCCTTTA 153855
                *   * * *    *  **** * *  * * **  ** *     *    *   ***    *

Human           GCCGTCAAT-ACAGCTTTACCACTAACTCTATCCAGTGGTCAGCCCTACACATCTGTGC- 146158
Gallid          GCCATTCTTGACAAATTCGAATTTGACAACACATATTCACCAACTTCTAAGGTTTTTACA 153915
                *** *   * ***  **      * **   *   * *   ** *     *  * * * *

Human           ---CACAACATGGCGCATGTGAAGGAAATGGGACAATTCCCGTGGTACAGATCTGCAGTC 146215
Gallid          CCTCATAGCATG-TACACAATATACAAATGTGATGATCTCAGTGAAATG---CTATGA-- 153969
```

```
                   ** * ****   **    *   ***** **  **  * ***  *     **

Human              CCAATACGGCGTTCAAAGCGCACTACA---GTTTGCTCGGTACAGTGGATGAAAACAACC 146272
Gallid             CTAAAATGGAGGGAAATGTACATGGGATGGATGTGTACAGGACAATGTAAAGGAAGTACT 154029
                   * ** * ** *   ** *  **     *     * **  * * *** ** *     **  **

Human              CCTTGTCCGTCAGAGAGAGCATACAGGACACAAGTTTTAGTAATG-GATGTGCTCCTCAG 146331
Gallid             ATATATACAGATTGGAGGGCGAATTGAGT--GATTCATACTAGTTCGACACTTGTCTAAA 154087
                      * * *      *** **  *  *       * *  ** ** *  **        ** *

Human              TTGTCCTC--GCCTGGAGGAAAC-------------CCGACAATAATTGCACATTCAATG 146376
Gallid             TTGTTATGAGGTTTGGGGACAATATTTATTGACTAGTCTGTAAAGGTCGTGTCCCGATTC 154147
                   ****  *   *  *** *  **                *   **   * *        * *

Human              ATTGGAAATAACGGAACCCCGAATAAAGAT---GTATGCAAGCCTACCCCCA--GCCTAC 146431
Gallid             CCTATGTTTAATGGAGACATGTATGGAGGTTTAGTGGGGGGTCGAACTTTCATTGCCCGA 154207
                     *     *** ***  *  * **  ** *   **  *     *  **   **  ***

Human              GTGCAATAAAAAAATTAAACTTTGACT-----------------ATGATGACCGAGGGGA 146474
Gallid             ATGTCCGAATTTTCCTGGGCTGTGGTTTCCGGACCACCTTCCTCATGCTGTCCCCAGGTA 154267
                    **    **      *   ** **  *                 *** ** **   ** *

Human              AAATATAGGATTTCCTTCCAAACTGGCAGCCCTTCTGTCTATGGGAGAAAACATGTC--- 146531
Gallid             ATACAACTGCTTCGAATTGAGGCTACCATATCTTCTG-CTAGAGCGGAACATGTTCCCTC 154326
                   * * *   * **    *  *  **  **   ****** ***  *  *** *  *  *

Human              --AAAGATGGACAAT------CCTTGTTATGGAACTT-----CTCTAGCGCAGTTCGAGG 146578
Gallid             GGGAATGTGAACAACAACCGGCCTGGCCTCTGGATCTGGAGGTAATATTACAGACCGACA 154386
                      **  ** ****        *** *      * *  *         **   ***  ***

Human              AA--TCAC-ATCAGCAGAATGCATCG--GAGGGTAAAAT------ATCCATTGCGGATTT 146627
Gallid             AAGATTACGAGCCGTCGGATGTAAGAAAGAGGGGGAAACGATCGCATTCATTTTAGATTC 154446
                   **  * ** * * *  * *** *     *****  ***        ** ****   ****

Human              AGAGTTTTCTGAAGAAGAT-GATGTGCTTTCCAGTGCAGCCTCTGT-CAGCTGTAATGAC 146685
Gallid             AATCACATATATGGGTCTCAGGGGGGCACATAAACCCCATTTTCGTGCAGCTCGGTTAGA 154506
                   *      * *   *      *  * **      *   *    *  ** *****    *

Human              AACTGCGTCATGAAGATCGGAGCTTCGCAGCAAG-GTACCACTGTGGCTGATTTACAACA 146744
Gallid             ATATGTCTCGCCGCAGAGAAAGTCTGGCCTCACGTGTTTAACTGCAGCAAAATAAAAGAG 154566
                   *  **  **            **  * **  ** * **   ****  **  * * * *

Human              AGGGTTTA-AGCAACAAATGA-ACG------GAGAATTCTCCATGTTTGCAGTGGACGAG 146796
Gallid             GGGAGTAGTAGGAACGCAAGATACGTCTTCCAGGCGCGTTTCGACTGTATACCGGACATT 154626
                    **  *   ** ***  * ** ***        *     * *   * *  *  ****

Human              AACATTAAAACACAGGA-GATGTCGAATGACTGCGCGACAAACGTCACGG--ATAATGC- 146852
Gallid             GCCGGTACAACATAGATTGATGGT-ACTGTCCTCTATCCCAATTTCACAGTAATAATACT 154685
                     *  ** **** **   ****   * ** *  *    * **  **** *  ***** *

Human              ATGCGCCATTCGACAAAATAAACGT-------ATGCACTGTGAAATTGGGAT-----TTC 146900
Gallid             GTTCTAGATCCATGAGAGTTAATGGGCCGACAACATCCCGCGAGGTTATAATAGTGGTCC 154745
                    * *   ** *   * * * ** *        *    * * **  **   **     * *

Human              AGAG-----GATGGCCGGGTGCGGGAAGAGGAGAAGTGCTCAGACGTCGCAATTCACGTT 146955
Gallid             CGAAATCGAGAAGGCCGGGCCAGCGGACACAGCACTGACTACGGGGTGAAATTATACATT 154805
                    **      ** *******   * * * *    *    **  *  **   * *  ** **

Human              CCGCGGAAAAGTGCGCGTATTCA-TAACATGAAGTCCGAAGGCGTGACTTGCGGTATGTG 147014
Gallid             TCATGTAACGTGTCGCTTCTTCAATGATACATGGACCGTTTG-GTAGTCTATATCGATCT 154864
                    *  * **     *** * **** * * *    * ***   * **    *

Human              TGTGACGGCTGCG-GATTCCACACGGCAGGATGCGTCTGGAGGTTCGA----GCAGCGGA 147069
Gallid             TCTACCGTATCCATGATTATAAGTTTCTTTTTACGTTTCATACATTGACGTTGTAGGTAT 154924
                   * *  **  * *  ****  *     *    * *** *       * **    * **

Human              ACCAAGAAAGGCGAAAAGTTGCAAGGACTTTGGAAGGGATACCAGGATGACGACGATTCG 147129
Gallid             TCCACAGCACCCTGATAGCC-CAAATCCTCTGTTGATAAAAACAGGACTCCCA----TCC 154979
```

```
                 ***    *  *  * **   ***   ** **      * * *****   * *    **

Human           GAATTAACCGAACTGT--CGGATACAGACAGCGACAACGATGTCCAAA-ACTGTCACGGA 147186
Gallid          GAGTCAGTGGAGATATATCGGGTGCTGCTTTCCATCGGCCAGCCCACGCACTGCCCTTGG 155039
                ** * *   **  * *  *** * * *    * *       * ***   **** *   *

Human           GTAAGAAAGACCGGTTCCAAGACGTACTCCTCAG------TATTTTTAATCCTGACTAT 147240
Gallid          GTAAGCATAATCGGTTAGAAAATGCAGCAAGGAGGAATGATAATCCTCCTCGCTTATTAT 155099
                ***** *  * *****  ** * * *      **      ** *  *     ** * ***

Human           C--------GCCAAGCCAAGAGATTGCTTGCCGACATTCCATACAGAAGATGGATTCC-G 147291
Gallid          CAAACCTCAGCCCTCCCGGGGGAGTATCTGGAGCGGGTACGCAAAACAGGTGTTCGCCTG 155159
                *        ***   **  * ** *   **  *    * *  * *  ** **    ** *

Human           GACACATTCAACATGGAAGAACACGAG----------GGTCCATTCCTTCCAATAGTG-- 147339
Gallid          GGAGGGGTGAGTATAAAACAACGATAGACACATCATCGGGAGATTCTTGTTGGTATCGCA 155219
                *      * *  **  ** ***   **          **   **** *     **  *

Human           ----ACTAGACCACCCACTGTGTTT---ATTGGCGGT---AGACGCCGGAGGACTTACCT 147389
Gallid          TCCAAGCAGTCCTTGCTCTAAGCTTTGAAGTGGCTGTGTGAAATGCCTCAGATGCAGCGC 155279
                    *  ** **   * **  * **   * **** **   * * ***  **      *

Human           CCGGCGAAGTGTCACCTCTATCGGTCCTCTGAGTAAATTAACTTACTTCA--AAGAATTA 147447
Gallid          TAGTTATAATTTCGTTACTATTATATTTTCCAGGTAACGCATTTGCGACACGAGCGATGT 155339
                  *    * * **    ****      *   **  **   * ** *  **  *   **

Human           CTGC-AGAGCTACGTGTTGAGAA-ATAGCAACTGTTATTTAAGTATCGGTTGGCCTGCCA 147505
Gallid          CTGCCATAGCCTCGGGAGAATAGCGCAACACCTCTTGCCATGGT-TCGGGGATTCTGGCA 155398
                **** * ***  ** *   * *    * ** ** **      ** ****     *** **

Human           AACACAGGGTGT-------ATATCATGTCAGAGGAG--------------AAATTGGGG- 147543
Gallid          AACTCTACTAGTTCCACAAATGCCCCGTTGGGGCAGTATGCGTTCAGAACAGATTGGGGA 155458
                *** *     **       **  *  **  * * **              * *******

Human           ----TATAATCATATACCTACCTTGAGGGAGATGTTTCCACTGCCACCCGGATGGATGAT 147599
Gallid          GGGGTTTGACCGATCACCGTTCCAACCGGGAATTGCCTGAATGCGGCACGGTCCTGACGT 155518
                    * * * *    ***   *     **  **      * ***  * ***        *

Human           TGTCCTTGGAATTGTC--GGTTCAGA----GACGCCCGCGGCTCTGTATAAACACATGGT 147653
Gallid          C-CCCGTGGGCCTACTAAGGCCCCGGTCATAATATCGGTAGGACTATAGCAGGACG-GGT 155576
                   ** ***   *     **  * *      *   * *  *  ** **  *  **  ***

Human           GGTTCTCCTGTGTGAAAATAAATGGG-TGCTTCTGCATAA-----TTACCGGGACTCTAA 147707
Gallid          GGTATGGGCGTTTGGATGGGGACGGGCTGCTACTGCAAGAGCGGTTTGGAAGGGAACTTG 155636
                ***      ** ** *     * *** **** *****  *     **    **   **

Human           ACACGA-GCTGTATTTCGCCGCCTCGGATTTGAAACAGTTTATGGAGGAGGGTCTGTCTA 147766
Gallid          ATAGAATATCGTGGGTTGTGGTGGTAGGGTC-AAGCAGTGTTTGTCCGACCGACGGTTCC 155695
                * *  *    **   * *  *     *  *  ** **** * **   **  * * **

Human           GAT----------GTGACTGCATATATTAT--GAAAAGTCAGTGCCGTACGGCGTGGCCA 147814
Gallid          GGCATTGGGAGTGGTGTCTGCAAACCCTGTTTGTATTGCTCGGGAGCTGACTTGCGCTTA 155755
                *            *** ***** *   * *  * *  *   * *   *     * *   *

Human           TGGAAGACTC------TGTTCGAGAGTTCTTGAGAAACAGTAAAACGTTTCAGAGTCTTA 147868
Gallid          CGGGAAACTCCACCAATATCAGAAGGGGTACTAGACTGGGCAGATCTGCGTCGCTCCTTG 155815
                 ** * ****      * *  **  *      ***    * * * *      *   ***

Human           TGGAGTATAGGAAGAATA-TGCACGGATTTACCTGGACCTTTAACG--GAATGCCTGGTA 147925
Gallid          CTAGAAACGGCGGGTCCAGTGTATAAGTTCTTTCGTTCCAGAGAGGATGATTGGTCCATA 155875
                      *  *   *   * ** *    **     *  **    * *  ** **     **

Human           GACTGGGCGACAGAGTTATACATATCTG-CAATCCTGAATTGGT---TAACTCAATCCCA 147981
Gallid          GCCGAGGGATGGGCGGTGCTAGTTTCTGGCAATGTCTCATCCGTCTGTGCTTTAGCCGAG 155935
                * *  **     * * *     * **** ****     **  **   *   * *  *

Human           GC----AGACGAGGCC--ATCAGATAT------GAAGGAAAACCTTTGTA---TTTCTTT 148026
Gallid          GCTTTAATCCGAGTCTTTGTCGGATCTTTTAGCGAAATAGTATCACTAGAAGTTTTATTC 155995
```

```
                   **    *  **** *    ** *** *       ***  *  * *  *  *   *** **

Human              GCATTTGTCACCACTTTCAAATCACACCCCGGTAGCAAGGCCAACGTATTGATCGCTGCG 148086
Gallid             CCTTTTTTAGCCGGGGTTGGAGTAGAGCT--GCAACTGTGCCCATTTGCCGCAGGCTGTA 156053
                    * *** *  **    *   *  * * *   * * *   *** *  *   *   ****

Human              --GATAAGAACTTAGGGAT---ATACGGC-TATCATAAAGGTCGCCCCAGGATAC---GG 148137
Gallid             CTGGGCCGGACATTGTGTTCTCGTGTGGCGTTCCGGCAGGGTCCCTTTCGGGTCTTGGGG 156113
                     *    * ** * * * *     *  *** *  *   * **** *    ** *     **

Human              TACTTGTGCAAGAACGTACAGGCTTTTTTAGA--GCCGGAGTTA--GAAAGATGTAT-- 148191
Gallid             TGCTTAT-CGGTATTGGATCGCTTGTATGTCGGTGGGTGGATTTAATGGGAGATTTATCA 156172
                   * *** * *   *  * *  *  * * * * *   *  *** ***  *  **** ***

Human              ---------TTGGATTATGAGATTC--CATCGAAAACGTTGCTTGCCGTCAGCAAAGATG 148240
Gallid             GACGCAGACTGGGATGATGAAGTGGAGCGTCGGGGACGCC-CAGGACCTCGAAACAGCTG 156231
                            * **** ****  *    * ***   ***   *  * * **   * ** **

Human              ACTA--TCTGTGTACTCTACAAAAAGCTCCTTGTCTGCTCTTAAAAC--CAGCTGTGTTT 148296
Gallid             ATCGGGTCTCCGTGGTGGAGCAAATACCCCATAAGTGCCCTTTGAACTCCGCCCATCGTT 156291
                   *     ***  **  *  *  ***  * ** *   *** ***  ***  *  *  *  **

Human              AGAAAGACTTTCTCTCAGGA--AGGTAAATAAGAATTGCTATGTCAAGAGTTTTCCTTGA 148354
Gallid             AAGCAACTCCGCTGTGCGGCGCAGGTGCTTGGCCTTTACAAGGCCAGGA-TCACCATCGT 156350
                   *   *      ** *  **   ****   *     ** * * * ** ** *   * * *

Human              CAGTTCC-CACCCTCTTTTTTTAATTAAAACAGAAAGGCCATTGGATTCCATCCGCTAAG 148413
Gallid             TCGTTCCGCGCCAGTCTCGTAGTAAGAGAGAAGTAATGTCCCTTGACCCCATGCGCACCC 156410
                     ***** * **    *  *   *  * *  ** ** * *  * **  **** ***

Human              AGATTTACTGTTTCTTTGACATTTATCAGAGATACGTAATACTCGGTCTACTGTG-TATT 148472
Gallid             ACGCTCGATCTGGGTCTTGT-CTAACCAAAGGTCCGATAGTTTCTGCAATCAGTGATACC 156469
                   *   *   * *   * *     * * ** ** * **  *   ** *    * *** **

Human              GTGTACCTTGTAA---------TCTCTAGGT----AAACTTCTGCTATTTTTTTAATGTT 148519
Gallid             ACGCGAGCTGCAAGAGCACGCTCATCGAGATGTTGAGAGGTAGGCGTGTTTCCATACGCG 156529
                     *     ** **           ** ** *    * *  *  **   ***    * *

Human              GATTAATTGGACATTGTT-GATAGGTAAACTGTTCTTTCAAAATGACCAGAGATTCTATG 148578
Gallid             CCATAAACAGCTGCTCTGAGATCCGATAATTCATGGTACCAGGAGACGAGGAGAATACGG 156589
                      ***   *    * *  ***  *  ** *  *  * * *   *** **          *

Human              TACTAATCTCTTTGTTTTTTTTTTC---TGTGTATTGTGATAGGGAAGGTTTGTAAGGAC 148635
Gallid             CATTTCTCCCATTGTTGCAAATCGGGAACGTGCGGTGTAATACGCGAGTCGTAAAAAGAT 156649
                    * *  ** * *****     *       ***   *** *** *  **   *  ** **

Human              AAAGGACAAAGT--TCAGTAAGATTTAATAAAATAACATGTGA-CACAATTTCTG-ACTC 148691
Gallid             AACATTTTAGCTACTCTATGCGGCCCGTCTGGACCACATAGGAGCGCGGCCACTACACTA 156709
                   **      *  *  **  *  *          *  ****  ** * *     **  ***

Human              TCCC---------------TTTCAGTAATTTTTTT----ATTTTAATAATGTCCGTTGCC 148732
Gallid             TCGCAGGCCAAAACACAAGCATCTGCGAGTTCTTCCAAGGGTCTGGCTGCGTCGAATTCT 156769
                   ** *                 ** *  * ** **       * *      ***   * *

Human              ---ATAAGGTGAGGGCAACGTAGG----------GTCTGA----GGGATAAAAAAGTAAT 148775
Gallid             TCGACAAGGCAGTCGGGACGTGTGCATCCCGCAAAACTAAATTCGGGGTCATCAAGAGAT 156829
                      * ****     *  ****  *            ** *    *** * *  ***  **

Human              TCGTTCATATGAG---TAGTCGGAT--AAAACATTTGTAGTTTTTTTTCTAAAACATAAC 148830
Gallid             CCGTCCGCCAGACACCTACTCAAGTCCACAACACTT-TGGTATACGCGTCCATGGATTGC 156888
                    *** *    **    ** **   *  * **** ** * ** *        *   **  *

Human              GATTATCTCGATGACATT--CATACCGGAATAAATCCGAT---ATAAACGTTTCCATCTG 148885
Gallid             CGTCACATCAATTTTAGGGGCATGTGGGGGTGCGGTTGTTTGGGTAGATGTGGTTATCAA 156948
                     * *  ** **   *    ***   **  *      * *    ** * **    ***

Human              TAGATATCTTGGCCC-----GTTTTTGTGTGCTGACATAGCAC-TTTCGGTTAAATTTTC 148939
Gallid             AACCGATGGCGACGTTGAGGGTTTAGATGAGATTCCCTTTTCCGTTTCGGAAGAGTTATC 157008
```

```
                           *   **   * *       ****   ** * *  * *    * ******   * ** **

Human           TATCACAGCTCTCC---CGTAGTCACTTAAAAAAAAACAAAA-AAAAAGACATATGAAAT 148995
Gallid          TGGAAAAGCCATTGATGCATATGCTTTCTTAAGGGAACGGAACAGAAAGGTTTTTTGGTC 157068
                *   * ***  *     * **  *  *   **   ***  ** * ****   * *

Human           TTTATAGTAGGCCTACCTGCAATTTATATAAGTACGGATGAAAACAAAGAAAC----AAA 149051
Gallid          TTTGCAATA--CCTGCGTGTCATAGCTATACTCGCGGCTATATGCGGAAACGCCCATAAT 157126
                ***  * **  *** * **  **   ****    *** *  *  *  * *  *    **

Human           CATAGTGGAAATAAAATGATTCG---CATGTGAATTA-ATACCTAATGACTA----GATT 149103
Gallid          AATTTTCTCTCTTTCATGGCACCAGCTATGTGAGGGATGCATCTGTTAACTGTGCCAGTT 157186
                 **  *     *   ***   *     ******   *   * **  * ***        **

Human           GAAATCCATCCATGT------TACGGACC--AGGGTCTTTGGACTT------GGTCTTGC 149149
Gallid          ACAGCCGCTCCGGGCGAATCGTATGAACTCTGGAATTTCTGAACATATATGTGGTTTAGG 157246
                  *  *  ***  *       ** * **    *  * * ** ** *      *** * *

Human           ATAGTAT-------ATACAATTGTTCTATTGTCCGTGAAAGGTCGACAAAATTTCTGGCC 149202
Gallid          GCAGCATCGGCGGGAGACAGCT-TCCCGTGGTGCATCCA-GTTCAGAGGATTTACTTTCA 157304
                  ** **       * ***  * * *  * ** * *  * * **     * ** **  *

Human           TGCATTT-TGAAACAGA----CTGAACAAATATCAAGTTAT--AGATTAACCGGCGATGT 149255
Gallid          TGCTTTTGTGAAACAAAGAGTTTACGAGAGCGTCGTATTGTTTGGCATAATCTTTCATTT 157364
                *** *** ******* *      *     *   **   ** *   *  *** *    ** *

Human           CCTGTGTTATTACGCAGACTATACGCGTATACAT-CCACATACACGCACACACGAGAAAA 149314
Gallid          CCTCTACATAGACGGGAACTGGGCCATGGGATGTGTTGAATCTGTGGATAGCTTCTACTA 157424
                *** *      ***   ***   *      *  *     **    * * *       *  *

Human           TGG-TGTACTAATCATTCAAGTTTACCCGTGGTGAC------------ATTTTCCCCGAG 149361
Gallid          TCTCTGGACTATCCGTAAGTGCTTGTCGATGCCCACCAAGGCCACCGAACCAGCACCGGC 157484
                *   ** ****  * *    * **  *  **   **            *    * ***

Human           TAGATAGAACGACACTTACATTTCACCGTCTAGGCAAGTCGGAGTCG-AGGAAGATACAG 149420
Gallid          CAGGCGGTGGCAACGGAACCTCCCACCATACGCTTCCGCAGAGGTCATAGGGGGACGTGT 157544
                 **   *    *     ** *  **** *        *  *  ***  ***  **

Human           A-ACTCAGTGAATTAAGAACGACATAGTGATCACAGCACGAAGTAATCTTCGGTAACGTG 149479
Gallid          CCGCAAGGCCAGAGGAGGATGTCACCTGAATATCATTGCGTTGGACGGCTCGGCCGGACT 157604
                   *   *  *    ** * * **     **  **   **  * *    ****

Human           CTAAAAAACGGTAGCGCAGCCACTCCAGGATATGCATGGAAATG-TGTTAAAATATACAC 149538
Gallid          TGGGAGGACGCCCACGTGGGCGTCCTCCCCCACGCCTGTTTGCGGTTTCTTGGGGTAAGT 157664
                    *  ***    **  * *   *      * ** **     * * *        **

Human           AGCCACGAAATGCATAATTAT---CTAGCAGCAATAGTTTGTTAT--TTAAACAATGTTT 149593
Gallid          AGTCCTCATCTTCCAGTTTACGCGTTCGGGGCGATGTTTTTCTCCCCTTAGACTGCGGTC 157724
                ** *   *  * *    ***     * *  ** **  ***  *    *** **   * *

Human           TTTTATGTTCATT-TAAATATAGTATGAATATTTACA---ACTAAATATACAATCGACTA 149649
Gallid          CGGATCGCCCACCACGATTACTACCTCTGCCCCGACGCGGGCGGCTCGAACAATCAGCCC 157784
                      *  **     * **     *        **     *       ******  *

Human           TACACATGGTCGTTAACGCA--CTATTACATGCGTTCCAAAA---AACAGAGGTTTATCG 149704
Gallid          GCGGTCTGGGGGGCGACGGAGATGGTGACCTAGATGATGAAGGTGAAGACGGGACGATTA 157844
                      ***  *   *** *     * ** *   *    **    ** *  **   **

Human           GGAGTTATCGTTAT--CTACATCGATAAAGTGTACAA-------------GTCTACATGT 149749
Gallid          GAGAAGATGATGACGGTGAGGATGATGAAGAGGAGGAAGATGAAGAGGATGGCGATCTGG 157904
                *     **  * *      *    *** *** * *  *             * * *  **

Human           AAACAT-----------CACAGAAAAATATAGAA------GTCCGAGGCCGTGTACATCC 149792
Gallid          AAACATGTCCGACGTTCCGGTGAGTAGTATTGGATGGGGGATTCCGGGGAGGGGATATAA 157964
                ******           *   **  * *** * *       * *  **  * * * **

Human           AAACACGCACAAATAACGGTCAGGTTCTCACGACAGTAGGTTATGTAGCTGCCATTT--- 149849
Gallid          TAATGTGCTCATTTGACGGTGAAGT-CTCAGGGCCGCGTGGTCTGGGGGAACTTCGTGGA 158023
```

```
                 **   ** **  * ***** * ** **** * * *    * * **  *   *    *

Human           --TGATGTTCTGAAAGT--ATACATCTCTCGGGTTGTAAAATAC-GTGCCCACCCTCGCA 149904
Gallid          GATGAGGTTGTGTGAGGCAGTTCGCTCATTCTGCCAGAAGATGGAGTAGTCAGCCGTGGA 158083
                  *** *** **  **    * *     *   *    ** **   **   ** **  * *

Human           --TAAAGCTTGTCCGAGGTGTTCATATCCATATATTTTTTTTTCGAACATATTTTGTTTA 149962
Gallid          CCTGAGGTGAGTGGTGCAGGTAAGTCTCCACAGAAATCTTTCTTTGGCCCAGAACGAGGG 158143
                  * * *   **       **   * **** * *  * *** *    *  *    *

Human           CTATATCTCGCTTGGACAGTGAAGATGTTGTTAACCATCAGTAGTCCCGCATTTCTGAAA 150022
Gallid          GGACTTGGGCGCCAGTCTGTGGACGGATTATTC-CAATCTGGAACGGGAAGAGGGGGGAA 158202
                  *  *        * * *** *    ** **  * *** * *             * **

Human           CGTTGTTTTGAAGTTCCGGTCTGTA---------CAAAGATGGTGCTGCGGATTAAGAAA 150073
Gallid          CATGGGCTGCCCGTCATCGTCCATATTGGCGTTGCAACGGAGCTGCTGGAACCGCCGTCG 158262
                * * *  *    **    ***  **         *** *  * *****         *

Human           AACTGCATAAATATAAAAAAAAACTCGGGAAAAGTCATTTGTTTAATTATGGCCT--TTC 150131
Gallid          GGCTGC-TAATGTTACGGCAGCACGCCAGTCGCATAAAGCTTTGTATAGGGGCCGGACTC 158321
                  **** ***   **    *  ** *  *      * *    **  **   ****    **

Human           GTCTTCTAATCCAAAGAAAGTGAAATTA-AACGTAAAAAAAAACTCTAC-ATA-CAGATT 150188
Gallid          GCTTTTCGATGGCGGGACCGTACGCCGGGAACCCCAACTCGACCATCGCGATAGCGAGTT 158381
                *  **   **     **  **        ***   **    * *    * *** *   **

Human           GATAGTTTAACAGCGCTCACCATAATGCGGGACTC--ATATGGGTCGGACTGGCT---AT 150243
Gallid          GTTGGACCATAAAGGCATAGGAAACTTTGGAGTCCCGGTGTTCTCCAGACTCGCTTCCAT 158441
                * * *   *  *  **  *  * * *  **    *   * *    * **** ***   **

Human           ATCGTATTTA-ATATTAGTCGTTGTC---AGTTT--TTCTGGC---AAAGAGTGCCACAA 150294
Gallid          CTCCAGTCTGTATATTATTTCTATTCCTAAGCTCAACTCTAGCTTCGCGAATGGCCGCGG 158501
                 **   * *  ****** *  *  **   ** *    *** **       *  *** *

Human           TGCGCTTACGATGGCCGGATCTATGTTGCTGTGCATAACCGAAAAAGCAAAAAAAAAAAT 150354
Gallid          AGAGCGCAGGAGGCAGCGTGGTAGATGGTTGTGGC--ATCTCAAAAGTAACTTCGCAGCC 158559
                 * **  * ** *     *   **  * * ****    * *  ***** **       *

Human           AAATATAACAGG--TTACTAAAAAAAAAATTC-----GTTCCTATCAATCCCCT------- 150400
Gallid          AGGTATTACAGAGTTTTCTGTAAGGTAATTCTCGGGGGCCATCACAAACCCCTTACGTAC 158619
                *  *** ****   ** **  **   *****     *  * *  *** *****

Human           ATTATTTAAAC--TCACCGTA----------TAGATGTTCCTCGCTGTCCGACCAGTGAC 150448
Gallid          ATTATAAAAAAGGTATCTGTAATGCAGGCATTTAAAGTCTTTCCATGCCAAACCGGTCCA 158679
                *****  ***    *  * ***          *  * **   **  ** *  *** **

Human           AATATTTGTGAGGCAA-----GTCG--CATTCCGATGGTTTCCATCAGT----------- 150490
Gallid          GGGATTGGTGCTTCCATCTCGGCCGTCCACCCAGGTAATCTCGGTGAGTACGGGGCAATA 158739
                   *** ***   * *     * **  **  * * *  * **  * ***

Human           -TCCTCAGTTTCGTACAG--TGCATTTTTAAACGCTCT--------GTGAGAAACACAAG 150539
Gallid          ATTGTCGGAGTAGAATTGGCCGCAATGCCTAAGGCGGTCGGGAATAGTAGGACACCTAGT 158799
                 *  ** *  * * *  *   *** *    ** **  *        **  ** **  *

Human           TTAAGAAATCTCAAATATAAACTCTTTCAAACGATACATCGCAGATAGTATAAACTCGAA 150599
Gallid          AGCGGGAATCCCATACCAATCCTCATCCAGAGGAGTGCCTGCACCTGGTGGGGGTGCACA 158859
                    * **** ** *   *  *** * ** * **      ***  * **       *  *

Human           CTCACGTTCAAAGAAGAGAAAATAAGCTTACTTACAGTACGGAAGATAAATAATTCTTCG 150659
Gallid          TGCCGGAAAACACGGCAGGCCCTGAGCT-GCTTGCAGCT---GATATGAAAAATCTTGTG 158915
                  *  *   * *    **    * ****  *** ***      * ** ** ***  *  *

Human           TGTCGTTCTTATTCAAACG-TGAACACCATTCGTGACCTTGCAG--GGTTTGAAATCTGA 150716
Gallid          TAAGGATGTACGCCATATACTGGACAC-GTCTAAGACAACGGAGCCGGTCCAACAAACGG 158974
                *   * * *    ** *   ** ****  *    ***   * **  ***   * *   *

Human           CAAAAAAAA-GCGAATTTCCAAATTAGAATTTCAGAATTGTGTT---CCACTGCTAT-AT 150771
Gallid          TAGGAAAGGCGTGGAACCTTAAACACAAATTCGGGGGTAACACGAAGCCCCAGAAATCGA 159034
```

```
                 * ***   * * *     ***    ****   *  *         ** * *  **

Human           GGCAAAACCAATAAAAAAACATAGATGTAGTTTTTTTTAAAAAAAATTAGCATGAA-TCAC 150830
Gallid          AGAAAAGCTGATATAATATATGCATACTTGGGAATTTGGAGGGCGGGAGTAGGGAGCCGC 159094
                 * *** *  *** ** * **  **         ***  *       ** * *  * *

Human           TCA--CACGACGTAGCTAT-CGTTTGGACAATGATTATCGTTTATAG-------GGACGT 150880
Gallid          GCAGCTGCTTCCCACACACATGATGGGAGAGTTACCATACCCGGGGGAGGTACCGGGCAT 159154
                 **    *  *  *   *   * * *** * * *  **        *       ** * *

Human           ATGCGTCAACGACGTCCTGCCACGGTTCGAAATACTCAGTCCGAGTCCATGAGTTAGAAG 150940
Gallid          TCGCTTCTAGGGC-CCCAACCACAGCCACGAGACCCCAGGACGTTCTCATGACTCTCGGA 159213
                  ** ** * * *  **  **** *     *   * ***  **    ***** *

Human           ATTGTAACGTAGGA-CGATCATGGT--GCACGTCTCGGGGAG-----GACAACATGTGCC 150992
Gallid          AT-GCAGTGCATCATCGAGCACAGAAAGCACGAGTCGGGAACAGTGTGAGAACAACCAGA 159272
                ** * *  * *  * *** **  *   ***** *****  *      ** ****

Human           ATTGGCGGCAGGTTTAGAC-TTCAATATGTCCTCTAT--GCTAACGGACGC---GAAGCG 151046
Gallid          TTTTACAGCCCACCCAGTCCTTCGATAGTTCACATATAGGTTCTGGGAAGTCTGGAAGGG 159332
                 **  * **      ** * *** ***  **   ***  * *   *** *    **** *

Human           C--GTGCAGGTTTCCCAATCTTGCCC--GTCGTTGTCTTTTCGAACGATCGCAAAC-GAG 151101
Gallid          GAGGTGCATAATTGCACTCCACATCCCAATCAAACGGTTTATAATCGCCGTCAAGCAGAG 159392
                   *****   ** *    *    **   **      ***   * **    *** * ***

Human           GTTAACGTTTTGATAGTCTGTCCGCCATGGTTTTGTATCTTCAAGAGTTCGATAT----T 151157
Gallid          GGGGTTCCCTCGGCAGTTCGATTATC-CGGGGACATTTCTGGGGGGAGCTAGTATGGGGT 159451
                *        * *  ***  *     *  **     * ***    *       ***    *

Human           GACGATCAGTTCGC------TGGATTTTGAGTGGATGAATA---CATGTGTGCCGCACAA 151208
Gallid          GGGGGTAGAATCTCCCTTAATAAAGCTTCCGTTGCCAAACAATCCCAGTAGGCAGAGCAG 159511
                *  * *    ** *      *  *  **  ** *   ** *   *  **  ** *  **

Human           AGAGGCAAGATACAAGGAACGAGAAAAAATTTCTTGTTACGAAGGCTTCCAGCCC--CA 151266
Gallid          CGGTGGGTATCTGGCAGTGTTAGGAGAAAGCTTCTCAT-GTTTGTCTCCCTGCCCGTTT 159570
                 *  *           *    ** * ***  ****  *      * ** ** ****

Human           TAGTTTCTGCTCTGAAAGGATGTAGGTATGCGTGTGGGGTTGACACGTATGCGCGTA--- 151323
Gallid          TAGTTCCTCGGCAAGGGGCTGAATCCATCGGGATCATGGAGCCACAATTTAGCATAACT 159630
                ***** **      *  ** ** *   **  *  *   *  * ***   *  ** **

Human           ---------------CATGGGTGTGGTCTGTCTTCTAAGAGGCGGTGGAGTGGAGGGTGG 151368
Gallid          ACCAGGCAAATGTTTTATCAGTTCGACTTCTTCTCTGGGGGGAGATGGGATTGCAACGGA 159690
                                **  **  *   * *  ***  * ** * ***  * *     *

Human           AG--GAGAAGTGCTGCGACATTTTTAGAGT--GACAGGTGCATCCGAGGG--CGGGGCGG 151422
Gallid          AGTTGACAGGCGATAC-ACATACCGAAAGCCGGCCCCCCGTACTCGGGGGGCTGCAACTT 159749
                **  ** * * * * * ****    * **   * *    * *  ** ***   *   *

Human           GGTGTATAAATGGGACTCTTTTTAGTGAATT-----CGGGATATTTATACGGCGGGGATC 151477
Gallid          CCCGTCTGAAATCTGCGCTCCGACCTAAACTAGGGACGGGACAACTCTGCGGCTGGGATG 159809
                   ** * **     * **      * ** *     ***** *  * * **** *****

Human           TTAC-GCTTTTCTATGTGAATAAATACACAATGCATGCCTTTCCTTGTCGATAAAGGAGA 151536
Gallid          AGATAGAATGACGAGGGGGGGTCCGGGCTTGGAGCAAG--TTCTCCGGGAGACAAAGGAGG 159867
                  *  *  *  * * * *  *     *     *** *  **  *  *  ** *******

Human           CGTGGCGGGAAGGCACAAAAACTTGTACAAGTACTCACCATCGAGCCTTTTCCAGTCTGT 151596
Gallid          TGGGG-ACGATGGGGGTGATACGGGGGCGGGGGACAATTGTGGAGGAGAAGAGAGGGGAG 159926
                 * **   ** **     * **  *  *  *     *   * ***        **

Human           AGTTTTGGCGTAGCTCCAATTTACTAGTCATTGGACAAAAAGCTATCGCCGTCATCGCGG 151656
Gallid          AAGAGGAGAGGGGAGAAGGTGCAAGGTGCGAGGGGGAACAAGAACTGGAGGAATGCGCAG 159986
                *      * *  *      *  *     *   **  ** ***   * *  *    *** *

Human           TTAGTG-----GTTTCAGGCG--CTCCATATAGGCCAAACTATTATAGTTGCTCCCGAAA 151709
Gallid          GCGGAGACGGAGCCTGGGGAGGGCTCGGGGGGGATTGGGGCTGTATAAATTCGACCGGAG 160046
```

```
                   * *     *  *  ** *  ***       *         ****   * *  *** *

Human           GCGCCATATAGCAGAGAGAAGTGAAGGCCGA-GGCCGCTAGAGAGAATCTGTGATAGACC 151768
Gallid          TTATTGGAGAAAATATCAAGGGGAGGAGCGCTGGGGGCTGGACGGGAGAGGAAATGGGGG 160106
                       * *  * *   * * ** *  **  **  *** **  * *   *  ** *

Human           CATAGGCATTAATCTGTTTTTCAAATTTCTCACTCCTCTGCTTTCGGCCGAGAGAGG--- 151825
Gallid          CAGGTGAGGGAAGGCATGCTGGGGGCTGAGTGGGAGATGGAATGGGAGCAGGTGAGGTGG 160166
                **   *    **    *  *      *            *  *  *  *  *  ****

Human           ----AACGCAGATTATGATATCTCCGAACATGATGCTGCGGGGAATTAGCTATGATTATT 151881
Gallid          AAGTAGGGGGGGTAGGCGAATTTGTGTGCAAGAT-TTGGGCAGAAGCAGTGGACGTTTCC 160225
                    *  *  * *      ** *  *  ** ***  ** *  ***  **      **

Human           ATATAT------TCATTTATTTGTTATCTCGGCGGATTCCGACATCGGTGTCCGGGAGAA 151935
Gallid          GGGCAGAGTGGATCAGAGTTTGACTGGTTCGCGGGAGCTAAATGCGGGTATC--GACTAT 160283
                    *       ***    **   *   ***  ***     *    *** **  *   *

Human           TGAGCGCGTGGCAAATAAACAAGACGAAATAAAAT-AAAACGCTAGAT-CCTTACCGTGC 151993
Gallid          CGAAAGC-TGACCAGAAAACGGGAGAGGGTGCCCTCGAGGCGCTCGCCGCCGTATCTGCC 160342
                 **  ** ** * *  ****  **     *    *  *  **** *   ** ** *   *

Human           CTCTGTCTCTATGAGACAACCGCAGCTGTTATCATCGCAAATGGCGTAGTTCAGGAGGTC 152053
Gallid          AATTGCCTCTCCGAGGCCCTATTGACCGAC----TGGGGACTCAACAAACTAGGAGTTC 160398
                   ** ****  *** *        * *      * *  * *     *    ***** **

Human           TTCTATATCTTCTAAGGTAGTAGTTGGGGTATAATCTA---CGGGGACAAAAACTTTAAG 152110
Gallid          ATCTATGTCCTCTGAAGTAGTGGGGCTTATTTCATCCATTCCATAGAAATAAGCCTTGAA 160458
                 ***** ** *** * ***** *      * * *** *   *   ** * ** * ** *

Human           GCCTCCTAGACGTGACATTTCGGAGGTAG--AAAATTTAGAGATGATTTCGGGTAGCGTT 152168
Gallid          ----CCTGGGATCGGCTCTCAGATAGTAATCGGGATCTAG---CGCCTTGGTGTCCTCAC 160511
                    *** *    * *  *  *   ***      ** ***    *  ** * **

Human           CGTCTTCTGGGCGTAGGATTTGTACCGTCGTAAATGTTAAAATAAATGCTGTCGTCATAG 152228
Gallid          CAAAATCGGCTGCCAGGATCTCCAGTAGAGGAGGACTGGATGTCC-CGCCGCTTCCGGAA 160570
                *    ** *     ***** *  *     * *    *  *  *    ** *    *  *

Human           TATTTTGCGGTATAGTTGTCTTCGTCGT--CCGGCGGCGGTGTGGATTCCACGG-TTCCC 152285
Gallid          GCGGACTCGATGTCCCCGGATTCGGGGGAACCGTCAACAG-ATGAACGATAGGAACTAAC 160629
                       ** * *    *  ****  *   *** *  *  * ** *    * *   *  *

Human           GCATCTCGGTAGACCGTAGCATTCGACAGGGCTGTCGCCATGGAAATCGCGCACACGAGT 152345
Gallid          GCGATCGGGGTCGCAGTAGCTATCGCAGGGGCTATAAGAAGGGGAA-CGGTCACTGGAAC 160688
                **     **    * *****  ***   ***** *    * ** ** **  ***  **

Human           ATCGGCGCAGAACATCTCGGCGGTCTCA----TAGCGCTTAGCGTTGCGGTCGCCGT--- 152398
Gallid          TCTCACCAGATTCATCTGCGCCGTTTGAGGAGTTACGACCGCCGGGGCGATTGCTGGAAG 160748
                     *      *****  ** ** * *    *  **     **  *** * ** *

Human           TCTCCGTCTTAGT-CTTGCGAAAATGC-----------GTATCCGCGGGAGGAAGAG-GA 152445
Gallid          TATCGGTGGTGGGGCCCGCGGTATCGCAGACTGCTCGGGTGGCTGCGGGAGGGGCAGCGG 160808
                * ** **  * *  *  ***  *  **           **  * ********   ** *

Human           CAGAAAGGAAGATGG-TGTTAGATAAGATCTACAGGAGGAGAAACGGGAGACAGATCCTA 152504
Gallid          CGGCAACGCCGTTGGACGTCGAGTGCTGTCCAGGCCGACCGGAGCGTGGGCTATGGGCAG 160868
                * * ** *  * ***  **    *    ** *        * * ** * *  *    *

Human           CGAGGACGGGAGAGCATAATAGAGCGGTTTGCATCCGGGAACATTCGCGTGAATGTAAAG 152564
Gallid          CGGGGACGGGGCCGCTGGAAAGCGAGCGCTGTCCATGGTAACTGGAGTGGGGGCGGTATG 160928
                ** *******   **   *  ** * *   **     ** ***    * * *   *  * *

Human           CCACAAGAAATTAATGCAACTAGAGACTTCCCAAAGATGATATATGGCTACGAGCAACGT 152624
Gallid          T-ACCCGA---TTGTCCGGCTAGCGATGGTACGGGGACACAGGGTGTTTGC-AGCGA--- 160980
                  **  **   *  * *  **** **     *   **       **  * * *** *

Human           CTGGCGCGTTCACACTCGTTTACCGTGTGTGACTGAGACACGTAGT----CTTCAATCTC 152680
Gallid          --GACGCCTTGATAAGGGTCCCCCG-GAGTGGCTGGCGTATGTGGAAAAATCCCCACGTC 161037
```

```
                    * *** ** * *   **   *** * *** ***    * ** *        * *  **

Human             TGCCTTGTAAAATCACACGTGGGTGGAAAGATCGTGCGGTGTATCGTCGTCGGGGAGCCT 152740
Gallid            TTCGTCAAAAGCTGCCAACAAGCTGTCGAAATCAGGCGGGTTGTCCATTTTTAGGGTTTA 161097
                  * * *   **  *  **    * **   * ***  ****  * **    *   **

Human             ACTCGGAGATAAGTCTGGGGCGATCATTTTTATCTGCGACGATATAAAACGGGGGGGGGG 152800
Gallid            GGAGGGGCGCAAATAGATGGTGGGTGGGCCGATGTGAGGGGA-ATACGGAAGGCGTAGCT 161156
                      **    ** *    ** *         ** ** *  ** ***     ** *  *

Human             TGGTATGAATCGATAACGAGGAAGAAATACATCTGATCGTCATTTGGCGATCAGTAGTTT 152860
Gallid            GTCTATGACTCCCTT-TGGGGGTCTCGTGGAAATGGCCCTCCCATGCTAGACCACAAGAT 161215
                     ***** **  *   * **      *  *  **  * **   **     *   *   *

Human             GTCGGTGATTTCTTCCGTAACCGGCGTGTGTGTAGACTTTAGTTTTGGTG-GTCAGATAT 152919
Gallid            GTGGGGGGAGGGAAGC-TA--CGGTTCAAGTGCGGA----AATTTCGATGTGCTGAAAGT 161268
                  ** ** *        * **  ***     ***  **    * *** * ** *    *  *

Human             TAAGTCTTGATCACACGCGT-ACCCGCGCGTTTGTAGCGGCTTCTCGGGTGTCTTATTTT 152978
Gallid            CGAAACATAAATGTAGGTGTCAGCTGCGGATATAATCCCGAACAGCTAGCATCTGGTTTT 161328
                    *  * * *    * * ** * * ***  * *    * *     *  *  ***  ****

Human             CACCCCGCAGGGGCTGAACCGCACTCCGGTCGGGCCACTTCTTTTCATACAGGCGTTGTC 153038
Gallid            ---------GTATTTAGGCCATGCTCCAAT---ACCAGGCCCGCTC-CACAG-CATTGT- 161373
                           *    *   **   ****  *    ***   *   **  **** * ****

Human             TGTCTGCTCCGGGGAGCTGGGCTGCGCTCGGGCCGGGACACTTATTCCCATACAGGCGCT 153098
Gallid            TATACGTATCTGATTGCTGCCCGAGATGGATGTCGCGGCTAATGTTCTCGATCCCCCCCC 161433
                  * *  *   * *   ****  *         * ** * *   * *** *   *   * *

Human             GCCTTCTCTGCTTTGGGAGGTC---GGACTGCGCTCGGGCCGGGACACTT------ATTC 153149
Gallid            CCCCCAATTGCAAAGTGCGGTATCAGGTTTGTACCCGCGCCGGTGTACTTCAAATAGTTT 161493
                   **     ***   * * ***    **  **  * ** *****   ****       **

Human             CCATACAGGCGCTGCTTTCTCTGCATCGGGAAGCCGGACTGCGCTCGGGCCGGGACACTT 153209
Gallid            AAATGCCAGCATTGCATTTCTAGCAC--ACAAACTAGACCG-GCCCACCGAGTCAAATTG 161550
                    ** *  **  *** **    ***     ** *  *** * ** *     *  * * *

Human             TTTGTGCGATACTGTTGCTCTGGAAGACAGATATCCTCGCGTTTCAAA-AATTACTTTA 153268
Gallid            TGTCCTGCTGAACGATTGCATAGTTTG--AGATGGCCATGCCCAGTAAACAAATACTTGA 161608
                  * *  ***   **  ****   *   *  ****  **  **     *** ** ***** *

Human             AACTCCCCGGGGGGGTTAAAAAAAGGGGGGTATTAACCCTAACCCTAACCCTAGGCCCTA 153328
Gallid            ATACCATCGACTAAGC--ACAAAAGGCTCTTATGATCTACTAGCAGAGTTGTGAAGTAGA 161666
                  *   *  **     *   * ******    *** * *    * *  *    *       *

Human             ACCCTAACCCTAACCCTAGGTCTAACCCTAACCCTAACCCTAGGTCTAACCCTAACCCTA 153388
Gallid            TGAGCGATTGTGGGGCCCAGCC-GACCAAAGCCCTGTAGGCAAACATTCCTGTACTGCCG 161725
                        *   *    *   * *  ***  * ****      *    *  *  **   *

Human             ACC---CTAACCCTAGGTCTAACCCTA-ACCCTAACCCTAACCCTAGGTCTAACCCTAAC 153444
Gallid            GTTAAGCAGATTAAACACTCATCGCTAGAGCCGCAAATTATTTCCCGATGACAACTTTAT 161785
                        *  *    *     * * *** * **  *   **   *  * *   * * * *

Human             CCTAACCCTAACCCTAACCCTAACCCTAGGTCTAACC--CTAACCCTAACCCTAACCCTA 153502
Gallid            GCGGCGGCTGCGGTTTGTTTTAATTA-ATACCTAATGGGCAAATATTCACGCTC-CCACA 161843
                   *     **     *     ***    *   ****    * **   * ** **  **  *

Human             GGTCTAACCCTAACCCTAACCCTAGGTCTAACCCTAACCCTAACCCTAACCCTAACCCTA 153562
Gallid            AAATAAACACAAATC--ACCCAGTGTTCCCACATCAAAAATCGCCATGTAGGGAAGATGG 161901
                       *** * ** *  * **   * **  **   **   *  ** *      **

Human             ACCCTAA--CCCTAACCCTAACCCTAACCCTANNNNNNNNNNNNNNNNNNNNNNNNNNNN 153620
Gallid            ATTTGAAGATCGCAGACTCGACAGGGAACGCGCTCAAAAAAAAAAAAAAAAAAAAACGGA 161961
                  *    **   *  *  *   **    * *

Human             NNNNNNNNNNNNNNNNNNNNNNNNNNNNNNNTCTGGCCCTAACACTACCCCTCTTTCAACCA 153680
Gallid            GGGATGGGTCTTTATTCAAAGAAGTGTAAAA-GGTAATGCGACCACTCGAGCAGCAAACA 162020
```

```
                                                **   *   ** ** *      *** **

Human           CTCACCATCACCCCCCCNNNNNNCACACACACAACACACACACCGCCACCGCT--ACCAC 153738
Gallid          TTAACCG-CGCGCTTATGGATACTACGAGC-TAGCACCGGGAAAGGCAGTGCATGAGTCC 162078
                 * ***  * * *           **   *  * ***    *  * **  **   *   *

Human           CACCTCTGAACTTCACCTTTTCCCTCCAT---CTCGCCCCACTTCTCTCTACACTTCTCC 153795
Gallid          CGTTGATGAATATGGGGGGAGGGGGGAATAGGTCGGTAAAACTTGTGCCCGCAGACCTCC 162138
                *     ****  *              **      *    **** *  *  **   ****

Human           GCCCCTCTATTCTTACTCCTGTTTTCTAGGATGCCGCTGACGGCGCGTGCCGGCCACGCC 153855
Gallid          GACA-TGAACAGTAGACTGTTTAATGTAGAACGAAATCGAGGAGCCATACCGGATACATT 162197
                * *  *  *   *      * *  * *** * *     ** *   * * ****  **

Human           CTGCATCGTCTTCCGCTCTCCCACTATTGGTGGCTACTG--TTGGGTCGACACTCCCTTC 153913
Gallid          TTGCGGGCAAGTTCGCCATCCGGTTTTCAGAAAAATCAGACTTTTGCTGA-ACATCGAGA 162256
                 ***       * ***  ***   * *  *      * *  **  *  ** **  *

Human           GTCATGTCCATTCCTACCTTCGCCTGCACAAAGGTCTACGCCTTCCTTTACCTTGGCCCG 153973
Gallid          GTATTATTCATCACAAATTAAAAACTCGGGCATGTCTG-ATCCTTCATGAGCTGGCCAAG 162315
                **  * * ***  * *  *       *    * ****    * * * * * ** * *  *

Human           AG-CAAGAATGCCTGCATTTACATCCTAAGCCTTACAAGTTTCTCCTGCGTTACCCTTGT 154032
Gallid          AGGCGACAGTAAAAAAAAAAAAAATCGAAGGGGGGGAAACATT-----GCTCGATAATCGC 162370
                ** * * * *      *   * ***  * *      **  **     **   *   * *

Human           CTAACAAGACAACCGCATCTTCTTCAGGGCTGGCCCGCGGATTC----TTCTCTATGTGA 154088
Gallid          GTAGCCAGACCGCACCATATTCTCC----CCCTCCCGCAAATTCAGAATTGTATAAGAGA 162426
                 ** * ****  *  *** **** *    *   *****  ****    ** * ** * **

Human           GTG-----ACATTTACACTTCCACTTGT-TCACATGATTTATTGTGTTTTGTCTGCTACA 154142
Gallid          ACGTAGGAAAAAAAAAAGGAATATACATACCACACACTTTGATATTTTGGATGTGTCCCA 162486
                  *     * *   * *     *    *  ****   ***  * **   * **   **

Human           ---CCAAGCACATTTCGGTTTCTCTTTTATACATTTGTCTCTATTCTCTCGGTCTCAGGG 154199
Gallid          GGGCAATGGGAACAGCAATCACTTTCGAATTCCTTCAGCCCAAAGCAC---AACCGCGGG 162543
                   * * *   *   *  *  ** *   ** * **   * * *  * *     *   ***

Human           TTCGACCCTAAACCCTACCATCCTTCG-GCCGACAGCAAGTTGCTACCGCTGGGCCTGAT 154258
Gallid          TCCACCGGGACTCTCCGACATAGCTCGAGCCAAAAGGGAATTGTAGCC--CAGGCAAGTG 162601
                * *  *   *  * *   ***   *** *** * **  * ***   **    ***  *

Human           CACGCTGTCCGCCTTTTCCATGCGCGTTTCTGAGCCGACACACTGCAGCGGCTTCCACGC 154318
Gallid          CAATGTTTTAATTTGTTCTTCTCCCTCCCCCACATAAAAAAACCACTGGATCGTACA--- 162658
                **   * *     * ***    * *    *       * * **  * *   * * **

Human           GGCACATCCGTCTCTCAGTTGGTTAACGGGAT-CGTCCCCTTGGCTC-GTGCTCCTACAA 154376
Gallid          AGTATACGAGTATATGGGTGGGGTGCCGTTTTATATAAACACAGCTTAGTTTGTTTGCCA 162718
                 * * *   ** * *  ** ** *  **   *   *   *   ***  **     * * *

Human           GCGCCAGGAGGGTCTCTGTTCTGCCACGACGTGTTCCAAGGCCGACTCTATCTCCTGTCG 154436
Gallid          CGTCAAGGAAGGGCGGTGCATATCTGCAA-GTAAACAAAACTCGGGGTTCTGTACGATTG 162777
                   * **** ** *  **     *  * * **   * **   **    * * * *  * *

Human           CACTCCGTGTCGCTCTTTCTAAAGACGGGCCTTCGCCAGTGTGAGGCCATCTATCGCGCA 154496
Gallid          GCCGGGGTCTTACATGCTCGCCCAATTGGATTT-------GAGAATCAATCTTCCGACGG 162830
                  *   ** *  *    **     *  **  **       * **  * ****  **

Human           CCGCTGTGGCGCGTACGGCCCCTGCCGAGCCTATGGACGTGTCGAGATCCCGACACGGCC 154556
Gallid          GTTTCCTGACTTGAACAGGGGAAAGGGGGAGGGGGAGTGTGTT---ATCTTGTCGCGAAC 162887
                      ** *  * ** *        * *     *   ****    ***  * * **  *

Human           TTCTTGCCGAAATTACTGGC-----GAGAACCGCCCGACGCGGCCTGGCCGCTTTCTATG 154611
Gallid          CAATAAAATAGATTTGTGGCCTAACGAGTTCTCTTTTTTTTTATATCGCAAGTGTTAACG 162947
                   *     * ***  ****     ***  *              * **   * *  * *

Human           CCCTGTGGAGACTG--CATCTGGGATCCCGCTCGGAGCTCTCTCACCC-------CGTGT 154662
Gallid          AAGCTATGGGACTAGTCTTTTCGTACAAGTCTCAGACAAACCGCGACCAAAAAAGCGCGG 163007
```

```
                        * ****   * * * * *     *** **     * *  **       ** *

Human           TGGAGTGGGAGAGAACAGAGCTGGTCCTGACGGATCAGAGACGCAGGTGGCCGTGTACGC 154722
Gallid          CCCTGTCGAAGAGGAAATACTGAATC--GACGAGCTAACCACAGCGTGTCTCCTGGGTTA 163065
                    ** * **** * * *     **  ****    *   **   *     * **

Human           ACCTCCTGTCCGGCTCGGAGT--TCCAGCGCGTTTCCTCGAGTGACGCCGGA--GACACA 154778
Gallid          ACCTCTCTATAGGCGGGGGGAAGTCCCTCTTATATTGGCGAGTGGCCTAGGATTGAATAT 163125
                *****      ***  ** *   ***  *   * *   ****** *   ***  **

Human           TGGAACGCAGCGACAGAGAAGGCGGCGGGGGGAAAAGAGGAGGCGGAGAGAGGCGGGCGA 154838
Gallid          TAGACCTCAGTTCCCTACGGCGCTTTGAGGTAGAGGGAAGTTTTCAGAGCTTGCATATGC 163185
                * ** * ***    *  *    **   * **   *  ** *            **    *

Human           CAGCAAGCCACTGACAGACTCGCAAGTCCGCACCTGACGCGGGGCCTCCGCGACTCCGGT 154898
Gallid          AAACGAGATGTTGTAGGG---GAAAAAAAAAAGAGGAACCGTGCCTTTCTCTACGCAGAT 163242
                 * * **    **    *    * **      *   **  ** * * * * * * ** * * *

Human           CGGTCCCTTCAGGGTG-AGGAGCCCAGCGCCGCGGAAGACTTTGCGAGGTGCAGACCGCT 154957
Gallid          GGGTCCCCCCCCCCCCCAAAAAAAAGGAACCGTG-----CCTTCCTCCGCGCAAATAGGT 163297
                 ******  *        *  *     *  *** *     * ** *   * *** *  * *

Human           GCTGGACGAACTGTGCGGGGAGGGCGGCTGGCTTCCCTTTGCGTTTCTCACGGCATCTCC 155017
Gallid          GCCCCACGAGGCCTCGGGGTCCGCGGGGCGGAGAGGGGAAAAAGAGTAGACCTCAGTTCC 163357
                **   ****    *  ***   *  **  **                     **  **  ***

Human           GCACGTCTGTCTGATCCTAACGGAGGGAGGCCCCGTCCTGGCGCTTG---ACCTGAACGA 155074
Gallid          CTACGGCGCTTTGA----GGTAGAGGGAAGT----TCTCAGAGCTTGCATATGCAAACGA 163409
                  *** *  * ***        ****** *     **   * *****   *    *****

Human           CACCTCCCTGTGGCGCATCGCGGACGACTTGGAGCTGCTGCTGCGCCTGGGGAGCCTGCT 155134
Gallid          GATGTTGTAGGGGAAAAAAAAAGAGGAACCGTGCCTTTCTCTACGCAGATGGGTCCCCCC 163469
                 *  *    * **   *      ** **   *   **    ** ***    **  **  *

Human           CCTGCTCTCAGGGCTCCGGCTTCCTCTCCGTCCCCCGAGCGGGAGCGGCGAGGCGGCGAG 155194
Gallid          CCCCCAAAAAAAAAGGAACCGTGCCTTCCTCCGCGCAAATAGGTGCCCCACGAGGCCTCG 163529
                **  *    *          * * *  ***  * * * *   ** **  *  *  * *  *

Human           AAAGCCGGGGTACGAGAAGGAAGAGGGAAGAGGGAGAGCGGCGACGGCGAGCGCGACGGC 155254
Gallid          GGGTCCGCGGGGCG-GAGAGGGGAAAAAGAGTAGACCTCAGTTCCCTACGGCGCTTTGAG 163588
                    *** **  ** **  *  **   *     **   * *   *     ****   *

Human           CGCGACGTCGCCGCGCAGA-CCGACCCGTCCGAGGGGGGTGACGGAGAAGGGACGTGTGA 155313
Gallid          GTAGAGGGAAGTTCTCAGAGCTTGCATATGCAAACGAGATGTTGTAGGGGAAAAA----A 163644
                   ** *      * **** *   *   * * *  * * **  * **  *  *      *

Human           CAACCGGGGACGTCCCTTTCTCCGCACA--------TCCCGAATCTGAGGAACAGACAGA 155365
Gallid          AAAGAGGAACCGTGCCTTTCTCTACGCAGATGGGTCCCCCCCCCCCAAAAAAAAAGGAAC 163704
                 **  **   *** ********  * **         ***    *  *  ** *   *

Human           CG-GCCACCACGGGCGCCAGGAAAGCGGCCACG-------GCGACCAGCGCGGCGGGGAC 155417
Gallid          CGTGCCTTCCTCCGCGCAAATAGGTGCCCCACGAGGCCTCGGGGTCCGCGGGGCGGAGAG 163764
                ** ***  *    **** *  *      *****       * *  * *** ***** **

Human           GG----ACGAGGACACCGCGATGACGGCGCGCGCCGCCACGCGAATGACGAAACAGAGCC 155473
Gallid          GGGAAAAAGAGTAGACCTCAGTTCCCTACGGCGCTTTGAGGTAGAGGGAAGTTCTCAGAG 163824
                **    * *** * *** *  *  *      ****    * *   * *       *  **

Human           CCAGCA-GCGCGGAGAGCACGAGGACGGGGAACAGACCGACTCCGGGCGCGAGGAGG--- 155529
Gallid          CTTGCATATGCAAACGAGATGTTGTAGGGGAAAAAAAAAAGAGGAACCGTGCCTTTCTCT 163884
                *  ***   **  *    * *  *  ****** * *   *       ** *

Human           ACGCACAGGAGAGCGAGGTCGCAAGAAGAGACGAGAAGGGAACG-------GAGCAGGGC 155582
Gallid          ACGCAGATGGGTCCCCCCCCCCCCCAAAAAAAAAGGAACCGTGCCTTCCTCCGCGCAAATA 163944
                ***** * * *  *      * *   ** * *   ***  *  *         * ***

Human           GGTAGCGGAAGGAGCTGCGGGA---GGGCAACGCAGACGTACGGCGGGAGA------GGC 155633
Gallid          GGTGCCCCACGAGGCCTCGGGGTCCGCGGGGCGGAGAGGGGAAAAAGAGTACGGTTCAGG 164004
```

```
                ***  *  * *  **  ****    * *   ** *** *       *   *       *

Human           GAACATGGTGCCTGGTCGTCGATCCCCCTGTCTGTCCCCAGGC-CCGATCCCCGCGTGTG 155692
Gallid          GGATATGAGAACAGCTGCGTATTTTCCCCGTGCATCTCATACCGCCCATTTTTGGGTAGA 164064
                * * ***    * * *      *  *** **   ** *    * ** **    * **

Human           GGTCCCTCCTCCTCATCTGTTATTCCCTTCCCCGCTGCCGTCGATAACGCCCGTCGAAGA 155752
Gallid          GGTATATTTTTATTAGCCAA-ATCGTATTCCTGGAAGT--TTGACAAAACCTGTCAAACT 164121
                ***   *  *  * * *    **    ****  *  *   * ** **  ** *** **

Human           CGAGCCGTCCGCCCGCC-CTCGGTGCCCGCCAGGTCCCGCGGAGGAACCCTCTAAGTGTT 155811
Gallid          GACACGGTCCAAGCGAAACTCGAAAA--AAAAGGGGGGGGGGGAGAATATTCTGTAGGAC 164179
                    * ****   **   ****         ***    * **  ***   ***    *

Human           CTCCGTGCCCGCCCTGCCCGTCGCCCGACGCCCCGCAGTCCGCTGTCCCGCGCCTCTCCG 155871
Gallid          CGGCAGAACTTCTCAAGGCAGAGGAAAGATACACATTATTTTTGTTAGATTTAGGCAAG 164239
                *  *    *  * *    *   *        * *    *    ***             *

Human           CTCTGTCCGTCCCCTCGCCGTCCACCGCGCGTGTCCGTTTCTCCCTCTCCTCCCTCTCCT 155931
Gallid          TTTTGCAGAACCTGCAGGGAATGTATACACCATCAAATCTACTCGACTTATTGCTTGAGT 164299
                 * **     **    *          * *       * *   *  **  *  **    *

Human           CCTCCTCCTCCTCCTCTTCCTCCTCCTCCCCGTCCTAC-TCTCCCTCCCCGTTGTCTCCG 155990
Gallid          CCAATTTAACAGAAATTAAAATATATTGATGTTGCGACATATGCATCCTCGCATATGGGG 164359
                **   *   *      *      *  *     * * ** * * * *** **        *

Human           CCCTCTCCCGTCTCTCCCTCGTCTCCCAGATCTCCGT-TCATCTCCCCCATTAGATCTCC 156049
Gallid          GTGGGACACAGGACGATTATATC-CCCAGACATGAACCTCAAACTGCCATTTTGATCCCA 164418
                      * *    *       ** ******  *     ***     **  ** **** *

Human           GGGACTCCGAG-CAAAGCCGCGGGTGTCCT-CCGGGCATCC-CGTGGCGTTCCCGCCGGC 156106
Gallid          TCATTGGAGAGACAAATTCGCATACATCCTACTTATCGCACACATTGGATGTCGGTCTTT 164478
                        *** ****  ***     **** *    *   * * * *  *  * * *

Human           GCCCTCGTCCGCGCCGCCCTTTTCCAAA--AGAGTCCCGTCCGT---TCCCTCATCG--G 156159
Gallid          ATTCAGG-CCATATCAGCTTTCACGGGGGCAAATTCGTATTCATAGATCCGTCATCGATG 164537
                   *  * **    *  * **  *      * * **   * * *   *** ******  *

Human           CATCTCCGTCCGCGCCGTGCATCGG-CAGGTCGCGGCCGCCCTCCG---CACAGACGGCG 156215
Gallid          CAGCGCCAAACCGGACATATGGAAGACAAAAAGAGAACCGGTTTGGAATCGCAGGGGACC 164597
                ** * **   * * *  *      * **    * *  *    *  *   * ***  * *

Human           TGAGACGCCGCGTGAGGTGACGGAGGCGGCGAAGGGACACGAGAAGAACATGCAGAAGAA 156275
Gallid          GAGAATGCTTTTTCAGATGGCAGAGATGGCAAAGATG---GATTGTTACATGAAGGAATT 164654
                    * **    * ** ** * ***  *** ***      **     ***** ** *

Human           CATGAAGACGAAGAAGACGAAGAAACGAGGACGAAAG--GAGGGGAATACACCGGAGACC 156333
Gallid          AATGAGCCCATTTTGATTCCGTCTACCATCGCAGATCTCGAGGGGATT----CGTGAATT 164710
                 ****   *               ** *   *  *    ******* *    **   *

Human           GAGCGGCGGATGGAGCCCGCTCGGTCTCGCACGTCCGCGATACCGAGCGGACTCCGTC-G 156392
Gallid          GGTCCGAAAATTCCGT--GGTCGTCTACTGCCCTTTGAAAAGTGTCCCGATTTTTGTCTG 164768
                *  * *   **   *   * ***    *   * *  *  *       **   *  *** *

Human           CCGCTCCGGACCCTCCACGCC--CCTCCGTCCAGGACCCGAGGTCCGACACGCCCCCACC 156450
Gallid          AGAATTGGGGGTTTGGAGGCCAGCTTTCATAAAGGGCAGGAGGAGCTGTTAGAGTAT-TG 164827
                    *  **    *  * ***  * * * *  *** *  ****  *     *

Human           TCGCGCACCTCCTCCGCCACCACCGCTGACAGTCACCGCATATCACCCCCATACACACCC 156510
Gallid          TGAAGCACTTTATTTACCACAACCTGTTAAGATGGAAATA-GTAGGCATTGTAGACGATG 164886
                *   **** *  *   **** ***  * *   *      *  *   *    ** **

Human           AGCAGCAGAGGCCGGCGCACACACACGCGCGGAGCACG--CACGAGAACACGCGAGACAT 156568
Gallid          TGCCATGTCTGGCAACGGGGATGCAATTACTCATTCTTGTTGCCGAGGGGGGAGAGGTAT 164946
                 **       * *  **   *  **    *  *         *        * ***  **

Human           CGGCCGCCGAAATTAACGGAGTCTATGCGCGCGCCGTGA----CACGGAAAACC----AA 156620
Gallid          ATGCCTATGAAGAAGATACTCTGCATAAGTTAGCCACGAGTTTTTCCGAATTCCTTGAAA 165006
```

```
                  ***   ***    *     *  **  *   ***  **      * ***  **    **

Human           GCGGAGCGAGACGATCGACCGACTCCTGCTATCCTTCCTCCCTGGGCACGGTCCACACGC 156680
Gallid          TTGGAGTGAAATCTTTAGGGAGGGAGGTTTACCATTGTGGAGAATATATAGAGCAAGTAG 165066
                  **** ** *   *              ** * **           *  *  **

Human           CAGTCTGCGGAGTCACCTGAGGGCCGGATCCGCTCCG--CGCCCCCCGCCCGATCCG--C 156736
Gallid          TACATTAGGGGCTGGGTTAAAGACCAAGTAATTTTTGACCGGATATCACGTGATGTAAAT 165126
                 *   *  **  *    * * * **    *    *  *  **     * *  ***

Human           CCCGATAAAAAAAAATCATCTGACTCG-TGCCAGTTCACACAGATGCAACCGATGACAAA 156795
Gallid          TCTAGCAATTATTGTTCCTAGCAGAAGATAAAAGCTGGTAGCTATATAATACAGGCCAAA 165186
                 *    **  *    ** *   *   * *   ** *   *   **  **   * * ****

Human           AAA---AAAACACACCACCACAAGCAAACGCTCGCCGGCCCGTTCACCGTCCCTCTCACC 156852
Gallid          GTCTCCAAATTACACTTGAGCAGAAAACCTGCTTTCGGCTCCATCGGAGGCAACATGAGT 165246
                      ***  ****     **   **  *      **** *  **   * *    * *

Human           CCCACTCGA-ATCGCGC-AGGCGCGTCGGCGGGCAGTCCCC--CGAACGCCT----CCAC 156904
Gallid          CGTGATCGAGATCGAGCCAGACCCGATACACGATTATCATCGTCAGATAATGAGAGCGAC 165306
                *    **** **** ** ** * **      *    **  *  *  *         * **

Human           GGCAACGCTCCTCCCACGCCCCCCCNNNCCCCCCTCCCTCCATCCCCCTCCCCGGAGCAT 156964
Gallid          GACGAAGATTATCAACTGCCACATTCACATCCGGAATATGGCAGTGACTCGTCCGATCA- 165365
                * * * * *  **    *** *        **      *        ***  * ** **

Human           ACACGCCGACTCTCTCCGCAGAGGCGGCGACCA-CAGCGGCAGCGGAAACTGTA-ACGGC 157022
Gallid          AGACTTTGAACTTAATAATGTGGGCAAATTTTGTCCTCTACCATGGAAACCCGATGTCGC 165425
                * **    **   *         ***         *  *  *   ******   *   **

Human           CACGTTCTTTGAACAGTGACAGTAACGGCGGCGGCGGCTGTCGCCGTCACGGTAGTCGTG 157082
Gallid          TCGGTTATGTGCGGATACAAACAAACTATTTCGATGTTTATTCGATGTCGACTAA-ATA 165484
                   *** * **   *   * *  ***     **  *  *    *  *  **       *

Human           GCGGAGCACCCGGGGAGGCGACGACGGGGACTACCGAT--GCTCTCAGATCATCGCCATA 157140
Gallid          GCGGTCCGTTCCACGATGCT-CTTCGGAGAGCACTATTCGATATTCATATGATTGGTCGA 165543
                ****  *   *   ** **  *  *** **  **   *      *** ** ** *    *

Human           GTGAAAGAGTACCGAATAAAACAAGTTAAATTTGTTGTAAATAAAAAAAAATACTATGT 157200
Gallid          ATG---GGATATCGACTAAAACAAGCCGAATGGGAAACTATCATGAATTTGACCCCACGC 165600
                 **   *  ** *** *********   ***       **     **    *  * * *

Human           GGAAATTAAAAATAAAAAAAATGAAAATAAAGGCTAAACACTGACTAAACGTGCATCCT- 157259
Gallid          CAAAGTCTACATCTGCGCAG--GACTCTGAGGGATGCTGATAGTCGAAGCGCCCATCCTA 165658
                  ** *  * *       *   **   * * ** *    *  * * ** **  ******

Human           TCTCCG-TACGTCCCCTTTCAATAA-ATGGCGGACCCGACCTCTCCATAAACGGAGAC-A 157316
Gallid          TATCCGATATATATGCCTCCGATAGCATTTTTCACCCAATCGCTGCGTCCTCGGGAACTA 165718
                * **** **  *   * * * ***  **     **** * * ** * *   ***  ** *

Human           CGCCGGGAGACTCCGGCCCTCC---------CAATCCACAGGTAGCCCGAGACCTA-ATC 157366
Gallid          TTTCTTCAGACTGCGATGTAAAAGGAATGAACGATTTGTCGGTAGACAGTAAATTGCATT 165778
                   *   ***** **                * **     ***** * *  *  *  **

Human           ACAAAACTGTACCTGAAACCGAAAGTAT-AGAGGGCGGCCTCGAATGCCCCACCTATGAG 157425
Gallid          AACTATCCAGACTTGAAGAGAAAGCTCTTATTATATAATTTTAATTGTTAGACATA-GAG 165837
                *   * *   ** ****    **  * * *          *  * **    ** ** ***

Human           GCGGGACTTCGCGACGCGCCACCCTTCCTATACGCCCGGCCTTCCG----------CGTG 157475
Gallid          CCGACATTCTTTGATCTATCTAATGAGATAAAATAATAGATTTTGGATTTATTTGTCATG 165897
                 **  * *    **     *        ** *      *  **  *          * **

Human           CGCGCCTATAGGACGCAT---CCCCTCGCCCCGAAACCGCAACGCGGCAGAGTCGGCGCC 157532
Gallid          ATCTGTTGCAACAAACGCTGACCCCCCCCATCCATGAAGGGGCGTGTCAAATAACGTGTT 165957
                  *   *  *  *  *     **** * *  * *    *   ** * ** *    * *

Human           ACACCCCCGTCCCCCGAGTTCGCGATGGGC-TGGCGCCGACCCTGCCGCGGC---AACGA 157588
Gallid          GCCTTTTTGTTGTATATGAAGATATTTAATGTGGCGTTGAGCCTAATGAGAGGAGAACGT 166017
```

```
                 *      **       *       *     *****  ** ***   * *     ****

Human           AGGCGAA-ACGAGACCCCGACGC-ATGCGGGCATAAAG------AGCGAGAAGTAAACGC 157640
Gallid          GTTTGAATACTGGAGACGAGCGCCGTGTAAGATTAAAACATATTGGAGAGGTATGGCCAT 166077
                    *** **  **  *   ***  **   *  ****        * ***   *   *

Human           GGGGTCCCGA-------ATTATCGCGAGACTATTTG--CCCGGGCCCGCCCTC------- 157684
Gallid          GTGGTCTCTACGGCGCAAATCTAGCAGGAGTGTGCAACTCCGGGTAGATTCTCCAAAAGA 166137
                * **** * *       * * * **  ** * *      *****      ***

Human           --GTCCTCATGCCCTTCCCCCCGCCCTCATCCCCCGACACACCCGGTGGAAAAAAC--GA 157740
Gallid          ACAGAGTTATGATATACTTTCTGCCGGCGGGGAACATGTTGCGCTATTGCCTAAATCTGT 166197
                      * ***   * *   * ***  *      *      * *  * *   ***   *

Human           AAGACACATAAATAAAACGAATTTTGTTGTTAAAAATGTTTCCACG--TGACGTCC---C 157795
Gallid          ACGCAGTCTAGCCAGGACCATATTAACCGCCGCTACGATCTCCCAGGCTGCTATGAAAGC 166257
                * *     **   *  ** *  **    *     *   * ***  *  **   *     *

Human           CGGTGATTCCCGGAGATGCGGCACGCGGCGG-----ATTCGCCCGTTTA--CATCGGGGT 157848
Gallid          TGGAAAACCACCATCGTCTCGTTTGTGGGGTGAGATATTCGACAGAATGACTGTCACGCT 166317
                 **  *  * *     *   *   * ** *      ***** * *  *     **  * *

Human           CCGCAGACGCGGT---TCTGCCAGGGCGTTGCGGT-GTCGGTTCCG-CACAGAACACCGC 157903
Gallid          TAACGAATATGATATTTCTGCTTCGCCATTCCACCCGACAGACCCGACGAGAAAAATTGT 166377
                   *  *   * *   *****   * * ** *    * * *  *** *    ** *  *

Human           GGTCCGTCGTCACCGTTCAACCGCGCGGTAGTTCCGCGATTCTATGGTTCATCTCCGCTC 157963
Gallid          AGGCCGGGCTTTACGGTGTATTGAACG-TGCTCCTCTTACACACGAAGAAATGGACACTC 166436
                 * ***   *   ** *  *  *  ** *  * *    *  *        **   * ***

Human           ACAGCGTTCTCTT--TCTTTTATTTTTCACTGAGCCGCTCCACCACCAAGAGTGTTAC-T 158020
Gallid          GGTTTACTATCATGATGTATTGGTGTTGTCTTGGACATGCTGGATACTGTACTGTTTCGC 166496
                       * ** *  * * **  * **  **  * *   *      *   * **** *

Human           GACTACACCGTATACATGTTT-TTTTATCTCTCCACCCCTCCCCATCACAGACCATCAAA 158079
Gallid          GCTTATATGAGAAGAATGTCCGTCTTATGGACATAGTAGGTTCGGCAACGGGCTGTGGAA 166556
                *  ** *    *   ****   * ****      *       *    ** * *  *  **

Human           CACCTTGCCGTATCGACACTGACTATACTCGACCGCGGATGACAACGGTCGTTGGTCACA 158139
Gallid          TAAGTCCACTCCCCGAAATAGAGTCTTATTGGAAACCTTTATGTCGTGCCGTCGCTACTA 166616
                 *  *   *    *** *  ** * *  * *    *   *       * *** * *   *

Human           ACAGAGCTACAGCGGACAGCAAAAATA--AGATAAATACAGTAAGAGAGAGGCCCCGGTC 158197
Gallid          AGGGGAATGCAGCAATCGGTGATGATGCTGAATTGGCACATTATCTGACAA------ATC 166670
                *  *   * ****   * *  *  **     **    *** **   ** *        **

Human           CACATAGGAAGCCAAGGGCGGACGAAAATAAAAACCCGTCCGTGATT-CACA--CACGAA 158254
Gallid          TTCGGGAATCGCCAACAGGAGACGGGGAATCCTACTTATAACTAATCGCACAATTATTAA 166730
                  *       *****  *  ****   *     **   *   * **  ****   *  **

Human           TCGGGGGTTATGACAA--CGCGACACACGCAGATGAGGGACGGACGCATCG----CGATC 158308
Gallid          TAGGATTTTAGGAAAAACTGCTACTAACGTTGTTTAAATAATAAAATTTTATTTTCAATA 166790
                * **   *** ** **   ** **  ***  * * *   *   *    *      * **

Human           CGGCGCGACGGGGC-GCGATTGGCCCACGCGCGAGCCCGTGCCCGCTTCGAG-TGGCTGC 158366
Gallid          AGGCATTACAGTGTTGTCATGATTGTATGTATTATATGGGGTATGCATGAGGATTACTTC 166850
                 ***   ** * *  *  **      * *    *    * *   ** *   * *  ** *

Human           TCCTGGCTCGCGG-CAGGCCGTCCAAACTGTACGGCTATACG--AGTCGGCACCGGGGAG 158423
Gallid          GATTGAAACTTTGTCTAAATGTCTGTAGGATTTTACTATTCATTAGTCTGGATCGAGGCG 166910
                   **   *   * *     ***   *   *    **** *   **** * * ** ** *

Human           AACGGATCCACCTACCGTGGCC------GCGGTACTGGTGCCTAGAAC--------TCCA 158469
Gallid          GACGTAAATGGAGATTGCGGCAAATGTAGGGGTGCTGGTACATAAGACCTCCAACATCCA 166970
                 *** *       *  * ***       * *** ***** * **  **        ****

Human           TCCGGATC---------CGTACAGGGACGCCAGGAGCGCCACCGTGTGGGGTCACCGCTG 158520
Gallid          TTCGACTCATCGGCCTGCGTCCAAATGGATATGTTGATGTACCTTGTAAAGTTATGACAT 167030
```

```
                * **  **          *** **          *  *    *** ***   ** *   *

Human           GGGTTGGCCGCCAACGCAC-GTGAGACCCAGATCCGTTCAAGACT--GCGGTGAGTGAGC 158577
Gallid          TAGAAGATCGATGGTGAATAGTGGGATCTATATCCATGCTATTCTCAATATTGCATGATA 167090
                  *  *  **      * *  *** ** * * **** * * *  **       **  ***

Human           GCCGCCGCGACCGACGACGCT--ATGCGCACACGGGCGAG-CGGCGGGGAGGGACGTCGG 158634
Gallid          TGCAATGTTCCCGGTTAGGTTTGATAAGATCATGTATGGTTCTATAATACAACTCCTCTT 167150
                  *   *   ***   * * *  **  *  ** *   *   *            * **

Human           CCGCAGGGCGAGCGGGTGGAGGGCGAGCGGGTGGAGGGCGAGCGGGTGGAGGGCGAGCGG 158694
Gallid          CAGAAGAATCATTTATTTTATGTCCA-CTGTCCTTGGATATTCCAGTTTCTGTCAATCGA 167209
                * * **    *     *  * * * * * *     **     *  **    * * * * **

Human           GTGGAGGGTGAGTGGGTGGAGGGTTCGTCGAAAAC--CACCGAAGACATTGGCTGGCGGA 158752
Gallid          TTCGCTTGCATTTGCGTGCAGCATGTCTTGATGGCATTTCCTATGCTATCATCCGGCAGG 167269
                 * *   *     ** *** **  *   * **   *    ** * *  **   * *** *

Human           GAGTGGGG-ACAAAACAACGTCACGTCAGGGGCG-GAGAAATATAGGGTGCGCAG--ACC 158808
Gallid          CCTAAGGGTGTTCTATACTCGCACACAGGTAGAGCAAGAACCACGGCATATCGAGCTACC 167329
                     ***      * *    ***    *  * *  ****  *  *  *    **  ***

Human           GTCAAAGCGCGAGTAACGGAAAACGAGTACGGATACGGGGGGGGGGGGGAGGAAACATGG 158868
Gallid          TCCATTGCCCCGCTAA-GGACATTTCTTGCAGACT---GTATTGTCATGAACATATTTCG 167385
                  **  ** *   *** *** *     * * **     *    *    **  * *  * *

Human           TGAAGTCTGTGACACGCGTGACC---GTCAGCCGGCAACGCCTGCCACGGCCTCCGGCCT 158925
Gallid          TGTA--TTGTGTCGATCATAACCCTTGTTGATTCCTATGGAAAGCATTGTGGTCCAGTTT 167443
                ** *   **** *   * * ***   **         *  *   **   *   *** *  *

Human           CACAGACGACTCACGGA-GTTTCCTCCAGACATTGTT-------TTTTTTTTTGTATTCA 158977
Gallid          TCCAGATGAAATGAAAACAATGCGGGCAAAAATGGTCCCACCTGTTTCATCTTCAATGCA 167503
                  **** **       *   * *   ** * ** **        ***  * **  ** **

Human           TCGGTAATGCGGTA---TACGTA-AATTCCCCGCAAAGGTACTTTCCGTTAAGGACGGTA 159033
Gallid          TCTCTCACATCCCAAGTTCTATAGAATATTCTCCACTGACCAGTTTCGGTAAGATCAGTT 167563
                **  * *      *   *   ** ***   *  **  *     ** ** ****  * **

Human           CAGGCGG----TATGACT-TCCGGAACACGATGATTACGGATATCCTGC---ATAGTGTA 159085
Gallid          TCTGTAAAATTTGTGATAGTTTCAATCGAAAACATTTTGTCCATCATGGCAAAAAATCTA 167623
                   *       * ***   *    * *   *  ***  *   *** **    * * * **

Human           CAGG-AGACC-GCTTTTTTTTAGGTTCCGTATAAG---ACGTACGTCGG-CGACGTAC-- 159137
Gallid          TAGGCAGACCAGATAACCATTTGACACCACATATCCTTGTGTATATCAAACGATGTAATA 167683
                 *** ***** * *     ** *   **  ***       ***  **   *** ***

Human           -ACACAACGGTTAAAAAAATAAAAAAAAGAGGAAACAGTAAAGCTC---CTCCTTTCCTC 159193
Gallid          GATCCCTCGTTAGTAGATATGGTACATAAAAGGCCTAATCTCTCTCGGGCTTCCATACAT 167743
                 *  *  ** *   * * **   * * * * *    * *    ***   ** *  * *

Human           TGTGTTATCGCGTTCAGTTAGTAGTCGCCATTGCTGTTGCGTATGCTTTTCGCGTTAACG 159253
Gallid          TGAACGATTCCTTCTGTGAATTCATCAACAACCACATGCCAAAAATTTACATTAGTAATC 167803
                **    **  * *      * *  **  **      *  *  *   **        ***

Human           GTACGCGACAGGATACGAATGCGT------------TACACGCCGCAAAGGCTCCCTACG 159301
Gallid          TTTCTCGGTGGCTTACCAAATCGTCCTCTTGGTATATCCATATCATCGAACATTGTAGCA 167863
                 * * **   *  *** **  ***              * **   *    *   *     *

Human           ATGACTCTATGCGGCAT---CTCCCGTTCGCGCAGAAACGC--GAGTTCCCGGGACGCCA 159356
Gallid          TTGACTCTGCTCATCGTTGTCTTTCAAATGCGCTCGATTGTTGAATCTCTCCTGATGTTA 167923
                 *******   *  * *    **  *    ****   *  *    *  ** *  ** *  *

Human           GTCGTG-ACGTCCGGCCGATGGGATGCCTTTGCG-TGTGCGGATGTTTTCTGCTGTTTTA 159414
Gallid          GAAGTATATGGAAGATAGCCTGGATACATAAGTGATCTAGAAGGGTTTGTTATTGCACTA 167983
                *  **  * *   *   *   **** * *  * * * *       ****  *  **   **

Human           ---TAGGAACA----GAGACA----AGAGACGAAAATAGCGGAACGCCCACT-ATAACGT 159462
Gallid          ATATACAAATTATACGTGACACTATAGCGACGGTTGTAGCGATGCACCTAATCGTAATGT 168043
```

```
                      **  **       * ****    ** ****    *****   * ** * *  *** **

Human           GCATTTTTCTC--TGTGTT-TTGCGTTTTCATGTTTTATGATTCTTCTACGGTATCGGCC 159519
Gallid          GTATACGCCCCATCATGTAATTATATCTAATTGGTAGCAAGTAGGTCTGTCGAATAACAG 168103
                * **    * *    ***  **   * *   ** *       *   ***    * **

Human           GGAACGGTCATC----CTCGATCCTTCTCGTACGCGTG----TCCCGCCCCAGCC----- 159566
Gallid          CTAATGACTACCGGCTCTACATTTTTTCTGTATTCGTGACTTTCCTGTCGCAGTGTAACG 168163
                  ** *   * *    **  **  **   ***  ****    *** * * ***

Human           --CTGGACTCGAGTCTGTACGTATGTTGCGGATACGGAGAAAAACTTCA------ACCCG 159618
Gallid          AACCGGAATTGCAATCGCATCTCTATCTTCTTTCTTGCAACATTTTCCACAACAGAATAA 168223
                  * *** * *     * *  * * *       *   *  * *   * **       *

Human           TGGGCTTCGTAAGCT-CGTATCTGACCCACTCCCCGCTCGACACGCTTCGCGTGCTCCTG 159677
Gallid          TCTGCCGGGTGTACTACTCATTTGAGGTGGTTCGATTTCCGGAGGTTTTAGAGGATTGGG 168283
                *  **   **   ** *  ** ***      * *    **   * * **     * *   *

Human           GTCGGTAGAGACGGAGCCGTGTACGTCCACCACATGAGGGCGGCCAGA-----CTCTGCC 159732
Gallid          TGGGGACCCGAGGATTTTGTATACACATACCATATCACTGTCGCAAAAATGCGCTCTATC 168343
                   **    ** *     ** ***    **** ** *  *  ** * *     ****  *

Human           GACTGG--CGTCGAACGTA-------ACGGAGTTTGCAAGG-----CGAGGGCTGCAGCG 159778
Gallid          TTCTGGGGTGTCGAACTTCGGTTCCCATGTAGATGTCAAGAGAGTTTGAATATTGTCGGG 168403
                  ****   ******* *         * * ** *  ****       **    **  * *

Human           GG-ACCCCGTGGCGTATGAGG--AGGACCTAGAGCTGCCGG-----ACCGGCGTATGTGC 159830
Gallid          AATGGCCCACGGCATACCGGACCAGGTCCCAGACACTTTGATTGCAAGTAACCTTTTTGG 168463
                     ***  *** **   *   *** ** ***       *       *    * * * **

Human           GGAACGAA--CGTCAGACATCTGTTCGACGTG-----ATCGCCGCGGCCGCCGACGAAC- 159882
Gallid          CAAAGGAATACATTCGAGCGCAATGCGACATATATCTGCCGCCCCAACTATCCACAAGCT 168523
                  ** ***  * *  **   *  * **** *        **** *  *   * ** * *

Human           --ACGACCTGCTGACCG-----TCGGCGGCCTGTGTCAAACGCAC-GCCGGAGTGAGCTG 159934
Gallid          ATGTGGAGCATTACCAGAAACTTCAGATTCCAACATCAAATATCCAGATAGAACATCCTG 168583
                    *      *  * *     ** *   **    *****    * *   **     ***

Human           TGAATTACTAGAG-ACCGTGCGAGATCCGTGGACGGCGGTTCCG--GGCGTACGCATGAC 159991
Gallid          CCATTCTGTGGAACATCCTGCAACATCTTCAAATAGCCGCACTATAAACGAATCCCTAGT 168643
                  * *   * **  * * *** * ***     *  ** *  *      ** *  * *

Human           TCTGACC------GTGGCGCGGGCTC-AGTATCGCTTG-TGGCC---------------- 160027
Gallid          TCCGGCCAATCCGGTACCACGAACTCCAGTTCCATCTGGTGGCTTTGTCCTTACTATCGG 168703
                ** * **      **  * **  *** ***  *   ** ****

Human           -CGATGC--CCGGAGACAGCTCCGCCTGCACCTGT--ACGCGGGACACCCCC-TGGGACC 160081
Gallid          TCGATGTTGCCGAGGAAGAATTAACATGGGTTTGGCGAAACGGAATAGGTCTGCAGCTCT 168763
                 *****   ***  **    *   * **    **   *  *** * *   *    *  *

Human           GTGGATAGTGTGCGCCGTTCTGTC-TCGAGAGAGGGAGACGCAGACGCCGTCGCCTCCGA 160140
Gallid          GACGATTATGGGCACACCCACATCATCCTGTATTTGTTCCATACATTGCTTTATAAGGAA 168823
                *  ***  ** ** *        ** **  *      *   *  * *   * *        *

Human           TAGGCA--GCGGAGGCGTGAT-TCTGGGAAACGTGCCCACG-CCGAGGCCACGCGAAGTG 160196
Gallid          TATCCATAAAGTAGATGCAGCATCTCTAGATCTTCCTGGCAATCGATCGCATTCATCTAG 168883
                **  **    * **  *      ***    * * * *   *   ***   **  *      *

Human           GAG-ACGGCTTGGGTGATCGT-----CACCC--TGGCGGGACCGCTGTTATCGTTCTGGC 160248
Gallid          AAGTGTGACTATAGTTATCATGGACACACCCATCTTCACCTCCACCAATAATCTTTTTTA 168943
                 **   * **   ** *** *     *****     *    ** *   **   ** *

Human           CCGATAACGGCAAGATCTGCCGTCTGGCGAACTCGTTCGCCGCCCTGTGGAGGATGGGGC 160308
Gallid          TTGTTAATAACTGGGCCGGTCTGATCTCCAAATCTTATAC--TCTGGTAGAATATGAAAC 169001
                  * ***   *  *  * * *   *  * ** ** *   *   *  ** **  ***   *

Human           CGCG---GGCCATGAGAGGACATTGGACGTATTCGGCCCCGGGTAGACATCTCCCCGGGG 160365
Gallid          AGGGTTAAAACTAGGTAATAGACTGGATGTCTTCGAGTCCGG--AGGCAGAAACGATGGA 169059
```

```
                * *      *  *  *  * * **** ** ****   ****  ** **    *   **

Human           ACGCTTGGCCACTCTGT-GAACACGTGAGACCGCCAGTCGGAAAACTTCCCCGGCAAAGA 160424
Gallid          ATGCGGCATTTCTTCGTCGAAAGTACACGACTCTAAAACTAATACTACCTACGGAATTAT 169119
                * **       **  ** ***       ***    *  *  * *    *  *** *

Human           GCGTACCTGGATTAGACGCCACGGCGGCGACGTAAG--GATGCGACCTACGGCCAGAGAC 160482
Gallid          ACATAACAGCATCA-ATGGTACGGATACGACGTTGTTTGATACTTTTCCCGACAGTACCG 169178
                 * ** * * ** * * *  ****   ******     *** *      ** *   *

Human           ATGAC----------CGGAGGAAGATCATGACAAGGATCTCGAAATATAAAACCAAAAAA 160532
Gallid          ATAACGCGGAAGTGACGGGGGATGTGGACGATGTGAAGACTGAGAGCTCTCCCGAGTCCC 169238
                ** **          *** *** *   * **    * *    ** *  *    * *

Human           AAAGTGAGGAGTGCGTAATCGCAAGAAAAGAAAAAGCAGAGCCGCGGTGGAAATGTCTAT 160592
Gallid          AATCTGAAGATTTGTCACCTTTTGGGAACGATGGAAATGAATCCC--CCGAAACGGTGAC 169296
                **  *** ** *     *        * ** **    *    **   * *     **** *    *

Human           CCGCCTGCGTGTATGTGTGGGGTGTGTATATGTGCGCGTGACCGTATCCCTCCCTGCCTT 160652
Gallid          GGACATTGATGCA-GTTTCAGCTGTGCGAATGCA-GTATAACATTGT--TTCATCGTTAT 169352
                  * *   ** * ** *  * ****   ***    *  * **  * *    **    *    *

Human           TAACCCGATGAGAAATAAA-ATCGGCGAATGTACACAGTCAC------ACACAATGTGCT 160705
Gallid          CGCCCGGATCTGAAGGGTATATCTATGTTTGTACAAAGCGTGGGGATAATACCAAGAGAA 169412
                   ** ***  ***     * ***    *  ****** **          * ** * * *

Human           TACGTGCCGTGTTACTGACATCACATAACAAAAA----AAAAACATACTTCCTGTCCGTG 160761
Gallid          AAGTCATTGTGAAAGCTGTGACTGGTGGCAAAACCCTTGGGAGTGAAATTGATATATTAA 169472
                 *      ***  *        *    *  *****         *    * **  * *

Human           AGACACCGCCGCCCTCTAGCGGGGTTGCTATAGCAGCGCCCGATGCCAACGCGACACGGT 160821
Gallid          AAAAAATGTCTCACCGCTCCATAATTAGATTAGTTCATGCTTATA--GATGGAAATCGAC 169530
                * * *  * * * *      *     **     ***       *  **     * *  *  **

Human           GAGTCGCATAGATCGGGACTGCTTGAAAGCGCGCCACATTCGTCTTTTATATAGACACCC 160881
Gallid          AGTTTGTAT-GGTAATGCCTAAATACAAATGCGACTTGTTTACGT---ACATAGATATCA 169586
                   * * ** * *   * **    *  **   *** *    **     *    * ***** * *

Human           CGGGG--AGTGGGAGGAGCCAACATACACACGAGGTCGGTCTGGGGTTGGAGACGAACGC 160939
Gallid          TGGGACCATTGCCACTAAATCAAATAATTACGA---TAGAACGGGGTTTGCTTGGAGCAT 169643
                 ***   * **  *  *     * ***   ****       *   ****** *     ** *

Human           GGAAAATCAACGGCGTAAAAAAAAATAAAAAAGGCGGAGACACATAGCCTTGGCGGGGAGA 160999
Gallid          TGGCATATATC--CACGAAAAGGGTATAATACATCGTGATGTAAAAACTGAAAATATATT 169701
                 *  *   * *  *    ****   ** ** *     * **   * *  **         *

Human           CGATAACAGGTTCAAAGATAGAGAAAAAAAAAAAAAGAGGAG-ACCATA-ACATTGAATAC 161057
Gallid          TTTGGACAAACCTGAAAATGTAGTATTGGGGGACTTTGGGGCAGCATGTAAATTAGATGA 169761
                     ***       ** **  ** *       *     ** * * ***  * ***  **

Human           ACGTGCGGCGGCAATCCAATCAACGGGACGTGCCGGGGCGCAAAAAA--AAGTATACGGA 161115
Gallid          ACATACAGATAAACCCAAATGTTATGGATGGAGTGGAACTCTGGAAACCAATTCGCCTGA 169821
                ** * * *     *  * ***     *** *     **  * *   ***  ** *   * **

Human           AGTGCATGC----GACAGACAGT-CACGCGGACCGACG------ACAAAGGCCGACTCCT 161164
Gallid          ACTGCTTGCACTTGATCCATACTGTACAAAAACTGATATATGGAGTGCAGGATTAGTTCT 169881
                * *** ***    **   * * *  **    ** **            ***   * * **

Human           AGCATGGCAACTAGGAAAAAAAAATAAGCAACGCTGCAAAATACTAACAAAGGAAACATCC 161224
Gallid          GTTTGAGATGTCAGTAAAAAAATATAACCTTTTTTGGCAAACA--AGTAAACGGCTCAGGT 169939
                      *      ** ****** **** *      **  *** *  *  *** *   **

Human           CTTCACCCC---TCCCTACCCCATCTACCACAACCCCCCCCTCCGTAATCCCTTCTGTCC 161281
Gallid          TCTCAGCTGAGATCCATAATTAGATGCCTGCAAGTCCATCCGTTGGAATTTCCACAGAAC 169999
                  *** *     *** **          *  ***  **  **   * ***  *  * *  *

Human           GCGTTTCTCCCACAGGCGCGTGCACTCGCAGACACGCAGGCACGCACACACCACCTCTAT 161341
Gallid          A-ATTCTACAAACTTATGCAAACACTT-CAAGCAGTACGCGATTCAGTTACGACATCCAT 170057
```

```
                   **   *  **    **   ****  **  **    *  *  **   ** ** ** **

Human           G-GCAGTCGC---GGGCGGGCAGGCGGGGAGCATACGGGGGGCAGATGTAAAGACAATGA 161397
Gallid          ATGCAATCCCTCAGATTATACGAAAGAGTGGTATGACGATGGATCTTGAATATGCTATTG 170117
                  *** ** *   *      *    * *  * **   *  **    ** * *  * **

Human           GGAACGGCATAGCGCGCGACGTGCCGCC---GTCTCGGACCCTTGCTATTCTGGCACGAC 161454
Gallid          CAAAAATGCTCACATTCGATCAGGAGTTTAGACCATCTGCCCAAGATATTTTAATGTTGC 170177
                  **     *  *   ***   *  *       *     ***  * **** *       *

Human           GCCA-------AGGGAAGCCTCTGGCGCAATCTA-ACCCTAACCCTAACCCTAACCCTAA 161506
Gallid          CTCTTTTTACTAAAGAACCCGCTGACGCATTATACACGATAACTGCCGCTCATATGTAAA 170237
                  *        *  *** ** *** **** * ** **  ****     * *  *    **

Human           CCCTAACCCTAACCCTAACCCTAACCCTAACCCTAACCCTA-ACCCTAACCCTAACCC-T 161564
Gallid          CACCCGTC--AAAAATAACTTCAATGATTCATTTTATAATATATACTACGCGTTACCTGC 170295
                * *    *  **   ****   **   *     * *   ** *  ***  * * ***

Human           AACCCTAACCCTAACCCTAACCCTAACCCTAACCCTAACCCTAACCCTAACCCTAACCCT 161624
Gallid          AATAATGACAACATTCGAAGTCTTTGAAGATTCGCAGACCTTTTTTGCGAATGGCACCTT 170355
                **   * **   *  *  *  * *        * *  *** *       *     *** *

Human           AACCCTAACCCTAACCCTAACCCTAACCCTAACCCTAACCCTAACCCTAACCCTAACCCT 161684
Gallid          ---CGGGACCTACGCCATATTCCCA---CAGACCGCAAATAAAGCATTATGGAACATTTT 170409
                   *   ***    ** **  ** *   *  ***  **    * *  **      *   *

Human           AACCCTAACCCTAACCCTAACCCTAACCCTAACCCTAACCCTAACCCTA-ACCCTAACCC 161743
Gallid          TGGATTG-CATGAGATATACTCTAAACGATGAGAGTAAGGTAGATGATAGATGTTCAGAC 170468
                     *  *   *    **  *  ***  * *   ***     *   ** *   * *  *

Human           TAACCCTAACCCTAACCCTAACCCTAACCCTAACCCTAACCCTAACCCTAACCCTAACCC 161803
Gallid          ATACATAACTCCTTAGCACAATCCAA--TGTTACTTCAAGCATGTCTGTAATGAACGATT 170526
                  **   *  *** * *  ** ** *    * **   ** * *  *  ***

Human           TAACCCTAACCCTAACCCTAACCCTAACCCTAACCCTAACCCTA-ACCCTAACCCTAACC 161862
Gallid          CGGAAGAATATCCATTAATAAATGGACCTTCGATGCAGGCAGAGGACCCTAAAAGTGTTT 170586
                       *   * *    ***    * *    *  *   *     *******   *

Human           CTAACCCTAACCCTAACCCTAACCCTAACCCTAACCCTAACCCTAACCCTAACCCTAACC 161922
Gallid          TTTATAAAGTTCGTAAGCCTGACCGAAGTCGTGATTTT---TCATGGCAAAATCTGAACT 170643
                 * *       * *** *** ***  *  * * *   *    *    *  ** *  ***

Human           CTAACCCTAACCCTAACCCATCCCCCAACGCGCGCGCGCGCGCCTCTATGGGAGGCGCCG 161982
Gallid          CCCATGGCAATAGTGGTCTACGTCGTGAAAAATATATACGTTCCTCTA--AGAGGCGATG 170701
                *  *    **   *   * *   *   *          **  ******   ******  *

Human           ------------------------------------------------------------
Gallid          GAAGAATCCCGAGATATTTAAGGTATCTTTGAAATGTGAATCAATTGGCGCTGGTAACGG 170761

Human           ------------------------------------------------------------
Gallid          AATAAAAATTTCATTCTCATTTTTCTAACATTATAATATATCAGATCGTTTCTTATATAC 170821

Human           ------------------------------------------------------------
Gallid          TTATTTTCATCGTCGGGATATGACTAACGTATACTAAGTTACAAGAAACAACTGCTTAAC 170881

Human           ------------------------------------------------------------
Gallid          GTCGAACATAACGGAAATAAAAATATATATAGCGTCTCCTATAACTGTTATATTGGCACC 170941

Human           ---------
Gallid          TTTTAGAGC 170950
```
