# Supplementary material for: Investigation of a herpesvirus outbreak in mixed breeds of adult domestic ducks using next generation sequencing
Source: PLoS One. 2023 Jan 27;18(1):e0280923. doi: 10.1371/journal.pone.0280923 (PMC9882916; doi:10.1371/journal.pone.0280923)
Supplement: S4 File — (DOCX) [file pone.0280923.s004.docx]

**Highlights**

- Report of a fatal outbreak of Marek’s disease virus (MDV) in domestic ducks
- Its clinicopathological features suggested a betaherpesvirus as the pathogen
- Next generation sequencing identified telomeric repeats (TMR)
- Megablast identified a Gallid alphaherpesvirus type 2 (GAHV-2) as the pathogen
- Presence of dead flamingo 3 days prior to outbreak suggests species jump into ducks
