# Supplementary figures and images for: Investigation of a herpesvirus outbreak in mixed breeds of adult domestic ducks using next generation sequencing
Source: PLoS One. 2023 Jan 27;18(1):e0280923. doi: 10.1371/journal.pone.0280923 (PMC9882916; doi:10.1371/journal.pone.0280923)

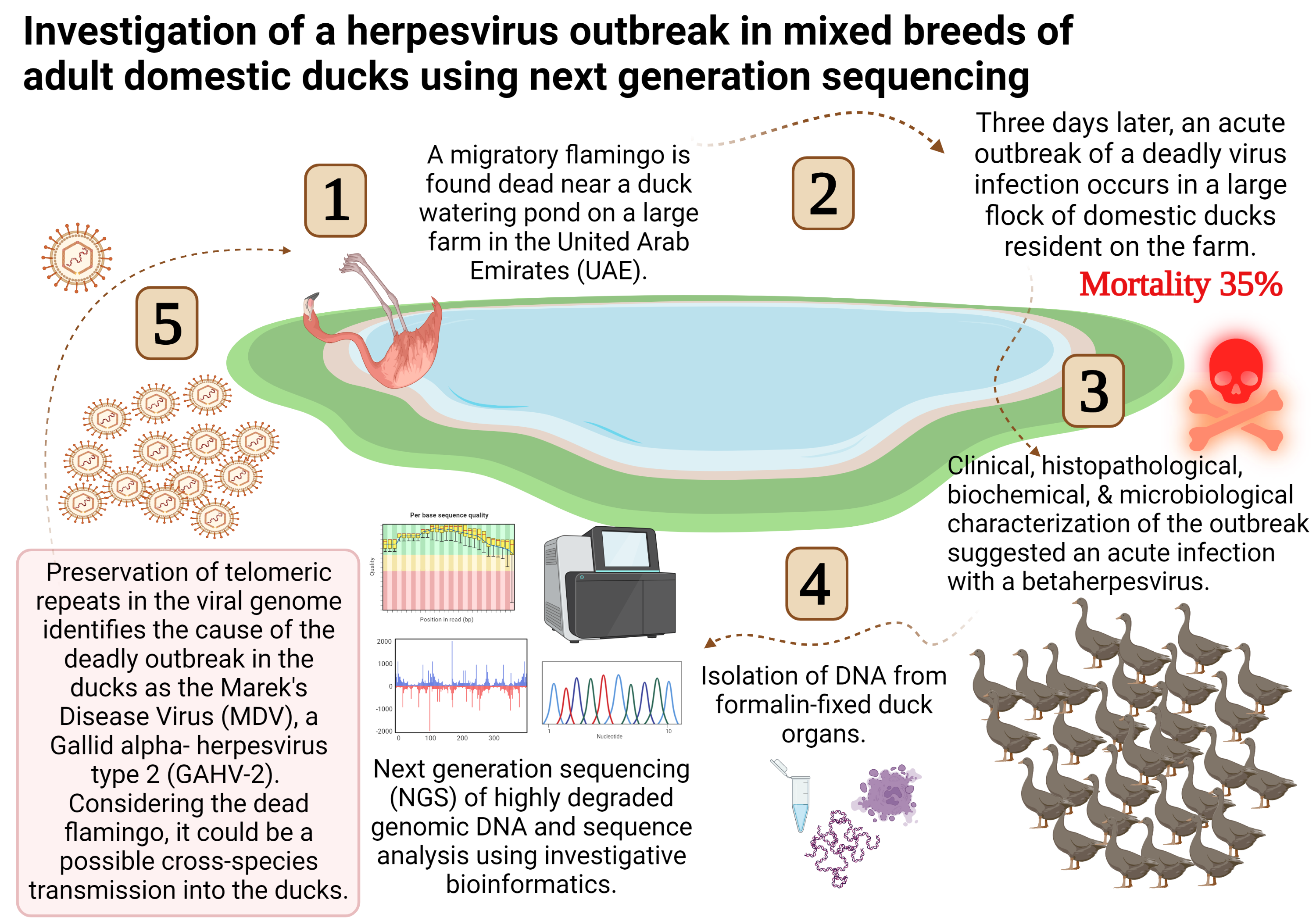

Supplement: S1 Graphical abstract — (TIFF) [file pone.0280923.s005.tiff]
